# Study on the Anti-demyelination Mechanism of Bu-Shen-Yi-Sui Capsule in the Central Nervous System Based on Network Pharmacology and Experimental Verification

**DOI:** 10.1155/2022/9241261

**Published:** 2022-07-12

**Authors:** Zheng Zha, Yi-Jiang Liu, Si-Si Liu, Nan Zhang, Jun-Ling Li, Fang Qi, Liang-Yun Jin, Bing Xue, Tao Yang, Yong-Ping Fan, Hui Zhao, Lei Wang

**Affiliations:** ^1^School of Traditional Chinese Medicine, Beijing Key Lab of TCM Collateral Disease Theory Research, Capital Medical University, Beijing 100069, China; ^2^Core Facility Center, Capital Medical University, Beijing 100069, China; ^3^Beijing Tian Tan Hospital, Capital Medical University, Beijing 100070, China

## Abstract

**Methods:**

The potential active ingredients and corresponding potential targets of BSYS Capsule were obtained from the TCMSP, BATMAN-TCM, Swiss Target Prediction platform, and literature research. Disease targets of CNSD were explored through the GeneCards and the DisGeNET databases. The matching targets of BSYS in CNSD were identified from a Venn diagram. The protein-protein interaction (PPI) network was constructed using bioinformatics methods. Gene Ontology (GO) function and Kyoto Encyclopedia of Genes and Genomes (KEGG) pathway enrichment analyses were performed to predict the mechanisms of BSYS. Furthermore, the neuroprotective effects of BSYS were evaluated using a cell model of hydrogen peroxide- (H_2_O_2_-) induced cell death in OLN-93 cells.

**Results:**

A total of 59 potential bioactive components of BSYS Capsule and 227 intersection targets were obtained. Topological analysis showed that AKT had the highest connectivity degrees in the PPI network. Enrichment analysis revealed that the targets of BSYS in the treatment of CNSD were the PI3K-Akt and MAPK signaling pathway, among other pathways. GO analysis results showed that the targets were associated with various biological processes, including apoptosis, reactive oxygen species metabolic process, and response to oxidative stress, among others. The experimental results demonstrated that BSYS drug-containing serum alleviated the H_2_O_2_-induced increase in LDH, MDA, and ROS levels and reversed the decrease in SOD and mitochondrial membrane potential induced by H_2_O_2_. BSYS treatment also decreased the number of TUNEL (+) cells, downregulated Bcl-2 expression, and upregulated Bax and c-caspase-3 expression by promoting Akt phosphorylation.

**Conclusion:**

BSYS Capsule alleviated H_2_O_2_-induced OLN-93 cell injury by increasing Akt phosphorylation to suppress oxidative stress and cell apoptosis. Therefore, BSYS can be potentially used for CNSD treatment. However, the results of this study are only derived from in vitro experiments, lacking the validation of in vivo animal models, which is a limitation of our study. We will further verify the underlying mechanisms of BSYS in animal experiments in the future.

## 1. Introduction

Multiple sclerosis (MS) and neuromyelitis optica (NMO) are primary central nervous system demyelinating diseases (CNSD) [1], which are the most common causes of neurological disability in young people [2]. MS and NMO are chronic neurological diseases characterized by demyelination and axonal injury of the central nervous system (CNS) [3]. They may present with a combination of limb weakness, visual impairment, fatigue, and ataxia [4, 5]. Thus, MS and NMO exert a heavy burden on individuals and society. Besides, it has been shown that persistent apoptosis of mature oligodendrocytes in lesion foci results in progressive demyelination [6]. Modern medicine uses immunosuppressants and immunomodulators as the primary treatment options for CNSD [7], aiming to alleviate neuroimmune activities. However, the overall outcome is not satisfactory. Therefore, there is a need to explore more effective and safer therapeutic agents to reduce the apoptosis of oligodendrocytes, which induces chronic demyelination in patients with MS and NMO.

Because of multicomponent, multitargeting advantages coupled with few side effects associated with traditional Chinese medicine (TCM), it has received increased attention as a potential therapeutic option for neurodegenerative diseases [8]. TCM has been shown to exert significant therapeutic effects in treating CNS demyelination [9, 10]. TCM not only results in less severe symptoms and fewer relapses but also avoids the side effects and adverse reactions associated with immunosuppressants, which has led to its long-time application as an adjuvant therapy in China. For instance, Bu-Shen-Yi-Sui Capsule (BSYS) has been used in the clinical treatment of MS [11] and NMO [12] for more than a decade and has yielded favorable curative effects in improving survival and quality of life. BSYS consists of several Chinese medicines which include *Rehmanniae Radix* (Dihuang, DH), *Polygoni Multiflori Radix* (Heshouwu, HSW), *Rhei Radix et Rhizoma* (Dahuang, DAH), *Leonuri Herba* (Yimucao, YMC), *Fritillariae Thunbergii Bulbus* (Zhebeimu, ZBM), *Hirudo* (Shuizhi, SZ), *Scorpio* (Quanxie, QX), *Gastrodiae Rhizoma* (Tianma, TM), and *Forsythiae Fructus* (Lianqiao, LQ). Our previous studies demonstrated that BSYS ameliorates axonal damage by inhibiting NogoA/NgR and RhoA/ROCK signaling pathways in experimental autoimmune encephalomyelitis (EAE) mice [13]. In addition, BSYS plays a neuroprotective effect by regulating the polarization of T cells [14] and microglia [15] to alleviate demyelination. However, TCM often contains different components which may have multiple targets, thus conferring a wide range of pharmacological activities. Therefore, the neuroprotective effects of BSYS and its underlying mechanisms in treating CNS demyelination remain to be elucidated.

Network pharmacology strategy may assist in studying complex TCM compounds and provide a method for exploring the therapeutic mechanisms of TCM [16]. Network pharmacology is a novel discipline that integrates systems biology, polypharmacology, and computational network analysis [17]. It is a novel method used to study interaction networks encompassing drugs, diseases, and target genes. A comprehensive network analysis was successfully conducted to uncover potential mechanisms of drugs at a systemic level [18]. Similarly, TCM network pharmacology presents a comprehensive method of exploring complex relationships between the active components of the TCM and disease targets, suitable for studying TCM-related issues.

In the present research, network pharmacology was employed to uncover the potential mechanism of BSYS against CNS demyelination, which suggested that the antidemyelination effect of BSYS is related to modulating Akt-mediated oxidative stress. In this study, the injured model of OLN-93 cells was established by H_2_O_2_ to simulate the occurrence of demyelination induced by oligodendroglial oxidative damage. The model showed increased oxygen free radical, abnormal mitochondrial membrane potential, downregulation of the Akt pathway, and oligodendrocyte apoptosis. So, we measured the levels of oxidative stress and apoptosis markers in OLN-93 cells after BSYS treatment. The current study is aimed at investigating the neuroprotective effects of BSYS in OLN-93 cells with H_2_O_2_-induced oxidative injury and verifies the Akt signaling pathway involved.

## 2. Material and Methods

### 2.1. BSYS Bioactive Ingredient Collection and Target Gene Prediction

The TCM system pharmacology database and analysis platform (TCMSP, https://tcmspw.com/tcmsp.php) [19] and Bioinformatics Analysis Tool for Molecular Mechanism of TCM (BATMAN-TCM, http://bionet.ncpsb.org/batman-tcm/) [20] were employed to search for potential bioactive components in the BSYS Capsule. The screening criteria for the ingredients were set based on the recommendations from the TCMSP, which include oral bioavailability (OB) ≥ 30% and drug similarity (DL) ≥ 0.18. However, based on relevant literature reports, we included some other bioactive ingredients in the BSYS Capsule with actual targets but with low OB or DL. Target genes were predicted using TCMSP, PubChem database (https://pubchem.ncbi.nlm.nih.gov/) [21], and SwissTargetPrediction database (http://www.swisstargetprediction.ch/) [22] according to the molecular structure information of compounds.

### 2.2. Identification of Disease Targets and Network Establishment

The CNSD-related targets were integrated using the search term “Central nerve system demyelination” from GeneCards (https://www.genecards.org/) [23] and DisGeNET database (http://www.disgenet.org/) [24]. We outlined the BSYS gene targets and CNSD disease-related targets, and then their intersection genes were visualized via the Venn diagram tool (http://www.bioinformatics.com.cn). Thereafter, we selected the intersecting genes, which might be the potential targets of BSYS Capsule in CNSD. To further define the interactive relationship between the bioactive ingredients and potential targets, we employed Cytoscape 3.7.3 software [25] to construct the BSYS-ingredient-target network, intersecting target-BSYS targets network and target-pathway network. Besides, based on intersecting targets of the BSYS Capsule and CNSD, protein-protein interaction (PPI) networks were constructed on the Metascape platform (http://metascape.org/gp/index.html) [26]. Modules of the PPI network were divided into different clusters based on the Molecular Complex Detection (MCODE) algorithm. Furthermore, to select crucial nodes, this study evaluated the topological properties of the nodes in the interaction network by calculating the “degree centrality” parameter.

### 2.3. GO and KEGG Enrichment Analysis

The intersection genes were analyzed by *R* packages for Kyoto Encyclopedia of Genes and Genomes (KEGG) pathway enrichment. On the other hand, Gene Ontology (GO) was analyzed by the DAVID database (https://david.ncifcrf.gov/) [27]. The GO enrichment involved three categories: biological process (BP), molecular function (MF), and cellular component (CC). An adjusted *P* value <0.01 indicated significance in the GO and KEGG analysis. The top 10 GO items (including BP, CC, or MF) and the top 20 KEGG pathways were visualized in a bar chart and bubble plot, respectively, using a web-based bioinformatics tool (http://www.bioinformatics.com.cn/).

### 2.4. Preparation of BSYS Drug-Containing Serum

BSYS Capsule was provided by Asia-East Biopharmaceutical Co., Ltd. (Beijing, China). We employed previously described steps to obtain the BSYS drug-containing serum (BSYS serum) and blank serum [28]. Adult Sprague-Dawley (SD) rats weighing between 180 and 220 g were maintained in a specific pathogen-free condition. BSYS was intragastrically administered to the SD rats (11.7 g/kg body weight) twice per day for one week. On the seventh day, the rats were sacrificed after gavage feeding for 2 h, and then blood samples were collected and centrifuged to obtain serum samples. The control rats were administered with the same amount of water to provide blank serum.

### 2.5. Cell Culture and Treatment

An oligodendroglial cell line, OLN-93, was purchased from BeNa Culture Collection (BNCC, Beijing, China). The OLN-93 cells were grown in DMEM with 10% fetal bovine serum (Corning, New York, USA) and 1% penicillin-streptomycin (KeyGen, Nanjing, China) at 37°C in a 5% CO_2_ humidified atmosphere. The OLN-93 cells were stimulated with100 *μ*mol/L H_2_O_2_ for 12 h [29]. After medium replacement, the cells were treated with BSYS serum for 24 h. LY294002 (CST, Danvers, USA) was dissolved in dimethyl sulfoxide. The OLN-93 cells were preincubated with the LY294002 (25 *μ*mol/L) for 1 h [29] before administration of H_2_O_2._

### 2.6. Cell Counting Kit- (CCK-) 8 Assay

The OLN-93 cells were seeded in 96-well plates for 24 h, stimulated by H_2_O_2,_ and then treated with BSYS drug-containing serum (5%, 10%. 15%, 20%, and 25%) for 24 h. Cell viability was assessed by the CCK-8 kit (Dojindo, Kumamoto, Japan), following the manufacturer's instructions. Absorbance was measured by a microplate reader at 450 nm.

### 2.7. Measurement of LDH, MDA, and SOD in OLN-93 Cells

OLN-93 cells were seeded in 24-well plates for 24 h. 100 *μ*mol/L H_2_O_2_ was added, and then the cells were cultured for 12 h. 15% BSYS serum was added and incubated for another 24 h. Cell lysates were collected and used to determine malondialdehyde (MDA) and superoxide dismutase (SOD), while the obtained medium was used to measure lactate dehydrogenase (LDH). The relative levels of LDH, MDA, and SOD were determined according to the instructions of corresponding commercial kit (Beyotime, Shanghai, China).

### 2.8. Evaluation of Apoptosis

The Hoechst staining was performed using a ready-to-use Hoechst dye solution (Solarbio, Beijing, China). Briefly, after discarding the culture medium, the OLN-93 cells were incubated with Hoechst staining solution at 37°C for 20 min in darkness. The cells were washed in PBS and then analyzed under a fluorescence microscope. A one-step TUNEL Apoptosis Assay kit (Beyotime, Shanghai, China) was used to perform the TUNEL assay, following the manufacturer's instructions. Briefly, the OLN-93 cells were fixed, permeabilized, and then incubated in darkness with the TUNEL reaction mixture at 37°C for 1 h. The nuclei were counterstained with DAPI (Bioss, Beijing, China).

### 2.9. Determination of Intracellular Reactive Oxygen Species (ROS)

Total levels of intracellular ROS were assessed by ROS Assay Kit (Beyotime, Shanghai, China). Briefly, the cells were labeled with 10 *μ*mol/L 2′,7′-dichlorofluorescein diacetate (DCFH-DA) at 37°C for 20 min. Then, the ROS was imaged with a fluorescence microscope.

### 2.10. Detection of Mitochondrial Membrane Potential (MMP)

Alterations of MMP in the OLN-93 cells were assessed using an enhanced mitochondrial membrane potential assay kit with JC-1 (Beyotime, Shanghai, China). Cell samples were stained with JC-1 dye working solution, following the manufacturer's instructions. After that, the OLN-93 cells were rinsed with JC-1 staining buffer. The MMP changes were quantified by the relative fluorescence intensity ratio of the polymer-to-monomer (red/green).

### 2.11. Western Blot Analysis

Total protein was extracted from the OLN-93 cell homogenate. The denatured protein was electrophoresed on SDS polyacrylamide gels and then transferred onto polyvinylidene fluoride membranes (Millipore, Darmstadt, Germany). The membranes were blocked with StartingBlock blocking buffer (ThermoFisher, Waltham, USA) and then incubated in a universal antibody diluent (New Cell & Molecular Biotech, Suzhou, China) overnight at 4°C with primary antibodies against Bax (Proteintech, Wuhan, China), Bcl-2 (Proteintech, Wuhan, China), cleaved caspase-3 (CST, Danvers, USA), p-Akt (CST, Danvers, USA), Akt (CST, Danvers, USA), or *β*-actin (Genetex, Irvine, USA). Thereafter, the blots were incubated with HRP-conjugated secondary antibody (Proteintech, Wuhan, China) at room temperature for 1 hour. The protein bands were detected with ECL chemiluminescence (Millipore, Darmstadt, Germany) and visualized by the Fusion FX imaging system. The intensity of the bands was quantified by ImageJ software.

### 2.12. Statistical Analysis

Statistical analysis was conducted in GraphPad Prism 7.0 (GraphPad Software, San Diego, USA). All the data were shown as a mean ± standard deviation (SD) from at least three independent experiments. The difference between groups was analyzed by one-way analysis of variance. *P* < 0.05 was considered to be statistically significant (^∗^*P* < 0.05, ^∗∗^*P* < 0.01, ^∗∗∗^*P* < 0.001, ^∗∗∗∗^*P* < 0.0001).

## 3. Results

### 3.1. Potential Active Ingredients and Targets of the BSYS Capsule

**Figure 1:**
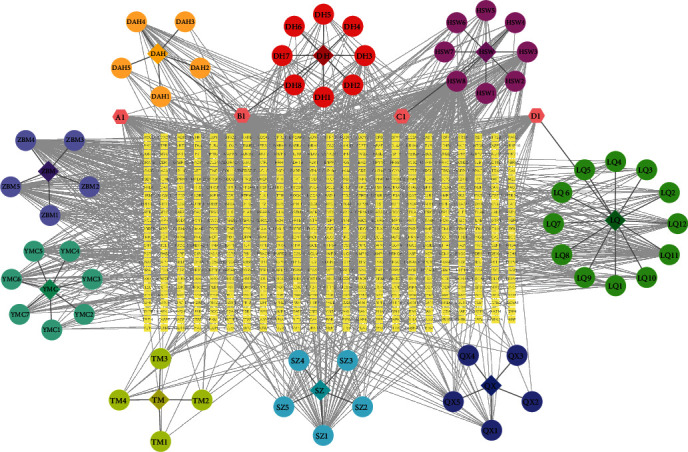
The ingredient-target network of BSYS Capsule. Diamond nodes represent the herbs, circle nodes represent the compounds, and square nodes represent the targets.

Our screening and literature mining analyses showed 65 potentially active compounds in the BSYS Capsule: 8 ingredients were identified from Dihuang, 8 from Heshouwu, 5 from Dahuang, 5 from Zhebeimu, 7 from Yimucao and 4 from Tianma, 5 from Shuizhi, 5 from Quanxie, and 12 from Lianqiao, while 4 of them were in multiple herbs. The 65 potentially active ingredients were searched in databases, and the data showed that the BSYS Capsule had 547 corresponding targets. The ingredient-target network of the BSYS Capsule consisted of 619 nodes and 1473 edges, indicating the potential synergistic roles of these herbs (Figure 1 and Table S1).

### 3.2. Construction of the “Chinese Medicine-Active Ingredient-Intersection Target” Network

**Figure 2:**
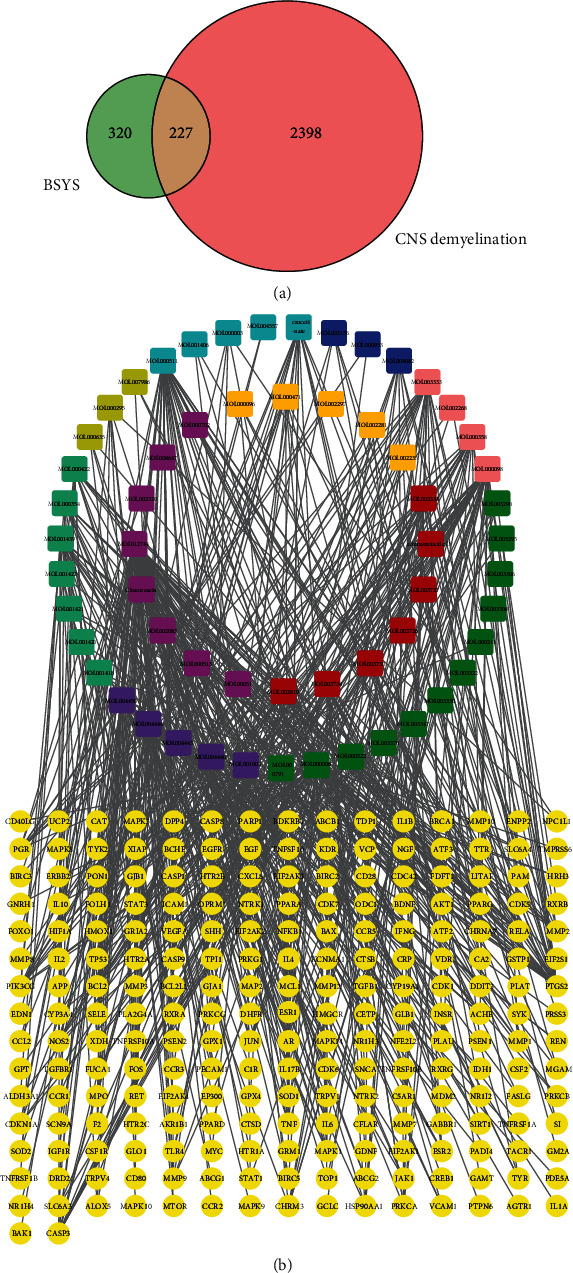
BSYS Capsule and CNS demyelination-related targets and overlapping targets. (a) Venn diagram of screened intersection targets of BSYS Capsule and CNSD-related targets. (b) Active ingredient-intersection target network. Circles of various colors and yellow squares represent the targets and ingredients, respectively. Different colors represent ingredients from different herbs, which is consistent with Figure 1.

A total of 2625 target genes related to CNS demyelination were obtained from GeneCards and DisGeNET database (Table S2). 227 overlapping genes were obtained by taking the intersection of the ingredient-target genes and CNSD-related genes (Table S3), which were thought to be the target genes of the BSYS Capsule in CNSD (Figure 2(a)). Six potential ingredients were excluded because their targets did not intersect with disease targets. This analysis showed that the 227 overlapping genes were regulated by 59 bioactive ingredients. Finally, we constructed an active ingredient-intersection target network containing 286 nodes and 621 edges using Cytoscape software (Figure 2(b)).

### 3.3. Construction and Analysis of Protein-Protein Interaction (PPI) Network

**Figure 3:**
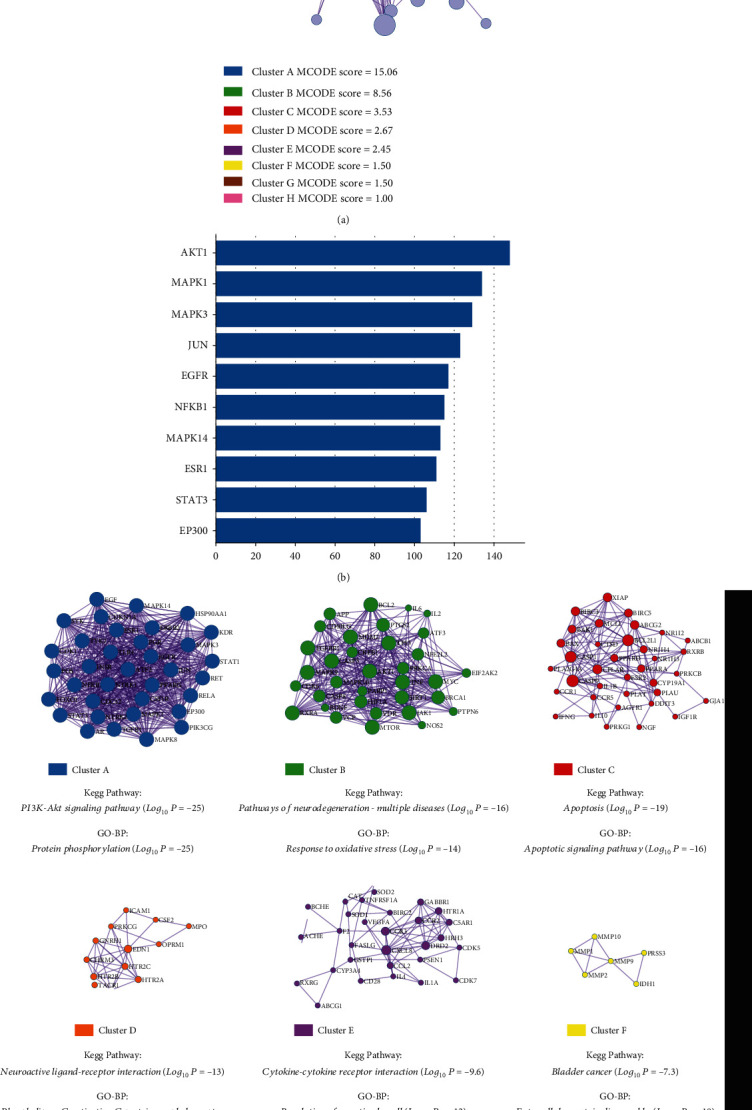
Protein-protein interaction (PPI) network of common targets between BSYS Capsule and CNSD. (a) PPI network and (b) the top 10 significant genes in the PPI network. (c) Eight clusters with the corresponding description from the PPI network.

To define the PPI network, we submitted the 227 intersecting targets to the Metascape database as shown in Figure 3(a). The results demonstrated that there were 226 nodes and 4859 edges in the PPI network (Table S4), and the degree values of all nodes were ranked in descending order (Table S5). The target node with high degree values was considered as core targets. As shown in Figure 3(b), the top 10 core targets included AKT1 (148), MAPK1 (134), MAPK3 (129), JUN (123), EGFR (117), NFKB1 (115), MAPK14 (113), ESR1 (111), STAT3 (106), and EP300 (103). Molecular Complex Detection (MCODE) analysis was employed to identify clusters with highly interconnected regions in the network. In this analysis, modules of the PPI network were divided into 8 clusters. On the other hand, the KEGG pathway and GO-BP analysis results showed that these clusters were mainly enriched in the PI3K-Akt signaling pathway, pathways of neurodegeneration, apoptosis, and neuroactive ligand-receptor interaction, while the GO terms were generally related to protein phosphorylation, response to oxidative stress, apoptotic signaling pathway, and extracellular matrix disassembly (Figure 3(c)).

### 3.4. GO and KEGG Pathway Enrichment Analysis of the Total Intersection Targets

**Figure 4:**
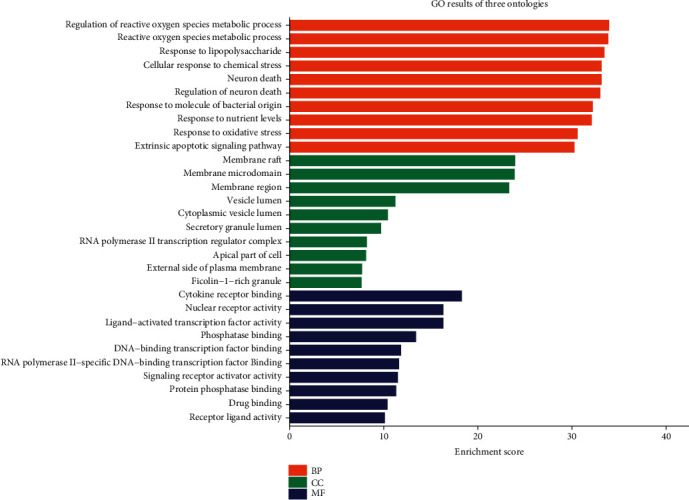
Gene Ontology terms of 227 intersection targets. The top 10 GO functional terms were selected (adj. *P* < 0.01). Abbreviations: BP: biological processes; CC: cellular component; MF: molecular function.

**Figure 5:**
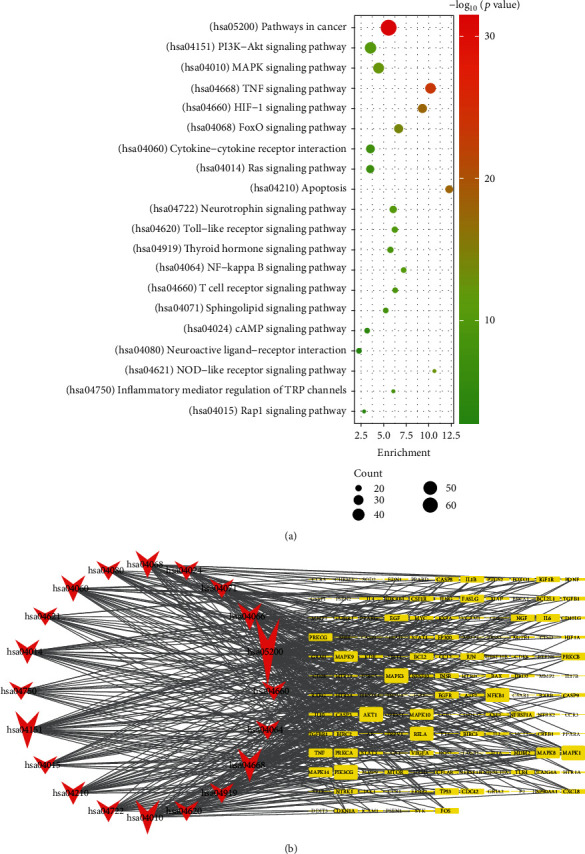
KEGG enrichment of potential targets of BSYS Capsule against CNSD. (a) The top 10 pathways with the highest count values were displayed (adj. *P* < 0.01). Sizes and colors of the spot represent the count value and *P* value, respectively. (b) The network of targets involved in the major pathways. The V-shapes and the squares represent pathways and target genes, respectively. The node degree is positively correlated with the shape area.

The 227 intersection genes were analyzed by GO functional enrichment and KEGG signaling pathway enrichment (adj. *P* < 0.01). The data showed that there were 2306 GO terms (Table S6). The top 10 terms of BP, CC, and MF were screened according to the enrichment scores. As shown in Figure 4, the main BP categories were reactive oxygen species metabolic process, response to lipopolysaccharide, neuron death, response to oxidative stress, and extrinsic apoptotic signaling pathway. MF mainly included cytokine receptor binding, nuclear receptor activity, ligand-activated transcription factor activity, DNA-binding transcription factor binding, and protein phosphatase binding. On the other hand, the top five CCs were membrane raft, membrane microdomain, membrane region, vesicle lumen, and cytoplasmic vesicle lumen. We then performed pathway enrichment using the KEGG database to screen related signaling pathways (adjusted *P* value <0.01) and showed that many pathways were associated with CNS demyelination (Table S7) (Figure 5(a)). The enriched pathways mainly included the PI3K-Akt signaling pathway, MAPK signaling pathway, TNF signaling pathway, HIF-1 signaling pathway, cytokine-cytokine receptor interaction, and apoptosis. The PI3K-Akt signaling pathway had the highest number of genes, which contained 37 targets. The 20 signaling pathways with high count values were selected as bubble charts. To establish a gene-pathway network, 20 significantly enriched pathways and 156 related genes were imported into Cytoscape (Figure 5(b)). The node degree was positively correlated with the shape area. The network analysis indicated that out of genes such as MAPK3, MAPK1, PIK3CG, NFKB1, and RELA, which had larger degrees, AKT1 had the highest degree (Table S8). Thus, these genes might be the key targets for BSYS Capsule in CNS demyelination. Besides, the data demonstrated that the enrichment analysis results of total intersection genes were generally consistent with 3.3. Taken together, we presumed that BSYS Capsule exerted antidemyelination effects by elevating AKT phosphorylation levels in injured oligodendrocytes to alleviate oxidative stress-induced apoptosis in CNSD.

### 3.5. BSYS Serum Alleviates H_2_O_2_-Induced Cytotoxicity in OLN-93 Cells

**Figure 6:**
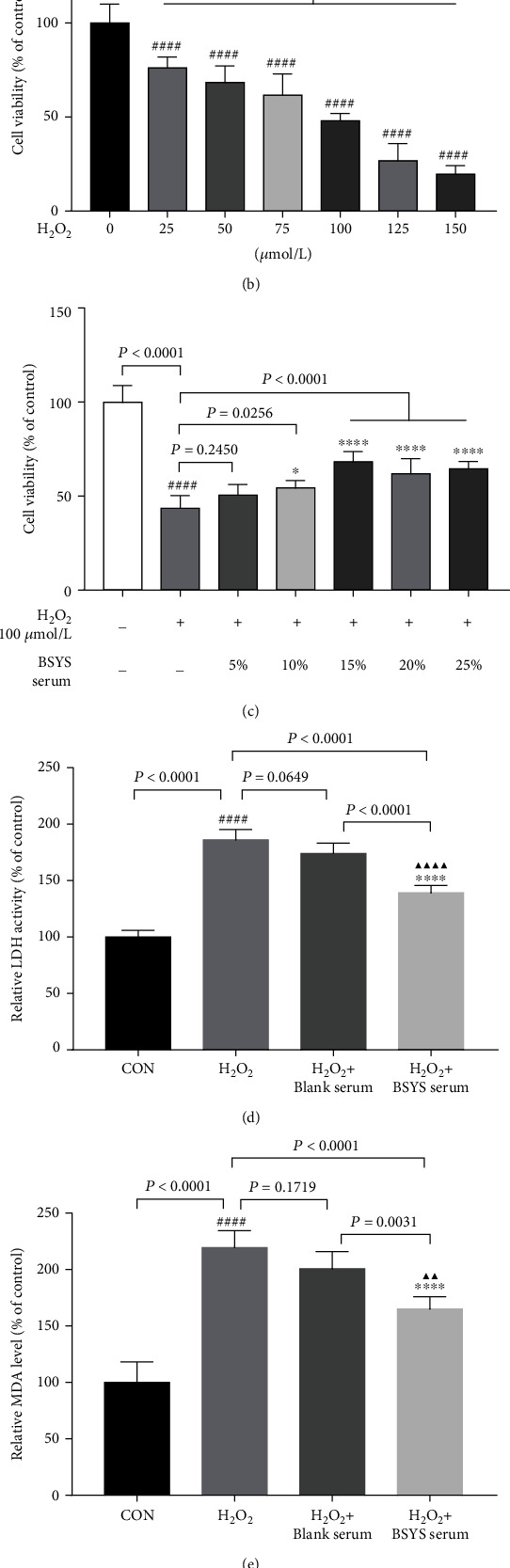
BSYS serum alleviates H_2_O_2_-induced cytotoxicity in OLN-93 cells. (a) The toxic effects of BSYS serum on OLN-93 cells accessed by CCK-8 assay. (b) Viability of OLN-93 cells exposed to various concentrations of H_2_O_2_. (c) To investigate the neuroprotective effect of BSYS serum, OLN-93 cells were incubated with H_2_O_2_ (100 *μ*mol/L) for 12 h and then treated with different concentrations of BSYS serum (5%-25%) for 24 h. (d) LDH content, (e) MDA level, and (f) SOD activity of OLN-93 cells were measured by the corresponding commercial kit. Data are presented as means ± SD, compared with the CON group, ^####^*P* < 0.0001; compared with the H_2_O_2_ group, ^∗∗∗∗^*P* < 0.0001; and compared with the H_2_O_2_ + blank serum group, ^▲▲^*P* < 0.01, ^▲▲▲^*P* < 0.001, ^▲▲▲▲^*P* < 0.0001.

To assess the safety of BSYS serum in the OLN-93 cells, we performed the CCK-8 assay to determine whether the concentration of BSYS serum affected the cell viability. The CCK-8 results showed that treatment with BSYS serum (5%–25%) for 24 h did not have a cytotoxic effect on the OLN-93 cells (Figure 6(a)). Next, we treated the OLN-93 cells with H_2_O_2_ at 25, 50, 75, 100, and 125 *μ*mol/L to induce oxidative damage. The viability of OLN-93 cells was significantly suppressed with H_2_O_2_ concentration from 25 to 125 *μ*mol/L (*P* < 0.0001). After administration with 100 *μ*mol/L H_2_O_2_ for 12 h, cell viability was suppressed to 48.03% compared to the control group (Figure 6(b)). In contrast, treated OLN-93 cells with BSYS serum in concentrations of 10% (*P* = 0.0256), 15% (*P* < 0.0001), 20% (*P* < 0.0001), and 25% (*P* < 0.0001) effectively ameliorated the suppressed cell viability by H_2_O_2_ (Figure 6(c)).

Moreover, H_2_O_2_-induced cytotoxicity was accompanied by LDH, MDA, and SOD dysregulation. As shown in Figures 6(d)–6(f), compared with the control group (CON), H_2_O_2_ significantly upregulated the levels of LDH and MDA while markedly suppressing the concentrations of SOD (*P* < 0.0001). After treatment with BSYS serum, the level of LDH and MDA was markedly decreased (*P* < 0.0001). Moreover, treatment with BSYS serum increased the SOD levels (*P* < 0.0001). However, administration of blank serum had no appreciable therapeutic effect on H_2_O_2_-injured OLN-93 cells (*P* = 0.0649, *P* = 0.1719, *P* = 0.5214, respectively).

### 3.6. BSYS Serum Inhibits H_2_O_2_-Induced ROS Accumulation and MMP Suppression in OLN-93 Cells

**Figure 7:**
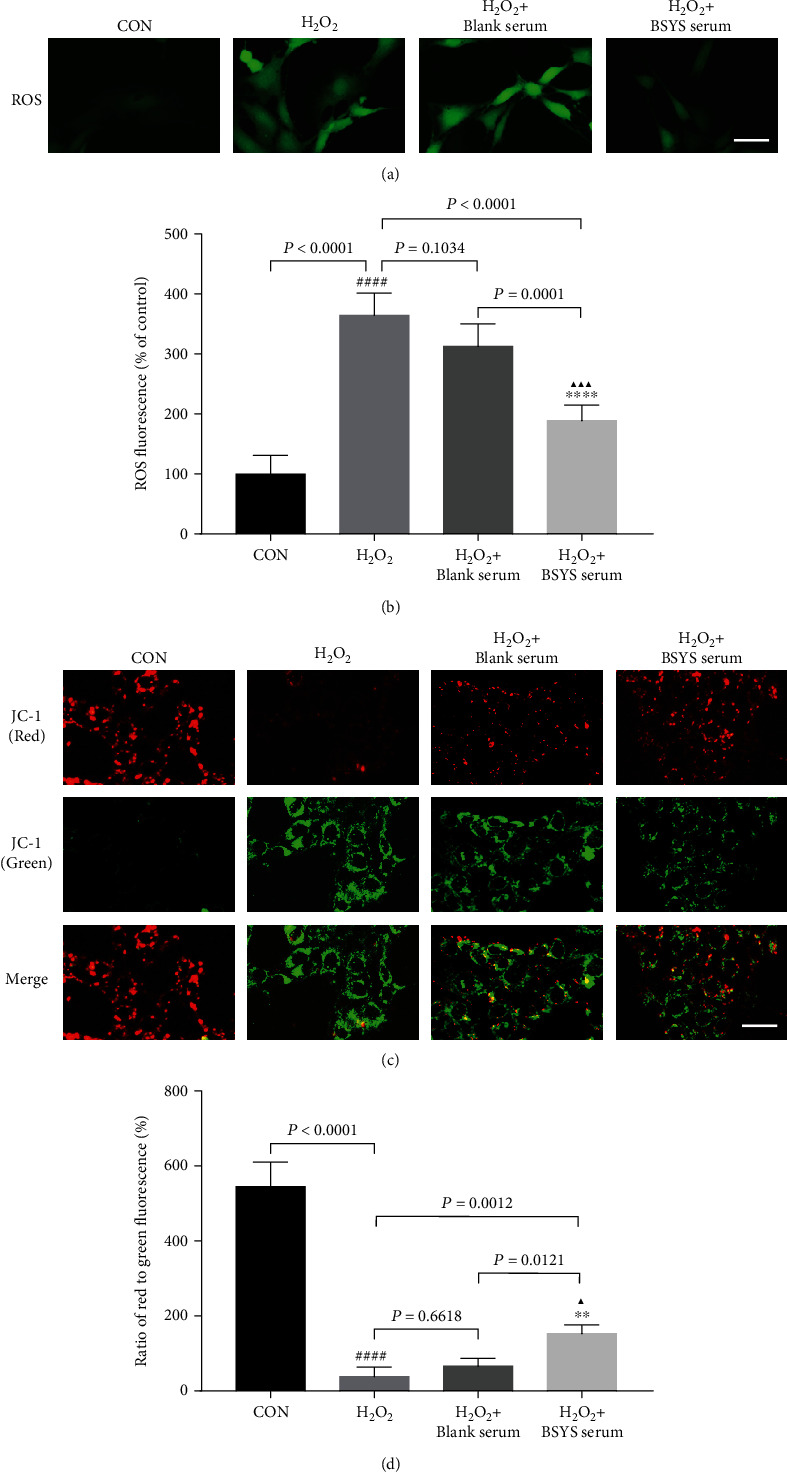
BSYS serum inhibits H2O2-induced ROS accumulation and MMP decrease in OLN-93 Cells. (a, b) The intracellular ROS level was assessed by DCFH-DA probe using fluorescence microscope (scale bars: 25 *μ*m) and microplate reader. (c, d) The MMP was detected via fluorescence microscope using JC-1 staining. Scale bar = 100 *μ*m. Data are presented as means ± SD, compared with the CON group, ^####^*P* <0.0001, compared with the H_2_O_2_ group, ^∗∗^*P* < 0.01, ^∗∗∗∗^*P* < 0.0001, and compared with the H_2_O_2_ + blank serum group, ^▲^*P* < 0.05, ^▲▲▲^*P* < 0.001.

The ROS accumulation was determined with DCF fluorescence. The DCF fluorescence was significantly brighter in the OLN-93 cells exposed to 100 *μ*mol/L H_2_O_2_ for 12 h compared to cells in the CON group (*P* < 0.0001), indicating an upregulation of ROS. However, compared with the H_2_O_2_ (*P* < 0.0001) and blank serum treatment groups (*P* = 0.0001), the intracellular ROS levels were remarkably inhibited after 24 h of incubation with BSYS serum (Figures 7(a) and 7(b)). Therefore, BSYS serum can reduce the overgeneration of ROS in oxidative stress.

ROS overproduction led to depolarization of MMP and mitochondria dysfunction. We analyzed whether BSYS serum could rescue the reduction of H_2_O_2_-induced MMP using JC-1 fluorescence dye. JC-1 aggregates (red fluorescence) were considered a marker of intact mitochondria, while the JC-1 monomers (green fluorescence) indicated MMP collapse. The OLN-93 cells in the CON group showed bright red fluorescence and dull green fluorescence. However, after stimulation with H_2_O_2_ for 12 h, the MMP was significantly suppressed (*P* < 0.0001), evidenced by the reduction in the ratio of red to green fluorescence. After treatment with BSYS serum, the compromised MMP was significantly ameliorated (*P* = 0.0012) (Figures 7(c) and 7(d)). Thus, BSYS serum effectively attenuated the suppression of MMP caused by H_2_O_2_-induced oxidative damage in the OLN-93 cells.

### 3.7. BSYS Serum Inhibited H_2_O_2_-Induced Apoptosis and Promoted Akt Phosphorylation in OLN-93 Cells

**Figure 8:**
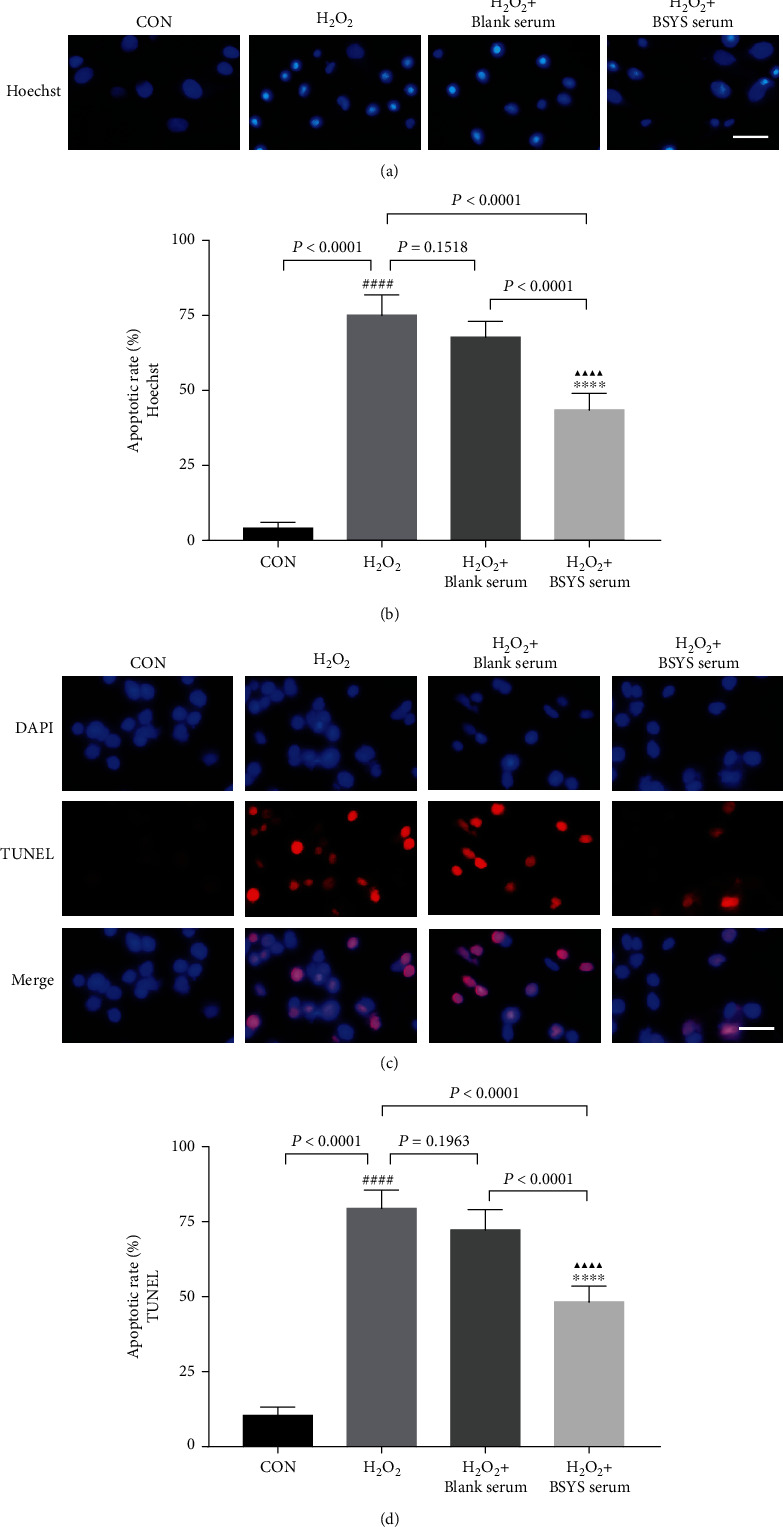
BSYS serum suppresses H_2_O_2_-induced apoptosis of OLN-93 cells. (a, b) Apoptotic nuclei of OLN-93 cells were stained by Hoechst 33342. Scale bar = 25 *μ*m. (c, d) The apoptotic cells (red fluorescence) were detected by TUNEL, and the nuclei (blue fluorescence) were stained by DAPI. Scale bar = 25 *μ*m. Data are presented as means ± SD, compared with the CON group, ^####^*P* < 0.0001; compared with the H_2_O_2_ group, ^∗∗∗∗^*P* < 0.0001, and compared with the H_2_O_2_ + blank serum group, ^▲▲▲▲^*P* < 0.0001.

It has been shown that ROS-mediated depolarization of the MMP is one of the major mechanisms of cellular apoptosis. To evaluate the protective effects of BSYS serum on H_2_O_2_-induced cell death, the OLN-93 cell apoptosis was examined by Hoechst 33342. The data showed that compared with the CON group, H_2_O_2_ stimulation exhibited typical characteristics of apoptosis (*P* < 0.0001), including cell shrinkage, nuclear pyknosis, and chromatin condensation. The H_2_O_2_-induced apoptosis of OLN-93 cells was reduced by administration with BSYS serum (*P* < 0.0001) (Figures 8(a) and 8(b)). Consistent with the Hoechst staining results, few TUNEL-positive OLN-93 cells were in the CON group. However, H_2_O_2_ treatment significantly increased the rate of TUNEL-positive cells (*P* < 0.0001), which was reduced by incubation with BSYS serum, relative to the blank serum group (*P* < 0.0001) (Figures 8(c) and 8(d)).

**Figure 9:**
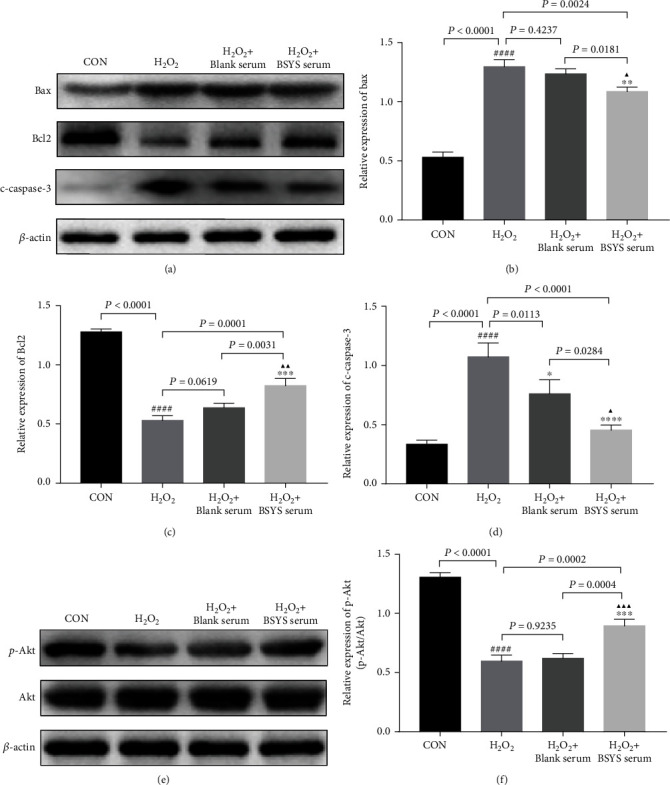
BSYS serum regulates the level of apoptosis-related proteins and the Akt pathway. (a)–(d) Representative blots and statistical graphs of relative protein expression of Bax, Bcl-2, and c-caspase3 in OLN-93 cells. (e, f) Representative western blot images and quantitative data of p-AKT and AKT in OLN-93 cells. Data are presented as means ± SD, compared with the CON group, ^####^*P* < 0.0001; compared with the H_2_O_2_ group, ^∗^*P* < 0.05, ^∗∗^*P* < 0.01, ^∗∗∗^*P* < 0.001; and compared with the H_2_O_2_ + blank serum group, ^▲^*P* < 0.05, ^▲▲^*P* < 0.01, ^▲▲▲^*P* < 0.001.

In addition, the expression of protein-related apoptosis was examined by Western blot analysis of Bcl-2, Bax, and cleaved-caspase-3 (c-caspase-3) proteins. The results showed that H_2_O_2_ notably increased the level of proapoptotic proteins such as Bax (*P* < 0.0001) and c-caspase-3 (*P* < 0.0001) compared with the CON group while significantly suppressing the expression of antiapoptotic protein (Bcl-2) (*P* < 0.0001). However, BSYS serum elevated the Bcl-2 protein levels (*P* = 0.0001) and suppressed the expression of Bax (*P* = 0.0024) and c-caspase-3 (*P* < 0.0001) (Figures 9(a)–9(d)). These outcomes demonstrated that BSYS serum markedly inhibited the H_2_O_2_-induced apoptosis in OLN-93 cells. To explore whether the activation of AKT was involved in the neuroprotective effect of BSYS in oxidative damage, the phosphorylation of AKT was analyzed by Western blot analysis. The results demonstrated that H_2_O_2_ inhibited AKT phosphorylation (p-AKT) (*P* < 0.0001). However, treatment with BSYS serum for 24 h attenuated the suppression of p-AKT induced by H_2_O_2_ (*P* = 0.0002) (Figures 9(e) and 9(f)).

### 3.8. Inhibition of AKT Phosphorylation Reverses the Neuroprotective Effects of BSYS Serum on the H_2_O_2_-Injured OLN-93 Cells

**Figure 10:**
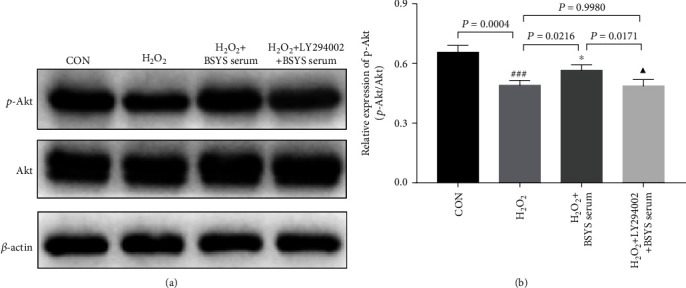
LY294002 blocks the promoting effect of BSYS serum on H_2_O_2_-induced p-Akt downregulation. (a, b) Representative western blot images and quantitative data of p-AKT and AKT in OLN-93 cells. Data are presented as means ± SD, compared with the CON group, ^###^*P* < 0.001, compared with the H_2_O_2_ group, ^∗^*P* < 0.05, and compared with the H_2_O_2_ + BSYS serum group, ^▲^*P* < 0.05.

To determine whether suppression of Akt activation weakened the neuroprotective effects of BSYS, Akt inhibitor (LY294002) was used to reduce the expression of p-AKT. As shown in Figures 10(a) and 10(b), preincubation with LY294002 attenuated the upregulation of p-Akt expression induced by BSYS serum in H_2_O_2_-injured OLN-93 cells (*P* = 0.0171).

**Figure 11:**
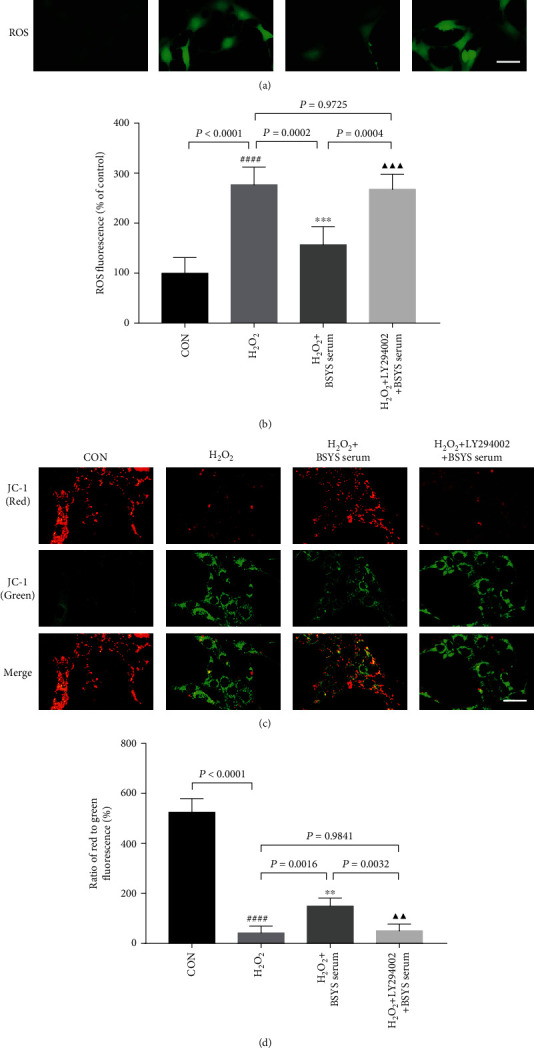
BSYS serum inhibits H_2_O_2_-induced ROS accumulation and MMP decrease via regulating Akt phosphorylation in OLN-93 Cells. (a, b) The intracellular ROS level was assessed by DCFH-DA probe using fluorescence microscope (scale bars: 25 *μ*m) and microplate reader. (c, d) The MMP was detected via fluorescence microscope using JC-1 staining. Scale bar = 100 *μ*m. Data are presented as means ± SD, compared with the CON group, ^####^*P* < 0.0001, compared with the H_2_O_2_ group, ^∗∗^*P* < 0.01, ^∗∗∗^*P* < 0.001, and compared with the H_2_O_2_ + BSYS serum group, ^▲▲^*P* < 0.01, ^▲▲▲^*P* < 0.001.

To investigate the effect of BSYS on H_2_O_2_-induced ROS overgeneration and MMP loss mediated by the Akt activation, OLN-93 cells were pretreated with LY294002 and then H_2_O_2_, followed by BSYS serum treatment. The results showed that compared with the BSYS serum treatment group, pretreatment with LY294002 notably increased ROS production (*P* = 0.0004) (Figures 11(a) and 11(b)) and decreased MMP (*P* = 0.0032) (Figures 11(c) and 11(d)).

**Figure 12:**
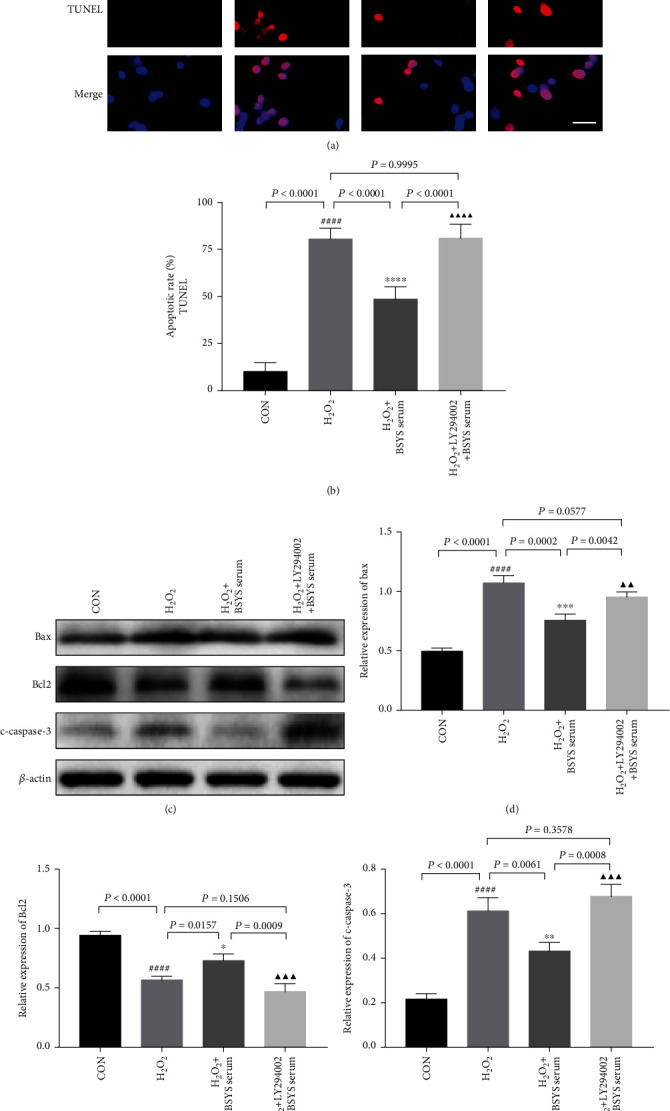
BSYS serum inhibits H_2_O_2_-induced apoptosis of OLN-93 cells via regulating Akt phosphorylation. (a, b) The apoptotic cells (red fluorescence) were detected by TUNEL, and the nuclei (blue fluorescence) were stained by DAPI. Scale bar = 25 *μ*m. (c)–(f) Representative blots and statistical graphs of relative protein expression of Bax, Bcl-2, and c-caspase3 in OLN-93 cells. Data are presented as means ± SD, compared with the CON group, ^####^*P* < 0.0001; compared with the H_2_O_2_ group, ^∗^*P* < 0.05, ^∗∗^*P* < 0.01, ^∗∗∗^*P* < 0.001, ^∗∗∗∗^*P* < 0.0001; and compared with the H_2_O_2_ + BSYS serum group, ^▲▲^*P* < 0.01, ^▲▲▲^*P* < 0.001, ^▲▲▲▲^*P* < 0.0001.

Furthermore, we evaluated whether inhibiting Akt activation abolished the antiapoptotic effects of BSYS. As shown in Figures 12(a) and 12(b), preincubation with LY294002 reversed the therapeutic effect of BSYS serum, which significantly elevated the ratio of TUNEL-positive cells (*P* < 0.0001), further demonstrating the involvement of Akt phosphorylation in BSYS-mediated antiapoptosis in H_2_O_2_-injured OLN-93 cells. Moreover, Western blot assays showed that LY294002 suppressed the Bcl-2 expression (*P* = 0.0009) and increased the expression of Bax (*P* = 0.0042) and c-caspase-3 (*P* = 0.0008) regulated by BSYS serum (Figures 12(c) and 12(d)).

## 4. Discussion

MS and NMO are autoimmune-mediated chronic inflammatory disorders of the CNS that mainly affects the brain, spinal cord, and optic nerve. These diseases are characterized by myelin damage, axonal degeneration, and neuronal loss [30, 31]. According to the theory of TCM, CNSD is described as “flaccidity syndrome” or “bone flaccidity.” In TCM, the primary cause of CNS demyelination is liver and kidney deficiency, as well as blood stasis and phlegm retention. Therefore, the treatment of CNSD follows the basic rules of tonifying the liver and kidney, activating blood, and resolving phlegm [32]. BSYS was designed in accordance with these therapeutic principles. Hence, it can be used for the clinical treatment of MS and NMO. Its efficacy and safety have been confirmed in a clinical trial, but the molecular mechanism of action has not been completely elucidated. In the present study, network pharmacology and *in vitro* experiments were conducted to systematically analyze the pharmacological mechanisms of BSYS Capsule in the treatment of CNS demyelination disorder.

In the present study, 59 bioactive ingredients were identified in BSYS, and 547 ingredient-related targets, 2625 CNSD-related targets, and 227 intersection targets shared between the BSYS Capsule and CNSD were obtained from the public databases. Among the identified ingredients, resveratrol, ursolic acid, and quercetin exhibited the most significant bioactivity with more than 35 target genes (Table S9) and thus were identified as key ingredients. In a previous study, resveratrol effectively ameliorated the clinical severity of MS in mice by maintaining the integrity of the blood-brain barrier [33] and reducing the number of activated encephalitogenic T cells [34]. It has also been found that resveratrol can ameliorate optic nerve inflammation and demyelination, attenuate retinal ganglion cell loss and axon damage [35], and prevent neurological dysfunction in optic neuritis mice [36]. Ursolic acid has immunomodulatory and remyelination effects in EAE/cuprizone-induced demyelination [37]. Additionally, ursolic acid was found to reduce the cognitive deficits and mitochondria-dependent apoptosis in the ethidium bromide-induced demyelination in rats [38]. Moreover, quercetin not only protected the cholinergic neurotransmission in demyelinated rats but also downregulated the MMP-9/TIMP-1 ratio by decreasing MMP-9 production in peripheral blood mononuclear cells isolated from MS patients [39]. It also improved myelin repair in the optic nerve in the lyolecithin-induced demyelination model, which significantly shortened the delay of visual signals [40].

Analysis of the PPI network revealed that AKT1, MAPK1, MAPK3, JUN, and EGFR were the top 5 targets based on degree value, which suggested that the BSYS Capsule may mitigate CNS demyelination by regulating these targets. Next, GO enrichment analysis was conducted on different clusters of the PPI network and total intersection targets. Results showed that the effects of BSYS Capsule on CNSD were associated with protein phosphorylation, reactive oxygen species metabolic process, regulation of neuron death, response to oxidative stress, and extrinsic apoptotic, among others. Moreover, the KEGG pathways analysis demonstrated that the intersection targets were mainly associated with the PI3K-Akt signaling pathway, MAPK signaling pathway, TNF signaling pathway, cytokine-cytokine receptor interaction, and apoptosis, among other pathways. Each signaling pathway contains numerous target genes, and the number of intersection targets reflects the importance of a pathway. Apart from cancer pathways, the PI3K-Akt signaling pathway had the highest number of genes, which contained 37 targets. Taken together, these results show that BSYS prevented apoptosis by elevating AKT phosphorylation levels in oxidative stress-injured oligodendrocytes.

Oxidative stress plays a vital role in inflammation-induced demyelination and neurodegeneration in MS. In the CNS, oxidative damage is driven by reactive microglia and astrocytes [41]. The formation of lipid peroxidation products and reactive oxygen species (ROS) in brain tissue, plasma, and cerebrospinal fluid [42] have been well documented during MS processes [43]. Excessive levels of ROS lead to the damage of lipids, proteins, and mitochondrial DNA. The subsequent mitochondrial dysfunction impairs energy production, exacerbating demyelination [44].

Oxidative stress can destroy various nerve cells, including neurons and glial cells. Of all types of glial cells, oligodendrocytes are the most vulnerable to ROS due to their weak antioxidant defense systems [45]. ROS-induced oxidative damage has been known to cause oligodendrocytes apoptosis, especially in demyelinating diseases [46]. Previous studies found that H_2_O_2_ reduced mitochondrial membrane potential, which increased intracellular accumulation of ROS, and oxidative-related apoptosis of oligodendroglial (OLN-93) cells [29]. Therefore, H_2_O_2_ can be used to induce oxidative stress-mediated oligodendrocyte loss of CNS demyelination *in vitro*.

In this study, we successfully established H_2_O_2_-induced oxidative damage in OLN-93 cells. The results of the CCK-8 assay showed that treatment with BSYS serum effectively alleviated the H_2_O_2_-induced decrease in viability of OLN-93 cells. Studies have shown that LDH, MDA, and SOD are sensitive indicators of intracellular oxidative stress. LDH is a stable enzyme present in all types of cells and is rapidly released following damage to the plasma membrane by ROS [47]. Therefore, it is the most widely used marker to investigate oxidative damage. Oxygen free radicals attack the cell membrane, leading to lipid peroxidation, which results in the formation of MDA. The MDA, in turn, impairs the mitochondrial respiratory chain complex [48]. As an important antioxidant enzyme, SOD mainly generates oxygen and hydrogen peroxide by catalyzing free radicals. In this way, it maintains the dynamic balance between oxidation and antioxidant systems [49]. In the present study, we found that BSYS serum effectively decreased MDA and LDH levels in OLN-93 cells exposed to H_2_O_2_, but it increased SOD activity.

To further explore the antioxidative stress effects of BSYS, we evaluated the level of ROS and apoptosis in oxidative injured OLN-93 cells. Excessive accumulation of ROS decreases cell survival by damaging DNA and lipids, as well as other cellular components [50]. Damage to these cellular components leads to the loss of oligodendrocytes by necrosis or apoptosis. Analysis of DCF fluorescence showed that treatment with BSYS serum alleviated the increase in ROS level induced by H_2_O_2_. The results of the TUNEL assay and western blotting assay targeting apoptosis-related proteins were consisted with the ROS results. Moreover, ROS has been found to decrease mitochondrial membrane potential (MMP) and trigger the activation of apoptosis-related factors, leading to cell death [51]. The present results indicated that the decrease in MMP following exposure to H_2_O_2_ was reversed by administration with BSYS serum. This suggests that BSYS inhibited the mitochondrial apoptosis pathway in oligodendrocytes by rescuing MMP.

PI3K/Akt signaling pathway regulates several cellular processes, including cell survival, proliferation, and differentiation. It also modifies the occurrence of apoptosis under oxidative stress conditions [52]. Studies have shown that activating the PI3K/Akt pathway can prevent oligodendrocyte precursor cells from oxidative damage, while inhibiting the Akt activity increases ROS sensitivity and the vulnerability of nerve cells to oxidative stress [53]. The serine/threonine kinase named protein kinase B (PKB), also known as Akt, is an important mediator of PI3K-related signals. Activation of PI3K leads to Akt phosphorylation (p-Akt), regarded as an indicator of PI3K activation [54]. The p-Akt can downregulate proapoptotic Bax protein's expression and upregulate the expression of Bcl-2, an antiapoptotic protein, thereby suppressing the caspase-mediated apoptosis cascade [55, 56]. In this study, H_2_O_2_ treatment reduced Akt phosphorylation and aggravated apoptosis of oligodendrocytes, whereas BSYS-containing serum protected OLN-93 cells and increased Akt phosphorylation. Meanwhile, treatment of serum-containing BSYS ameliorated the production of ROS and the loss of MMP in OLN93 cells. Notably, the therapeutic effect of serum-containing BSYS was significantly weakened by the administration of LY294002. Our finding shows that BSYS inhibits H_2_O_2_-induced OLN-93 cell death via regulating the Akt pathway.

## 5. Conclusions

In this study, network pharmacology and *in vitro* experiments were performed to investigate the antidemyelination mechanisms of BSYS Capsule in CNSD. The results showed that BSYS treatment alleviated oxidative stress-mediated oligodendrocyte apoptosis by promoting Akt phosphorylation in CNS demyelination disease. Thus, BSYS has promising prospects for the treatment of MS, NMO, and other CNS demyelinating diseases.

## Data Availability

The data used to support the findings of this study are included within the article.

## Supplementary Materials

Supplementary Materials Table S1: all the potential targets of BSYS Capsule. Table S2: known CNSD-related targets. Table S3: BSYS Capsule shared 227 intersection targets with known CNSD-related targets. Table S4: PPI information of 227 intersection targets in Metascape. Table S5: the degree values of all nodes in the PPI network. Table S6: results for GO pathway enrichment analysis. Table S7: results for KEGG pathway enrichment analysis. Table S8: information of gene-pathway network. Table S9: information of the “active ingredients-intersection targets” network.

## References

[1] Jurynczyk M., Craner M., Palace J. (2015). Overlapping CNS inflammatory diseases: differentiating features of NMO and MS. *Journal of Neurology, Neurosurgery, and Psychiatry*.

[2] Kawachi I., Lassmann H. (2017). Neurodegeneration in multiple sclerosis and neuromyelitis optica. *Journal of Neurology, Neurosurgery, and Psychiatry*.

[3] Wegner C. (2013). Recent insights into the pathology of multiple sclerosis and neuromyelitis optica. *Clinical Neurology and Neurosurgery*.

[4] Miao J., Aboagye D. E., Chulpayev B. (2018). Importance of regular and maintenance therapy adherence in neuromyelitis optica (NMO): lessons from a repeating relapse case. *American Journal of Case Reports*.

[5] Chitnis T., Glanz B. I., Gonzalez C. (2019). Quantifying neurologic disease using biosensor measurements in-clinic and in free-living settings in multiple sclerosis. *npj Digital Medicine*.

[6] Liu Y., Given K. S., Harlow D. E. (2017). Myelin-specific multiple sclerosis antibodies cause complement-dependent oligodendrocyte loss and demyelination. *Acta Neuropathologica Communications*.

[7] Gulati S., Chakrabarty B., Kumar A., Jain P., Patel H., Saini L. (2015). Acquired demyelinating disorders of central nervous system: a pediatric cohort. *Annals of Indian Academy of Neurology*.

[8] Wang Z. Y., Liu J., Zhu Z. (2021). Traditional Chinese medicine compounds regulate autophagy for treating neurodegenerative disease: a mechanism review. *Biomedicine & Pharmacotherapy*.

[9] Song L., Zhou Q. H., Wang H. L. (2017). Chinese herbal medicine adjunct therapy in patients with acute relapse of multiple sclerosis: a systematic review and meta-analysis. *Complementary Therapies in Medicine*.

[10] Liu J., Gao Y., Kan B. H., Zhou L. (2012). Systematic review and meta-analysis of randomized controlled trials of Chinese herbal medicine in treatment of multiple sclerosis. *Zhong Xi Yi Jie He Xue Bao*.

[11] Zhao X., Yang T., Cheng F. (2021). Abnormalcortical thickness in relapsing-remitting multiple sclerosis, correlations with cognition impairment, and effect of modified Bushenyisui decoction on cognitive function of multiple sclerosis. *Journal of Traditional Chinese Medicine*.

[12] Zhao T. Y., Yang T., Tong Y. P. (2020). Effects of Bushen Yisui capsule on IL-23/IL-17A axis and serum related cytokines in patient with neuromyelitis optica spectrum disorder during remission. *China Journal of Traditional Chinese Medicine and Pharmacy*.

[13] Fang L., Wang Y., Zheng Q. (2017). Effects of Bu Shen Yi sui capsule on NogoA/NgR and its signaling pathways RhoA/ROCK in mice with experimental autoimmune encephalomyelitis. *BMC Complementary and Alternative Medicine*.

[14] Zheng Q., Yang T., Fang L. (2015). Effects of Bu Shen Yi sui capsule on Th17/Treg cytokines in C57BL/6 mice with experimental autoimmune encephalomyelitis. *BMC Complementary and Alternative Medicine*.

[15] Zha Z., Gao Y. F., Ji J. (2021). Bu Shen Yi Sui Capsule alleviates neuroinflammation and demyelination by promoting microglia toward M2 polarization, which correlates with changes in miR-124 and miR-155 in experimental autoimmune encephalomyelitis. *Oxidative Medicine and Cellular Longevity*.

[16] Zhang J., Zhang Q., Chen X. (2018). Revealing synergistic mechanism of multiple components in Gandi capsule for diabetic nephropathy therapeutics by network pharmacology. *Evidence-based Complementary and Alternative Medicine*.

[17] Hopkins A. L. (2007). Network pharmacology. *Nature Biotechnology*.

[18] Hopkins A. L. (2008). Network pharmacology: the next paradigm in drug discovery. *Nature Chemical Biology*.

[19] Ru J., Li P., Wang J. (2014). TCMSP: a database of systems pharmacology for drug discovery from herbal medicines. *Journal of Cheminformatics*.

[20] Liu Z., Guo F., Wang Y. (2016). BATMAN-TCM: a bioinformatics analysis tool for molecular mechANism of traditional Chinese medicine. *Scientific Reports*.

[21] Kim S., Chen J., Cheng T. (2021). PubChem in 2021: new data content and improved web interfaces. *Nucleic Acids Research*.

[22] Daina A., Michielin O., Zoete V. (2019). SwissTargetPrediction: updated data and new features for efficient prediction of protein targets of small molecules. *Nucleic Acids Research*.

[23] Stelzer G., Rosen N., Plaschkes I. (2016). The GeneCards suite: from gene data mining to disease genome sequence analyses. *Current Protocols in Bioinformatics*.

[24] Piñero J., Bravo À., Queralt-Rosinach N. (2017). DisGeNET: a comprehensive platform integrating information on human disease-associated genes and variants. *Nucleic Acids Research*.

[25] Shannon P., Markiel A., Ozier O. (2003). Cytoscape: a software environment for integrated models of biomolecular interaction networks. *Genome Research*.

[26] Zhou Y., Zhou B., Pache L. (2019). Metascape provides a biologist-oriented resource for the analysis of systems-level datasets. *Nature Communications*.

[27] Dennis G. J., Sherman B. T., Hosack D. A. (2003). DAVID: Database for annotation, visualization, and integrated discovery. *Genome Biology*.

[28] Zhao P. Y., Ji J., Liu X. H. (2020). Bu-Shen-Yi-Sui Capsule, an herbal medicine formula, promotes remyelination by modulating the molecular signals via exosomes in mice with experimental autoimmune encephalomyelitis. *Oxidative Medicine and Cellular Longevity*.

[29] Li F., Song X., Xu J. (2021). Morroniside protects OLN-93 cells against H2O2-induced injury through the PI3K/Akt pathway-mediated antioxidative stress and antiapoptotic activities. *Cell Cycle*.

[30] Liu Y., Duan Y., Huang J. (2018). Different patterns of longitudinal brain and spinal cord changes and their associations with disability progression in NMO and MS. *European Radiology*.

[31] Vaknin-Dembinsky A., Karussis D., Avichzer J., Abramsky O. (2014). NMO spectrum of disorders: a paradigm for astrocyte-targeting autoimmunity and its implications for MS and other CNS inflammatory diseases. *Journal of Autoimmunity*.

[32] Zhao P. Y., Wang Y. Q., Liu X. H. (2018). Bu Shen Yi sui capsule promotes remyelination correlating with Sema3A/NRP-1, LIF/LIFR and Nkx6.2 in mice with experimental autoimmune encephalomyelitis. *Journal of Ethnopharmacology*.

[33] Wang D., Li S. P., Fu J. S., Zhang S., Bai L., Guo L. (2016). Resveratrol defends blood-brain barrier integrity in experimental autoimmune encephalomyelitis mice. *Journal of Neurophysiology*.

[34] Gandy K., Zhang J., Nagarkatti P., Nagarkatti M. (2019). Resveratrol (3, 5, 4'-trihydroxy-trans-stilbene) attenuates a mouse model of multiple sclerosis by altering the miR-124/sphingosine kinase 1 axis in encephalitogenic T cells in the brain. *Journal of Neuroimmune Pharmacology*.

[35] Fonseca-Kelly Z., Nassrallah M., Uribe J. (2012). Resveratrol neuroprotection in a chronic mouse model of multiple sclerosis. *Frontiers in Neurology*.

[36] Shindler K. S., Ventura E., Rex T. S., Elliott P., Rostami A. (2007). SIRT1 activation confers neuroprotection in experimental optic neuritis. *Investigative Ophthalmology & Visual Science*.

[37] Zhang Y., Li X., Ciric B. (2020). A dual effect of ursolic acid to the treatment of multiple sclerosis through both immunomodulation and direct remyelination. *Proceedings of the National Academy of Sciences of the United States of America*.

[38] Garabadu D., Singh D. (2020). Ocimum basilicum attenuates ethidium bromide-induced cognitive deficits and pre-frontal cortical neuroinflammation, astrogliosis and mitochondrial dysfunction in rats. *Metabolic Brain Disease*.

[39] Sternberg Z., Chadha K., Lieberman A. (2008). Quercetin and interferon-*β* modulate immune response(s) in peripheral blood mononuclear cells isolated from multiple sclerosis patients. *Journal of Neuroimmunology*.

[40] Naeimi R., Baradaran S., Ashrafpour M., Moghadamnia A. A., Ghasemi-Kasman M. (2018). Querectin improves myelin repair of optic chiasm in lyolecithin-induced focal demyelination model. *Biomedicine & Pharmacotherapy*.

[41] Kim Y. S., Choi J., Yoon B. E. (2020). Neuron-glia interactions in neurodevelopmental disorders. *Cells*.

[42] Ferretti G., Bacchetti T. (2011). Peroxidation of lipoproteins in multiple sclerosis. *Journal of the Neurological Sciences*.

[43] Wang P., Xie K., Wang C., Bi J. (2014). Oxidative stress induced by lipid peroxidation is related with inflammation of demyelination and neurodegeneration in multiple sclerosis. *European Neurology*.

[44] Ohl K., Tenbrock K., Kipp M. (2016). Oxidative stress in multiple sclerosis: central and peripheral mode of action. *Experimental Neurology*.

[45] Giacci M. K., Bartlett C. A., Smith N. M. (2018). Oligodendroglia are particularly vulnerable to oxidative damage after neurotraumaIn vivo. *Journal of Neuroscience*.

[46] Takase H., Lok J., Arai K. (2018). A radical scavenger edaravone and oligodendrocyte protection/regeneration. *Neural Regeneration Research*.

[47] Smith S. M., Wunder M. B., Norris D. A., Shellman Y. G. (2011). A simple protocol for using a LDH-based cytotoxicity assay to assess the effects of death and growth inhibition at the same time. *PLoS One*.

[48] Tsikas D. (2017). Assessment of lipid peroxidation by measuring malondialdehyde (MDA) and relatives in biological samples: analytical and biological challenges. *Analytical Biochemistry*.

[49] Wang Y., Branicky R., Noe A., Hekimi S. (2018). Superoxide dismutases: dual roles in controlling ROS damage and regulating ROS signaling. *Journal of Cell Biology*.

[50] Lushchak V. I. (2014). Free radicals, reactive oxygen species, oxidative stress and its classification. *Chemico-Biological Interactions*.

[51] Kalpage H. A., Bazylianska V., Recanati M. A. (2019). Tissue-specific regulation of cytochrome c by post-translational modifications: respiration, the mitochondrial membrane potential, ROS, and apoptosis. *FASEB Journal*.

[52] Checa J., Aran J. M. (2020). Reactive oxygen species: drivers of physiological and pathological processes. *Journal of Inflammation Research*.

[53] Kim J. Y., Kim C. H., Lee E. Y., Seo J. H. (2020). Alpha B-crystallin overexpression protects oligodendrocyte precursor cells against oxidative stress-induced apoptosis through the Akt pathway. *Journal of Molecular Neuroscience*.

[54] Cantley L. C. (2002). The phosphoinositide 3-kinase pathway. *Science*.

[55] Roos W. P., Kaina B. (2006). DNA damage-induced cell death by apoptosis. *Trends in Molecular Medicine*.

[56] Redza-Dutordoir M., Averill-Bates D. A. (2016). Activation of apoptosis signalling pathways by reactive oxygen species. *Biochimica et Biophysica Acta*.

