# Supplementary material for: Study on the Anti-demyelination Mechanism of Bu-Shen-Yi-Sui Capsule in the Central Nervous System Based on Network Pharmacology and Experimental Verification
Source: Mediators Inflamm. 2022 Jul 12;2022:9241261. doi: 10.1155/2022/9241261 (PMC9296285; doi:10.1155/2022/9241261)
Supplement: Supplementary Materials — Table S1: all the potential targets of BSYS Capsule. Table S2: known CNSD-related targets. Table S3: BSYS Capsule shared 227 intersection targets with known CNSD-related targets. Table S4: PPI information of 227 intersection targets in Metascape. Table S5: the degree values of all nodes in the PPI network. Table S6: results for GO pathway enrichment analysis. Table S7: results for KEGG pathway enrichment analysis. Table S8: information of gene-pathway network. Table S9: information of the “active ingredients-intersection targets” network. [file 9241261.f1.zip › Table S1.docx]

Drug Code Molecular ID Molecular Name Gene Symbol

Dihuang DH1 MOL002819 catalpol GPT

Dihuang DH1 MOL002819 catalpol HMOX1

Dihuang DH1 MOL002819 catalpol KEAP1

Dihuang DH1 MOL002819 catalpol NFE2L2

Dihuang DH1 MOL002819 catalpol NQO1

Dihuang DH1 MOL002819 catalpol IFNG

Dihuang DH1 MOL002819 catalpol NOS2

Dihuang DH1 MOL002819 catalpol PTGS2

Dihuang DH1 MOL002819 catalpol TLR4

Dihuang DH1 MOL002819 catalpol ACHE

Dihuang DH1 MOL002819 catalpol CHAT

Dihuang DH1 MOL002819 catalpol IL1B

Dihuang DH1 MOL002819 catalpol TNF

Dihuang DH1 MOL002819 catalpol CHRM1

Dihuang DH1 MOL002819 catalpol DDP4

Dihuang DH1 MOL002819 catalpol BCL2

Dihuang DH1 MOL002819 catalpol CASP3

Dihuang DH1 MOL002819 catalpol SOD1

Dihuang DH2 MOL003734 echinacoside ATF3

Dihuang DH2 MOL003734 echinacoside CASP3

Dihuang DH2 MOL003734 echinacoside DDIT3

Dihuang DH2 MOL003734 echinacoside GDNF

Dihuang DH2 MOL003734 echinacoside SNCA

Dihuang DH2 MOL003734 echinacoside AKT1

Dihuang DH2 MOL003734 echinacoside BDNF

Dihuang DH2 MOL003734 echinacoside MAPK1

Dihuang DH2 MOL003734 echinacoside MAPK3

Dihuang DH2 MOL003734 echinacoside NGF

Dihuang DH2 MOL003734 echinacoside NTRK1

Dihuang DH2 MOL003734 echinacoside NTRK2

Dihuang DH2 MOL003734 echinacoside BACH1

Dihuang DH2 MOL003734 echinacoside HMOX1

Dihuang DH2 MOL003734 echinacoside NFE2L2

Dihuang DH2 MOL003734 echinacoside MMP2

Dihuang DH2 MOL003734 echinacoside MMP12

Dihuang DH2 MOL003734 echinacoside PRKCA

Dihuang DH2 MOL003734 echinacoside HSP90AA1

Dihuang DH2 MOL003734 echinacoside CA2

Dihuang DH3 MOL003333 acteoside PRKCA

Dihuang DH3 MOL003333 acteoside MMP2

Dihuang DH3 MOL003333 acteoside MMP12

Dihuang DH3 MOL003333 acteoside MMP13

Dihuang DH3 MOL003333 acteoside AKR1B1

Dihuang DH3 MOL003333 acteoside HSP90AA1

Dihuang DH3 MOL003333 acteoside APP

Dihuang DH3 MOL003333 acteoside IMPDH1

Dihuang DH3 MOL003333 acteoside IMPDH2

Dihuang DH3 MOL003333 acteoside MMP7

Dihuang DH3 MOL003333 acteoside MMP8

Dihuang DH3 MOL003333 acteoside LGALS1

Dihuang DH3 MOL003333 acteoside AKR1B10

Dihuang DH3 MOL003333 acteoside MMP1

Dihuang DH3 MOL003333 acteoside SLC28A3

Dihuang DH3 MOL003333 acteoside AIFM1

Dihuang DH3 MOL003333 acteoside BAX

Dihuang DH3 MOL003333 acteoside BCL2

Dihuang DH3 MOL003333 acteoside CAT

Dihuang DH3 MOL003333 acteoside CYCS

Dihuang DH3 MOL003333 acteoside TRP53

Dihuang DH3 MOL003333 acteoside GCLC

Dihuang DH3 MOL003333 acteoside GCLM

Dihuang DH3 MOL003333 acteoside HMOX1

Dihuang DH3 MOL003333 acteoside NFE2L2

Dihuang DH4 MOL000519 coniferin ADORA2A

Dihuang DH4 MOL000519 coniferin ADORA1

Dihuang DH4 MOL000519 coniferin ADORA3

Dihuang DH4 MOL000519 coniferin SLC5A2

Dihuang DH5 MOL003730 Rehmannioside A SI

Dihuang DH5 MOL003730 Rehmannioside A CA2

Dihuang DH5 MOL003730 Rehmannioside A ADORA2A

Dihuang DH5 MOL003730 Rehmannioside A MGAM

Dihuang DH5 MOL003730 Rehmannioside A TYR

Dihuang DH5 MOL003730 Rehmannioside A FOLH1

Dihuang DH5 MOL003730 Rehmannioside A CA1

Dihuang DH5 MOL003730 Rehmannioside A CA12

Dihuang DH5 MOL003730 Rehmannioside A CA9

Dihuang DH5 MOL003730 Rehmannioside A FUCA1

Dihuang DH5 MOL003730 Rehmannioside A HSP90AA1

Dihuang DH5 MOL003730 Rehmannioside A TREH

Dihuang DH6 MOL003726 rehmannioside B HSP90AA1

Dihuang DH6 MOL003726 rehmannioside B CA2

Dihuang DH6 MOL003726 rehmannioside B CA1

Dihuang DH6 MOL003726 rehmannioside B CA12

Dihuang DH6 MOL003726 rehmannioside B CA9

Dihuang DH7 MOL003727 rehmannioside C TYR

Dihuang DH7 MOL003727 rehmannioside C AMY2A

Dihuang DH7 MOL003727 rehmannioside C CA2

Dihuang DH7 MOL003727 rehmannioside C CA1

Dihuang DH7 MOL003727 rehmannioside C CA12

Dihuang DH7 MOL003727 rehmannioside C CA9

Dihuang DH7 MOL003727 rehmannioside C CA14

Dihuang DH7 MOL003727 rehmannioside C SI

Dihuang DH7 MOL003727 rehmannioside C HSP90AA1

Dihuang DH7 MOL003727 rehmannioside C FUCA1

Dihuang DH7 MOL003727 rehmannioside C ADORA1

Dihuang DH7 MOL003727 rehmannioside C MGAM

Dihuang DH7 MOL003727 rehmannioside C ADORA2A

Dihuang DH7 MOL003727 rehmannioside C FOLH1

Dihuang DH7 MOL003727 rehmannioside C AMY1A

Dihuang DH7 MOL003727 rehmannioside C GAA

Dihuang DH7 MOL003727 rehmannioside C EPHX2

Dihuang DH7 MOL003727 rehmannioside C SLC6A2

Dihuang DH7 MOL003727 rehmannioside C FPGS

Dihuang DH7 MOL003727 rehmannioside C TREH

Dihuang DH8 rehmannioside D rehmannioside D ADORA2A

Dihuang DH8 rehmannioside D rehmannioside D CDK1

Dihuang DH8 rehmannioside D rehmannioside D CASP3

Dihuang A1 MOL000449 Stigmasterol PGR

Dihuang A1 MOL000449 Stigmasterol NR3C2

Dihuang A1 MOL000449 Stigmasterol NCOA2

Dihuang A1 MOL000449 Stigmasterol ADH1C

Dihuang A1 MOL000449 Stigmasterol IGHG1

Dihuang A1 MOL000449 Stigmasterol RXRA

Dihuang A1 MOL000449 Stigmasterol NCOA1

Dihuang A1 MOL000449 Stigmasterol PTGS1

Dihuang A1 MOL000449 Stigmasterol PTGS2

Dihuang A1 MOL000449 Stigmasterol ADRA2A

Dihuang A1 MOL000449 Stigmasterol SLC6A2

Dihuang A1 MOL000449 Stigmasterol SLC6A3

Dihuang A1 MOL000449 Stigmasterol ADRB2

Dihuang A1 MOL000449 Stigmasterol PLAU

Dihuang A1 MOL000449 Stigmasterol LTA4H

Dihuang A1 MOL000449 Stigmasterol MAOB

Dihuang A1 MOL000449 Stigmasterol MAOA

Dihuang A1 MOL000449 Stigmasterol PRKACA

Dihuang A1 MOL000449 Stigmasterol CTRB1

Dihuang A1 MOL000449 Stigmasterol CHRM3

Dihuang A1 MOL000449 Stigmasterol CHRM1

Dihuang A1 MOL000449 Stigmasterol ADRB1

Dihuang A1 MOL000449 Stigmasterol SCN5A

Dihuang A1 MOL000449 Stigmasterol HTR2A

Dihuang A1 MOL000449 Stigmasterol ADRA1A

Dihuang A1 MOL000449 Stigmasterol GABRA3

Dihuang A1 MOL000449 Stigmasterol CHRM2

Dihuang A1 MOL000449 Stigmasterol ADRA1B

Dihuang A1 MOL000449 Stigmasterol GABRA1

Dihuang A1 MOL000449 Stigmasterol CHRNA7

Dihuang B1 MOL000358 beta-sitosterol RELA

Dihuang B1 MOL000358 beta-sitosterol NR3C2

Dihuang B1 MOL000358 beta-sitosterol PGR

Dihuang B1 MOL000358 beta-sitosterol NCOA2

Dihuang B1 MOL000358 beta-sitosterol PTGS1

Dihuang B1 MOL000358 beta-sitosterol PTGS2

Dihuang B1 MOL000358 beta-sitosterol HSP90

Dihuang B1 MOL000358 beta-sitosterol PI3K

Dihuang B1 MOL000358 beta-sitosterol KCNH2

Dihuang B1 MOL000358 beta-sitosterol PRKACA

Dihuang B1 MOL000358 beta-sitosterol CHRM3

Dihuang B1 MOL000358 beta-sitosterol CHRM1

Dihuang B1 MOL000358 beta-sitosterol SCN5A

Dihuang B1 MOL000358 beta-sitosterol GABRA2

Dihuang B1 MOL000358 beta-sitosterol CHRM4

Dihuang B1 MOL000358 beta-sitosterol PDE3A

Dihuang B1 MOL000358 beta-sitosterol HTR2A

Dihuang B1 MOL000358 beta-sitosterol GABRA5

Dihuang B1 MOL000358 beta-sitosterol ADRA1A

Dihuang B1 MOL000358 beta-sitosterol GABRA3

Dihuang B1 MOL000358 beta-sitosterol CHRM2

Dihuang B1 MOL000358 beta-sitosterol ADRA1B

Dihuang B1 MOL000358 beta-sitosterol ADRB2

Dihuang B1 MOL000358 beta-sitosterol CHRNA2

Dihuang B1 MOL000358 beta-sitosterol SLC6A4

Dihuang B1 MOL000358 beta-sitosterol OPRM1

Dihuang B1 MOL000358 beta-sitosterol GABRA1

Dihuang B1 MOL000358 beta-sitosterol CHRNA7

Dihuang B1 MOL000358 beta-sitosterol BCL2

Dihuang B1 MOL000358 beta-sitosterol BAX

Dihuang B1 MOL000358 beta-sitosterol CASP9

Dihuang B1 MOL000358 beta-sitosterol JUN

Dihuang B1 MOL000358 beta-sitosterol CASP3

Dihuang B1 MOL000358 beta-sitosterol CASP8

Dihuang B1 MOL000358 beta-sitosterol PRKCA

Dihuang B1 MOL000358 beta-sitosterol TGFB1

Dihuang B1 MOL000358 beta-sitosterol PON1

Dihuang B1 MOL000358 beta-sitosterol MAP2

Dahuang DAH1 MOL002235 EUPATIN NOS2

Dahuang DAH1 MOL002235 EUPATIN AR

Dahuang DAH1 MOL002235 EUPATIN PTGS2

Dahuang DAH1 MOL002235 EUPATIN F7

Dahuang DAH1 MOL002235 EUPATIN ESR2

Dahuang DAH1 MOL002235 EUPATIN DDP4

Dahuang DAH1 MOL002235 EUPATIN HSP90

Dahuang DAH1 MOL002235 EUPATIN PRSS1

Dahuang DAH1 MOL002235 EUPATIN NCOA2

Dahuang DAH1 MOL002235 EUPATIN SCN5A

Dahuang DAH1 MOL002235 EUPATIN KDR

Dahuang DAH1 MOL002235 EUPATIN PPARD

Dahuang C1 MOL002268 rhein PTGS1

Dahuang C1 MOL002268 rhein PTGS2

Dahuang C1 MOL002268 rhein HSP90

Dahuang C1 MOL002268 rhein PIK3CG

Dahuang C1 MOL002268 rhein NCOA2

Dahuang C1 MOL002268 rhein JUN

Dahuang DAH2 MOL002281 Toralactone NOS2

Dahuang DAH2 MOL002281 Toralactone PTGS1

Dahuang DAH2 MOL002281 Toralactone ESR1

Dahuang DAH2 MOL002281 Toralactone PTGS2

Dahuang DAH2 MOL002281 Toralactone ESR2

Dahuang DAH2 MOL002281 Toralactone HSP90

Dahuang DAH2 MOL002281 Toralactone PIK3CG

Dahuang DAH2 MOL002281 Toralactone CHEK1

Dahuang DAH2 MOL002281 Toralactone PRKACA

Dahuang DAH3 MOL002297 Daucosterol_qt PGR

Dahuang DAH3 MOL002297 Daucosterol_qt NCOA2

Dahuang B1 MOL000358 beta-sitosterol PGR

Dahuang B1 MOL000358 beta-sitosterol NCOA2

Dahuang B1 MOL000358 beta-sitosterol PTGS1

Dahuang B1 MOL000358 beta-sitosterol PTGS2

Dahuang B1 MOL000358 beta-sitosterol HSP90

Dahuang B1 MOL000358 beta-sitosterol PIK3CG

Dahuang B1 MOL000358 beta-sitosterol KCNH2

Dahuang B1 MOL000358 beta-sitosterol PRKACA

Dahuang B1 MOL000358 beta-sitosterol CHRM3

Dahuang B1 MOL000358 beta-sitosterol CHRM1

Dahuang B1 MOL000358 beta-sitosterol SCN5A

Dahuang B1 MOL000358 beta-sitosterol GABRA2

Dahuang B1 MOL000358 beta-sitosterol CHRM4

Dahuang B1 MOL000358 beta-sitosterol PDE3A

Dahuang B1 MOL000358 beta-sitosterol HTR2A

Dahuang B1 MOL000358 beta-sitosterol GABRA5

Dahuang B1 MOL000358 beta-sitosterol ADRA1A

Dahuang B1 MOL000358 beta-sitosterol GABRA3

Dahuang B1 MOL000358 beta-sitosterol CHRM2

Dahuang B1 MOL000358 beta-sitosterol ADRA1B

Dahuang B1 MOL000358 beta-sitosterol ADRB2

Dahuang B1 MOL000358 beta-sitosterol CHRNA2

Dahuang B1 MOL000358 beta-sitosterol SLC6A4

Dahuang B1 MOL000358 beta-sitosterol OPRM1

Dahuang B1 MOL000358 beta-sitosterol GABRA1

Dahuang B1 MOL000358 beta-sitosterol CHRNA7

Dahuang B1 MOL000358 beta-sitosterol BCL2

Dahuang B1 MOL000358 beta-sitosterol BAX

Dahuang B1 MOL000358 beta-sitosterol CASP9

Dahuang B1 MOL000358 beta-sitosterol JUN

Dahuang B1 MOL000358 beta-sitosterol CASP3

Dahuang B1 MOL000358 beta-sitosterol CASP8

Dahuang B1 MOL000358 beta-sitosterol PRKCA

Dahuang B1 MOL000358 beta-sitosterol TGFB1

Dahuang B1 MOL000358 beta-sitosterol PON1

Dahuang B1 MOL000358 beta-sitosterol MAP2

Dahuang DAH4 MOL000471 aloe-emodin PTGS1

Dahuang DAH4 MOL000471 aloe-emodin PTGS2

Dahuang DAH4 MOL000471 aloe-emodin HSP90

Dahuang DAH4 MOL000471 aloe-emodin PIK3CG

Dahuang DAH4 MOL000471 aloe-emodin PRKACA

Dahuang DAH4 MOL000471 aloe-emodin NCOA2

Dahuang DAH4 MOL000471 aloe-emodin PKIA

Dahuang DAH4 MOL000471 aloe-emodin IGHG1

Dahuang DAH4 MOL000471 aloe-emodin CDKN1A

Dahuang DAH4 MOL000471 aloe-emodin EIF6

Dahuang DAH4 MOL000471 aloe-emodin BAX

Dahuang DAH4 MOL000471 aloe-emodin TNF

Dahuang DAH4 MOL000471 aloe-emodin CASP3

Dahuang DAH4 MOL000471 aloe-emodin TP53

Dahuang DAH4 MOL000471 aloe-emodin FASN

Dahuang DAH4 MOL000471 aloe-emodin PRKCA

Dahuang DAH4 MOL000471 aloe-emodin PRKCE

Dahuang DAH4 MOL000471 aloe-emodin CDC2

Dahuang DAH4 MOL000471 aloe-emodin PCNA

Dahuang DAH4 MOL000471 aloe-emodin MYC

Dahuang DAH4 MOL000471 aloe-emodin IL1B

Dahuang DAH4 MOL000471 aloe-emodin PRKCD

Dahuang DAH4 MOL000471 aloe-emodin CCNB1

Dahuang DAH5 MOL000096 (-)-catechin PTGS1

Dahuang DAH5 MOL000096 (-)-catechin ESR1

Dahuang DAH5 MOL000096 (-)-catechin PTGS2

Dahuang DAH5 MOL000096 (-)-catechin HSP90

Dahuang DAH5 MOL000096 (-)-catechin PRKACA

Dahuang DAH5 MOL000096 (-)-catechin NCOA2

Dahuang DAH5 MOL000096 (-)-catechin FASN

Dahuang DAH5 MOL000096 (-)-catechin PPARG

Dahuang DAH5 MOL000096 (-)-catechin KLF7

Heshouwu HSW1 MOL000702 Guajavarin PTGS2

Heshouwu HSW1 MOL000702 Guajavarin PTPN1

Heshouwu HSW1 MOL000702 Guajavarin PRSS1

Heshouwu HSW2 MOL008647 Moupinamide MTNR1A

Heshouwu HSW2 MOL008647 Moupinamide MTNR1B

Heshouwu HSW2 MOL008647 Moupinamide HTR2C

Heshouwu HSW7 MOL002320 γ-sitosterol PGR

Heshouwu HSW7 MOL002320 γ-sitosterol NCOA2

Heshouwu C1 MOL002268 rhein PTGS1

Heshouwu C1 MOL002268 rhein PTGS2

Heshouwu C1 MOL002268 rhein HSP90

Heshouwu C1 MOL002268 rhein PIK3CG

Heshouwu C1 MOL002268 rhein NCOA2

Heshouwu C1 MOL002268 rhein JUN

Heshouwu HSW8 MOL012744 Resveratrol PTGS1

Heshouwu HSW8 MOL012744 Resveratrol PTGS2

Heshouwu HSW8 MOL012744 Resveratrol HSP90

Heshouwu HSW8 MOL012744 Resveratrol MAOB

Heshouwu HSW8 MOL012744 Resveratrol PRKACA

Heshouwu HSW8 MOL012744 Resveratrol NCOA2

Heshouwu HSW8 MOL012744 Resveratrol CA2

Heshouwu HSW8 MOL012744 Resveratrol RELA

Heshouwu HSW8 MOL012744 Resveratrol STAT3

Heshouwu HSW8 MOL012744 Resveratrol AKT1

Heshouwu HSW8 MOL012744 Resveratrol VEGFA

Heshouwu HSW8 MOL012744 Resveratrol CCND1

Heshouwu HSW8 MOL012744 Resveratrol BCL2

Heshouwu HSW8 MOL012744 Resveratrol BCL2L1

Heshouwu HSW8 MOL012744 Resveratrol FOS

Heshouwu HSW8 MOL012744 Resveratrol CDKN1A

Heshouwu HSW8 MOL012744 Resveratrol EIF6

Heshouwu HSW8 MOL012744 Resveratrol NFKBIA

Heshouwu HSW8 MOL012744 Resveratrol BAX

Heshouwu HSW8 MOL012744 Resveratrol CASP9

Heshouwu HSW8 MOL012744 Resveratrol PLAU

Heshouwu HSW8 MOL012744 Resveratrol MMP2

Heshouwu HSW8 MOL012744 Resveratrol MMP9

Heshouwu HSW8 MOL012744 Resveratrol MAPK3

Heshouwu HSW8 MOL012744 Resveratrol MAPK1

Heshouwu HSW8 MOL012744 Resveratrol IL10

Heshouwu HSW8 MOL012744 Resveratrol CDK4

Heshouwu HSW8 MOL012744 Resveratrol TNF

Heshouwu HSW8 MOL012744 Resveratrol JUN

Heshouwu HSW8 MOL012744 Resveratrol IL6

Heshouwu HSW8 MOL012744 Resveratrol CDK6

Heshouwu HSW8 MOL012744 Resveratrol AHSA1

Heshouwu HSW8 MOL012744 Resveratrol CASP3

Heshouwu HSW8 MOL012744 Resveratrol TP53

Heshouwu HSW8 MOL012744 Resveratrol MAPK8

Heshouwu HSW8 MOL012744 Resveratrol NFKBIA

Heshouwu HSW8 MOL012744 Resveratrol ODC1

Heshouwu HSW8 MOL012744 Resveratrol XDH

Heshouwu HSW8 MOL012744 Resveratrol BAK1

Heshouwu HSW8 MOL012744 Resveratrol BBC3

Heshouwu HSW8 MOL012744 Resveratrol SOD1

Heshouwu HSW8 MOL012744 Resveratrol CAT

Heshouwu HSW8 MOL012744 Resveratrol PRKCA

Heshouwu HSW8 MOL012744 Resveratrol TEP1

Heshouwu HSW8 MOL012744 Resveratrol HIF1A

Heshouwu HSW8 MOL012744 Resveratrol IGF1R

Heshouwu HSW8 MOL012744 Resveratrol STAT1

Heshouwu HSW8 MOL012744 Resveratrol RUNX1T1

Heshouwu HSW8 MOL012744 Resveratrol HERC5

Heshouwu HSW8 MOL012744 Resveratrol CDC2

Heshouwu HSW8 MOL012744 Resveratrol EDN1

Heshouwu HSW8 MOL012744 Resveratrol FOXO1

Heshouwu HSW8 MOL012744 Resveratrol HBEGF

Heshouwu HSW8 MOL012744 Resveratrol PPARG

Heshouwu HSW8 MOL012744 Resveratrol CTNNB1

Heshouwu HSW8 MOL012744 Resveratrol MYC

Heshouwu HSW8 MOL012744 Resveratrol F3

Heshouwu HSW8 MOL012744 Resveratrol GJA1

Heshouwu HSW8 MOL012744 Resveratrol CYP1A1

Heshouwu HSW8 MOL012744 Resveratrol ICAM1

Heshouwu HSW8 MOL012744 Resveratrol IL1B

Heshouwu HSW8 MOL012744 Resveratrol CCL2

Heshouwu HSW8 MOL012744 Resveratrol SELE

Heshouwu HSW8 MOL012744 Resveratrol VCAM1

Heshouwu HSW8 MOL012744 Resveratrol PRKCD

Heshouwu HSW8 MOL012744 Resveratrol CXCL8

Heshouwu HSW8 MOL012744 Resveratrol MCL1

Heshouwu HSW8 MOL012744 Resveratrol BRCA1

Heshouwu HSW8 MOL012744 Resveratrol XRCC6

Heshouwu HSW8 MOL012744 Resveratrol SOD2

Heshouwu HSW8 MOL012744 Resveratrol PRKCB

Heshouwu HSW8 MOL012744 Resveratrol PECAM1

Heshouwu HSW8 MOL012744 Resveratrol BIRC5

Heshouwu HSW8 MOL012744 Resveratrol DUOX2

Heshouwu HSW8 MOL012744 Resveratrol NOS3

Heshouwu HSW8 MOL012744 Resveratrol PTPN1

Heshouwu HSW8 MOL012744 Resveratrol CCND2

Heshouwu HSW8 MOL012744 Resveratrol BCL2A1

Heshouwu HSW8 MOL012744 Resveratrol MRP1

Heshouwu HSW8 MOL012744 Resveratrol HGF

Heshouwu HSW8 MOL012744 Resveratrol TGFB1

Heshouwu HSW8 MOL012744 Resveratrol MGAM

Heshouwu HSW8 MOL012744 Resveratrol CYP1B1

Heshouwu HSW8 MOL012744 Resveratrol CCNB1

Heshouwu HSW8 MOL012744 Resveratrol DDIT3

Heshouwu HSW8 MOL012744 Resveratrol PLAT

Heshouwu HSW8 MOL012744 Resveratrol PTEN

Heshouwu HSW8 MOL012744 Resveratrol CDK7

Heshouwu HSW8 MOL012744 Resveratrol IL1A

Heshouwu HSW8 MOL012744 Resveratrol MPO

Heshouwu HSW8 MOL012744 Resveratrol TNFRSF10B

Heshouwu HSW8 MOL012744 Resveratrol ABCG2

Heshouwu HSW8 MOL012744 Resveratrol NFE2L2

Heshouwu HSW8 MOL012744 Resveratrol NQO1

Heshouwu HSW8 MOL012744 Resveratrol XIAP

Heshouwu HSW8 MOL012744 Resveratrol AHR

Heshouwu HSW8 MOL012744 Resveratrol PRKAG2

Heshouwu HSW8 MOL012744 Resveratrol SLC2A4

Heshouwu HSW8 MOL012744 Resveratrol PPARA

Heshouwu HSW8 MOL012744 Resveratrol CRP

Heshouwu HSW8 MOL012744 Resveratrol PON1

Heshouwu HSW8 MOL012744 Resveratrol CD80

Heshouwu HSW8 MOL012744 Resveratrol CCNE1

Heshouwu HSW8 MOL012744 Resveratrol CCNE2

Heshouwu HSW8 MOL012744 Resveratrol BCAM

Heshouwu HSW8 MOL012744 Resveratrol SIRT1

Heshouwu HSW8 MOL012744 Resveratrol CREBZF

Heshouwu HSW8 MOL012744 Resveratrol NTRK1

Heshouwu HSW8 MOL012744 Resveratrol KLF10

Heshouwu HSW8 MOL012744 Resveratrol APAF1

Heshouwu HSW8 MOL012744 Resveratrol ABCC3

Heshouwu HSW8 MOL012744 Resveratrol SREBF1

Heshouwu HSW8 MOL012744 Resveratrol ADIPOR1

Heshouwu HSW8 MOL012744 Resveratrol ADIPOR2

Heshouwu HSW8 MOL012744 Resveratrol JAK1

Heshouwu HSW8 MOL012744 Resveratrol AGTR1

Heshouwu HSW8 MOL012744 Resveratrol IRS1

Heshouwu HSW8 MOL012744 Resveratrol CFLAR

Heshouwu HSW8 MOL012744 Resveratrol LGSN

Heshouwu HSW8 MOL012744 Resveratrol PYGO1

Heshouwu HSW8 MOL012744 Resveratrol IL17B

Heshouwu HSW8 MOL012744 Resveratrol SIRT2

Heshouwu HSW8 MOL012744 Resveratrol C5AR1

Heshouwu HSW8 MOL012744 Resveratrol MTOR

Heshouwu HSW8 MOL012744 Resveratrol CCR2

Heshouwu HSW8 MOL012744 Resveratrol EIF2S1

Heshouwu HSW8 MOL012744 Resveratrol PTGES

Heshouwu HSW8 MOL012744 Resveratrol PMAIP1

Heshouwu HSW8 MOL012744 Resveratrol BCL2L11

Heshouwu HSW8 MOL012744 Resveratrol TNFSF10

Heshouwu HSW8 MOL012744 Resveratrol TNFRSF10A

Heshouwu HSW8 MOL012744 Resveratrol RPS6

Heshouwu HSW8 MOL012744 Resveratrol GCH1

Heshouwu HSW8 MOL012744 Resveratrol BIRC3

Heshouwu HSW8 MOL012744 Resveratrol TRAF2

Heshouwu HSW8 MOL012744 Resveratrol CDC42

Heshouwu HSW8 MOL012744 Resveratrol BSG

Heshouwu HSW8 MOL012744 Resveratrol COL11A2

Heshouwu HSW8 MOL012744 Resveratrol ITGB1

Heshouwu HSW8 MOL012744 Resveratrol CD28

Heshouwu HSW8 MOL012744 Resveratrol NR1H3

Heshouwu HSW8 MOL012744 Resveratrol NFKB1

Heshouwu HSW8 MOL012744 Resveratrol PAPPA

Heshouwu HSW8 MOL012744 Resveratrol SPARC

Heshouwu HSW8 MOL012744 Resveratrol BRCA2

Heshouwu HSW8 MOL012744 Resveratrol AAGAB

Heshouwu HSW8 MOL012744 Resveratrol PRKD1

Heshouwu HSW8 MOL012744 Resveratrol TGFA

Heshouwu HSW8 MOL012744 Resveratrol CD320

Heshouwu HSW3 Citreorosein Citreorosein ESR1

Heshouwu HSW3 Citreorosein Citreorosein ESR2

Heshouwu HSW3 Citreorosein Citreorosein PIM1

Heshouwu HSW3 Citreorosein Citreorosein CSNK2A1

Heshouwu HSW3 Citreorosein Citreorosein PTP4A3

Heshouwu HSW3 Citreorosein Citreorosein FTO

Heshouwu HSW3 Citreorosein Citreorosein ELANE

Heshouwu HSW3 Citreorosein Citreorosein CYP19A1

Heshouwu HSW3 Citreorosein Citreorosein FNTA FNTB

Heshouwu HSW3 Citreorosein Citreorosein MCL1

Heshouwu HSW3 Citreorosein Citreorosein LIMK1

Heshouwu HSW3 Citreorosein Citreorosein BCL2

Heshouwu HSW3 Citreorosein Citreorosein LDHA

Heshouwu HSW3 Citreorosein Citreorosein LDHB

Heshouwu HSW3 Citreorosein Citreorosein NOX4

Heshouwu HSW3 Citreorosein Citreorosein CDK5R1 CDK5

Heshouwu HSW3 Citreorosein Citreorosein XDH

Heshouwu HSW3 Citreorosein Citreorosein FLT3

Heshouwu HSW3 Citreorosein Citreorosein CCNB3 CDK1 CCNB1 CCNB2

Heshouwu HSW3 Citreorosein Citreorosein GLO1

Heshouwu HSW3 Citreorosein Citreorosein APP

Heshouwu HSW3 Citreorosein Citreorosein SYK

Heshouwu HSW3 Citreorosein Citreorosein GSK3B

Heshouwu HSW3 Citreorosein Citreorosein PARP1

Heshouwu HSW3 Citreorosein Citreorosein TTR

Heshouwu HSW3 Citreorosein Citreorosein MMP9

Heshouwu HSW3 Citreorosein Citreorosein MMP2

Heshouwu HSW3 Citreorosein Citreorosein MMP12

Heshouwu HSW3 Citreorosein Citreorosein CD38

Heshouwu HSW3 Citreorosein Citreorosein CYP1B1

Heshouwu HSW3 Citreorosein Citreorosein ABCG2

Heshouwu HSW3 Citreorosein Citreorosein AKR1B10

Heshouwu HSW3 Citreorosein Citreorosein TNKS2

Heshouwu HSW3 Citreorosein Citreorosein TNKS

Heshouwu HSW3 Citreorosein Citreorosein TOP1

Heshouwu HSW3 Citreorosein Citreorosein ADORA1

Heshouwu HSW3 Citreorosein Citreorosein ARG1

Heshouwu HSW3 Citreorosein Citreorosein PTPRS

Heshouwu HSW3 Citreorosein Citreorosein PLK1

Heshouwu HSW3 Citreorosein Citreorosein CDK6

Heshouwu HSW3 Citreorosein Citreorosein CDK2

Heshouwu HSW3 Citreorosein Citreorosein DAPK1

Heshouwu HSW3 Citreorosein Citreorosein SLC22A12

Heshouwu HSW3 Citreorosein Citreorosein PPARG

Heshouwu HSW3 Citreorosein Citreorosein ABCC1

Heshouwu HSW3 Citreorosein Citreorosein AHR

Heshouwu HSW3 Citreorosein Citreorosein ESRRA

Heshouwu HSW3 Citreorosein Citreorosein ABCB1

Heshouwu HSW3 Citreorosein Citreorosein F2

Heshouwu HSW3 Citreorosein Citreorosein DRD4

Heshouwu HSW3 Citreorosein Citreorosein MPO

Heshouwu HSW3 Citreorosein Citreorosein PIK3R1

Heshouwu HSW3 Citreorosein Citreorosein PYGL

Heshouwu HSW3 Citreorosein Citreorosein MMP13

Heshouwu HSW3 Citreorosein Citreorosein MMP3

Heshouwu HSW3 Citreorosein Citreorosein CDK1

Heshouwu HSW3 Citreorosein Citreorosein PKN1

Heshouwu HSW3 Citreorosein Citreorosein CAMK2B

Heshouwu HSW3 Citreorosein Citreorosein AKT1

Heshouwu HSW4 MOL002083 tricin NOS2

Heshouwu HSW4 MOL002083 tricin ESR1

Heshouwu HSW4 MOL002083 tricin AR

Heshouwu HSW4 MOL002083 tricin PPARG

Heshouwu HSW4 MOL002083 tricin PTGS2

Heshouwu HSW4 MOL002083 tricin F7

Heshouwu HSW4 MOL002083 tricin ESR2

Heshouwu HSW4 MOL002083 tricin DPP4

Heshouwu HSW4 MOL002083 tricin MAPK14

Heshouwu HSW4 MOL002083 tricin GSK3B

Heshouwu HSW4 MOL002083 tricin HSP90

Heshouwu HSW4 MOL002083 tricin CDK2

Heshouwu HSW4 MOL002083 tricin PRSS1

Heshouwu HSW4 MOL002083 tricin NCOA2

Heshouwu HSW5 MOL000513 3,4,5-trihydroxybenzoic acid PTGS1

Heshouwu HSW5 MOL000513 3,4,5-trihydroxybenzoic acid PTGS2

Heshouwu HSW5 MOL000513 3,4,5-trihydroxybenzoic acid MAOB

Heshouwu HSW5 MOL000513 3,4,5-trihydroxybenzoic acid PGR

Heshouwu HSW5 MOL000513 3,4,5-trihydroxybenzoic acid PTPN1

Heshouwu HSW5 MOL000513 3,4,5-trihydroxybenzoic acid HSP90

Heshouwu HSW5 MOL000513 3,4,5-trihydroxybenzoic acid PIK3CG

Heshouwu HSW5 MOL000513 3,4,5-trihydroxybenzoic acid CASP9

Heshouwu HSW5 MOL000513 3,4,5-trihydroxybenzoic acid CASP3

Heshouwu HSW5 MOL000513 3,4,5-trihydroxybenzoic acid TP53

Heshouwu HSW5 MOL000513 3,4,5-trihydroxybenzoic acid FASN

Heshouwu HSW5 MOL000513 3,4,5-trihydroxybenzoic acid FASLG

Heshouwu HSW5 MOL000513 3,4,5-trihydroxybenzoic acid MGST1

Heshouwu HSW5 MOL000513 3,4,5-trihydroxybenzoic acid CYP3A43

Heshouwu HSW6 MOL00051 何首乌苷 COL1A1

Heshouwu HSW6 MOL00051 何首乌苷 COL4A2

Heshouwu HSW6 MOL00051 何首乌苷 HMOX1

Heshouwu HSW6 MOL00051 何首乌苷 KEAP1

Heshouwu HSW6 MOL00051 何首乌苷 NFE2L2

Heshouwu HSW6 MOL00051 何首乌苷 NPHS2

Heshouwu HSW6 MOL00051 何首乌苷 NQO1

Heshouwu HSW6 MOL00051 何首乌苷 PI3K

Heshouwu HSW6 MOL00051 何首乌苷 AKT1

Heshouwu HSW6 MOL00051 何首乌苷 SIRT1

Tianma TM1 MOL001843 p-Hydroxybenzaldehyde ADH1B

Tianma TM1 MOL001843 p-Hydroxybenzaldehyde ADH1C

Tianma TM1 MOL001843 p-Hydroxybenzaldehyde PDXK

Tianma TM2 MOL000635 vanillin MAOA

Tianma TM2 MOL000635 vanillin PTGS2

Tianma TM2 MOL000635 vanillin GABRA1

Tianma TM2 MOL000635 vanillin ADH1C

Tianma TM2 MOL000635 vanillin MMP9

Tianma TM2 MOL000635 vanillin MAPK1

Tianma TM2 MOL000635 vanillin JUN

Tianma A1 MOL000449 Stigmasterol PGR

Tianma A1 MOL000449 Stigmasterol NR3C2

Tianma A1 MOL000449 Stigmasterol NCOA2

Tianma A1 MOL000449 Stigmasterol ADH1C

Tianma A1 MOL000449 Stigmasterol IGHG1

Tianma A1 MOL000449 Stigmasterol RXRA

Tianma A1 MOL000449 Stigmasterol NCOA1

Tianma A1 MOL000449 Stigmasterol PTGS1

Tianma A1 MOL000449 Stigmasterol PTGS2

Tianma A1 MOL000449 Stigmasterol ADRA2A

Tianma A1 MOL000449 Stigmasterol SLC6A2

Tianma A1 MOL000449 Stigmasterol SLC6A3

Tianma A1 MOL000449 Stigmasterol ADRB2

Tianma A1 MOL000449 Stigmasterol PLAU

Tianma A1 MOL000449 Stigmasterol LTA4H

Tianma A1 MOL000449 Stigmasterol MAOB

Tianma A1 MOL000449 Stigmasterol MAOA

Tianma A1 MOL000449 Stigmasterol PRKACA

Tianma A1 MOL000449 Stigmasterol CTRB1

Tianma A1 MOL000449 Stigmasterol CHRM3

Tianma A1 MOL000449 Stigmasterol CHRM1

Tianma A1 MOL000449 Stigmasterol ADRB1

Tianma A1 MOL000449 Stigmasterol SCN5A

Tianma A1 MOL000449 Stigmasterol HTR2A

Tianma A1 MOL000449 Stigmasterol ADRA1A

Tianma A1 MOL000449 Stigmasterol GABRA3

Tianma A1 MOL000449 Stigmasterol CHRM2

Tianma A1 MOL000449 Stigmasterol ADRA1B

Tianma A1 MOL000449 Stigmasterol GABRA1

Tianma A1 MOL000449 Stigmasterol CHRNA7

Tianma B1 MOL000358 beta-sitosterol RELA

Tianma B1 MOL000358 beta-sitosterol NR3C2

Tianma B1 MOL000358 beta-sitosterol PGR

Tianma B1 MOL000358 beta-sitosterol NCOA2

Tianma B1 MOL000358 beta-sitosterol PTGS1

Tianma B1 MOL000358 beta-sitosterol PTGS2

Tianma B1 MOL000358 beta-sitosterol HSP90

Tianma B1 MOL000358 beta-sitosterol PI3K

Tianma B1 MOL000358 beta-sitosterol KCNH2

Tianma B1 MOL000358 beta-sitosterol PRKACA

Tianma B1 MOL000358 beta-sitosterol CHRM3

Tianma B1 MOL000358 beta-sitosterol CHRM1

Tianma B1 MOL000358 beta-sitosterol SCN5A

Tianma B1 MOL000358 beta-sitosterol GABRA2

Tianma B1 MOL000358 beta-sitosterol CHRM4

Tianma B1 MOL000358 beta-sitosterol PDE3A

Tianma B1 MOL000358 beta-sitosterol HTR2A

Tianma B1 MOL000358 beta-sitosterol GABRA5

Tianma B1 MOL000358 beta-sitosterol ADRA1A

Tianma B1 MOL000358 beta-sitosterol GABRA3

Tianma B1 MOL000358 beta-sitosterol CHRM2

Tianma B1 MOL000358 beta-sitosterol ADRA1B

Tianma B1 MOL000358 beta-sitosterol ADRB2

Tianma B1 MOL000358 beta-sitosterol CHRNA2

Tianma B1 MOL000358 beta-sitosterol SLC6A4

Tianma B1 MOL000358 beta-sitosterol OPRM1

Tianma B1 MOL000358 beta-sitosterol GABRA1

Tianma B1 MOL000358 beta-sitosterol CHRNA7

Tianma B1 MOL000358 beta-sitosterol BCL2

Tianma B1 MOL000358 beta-sitosterol BAX

Tianma B1 MOL000358 beta-sitosterol CASP9

Tianma B1 MOL000358 beta-sitosterol JUN

Tianma B1 MOL000358 beta-sitosterol CASP3

Tianma B1 MOL000358 beta-sitosterol CASP8

Tianma B1 MOL000358 beta-sitosterol PRKCA

Tianma B1 MOL000358 beta-sitosterol TGFB1

Tianma B1 MOL000358 beta-sitosterol PON1

Tianma B1 MOL000358 beta-sitosterol MAP2

Tianma TM3 MOL000295 Daucosterol IL2

Tianma TM3 MOL000295 Daucosterol STAT3

Tianma TM3 MOL000295 Daucosterol BCL2L1

Tianma TM3 MOL000295 Daucosterol PTAFR

Tianma TM3 MOL000295 Daucosterol PSEN2

Tianma TM3 MOL000295 Daucosterol F2

Tianma TM3 MOL000295 Daucosterol PPM1B

Tianma TM3 MOL000295 Daucosterol PTPN1

Tianma TM3 MOL000295 Daucosterol PPP1CC

Tianma TM3 MOL000295 Daucosterol PPP2CA

Tianma TM3 MOL000295 Daucosterol PPP2R5A

Tianma TM3 MOL000295 Daucosterol HSD11B2

Tianma TM3 MOL000295 Daucosterol HSD11B1

Tianma TM3 MOL000295 Daucosterol NPC1L1

Tianma TM3 MOL000295 Daucosterol HSP90AA1

Tianma TM4 MOL007986 Gastrodin TYR

Tianma TM4 MOL007986 Gastrodin SLC5A4

Tianma TM4 MOL007986 Gastrodin SLC5A2

Tianma TM4 MOL007986 Gastrodin SLC5A1

Tianma TM4 MOL007986 Gastrodin AKR1B1

Tianma TM4 MOL007986 Gastrodin TDP1

Tianma TM4 MOL007986 Gastrodin PTPN1

Tianma TM4 MOL007986 Gastrodin EPHX2

Shuizhi SZ1 MOL000511 ursolic acid PLAU

Shuizhi SZ1 MOL000511 ursolic acid CTSB

Shuizhi SZ1 MOL000511 ursolic acid RELA

Shuizhi SZ1 MOL000511 ursolic acid STAT3

Shuizhi SZ1 MOL000511 ursolic acid VEGFA

Shuizhi SZ1 MOL000511 ursolic acid CCND1

Shuizhi SZ1 MOL000511 ursolic acid BCL2

Shuizhi SZ1 MOL000511 ursolic acid BCL2L1

Shuizhi SZ1 MOL000511 ursolic acid FOS

Shuizhi SZ1 MOL000511 ursolic acid CDKN1A

Shuizhi SZ1 MOL000511 ursolic acid BAX

Shuizhi SZ1 MOL000511 ursolic acid CASP9

Shuizhi SZ1 MOL000511 ursolic acid MMP2

Shuizhi SZ1 MOL000511 ursolic acid MMP9

Shuizhi SZ1 MOL000511 ursolic acid CDK4

Shuizhi SZ1 MOL000511 ursolic acid TNF

Shuizhi SZ1 MOL000511 ursolic acid JUN

Shuizhi SZ1 MOL000511 ursolic acid IL6

Shuizhi SZ1 MOL000511 ursolic acid CDK6

Shuizhi SZ1 MOL000511 ursolic acid CASP3

Shuizhi SZ1 MOL000511 ursolic acid TP53

Shuizhi SZ1 MOL000511 ursolic acid MAPK8

Shuizhi SZ1 MOL000511 ursolic acid PTGS2

Shuizhi SZ1 MOL000511 ursolic acid NFKBIA

Shuizhi SZ1 MOL000511 ursolic acid CASP8

Shuizhi SZ1 MOL000511 ursolic acid FASN

Shuizhi SZ1 MOL000511 ursolic acid MMP1

Shuizhi SZ1 MOL000511 ursolic acid MMP3

Shuizhi SZ1 MOL000511 ursolic acid HERC5

Shuizhi SZ1 MOL000511 ursolic acid HBGF2

Shuizhi SZ1 MOL000511 ursolic acid MMP10

Shuizhi SZ1 MOL000511 ursolic acid ICAM1

Shuizhi SZ1 MOL000511 ursolic acid IL1B

Shuizhi SZ1 MOL000511 ursolic acid CREB1

Shuizhi SZ1 MOL000511 ursolic acid SELE

Shuizhi SZ1 MOL000511 ursolic acid PTGER3

Shuizhi SZ1 MOL000511 ursolic acid PTGS1

Shuizhi SZ1 MOL000511 ursolic acid MCL1

Shuizhi SZ1 MOL000511 ursolic acid PRKCG

Shuizhi SZ1 MOL000511 ursolic acid ATF2

Shuizhi SZ1 MOL000511 ursolic acid CSF2

Shuizhi SZ1 MOL000511 ursolic acid PECAM1

Shuizhi SZ1 MOL000511 ursolic acid MAPK8IP2

Shuizhi SZ1 MOL000511 ursolic acid BIRC5

Shuizhi SZ1 MOL000511 ursolic acid PTPN6

Shuizhi SZ1 MOL000511 ursolic acid GAP43

Shuizhi SZ1 MOL000511 ursolic acid DUOX2

Shuizhi SZ1 MOL000511 ursolic acid NOS3

Shuizhi SZ1 MOL000511 ursolic acid PTPN1

Shuizhi SZ1 MOL000511 ursolic acid LITAF

Shuizhi SZ1 MOL000511 ursolic acid CCND2

Shuizhi SZ1 MOL000511 ursolic acid FASLG

Shuizhi SZ1 MOL000511 ursolic acid CASP1

Shuizhi SZ1 MOL000511 ursolic acid ENPP7

Shuizhi SZ2 MOL001406 crocetin CHRM3

Shuizhi SZ2 MOL001406 crocetin CHRM1

Shuizhi SZ2 MOL001406 crocetin GABRB2

Shuizhi SZ2 MOL001406 crocetin GABRB5

Shuizhi SZ2 MOL001406 crocetin ADRA1A

Shuizhi SZ2 MOL001406 crocetin GABRB3

Shuizhi SZ2 MOL001406 crocetin CHRM2

Shuizhi SZ2 MOL001406 crocetin ADRA1B

Shuizhi SZ2 MOL001406 crocetin GABRA1

Shuizhi SZ2 MOL001406 crocetin IGHG1

Shuizhi SZ2 MOL001406 crocetin PTGS2

Shuizhi SZ2 MOL001406 crocetin NCOA2

Shuizhi SZ2 MOL001406 crocetin VCAM1

Shuizhi SZ3 MOL000003 MTL ADH1C

Shuizhi SZ3 MOL000003 MTL NPC1L1

Shuizhi SZ3 MOL000003 MTL NR1H3

Shuizhi SZ3 MOL000003 MTL DPP4

Shuizhi SZ3 MOL000003 MTL POLA1

Shuizhi SZ3 MOL000003 MTL NR1H4

Shuizhi SZ3 MOL000003 MTL GPBAR1

Shuizhi SZ3 MOL000003 MTL SHH

Shuizhi SZ3 MOL000003 MTL PYGB

Shuizhi SZ4 MOL004557 geniposide BCL2

Shuizhi SZ4 MOL004557 geniposide HMOX1

Shuizhi SZ4 MOL004557 geniposide GAP43

Shuizhi SZ4 MOL004557 geniposide PLB1

Shuizhi SZ4 MOL004557 geniposide GSTM1

Shuizhi SZ4 MOL004557 geniposide GSTM2

Shuizhi SZ5 crocetinate crocetinate AKT1

Shuizhi SZ5 crocetinate crocetinate BAX

Shuizhi SZ5 crocetinate crocetinate BCL2

Shuizhi SZ5 crocetinate crocetinate CASP3

Shuizhi SZ5 crocetinate crocetinate CAT

Shuizhi SZ5 crocetinate crocetinate IL1B

Shuizhi SZ5 crocetinate crocetinate TNF

Shuizhi SZ5 crocetinate crocetinate SIRT1

Shuizhi SZ5 crocetinate crocetinate RELA

Shuizhi SZ5 crocetinate crocetinate GPX1

Shuizhi SZ5 crocetinate crocetinate SOD1

Shuizhi SZ5 crocetinate crocetinate SOD2

Shuizhi SZ5 crocetinate crocetinate GPX4

Shuizhi SZ5 crocetinate crocetinate CCL2

Shuizhi SZ5 crocetinate crocetinate MPO

Shuizhi SZ5 crocetinate crocetinate MMP2

Shuizhi SZ5 crocetinate crocetinate MMP9

Quanxie QX1 MOL002156 Trimethylamine BCHE

Quanxie QX1 MOL002156 Trimethylamine ADH1B

Quanxie QX1 MOL002156 Trimethylamine ADH1C

Quanxie QX1 MOL002156 Trimethylamine ADH1A

Quanxie QX1 MOL002156 Trimethylamine GSTA1

Quanxie QX1 MOL002156 Trimethylamine ARG1

Quanxie QX1 MOL002156 Trimethylamine PLA2G2E

Quanxie QX1 MOL002156 Trimethylamine TPI1

Quanxie QX1 MOL002156 Trimethylamine GAMT

Quanxie QX1 MOL002156 Trimethylamine PRSS3

Quanxie QX1 MOL002156 Trimethylamine ALDH2

Quanxie QX1 MOL002156 Trimethylamine CELA1

Quanxie QX1 MOL002156 Trimethylamine ARG1

Quanxie QX1 MOL002156 Trimethylamine ANXA3

Quanxie QX1 MOL002156 Trimethylamine GM2A

Quanxie QX1 MOL002156 Trimethylamine GNMT

Quanxie QX2 MOL002223 TAU GATM

Quanxie QX2 MOL002223 TAU ME2

Quanxie QX3 MOL002307 20-Hexadecanoylingenol NR3C2

Quanxie QX3 MOL002307 20-Hexadecanoylingenol PKCA

Quanxie QX3 MOL002307 20-Hexadecanoylingenol EP3

Quanxie QX3 MOL002307 20-Hexadecanoylingenol EP2

Quanxie QX3 MOL002307 20-Hexadecanoylingenol PKCB

Quanxie QX4 MOL000953 Cholesterol PGR

Quanxie QX4 MOL000953 Cholesterol NR3C2

Quanxie QX4 MOL000953 Cholesterol NCOA2

Quanxie QX4 MOL000953 Cholesterol NFKB1

Quanxie QX4 MOL000953 Cholesterol IRX5

Quanxie QX4 MOL000953 Cholesterol SNAI1

Quanxie QX4 MOL000953 Cholesterol GPBAR1

Quanxie QX4 MOL000953 Cholesterol PGR

Quanxie QX4 MOL000953 Cholesterol GPER1

Quanxie QX5 MOL004082 Stearin PRKCG

Quanxie QX5 MOL004082 Stearin PRKCA

Quanxie QX5 MOL004082 Stearin PRKCE

Quanxie QX5 MOL004082 Stearin PRKCH

Quanxie QX5 MOL004082 Stearin PRKCQ

Quanxie QX5 MOL004082 Stearin PRKCD

Quanxie QX5 MOL004082 Stearin TRPV4

Quanxie QX5 MOL004082 Stearin ABCB1

Quanxie QX5 MOL004082 Stearin SIRT2

Quanxie QX5 MOL004082 Stearin HMGCR

Quanxie QX5 MOL004082 Stearin PSEN1

Quanxie QX5 MOL004082 Stearin PSEN2

Quanxie QX5 MOL004082 Stearin PAM

Quanxie QX5 MOL004082 Stearin OPRK1

Quanxie QX5 MOL004082 Stearin HSD11B1

Quanxie QX5 MOL004082 Stearin UGT2B7

Quanxie QX5 MOL004082 Stearin ENPP2

Yimucao YMC1 MOL001418 galeopsin CHRM1

Yimucao YMC1 MOL001418 galeopsin ADRB1

Yimucao YMC1 MOL001418 galeopsin PTGS2

Yimucao YMC1 MOL001418 galeopsin ADRA2A

Yimucao YMC1 MOL001418 galeopsin RXRA

Yimucao YMC1 MOL001418 galeopsin ACHE

Yimucao YMC1 MOL001418 galeopsin SLC6A2

Yimucao YMC1 MOL001418 galeopsin CHRM2

Yimucao YMC1 MOL001418 galeopsin ADRA2B

Yimucao YMC1 MOL001418 galeopsin SLC6A3

Yimucao YMC1 MOL001418 galeopsin ADRB2

Yimucao YMC1 MOL001418 galeopsin SLC6A4

Yimucao YMC1 MOL001418 galeopsin MAOB

Yimucao YMC2 MOL001420 ZINC04073977 PGR

Yimucao YMC2 MOL001420 ZINC04073977 PTGS2

Yimucao YMC2 MOL001420 ZINC04073977 GABRA1

Yimucao YMC2 MOL001420 ZINC04073977 ADH1C

Yimucao YMC3 MOL001421 preleoheterin CHRM3

Yimucao YMC3 MOL001421 preleoheterin CHRM1

Yimucao YMC3 MOL001421 preleoheterin PTGS2

Yimucao YMC3 MOL001421 preleoheterin PDE3A

Yimucao YMC3 MOL001421 preleoheterin SLC6A2

Yimucao YMC3 MOL001421 preleoheterin ADRA1B

Yimucao YMC3 MOL001421 preleoheterin ADRB2

Yimucao YMC3 MOL001421 preleoheterin SLC6A4

Yimucao YMC3 MOL001421 preleoheterin HSP90

Yimucao YMC3 MOL001421 preleoheterin PRKACA

Yimucao YMC4 MOL001422 iso-preleoheterin PTGS1

Yimucao YMC4 MOL001422 iso-preleoheterin CHRM1

Yimucao YMC4 MOL001422 iso-preleoheterin PTGS2

Yimucao YMC4 MOL001422 iso-preleoheterin RXRA

Yimucao YMC4 MOL001422 iso-preleoheterin SLC6A2

Yimucao YMC4 MOL001422 iso-preleoheterin GABRA1

Yimucao YMC4 MOL001422 iso-preleoheterin GRIA2

Yimucao D1 MOL000098 quercetin PTGS1

Yimucao D1 MOL000098 quercetin AR

Yimucao D1 MOL000098 quercetin PPARG

Yimucao D1 MOL000098 quercetin PTGS2

Yimucao D1 MOL000098 quercetin HSP90

Yimucao YMC5 MOL001439 arachidonic acid PTGS1

Yimucao YMC5 MOL001439 arachidonic acid PTGS2

Yimucao YMC5 MOL001439 arachidonic acid RXRA

Yimucao YMC5 MOL001439 arachidonic acid TRPV1

Yimucao YMC5 MOL001439 arachidonic acid RXRG

Yimucao YMC5 MOL001439 arachidonic acid SLC6A2

Yimucao YMC5 MOL001439 arachidonic acid RELA

Yimucao YMC5 MOL001439 arachidonic acid CCND1

Yimucao YMC5 MOL001439 arachidonic acid MAPK1

Yimucao YMC5 MOL001439 arachidonic acid EGF

Yimucao YMC5 MOL001439 arachidonic acid CDK4

Yimucao YMC5 MOL001439 arachidonic acid CASP3

Yimucao YMC5 MOL001439 arachidonic acid HERC5

Yimucao YMC5 MOL001439 arachidonic acid PPARG

Yimucao YMC5 MOL001439 arachidonic acid G6PD

Yimucao YMC5 MOL001439 arachidonic acid TNFRSF1A

Yimucao YMC5 MOL001439 arachidonic acid PRKCB

Yimucao YMC5 MOL001439 arachidonic acid PECAM1

Yimucao YMC5 MOL001439 arachidonic acid NOS3

Yimucao YMC5 MOL001439 arachidonic acid ALOX5

Yimucao YMC5 MOL001439 arachidonic acid PLA2G4A

Yimucao YMC5 MOL001439 arachidonic acid PTEN

Yimucao YMC5 MOL001439 arachidonic acid SELP

Yimucao YMC5 MOL001439 arachidonic acid PTGES

Yimucao YMC5 MOL001439 arachidonic acid GLB1

Yimucao YMC5 MOL001439 arachidonic acid ALDH2

Yimucao YMC5 MOL001439 arachidonic acid ALDH3A1

Yimucao YMC5 MOL001439 arachidonic acid UCP2

Yimucao YMC5 MOL001439 arachidonic acid C1R

Yimucao YMC5 MOL001439 arachidonic acid CETP

Yimucao YMC5 MOL001439 arachidonic acid ABCG1

Yimucao YMC5 MOL001439 arachidonic acid ABCC4

Yimucao YMC5 MOL001439 arachidonic acid KCNK10

Yimucao YMC5 MOL001439 arachidonic acid TNFRSF1B

Yimucao YMC5 MOL001439 arachidonic acid PTGES2

Yimucao YMC5 MOL001439 arachidonic acid KCNK2

Yimucao YMC6 MOL000354 isorhamnetin NOS2

Yimucao YMC6 MOL000354 isorhamnetin PTGS1

Yimucao YMC6 MOL000354 isorhamnetin ESR1

Yimucao YMC6 MOL000354 isorhamnetin AR

Yimucao YMC6 MOL000354 isorhamnetin PPARG

Yimucao YMC6 MOL000354 isorhamnetin PTGS2

Yimucao YMC6 MOL000354 isorhamnetin PTPN1

Yimucao YMC6 MOL000354 isorhamnetin ESR2

Yimucao YMC6 MOL000354 isorhamnetin DPP4

Yimucao YMC6 MOL000354 isorhamnetin MAPK14

Yimucao YMC6 MOL000354 isorhamnetin GSK3B

Yimucao YMC6 MOL000354 isorhamnetin HSP90

Yimucao YMC6 MOL000354 isorhamnetin CDK2

Yimucao YMC6 MOL000354 isorhamnetin PIK3CG

Yimucao YMC6 MOL000354 isorhamnetin PRKACA

Yimucao YMC6 MOL000354 isorhamnetin PRSS1

Yimucao YMC6 MOL000354 isorhamnetin CCNA2

Yimucao YMC6 MOL000354 isorhamnetin NCOA2

Yimucao YMC6 MOL000354 isorhamnetin PYGM

Yimucao YMC6 MOL000354 isorhamnetin PPARG

Yimucao YMC6 MOL000354 isorhamnetin CHEK1

Yimucao YMC6 MOL000354 isorhamnetin NCOA1

Yimucao YMC6 MOL000354 isorhamnetin F7

Yimucao YMC6 MOL000354 isorhamnetin NOS2

Yimucao YMC6 MOL000354 isorhamnetin ACHE

Yimucao YMC6 MOL000354 isorhamnetin GABRA1

Yimucao YMC6 MOL000354 isorhamnetin MAOB

Yimucao YMC6 MOL000354 isorhamnetin GRIA2

Yimucao YMC6 MOL000354 isorhamnetin RELA

Yimucao YMC6 MOL000354 isorhamnetin XDH

Yimucao YMC6 MOL000354 isorhamnetin NCF1

Yimucao YMC6 MOL000354 isorhamnetin OLR1

Yimucao YMC7 MOL000422 kaempferol NOS2

Yimucao YMC7 MOL000422 kaempferol PTGS1

Yimucao YMC7 MOL000422 kaempferol AR

Yimucao YMC7 MOL000422 kaempferol PPARG

Yimucao YMC7 MOL000422 kaempferol PTGS2

Yimucao YMC7 MOL000422 kaempferol HSP90

Yimucao YMC7 MOL000422 kaempferol PIK3CG

Yimucao YMC7 MOL000422 kaempferol PRKACA

Yimucao YMC7 MOL000422 kaempferol NCOA2

Yimucao YMC7 MOL000422 kaempferol RELA

Yimucao YMC7 MOL000422 kaempferol IKBKB

Yimucao YMC7 MOL000422 kaempferol AKT1

Yimucao YMC7 MOL000422 kaempferol BCL2

Yimucao YMC7 MOL000422 kaempferol BAX

Yimucao YMC7 MOL000422 kaempferol TNF

Yimucao YMC7 MOL000422 kaempferol JUN

Yimucao YMC7 MOL000422 kaempferol AHSA1

Yimucao YMC7 MOL000422 kaempferol CASP3

Yimucao YMC7 MOL000422 kaempferol MAPK8

Yimucao YMC7 MOL000422 kaempferol XDH

Yimucao YMC7 MOL000422 kaempferol MMP1

Zhebeimu ZB1 MOL001004 pelargonidin CDH1

Zhebeimu ZB1 MOL001004 pelargonidin ICAM1

Zhebeimu ZB1 MOL001004 pelargonidin IL6

Zhebeimu ZB1 MOL001004 pelargonidin MAPK1

Zhebeimu ZB1 MOL001004 pelargonidin RELA

Zhebeimu ZB1 MOL001004 pelargonidin TLR4

Zhebeimu ZB1 MOL001004 pelargonidin TNF

Zhebeimu ZB1 MOL001004 pelargonidin VCAM1

Zhebeimu ZB1 MOL001004 pelargonidin AHR

Zhebeimu ZB1 MOL001004 pelargonidin CYP1A1

Zhebeimu ZB1 MOL001004 pelargonidin CYP1A2

Zhebeimu ZB1 MOL001004 pelargonidin NOS2

Zhebeimu ZB1 MOL001004 pelargonidin PTGS2

Zhebeimu ZB1 MOL001004 pelargonidin TNF

Zhebeimu ZB1 MOL001004 pelargonidin PTGES

Zhebeimu ZB1 MOL001004 pelargonidin STAT1

Zhebeimu ZB1 MOL001004 pelargonidin BAX

Zhebeimu ZB1 MOL001004 pelargonidin BCL2

Zhebeimu ZB1 MOL001004 pelargonidin CASP3

Zhebeimu ZB1 MOL001004 pelargonidin MAPK3

Zhebeimu ZB1 MOL001004 pelargonidin PARP1

Zhebeimu ZB2 MOL004440 Peimisine SHH

Zhebeimu ZB2 MOL004440 Peimisine EBP

Zhebeimu ZB2 MOL004440 Peimisine SMO

Zhebeimu ZB2 MOL004440 Peimisine ESRRG

Zhebeimu ZB2 MOL004440 Peimisine AKT2

Zhebeimu ZB2 MOL004440 Peimisine AKT1

Zhebeimu ZB2 MOL004440 Peimisine AKT3

Zhebeimu ZB2 MOL004440 Peimisine TNK2

Zhebeimu ZB2 MOL004440 Peimisine JAK3

Zhebeimu ZB2 MOL004440 Peimisine JAK1

Zhebeimu ZB2 MOL004440 Peimisine XIAP

Zhebeimu ZB2 MOL004440 Peimisine HRH3

Zhebeimu ZB2 MOL004440 Peimisine MAP3K14

Zhebeimu ZB2 MOL004440 Peimisine DGAT1

Zhebeimu ZB2 MOL004440 Peimisine CCR1

Zhebeimu ZB2 MOL004440 Peimisine CCR3

Zhebeimu ZB2 MOL004440 Peimisine PIM1

Zhebeimu ZB2 MOL004440 Peimisine CTSC

Zhebeimu ZB2 MOL004440 Peimisine PIM2

Zhebeimu ZB2 MOL004440 Peimisine PIM3

Zhebeimu ZB2 MOL004440 Peimisine FDFT1

Zhebeimu ZB2 MOL004440 Peimisine CSF1R

Zhebeimu ZB2 MOL004440 Peimisine VDR

Zhebeimu ZB2 MOL004440 Peimisine PRKG1

Zhebeimu ZB3 MOL004443 Zhebeiresinol ALOX5

Zhebeimu ZB3 MOL004443 Zhebeiresinol ERN1

Zhebeimu ZB3 MOL004443 Zhebeiresinol ELAVL1

Zhebeimu ZB3 MOL004443 Zhebeiresinol HSD17B3

Zhebeimu ZB3 MOL004443 Zhebeiresinol PIK3CD

Zhebeimu ZB3 MOL004443 Zhebeiresinol PRKDC

Zhebeimu ZB3 MOL004443 Zhebeiresinol PIK3CB

Zhebeimu ZB3 MOL004443 Zhebeiresinol PDE10A

Zhebeimu ZB3 MOL004443 Zhebeiresinol EIF2AK3

Zhebeimu ZB3 MOL004443 Zhebeiresinol PTAFR

Zhebeimu ZB3 MOL004443 Zhebeiresinol CDK1

Zhebeimu ZB3 MOL004443 Zhebeiresinol PIK3CG

Zhebeimu ZB3 MOL004443 Zhebeiresinol PIK3CA

Zhebeimu ZB3 MOL004443 Zhebeiresinol PARP1

Zhebeimu ZB4 MOL004444 Ziebeimine CHRM2

Zhebeimu ZB4 MOL004444 Ziebeimine CHRM1

Zhebeimu ZB4 MOL004444 Ziebeimine KCNH2

Zhebeimu ZB4 MOL004444 Ziebeimine OPRK1

Zhebeimu ZB4 MOL004444 Ziebeimine SLC6A2

Zhebeimu ZB4 MOL004444 Ziebeimine AKT2

Zhebeimu ZB4 MOL004444 Ziebeimine AKT1

Zhebeimu ZB4 MOL004444 Ziebeimine AKT3

Zhebeimu ZB4 MOL004444 Ziebeimine SIGMAR1

Zhebeimu ZB4 MOL004444 Ziebeimine HTR7

Zhebeimu ZB4 MOL004444 Ziebeimine HTR6

Zhebeimu ZB4 MOL004444 Ziebeimine HTR5A

Zhebeimu ZB4 MOL004444 Ziebeimine XIAP

Zhebeimu ZB4 MOL004444 Ziebeimine BIRC3

Zhebeimu ZB4 MOL004444 Ziebeimine BIRC2

Zhebeimu ZB4 MOL004444 Ziebeimine HRH3

Zhebeimu ZB4 MOL004444 Ziebeimine ATP12A

Zhebeimu ZB4 MOL004444 Ziebeimine OPRD1

Zhebeimu ZB4 MOL004444 Ziebeimine CCR1

Zhebeimu ZB4 MOL004444 Ziebeimine CCR3

Zhebeimu ZB4 MOL004444 Ziebeimine CTSS

Zhebeimu ZB4 MOL004444 Ziebeimine MDM2

Zhebeimu ZB4 MOL004444 Ziebeimine PRKG1

Zhebeimu ZB4 MOL004444 Ziebeimine HTR1A

Zhebeimu ZB4 MOL004444 Ziebeimine TTK

Zhebeimu ZB4 MOL004444 Ziebeimine AR

Zhebeimu ZB4 MOL004444 Ziebeimine TACR1

Zhebeimu ZB4 MOL004444 Ziebeimine TACR2

Zhebeimu ZB4 MOL004444 Ziebeimine TACR3

Zhebeimu ZB4 MOL004444 Ziebeimine NMT1

Zhebeimu ZB4 MOL004444 Ziebeimine CHKA

Zhebeimu ZB4 MOL004444 Ziebeimine ADRA2A

Zhebeimu ZB4 MOL004444 Ziebeimine ADRA2C

Zhebeimu ZB4 MOL004444 Ziebeimine ADRA2B

Zhebeimu ZB4 MOL004444 Ziebeimine KDR

Zhebeimu ZB4 MOL004444 Ziebeimine INCENP AURKB

Zhebeimu ZB4 MOL004444 Ziebeimine AURKA

Zhebeimu ZB4 MOL004444 Ziebeimine SOAT1

Zhebeimu ZB4 MOL004444 Ziebeimine OGFRL1

Zhebeimu ZB4 MOL004444 Ziebeimine EP300

Zhebeimu ZB4 MOL004444 Ziebeimine TP53

Zhebeimu ZB4 MOL004444 Ziebeimine CREBBP

Zhebeimu ZB4 MOL004444 Ziebeimine HTR1B

Zhebeimu ZB4 MOL004444 Ziebeimine CCND1 CDK4

Zhebeimu ZB4 MOL004444 Ziebeimine HTR1D

Zhebeimu ZB4 MOL004444 Ziebeimine CCNE2 CDK2 CCNE1

Zhebeimu ZB4 MOL004444 Ziebeimine CTSD

Zhebeimu ZB4 MOL004444 Ziebeimine PRKCD

Zhebeimu ZB4 MOL004444 Ziebeimine PRKCQ

Zhebeimu ZB4 MOL004444 Ziebeimine BACE1

Zhebeimu ZB4 MOL004444 Ziebeimine DPP4

Zhebeimu ZB4 MOL004444 Ziebeimine NPY1R

Zhebeimu ZB4 MOL004444 Ziebeimine ACHE

Zhebeimu ZB4 MOL004444 Ziebeimine HTR2A

Zhebeimu ZB4 MOL004444 Ziebeimine PDE10A

Zhebeimu ZB4 MOL004444 Ziebeimine REN

Zhebeimu ZB4 MOL004444 Ziebeimine SCN9A

Zhebeimu ZB4 MOL004444 Ziebeimine OPRL1

Zhebeimu ZB4 MOL004444 Ziebeimine MAP3K14

Zhebeimu ZB4 MOL004444 Ziebeimine HRH1

Zhebeimu ZB4 MOL004444 Ziebeimine MCHR1

Zhebeimu ZB4 MOL004444 Ziebeimine IDH1

Zhebeimu ZB4 MOL004444 Ziebeimine PDE9A

Zhebeimu ZB4 MOL004444 Ziebeimine PDE1C

Zhebeimu ZB4 MOL004444 Ziebeimine HSP90AA1

Zhebeimu ZB4 MOL004444 Ziebeimine ROCK2

Zhebeimu ZB4 MOL004444 Ziebeimine ADRA1D

Zhebeimu ZB4 MOL004444 Ziebeimine ADRA1A

Zhebeimu ZB4 MOL004444 Ziebeimine ADRA1B

Zhebeimu ZB4 MOL004444 Ziebeimine MELK

Zhebeimu ZB4 MOL004444 Ziebeimine INSR

Zhebeimu ZB4 MOL004444 Ziebeimine PARP1

Zhebeimu ZB4 MOL004444 Ziebeimine ALK

Zhebeimu ZB4 MOL004444 Ziebeimine BDKRB1

Zhebeimu ZB4 MOL004444 Ziebeimine PRCP

Zhebeimu ZB4 MOL004444 Ziebeimine CDK9

Zhebeimu ZB4 MOL004444 Ziebeimine BAD

Zhebeimu ZB4 MOL004444 Ziebeimine ABCB1

Zhebeimu ZB4 MOL004444 Ziebeimine SHH

Zhebeimu ZB4 MOL004444 Ziebeimine HTR2C

Zhebeimu ZB4 MOL004444 Ziebeimine PIK3CA

Zhebeimu ZB4 MOL004444 Ziebeimine BCL2L1

Zhebeimu ZB4 MOL004444 Ziebeimine BCL2

Zhebeimu ZB5 MOL004450 Chaksine JAK3

Zhebeimu ZB5 MOL004450 Chaksine MAPK10

Zhebeimu ZB5 MOL004450 Chaksine SRC

Zhebeimu ZB5 MOL004450 Chaksine CCR5

Zhebeimu ZB5 MOL004450 Chaksine JAK1

Zhebeimu ZB5 MOL004450 Chaksine JAK2

Zhebeimu ZB5 MOL004450 Chaksine MAPK9

Zhebeimu ZB5 MOL004450 Chaksine HRH2

Zhebeimu ZB5 MOL004450 Chaksine OPRL1

Zhebeimu ZB5 MOL004450 Chaksine ADORA1

Zhebeimu ZB5 MOL004450 Chaksine TYK2

Zhebeimu ZB5 MOL004450 Chaksine DHFR

Zhebeimu ZB5 MOL004450 Chaksine DRD2

Zhebeimu ZB5 MOL004450 Chaksine HTR2C

Zhebeimu ZB5 MOL004450 Chaksine F11

Zhebeimu ZB5 MOL004450 Chaksine REN

Zhebeimu ZB5 MOL004450 Chaksine QPCTL

Zhebeimu ZB5 MOL004450 Chaksine PADI1

Zhebeimu ZB5 MOL004450 Chaksine PADI3

Zhebeimu ZB5 MOL004450 Chaksine CA2

Zhebeimu ZB5 MOL004450 Chaksine CA1

Zhebeimu ZB5 MOL004450 Chaksine SLC9A1

Zhebeimu ZB5 MOL004450 Chaksine MTOR

Zhebeimu ZB5 MOL004450 Chaksine PIK3CA

Zhebeimu ZB5 MOL004450 Chaksine MAPK1

Zhebeimu ZB5 MOL004450 Chaksine PADI4

Zhebeimu ZB5 MOL004450 Chaksine MAPK14

Zhebeimu ZB5 MOL004450 Chaksine MAP2K7

Zhebeimu ZB5 MOL004450 Chaksine F2R

Zhebeimu ZB5 MOL004450 Chaksine TGFBR1

Zhebeimu ZB5 MOL004450 Chaksine QRFPR

Zhebeimu ZB5 MOL004450 Chaksine SLC22A2

Zhebeimu ZB5 MOL004450 Chaksine SLC47A1

Zhebeimu ZB5 MOL004450 Chaksine PDE5A

Zhebeimu ZB5 MOL004450 Chaksine HDAC3

Zhebeimu ZB5 MOL004450 Chaksine HTR2B

Zhebeimu ZB5 MOL004450 Chaksine SERPINC1

Zhebeimu ZB5 MOL004450 Chaksine PRSS3

Zhebeimu ZB5 MOL004450 Chaksine EIF2AK4

Zhebeimu ZB5 MOL004450 Chaksine EIF2AK2

Zhebeimu ZB5 MOL004450 Chaksine EIF2AK1

Zhebeimu ZB5 MOL004450 Chaksine ACHE

Zhebeimu ZB5 MOL004450 Chaksine ATR

Zhebeimu ZB5 MOL004450 Chaksine HTR3A

Zhebeimu ZB5 MOL004450 Chaksine RET

Zhebeimu ZB5 MOL004450 Chaksine KDR

Zhebeimu ZB5 MOL004450 Chaksine NPY5R

Zhebeimu ZB5 MOL004450 Chaksine RIPK2

Zhebeimu ZB5 MOL004450 Chaksine ALOX5

Zhebeimu ZB5 MOL004450 Chaksine PLK1

Zhebeimu ZB5 MOL004450 Chaksine TMPRSS6

Zhebeimu B1 MOL000358 beta-sitosterol RELA

Zhebeimu B1 MOL000358 beta-sitosterol NR3C2

Zhebeimu B1 MOL000358 beta-sitosterol PGR

Zhebeimu B1 MOL000358 beta-sitosterol NCOA2

Zhebeimu B1 MOL000358 beta-sitosterol PTGS1

Zhebeimu B1 MOL000358 beta-sitosterol PTGS2

Zhebeimu B1 MOL000358 beta-sitosterol HSP90

Zhebeimu B1 MOL000358 beta-sitosterol PI3K

Zhebeimu B1 MOL000358 beta-sitosterol KCNH2

Zhebeimu B1 MOL000358 beta-sitosterol PRKACA

Zhebeimu B1 MOL000358 beta-sitosterol CHRM3

Zhebeimu B1 MOL000358 beta-sitosterol CHRM1

Zhebeimu B1 MOL000358 beta-sitosterol SCN5A

Zhebeimu B1 MOL000358 beta-sitosterol GABRA2

Zhebeimu B1 MOL000358 beta-sitosterol CHRM4

Zhebeimu B1 MOL000358 beta-sitosterol PDE3A

Zhebeimu B1 MOL000358 beta-sitosterol HTR2A

Zhebeimu B1 MOL000358 beta-sitosterol GABRA5

Zhebeimu B1 MOL000358 beta-sitosterol ADRA1A

Zhebeimu B1 MOL000358 beta-sitosterol GABRA3

Zhebeimu B1 MOL000358 beta-sitosterol CHRM2

Zhebeimu B1 MOL000358 beta-sitosterol ADRA1B

Zhebeimu B1 MOL000358 beta-sitosterol ADRB2

Zhebeimu B1 MOL000358 beta-sitosterol CHRNA2

Zhebeimu B1 MOL000358 beta-sitosterol SLC6A4

Zhebeimu B1 MOL000358 beta-sitosterol OPRM1

Zhebeimu B1 MOL000358 beta-sitosterol GABRA1

Zhebeimu B1 MOL000358 beta-sitosterol CHRNA7

Zhebeimu B1 MOL000358 beta-sitosterol BCL2

Zhebeimu B1 MOL000358 beta-sitosterol BAX

Zhebeimu B1 MOL000358 beta-sitosterol CASP9

Zhebeimu B1 MOL000358 beta-sitosterol JUN

Zhebeimu B1 MOL000358 beta-sitosterol CASP3

Zhebeimu B1 MOL000358 beta-sitosterol CASP8

Zhebeimu B1 MOL000358 beta-sitosterol PRKCA

Zhebeimu B1 MOL000358 beta-sitosterol TGFB1

Zhebeimu B1 MOL000358 beta-sitosterol PON1

Zhebeimu B1 MOL000358 beta-sitosterol MAP2

Lianqiao LQ1 MOL003290 (3R,4R)-3,4-bis[(3,4-dimethoxyphenyl)methyl]oxolan-2-one CHRM3

Lianqiao LQ1 MOL003290 (3R,4R)-3,4-bis[(3,4-dimethoxyphenyl)methyl]oxolan-2-one KCNH2

Lianqiao LQ1 MOL003290 (3R,4R)-3,4-bis[(3,4-dimethoxyphenyl)methyl]oxolan-2-one ESR1

Lianqiao LQ1 MOL003290 (3R,4R)-3,4-bis[(3,4-dimethoxyphenyl)methyl]oxolan-2-one SCN5A

Lianqiao LQ1 MOL003290 (3R,4R)-3,4-bis[(3,4-dimethoxyphenyl)methyl]oxolan-2-one PTGS2

Lianqiao LQ1 MOL003290 (3R,4R)-3,4-bis[(3,4-dimethoxyphenyl)methyl]oxolan-2-one F7

Lianqiao LQ1 MOL003290 (3R,4R)-3,4-bis[(3,4-dimethoxyphenyl)methyl]oxolan-2-one PDE3A

Lianqiao LQ1 MOL003290 (3R,4R)-3,4-bis[(3,4-dimethoxyphenyl)methyl]oxolan-2-one ADRA1B

Lianqiao LQ1 MOL003290 (3R,4R)-3,4-bis[(3,4-dimethoxyphenyl)methyl]oxolan-2-one PTPN1

Lianqiao LQ1 MOL003290 (3R,4R)-3,4-bis[(3,4-dimethoxyphenyl)methyl]oxolan-2-one SLC6A3

Lianqiao LQ1 MOL003290 (3R,4R)-3,4-bis[(3,4-dimethoxyphenyl)methyl]oxolan-2-one ADRB2

Lianqiao LQ1 MOL003290 (3R,4R)-3,4-bis[(3,4-dimethoxyphenyl)methyl]oxolan-2-one ADRA1D

Lianqiao LQ1 MOL003290 (3R,4R)-3,4-bis[(3,4-dimethoxyphenyl)methyl]oxolan-2-one HSP70

Lianqiao LQ1 MOL003290 (3R,4R)-3,4-bis[(3,4-dimethoxyphenyl)methyl]oxolan-2-one NCOA2

Lianqiao LQ2 MOL003295 (+)-pinoresinol monomethyl ether PTGS1

Lianqiao LQ2 MOL003295 (+)-pinoresinol monomethyl ether KCNH2

Lianqiao LQ2 MOL003295 (+)-pinoresinol monomethyl ether SCN5A

Lianqiao LQ2 MOL003295 (+)-pinoresinol monomethyl ether PTGS2

Lianqiao LQ2 MOL003295 (+)-pinoresinol monomethyl ether RXRA

Lianqiao LQ2 MOL003295 (+)-pinoresinol monomethyl ether PDE3A

Lianqiao LQ2 MOL003295 (+)-pinoresinol monomethyl ether ADRA1B

Lianqiao LQ2 MOL003295 (+)-pinoresinol monomethyl ether ADRB2

Lianqiao LQ2 MOL003295 (+)-pinoresinol monomethyl ether HSP90

Lianqiao LQ2 MOL003295 (+)-pinoresinol monomethyl ether RXRB

Lianqiao LQ2 MOL003295 (+)-pinoresinol monomethyl ether NCOA2

Lianqiao LQ2 MOL003295 (+)-pinoresinol monomethyl ether NCOA1

Lianqiao LQ3 MOL003306 ACon1_001697 PTGS1

Lianqiao LQ3 MOL003306 ACon1_001697 KCNH2

Lianqiao LQ3 MOL003306 ACon1_001697 SCN5A

Lianqiao LQ3 MOL003306 ACon1_001697 PTGS2

Lianqiao LQ3 MOL003306 ACon1_001697 ADRA1B

Lianqiao LQ3 MOL003306 ACon1_001697 ADRB2

Lianqiao LQ3 MOL003306 ACon1_001697 HSP90

Lianqiao LQ3 MOL003306 ACon1_001697 PRKACA

Lianqiao LQ3 MOL003306 ACon1_001697 NCOA2

Lianqiao LQ3 MOL003306 ACon1_001697 NCOA1

Lianqiao LQ4 MOL003308 (+)-pinoresinol monomethyl ether-4-D-beta-glucoside_qt KCNH2

Lianqiao LQ4 MOL003308 (+)-pinoresinol monomethyl ether-4-D-beta-glucoside_qt SCN5A

Lianqiao LQ4 MOL003308 (+)-pinoresinol monomethyl ether-4-D-beta-glucoside_qt PTGS2

Lianqiao LQ4 MOL003308 (+)-pinoresinol monomethyl ether-4-D-beta-glucoside_qt ADRB2

Lianqiao LQ4 MOL003308 (+)-pinoresinol monomethyl ether-4-D-beta-glucoside_qt HSP90

Lianqiao LQ4 MOL003308 (+)-pinoresinol monomethyl ether-4-D-beta-glucoside_qt PRKACA

Lianqiao LQ4 MOL003308 (+)-pinoresinol monomethyl ether-4-D-beta-glucoside_qt NCOA2

Lianqiao LQ4 MOL003308 (+)-pinoresinol monomethyl ether-4-D-beta-glucoside_qt NCOA1

Lianqiao LQ5 MOL000211 Mairin PGR

Lianqiao LQ6 MOL003322 FORSYTHINOL KCNH2

Lianqiao LQ6 MOL003322 FORSYTHINOL SCN5A

Lianqiao LQ6 MOL003322 FORSYTHINOL PTGS2

Lianqiao LQ6 MOL003322 FORSYTHINOL ADRA1B

Lianqiao LQ6 MOL003322 FORSYTHINOL ADRB2

Lianqiao LQ6 MOL003322 FORSYTHINOL HSP90

Lianqiao LQ6 MOL003322 FORSYTHINOL NCOA2

Lianqiao LQ6 MOL003322 FORSYTHINOL NCOA1

Lianqiao LQ7 MOL003330 (-)-Phillygenin CHRM3

Lianqiao LQ7 MOL003330 (-)-Phillygenin KCNH2

Lianqiao LQ7 MOL003330 (-)-Phillygenin CHRM1

Lianqiao LQ7 MOL003330 (-)-Phillygenin SCN5A

Lianqiao LQ7 MOL003330 (-)-Phillygenin CHRM5

Lianqiao LQ7 MOL003330 (-)-Phillygenin PTGS2

Lianqiao LQ7 MOL003330 (-)-Phillygenin ADRA1B

Lianqiao LQ7 MOL003330 (-)-Phillygenin ADRB2

Lianqiao LQ7 MOL003330 (-)-Phillygenin HSP90

Lianqiao LQ7 MOL003330 (-)-Phillygenin IGHG1

Lianqiao LQ7 MOL003330 (-)-Phillygenin NCOA2

Lianqiao LQ8 MOL003347 hyperforin CYP3A4

Lianqiao LQ8 MOL003347 hyperforin ICAM1

Lianqiao LQ8 MOL003347 hyperforin CXCL8

Lianqiao LQ8 MOL003347 hyperforin NR1I2

Lianqiao LQ9 MOL003370 Onjixanthone I NOS2

Lianqiao LQ9 MOL003370 Onjixanthone I PTGS1

Lianqiao LQ9 MOL003370 Onjixanthone I SCN5A

Lianqiao LQ9 MOL003370 Onjixanthone I PTGS2

Lianqiao LQ9 MOL003370 Onjixanthone I RXRA

Lianqiao LQ9 MOL003370 Onjixanthone I ESR2

Lianqiao LQ9 MOL003370 Onjixanthone I DPP4

Lianqiao LQ9 MOL003370 Onjixanthone I HSP90

Lianqiao LQ9 MOL003370 Onjixanthone I PIK3CG

Lianqiao LQ9 MOL003370 Onjixanthone I CHEK1

Lianqiao LQ9 MOL003370 Onjixanthone I KCNMA1

Lianqiao B1 MOL000358 beta-sitosterol PGR

Lianqiao B1 MOL000358 beta-sitosterol NCOA2

Lianqiao B1 MOL000358 beta-sitosterol PTGS1

Lianqiao B1 MOL000358 beta-sitosterol PTGS2

Lianqiao B1 MOL000358 beta-sitosterol HSP90

Lianqiao B1 MOL000358 beta-sitosterol PIK3CG

Lianqiao B1 MOL000358 beta-sitosterol KCNH2

Lianqiao B1 MOL000358 beta-sitosterol PRKACA

Lianqiao B1 MOL000358 beta-sitosterol CHRM3

Lianqiao B1 MOL000358 beta-sitosterol CHRM1

Lianqiao B1 MOL000358 beta-sitosterol SCN5A

Lianqiao B1 MOL000358 beta-sitosterol GABRA2

Lianqiao B1 MOL000358 beta-sitosterol CHRM4

Lianqiao B1 MOL000358 beta-sitosterol PDE3A

Lianqiao B1 MOL000358 beta-sitosterol HTR2A

Lianqiao B1 MOL000358 beta-sitosterol GABRA5

Lianqiao B1 MOL000358 beta-sitosterol ADRA1A

Lianqiao B1 MOL000358 beta-sitosterol GABRA3

Lianqiao B1 MOL000358 beta-sitosterol CHRM2

Lianqiao B1 MOL000358 beta-sitosterol ADRA1B

Lianqiao B1 MOL000358 beta-sitosterol ADRB2

Lianqiao B1 MOL000358 beta-sitosterol CHRNA2

Lianqiao B1 MOL000358 beta-sitosterol SLC6A4

Lianqiao B1 MOL000358 beta-sitosterol OPRM1

Lianqiao B1 MOL000358 beta-sitosterol GABRA1

Lianqiao B1 MOL000358 beta-sitosterol CHRNA7

Lianqiao B1 MOL000358 beta-sitosterol BCL2

Lianqiao B1 MOL000358 beta-sitosterol BAX

Lianqiao B1 MOL000358 beta-sitosterol CASP9

Lianqiao B1 MOL000358 beta-sitosterol JUN

Lianqiao B1 MOL000358 beta-sitosterol CASP3

Lianqiao B1 MOL000358 beta-sitosterol CASP8

Lianqiao B1 MOL000358 beta-sitosterol PRKCA

Lianqiao B1 MOL000358 beta-sitosterol TGFB1

Lianqiao B1 MOL000358 beta-sitosterol PON1

Lianqiao B1 MOL000358 beta-sitosterol MAP2

Lianqiao LQ10 MOL000522 arctiin KCNH2

Lianqiao LQ10 MOL000522 arctiin SCN5A

Lianqiao LQ10 MOL000522 arctiin PTGS2

Lianqiao LQ10 MOL000522 arctiin KDR

Lianqiao LQ10 MOL000522 arctiin PTPN1

Lianqiao LQ10 MOL000522 arctiin ADRB2

Lianqiao LQ10 MOL000522 arctiin HSP90

Lianqiao LQ10 MOL000522 arctiin NCOA1

Lianqiao LQ10 MOL000522 arctiin MUC1

Lianqiao LQ11 MOL000006 luteolin PTGS1

Lianqiao LQ11 MOL000006 luteolin AR

Lianqiao LQ11 MOL000006 luteolin PTGS2

Lianqiao LQ11 MOL000006 luteolin HSP90

Lianqiao LQ11 MOL000006 luteolin PRSS1

Lianqiao LQ11 MOL000006 luteolin NCOA2

Lianqiao LQ11 MOL000006 luteolin PRKACA

Lianqiao LQ11 MOL000006 luteolin DPP4

Lianqiao LQ11 MOL000006 luteolin PIK3CG

Lianqiao LQ11 MOL000006 luteolin RELA

Lianqiao LQ11 MOL000006 luteolin EGFR

Lianqiao LQ11 MOL000006 luteolin AKT1

Lianqiao LQ11 MOL000006 luteolin VEGFA

Lianqiao LQ11 MOL000006 luteolin CCND1

Lianqiao LQ11 MOL000006 luteolin BCL2L1

Lianqiao LQ11 MOL000006 luteolin CDKN1A

Lianqiao LQ11 MOL000006 luteolin CASP9

Lianqiao LQ11 MOL000006 luteolin MMP2

Lianqiao LQ11 MOL000006 luteolin MMP9

Lianqiao LQ11 MOL000006 luteolin MAPK1

Lianqiao LQ11 MOL000006 luteolin IL10

Lianqiao LQ11 MOL000006 luteolin RB1

Lianqiao LQ11 MOL000006 luteolin CDK4

Lianqiao LQ11 MOL000006 luteolin TNF

Lianqiao LQ11 MOL000006 luteolin JUN

Lianqiao LQ11 MOL000006 luteolin IL6

Lianqiao LQ11 MOL000006 luteolin CASP3

Lianqiao LQ11 MOL000006 luteolin TP53

Lianqiao LQ11 MOL000006 luteolin NFKBIA

Lianqiao LQ11 MOL000006 luteolin XDH

Lianqiao LQ11 MOL000006 luteolin TOP1

Lianqiao LQ11 MOL000006 luteolin MDM2

Lianqiao LQ11 MOL000006 luteolin MMP1

Lianqiao LQ11 MOL000006 luteolin PCNA

Lianqiao LQ11 MOL000006 luteolin ERBB2

Lianqiao LQ11 MOL000006 luteolin PPARG

Lianqiao LQ11 MOL000006 luteolin HMOX1

Lianqiao LQ11 MOL000006 luteolin CASP7

Lianqiao LQ11 MOL000006 luteolin ICAM1

Lianqiao LQ11 MOL000006 luteolin MCL1

Lianqiao LQ11 MOL000006 luteolin BIRC5

Lianqiao LQ11 MOL000006 luteolin IL2

Lianqiao LQ11 MOL000006 luteolin CCNB1

Lianqiao LQ11 MOL000006 luteolin TYR

Lianqiao LQ11 MOL000006 luteolin IFNG

Lianqiao LQ11 MOL000006 luteolin IL4

Lianqiao LQ11 MOL000006 luteolin TOP2A

Lianqiao LQ11 MOL000006 luteolin GSTP1

Lianqiao LQ11 MOL000006 luteolin SLC2A4

Lianqiao LQ11 MOL000006 luteolin INSR

Lianqiao LQ11 MOL000006 luteolin CD40LG

Lianqiao LQ11 MOL000006 luteolin PTGES

Lianqiao LQ11 MOL000006 luteolin NUF2

Lianqiao LQ11 MOL000006 luteolin ADCY2

Lianqiao LQ11 MOL000006 luteolin MET

Lianqiao LQ12 MOL000791 bicuculline PTGS1

Lianqiao LQ12 MOL000791 bicuculline KCNH2

Lianqiao LQ12 MOL000791 bicuculline AR

Lianqiao LQ12 MOL000791 bicuculline SCN5A

Lianqiao LQ12 MOL000791 bicuculline PTGS2

Lianqiao LQ12 MOL000791 bicuculline KDR

Lianqiao LQ12 MOL000791 bicuculline ACHE

Lianqiao LQ12 MOL000791 bicuculline HSP90

Lianqiao LQ12 MOL000791 bicuculline PTPN1

Lianqiao LQ12 MOL000791 bicuculline PRKACA

Lianqiao LQ12 MOL000791 bicuculline FOS

Lianqiao LQ12 MOL000791 bicuculline GJA1

Lianqiao LQ12 MOL000791 bicuculline GABBR1

Lianqiao LQ12 MOL000791 bicuculline BMPR2

Lianqiao LQ12 MOL000791 bicuculline GRM5

Lianqiao LQ12 MOL000791 bicuculline GNRH1

Lianqiao LQ12 MOL000791 bicuculline ALDH3A1

Lianqiao LQ12 MOL000791 bicuculline GNRHR

Lianqiao LQ12 MOL000791 bicuculline CRH

Lianqiao LQ12 MOL000791 bicuculline GRIN2D

Lianqiao LQ12 MOL000791 bicuculline SLC6A2

Lianqiao LQ12 MOL000791 bicuculline GJB1

Lianqiao LQ12 MOL000791 bicuculline GRM1

Lianqiao LQ12 MOL000791 bicuculline VCP

Lianqiao D1 MOL000098 quercetin PTGS1

Lianqiao D1 MOL000098 quercetin AR

Lianqiao D1 MOL000098 quercetin PPARG

Lianqiao D1 MOL000098 quercetin PTGS2

Lianqiao D1 MOL000098 quercetin HSP90

Lianqiao D1 MOL000098 quercetin PIK3CG

Lianqiao D1 MOL000098 quercetin NCOA2

Lianqiao D1 MOL000098 quercetin DPP4

Lianqiao D1 MOL000098 quercetin PRSS1

Lianqiao D1 MOL000098 quercetin KCNH2

Lianqiao D1 MOL000098 quercetin SCN5A

Lianqiao D1 MOL000098 quercetin ADRB2

Lianqiao D1 MOL000098 quercetin MMP3

Lianqiao D1 MOL000098 quercetin PRKACA

Lianqiao D1 MOL000098 quercetin F7

Lianqiao D1 MOL000098 quercetin NOS2

Lianqiao D1 MOL000098 quercetin RXRA

Lianqiao D1 MOL000098 quercetin ACHE

Lianqiao D1 MOL000098 quercetin GABRA1

Lianqiao D1 MOL000098 quercetin MAOB

Lianqiao D1 MOL000098 quercetin RELA

Lianqiao D1 MOL000098 quercetin EGFR

Lianqiao D1 MOL000098 quercetin AKT1

Lianqiao D1 MOL000098 quercetin VEGFA

Lianqiao D1 MOL000098 quercetin CCND1

Lianqiao D1 MOL000098 quercetin BCL2

Lianqiao D1 MOL000098 quercetin BCL2L1

Lianqiao D1 MOL000098 quercetin FOS

Lianqiao D1 MOL000098 quercetin CDKN1A

Lianqiao D1 MOL000098 quercetin EIF6

Lianqiao D1 MOL000098 quercetin BAX

Lianqiao D1 MOL000098 quercetin CASP9

Lianqiao D1 MOL000098 quercetin PLAU

Lianqiao D1 MOL000098 quercetin MMP2

Lianqiao D1 MOL000098 quercetin MMP9

Lianqiao D1 MOL000098 quercetin MAPK1

Lianqiao D1 MOL000098 quercetin IL10

Lianqiao D1 MOL000098 quercetin EGF

Lianqiao D1 MOL000098 quercetin RB1

Lianqiao D1 MOL000098 quercetin TNF

Lianqiao D1 MOL000098 quercetin JUN

Lianqiao D1 MOL000098 quercetin IL6

Lianqiao D1 MOL000098 quercetin CDKN2A

Lianqiao D1 MOL000098 quercetin AHSA1

Lianqiao D1 MOL000098 quercetin CASP3

Lianqiao D1 MOL000098 quercetin TP53

Lianqiao D1 MOL000098 quercetin ELK1

Lianqiao D1 MOL000098 quercetin NFKBIA

Lianqiao D1 MOL000098 quercetin POR

Lianqiao D1 MOL000098 quercetin ODC1

Lianqiao D1 MOL000098 quercetin XDH

Lianqiao D1 MOL000098 quercetin CASP8

Lianqiao D1 MOL000098 quercetin TOP1

Lianqiao D1 MOL000098 quercetin RAF1

Lianqiao D1 MOL000098 quercetin SOD1

Lianqiao D1 MOL000098 quercetin PRKCA

Lianqiao D1 MOL000098 quercetin MMP1

Lianqiao D1 MOL000098 quercetin HIF1A

Lianqiao D1 MOL000098 quercetin STAT1

Lianqiao D1 MOL000098 quercetin RUNX1T1
