# Supplementary material for: Study on the Anti-demyelination Mechanism of Bu-Shen-Yi-Sui Capsule in the Central Nervous System Based on Network Pharmacology and Experimental Verification
Source: Mediators Inflamm. 2022 Jul 12;2022:9241261. doi: 10.1155/2022/9241261 (PMC9296285; doi:10.1155/2022/9241261)
Supplement: Supplementary Materials — Table S1: all the potential targets of BSYS Capsule. Table S2: known CNSD-related targets. Table S3: BSYS Capsule shared 227 intersection targets with known CNSD-related targets. Table S4: PPI information of 227 intersection targets in Metascape. Table S5: the degree values of all nodes in the PPI network. Table S6: results for GO pathway enrichment analysis. Table S7: results for KEGG pathway enrichment analysis. Table S8: information of gene-pathway network. Table S9: information of the “active ingredients-intersection targets” network. [file 9241261.f1.zip › Table S2.docx]

Gene Symbol

MPZ

SOX10

ASPA

NEFL

RYR1

SMARCB1

HLA-DRB1

PMP22

IL6

EGR2

BDNF

SPATA22

PRX

RET

TP53

GDNF

GFAP

IL10

GDAP1

PLP1

GJB1

SHH

TNF

MBP

TREX1

POLR2F

MAPT

ARSA

HLA-DQB1

POLG

TRPV4

ACE

SURF1

CTLA4

ATRIP

IFNG

SH3TC2

EDN3

ASCL1

CHAT

NF1

TTR

PDCD1

KRAS

PRNP

PSAP

HFE

CCR6

MT-ATP6

MECP2

SNCA

NF2

LMNA

MFN2

MAG

AKT1

FIG4

LMNB1

CCL2

POLR1C

NGF

EIF2B5

FGFR1

APOE

MYC

IL1B

DNM2

ABCD1

SOX2

EIF2B2

SBF2

MTMR2

ATRIP-TREX1

TH

LITAF

PTPN22

ALB

FGD4

CPT2

NDRG1

ITGAM

MT-ND1

PAH

EIF2B4

COQ2

PMP2

TLR4

MYCN

GBA

HARS1

ERBB2

AARS2

LRRK2

ERCC6

NTRK2

CD4

NFASC

EIF2B3

MIR21

PEX6

EIF2B1

VEGFA

EGFR

MT-ND6

GALC

IARS2

IRF5

HLA-B

MOG

INS

NTRK1

IL17A

SPP1

SMARCA4

MIR17

IL2

PRKN

SCN2A

MTOR

ICAM1

MT-ND4

GJC2

MFSD8

PAX6

MMP9

FAS

MT-ND5

SOD1

VCP

FCGR2B

MTHFR

PMPCA

IL4

BRAF

CD40LG

MT-TK

MT-TL1

ATM

TNFRSF1A

MME

ARSB

TBK1

NOS2

MYO1H

SERPINA3

CRP

H2AC18

IDH1

CXCL8

KIF1B

SPG7

PRF1

FGFR3

FGF2

CNTF

SYP

WFS1

IL7R

AVP

IGF1

GARS1

NPY

SLC6A3

ENO2

APP

B2M

SMPD1

NPC1

C3

AQP4

SCN9A

PRPS1

MT-CYB

TSC1

TGIF1

NES

GLB1

EGF

BRCA1

MTM1

S100B

STAT3

PSEN1

BCL2

SDHA

SCN1A

CXCL10

ANKLE2

LEP

CACNA1A

SOX9

EDNRB

SLC6A4

PTPRC

APOA1

PLA2G6

REN

MT-CO3

CR2

MYD88

GAL

SDHB

FOXP3

MT-ND2

FCGR2A

CASP3

MT-ND3

CCR1

FASLG

TGFB1

NDUFAF2

CCL5

GRIN2B

MIR155

PAX3

CD34

CLCN1

MDM2

DBH

ACTB

ICOSLG

KARS1

F2

POLR3A

DNASE1

GJB2

MAPK1

CDC42

POLR3B

TPP1

IFNB1

GRN

HLA-DQA1

MYO7A

ESR1

CCR5

CD8A

SDHD

CALCA

CFH

IFNA1

HTT

MIR125A

CNP

PPARG

BSCL2

NDUFS4

TLR3

MPO

RAB7A

NLRP3

IL13

TNFAIP3

MIR142

HLA-A

ERCC4

STAT1

CD44

DYNC1H1

NPC2

MIR146A

CSF1R

HMOX1

SPTLC1

CNR1

ATXN2

SLC25A12

MEFV

IDH2

CTSD

AGTR1

HLA-DPB1

FCGR3B

SBF1

MIR143

DLD

ABCA4

NDUFV1

SPTLC2

IFIH1

TNFSF13B

AARS1

MIR126

CD55

VIP

SCN10A

MT-CO1

MYRF

EP300

TNFSF4

FOS

CP

CD79A

MIR145

NTF3

CD19

COMT

IGF2

SCO2

BLK

FOXH1

CDK5

PDGFRA

TYMP

ADAR

RTN4

MIF

SCN11A

TLR2

CXCR4

PHGDH

NAGLU

NEFH

FBN1

NOTCH3

C9orf72

PNPLA6

CRYAA

ADA2

PDHA1

CD40

TERT

TWNK

NGFR

TBP

JUN

HTR2A

FBLN5

SGSH

AFG3L2

AQP1

GAN

KDM4C

AAAS

KIF1A

HGSNAT

MT-TV

MT-TW

ABCG2

EDN1

CXCR3

HCRT

CXCL12

TREM2

NOD2

MIR9-1

CD274

BCS1L

LAMA2

F5

MMACHC

ACHE

ERCC2

RELN

FXN

KIF5A

GLA

CDKN1A

MIR223

ACOX1

CNTNAP1

MMP2

OLIG2

PDHB

ELP1

KCNJ10

SERPINE1

CASP8

IL5

CD46

HSPB1

PRODH

RHO

DMD

IL2RA

EPRS1

ERCC8

GRM1

IKBKG

ADA

SLC1A3

SAMHD1

VCAM1

PTGS2

CD59

DCTN1

VIM

LIFR

CD28

CLN8

CLN3

TGFBR2

MGMT

SMC1A

ASAH1

GJA1

CSF2

FMR1

HSPB8

SERPINI1

CXCL13

NCAM1

U2AF1

ITGA4

ERBB4

FN1

CAV1

ATXN3

FGA

DRD2

HTR1A

DNASE1L3

FOXG1

ABCB1

TF

RNASEH2B

HK1

TDGF1

CLCN6

HSD17B4

ERCC1

PRTN3

MAPK10

HSP90AA1

DNMT1

MTR

CDH23

TARDBP

MIR132

DAG1

TAC1

CR1

CYCS

DDC

SLC1A2

GNB1

CLN5

IDUA

MIR96

DNAJC6

HIF1A

MBL2

SCARB2

CTDP1

GAPDH

FUS

OPA1

AGRN

HPRT1

GLUL

PANK2

IL18

FASTKD2

IGHMBP2

CFI

DNM1L

KNG1

ZFYVE26

PECAM1

IRF4

STXBP1

SACS

BANK1

TBC1D24

NDUFAF6

GSN

PDYN

AIF1

FOXRED1

GRIA3

ECE1

ARHGEF10

AR

CRYAB

NDUFA13

PLAT

MIR326

NR1H3

COL4A1

RNASEH2A

APOB

POU3F2

CCL3

NRG1

SDHC

FLVCR1

TNFRSF1B

MAPK3

ITGB2

TFRC

MIR338

MT-TS1

MARS1

MTTP

PMM2

NOTCH1

CYP3A4

SPTAN1

IL1RN

MUSK

CBS

TRPV1

DEGS1

CFB

MTFMT

ELN

SYK

ABCA1

SPTBN4

MIR20A

CREB1

IL23R

ATXN8OS

RAD51

MT-CO2

AGTPBP1

SPG11

CX3CR1

NOS1

IL17F

MIR27A

GNS

ZSWIM6

SLC25A46

MRPS34

CD27

SLC18A3

ABHD12

CLN6

MT-TE

NTRK3

TACR1

BCL2L1

MPV17

FANCI

HSPD1

NR3C1

MAP2

RNASEH2C

VDR

PNKP

MIR124-1

GBE1

HTRA1

FA2H

GNAS

CNTNAP2

PEX1

DNAJC3

GRIA2

C1R

PPARGC1A

JAG1

PPT1

SUMF1

CALR

LRSAM1

SCN8A

RTN4R

FANCB

DNM1

SETX

ABCB6

CD86

INSR

IL2RB

MERTK

ATP7A

ATP7B

ATL1

SEMA4A

TUBB3

RELA

PNP

SLC46A1

CD80

CDH2

SYNE1

ITGB4

SYNJ1

AIFM1

ABL1

GCDH

PLEC

RRM2B

RAI1

MIR181A1

NDUFS2

ERCC5

WDR45

UCHL1

GNPTAB

EDAR

CAT

NFKB1

IDS

SNAP29

CASP9

IL12B

HLA-DPA1

PINK1

SLC25A4

SLC5A7

LTF

MITF

XIAP

TNFRSF13C

IRF1

TRAPPC11

VPS11

SEMA7A

MAT1A

MIR24-1

CSF3

SMAD4

ATXN7

NOG

NEU1

MS3

RBFOX3

CXCR5

LGI1

ADSL

ADAMTS13

PARK7

MORC2

ACKR1

ESR2

DNAJB2

PTGDS

BIN1

ATP1A3

GNA11

OCRL

TBX21

MIR149

IL1A

COX6A1

STX11

SLC25A13

YARS1

SBF2-AS1

FLNC

DICER1

PLAU

LIF

KCNA1

ZMPSTE24

IGF1R

ERCC3

GALNS

IL7

APTX

CACNA1C

AIRE

BAX

CNTN1

DCAF8

OPRM1

UGT8

FOXO1

LTA

XPA

ACP5

GAD1

GPT

HP

DNAJC5

SMN1

ATXN1

CISH

PON1

CYP27A1

LOC101928008

WNK1

GNRH1

GSR

CDKN1C

IL12A

MT-TS2

TIMP1

EMD

CD36

POU5F1

CCL11

RERE

RAB9B

DNAH8

CCL4

MLH3

KIF1C

CASP1

FGF14

INF2

HSPA4

MIR22

SLC12A2

STIM1

CLDN11

STXBP2

PYCR2

APBB1

ERBB3

SMN2

NOTCH2

CD68

TBX1

MED25

CDK6

PEX5

ATXN10

TYROBP

SNAP25

TSPO

TGFBR1

ALAD

BTD

ANXA5

NARS1

SUCLA2

GMPPB

TYR

MLC1

IL15

PEX2

MS2

MS4

TPRKB

DMPK

ANK3

GPHN

SOCS1

HNRNPA1

FTH1

KDR

SMAD3

CYBB

MIR92A1

SPG21

KMT2A

MRE11

ITGA2

MAPK8

ACE2

PURA

SLC17A5

HRH3

SAG

SEMA3A

MPL

DMXL2

LYST

GRIN1

IL4R

FGF13

MT-TF

CIITA

XRCC4

EZR

GCK

SOX8

YWHAE

SYNE2

SELE

ADIPOQ

PEX16

NRP1

SLC16A2

RETREG1

EIF2AK2

RAB3GAP1

EPO

PTX3

STAT6

NDUFAF3

IGHM

TRIM2

IRF3

COX10

KCNK3

POU3F1

SLC12A6

MIR98

TNFSF10

MOCS2

CST3

HLA-DRA

USP7

ADGRG1

S1PR1

CXCL11

ISG15

MMP1

ATP13A2

ALDH3A2

MANBA

GFRA1

PEX10

IL1R1

ELOVL4

HNRNPUL2-BSCL2

HLA-DRB5

AVPR2

VPS13A

CXCR2

ARF1

C5

MDH2

CCR4

ATG5

COQ4

XDH

SLC1A1

LRPPRC

PRKAR1A

HEPACAM

L2HGDH

MTMR14

FCGR3A

GLS

GRIA1

GMPPA

SCN3A

EIF4G1

TLR9

FABP7

MT-TH

FGB

CNBP

TCF3

NALCN

CCR2

CCR7

CISD2

CNTN2

RAG1

DGUOK

CHCHD10

KCNJ11

CD1A

RPS27A

GC

GNB4

MYOC

ISL1

MYT1

CSF1

FAM126A

MIR455

TPI1

POLG2

NKX2-2

PRDM1

POU3F4

PSEN2

SLC19A1

CD1D

ADCY10

TMEM106B

GJB6

ACTG1

CHRM3

PEX19

DARS2

YARS2

CLU

NIPBL

VAC14

NAB2

GPX4

LMNB2

OMG

CACNA1B

MT-TQ

PRKCA

MAPK14

TUBB4A

NDUFAF4

SUCLG1

GATA2

XK

NARS2

SOD2

PIK3CG

CHRNA7

DHFR

QDPR

HAVCR2

ALS2

ATN1

WASHC5

CSTB

ATP1A1

FOLH1

UBQLN2

MVK

HCFC1

COX7B

PPARA

HMGB1

KCNK9

POU4F1

MAN2B1

BTNL2

DRP2

GLUD1

GABBR1

RAD21

CHM

ALDH5A1

TNC

EOMES

SCN4A

MIR184

TUBB

KCNA2

GM2A

SCN1A-AS1

PGR

DHTKD1

MADD

SPAST

TNFRSF13B

JAK1

NFE2L2

SIRT1

LOX

LINGO1

TCIRG1

MMP3

IL3

IL23A

CSPG4

KCNJ5

AMPH

HDAC6

ATP5F1A

DST

C11orf65

SIL1

LGALS3

PARP1

MIR186

MT-TN

HNRNPA2B1

PTPRZ1

HSPA8

EXOSC8

IL12RB1

DHH

TERC

RAB11A

CCT5

CACNA1G

IFNA2

PRPH

OTOF

NDUFV2

OPA3

LAMP2

CYP19A1

PDK3

EXOSC3

POLR2A

SLC19A3

GRIA4

ITGAL

JPH1

RFC1

MTRFR

ALG3

CD5

IRF7

SNAI2

ADCYAP1

PEX12

SLC12A5

AGER

ACER3

TFG

SMC3

CXCL9

OPTN

PLEKHG5

PCSK9

SUOX

OSTM1

NAGA

SMARCA2

GSTP1

PET100

PRDX1

RAB27A

PIGG

GBA2

ITPR1

LRP2

NDUFA1

TLR7

ADAM17

NEDD4

MAL

RDX

ETFDH

IFNGR1

SETD5

TJP1

CCR3

SELL

KCNMA1

MT-TT

DGKE

HLA-G

MFSD2A

CRAT

IL33

ACO2

SDHAF2

IRF8

MOCS1

ADM

TBL1X

NDUFB11

BAG3

MT-TI

TACO1

NDUFS1

CTSF

CYP11A1

ABCC8

ACAD9

DDB2

TRPM7

MOBP

TIMM8A

BMP6

MT-ATP8

CLEC7A

LCN2

C5AR1

PTCD3

FUCA1

PTS

MYH14

HADHA

GTF2I

MCM3AP

STAC3

KRT14

OCLN

SPTBN2

CA2

RORA

NDUFA12

AQP2

MT-ND4L

SOCS3

NDUFS3

LAMB1

AMACR

CACNA1H

SPART

MX1

LARS2

TDP1

WRN

DCT

HEXA

ATAD1

PEX14

DNAAF2

SERPING1

INA

BLOC1S1

IFNAR1

MB

ANPEP

DKK1

ATL3

WIPF1

PRICKLE1

WAS

PPP1R15B

RAB7B

SLC17A6

THY1

MSN

EXT1

CCL7

HMGCR

PDHX

PIKFYVE

SCP2

PCCB

RARS1

AKR1B1

AIMP2

ODC1

MYF6

UNC119

XPC

ARHGEF6

SLC7A11

CXCL1

TSEN2

KRT5

RAB28

CANX

LOC108663987

PNOC

PEX26

GRIP1

MIR219A1

MSI1

FAAH

XRCC1

LGI4

NDUFS8

COX4I1

PC

KLK6

MT-TP

COQ8A

NDUFA2

DAB1

COA3

ALG12

COX6B1

RNASEL

ACY1

DCAF17

ICOS

SLC5A6

SCO1

CD69

PPP2R2B

PEX7

STUB1

DNAAF11

ARRB1

SLC25A22

TNFRSF10A

TNXB

TBCD

ANO10

MIR491

MMUT

MYO9A

FBXL4

OTC

RNASET2

HEXB

PIK3C2A

TMEM43

MCOLN1

HCCS

POLD1

NDUFS7

NDUFAF1

CAPN1

XRCC5

KCNT1

CA10

TDP2

MTRR

HADHB

RHOA

PEX13

BCHE

IMMT

NDUFB8

IDO1

KCNB1

MMP7

DDX58

C19orf12

ATF4

STING1

ACP1

ELP4

TNFRSF17

HIP1

TRPA1

NDUFS6

USP18

SP1

LOC102724058

BIRC5

ANG

NUBPL

PRKG1

SUMO1

NDUFA6

NDUFB9

TNPO3

SLC25A1

KLF4

HDAC8

PLD3

S100A1

HTR2C

PDZD7

PDE4A

EHHADH

ALDH18A1

NDUFB3

NDUFAF5

LPL

CYP11B1

FGF9

TAP2

COQ6

NGLY1

TRIM32

PFN1

TFAM

PEX3

GLE1

GALR2

TMEM70

PDP1

TIMMDC1

CYFIP2

PAK3

SGPL1

CLIP2

UGCG

EXT2

CYP27B1

MFF

PTRH2

CD209

UNC93B1

TMEM126B

CHIT1

ETFA

TNR

MYH6

IL16

CSGALNACT1

TOP1

NKX6-2

ERVW-1

MT-TL2

RNF168

VAMP1

EIF2S3

UCP2

CA8

IL9

IGFBP2

PTN

TTPA

M6PR

DPYSL5

IBA57

ARHGEF2

ADAT3

DLG1

PPOX

STS

NAT8L

SEPTIN9

ADARB1

RPL5

LAMP1

KL

CDK1

ATP5F1E

CNR2

WARS1

CDKN3

VTN

F13A1

NIPA1

BGLAP

KRT1

ITGA6

BIRC3

EIF2AK1

MFN1

TNFSF12

UBTF

KCNC3

BCAP31

EIF2AK4

CUL4B

DNA2

ALOX5

CTSB

SKIV2L

KIF20A

GNB3

ITIH4

FCRL3

GJB3

GAS6

MYOT

GJA8

TAT

C3AR1

COASY

BICD2

GALE

IL1RAPL2

PADI4

GAMT

TYK2

HIBCH

GRID2

SYT2

TSEN34

NOP56

EIF2AK3

COMP

GFM2

RAB18

EGR1

CYP7B1

SHC1

GTPBP3

CDH5

ELOVL1

TK2

TRMU

ENTPD1

LCAT

TXN2

CHD4

IL10RA

COL6A3

TPPP

EEA1

APOA2

CYP2U1

RNASEH1

HSPA5

DYSF

PDX1

PLOD1

CALB1

ANOS1

IGFBP1

CCR8

ABCD2

BAK1

PPP1R15A

NAXE

CPOX

NPHP3-ACAD11

NDUFA11

TMEM126A

ABCG5

STMN1

HMBS

POLR3H

MICB

PTDSS1

NR1H2

ETHE1

SMAD2

AXL

PRKCG

TRAF3IP2

HDAC1

SLC35A2

GATAD2B

CD14

PPP6R2

COX15

IER3IP1

FGF1

COG8

NR1H4

REEP1

UBA5

CD2

CLCN2

SLC25A24

PRKCB

MCL1

IQSEC2

TAP1

PLA2G2A

GCLC

COX5A

PNPT1

NEMF

DDHD2

HNF4A

OPALIN

DGCR2

SLC11A1

VPS13D

DAAM2

BUD23

SERAC1

IDH3A

LOC110806262

TIA1

PLEK

RIT1

ABCC2

B4GALNT1

SCNN1B

SLC25A19

PIGY

UQCRC2

SLC12A1

UBE3B

NDUFA9

LDB3

IL21

VCL

FLII

IFI16

CD24

SZT2

MRPS16

MT-TA

PIEZO2

JPH3

FDFT1

DEAF1

AXIN2

SLC13A3

AQP5

IL18R1

CXCL2

BRD4

ID2

TNFRSF12A

COQ9

FAH

CFLAR

LIPA

COA8

CCDC115

KAT5

CD1E

CLP1

FAM83H

SCARB1

SAR1B

GJC3

BLVRB

TGM2

TRIP4

NDUFAF8

MRPS22

INPP5K

RARRES2

MICU1

PDGFA

RNF135

KATNB1

TALDO1

APOA1-AS

LIPT1

MST1

HELLS

CFL2

PDE4D

IFNAR2

CLDN14

CTSL

COQ7

NDUFA10

P4HB

APEX1

UBE4B

BRCC3

TTC19

NPAP1

BIVM-ERCC5

EIF2S1

SLC52A3

TARS1

CCL21

ERLIN2

NT5E

HBEGF

TYRO3

STAT2

ECHS1

CCNH

CD163

SORT1

LOC100287944

CHKB

EXO1

NOP10

SELENOI

P2RX7

KCTD7

CHI3L1

PLCG1

SLC25A17

PDE5A

CNTFR

NEFM

ABCD4

RAB5A

NDUFA4

MLANA

PCYT1A

SLC52A2

PIP5K1C

ABCG8

RARA

GLP1R

TRAPPC9

SH2D2A

KCNJ6

CD33

CAST

P2RY12

HLA-DRB4

NAB1

ATPAF2

NCAPH2

LTB

GLDN

TBX4

UFM1

SNX14

DSTYK

RAB33A

HAAO

AHCY

REL

EIF2S2

SDHAF1

RNF125

CPT1C

LOC117038795

SYNJ2

MT-RNR1

NDRG2

GZMB

UBE2N

COL12A1

AGXT

ATCAY

ABCA7

PRRX1

PDSS2

CD70

SLC17A7

UTRN

BIRC2

PADI2

EPHA3

GPX1

DPYSL2

TPPP3

KCND3

GPI

CCL17

MAVS

POLR3K

LAMA1

PTPN6

TSFM

MMP19

EFNB3

RAB8A

CLPP

CLDN5

SYNGR2

HIKESHI

AMPD2

HSD3B7

POLR2L

DDB1

RAB3GAP2

AOC3

VDAC1

DCTN2

PVR

APRT

CDH3

ABCG1

PHF6

NAMPT

EARS2

SHMT2

PNMA2

ABCD3

CD58

UQCRC1

FEN1

COL13A1

APOD

EXOSC9

FURIN

MID2

DPP4

PGBD3

RARS2

SLC30A10

CDK5R1

TOMM40

HADH

MMP12

TTBK2

PDSS1

PAM

INCA1

NOTCH4

MSMB

FHL2

ADAM22

F2RL1

TOP3A

PTPN2

TBCE

ATAD3A

MIR20B

HGS

NAGS

PEX11B

WARS2

NFIA

ENPP2

IFIT1

KCNA4

AHNAK

OLIG1

CLEC16A

GDF15

COX14

RPA1

MAN2A1

CFAP47

CS

PYCR1

GPM6B

PHYH

MGME1

DHX37

COA5

MAT2A

FGD1

PIGB

CGA

TRPM8

DDHD1

TNFRSF10B

MARCKS

CD9

RXRA

FIS1

EFNA5

NDRG3

AHSG

MCF2

ELAC2

SHPK

HSPA1A

THRB

MOV10

ATP5MK

MCAM

AIM2

HSPE1

ACOT7

GFI1

TLR8

ATF2

FAR1

GNB5

TAPBP

MAP3K13

SOD3

HDAC9

CXCR1

CD47

DDX41

ABCA12

IFRD1

IL22

UNC80

SCN2B

TGM1

COA7

DNM3

UBE2G1

PPARD

CTH

RMDN3

HMGA1

MIA2

ZMIZ1

HINT1

NT5C2

CD52

CX3CL1

GNPAT

TRMT10A

ASCC1

MSTO1

SRSF1

NOD1

SELPLG

TBC1D20

MAP1LC3A

ADAM10

PLEKHG4

CGAS

MMADHC

MBNL1

CETP

ATP5PO

PYROXD1

TCF7L2

SACM1L

APOC3

YME1L1

PET117

ACO1

PLA2G4A

TSPAN2

MYO1D

MSR1

EHMT2

ICAM3

IRF9

MPLKIP

RAVER2

POLR1A

LPAR1

RPS6KB1

GATAD1

DNAAF4-CCPG1

MTO1

H2AX

ANTXR1

IQGAP1

UQCRFS1

KIF4A

KLC2

DHX16

IFT81

UQCRQ

ADPRS

CDH15

AFF2

TSG101

ANXA1

TRIT1

ADGRE5

CAPN2

AP5Z1

CCND3

WDR45B

MTMR7

ITGA1

EDN2

ITGAV

SMCR8

ABCA3

HPX

PLXNC1

APOBEC3G

MARS2

GLO1

ATP2B2

MST1R

SEMA4D

PIK3R4

SLC8A1

TRAK1

SIGLEC1

COPS5

H2AC20

SUMF2

APOA4

AP1S1

PRKAB1

ATOX1

TP53BP1

SUGCT

PALS1

TEKT3

HSPA12A

MDC1

HACD1

TJP2

GTF2H5

STK39

ENTPD2

PDYN-AS1

CMKLR1

POLR2B

LMBRD1

ACSBG1

CEBPB

KLC1

MTMR1

LPA

ATXN8

ATRN

WDR48

HEG1

HSPB3

TMPO

DVL1

CTSH

MRM2

OGG1

SCYL1

ISCA2

RMND1

CSF2RA

ARSH

CORO1A

HSPB2

INPP5B

D2HGDH

SGO2

TGM6

YUHAL

TRIM5

ARL6IP1

RNF113A

NECTIN2

LMAN1

FOXD3

STRADB

IFNG-AS1

GTF2E2

RPIA

RAB4A

PDIA4

RAN

RORC

TNNT1

PANX1

PSMD4

NR1I2

HYOU1

LYRM7

PTPRJ

SYNM

CLDN1

NISCH

IFI27

ST8SIA2

PTPN12

QKI

NDUFAF7

SUN2

MIR33A

MSTN

AP1B1

ZFYVE27

A2M

FKBP4

PLTP

FAM180A

STH

ST8SIA4

TREM1

RHOD

CES1

MGAM

CCL19

CLCF1

ATIC

DNAJB1

TFB1M

IGBP1

SMARCA1

MARCKSL1

CD200

VAV1

ZFR

IL21R

RSAD2

BLZF1

LOC108663996

IL11

UMPS

CACNG3

SLC13A5

RXRB

P2RX3

SCD

VWA1

CAND1

FGF21

ELOVL5

CD6

CKAP2

SLC27A2

ECM1

TRMT5

MTHFS

ACADSB

SREBF2

ICAM2

RHOT1

TMPRSS6

TRIM25

POLRMT

TOMM20

UQCC3

KPNB1

TUBB6

CLPX

YBX1

GJC1

RBX1

DDX39B

HBS1L

NDRG4

LGALS4

FOSL1

ATF3

BAG5

HMGN1

CBSL

GAS5

IFIT2

SPIB

MPC1

ACSS2

RBBP4

SYVN1

PLP2

EIF5

CYB561

UBE2K

IL27

PRPS2

DDIT3

MMP8

NCOA3

DCAF1

CAPN8

UQCC2

RHAG

SAA4

COX20

ESD

SUPV3L1

GJA3

BCYRN1

FRMD4A

DNASE2

CYP7A1

KIF2B

NRG2

PGAM1

SORD

RAB21

ATG13

MADCAM1

PUM1

ORM1

OPN4

HTR2B

TNFRSF21

BST2

FBXO38

MAGI2

TMEM240

CD226

SI

MAPKAPK2

PARS2

SMAD9

EMP2

SLC1A6

PDE3B

UBE2I

COMMD1

CD200R1

CELF1

PLLP

NKX2-6

UBE2S

DHX36

TNFSF8

BTG1

SPTLC3

SLC31A1

DLX4

CUL5

SMPD2

NUPR1

DSCAML1

MIR26A1

GRK2

DDR1

ARMC8

NANOG

ZIC4

ACOT12

EIF5AL1

ACOT13

NRG3

MARCHF3

EIF1

PITPNA

PREP

AMFR

PCTP

PI4K2A

TNFRSF19

RBFOX1

PSMF1

LIPG

ADI1

PRDM2

VTI1A

FECH

ACOT11

BDKRB1

HOXB3

AMPD1

SLC25A45

KHSRP

GNB2

COX4I2

EXOC4

MTMR12

PLD1

HOXD10

FLOT1

IKZF3

WIPI1

UBE4A

DHX58

KCNH6

FLRT1

ZNF608

KCNJ3

LARS2-AS1

SLC7A13

RILP

ATP1A1-AS1

H4C1

STAM

VPS29

MMAB

LINC01194

ARSK

COA6

SOX17

SP140

RNF8

ZACN

SNX9

RER1

TAGAP

MRPS27

ZNF711

HAVCR1

TAPBPL

FDX1

EMP3

GPR17

KIF21B

PLCH2

PHAX

GRAMD2B

ERP44

PABPC1

GZMA

ACOX2

MMAA

YBX3

KLHL9

CMTX3

PRPS1L1

SMPX

MRPL44

PACSIN3

CDC5L

MILR1

CBLB

RABGAP1

NFATC3

MMP11

OMP

PLXNA1

FDPS

STAC2

PARL

RAB6B

STX17

PLEKHA1

MAPK9

NAIP

ARSI

ALDH4A1

RHOT2

HPD

MALL

GTF2H4

SPHK1

INTS4

GGPS1

SUCLG2

MTMR3

ACY3

PRDM9

MVD

ICMT

CCNQ

KIF17

SIPA1L2

RGS6

IL34

CSH1

CLINT1

SIRPA

PTCD1

CCL22

GNA15

EVI5

DAB2

PSMC5

ACAD8

UBXN7

ACSS1

SMARCA5

RAB32

FES

PAF1

MROH7

SLIRP

OTUB1

ALDH3A1

GAL3ST1

H1-0

CYBRD1

ILDR1

OMA1

SLC6A6

GPC6

CEL

HSPB11

TTF2

LRIT1

TBX22

CYP46A1

FEZ1

FYB1

CAPN15

IRF2

FGD3

PSMD14

DNTT

TNFSF9

USP14

ITGB8

FAT2

EPS15L1

UIMC1

IDI1

BCKDK

TTYH1

MINAR2

ADARB2

GP6

BEAN1

PIK3R5

KIF20B

SQLE

SCRT1

NVL

APOC2

RNF170

RBL2

CCNT1

RABAC1

PFN2

MTCH1

SCAP

PRSS3

PASK

TEX43

REEP2

PARP2

NCL

AGPS

EXTL2

PRMT1

WDR36

TRA

SLC12A7

ASZ1

UBE2D1

COX6C

PACSIN2

TIMM13

GNLY

GBGT1

PMVK

KRTAP9-9

PRIMPOL

MTCL1

INTS5

ACSL6

GATAD2A

CDIP1

CUL4A

APOBEC3F

SFN

ATP1B1

CKMT1B

MIR584

CDK5RAP1

FUT4

BZW2

KLRB1

GOLM1

FUT1

MX2

RAB31

TCEA1

CBX3

SIRPB1

TRPV3

SPN

TBATA

POU4F2

TRIM56

POLR2D

DENR

ADPRH

TMPRSS5

GPS1

KLRD1

TAF8

PRPSAP2

COPS6

HOXA5

CST7

CTNNBL1

NLRX1

RBM17

EIF2B5-DT

MIR422A

CCR5AS

KCNA3

ZNF362

SUN1

PNPLA3

AZIN1

DRG1

CDK7

EGR3

DGAT2

RBPMS

C5orf63

NGF-AS1

LRTOMT

AMBP

TEFM

MYO1A

MGAT5

LIPN

MITD1

PRPSAP1

CTBS

CSH2

RNF26

FABP3

MOCS3

ZNF804A

JPH4

C1orf194

FDX2

SYBU

RXRG

TSPAN4

MIR638

LAPTM5

ABT1

SCAMP4

MAL2

ELOA

CCS

USF1

XAB2

COA4

CRB3

LILRB4

RPS6KC1

TASOR

GFRA4

PLCB2

CADM4

MIR1275

CDRT1

NPC1L1

SAFB

DDX23

MSS51

ZBP1

TVP23B

IFI30

MIR599

SLC25A48

RMC1

LOC105943586

PSMD7

CCPG1

SNORA40

KRBOX4

PTCD2

UBXN6

MTMR6

GET4

ENTPD7

MT-TR

GNPDA2

SVIP

ANGPTL4

KLHL2

H2AC13

NOM1

IL32

CTTNBP2

GDAP1L1

SSBP3

SLC44A4

YRDC

PDZD9

FYCO1

MT-TM

ANGPTL3

PYDC1

MIR664A

BCAS1

FBLN2

KIF13B

ABCD1P4

ABCD1P2

ABCD1P3

ABCD1P1

UCHL5

SYS1

VCPIP1

CUTC

TFB2M

DAND5

GJD3

SLC25A47

MARCHF5

NAAA

RAB11FIP2

MARCHF9

MIR18B

OBSCN

NSRP1

TNFRSF10C

TMEM119

ACYP1

SPTSSA

CCL8

UBXN10

COX10-DT

MPZL1

GRK5

DHRS4

NSUN3

STMN4

KLK11

ARHGEF15

ATXN3L

CLEC10A

CKMT1A

LILRB2

MFAP2

MIEF2

PHF23

ATP5MJ

MEX3D

PLS1

KCNS2

WNK3

GPC5

CYP4F2

CERS6

RNF115

EME2

RNF152

ARHGAP9

MMP25

SRXN1

CYP4F3

KLHDC7A

SYNC

LPCAT4

TTC7B

CLDN9

C1D

MS

GPRASP2

CSMD2

GTF2H1

CWC15

PDE7A

RNF122

RGS1

SPHK2

ATP1A4

GTF2H2

RTN1

PRORP

UVSSA

TNFRSF10D

RSC1A1

IFI44L

SCCPDH

SLC12A4

PDPR

SLA

MBD6

BPHL

NEIL1

SPTSSB

RFLNA

MCM3AP-AS1

MMP10

ASB16

UCHL3

CCL13

YIPF3

COX5B

GLTP

IZUMO1

ECH1

PLA2G1B

RAPGEF5

ST8SIA1

MICU3

AGTRAP

PRDX2

SLC25A18

AQP7

DEGS2

TLDC2

PIPOX

TMCO6

PPHLN1

ZFAND2B

CSNK1A1

SPATA5L1

FBXO48

RPL7L1

SNRNP40

THADA

ACOX3

CMYA5

TEN1

DENND2B

LOC109461484

XPO6

RIDA

LOC112081413

SGMS1-AS1

DNAH10

TRMT61B

MT-RNR2

SMTNL2

MPHOSPH8

AZGP1

ARHGAP39

IL17B

LOC108660406

COX7C

PEX5L

NEU4

XPO7

MRPL18

LOC112529895

LOC113788297

GTF2H3

APMAP

MIEF1

ADAMTS20

S1PR4

H2AC12

SLC35B2

GPR65

MRRF

CCL27

TREML1

PNPLA7

EGR4

TREML4

YPEL3

HSPB6

PPP4R4

NDUFB6

TCEA3

MIR4709

FCAR

DLEU1

NEIL2

LRIG3

TMEM65

AKR7A2

ATG16L2

EMC4

WDR7

DDIT4L

CCL1

SLC24A3

COPG1

LYPLA1

UBE2U

A3GALT2

MYO1G

MAP7D3

CSTF1

PYCR3

CCNDBP1

OSGEPL1

ABCG4

RBM7

SLC25A27

MANEA

S1PR5

TREX2

PRB4

RABEP2

PHOSPHO1

GPR22

MT-TG

ERV3-1

SH2D3C

ATXN1L

ATP1B4

ZCWPW1

PIGBOS1

DEFB128

PGPEP1

ELMO3

ENTPD5

PIRC16

KLHL1

ST3GAL2

YIPF2

ME3

MAPK4

POGK

AHRR

AP5B1

PEF1

ERMN

MTERF1

TSBP1

DMAC2L

SEC31B

ARRDC4

VWA7

TBKBP1

H2AC1

ELF2

C13orf42

PRM2

COLEC12

PLEKHM3

IFNA10

PNRC1

STAMBPL1

CCDC87

S100PBP

LOC108663985

MIR376A1

DNAJC28

CFAP126

LENG1

SNPH

RNASEH2B-AS1

ST20-MTHFS

MT-TD

ZNF165

HTATIP2

GLRX2

TRIM15

DHRS11

TERB2

JOSD1

CMTR2

AGMO

TAS2R4

ATXN7L3B

LINC01006

OR2H1

SCP2D1

MIR873

LOC285626

C1orf185

MMP28

MIR1538

SCAANT1

CCL4L1

OR12D3

JOSD2

CRYGN

HNF1A-AS1

MS4A6A

LOC108663993

OR4L1

TRL-AAG2-3

SNORD75

SNORA72

ZSCAN20

MZT1

LPAR5

CCDC177

MT1XP1

TRPM2-AS

MIR219A2

LOC110596866

ST13P4

MIR448
