# Supplementary material for: Study on the Anti-demyelination Mechanism of Bu-Shen-Yi-Sui Capsule in the Central Nervous System Based on Network Pharmacology and Experimental Verification
Source: Mediators Inflamm. 2022 Jul 12;2022:9241261. doi: 10.1155/2022/9241261 (PMC9296285; doi:10.1155/2022/9241261)
Supplement: Supplementary Materials — Table S1: all the potential targets of BSYS Capsule. Table S2: known CNSD-related targets. Table S3: BSYS Capsule shared 227 intersection targets with known CNSD-related targets. Table S4: PPI information of 227 intersection targets in Metascape. Table S5: the degree values of all nodes in the PPI network. Table S6: results for GO pathway enrichment analysis. Table S7: results for KEGG pathway enrichment analysis. Table S8: information of gene-pathway network. Table S9: information of the “active ingredients-intersection targets” network. [file 9241261.f1.zip › Table S3.docx]

Gene Symbol

PGR

RXRA

PTGS2

SLC6A3

PLAU

CHRM3

HTR2A

CHRNA7

PIK3CG

SLC6A4

OPRM1

BCL2

BAX

CASP9

JUN

CASP3

CASP8

PRKCA

TGFB1

PON1

MAP2

RELA

AR

PPARG

DPP4

MMP3

NOS2

ACHE

EGFR

AKT1

VEGFA

BCL2L1

FOS

CDKN1A

MMP2

MMP9

MAPK1

IL10

EGF

TNF

IL6

TP53

ODC1

XDH

TOP1

SOD1

MMP1

HIF1A

STAT1

ESR2

KDR

PPARD

ESR1

MYC

IL1B

GPT

HMOX1

NFE2L2

TLR4

ATF3

DDIT3

GDNF

SNCA

BDNF

MAPK3

NGF

NTRK1

NTRK2

MMP12

HSP90AA1

CA2

AKR1B1

APP

MMP7

MMP8

CAT

GCLC

SI

MGAM

TYR

FOLH1

FUCA1

CDK1

HTR2C

CYP19A1

MCL1

CDK5

GLO1

SYK

PARP1

TTR

ABCG2

CDK6

ABCB1

F2

MPO

MAPK14

FASLG

SIRT1

STAT3

MAPK8

BAK1

IGF1R

EDN1

FOXO1

GJA1

ICAM1

CCL2

SELE

VCAM1

CXCL8

BRCA1

SOD2

PRKCB

PECAM1

BIRC5

PLAT

CDK7

IL1A

TNFRSF10B

XIAP

PPARA

CRP

CD80

JAK1

AGTR1

CFLAR

IL17B

C5AR1

MTOR

CCR2

EIF2S1

TNFSF10

TNFRSF10A

BIRC3

CDC42

CD28

NR1H3

NFKB1

MDM2

ERBB2

IL2

IFNG

IL4

GSTP1

INSR

CD40LG

GABBR1

GNRH1

ALDH3A1

GJB1

GRM1

VCP

RXRB

CYP3A4

NR1I2

KCNMA1

BCHE

TPI1

GAMT

PRSS3

GM2A

PRKCG

TRPV4

HMGCR

PSEN1

PSEN2

PAM

ENPP2

CTSB

MMP10

CREB1

ATF2

CSF2

PTPN6

LITAF

CASP1

NPC1L1

NR1H4

SHH

GPX1

GPX4

TDP1

GRIA2

TRPV1

RXRG

TNFRSF1A

ALOX5

PLA2G4A

GLB1

UCP2

C1R

CETP

ABCG1

TNFRSF1B

HRH3

CCR1

CCR3

FDFT1

CSF1R

VDR

PRKG1

EIF2AK3

BIRC2

HTR1A

TACR1

EP300

CTSD

REN

SCN9A

IDH1

BDKRB1

MAPK10

CCR5

MAPK9

TYK2

DHFR

DRD2

PADI4

TGFBR1

PDE5A

HTR2B

EIF2AK4

EIF2AK2

EIF2AK1

RET

TMPRSS6
