# Supplementary material for: Study on the Anti-demyelination Mechanism of Bu-Shen-Yi-Sui Capsule in the Central Nervous System Based on Network Pharmacology and Experimental Verification
Source: Mediators Inflamm. 2022 Jul 12;2022:9241261. doi: 10.1155/2022/9241261 (PMC9296285; doi:10.1155/2022/9241261)
Supplement: Supplementary Materials — Table S1: all the potential targets of BSYS Capsule. Table S2: known CNSD-related targets. Table S3: BSYS Capsule shared 227 intersection targets with known CNSD-related targets. Table S4: PPI information of 227 intersection targets in Metascape. Table S5: the degree values of all nodes in the PPI network. Table S6: results for GO pathway enrichment analysis. Table S7: results for KEGG pathway enrichment analysis. Table S8: information of gene-pathway network. Table S9: information of the “active ingredients-intersection targets” network. [file 9241261.f1.zip › Table S4.docx]

SUID canonicalName DEGREE Gene id MCODE_CLUSTER_ID MCODE_SCORE MCODE_TYPE name shared name Symbol

1300 AKT1 148 207 207 3 8.558823529 Clustered 207 207 AKT1

1450 MAPK1 134 5594 5594 2 15.05714286 Clustered 5594 5594 MAPK1

1451 MAPK3 129 5595 5595 2 15.05714286 Clustered 5595 5595 MAPK3

1409 JUN 123 3725 3725 2 15.05714286 Clustered 3725 3725 JUN

1359 EGFR 117 1956 1956 2 15.05714286 Clustered 1956 1956 EGFR

1426 NFKB1 115 4790 4790 2 15.05714286 Clustered 4790 4790 NFKB1

1346 MAPK14 113 1432 1432 2 15.05714286 Clustered 1432 1432 MAPK14

1363 ESR1 111 2099 2099 2 15.05714286 Clustered 2099 2099 ESR1

1478 STAT3 106 6774 6774 2 15.05714286 Clustered 6774 6774 STAT3

1361 EP300 103 2033 2033 2 15.05714286 Seed 2033 2033 EP300

1391 HSP90AA1 103 3320 3320 2 15.05714286 Clustered 3320 3320 HSP90AA1

1429 NTRK1 103 4914 4914 2 15.05714286 Clustered 4914 4914 NTRK1

1308 APP 102 351 351 3 8.558823529 Clustered 351 351 APP

1332 CDC42 101 998 998 2 15.05714286 Clustered 998 998 CDC42

1408 JAK1 100 3716 3716 3 8.558823529 Clustered 3716 3716 JAK1

1445 PPARG 100 5468 5468 2 15.05714286 Clustered 5468 5468 PPARG

1452 MAPK8 97 5599 5599 2 15.05714286 Clustered 5599 5599 MAPK8

1479 SYK 96 6850 6850 2 15.05714286 Clustered 6850 6850 SYK

1488 TP53 96 7157 7157 3 8.558823529 Clustered 7157 7157 TP53

1310 AR 95 367 367 2 15.05714286 Clustered 367 367 AR

1324 CASP3 94 836 836 3 8.558823529 Clustered 836 836 CASP3

1407 INSR 94 3643 3643 2 15.05714286 Clustered 3643 3643 INSR

1461 RELA 91 5970 5970 2 15.05714286 Clustered 5970 5970 RELA

1369 FOS 89 2353 2353 2 15.05714286 Clustered 2353 2353 FOS

1430 NTRK2 89 4915 4915 2 15.05714286 Clustered 4915 4915 NTRK2

1370 MTOR 88 2475 2475 3 8.558823529 Clustered 2475 2475 MTOR

1484 TNF 87 7124 7124 3 8.558823529 Clustered 7124 7124 TNF

1482 TGFBR1 85 7046 7046 3 8.558823529 Clustered 7046 7046 TGFBR1

1483 TLR4 85 7099 7099 2 15.05714286 Clustered 7099 7099 TLR4

1362 ERBB2 84 2064 2064 2 15.05714286 Clustered 2064 2064 ERBB2

1446 PRKCA 84 5578 5578 3 8.558823529 Clustered 5578 5578 PRKCA

1477 STAT1 83 6772 6772 2 15.05714286 Clustered 6772 6772 STAT1

1331 CDK1 81 983 983 2 15.05714286 Clustered 983 983 CDK1

1424 MYC 79 4609 4609 3 8.558823529 Clustered 4609 4609 MYC

1491 TYK2 77 7297 7297 2 15.05714286 Clustered 7297 7297 TYK2

1367 FOXO1 76 2308 2308 2 15.05714286 Clustered 2308 2308 FOXO1

1325 CASP8 75 841 841 3 8.558823529 Clustered 841 841 CASP8

1344 ATF2 75 1386 1386 2 15.05714286 Clustered 1386 1386 ATF2

1459 PTGS2 73 5743 5743 3 8.558823529 Clustered 5743 5743 PTGS2

1453 MAPK9 71 5601 5601 3 8.558823529 Clustered 5601 5601 MAPK9

1414 MDM2 70 4193 4193 3 8.558823529 Clustered 4193 4193 MDM2

1438 PIK3CG 69 5294 5294 2 15.05714286 Clustered 5294 5294 PIK3CG

1514 SIRT1 68 23411 23411 3 8.558823529 Clustered 23411 23411 SIRT1

1495 VCP 67 7415 7415 3 8.558823529 Clustered 7415 7415 VCP

1364 ESR2 66 2100 2100 1 3.527777778 Clustered 2100 2100 ESR2

1411 KDR 66 3791 3791 2 15.05714286 Clustered 3791 3791 KDR

1464 RXRA 65 6256 6256 3 8.558823529 Clustered 6256 6256 RXRA

1481 TGFB1 65 7040 7040 2 15.05714286 Clustered 7040 7040 TGFB1

1315 BCL2 64 596 596 3 8.558823529 Clustered 596 596 BCL2

1436 PGR 64 5241 5241 2 15.05714286 Clustered 5241 5241 PGR

1330 CD40LG 63 959 959 3 8.558823529 Clustered 959 959 CD40LG

1343 CREB1 62 1385 1385 3 8.558823529 Clustered 1385 1385 CREB1

1388 HIF1A 62 3091 3091 3 8.558823529 Clustered 3091 3091 HIF1A

1496 VDR 62 7421 7421 3 8.558823529 Clustered 7421 7421 VDR

1443 PPARA 61 5465 5465 1 3.527777778 Clustered 5465 5465 PPARA

1347 CSF1R 60 1436 1436 2 15.05714286 Clustered 1436 1436 CSF1R

1299 AGTR1 59 185 185 1 3.527777778 Clustered 185 185 AGTR1

1323 CASP1 59 834 834 1 3.527777778 Clustered 834 834 CASP1

1342 CCR5 59 1234 1234 1 3.527777778 Clustered 1234 1234 CCR5

1454 MAPK10 59 5602 5602 3 8.558823529 Clustered 5602 5602 MAPK10

1358 EGF 57 1950 1950 2 15.05714286 Clustered 1950 1950 EGF

1334 CDK6 56 1021 1021 3 8.558823529 Clustered 1021 1021 CDK6

1305 BIRC3 55 330 330 1 3.527777778 Clustered 330 330 BIRC3

1326 CASP9 55 842 842 1 3.527777778 Clustered 842 842 CASP9

1316 BCL2L1 53 598 598 1 3.527777778 Clustered 598 598 BCL2L1

1336 CDKN1A 52 1026 1026 2 15.05714286 Clustered 1026 1026 CDKN1A

1356 DRD2 52 1813 1813 4 2.448275862 Clustered 1813 1813 DRD2

1357 EDN1 52 1906 1906 5 2.666666667 Clustered 1906 1906 EDN1

1401 IL1B 52 3553 3553 1 3.527777778 Clustered 3553 3553 IL1B

1405 CXCL8 52 3576 3576 4 2.448275862 Clustered 3576 3576 CXCL8

1428 NOS2 52 4843 4843 3 8.558823529 Clustered 4843 4843 NOS2

1311 ATF3 51 467 467 3 8.558823529 Clustered 467 467 ATF3

1319 BRCA1 51 672 672 3 8.558823529 Seed 672 672 BRCA1

1340 CCR1 51 1230 1230 1 3.527777778 Clustered 1230 1230 CCR1

1463 RET 51 5979 5979 2 15.05714286 Clustered 5979 5979 RET

1504 CFLAR 51 8837 8837 1 3.527777778 Clustered 8837 8837 CFLAR

1432 OPRM1 50 4988 4988 5 2.666666667 Seed 4988 4988 OPRM1

1441 PLAU 50 5328 5328 1 3.527777778 Clustered 5328 5328 PLAU

1460 PTPN6 50 5777 5777 3 8.558823529 Clustered 5777 5777 PTPN6

1398 IFNG 49 3458 3458 1 3.527777778 Clustered 3458 3458 IFNG

1447 PRKCB 48 5579 5579 1 3.527777778 Clustered 5579 5579 PRKCB

1507 ABCG2 48 9429 9429 1 3.527777778 Clustered 9429 9429 ABCG2

1304 BIRC2 47 329 329 4 2.448275862 Clustered 329 329 BIRC2

1306 XIAP 46 331 331 1 3.527777778 Clustered 331 331 XIAP

1423 MPO 46 4353 4353 5 2.666666667 Clustered 4353 4353 MPO

1425 NFE2L2 46 4780 4780 3 8.558823529 Clustered 4780 4780 NFE2L2

1444 PPARD 45 5467 5467 1 3.527777778 Clustered 5467 5467 PPARD

1511 NR1H4 45 9971 9971 1 3.527777778 Clustered 9971 9971 NR1H4

1333 CDK5 44 1020 1020 4 2.448275862 Clustered 1020 1020 CDK5

1387 GSTP1 44 2950 2950 4 2.448275862 Seed 2950 2950 GSTP1

1404 IL6 44 3569 3569 3 8.558823529 Clustered 3569 3569 IL6

1413 MCL1 44 4170 4170 1 3.527777778 Clustered 4170 4170 MCL1

1457 PSEN1 44 5663 5663 4 2.448275862 Clustered 5663 5663 PSEN1

1475 SOD1 44 6647 6647 4 2.448275862 Clustered 6647 6647 SOD1

1341 CCR3 43 1232 1232 4 2.448275862 Clustered 1232 1232 CCR3

1365 F2 43 2147 2147 4 2.448275862 Clustered 2147 2147 F2

1402 IL2 43 3558 3558 3 8.558823529 Clustered 3558 3558 IL2

1448 PRKCG 43 5582 5582 5 2.666666667 Clustered 5582 5582 PRKCG

1497 VEGFA 43 7422 7422 4 2.448275862 Clustered 7422 7422 VEGFA

1313 BAX 42 581 581 1 3.527777778 Clustered 581 581 BAX

1420 MMP9 42 4318 4318 6 1.5 Clustered 4318 4318 MMP9

1307 BIRC5 41 332 332 1 3.527777778 Clustered 332 332 BIRC5

1350 CTSD 41 1509 1509 1 3.527777778 Clustered 1509 1509 CTSD

1449 PRKG1 41 5592 5592 1 3.527777778 Seed 5592 5592 PRKG1

1505 NR1I2 41 8856 8856 1 3.527777778 Clustered 8856 8856 NR1I2

1512 NR1H3 41 10062 10062 1 3.527777778 Clustered 10062 10062 NR1H3

1298 PARP1 40 142 142 3 8.558823529 Clustered 142 142 PARP1

1353 DDIT3 40 1649 1649 1 3.527777778 Clustered 1649 1649 DDIT3

1403 IL4 40 3565 3565 4 2.448275862 Clustered 3565 3565 IL4

1406 IL10 40 3586 3586 1 3.527777778 Clustered 3586 3586 IL10

1439 PLA2G4A 40 5321 5321 1 3.527777778 Clustered 5321 5321 PLA2G4A

1327 CAT 39 847 847 4 2.448275862 Clustered 847 847 CAT

1351 CYP3A4 39 1576 1576 4 2.448275862 Clustered 1576 1576 CYP3A4

1352 CYP19A1 39 1588 1588 1 3.527777778 Clustered 1588 1588 CYP19A1

1455 EIF2AK2 39 5610 5610 3 8.558823529 Clustered 5610 5610 EIF2AK2

1476 SOD2 39 6648 6648 4 2.448275862 Clustered 6648 6648 SOD2

1392 HTR1A 38 3350 3350 4 2.448275862 Clustered 3350 3350 HTR1A

1468 CCL2 38 6347 6347 4 2.448275862 Clustered 6347 6347 CCL2

1318 BDNF 37 627 627 3 8.558823529 Clustered 627 627 BDNF

1348 CSF2 37 1437 1437 5 2.666666667 Clustered 1437 1437 CSF2

1437 ABCB1 37 5243 5243 1 3.527777778 Clustered 5243 5243 ABCB1

1485 TNFRSF1A 36 7132 7132 4 2.448275862 Clustered 7132 7132 TNFRSF1A

1375 GJA1 34 2697 2697 1 3.527777778 Clustered 2697 2697 GJA1

1393 HTR2A 34 3356 3356 5 2.666666667 Clustered 3356 3356 HTR2A

1415 MMP1 34 4312 4312 6 1.5 Clustered 4312 4312 MMP1

1465 RXRB 34 6257 6257 1 3.527777778 Clustered 6257 6257 RXRB

1469 SELE 34 6401 6401 0 0 6401 6401 SELE

1317 BDKRB1 33 623 623 0 0 623 623 BDKRB1

1399 IGF1R 33 3480 3480 1 3.527777778 Clustered 3480 3480 IGF1R

1427 NGF 33 4803 4803 1 3.527777778 Clustered 4803 4803 NGF

1440 PLAT 33 5327 5327 1 3.527777778 Clustered 5327 5327 PLAT

1386 GRM1 32 2911 2911 0 0 2911 2911 GRM1

1474 SNCA 32 6622 6622 0 0 6622 6622 SNCA

1385 GRIA2 31 2891 2891 0 0 2891 2891 GRIA2

1466 RXRG 31 6258 6258 4 2.448275862 Clustered 6258 6258 RXRG

1416 MMP2 30 4313 4313 6 1.5 Clustered 4313 4313 MMP2

1494 VCAM1 30 7412 7412 0 0 7412 7412 VCAM1

1309 FASLG 29 356 356 4 2.448275862 Clustered 356 356 FASLG

1395 HTR2C 29 3358 3358 5 2.666666667 Clustered 3358 3358 HTR2C

1510 ABCG1 29 9619 9619 4 2.448275862 Clustered 9619 9619 ABCG1

1302 AKR1B1 27 231 231 8 1 Clustered 231 231 AKR1B1

1312 BAK1 27 578 578 1 3.527777778 Clustered 578 578 BAK1

1360 EIF2S1 27 1965 1965 0 0 1965 1965 EIF2S1

1458 PSEN2 27 5664 5664 0 0 5664 5664 PSEN2

1297 ACHE 26 43 43 4 2.448275862 Clustered 43 43 ACHE

1349 CTSB 26 1508 1508 0 0 1508 1508 CTSB

1396 ICAM1 26 3383 3383 5 2.666666667 Clustered 3383 3383 ICAM1

1410 KCNMA1 26 3778 3778 0 0 3778 3778 KCNMA1

1394 HTR2B 25 3357 3357 5 2.666666667 Clustered 3357 3357 HTR2B

1487 TOP1 25 7150 7150 0 0 7150 7150 TOP1

1314 BCHE 24 590 590 4 2.448275862 Clustered 590 590 BCHE

1328 CD28 24 940 940 4 2.448275862 Clustered 940 940 CD28

1400 IL1A 24 3552 3552 4 2.448275862 Clustered 3552 3552 IL1A

1513 HRH3 24 11255 11255 4 2.448275862 Clustered 11255 11255 HRH3

1335 CDK7 23 1022 1022 4 2.448275862 Clustered 1022 1022 CDK7

1390 HMOX1 22 3162 3162 0 0 3162 3162 HMOX1

1397 IDH1 22 3417 3417 6 1.5 Clustered 3417 3417 IDH1

1462 REN 22 5972 5972 0 0 5972 5972 REN

1473 SLC6A4 22 6532 6532 0 0 6532 6532 SLC6A4

1417 MMP3 21 4314 4314 0 0 4314 4314 MMP3

1470 SHH 21 6469 6469 0 0 6469 6469 SHH

1418 MMP7 20 4316 4316 0 0 4316 4316 MMP7

1383 GPX1 19 2876 2876 8 1 Clustered 2876 2876 GPX1

1486 TNFRSF1B 19 7133 7133 0 0 7133 7133 TNFRSF1B

1498 TRPV1 19 7442 7442 0 0 7442 7442 TRPV1

1456 PRSS3 18 5646 5646 6 1.5 Clustered 5646 5646 PRSS3

1467 SCN9A 18 6335 6335 0 0 6335 6335 SCN9A

1321 C5AR1 17 728 728 4 2.448275862 Clustered 728 728 C5AR1

1355 DPP4 17 1803 1803 0 0 1803 1803 DPP4

1366 FDFT1 17 2222 2222 0 0 2222 2222 FDFT1

1372 GABBR1 17 2550 2550 4 2.448275862 Clustered 2550 2550 GABBR1

1472 SLC6A3 17 6531 6531 0 0 6531 6531 SLC6A3

1499 XDH 17 7498 7498 0 0 7498 7498 XDH

1502 TNFRSF10B 17 8795 8795 0 0 8795 8795 TNFRSF10B

1493 UCP2 16 7351 7351 0 0 7351 7351 UCP2

1500 PDE5A 16 8654 8654 0 0 8654 8654 PDE5A

1508 EIF2AK3 16 9451 9451 0 0 9451 9451 EIF2AK3

1522 CCR2 16 729230 729230 4 2.448275862 Clustered 729230 729230 CCR2

1345 CRP 15 1401 1401 0 0 1401 1401 CRP

1489 TPI1 15 7167 7167 0 0 7167 7167 TPI1

1301 ALDH3A1 14 218 218 0 0 218 218 ALDH3A1

1384 GPX4 14 2879 2879 8 1 Seed 2879 2879 GPX4

1412 MAP2 14 4133 4133 0 0 4133 4133 MAP2

1377 GLB1 13 2720 2720 7 1.5 Clustered 2720 2720 GLB1

1389 HMGCR 13 3156 3156 0 0 3156 3156 HMGCR

1419 MMP8 13 4317 4317 0 0 4317 4317 MMP8

1431 ODC1 13 4953 4953 0 0 4953 4953 ODC1

1435 PECAM1 13 5175 5175 0 0 5175 5175 PECAM1

1480 TACR1 13 6869 6869 5 2.666666667 Clustered 6869 6869 TACR1

1490 TTR 13 7276 7276 7 1.5 Clustered 7276 7276 TTR

1516 EIF2AK1 13 27102 27102 0 0 27102 27102 EIF2AK1

1338 CHRM3 12 1131 1131 5 2.666666667 Clustered 1131 1131 CHRM3

1379 GLO1 12 2739 2739 0 0 2739 2739 GLO1

1381 GNRH1 12 2796 2796 5 2.666666667 Clustered 2796 2796 GNRH1

1506 MGAM 12 8972 8972 0 0 8972 8972 MGAM

1322 CA2 11 760 760 0 0 760 760 CA2

1434 ENPP2 11 5168 5168 0 0 5168 5168 ENPP2

1503 TNFRSF10A 11 8797 8797 0 0 8797 8797 TNFRSF10A

1521 EIF2AK4 11 440275 440275 0 0 440275 440275 EIF2AK4

1303 ALOX5 10 240 240 0 0 240 240 ALOX5

1382 GPT 10 2875 2875 0 0 2875 2875 GPT

1421 MMP10 10 4319 4319 6 1.5 Clustered 4319 4319 MMP10

1519 TRPV4 9 59341 59341 0 0 59341 59341 TRPV4

1329 CD80 8 941 941 0 0 941 941 CD80

1380 GM2A 8 2760 2760 7 1.5 Clustered 2760 2760 GM2A

1422 MMP12 8 4321 4321 0 0 4321 4321 MMP12

1501 TNFSF10 8 8743 8743 0 0 8743 8743 TNFSF10

1368 FOLH1 7 2346 2346 0 0 2346 2346 FOLH1

1371 FUCA1 7 2517 2517 7 1.5 Seed 2517 2517 FUCA1

1376 GJB1 7 2705 2705 0 0 2705 2705 GJB1

1354 DHFR 6 1719 1719 0 0 1719 1719 DHFR

1374 GDNF 6 2668 2668 0 0 2668 2668 GDNF

1378 GCLC 6 2729 2729 0 0 2729 2729 GCLC

1509 LITAF 5 9516 9516 0 0 9516 9516 LITAF

1433 PAM 4 5066 5066 0 0 5066 5066 PAM

1492 TYR 4 7299 7299 0 0 7299 7299 TYR

1518 TDP1 4 55775 55775 0 0 55775 55775 TDP1

1520 TMPRSS6 4 164656 164656 0 0 164656 164656 TMPRSS6

1337 CETP 3 1071 1071 0 0 1071 1071 CETP

1339 CHRNA7 3 1139 1139 0 0 1139 1139 CHRNA7

1442 PON1 3 5444 5444 0 0 5444 5444 PON1

1320 C1R 2 715 715 0 0 715 715 C1R

1515 PADI4 2 23569 23569 0 0 23569 23569 PADI4

1373 GAMT 1 2593 2593 0 0 2593 2593 GAMT

1471 SI 1 6476 6476 0 0 6476 6476 SI

1517 NPC1L1 1 29881 29881 0 0 29881 29881 NPC1L1
