# Supplementary material for: Study on the Anti-demyelination Mechanism of Bu-Shen-Yi-Sui Capsule in the Central Nervous System Based on Network Pharmacology and Experimental Verification
Source: Mediators Inflamm. 2022 Jul 12;2022:9241261. doi: 10.1155/2022/9241261 (PMC9296285; doi:10.1155/2022/9241261)
Supplement: Supplementary Materials — Table S1: all the potential targets of BSYS Capsule. Table S2: known CNSD-related targets. Table S3: BSYS Capsule shared 227 intersection targets with known CNSD-related targets. Table S4: PPI information of 227 intersection targets in Metascape. Table S5: the degree values of all nodes in the PPI network. Table S6: results for GO pathway enrichment analysis. Table S7: results for KEGG pathway enrichment analysis. Table S8: information of gene-pathway network. Table S9: information of the “active ingredients-intersection targets” network. [file 9241261.f1.zip › Table S5.docx]

Gene Symbol DEGREE

AKT1 148

MAPK1 134

MAPK3 129

JUN 123

EGFR 117

NFKB1 115

MAPK14 113

ESR1 111

STAT3 106

EP300 103

HSP90AA1 103

NTRK1 103

APP 102

CDC42 101

JAK1 100

PPARG 100

MAPK8 97

SYK 96

TP53 96

AR 95

CASP3 94

INSR 94

RELA 91

FOS 89

NTRK2 89

MTOR 88

TNF 87

TGFBR1 85

TLR4 85

ERBB2 84

PRKCA 84

STAT1 83

CDK1 81

MYC 79

TYK2 77

FOXO1 76

CASP8 75

ATF2 75

PTGS2 73

MAPK9 71

MDM2 70

PIK3CG 69

SIRT1 68

VCP 67

ESR2 66

KDR 66

RXRA 65

TGFB1 65

BCL2 64

PGR 64

CD40LG 63

CREB1 62

HIF1A 62

VDR 62

PPARA 61

CSF1R 60

AGTR1 59

CASP1 59

CCR5 59

MAPK10 59

EGF 57

CDK6 56

BIRC3 55

CASP9 55

BCL2L1 53

CDKN1A 52

DRD2 52

EDN1 52

IL1B 52

CXCL8 52

NOS2 52

ATF3 51

BRCA1 51

CCR1 51

RET 51

CFLAR 51

OPRM1 50

PLAU 50

PTPN6 50

IFNG 49

PRKCB 48

ABCG2 48

BIRC2 47

XIAP 46

MPO 46

NFE2L2 46

PPARD 45

NR1H4 45

CDK5 44

GSTP1 44

IL6 44

MCL1 44

PSEN1 44

SOD1 44

CCR3 43

F2 43

IL2 43

PRKCG 43

VEGFA 43

BAX 42

MMP9 42

BIRC5 41

CTSD 41

PRKG1 41

NR1I2 41

NR1H3 41

PARP1 40

DDIT3 40

IL4 40

IL10 40

PLA2G4A 40

CAT 39

CYP3A4 39

CYP19A1 39

EIF2AK2 39

SOD2 39

HTR1A 38

CCL2 38

BDNF 37

CSF2 37

ABCB1 37

TNFRSF1A 36

GJA1 34

HTR2A 34

MMP1 34

RXRB 34

SELE 34

BDKRB1 33

IGF1R 33

NGF 33

PLAT 33

GRM1 32

SNCA 32

GRIA2 31

RXRG 31

MMP2 30

VCAM1 30

FASLG 29

HTR2C 29

ABCG1 29

AKR1B1 27

BAK1 27

EIF2S1 27

PSEN2 27

ACHE 26

CTSB 26

ICAM1 26

KCNMA1 26

HTR2B 25

TOP1 25

BCHE 24

CD28 24

IL1A 24

HRH3 24

CDK7 23

HMOX1 22

IDH1 22

REN 22

SLC6A4 22

MMP3 21

SHH 21

MMP7 20

GPX1 19

TNFRSF1B 19

TRPV1 19

PRSS3 18

SCN9A 18

C5AR1 17

DPP4 17

FDFT1 17

GABBR1 17

SLC6A3 17

XDH 17

TNFRSF10B 17

UCP2 16

PDE5A 16

EIF2AK3 16

CCR2 16

CRP 15

TPI1 15

ALDH3A1 14

GPX4 14

MAP2 14

GLB1 13

HMGCR 13

MMP8 13

ODC1 13

PECAM1 13

TACR1 13

TTR 13

EIF2AK1 13

CHRM3 12

GLO1 12

GNRH1 12

MGAM 12

CA2 11

ENPP2 11

TNFRSF10A 11

EIF2AK4 11

ALOX5 10

GPT 10

MMP10 10

TRPV4 9

CD80 8

GM2A 8

MMP12 8

TNFSF10 8

FOLH1 7

FUCA1 7

GJB1 7

DHFR 6

GDNF 6

GCLC 6

LITAF 5

PAM 4

TYR 4

TDP1 4

TMPRSS6 4

CETP 3

CHRNA7 3

PON1 3

C1R 2

PADI4 2

GAMT 1

SI 1

NPC1L1 1
