# Supplementary material for: Study on the Anti-demyelination Mechanism of Bu-Shen-Yi-Sui Capsule in the Central Nervous System Based on Network Pharmacology and Experimental Verification
Source: Mediators Inflamm. 2022 Jul 12;2022:9241261. doi: 10.1155/2022/9241261 (PMC9296285; doi:10.1155/2022/9241261)
Supplement: Supplementary Materials — Table S1: all the potential targets of BSYS Capsule. Table S2: known CNSD-related targets. Table S3: BSYS Capsule shared 227 intersection targets with known CNSD-related targets. Table S4: PPI information of 227 intersection targets in Metascape. Table S5: the degree values of all nodes in the PPI network. Table S6: results for GO pathway enrichment analysis. Table S7: results for KEGG pathway enrichment analysis. Table S8: information of gene-pathway network. Table S9: information of the “active ingredients-intersection targets” network. [file 9241261.f1.zip › Table S6.docx]

Ontology ID Description GeneRatio BgRatio pvalue p.adjust qvalue geneID Count

BP GO:2000377 regulation of reactive oxygen species metabolic process 38/227 200/18866 1.09149E-34 3.28297E-31 1.24012E-31 PTGS2/BCL2/TGFB1/MMP3/EGFR/AKT1/CDKN1A/IL10/TNF/TP53/XDH/SOD1/HIF1A/IL1B/NFE2L2/TLR4/SNCA/HSP90AA1/MMP8/SYK/F2/MAPK14/STAT3/EDN1/FOXO1/ICAM1/BRCA1/CRP/AGTR1/CFLAR/MTOR/IFNG/GSTP1/INSR/TRPV1/ALOX5/BIRC2/DHFR 38

BP GO:0072593 reactive oxygen species metabolic process 43/227 288/18866 1.38114E-34 3.28297E-31 1.24012E-31 PTGS2/BCL2/TGFB1/MMP3/NOS2/EGFR/AKT1/CDKN1A/IL10/TNF/TP53/XDH/SOD1/HIF1A/IL1B/NFE2L2/TLR4/SNCA/HSP90AA1/MMP8/CAT/SYK/F2/MPO/MAPK14/STAT3/EDN1/FOXO1/ICAM1/BRCA1/SOD2/CRP/AGTR1/CFLAR/MTOR/IFNG/GSTP1/INSR/GPX1/TRPV1/ALOX5/BIRC2/DHFR 43

BP GO:0032496 response to lipopolysaccharide 45/227 334/18866 3.52996E-34 5.59381E-31 2.11302E-31 PTGS2/OPRM1/CASP9/CASP3/CASP8/PRKCA/TGFB1/RELA/NOS2/AKT1/FOS/MAPK1/IL10/TNF/IL6/PPARD/IL1B/TLR4/SNCA/MAPK3/MPO/MAPK14/FASLG/MAPK8/EDN1/GJA1/ICAM1/CCL2/SELE/CXCL8/IL1A/CD80/C5AR1/NR1H3/NFKB1/GSTP1/GNRH1/CSF2/LITAF/CASP1/NR1H4/TNFRSF1B/REN/BDKRB1/CCR5 45

BP GO:0062197 cellular response to chemical stress 46/227 360/18866 6.87604E-34 6.53774E-31 2.46959E-31 PTGS2/BCL2/JUN/CASP3/RELA/PPARG/MMP3/EGFR/AKT1/FOS/MMP2/MMP9/MAPK1/IL10/TNF/IL6/TP53/SOD1/HIF1A/HMOX1/NFE2L2/TLR4/DDIT3/SNCA/MAPK3/AKR1B1/CAT/CDK1/MCL1/PARP1/MPO/SIRT1/MAPK8/FOXO1/SOD2/CFLAR/EIF2S1/MDM2/GSTP1/TRPV4/GPX1/ALOX5/EIF2AK3/MAPK10/MAPK9/DHFR 46

BP GO:0070997 neuron death 46/227 360/18866 6.87604E-34 6.53774E-31 2.46959E-31 CHRNA7/BCL2/BAX/CASP9/JUN/CASP3/CASP8/AKT1/BCL2L1/FOS/IL10/TNF/TP53/SOD1/HIF1A/HMOX1/TLR4/DDIT3/GDNF/SNCA/BDNF/NGF/NTRK1/NTRK2/APP/GCLC/MCL1/CDK5/PARP1/FASLG/SIRT1/STAT3/CCL2/SOD2/XIAP/PPARA/C5AR1/MTOR/EIF2S1/CDC42/IFNG/PRKCG/PSEN1/CREB1/ATF2/TNFRSF1B 46

BP GO:1901214 regulation of neuron death 44/227 321/18866 9.59371E-34 7.60141E-31 2.87138E-31 CHRNA7/BCL2/BAX/CASP9/JUN/CASP3/CASP8/AKT1/BCL2L1/FOS/IL10/TNF/TP53/SOD1/HIF1A/HMOX1/TLR4/DDIT3/GDNF/SNCA/BDNF/NGF/NTRK1/NTRK2/GCLC/MCL1/CDK5/PARP1/FASLG/SIRT1/STAT3/CCL2/SOD2/PPARA/C5AR1/MTOR/EIF2S1/CDC42/IFNG/PRKCG/PSEN1/CREB1/ATF2/TNFRSF1B 44

BP GO:0002237 response to molecule of bacterial origin 45/227 356/18866 6.15811E-33 4.18223E-30 1.57981E-30 PTGS2/OPRM1/CASP9/CASP3/CASP8/PRKCA/TGFB1/RELA/NOS2/AKT1/FOS/MAPK1/IL10/TNF/IL6/PPARD/IL1B/TLR4/SNCA/MAPK3/MPO/MAPK14/FASLG/MAPK8/EDN1/GJA1/ICAM1/CCL2/SELE/CXCL8/IL1A/CD80/C5AR1/NR1H3/NFKB1/GSTP1/GNRH1/CSF2/LITAF/CASP1/NR1H4/TNFRSF1B/REN/BDKRB1/CCR5 45

BP GO:0031667 response to nutrient levels 50/227 473/18866 7.65842E-33 4.55101E-30 1.71911E-30 PTGS2/SLC6A4/OPRM1/BCL2/JUN/PON1/RELA/PPARG/EGFR/AKT1/CDKN1A/MAPK1/TP53/SOD1/STAT1/PPARD/GPT/HMOX1/NFE2L2/ATF3/DDIT3/MAPK3/NTRK1/MMP7/CAT/GCLC/TYR/HTR2C/MPO/SIRT1/MAPK8/FOXO1/ICAM1/PPARA/MTOR/EIF2S1/MDM2/GSTP1/ALDH3A1/BCHE/PRKCG/TRPV4/HMGCR/NR1H4/TRPV1/VDR/EIF2AK3/EIF2AK4/EIF2AK2/EIF2AK1 50

BP GO:0006979 response to oxidative stress 48/227 458/18866 2.46243E-31 1.30071E-28 4.91334E-29 PTGS2/BCL2/JUN/CASP3/RELA/MMP3/EGFR/AKT1/FOS/MMP2/MMP9/MAPK1/IL10/TNF/IL6/TP53/SOD1/HIF1A/STAT1/HMOX1/NFE2L2/TLR4/SNCA/MAPK3/APP/CAT/GCLC/CDK1/MCL1/PARP1/MPO/SIRT1/MAPK8/BAK1/EDN1/FOXO1/SOD2/CFLAR/EIF2S1/MDM2/GSTP1/PSEN1/GPX1/GPX4/ALOX5/IDH1/MAPK9/DHFR 48

BP GO:0097191 extrinsic apoptotic signaling pathway 37/227 230/18866 5.29237E-31 2.51599E-28 9.50399E-29 BCL2/BAX/CASP9/CASP3/CASP8/TGFB1/RELA/AR/AKT1/BCL2L1/TNF/IL1B/HMOX1/ATF3/GDNF/NGF/GCLC/MCL1/FASLG/BAK1/ICAM1/BRCA1/IL1A/TNFRSF10B/CFLAR/TNFSF10/TNFRSF10A/IL2/IFNG/IL4/GSTP1/CSF2/GPX1/TNFRSF1A/TNFRSF1B/TGFBR1/RET 37

BP GO:0007568 aging 41/227 319/18866 2.67936E-30 1.15797E-27 4.37415E-28 PTGS2/SLC6A3/HTR2A/BCL2/CASP9/JUN/RELA/AKT1/FOS/CDKN1A/MAPK1/IL10/TP53/SOD1/NFE2L2/MAPK3/NTRK1/APP/MMP7/CAT/GCLC/CDK1/CDK6/MPO/MAPK14/SIRT1/STAT3/BAK1/EDN1/ICAM1/SOD2/MTOR/EIF2S1/GNRH1/ALDH3A1/HMGCR/PSEN1/CREB1/LITAF/GPX4/TNFRSF1B 41

BP GO:2001233 regulation of apoptotic signaling pathway 45/227 413/18866 4.30219E-30 1.70438E-27 6.43818E-28 PTGS2/BCL2/BAX/CASP8/RELA/AR/AKT1/BCL2L1/MMP9/TNF/TP53/SOD1/HIF1A/IL1B/HMOX1/NFE2L2/ATF3/DDIT3/GDNF/BDNF/GCLC/MCL1/PARP1/FASLG/SIRT1/MAPK8/BAK1/ICAM1/BRCA1/SOD2/IL1A/TNFRSF10B/CFLAR/TNFSF10/TNFRSF10A/MDM2/IL4/GSTP1/PSEN1/CSF2/GPX1/EIF2AK3/MAPK9/TGFBR1/RET 45

BP GO:0071496 cellular response to external stimulus 41/227 326/18866 6.46951E-30 2.36585E-27 8.93683E-28 PTGS2/BCL2/JUN/CASP8/PPARG/EGFR/AKT1/FOS/CDKN1A/MAPK1/TP53/IL1B/HMOX1/NFE2L2/TLR4/ATF3/MAPK3/MMP7/GCLC/SIRT1/MAPK8/BAK1/FOXO1/GJA1/ICAM1/VCAM1/TNFRSF10B/PPARA/MTOR/EIF2S1/TNFRSF10A/NFKB1/MDM2/GSTP1/CASP1/NR1H4/TNFRSF1A/VDR/EIF2AK3/EIF2AK4/EIF2AK2 41

BP GO:0050727 regulation of inflammatory response 45/227 425/18866 1.4951E-29 5.07694E-27 1.91778E-27 PTGS2/PIK3CG/RELA/PPARG/MMP3/NOS2/EGFR/MMP9/IL10/TNF/IL6/SOD1/PPARD/ESR1/IL1B/TLR4/SNCA/MMP8/CYP19A1/F2/MAPK14/STAT3/SELE/XIAP/PPARA/AGTR1/IL17B/CCR2/BIRC3/CD28/NR1H3/NFKB1/IL2/IFNG/IL4/GSTP1/TRPV4/CASP1/NR1H4/GPX1/GPX4/TNFRSF1A/ALOX5/TNFRSF1B/BIRC2 45

BP GO:2000379 positive regulation of reactive oxygen species metabolic process 27/227 106/18866 1.56759E-28 4.96822E-26 1.87671E-26 PTGS2/TGFB1/EGFR/AKT1/CDKN1A/TNF/TP53/XDH/SOD1/IL1B/NFE2L2/TLR4/SNCA/HSP90AA1/MMP8/SYK/F2/MAPK14/EDN1/ICAM1/CRP/AGTR1/MTOR/IFNG/GSTP1/INSR/TRPV1 27

BP GO:2001234 negative regulation of apoptotic signaling pathway 35/227 233/18866 2.7517E-28 8.176E-26 3.08842E-26 PTGS2/BCL2/BAX/CASP8/RELA/AR/AKT1/BCL2L1/MMP9/TNF/HIF1A/IL1B/HMOX1/NFE2L2/GDNF/BDNF/GCLC/MCL1/FASLG/SIRT1/ICAM1/BRCA1/SOD2/IL1A/TNFRSF10B/CFLAR/TNFSF10/TNFRSF10A/MDM2/IL4/GSTP1/PSEN1/CSF2/GPX1/TGFBR1 35

BP GO:0042493 response to drug 42/227 397/18866 1.45174E-27 4.05975E-25 1.53354E-25 PTGS2/SLC6A3/HTR2A/SLC6A4/BCL2/CASP3/RELA/PPARG/NOS2/EGFR/FOS/CDKN1A/IL10/TP53/TOP1/SOD1/STAT1/MYC/IL1B/NFE2L2/BDNF/NTRK1/CAT/CDK1/HTR2C/ABCB1/STAT3/BAK1/EDN1/ICAM1/MDM2/ALDH3A1/CYP3A4/NR1I2/BCHE/PAM/CREB1/NPC1L1/REN/DRD2/HTR2B/RET 42

BP GO:0034599 cellular response to oxidative stress 38/227 310/18866 2.51954E-27 6.46189E-25 2.44093E-25 BCL2/JUN/RELA/MMP3/EGFR/AKT1/FOS/MMP2/MMP9/MAPK1/IL10/TNF/IL6/TP53/SOD1/HIF1A/HMOX1/NFE2L2/TLR4/SNCA/MAPK3/CAT/CDK1/MCL1/PARP1/MPO/SIRT1/MAPK8/FOXO1/SOD2/CFLAR/EIF2S1/MDM2/GSTP1/GPX1/ALOX5/MAPK9/DHFR 38

BP GO:0050673 epithelial cell proliferation 44/227 453/18866 2.58258E-27 6.46189E-25 2.44093E-25 PGR/BAX/JUN/PRKCA/TGFB1/AR/PPARG/EGFR/AKT1/VEGFA/MAPK1/IL10/TNF/IL6/XDH/HIF1A/STAT1/KDR/PPARD/ESR1/MYC/HMOX1/TLR4/MMP12/CDK6/SIRT1/STAT3/GJA1/CCL2/AGTR1/CFLAR/C5AR1/MTOR/CDC42/ERBB2/PSEN1/ATF2/SHH/GPX1/ALOX5/CCR3/VDR/TGFBR1/HTR2B 44

BP GO:0000302 response to reactive oxygen species 34/227 235/18866 6.2139E-27 1.47704E-24 5.57943E-25 BCL2/JUN/CASP3/RELA/MMP3/EGFR/AKT1/FOS/MMP2/MMP9/MAPK1/IL10/TNF/IL6/SOD1/STAT1/HMOX1/NFE2L2/MAPK3/CAT/CDK1/MPO/SIRT1/MAPK8/BAK1/EDN1/FOXO1/SOD2/CFLAR/MDM2/GSTP1/GPX1/MAPK9/DHFR 34

BP GO:0048545 response to steroid hormone 39/227 346/18866 1.20029E-26 2.71723E-24 1.02641E-24 PGR/RXRA/PTGS2/BCL2/CASP9/CASP3/TGFB1/RELA/AR/EGFR/FOS/CDKN1A/IL10/TNF/IL6/ESR2/PPARD/ESR1/CA2/PARP1/SIRT1/EDN1/FOXO1/ICAM1/BRCA1/PPARA/CFLAR/NR1H3/MDM2/GSTP1/GNRH1/ALDH3A1/RXRB/BCHE/PAM/RXRG/GLB1/EP300/IDH1 39

BP GO:0050678 regulation of epithelial cell proliferation 40/227 395/18866 1.56822E-25 3.38877E-23 1.28009E-23 PGR/BAX/JUN/PRKCA/TGFB1/AR/PPARG/EGFR/AKT1/VEGFA/IL10/TNF/XDH/HIF1A/STAT1/KDR/PPARD/MYC/HMOX1/TLR4/MMP12/CDK6/SIRT1/STAT3/GJA1/CCL2/AGTR1/CFLAR/C5AR1/MTOR/CDC42/ERBB2/ATF2/SHH/GPX1/ALOX5/CCR3/VDR/TGFBR1/HTR2B 40

BP GO:0001819 positive regulation of cytokine production 42/227 447/18866 1.67977E-25 3.47201E-23 1.31153E-23 PTGS2/CASP8/TGFB1/RELA/IL10/TNF/IL6/SOD1/HIF1A/STAT1/IL1B/TLR4/DDIT3/APP/MMP8/SYK/MAPK14/STAT3/BRCA1/IL1A/CD80/IL17B/C5AR1/CCR2/BIRC3/CD28/NFKB1/IL2/IFNG/IL4/CD40LG/TRPV4/CREB1/ATF2/CSF2/CASP1/EIF2AK3/BIRC2/EP300/DRD2/HTR2B/EIF2AK2 42

BP GO:2001237 negative regulation of extrinsic apoptotic signaling pathway 25/227 107/18866 1.86726E-25 3.69872E-23 1.39717E-23 BCL2/CASP8/RELA/AR/AKT1/BCL2L1/TNF/IL1B/HMOX1/GDNF/GCLC/MCL1/FASLG/ICAM1/BRCA1/IL1A/TNFRSF10B/CFLAR/TNFSF10/TNFRSF10A/IL4/GSTP1/CSF2/GPX1/TGFBR1 25

BP GO:0071216 cellular response to biotic stimulus 33/227 246/18866 4.50517E-25 8.56703E-23 3.23613E-23 PRKCA/TGFB1/RELA/NOS2/AKT1/MAPK1/IL10/TNF/IL6/TP53/PPARD/IL1B/TLR4/DDIT3/MAPK3/SYK/MAPK14/MAPK8/ICAM1/CCL2/CXCL8/IL1A/CD80/NR1H3/NFKB1/GSTP1/CSF2/LITAF/CASP1/NR1H4/TNFRSF1B/EIF2AK3/CCR5 33

BP GO:0010038 response to metal ion 38/227 366/18866 1.15479E-24 2.11149E-22 7.976E-23 PTGS2/SLC6A3/BCL2/CASP9/JUN/CASP3/CASP8/EGFR/AKT1/FOS/MMP9/MAPK1/SOD1/HIF1A/HMOX1/NFE2L2/SNCA/MAPK3/CA2/APP/CAT/GCLC/CDK1/PARP1/MAPK8/EDN1/ICAM1/IL1A/EIF2S1/MDM2/GNRH1/KCNMA1/PAM/CREB1/SHH/EIF2AK3/MAPK9/DRD2 38

BP GO:0043281 regulation of cysteine-type endopeptidase activity involved in apoptotic process 31/227 215/18866 1.44455E-24 2.54348E-22 9.60781E-23 PTGS2/BAX/CASP9/CASP8/PPARG/AKT1/VEGFA/MMP9/TNF/XDH/MYC/SNCA/NGF/SYK/FASLG/SIRT1/BAK1/BIRC5/TNFRSF10B/XIAP/CFLAR/TNFSF10/TNFRSF10A/BIRC3/MDM2/VCP/CASP1/GPX1/EIF2AK3/BIRC2/CTSD 31

BP GO:0071214 cellular response to abiotic stimulus 36/227 331/18866 4.36319E-24 7.15263E-22 2.70185E-22 PTGS2/BAX/CASP9/CASP3/CASP8/EGFR/AKT1/BCL2L1/CDKN1A/TP53/MYC/IL1B/TLR4/MAPK3/AKR1B1/MMP7/GCLC/PARP1/MAPK14/SIRT1/MAPK8/BAK1/GJA1/TNFRSF10B/EIF2S1/TNFRSF10A/NFKB1/MDM2/GRM1/TRPV4/CASP1/TRPV1/TNFRSF1A/EP300/MAPK10/EIF2AK4 36

BP GO:0104004 cellular response to environmental stimulus 36/227 331/18866 4.36319E-24 7.15263E-22 2.70185E-22 PTGS2/BAX/CASP9/CASP3/CASP8/EGFR/AKT1/BCL2L1/CDKN1A/TP53/MYC/IL1B/TLR4/MAPK3/AKR1B1/MMP7/GCLC/PARP1/MAPK14/SIRT1/MAPK8/BAK1/GJA1/TNFRSF10B/EIF2S1/TNFRSF10A/NFKB1/MDM2/GRM1/TRPV4/CASP1/TRPV1/TNFRSF1A/EP300/MAPK10/EIF2AK4 36

BP GO:0033002 muscle cell proliferation 32/227 244/18866 5.08134E-24 8.05223E-22 3.04167E-22 RXRA/PTGS2/JUN/PPARG/EGFR/AKT1/CDKN1A/MMP2/MMP9/MAPK1/IL10/TNF/IL6/STAT1/PPARD/HMOX1/AKR1B1/CDK1/MAPK14/STAT3/EDN1/GJA1/SOD2/CFLAR/MTOR/MDM2/IFNG/GSTP1/HMGCR/SHH/PRKG1/TGFBR1 32

BP GO:0051402 neuron apoptotic process 32/227 245/18866 5.78553E-24 8.8724E-22 3.35148E-22 BCL2/BAX/CASP9/JUN/CASP3/BCL2L1/IL10/TNF/TP53/SOD1/HIF1A/HMOX1/DDIT3/GDNF/BDNF/NGF/NTRK1/NTRK2/APP/GCLC/MCL1/CDK5/PARP1/FASLG/CCL2/SOD2/XIAP/C5AR1/CDC42/PRKCG/PSEN1/ATF2 32

BP GO:0009612 response to mechanical stimulus 30/227 209/18866 9.70244E-24 1.44142E-21 5.44486E-22 PTGS2/HTR2A/JUN/CASP8/RELA/PPARG/EGFR/AKT1/FOS/STAT1/IL1B/TLR4/MAPK3/NTRK1/MMP7/GCLC/MPO/MAPK14/MAPK8/BAK1/EDN1/GJA1/TNFRSF10B/TNFRSF10A/NFKB1/TRPV4/CASP1/TNFRSF1A/BDKRB1/DRD2 30

BP GO:0048608 reproductive structure development 40/227 443/18866 1.20321E-23 1.73335E-21 6.54759E-22 PGR/RXRA/PTGS2/BCL2/BAX/CASP3/CASP8/AR/PPARG/EGFR/AKT1/VEGFA/BCL2L1/MAPK1/IL10/SOD1/HIF1A/PPARD/ESR1/MAPK3/NTRK1/CYP19A1/MAPK14/SIRT1/BAK1/GJA1/ICAM1/TNFSF10/INSR/GNRH1/GJB1/CTSB/CSF2/PTPN6/SHH/VDR/BIRC2/REN/IDH1/TGFBR1 40

BP GO:0061458 reproductive system development 40/227 447/18866 1.68475E-23 2.35567E-21 8.89838E-22 PGR/RXRA/PTGS2/BCL2/BAX/CASP3/CASP8/AR/PPARG/EGFR/AKT1/VEGFA/BCL2L1/MAPK1/IL10/SOD1/HIF1A/PPARD/ESR1/MAPK3/NTRK1/CYP19A1/MAPK14/SIRT1/BAK1/GJA1/ICAM1/TNFSF10/INSR/GNRH1/GJB1/CTSB/CSF2/PTPN6/SHH/VDR/BIRC2/REN/IDH1/TGFBR1 40

BP GO:0071902 positive regulation of protein serine/threonine kinase activity 36/227 345/18866 1.83006E-23 2.48575E-21 9.38974E-22 HTR2A/CHRNA7/PIK3CG/TGFB1/EGFR/AKT1/VEGFA/MAPK1/EGF/TNF/SOD1/IL1B/TLR4/SNCA/MAPK3/NGF/NTRK1/CDK1/SYK/MAPK14/SIRT1/EDN1/C5AR1/ERBB2/IFNG/INSR/CD40LG/GRM1/PSEN1/CSF1R/MAPK10/TGFBR1/PDE5A/HTR2B/EIF2AK2/RET 36

BP GO:0043523 regulation of neuron apoptotic process 30/227 214/18866 1.97683E-23 2.56521E-21 9.6899E-22 BCL2/BAX/CASP9/JUN/CASP3/BCL2L1/IL10/TNF/TP53/SOD1/HIF1A/HMOX1/DDIT3/GDNF/BDNF/NGF/NTRK1/NTRK2/GCLC/MCL1/CDK5/PARP1/FASLG/CCL2/SOD2/C5AR1/CDC42/PRKCG/PSEN1/ATF2 30

BP GO:0070482 response to oxygen levels 38/227 396/18866 1.99648E-23 2.56521E-21 9.6899E-22 PTGS2/PLAU/CHRNA7/SLC6A4/BCL2/CASP3/PPARG/DPP4/NOS2/AKT1/VEGFA/CDKN1A/MMP2/TP53/HIF1A/PPARD/MYC/HMOX1/NFE2L2/CAT/SIRT1/EDN1/FOXO1/ICAM1/PLAT/PPARA/CFLAR/MTOR/MDM2/ALDH3A1/KCNMA1/PSEN2/PAM/CREB1/UCP2/BIRC2/EP300/DRD2 38

BP GO:2001236 regulation of extrinsic apoptotic signaling pathway 27/227 162/18866 3.13535E-23 3.92249E-21 1.48169E-21 BCL2/CASP8/RELA/AR/AKT1/BCL2L1/TNF/IL1B/HMOX1/ATF3/GDNF/GCLC/MCL1/FASLG/ICAM1/BRCA1/IL1A/TNFRSF10B/CFLAR/TNFSF10/TNFRSF10A/IL4/GSTP1/CSF2/GPX1/TGFBR1/RET 27

BP GO:0051090 regulation of DNA-binding transcription factor activity 40/227 455/18866 3.26866E-23 3.98441E-21 1.50508E-21 JUN/RELA/AR/PPARG/AKT1/VEGFA/FOS/MAPK1/IL10/TNF/IL6/ESR2/ESR1/IL1B/HMOX1/TLR4/DDIT3/MAPK3/NTRK1/APP/CAT/SYK/MAPK14/SIRT1/STAT3/MAPK8/EDN1/ICAM1/PRKCB/CFLAR/NFKB1/CD40LG/ATF2/NR1H4/SHH/EP300/MAPK10/MAPK9/EIF2AK4/EIF2AK2 40

BP GO:2000116 regulation of cysteine-type endopeptidase activity 31/227 239/18866 3.81742E-23 4.537E-21 1.71382E-21 PTGS2/BAX/CASP9/CASP8/PPARG/AKT1/VEGFA/MMP9/TNF/XDH/MYC/SNCA/NGF/SYK/FASLG/SIRT1/BAK1/BIRC5/TNFRSF10B/XIAP/CFLAR/TNFSF10/TNFRSF10A/BIRC3/MDM2/VCP/CASP1/GPX1/EIF2AK3/BIRC2/CTSD 31

BP GO:0071222 cellular response to lipopolysaccharide 29/227 208/18866 1.31568E-22 1.52554E-20 5.76263E-21 PRKCA/TGFB1/RELA/NOS2/AKT1/MAPK1/IL10/TNF/IL6/PPARD/IL1B/TLR4/MAPK3/MAPK14/MAPK8/ICAM1/CCL2/CXCL8/IL1A/CD80/NR1H3/NFKB1/GSTP1/CSF2/LITAF/CASP1/NR1H4/TNFRSF1B/CCR5 29

BP GO:0062013 positive regulation of small molecule metabolic process 26/227 154/18866 1.48852E-22 1.68157E-20 6.35202E-21 PTGS2/HTR2A/PPARG/NOS2/AKT1/EGF/TNF/HIF1A/PPARD/IL1B/GPT/SNCA/APP/SIRT1/STAT3/FOXO1/PPARA/NR1H3/NFKB1/IFNG/IL4/INSR/VCP/PSEN1/NR1H4/ABCG1 26

BP GO:0043405 regulation of MAP kinase activity 35/227 342/18866 1.52098E-22 1.68157E-20 6.35202E-21 HTR2A/CHRNA7/PIK3CG/TGFB1/EGFR/VEGFA/MAPK1/EGF/TNF/SOD1/IL1B/TLR4/MAPK3/NGF/NTRK1/CDK1/SYK/MAPK14/IGF1R/EDN1/C5AR1/ERBB2/GSTP1/INSR/CD40LG/GRM1/HMGCR/PSEN1/PTPN6/MAPK10/TGFBR1/PDE5A/HTR2B/EIF2AK2/RET 35

BP GO:0018108 peptidyl-tyrosine phosphorylation 36/227 374/18866 2.90751E-22 3.14144E-20 1.18666E-20 HTR2A/TGFB1/EGFR/VEGFA/EGF/TNF/IL6/TP53/KDR/BDNF/MAPK3/NTRK1/NTRK2/APP/SYK/STAT3/IGF1R/ICAM1/PECAM1/CD80/JAK1/MTOR/ERBB2/IL2/IFNG/IL4/INSR/PSEN1/ENPP2/CSF2/PTPN6/TNFRSF1A/CSF1R/TYK2/EIF2AK2/RET 36

BP GO:0018212 peptidyl-tyrosine modification 36/227 377/18866 3.81533E-22 4.03069E-20 1.52256E-20 HTR2A/TGFB1/EGFR/VEGFA/EGF/TNF/IL6/TP53/KDR/BDNF/MAPK3/NTRK1/NTRK2/APP/SYK/STAT3/IGF1R/ICAM1/PECAM1/CD80/JAK1/MTOR/ERBB2/IL2/IFNG/IL4/INSR/PSEN1/ENPP2/CSF2/PTPN6/TNFRSF1A/CSF1R/TYK2/EIF2AK2/RET 36

BP GO:0018209 peptidyl-serine modification 34/227 333/18866 6.95152E-22 7.18424E-20 2.7138E-20 PTGS2/BCL2/BAX/PRKCA/TGFB1/EGFR/AKT1/VEGFA/MAPK1/TNF/IL6/TOP1/SNCA/BDNF/MAPK3/NGF/NTRK2/HSP90AA1/APP/CDK1/CDK5/SYK/PARP1/MAPK14/MAPK8/BAK1/PRKCB/MTOR/IFNG/PRKCG/EIF2AK3/MAPK9/TGFBR1/RET 34

BP GO:0001666 response to hypoxia 35/227 359/18866 7.64303E-22 7.65895E-20 2.89311E-20 PTGS2/PLAU/CHRNA7/SLC6A4/BCL2/CASP3/DPP4/NOS2/AKT1/VEGFA/MMP2/TP53/HIF1A/PPARD/MYC/HMOX1/NFE2L2/CAT/SIRT1/EDN1/ICAM1/PLAT/PPARA/CFLAR/MTOR/MDM2/ALDH3A1/KCNMA1/PSEN2/PAM/CREB1/UCP2/BIRC2/EP300/DRD2 35

BP GO:0018105 peptidyl-serine phosphorylation 33/227 310/18866 7.8473E-22 7.65895E-20 2.89311E-20 PTGS2/BCL2/BAX/PRKCA/TGFB1/EGFR/AKT1/VEGFA/MAPK1/TNF/IL6/TOP1/SNCA/BDNF/MAPK3/NGF/NTRK2/HSP90AA1/APP/CDK1/CDK5/SYK/MAPK14/MAPK8/BAK1/PRKCB/MTOR/IFNG/PRKCG/EIF2AK3/MAPK9/TGFBR1/RET 33

BP GO:0043406 positive regulation of MAP kinase activity 31/227 264/18866 7.89416E-22 7.65895E-20 2.89311E-20 HTR2A/CHRNA7/PIK3CG/TGFB1/EGFR/VEGFA/MAPK1/EGF/TNF/SOD1/IL1B/TLR4/MAPK3/NGF/NTRK1/CDK1/SYK/MAPK14/EDN1/C5AR1/ERBB2/INSR/CD40LG/GRM1/PSEN1/MAPK10/TGFBR1/PDE5A/HTR2B/EIF2AK2/RET 31

BP GO:0071219 cellular response to molecule of bacterial origin 29/227 222/18866 8.62408E-22 8.19977E-20 3.09741E-20 PRKCA/TGFB1/RELA/NOS2/AKT1/MAPK1/IL10/TNF/IL6/PPARD/IL1B/TLR4/MAPK3/MAPK14/MAPK8/ICAM1/CCL2/CXCL8/IL1A/CD80/NR1H3/NFKB1/GSTP1/CSF2/LITAF/CASP1/NR1H4/TNFRSF1B/CCR5 29

BP GO:0045862 positive regulation of proteolysis 35/227 370/18866 2.07298E-21 1.93234E-19 7.29928E-20 BAX/CASP9/CASP8/PPARG/AKT1/EGF/TNF/XDH/MYC/IL1B/NFE2L2/SNCA/MAPK3/NGF/APP/GCLC/SYK/MAPK14/FASLG/SIRT1/STAT3/BAK1/TNFRSF10B/CFLAR/TNFSF10/TNFRSF10A/MDM2/IFNG/VCP/PSEN1/CASP1/TNFRSF1B/EIF2AK3/CTSD/MAPK9 35

BP GO:0036293 response to decreased oxygen levels 35/227 371/18866 2.26589E-21 2.07155E-19 7.82512E-20 PTGS2/PLAU/CHRNA7/SLC6A4/BCL2/CASP3/DPP4/NOS2/AKT1/VEGFA/MMP2/TP53/HIF1A/PPARD/MYC/HMOX1/NFE2L2/CAT/SIRT1/EDN1/ICAM1/PLAT/PPARA/CFLAR/MTOR/MDM2/ALDH3A1/KCNMA1/PSEN2/PAM/CREB1/UCP2/BIRC2/EP300/DRD2 35

BP GO:0003018 vascular process in circulatory system 27/227 190/18866 2.47968E-21 2.22423E-19 8.40187E-20 PTGS2/CHRM3/HTR2A/SLC6A4/TGFB1/EGFR/AKT1/VEGFA/SOD1/PPARD/HMOX1/GCLC/ABCG2/ABCB1/EDN1/GJA1/ICAM1/SOD2/CRP/AGTR1/KCNMA1/TRPV4/HMGCR/GPX1/PRKG1/HTR1A/HTR2B 27

BP GO:0002573 myeloid leukocyte differentiation 28/227 210/18866 2.57423E-21 2.26627E-19 8.56068E-20 JUN/CASP8/PRKCA/TGFB1/PPARG/VEGFA/FOS/MMP9/TNF/MYC/TLR4/CA2/APP/GLO1/PARP1/CDK6/MAPK14/SIRT1/MTOR/CDC42/IFNG/IL4/PSEN1/CREB1/CSF2/CCR1/CSF1R/EIF2AK1 28

BP GO:0070371 ERK1 and ERK2 cascade 33/227 325/18866 3.48617E-21 3.01332E-19 1.13826E-19 HTR2A/CHRNA7/OPRM1/JUN/PRKCA/TGFB1/EGFR/MAPK1/EGF/TNF/KDR/MYC/IL1B/TLR4/ATF3/MAPK3/NTRK1/APP/HTR2C/SYK/ICAM1/CCL2/CFLAR/C5AR1/ERBB2/GSTP1/TRPV4/HMGCR/PTPN6/CCR1/CSF1R/DRD2/HTR2B 33

BP GO:0048732 gland development 37/227 443/18866 9.75659E-21 8.28265E-19 3.12871E-19 PGR/RXRA/SLC6A3/BCL2/BAX/JUN/TGFB1/RELA/AR/EGFR/AKT1/VEGFA/MAPK1/IL10/EGF/TNF/IL6/XDH/SOD1/HIF1A/ESR1/HMOX1/MAPK3/TYR/CYP19A1/CFLAR/CDC42/INSR/PSEN1/PAM/CREB1/SHH/GPX1/CSF1R/VDR/DRD2/TGFBR1 37

BP GO:0009314 response to radiation 37/227 447/18866 1.32573E-20 1.10571E-18 4.17672E-19 PTGS2/OPRM1/BCL2/BAX/CASP9/CASP3/RELA/EGFR/AKT1/BCL2L1/FOS/CDKN1A/TP53/HIF1A/MYC/NTRK1/APP/CAT/TYR/CDK5/PARP1/MAPK14/SIRT1/MAPK8/BAK1/ICAM1/BRCA1/MTOR/EIF2S1/MDM2/HMGCR/CREB1/GPX1/EP300/MAPK10/DRD2/EIF2AK4 37

BP GO:0062012 regulation of small molecule metabolic process 37/227 456/18866 2.61197E-20 2.14092E-18 8.08716E-19 PTGS2/HTR2A/TGFB1/PPARG/NOS2/AKT1/EGF/TNF/TP53/ODC1/SOD1/HIF1A/PPARD/IL1B/GPT/SNCA/APP/PARP1/SIRT1/STAT3/FOXO1/BRCA1/PPARA/MTOR/NR1H3/NFKB1/IFNG/IL4/INSR/VCP/HMGCR/PSEN1/NR1H4/ABCG1/FDFT1/VDR/EP300 37

BP GO:0034614 cellular response to reactive oxygen species 25/227 170/18866 3.38847E-20 2.7139E-18 1.02516E-18 JUN/RELA/MMP3/EGFR/AKT1/FOS/MMP2/MMP9/MAPK1/IL10/TNF/IL6/SOD1/NFE2L2/MAPK3/CDK1/MPO/SIRT1/MAPK8/FOXO1/SOD2/CFLAR/MDM2/MAPK9/DHFR 25

BP GO:0030099 myeloid cell differentiation 36/227 431/18866 3.4252E-20 2.7139E-18 1.02516E-18 CASP9/JUN/CASP3/CASP8/PRKCA/TGFB1/PPARG/VEGFA/FOS/MMP9/TNF/HIF1A/STAT1/MYC/TLR4/CA2/APP/GLO1/PARP1/CDK6/MAPK14/SIRT1/STAT3/PRKCB/MTOR/CDC42/IFNG/IL4/PSEN1/CREB1/CSF2/PTPN6/CCR1/CSF1R/EP300/EIF2AK1 36

BP GO:1901215 negative regulation of neuron death 27/227 212/18866 4.69435E-20 3.65852E-18 1.38198E-18 BCL2/BAX/JUN/AKT1/BCL2L1/IL10/SOD1/HIF1A/HMOX1/GDNF/SNCA/BDNF/NGF/NTRK1/NTRK2/GCLC/CDK5/SIRT1/STAT3/CCL2/SOD2/PPARA/C5AR1/PRKCG/PSEN1/CREB1/TNFRSF1B 27

BP GO:0051222 positive regulation of protein transport 33/227 354/18866 5.02209E-20 3.85081E-18 1.45461E-18 PTGS2/BCL2/CASP8/TGFB1/ACHE/EGFR/MAPK1/IL10/TNF/IL6/TP53/HIF1A/PPARD/IL1B/TLR4/CDK1/CDK5/SYK/MAPK14/MAPK8/GJA1/IL1A/CDC42/MDM2/ERBB2/IL2/IFNG/PSEN1/NR1H4/SHH/ABCG1/DRD2/HTR2B 33

BP GO:0048511 rhythmic process 31/227 305/18866 5.88109E-20 4.43789E-18 1.67638E-18 PGR/SLC6A4/OPRM1/JUN/CASP3/PPARG/NOS2/EGFR/TP53/TOP1/ESR1/BDNF/NTRK1/NTRK2/MMP7/CDK1/CDK5/SIRT1/MAPK8/PPARA/MTOR/NR1H3/GNRH1/PRKCG/PAM/CREB1/CSF2/EP300/MAPK10/MAPK9/DRD2 31

BP GO:0022407 regulation of cell-cell adhesion 36/227 439/18866 6.30082E-20 4.68033E-18 1.76796E-18 CASP3/PRKCA/TGFB1/RELA/DPP4/AKT1/VEGFA/IL10/TNF/IL6/IL1B/SYK/MAPK14/ICAM1/CCL2/SELE/VCAM1/IL1A/PPARA/CD80/JAK1/CCR2/CDC42/CD28/ERBB2/IL2/IFNG/IL4/CD40LG/GNRH1/TRPV4/PTPN6/SHH/ALOX5/PRKG1/PDE5A 36

BP GO:0042063 gliogenesis 31/227 307/18866 7.13141E-20 5.2158E-18 1.97023E-18 TGFB1/RELA/PPARG/EGFR/AKT1/MAPK1/TNF/IL6/SOD1/IL1B/TLR4/MAPK3/NTRK2/APP/CDK1/CDK5/CDK6/F2/STAT3/CCL2/C5AR1/MTOR/CCR2/ERBB2/IFNG/GSTP1/PSEN1/CREB1/SHH/TNFRSF1B/CSF1R 31

BP GO:1904951 positive regulation of establishment of protein localization 33/227 370/18866 1.96537E-19 1.41566E-17 5.34756E-18 PTGS2/BCL2/CASP8/TGFB1/ACHE/EGFR/MAPK1/IL10/TNF/IL6/TP53/HIF1A/PPARD/IL1B/TLR4/CDK1/CDK5/SYK/MAPK14/MAPK8/GJA1/IL1A/CDC42/MDM2/ERBB2/IL2/IFNG/PSEN1/NR1H4/SHH/ABCG1/DRD2/HTR2B 33

BP GO:1901216 positive regulation of neuron death 20/227 97/18866 2.08932E-19 1.48248E-17 5.59998E-18 BAX/CASP9/JUN/CASP3/CASP8/FOS/TNF/TP53/TLR4/DDIT3/SNCA/MCL1/CDK5/PARP1/FASLG/MTOR/EIF2S1/CDC42/IFNG/ATF2 20

BP GO:0009416 response to light stimulus 31/227 319/18866 2.2002E-19 1.5382E-17 5.81044E-18 PTGS2/BCL2/BAX/CASP9/CASP3/RELA/EGFR/AKT1/FOS/CDKN1A/TP53/HIF1A/MYC/APP/CAT/TYR/CDK5/PARP1/SIRT1/MAPK8/BAK1/MTOR/EIF2S1/MDM2/HMGCR/CREB1/GPX1/EP300/MAPK10/DRD2/EIF2AK4 31

BP GO:0010001 glial cell differentiation 27/227 230/18866 4.04917E-19 2.78982E-17 1.05383E-17 TGFB1/RELA/PPARG/EGFR/AKT1/MAPK1/TNF/IL6/SOD1/IL1B/TLR4/MAPK3/NTRK2/APP/CDK1/CDK5/CDK6/F2/STAT3/C5AR1/MTOR/ERBB2/IFNG/GSTP1/PSEN1/SHH/TNFRSF1B 27

BP GO:0050890 cognition 30/227 302/18866 4.68632E-19 3.18268E-17 1.20223E-17 PTGS2/HTR2A/CHRNA7/SLC6A4/JUN/CASP3/EGFR/FOS/MAPK1/TNF/HIF1A/BDNF/NGF/NTRK1/NTRK2/APP/CDK5/C5AR1/MTOR/INSR/BCHE/GM2A/PRKCG/HMGCR/PSEN1/CREB1/HRH3/EP300/DRD2/EIF2AK4 30

BP GO:0009266 response to temperature stimulus 27/227 233/18866 5.68934E-19 3.80946E-17 1.43899E-17 PTGS2/HTR2A/CASP8/PPARG/AKT1/FOS/CDKN1A/MAPK1/SOD1/HMOX1/MAPK3/NTRK1/HSP90AA1/GCLC/SIRT1/FOXO1/IL1A/MTOR/EIF2S1/VCP/TRPV4/TRPV1/UCP2/EIF2AK3/EP300/HTR2B/EIF2AK4 27

BP GO:0070372 regulation of ERK1 and ERK2 cascade 30/227 306/18866 6.81E-19 4.49649E-17 1.69852E-17 HTR2A/CHRNA7/OPRM1/JUN/PRKCA/TGFB1/EGFR/TNF/KDR/IL1B/TLR4/ATF3/MAPK3/NTRK1/APP/HTR2C/SYK/ICAM1/CCL2/CFLAR/C5AR1/ERBB2/GSTP1/TRPV4/HMGCR/PTPN6/CCR1/CSF1R/DRD2/HTR2B 30

BP GO:0048660 regulation of smooth muscle cell proliferation 24/227 173/18866 8.06835E-19 5.25437E-17 1.9848E-17 PTGS2/JUN/PPARG/EGFR/AKT1/CDKN1A/MMP2/MMP9/IL10/TNF/IL6/STAT1/PPARD/HMOX1/AKR1B1/EDN1/GJA1/SOD2/MTOR/MDM2/IFNG/GSTP1/HMGCR/PRKG1 24

BP GO:0043434 response to peptide hormone 35/227 447/18866 9.53494E-19 6.12555E-17 2.31388E-17 PTGS2/RELA/PPARG/AKT1/IL10/STAT1/IL1B/GPT/NFE2L2/CA2/CAT/GCLC/PARP1/SIRT1/STAT3/IGF1R/EDN1/FOXO1/GJA1/ICAM1/PRKCB/PPARA/AGTR1/CFLAR/MTOR/TNFSF10/NFKB1/MDM2/GSTP1/INSR/GNRH1/TRPV4/CREB1/NR1H4/TRPV1 35

BP GO:0048659 smooth muscle cell proliferation 24/227 175/18866 1.06225E-18 6.73327E-17 2.54344E-17 PTGS2/JUN/PPARG/EGFR/AKT1/CDKN1A/MMP2/MMP9/IL10/TNF/IL6/STAT1/PPARD/HMOX1/AKR1B1/EDN1/GJA1/SOD2/MTOR/MDM2/IFNG/GSTP1/HMGCR/PRKG1 24

BP GO:0070663 regulation of leukocyte proliferation 27/227 240/18866 1.23387E-18 7.71818E-17 2.91549E-17 BCL2/CASP3/CDKN1A/MAPK1/IL10/IL6/IL1B/TLR4/MAPK3/SYK/VCAM1/IL1A/CRP/CD80/CCR2/CD28/ERBB2/IL2/IL4/GSTP1/CD40LG/GNRH1/PTPN6/SHH/TNFRSF1B/CSF1R/PDE5A 27

BP GO:0070661 leukocyte proliferation 30/227 313/18866 1.29215E-18 7.97778E-17 3.01355E-17 PIK3CG/BCL2/BAX/CASP3/CDKN1A/MAPK1/IL10/IL6/TP53/IL1B/TLR4/MAPK3/SYK/VCAM1/IL1A/CRP/CD80/CCR2/CD28/ERBB2/IL2/IL4/GSTP1/CD40LG/GNRH1/PTPN6/SHH/TNFRSF1B/CSF1R/PDE5A 30

BP GO:0042110 T cell activation 36/227 483/18866 1.44395E-18 8.80066E-17 3.32439E-17 PIK3CG/BCL2/BAX/CASP3/CASP8/DPP4/AKT1/IL10/IL6/TP53/SOD1/IL1B/SYK/CDK6/STAT3/ICAM1/CCL2/VCAM1/IL1A/CD80/MTOR/CCR2/CDC42/CD28/ERBB2/IL2/IFNG/IL4/CD40LG/GNRH1/PSEN1/PTPN6/SHH/TNFRSF1B/PDE5A/EIF2AK4 36

BP GO:0009411 response to UV 22/227 140/18866 1.53815E-18 9.25616E-17 3.49645E-17 PTGS2/BCL2/BAX/CASP9/CASP3/RELA/EGFR/AKT1/CDKN1A/TP53/MYC/CAT/TYR/PARP1/SIRT1/MAPK8/BAK1/EIF2S1/MDM2/GPX1/EP300/EIF2AK4 22

BP GO:0097193 intrinsic apoptotic signaling pathway 29/227 290/18866 1.57889E-18 9.38253E-17 3.54418E-17 PTGS2/BCL2/BAX/CASP9/CASP3/AKT1/BCL2L1/CDKN1A/MMP9/TNF/TP53/SOD1/HIF1A/HMOX1/NFE2L2/DDIT3/MCL1/PARP1/SIRT1/BAK1/BRCA1/SOD2/TNFRSF10B/MDM2/GPX1/TNFRSF1A/TNFRSF1B/EIF2AK3/EP300 29

BP GO:1901653 cellular response to peptide 33/227 398/18866 1.82452E-18 1.07083E-16 4.045E-17 RELA/PPARG/AKT1/TP53/STAT1/IL1B/GPT/NFE2L2/TLR4/CA2/AKR1B1/APP/GCLC/CDK5/PARP1/SIRT1/STAT3/IGF1R/EDN1/FOXO1/GJA1/ICAM1/VCAM1/PRKCB/AGTR1/CFLAR/NFKB1/MDM2/GSTP1/INSR/PSEN1/CREB1/NR1H4 33

BP GO:0010952 positive regulation of peptidase activity 25/227 201/18866 2.17895E-18 1.26326E-16 4.77187E-17 BAX/CASP9/CASP8/PPARG/TNF/XDH/MYC/SNCA/MAPK3/NGF/APP/SYK/MAPK14/FASLG/SIRT1/STAT3/BAK1/TNFRSF10B/CFLAR/TNFSF10/TNFRSF10A/VCP/CASP1/EIF2AK3/CTSD 25

BP GO:0034612 response to tumor necrosis factor 30/227 320/18866 2.4115E-18 1.38124E-16 5.21752E-17 PTGS2/CASP3/CASP8/RELA/AKT1/MAPK1/TNF/TP53/STAT1/NFE2L2/MAPK3/SYK/MAPK14/SIRT1/EDN1/ICAM1/CCL2/SELE/CXCL8/BRCA1/BIRC3/NFKB1/GSTP1/CD40LG/CASP1/NR1H4/TRPV1/TNFRSF1A/TNFRSF1B/BIRC2 30

BP GO:0035296 regulation of tube diameter 22/227 143/18866 2.46974E-18 1.38131E-16 5.2178E-17 PTGS2/CHRM3/HTR2A/SLC6A4/EGFR/AKT1/SOD1/PPARD/HMOX1/GCLC/EDN1/GJA1/ICAM1/SOD2/CRP/AGTR1/KCNMA1/HMGCR/GPX1/PRKG1/HTR1A/HTR2B 22

BP GO:0097746 regulation of blood vessel diameter 22/227 143/18866 2.46974E-18 1.38131E-16 5.2178E-17 PTGS2/CHRM3/HTR2A/SLC6A4/EGFR/AKT1/SOD1/PPARD/HMOX1/GCLC/EDN1/GJA1/ICAM1/SOD2/CRP/AGTR1/KCNMA1/HMGCR/GPX1/PRKG1/HTR1A/HTR2B 22

BP GO:0035150 regulation of tube size 22/227 144/18866 2.88466E-18 1.59461E-16 6.02354E-17 PTGS2/CHRM3/HTR2A/SLC6A4/EGFR/AKT1/SOD1/PPARD/HMOX1/GCLC/EDN1/GJA1/ICAM1/SOD2/CRP/AGTR1/KCNMA1/HMGCR/GPX1/PRKG1/HTR1A/HTR2B 22

BP GO:0044706 multi-multicellular organism process 26/227 226/18866 3.17346E-18 1.73409E-16 6.55042E-17 PGR/RXRA/PTGS2/SLC6A4/BCL2/AR/AKT1/VEGFA/FOS/MMP2/MMP9/MAPK1/SOD1/PPARD/ESR1/IL1B/MAPK3/AKR1B1/MMP7/EDN1/GJA1/MTOR/GNRH1/PAM/CTSB/VDR 26

BP GO:0052547 regulation of peptidase activity 35/227 466/18866 3.57415E-18 1.93085E-16 7.29366E-17 PTGS2/BAX/CASP9/CASP8/PPARG/AKT1/VEGFA/MMP9/TNF/XDH/MYC/SNCA/MAPK3/NGF/APP/SYK/MAPK14/FASLG/SIRT1/STAT3/BAK1/BIRC5/TNFRSF10B/XIAP/CFLAR/TNFSF10/TNFRSF10A/BIRC3/MDM2/VCP/CASP1/GPX1/EIF2AK3/BIRC2/CTSD 35

BP GO:1903409 reactive oxygen species biosynthetic process 21/227 128/18866 3.79001E-18 2.02446E-16 7.64727E-17 PTGS2/NOS2/AKT1/IL10/TNF/SOD1/IL1B/TLR4/SNCA/HSP90AA1/MMP8/MPO/STAT3/EDN1/ICAM1/CFLAR/MTOR/IFNG/INSR/TRPV1/ALOX5 21

BP GO:0032147 activation of protein kinase activity 30/227 331/18866 6.22795E-18 3.28974E-16 1.24268E-16 CHRNA7/EGFR/AKT1/VEGFA/MAPK1/EGF/TNF/SOD1/IL1B/TLR4/MAPK3/NGF/NTRK1/CDK1/SYK/MAPK14/TNFRSF10B/C5AR1/MTOR/TNFRSF10A/IL4/INSR/CD40LG/GRM1/PSEN1/MAPK10/DRD2/TGFBR1/EIF2AK2/RET 30

BP GO:0050679 positive regulation of epithelial cell proliferation 25/227 211/18866 7.13705E-18 3.72852E-16 1.40842E-16 JUN/PRKCA/AR/EGFR/AKT1/VEGFA/IL10/HIF1A/KDR/MYC/HMOX1/TLR4/MMP12/SIRT1/STAT3/AGTR1/CFLAR/C5AR1/MTOR/CDC42/ERBB2/SHH/CCR3/TGFBR1/HTR2B 25

BP GO:0097305 response to alcohol 26/227 234/18866 7.60777E-18 3.93123E-16 1.485E-16 SLC6A3/OPRM1/CASP8/TGFB1/PPARG/AKT1/BCL2L1/FOS/CDKN1A/SOD1/CAT/CDK1/PARP1/STAT3/BAK1/ICAM1/BRCA1/IL2/GSTP1/GNRH1/HMGCR/GLB1/BIRC2/CCR5/DRD2/TGFBR1 26

BP GO:0007611 learning or memory 27/227 260/18866 9.82529E-18 5.02252E-16 1.89722E-16 PTGS2/HTR2A/CHRNA7/SLC6A4/JUN/CASP3/EGFR/FOS/MAPK1/HIF1A/BDNF/NGF/NTRK1/NTRK2/APP/CDK5/MTOR/INSR/BCHE/GM2A/PRKCG/HMGCR/PSEN1/CREB1/EP300/DRD2/EIF2AK4 27

BP GO:0043280 positive regulation of cysteine-type endopeptidase activity involved in apoptotic process 21/227 134/18866 1.01087E-17 5.11244E-16 1.93119E-16 BAX/CASP9/CASP8/PPARG/TNF/XDH/MYC/SNCA/NGF/SYK/FASLG/SIRT1/BAK1/TNFRSF10B/CFLAR/TNFSF10/TNFRSF10A/VCP/CASP1/EIF2AK3/CTSD 21

BP GO:0006874 cellular calcium ion homeostasis 34/227 456/18866 1.41449E-17 7.07842E-16 2.67382E-16 HTR2A/CHRNA7/PIK3CG/BCL2/BAX/ESR1/DDIT3/SNCA/APP/HTR2C/CDK5/F2/FASLG/BAK1/EDN1/GJA1/PRKCB/AGTR1/C5AR1/CCR2/IL2/GRM1/TRPV4/PSEN1/PTPN6/TRPV1/CCR1/CCR3/VDR/TACR1/BDKRB1/CCR5/DRD2/HTR2B 34

BP GO:0007565 female pregnancy 24/227 196/18866 1.55739E-17 7.71231E-16 2.91327E-16 PGR/RXRA/PTGS2/BCL2/AR/AKT1/VEGFA/FOS/MMP2/MMP9/MAPK1/SOD1/PPARD/ESR1/IL1B/MAPK3/AKR1B1/MMP7/GJA1/MTOR/GNRH1/PAM/CTSB/VDR 24

BP GO:0052548 regulation of endopeptidase activity 33/227 434/18866 2.47146E-17 1.21127E-15 4.57549E-16 PTGS2/BAX/CASP9/CASP8/PPARG/AKT1/VEGFA/MMP9/TNF/XDH/MYC/SNCA/NGF/APP/SYK/FASLG/SIRT1/STAT3/BAK1/BIRC5/TNFRSF10B/XIAP/CFLAR/TNFSF10/TNFRSF10A/BIRC3/MDM2/VCP/CASP1/GPX1/EIF2AK3/BIRC2/CTSD 33

BP GO:0051091 positive regulation of DNA-binding transcription factor activity 27/227 270/18866 2.58872E-17 1.25579E-15 4.74366E-16 RELA/AR/PPARG/AKT1/VEGFA/IL10/TNF/IL6/ESR2/ESR1/IL1B/TLR4/DDIT3/NTRK1/APP/CAT/STAT3/EDN1/ICAM1/PRKCB/CFLAR/NFKB1/CD40LG/ATF2/SHH/EP300/EIF2AK2 27

BP GO:0007204 positive regulation of cytosolic calcium ion concentration 29/227 322/18866 2.73922E-17 1.31538E-15 4.96875E-16 HTR2A/PIK3CG/BCL2/BAX/ESR1/DDIT3/SNCA/HTR2C/CDK5/F2/FASLG/BAK1/EDN1/GJA1/AGTR1/C5AR1/CCR2/IL2/GRM1/TRPV4/PTPN6/TRPV1/CCR1/CCR3/TACR1/BDKRB1/CCR5/DRD2/HTR2B 29

BP GO:1903706 regulation of hemopoiesis 35/227 498/18866 2.87721E-17 1.36782E-15 5.16686E-16 JUN/CASP8/PRKCA/TGFB1/FOS/TNF/SOD1/HIF1A/STAT1/MYC/IL1B/NFE2L2/TLR4/CA2/SYK/CDK6/MAPK14/STAT3/PRKCB/IL1A/CD80/MTOR/CCR2/CD28/ERBB2/IL2/IFNG/IL4/CREB1/CSF2/PTPN6/SHH/CCR1/EP300/EIF2AK2 35

BP GO:0055074 calcium ion homeostasis 34/227 468/18866 3.12933E-17 1.47295E-15 5.56397E-16 HTR2A/CHRNA7/PIK3CG/BCL2/BAX/ESR1/DDIT3/SNCA/APP/HTR2C/CDK5/F2/FASLG/BAK1/EDN1/GJA1/PRKCB/AGTR1/C5AR1/CCR2/IL2/GRM1/TRPV4/PSEN1/PTPN6/TRPV1/CCR1/CCR3/VDR/TACR1/BDKRB1/CCR5/DRD2/HTR2B 34

BP GO:0051235 maintenance of location 29/227 324/18866 3.23767E-17 1.50901E-15 5.70016E-16 HTR2A/BAX/PPARG/AKT1/IL10/TNF/IL6/PPARD/IL1B/DDIT3/SNCA/HTR2C/CDK5/F2/FASLG/SIRT1/GJA1/PPARA/CRP/NR1H3/NFKB1/GM2A/PTPN6/TRPV1/ABCG1/BDKRB1/CCR5/DRD2/HTR2B 29

BP GO:0150076 neuroinflammatory response 17/227 77/18866 3.28985E-17 1.51835E-15 5.73546E-16 PTGS2/JUN/MMP3/EGFR/MMP9/TNF/IL6/IL1B/SNCA/APP/MMP8/C5AR1/IFNG/IL4/PSEN1/TRPV1/TNFRSF1B 17

BP GO:0060135 maternal process involved in female pregnancy 16/227 64/18866 3.32159E-17 1.51835E-15 5.73546E-16 PGR/RXRA/PTGS2/AR/AKT1/MAPK1/PPARD/ESR1/MAPK3/AKR1B1/MMP7/GJA1/MTOR/PAM/CTSB/VDR 16

BP GO:0031098 stress-activated protein kinase signaling cascade 28/227 300/18866 3.95589E-17 1.79107E-15 6.76566E-16 EGFR/AKT1/VEGFA/MAPK1/TNF/XDH/MYC/IL1B/TLR4/MAPK3/AKR1B1/APP/MMP8/SYK/MAPK14/MAPK8/IGF1R/EDN1/FOXO1/CDC42/NFKB1/GSTP1/CD40LG/TRPV4/HMGCR/MAPK10/MAPK9/EIF2AK2 28

BP GO:1901342 regulation of vasculature development 33/227 444/18866 4.87038E-17 2.17337E-15 8.20975E-16 PTGS2/CHRNA7/PRKCA/PPARG/VEGFA/IL10/TNF/IL6/XDH/HIF1A/STAT1/KDR/IL1B/HMOX1/NFE2L2/FASLG/SIRT1/STAT3/CXCL8/BRCA1/SOD2/PRKCB/IL1A/JAK1/AGTR1/CFLAR/C5AR1/CCR2/ERBB2/ENPP2/ATF2/ALOX5/CCR3 33

BP GO:1903037 regulation of leukocyte cell-cell adhesion 29/227 329/18866 4.89168E-17 2.17337E-15 8.20975E-16 CASP3/RELA/DPP4/AKT1/IL10/TNF/IL6/IL1B/SYK/ICAM1/CCL2/SELE/VCAM1/IL1A/PPARA/CD80/CCR2/CDC42/CD28/ERBB2/IL2/IFNG/IL4/CD40LG/GNRH1/PTPN6/SHH/ALOX5/PDE5A 29

BP GO:0071260 cellular response to mechanical stimulus 17/227 79/18866 5.24783E-17 2.31002E-15 8.72592E-16 PTGS2/CASP8/EGFR/AKT1/IL1B/TLR4/MAPK3/MMP7/GCLC/MAPK8/BAK1/GJA1/TNFRSF10B/TNFRSF10A/NFKB1/CASP1/TNFRSF1A 17

BP GO:0031668 cellular response to extracellular stimulus 26/227 253/18866 5.31666E-17 2.31884E-15 8.75926E-16 BCL2/JUN/PPARG/FOS/CDKN1A/MAPK1/TP53/HMOX1/NFE2L2/ATF3/MAPK3/SIRT1/MAPK8/FOXO1/ICAM1/VCAM1/PPARA/MTOR/EIF2S1/MDM2/GSTP1/NR1H4/VDR/EIF2AK3/EIF2AK4/EIF2AK2 26

BP GO:0007159 leukocyte cell-cell adhesion 30/227 364/18866 8.70517E-17 3.76222E-15 1.42115E-15 CASP3/RELA/DPP4/AKT1/IL10/TNF/IL6/IL1B/SYK/ICAM1/CCL2/SELE/VCAM1/PECAM1/IL1A/PPARA/CD80/CCR2/CDC42/CD28/ERBB2/IL2/IFNG/IL4/CD40LG/GNRH1/PTPN6/SHH/ALOX5/PDE5A 30

BP GO:0035094 response to nicotine 14/227 45/18866 1.06889E-16 4.57794E-15 1.72928E-15 SLC6A3/CHRNA7/BCL2/CASP3/RELA/MAPK1/TNF/HMOX1/NTRK1/EDN1/PPARA/NFKB1/CREB1/DRD2 14

BP GO:0051403 stress-activated MAPK cascade 27/227 286/18866 1.12132E-16 4.75959E-15 1.7979E-15 EGFR/AKT1/VEGFA/MAPK1/TNF/XDH/MYC/IL1B/TLR4/MAPK3/APP/MMP8/SYK/MAPK14/MAPK8/IGF1R/EDN1/FOXO1/CDC42/NFKB1/GSTP1/CD40LG/TRPV4/HMGCR/MAPK10/MAPK9/EIF2AK2 27

BP GO:0048015 phosphatidylinositol-mediated signaling 23/227 192/18866 1.23816E-16 5.20904E-15 1.96768E-15 HTR2A/PIK3CG/EGFR/AKT1/MAPK1/TNF/KDR/PPARD/MAPK3/NGF/NTRK1/NTRK2/CAT/F2/SIRT1/IGF1R/EDN1/CD28/ERBB2/INSR/PTPN6/CSF1R/HTR2B 23

BP GO:2001056 positive regulation of cysteine-type endopeptidase activity 21/227 151/18866 1.26245E-16 5.26464E-15 1.98868E-15 BAX/CASP9/CASP8/PPARG/TNF/XDH/MYC/SNCA/NGF/SYK/FASLG/SIRT1/BAK1/TNFRSF10B/CFLAR/TNFSF10/TNFRSF10A/VCP/CASP1/EIF2AK3/CTSD 21

BP GO:0070374 positive regulation of ERK1 and ERK2 cascade 24/227 215/18866 1.34596E-16 5.5641E-15 2.1018E-15 HTR2A/CHRNA7/OPRM1/JUN/PRKCA/TGFB1/EGFR/TNF/KDR/TLR4/MAPK3/NTRK1/APP/HTR2C/ICAM1/CCL2/CFLAR/C5AR1/TRPV4/HMGCR/CCR1/CSF1R/DRD2/HTR2B 24

BP GO:0050730 regulation of peptidyl-tyrosine phosphorylation 26/227 263/18866 1.38311E-16 5.66836E-15 2.14118E-15 HTR2A/TGFB1/EGFR/VEGFA/EGF/TNF/IL6/TP53/BDNF/NTRK2/APP/SYK/STAT3/ICAM1/PECAM1/CD80/MTOR/IL2/IFNG/IL4/PSEN1/ENPP2/CSF2/PTPN6/TNFRSF1A/CSF1R 26

BP GO:0072503 cellular divalent inorganic cation homeostasis 34/227 492/18866 1.42646E-16 5.79604E-15 2.18941E-15 HTR2A/CHRNA7/PIK3CG/BCL2/BAX/ESR1/DDIT3/SNCA/APP/HTR2C/CDK5/F2/FASLG/BAK1/EDN1/GJA1/PRKCB/AGTR1/C5AR1/CCR2/IL2/GRM1/TRPV4/PSEN1/PTPN6/TRPV1/CCR1/CCR3/VDR/TACR1/BDKRB1/CCR5/DRD2/HTR2B 34

BP GO:0045834 positive regulation of lipid metabolic process 21/227 153/18866 1.66249E-16 6.69788E-15 2.53008E-15 PTGS2/HTR2A/TGFB1/PPARG/AKT1/TNF/PPARD/IL1B/HTR2C/F2/PPARA/AGTR1/MTOR/CDC42/NR1H3/IFNG/CREB1/NR1H4/TNFRSF1A/ABCG1/HTR2B 21

BP GO:0014065 phosphatidylinositol 3-kinase signaling 21/227 154/18866 1.90483E-16 7.60973E-15 2.87452E-15 HTR2A/PIK3CG/EGFR/AKT1/MAPK1/TNF/KDR/PPARD/MAPK3/NTRK1/NTRK2/CAT/F2/SIRT1/IGF1R/EDN1/CD28/ERBB2/INSR/PTPN6/HTR2B 21

BP GO:0048017 inositol lipid-mediated signaling 23/227 196/18866 1.96423E-16 7.78162E-15 2.93945E-15 HTR2A/PIK3CG/EGFR/AKT1/MAPK1/TNF/KDR/PPARD/MAPK3/NGF/NTRK1/NTRK2/CAT/F2/SIRT1/IGF1R/EDN1/CD28/ERBB2/INSR/PTPN6/CSF1R/HTR2B 23

BP GO:0071241 cellular response to inorganic substance 24/227 221/18866 2.54086E-16 9.98284E-15 3.77095E-15 PTGS2/JUN/MMP3/EGFR/AKT1/FOS/MMP9/MAPK1/SOD1/HMOX1/NFE2L2/SNCA/MAPK3/APP/PARP1/MAPK8/EDN1/FOXO1/CFLAR/EIF2S1/CREB1/SHH/EIF2AK3/MAPK9 24

BP GO:0071356 cellular response to tumor necrosis factor 27/227 297/18866 2.90842E-16 1.13333E-14 4.28108E-15 CASP8/RELA/AKT1/MAPK1/TNF/TP53/STAT1/NFE2L2/MAPK3/SYK/MAPK14/SIRT1/EDN1/ICAM1/CCL2/CXCL8/BRCA1/BIRC3/NFKB1/GSTP1/CD40LG/CASP1/NR1H4/TRPV1/TNFRSF1A/TNFRSF1B/BIRC2 27

BP GO:0010742 macrophage derived foam cell differentiation 13/227 38/18866 3.2569E-16 1.24865E-14 4.7167E-15 TGFB1/PPARG/STAT1/PPARA/CRP/AGTR1/NR1H3/NFKB1/CSF2/CETP/ABCG1/EP300/MAPK9 13

BP GO:0090077 foam cell differentiation 13/227 38/18866 3.2569E-16 1.24865E-14 4.7167E-15 TGFB1/PPARG/STAT1/PPARA/CRP/AGTR1/NR1H3/NFKB1/CSF2/CETP/ABCG1/EP300/MAPK9 13

BP GO:0009636 response to toxic substance 25/227 250/18866 4.18798E-16 1.59277E-14 6.01658E-15 PTGS2/SLC6A4/BCL2/BAX/PON1/FOS/CDKN1A/MAPK1/TNF/SOD1/NFE2L2/MAPK3/CAT/CDK1/ABCG2/MPO/BAK1/SOD2/MDM2/GSTP1/GPX1/GPX4/SCN9A/DHFR/DRD2 25

BP GO:0010950 positive regulation of endopeptidase activity 22/227 181/18866 4.29382E-16 1.62007E-14 6.11968E-15 BAX/CASP9/CASP8/PPARG/TNF/XDH/MYC/SNCA/NGF/SYK/FASLG/SIRT1/STAT3/BAK1/TNFRSF10B/CFLAR/TNFSF10/TNFRSF10A/VCP/CASP1/EIF2AK3/CTSD 22

BP GO:0051480 regulation of cytosolic calcium ion concentration 29/227 357/18866 4.33475E-16 1.62263E-14 6.12937E-15 HTR2A/PIK3CG/BCL2/BAX/ESR1/DDIT3/SNCA/HTR2C/CDK5/F2/FASLG/BAK1/EDN1/GJA1/AGTR1/C5AR1/CCR2/IL2/GRM1/TRPV4/PTPN6/TRPV1/CCR1/CCR3/TACR1/BDKRB1/CCR5/DRD2/HTR2B 29

BP GO:0008625 extrinsic apoptotic signaling pathway via death domain receptors 17/227 89/18866 4.47659E-16 1.66263E-14 6.28047E-15 BCL2/BAX/CASP8/BCL2L1/TNF/HMOX1/ATF3/NGF/FASLG/ICAM1/BRCA1/TNFRSF10B/CFLAR/TNFSF10/TNFRSF10A/GPX1/TNFRSF1A 17

BP GO:0038034 signal transduction in absence of ligand 16/227 75/18866 5.17366E-16 1.89197E-14 7.14677E-15 BCL2/BAX/CASP9/CASP3/AKT1/BCL2L1/TNF/IL1B/GDNF/MCL1/BAK1/IL1A/IL2/IL4/CSF2/RET 16

BP GO:0097192 extrinsic apoptotic signaling pathway in absence of ligand 16/227 75/18866 5.17366E-16 1.89197E-14 7.14677E-15 BCL2/BAX/CASP9/CASP3/AKT1/BCL2L1/TNF/IL1B/GDNF/MCL1/BAK1/IL1A/IL2/IL4/CSF2/RET 16

BP GO:0043491 protein kinase B signaling 26/227 278/18866 5.37314E-16 1.94992E-14 7.36567E-15 PIK3CG/TGFB1/EGFR/AKT1/EGF/TNF/XDH/KDR/ESR1/IL1B/DDIT3/NTRK1/NTRK2/HSP90AA1/SIRT1/IGF1R/CCL2/CD80/MTOR/CD28/ERBB2/INSR/GPX1/DRD2/TGFBR1/RET 26

BP GO:0001936 regulation of endothelial cell proliferation 22/227 184/18866 6.10984E-16 2.20047E-14 8.31212E-15 JUN/PRKCA/PPARG/AKT1/VEGFA/IL10/TNF/XDH/HIF1A/STAT1/KDR/HMOX1/SIRT1/STAT3/GJA1/CCL2/AGTR1/MTOR/ALOX5/CCR3/TGFBR1/HTR2B 22

BP GO:1903426 regulation of reactive oxygen species biosynthetic process 18/227 107/18866 6.38697E-16 2.28298E-14 8.6238E-15 PTGS2/AKT1/IL10/TNF/IL1B/TLR4/SNCA/HSP90AA1/MMP8/STAT3/EDN1/ICAM1/CFLAR/MTOR/IFNG/INSR/TRPV1/ALOX5 18

BP GO:0050804 modulation of chemical synaptic transmission 32/227 454/18866 6.92573E-16 2.45709E-14 9.28146E-15 PTGS2/HTR2A/CHRNA7/SLC6A4/ACHE/EGFR/MAPK1/TNF/IL1B/GDNF/SNCA/BDNF/NGF/NTRK1/NTRK2/CA2/APP/CDK5/STAT3/EDN1/CCL2/PRKCB/PLAT/MTOR/CCR2/GRM1/BCHE/PRKCG/PSEN1/CREB1/DRD2/EIF2AK4 32

BP GO:0031960 response to corticosteroid 21/227 164/18866 7.03832E-16 2.47853E-14 9.36248E-15 PTGS2/BCL2/CASP9/CASP3/EGFR/FOS/CDKN1A/IL10/TNF/IL6/PARP1/EDN1/FOXO1/ICAM1/CFLAR/GSTP1/GNRH1/ALDH3A1/BCHE/PAM/GLB1 21

BP GO:0099177 regulation of trans-synaptic signaling 32/227 455/18866 7.37357E-16 2.5775E-14 9.7363E-15 PTGS2/HTR2A/CHRNA7/SLC6A4/ACHE/EGFR/MAPK1/TNF/IL1B/GDNF/SNCA/BDNF/NGF/NTRK1/NTRK2/CA2/APP/CDK5/STAT3/EDN1/CCL2/PRKCB/PLAT/MTOR/CCR2/GRM1/BCHE/PRKCG/PSEN1/CREB1/DRD2/EIF2AK4 32

BP GO:0045785 positive regulation of cell adhesion 31/227 428/18866 9.64172E-16 3.34575E-14 1.26383E-14 PRKCA/RELA/DPP4/AKT1/VEGFA/IL10/TNF/IL6/KDR/IL1B/SYK/CDK6/ICAM1/CCL2/SELE/VCAM1/IL1A/CD80/JAK1/CCR2/CDC42/CD28/ERBB2/IL2/IFNG/IL4/CD40LG/PTPN6/SHH/ALOX5/RET 31

BP GO:0051047 positive regulation of secretion 28/227 340/18866 1.01748E-15 3.50513E-14 1.32404E-14 SLC6A4/TGFB1/ACHE/EGFR/IL10/TNF/IL6/HIF1A/PPARD/IL1B/TLR4/GDNF/SNCA/CYP19A1/CDK5/SYK/EDN1/GJA1/IL1A/IL2/IFNG/IL4/CREB1/NR1H4/TRPV1/ABCG1/DRD2/HTR2B 28

BP GO:0032943 mononuclear cell proliferation 26/227 286/18866 1.07027E-15 3.66048E-14 1.38272E-14 PIK3CG/BCL2/BAX/CASP3/CDKN1A/IL10/IL6/TP53/IL1B/TLR4/SYK/VCAM1/IL1A/CRP/CD80/CCR2/CD28/ERBB2/IL2/IL4/CD40LG/GNRH1/PTPN6/SHH/TNFRSF1B/PDE5A 26

BP GO:1903532 positive regulation of secretion by cell 27/227 313/18866 1.08283E-15 3.67697E-14 1.38895E-14 SLC6A4/TGFB1/ACHE/EGFR/IL10/TNF/IL6/HIF1A/PPARD/IL1B/TLR4/GDNF/SNCA/CYP19A1/CDK5/SYK/EDN1/GJA1/IL1A/IL2/IFNG/IL4/CREB1/NR1H4/ABCG1/DRD2/HTR2B 27

BP GO:0002685 regulation of leukocyte migration 23/227 212/18866 1.12164E-15 3.78175E-14 1.42853E-14 DPP4/AKT1/VEGFA/MAPK1/TNF/IL6/HMOX1/MAPK3/APP/CYP19A1/MAPK14/EDN1/ICAM1/CCL2/SELE/CXCL8/C5AR1/CCR2/IL4/TRPV4/CCR1/CSF1R/BDKRB1 23

BP GO:0019216 regulation of lipid metabolic process 31/227 431/18866 1.17011E-15 3.91739E-14 1.47977E-14 RXRA/PTGS2/HTR2A/PIK3CG/TGFB1/PPARG/AKT1/TNF/SOD1/PPARD/IL1B/HTR2C/F2/SIRT1/BRCA1/PPARA/AGTR1/MTOR/CDC42/NR1H3/NFKB1/IFNG/HMGCR/CREB1/NR1H4/TNFRSF1A/ABCG1/FDFT1/VDR/IDH1/HTR2B 31

BP GO:0045765 regulation of angiogenesis 30/227 403/18866 1.39354E-15 4.63279E-14 1.75E-14 PTGS2/CHRNA7/PRKCA/PPARG/VEGFA/IL10/TNF/IL6/HIF1A/STAT1/KDR/IL1B/HMOX1/NFE2L2/FASLG/SIRT1/STAT3/CXCL8/BRCA1/PRKCB/IL1A/JAK1/AGTR1/C5AR1/CCR2/ERBB2/ENPP2/ATF2/ALOX5/CCR3 30

BP GO:1902105 regulation of leukocyte differentiation 26/227 290/18866 1.49781E-15 4.94484E-14 1.86788E-14 JUN/CASP8/PRKCA/TGFB1/FOS/TNF/SOD1/MYC/IL1B/TLR4/CA2/SYK/CDK6/IL1A/CD80/MTOR/CCR2/CD28/ERBB2/IL2/IFNG/IL4/CREB1/PTPN6/SHH/CCR1 26

BP GO:0030522 intracellular receptor signaling pathway 25/227 265/18866 1.65243E-15 5.41769E-14 2.04649E-14 PGR/RXRA/CASP8/RELA/AR/PPARG/ESR2/PPARD/ESR1/TLR4/PARP1/SIRT1/STAT3/BRCA1/XIAP/PPARA/BIRC3/NR1H3/RXRB/NR1I2/NR1H4/RXRG/VDR/BIRC2/EP300 25

BP GO:1905952 regulation of lipid localization 21/227 171/18866 1.66519E-15 5.42212E-14 2.04817E-14 PON1/PPARG/AKT1/EGF/TNF/IL6/PPARD/IL1B/CYP19A1/SYK/SIRT1/EDN1/PPARA/CRP/AGTR1/NR1H3/NFKB1/SHH/CETP/ABCG1/REN 21

BP GO:0007623 circadian rhythm 23/227 218/18866 2.07316E-15 6.70464E-14 2.53263E-14 SLC6A4/JUN/PPARG/NOS2/EGFR/TP53/TOP1/BDNF/NTRK1/NTRK2/CDK1/SIRT1/MAPK8/PPARA/MTOR/NR1H3/PRKCG/CREB1/CSF2/EP300/MAPK10/MAPK9/DRD2 23

BP GO:0042098 T cell proliferation 22/227 195/18866 2.10807E-15 6.72601E-14 2.5407E-14 PIK3CG/BAX/CASP3/IL10/IL6/TP53/IL1B/SYK/VCAM1/IL1A/CD80/CCR2/CD28/ERBB2/IL2/IL4/CD40LG/GNRH1/PTPN6/SHH/TNFRSF1B/PDE5A 22

BP GO:0050731 positive regulation of peptidyl-tyrosine phosphorylation 22/227 195/18866 2.10807E-15 6.72601E-14 2.5407E-14 HTR2A/TGFB1/VEGFA/EGF/TNF/IL6/TP53/BDNF/NTRK2/SYK/STAT3/ICAM1/PECAM1/CD80/MTOR/IL2/IFNG/IL4/ENPP2/CSF2/TNFRSF1A/CSF1R 22

BP GO:0001659 temperature homeostasis 21/227 174/18866 2.37877E-15 7.5391E-14 2.84784E-14 PTGS2/HTR2A/ACHE/VEGFA/TNF/IL1B/TLR4/DDIT3/SYK/STAT3/IGF1R/FOXO1/GJA1/IL1A/CCR2/NR1H3/IL4/GPX1/TRPV1/UCP2/DRD2 21

BP GO:0002791 regulation of peptide secretion 29/227 381/18866 2.40639E-15 7.57614E-14 2.86183E-14 OPRM1/PRKCA/TGFB1/DPP4/NOS2/ACHE/EGFR/IL10/TNF/IL6/HIF1A/PPARD/IL1B/TLR4/HTR2C/SYK/GJA1/IL1A/NR1H3/IL2/IFNG/CD40LG/HMGCR/PAM/NR1H4/ALOX5/ABCG1/DRD2/HTR2B 29

BP GO:0050708 regulation of protein secretion 28/227 352/18866 2.46959E-15 7.72396E-14 2.91767E-14 OPRM1/PRKCA/TGFB1/DPP4/NOS2/ACHE/EGFR/IL10/TNF/IL6/HIF1A/PPARD/IL1B/TLR4/SYK/GJA1/IL1A/NR1H3/IL2/IFNG/CD40LG/HMGCR/PAM/NR1H4/ALOX5/ABCG1/DRD2/HTR2B 28

BP GO:0031669 cellular response to nutrient levels 23/227 221/18866 2.79791E-15 8.63718E-14 3.26263E-14 BCL2/JUN/PPARG/CDKN1A/MAPK1/TP53/HMOX1/NFE2L2/ATF3/MAPK3/SIRT1/MAPK8/FOXO1/ICAM1/PPARA/MTOR/EIF2S1/MDM2/NR1H4/VDR/EIF2AK3/EIF2AK4/EIF2AK2 23

BP GO:0032944 regulation of mononuclear cell proliferation 23/227 221/18866 2.79791E-15 8.63718E-14 3.26263E-14 BCL2/CASP3/CDKN1A/IL10/IL6/IL1B/TLR4/SYK/VCAM1/IL1A/CRP/CD80/CCR2/CD28/ERBB2/IL2/IL4/CD40LG/GNRH1/PTPN6/SHH/TNFRSF1B/PDE5A 23

BP GO:0001935 endothelial cell proliferation 22/227 199/18866 3.2418E-15 9.94293E-14 3.75587E-14 JUN/PRKCA/PPARG/AKT1/VEGFA/IL10/TNF/XDH/HIF1A/STAT1/KDR/HMOX1/SIRT1/STAT3/GJA1/CCL2/AGTR1/MTOR/ALOX5/CCR3/TGFBR1/HTR2B 22

BP GO:1904645 response to amyloid-beta 14/227 56/18866 3.30893E-15 1.00837E-13 3.80906E-14 CHRNA7/MMP3/MMP2/MMP9/TLR4/MMP12/APP/CDK5/PARP1/IGF1R/GJA1/ICAM1/VCAM1/PSEN1 14

BP GO:0045429 positive regulation of nitric oxide biosynthetic process 13/227 45/18866 4.07721E-15 1.23459E-13 4.66357E-14 PTGS2/AKT1/TNF/IL1B/TLR4/HSP90AA1/MMP8/EDN1/ICAM1/MTOR/IFNG/INSR/TRPV1 13

BP GO:0043122 regulation of I-kappaB kinase/NF-kappaB signaling 24/227 252/18866 5.05533E-15 1.52108E-13 5.74576E-14 CASP8/RELA/AKT1/TNF/STAT1/ESR1/IL1B/HMOX1/TLR4/FASLG/SIRT1/GJA1/PRKCB/TNFRSF10B/CFLAR/TNFSF10/BIRC3/GSTP1/LITAF/CASP1/NR1H4/TNFRSF1A/BIRC2/HTR2B 24

BP GO:0006919 activation of cysteine-type endopeptidase activity involved in apoptotic process 16/227 86/18866 5.20562E-15 1.55645E-13 5.87937E-14 BAX/CASP9/CASP8/PPARG/TNF/XDH/SNCA/NGF/FASLG/BAK1/TNFRSF10B/CFLAR/TNFSF10/TNFRSF10A/VCP/EIF2AK3 16

BP GO:0022409 positive regulation of cell-cell adhesion 25/227 279/18866 5.49292E-15 1.63208E-13 6.16508E-14 RELA/DPP4/AKT1/IL10/TNF/IL6/IL1B/SYK/ICAM1/CCL2/SELE/VCAM1/IL1A/CD80/JAK1/CCR2/CDC42/CD28/IL2/IFNG/IL4/CD40LG/PTPN6/SHH/ALOX5 25

BP GO:1904407 positive regulation of nitric oxide metabolic process 13/227 46/18866 5.62349E-15 1.6605E-13 6.27242E-14 PTGS2/AKT1/TNF/IL1B/TLR4/HSP90AA1/MMP8/EDN1/ICAM1/MTOR/IFNG/INSR/TRPV1 13

BP GO:0048661 positive regulation of smooth muscle cell proliferation 17/227 103/18866 5.85214E-15 1.71735E-13 6.48717E-14 PTGS2/JUN/EGFR/AKT1/MMP2/MMP9/IL10/TNF/IL6/STAT1/HMOX1/AKR1B1/EDN1/GJA1/MTOR/MDM2/HMGCR 17

BP GO:0006816 calcium ion transport 30/227 426/18866 6.17539E-15 1.80109E-13 6.8035E-14 PTGS2/HTR2A/CHRNA7/PIK3CG/OPRM1/BCL2/BAX/EGF/DDIT3/SNCA/HTR2C/CDK5/F2/FASLG/BAK1/GJA1/ICAM1/CCL2/PRKCB/TRPV4/PSEN1/PSEN2/PTPN6/TRPV1/CCR1/VDR/BDKRB1/CCR5/DRD2/HTR2B 30

BP GO:0051098 regulation of binding 28/227 367/18866 7.12114E-15 2.06426E-13 7.7976E-14 BAX/JUN/TGFB1/PON1/MAP2/PPARG/AKT1/CDKN1A/MMP9/IL10/EGF/HMOX1/DDIT3/BDNF/MAPK3/NGF/APP/MMP8/CDK5/PARP1/MAPK8/PPARA/EIF2S1/CDC42/IFNG/PSEN1/EP300/TGFBR1 28

BP GO:0046890 regulation of lipid biosynthetic process 22/227 207/18866 7.44406E-15 2.14479E-13 8.1018E-14 PTGS2/HTR2A/AKT1/TNF/SOD1/IL1B/HTR2C/SIRT1/BRCA1/MTOR/NR1H3/NFKB1/IFNG/HMGCR/CREB1/NR1H4/TNFRSF1A/ABCG1/FDFT1/VDR/IDH1/HTR2B 22

BP GO:0046651 lymphocyte proliferation 25/227 283/18866 7.64207E-15 2.18858E-13 8.2672E-14 PIK3CG/BCL2/BAX/CASP3/CDKN1A/IL10/IL6/TP53/IL1B/TLR4/SYK/VCAM1/IL1A/CD80/CCR2/CD28/ERBB2/IL2/IL4/CD40LG/GNRH1/PTPN6/SHH/TNFRSF1B/PDE5A 25

BP GO:0030225 macrophage differentiation 13/227 47/18866 7.69173E-15 2.18961E-13 8.27109E-14 CASP8/PRKCA/TGFB1/VEGFA/MMP9/APP/PARP1/SIRT1/CDC42/IFNG/CSF2/CSF1R/EIF2AK1 13

BP GO:0060326 cell chemotaxis 26/227 311/18866 8.02839E-15 2.27184E-13 8.58172E-14 PIK3CG/DPP4/VEGFA/MAPK1/IL10/IL6/KDR/IL1B/MAPK3/CYP19A1/SYK/MAPK14/EDN1/CCL2/CXCL8/AGTR1/C5AR1/CCR2/IL4/GSTP1/TRPV4/ALOX5/CCR1/CCR3/CSF1R/CCR5 26

BP GO:1903039 positive regulation of leukocyte cell-cell adhesion 23/227 235/18866 1.06729E-14 3.0023E-13 1.1341E-13 RELA/DPP4/AKT1/TNF/IL6/IL1B/SYK/ICAM1/CCL2/SELE/VCAM1/IL1A/CD80/CCR2/CDC42/CD28/IL2/IFNG/IL4/CD40LG/PTPN6/SHH/ALOX5 23

BP GO:0051384 response to glucocorticoid 19/227 147/18866 1.49085E-14 4.16911E-13 1.57485E-13 PTGS2/BCL2/CASP9/CASP3/EGFR/FOS/CDKN1A/IL10/TNF/IL6/EDN1/FOXO1/ICAM1/CFLAR/GSTP1/ALDH3A1/BCHE/PAM/GLB1 19

BP GO:0043524 negative regulation of neuron apoptotic process 19/227 149/18866 1.91941E-14 5.33618E-13 2.0157E-13 BCL2/BAX/JUN/BCL2L1/IL10/SOD1/HIF1A/HMOX1/GDNF/BDNF/NGF/NTRK1/NTRK2/GCLC/CCL2/SOD2/C5AR1/PRKCG/PSEN1 19

BP GO:0071248 cellular response to metal ion 21/227 193/18866 1.95162E-14 5.39418E-13 2.03761E-13 PTGS2/JUN/EGFR/AKT1/FOS/MMP9/MAPK1/SOD1/HMOX1/NFE2L2/SNCA/MAPK3/APP/PARP1/MAPK8/EDN1/EIF2S1/CREB1/SHH/EIF2AK3/MAPK9 21

BP GO:0015850 organic hydroxy compound transport 24/227 268/18866 2.00819E-14 5.51846E-13 2.08456E-13 RXRA/SLC6A3/HTR2A/SLC6A4/PON1/PPARG/EGF/GDNF/SNCA/CYP19A1/SYK/SIRT1/AGTR1/NR1H3/NFKB1/NPC1L1/NR1H4/SHH/CETP/ABCG1/HRH3/HTR1A/REN/DRD2 24

BP GO:0048638 regulation of developmental growth 27/227 353/18866 2.10052E-14 5.739E-13 2.16787E-13 SLC6A3/SLC6A4/BCL2/MAP2/AR/AKT1/VEGFA/CDKN1A/MAPK1/SOD1/PPARD/BDNF/NGF/APP/CDK1/CDK5/MAPK14/STAT3/EDN1/GJA1/PPARA/MTOR/INSR/GAMT/CREB1/DRD2/TGFBR1 27

BP GO:0050670 regulation of lymphocyte proliferation 22/227 219/18866 2.4205E-14 6.57547E-13 2.48384E-13 BCL2/CASP3/CDKN1A/IL10/IL6/IL1B/TLR4/SYK/VCAM1/IL1A/CD80/CCR2/CD28/ERBB2/IL2/IL4/CD40LG/GNRH1/PTPN6/SHH/TNFRSF1B/PDE5A 22

BP GO:0046686 response to cadmium ion 14/227 64/18866 2.50614E-14 6.76942E-13 2.5571E-13 JUN/EGFR/AKT1/FOS/MMP9/MAPK1/SOD1/HMOX1/MAPK3/CAT/GCLC/CDK1/MAPK8/MAPK9 14

BP GO:0006809 nitric oxide biosynthetic process 15/227 79/18866 2.75016E-14 7.38658E-13 2.79023E-13 PTGS2/NOS2/AKT1/IL10/TNF/IL1B/TLR4/HSP90AA1/MMP8/EDN1/ICAM1/MTOR/IFNG/INSR/TRPV1 15

BP GO:0032963 collagen metabolic process 17/227 113/18866 2.89745E-14 7.70427E-13 2.91023E-13 TGFB1/PPARG/MMP3/MMP2/MMP9/IL6/MMP1/HIF1A/PPARD/MMP12/MMP7/MMP8/F2/CTSB/MMP10/CTSD/TMPRSS6 17

BP GO:0001101 response to acid chemical 18/227 132/18866 2.90085E-14 7.70427E-13 2.91023E-13 CASP3/RELA/EGFR/VEGFA/BCL2L1/MMP2/TNF/NTRK1/NTRK2/AKR1B1/GCLC/EDN1/ICAM1/MTOR/GSTP1/CREB1/ATF2/MAPK10 18

BP GO:0043112 receptor metabolic process 21/227 198/18866 3.26298E-14 8.61116E-13 3.25281E-13 TGFB1/PPARG/ACHE/VEGFA/IL10/EGF/TNF/HIF1A/SNCA/CDK5/SYK/EDN1/SELE/CXCL8/PPARA/NR1H3/IFNG/INSR/PSEN1/DRD2/HTR2B 21

BP GO:0031331 positive regulation of cellular catabolic process 28/227 390/18866 3.27855E-14 8.61116E-13 3.25281E-13 HTR2A/BAX/AKT1/EGF/TNF/IL6/HIF1A/KDR/IL1B/HMOX1/NFE2L2/SNCA/MAPK3/HSP90AA1/APP/GCLC/SIRT1/FOXO1/PPARA/MDM2/IFNG/IL4/INSR/VCP/PSEN1/TNFRSF1B/MAPK9/EIF2AK4 28

BP GO:0008202 steroid metabolic process 26/227 332/18866 3.78096E-14 9.87619E-13 3.73066E-13 RXRA/PON1/TNF/SOD1/PPARD/ESR1/IL1B/AKR1B1/APP/CAT/CYP19A1/SIRT1/AGTR1/NFKB1/IFNG/IL4/CYP3A4/NR1I2/HMGCR/NPC1L1/NR1H4/SHH/CETP/ABCG1/FDFT1/VDR 26

BP GO:0032768 regulation of monooxygenase activity 14/227 66/18866 3.96899E-14 1.03107E-12 3.8948E-13 EGFR/AKT1/TNF/HIF1A/IL1B/GDNF/SNCA/HSP90AA1/IL1A/NFKB1/IFNG/VDR/DHFR/HTR2B 14

BP GO:0051896 regulation of protein kinase B signaling 23/227 253/18866 5.23444E-14 1.35242E-12 5.10867E-13 PIK3CG/TGFB1/EGFR/AKT1/EGF/TNF/XDH/ESR1/DDIT3/NTRK1/NTRK2/HSP90AA1/SIRT1/IGF1R/CD80/MTOR/CD28/ERBB2/INSR/GPX1/DRD2/TGFBR1/RET 23

BP GO:1903708 positive regulation of hemopoiesis 21/227 204/18866 5.92869E-14 1.52023E-12 5.74256E-13 JUN/CASP8/PRKCA/TGFB1/FOS/TNF/HIF1A/STAT1/IL1B/CA2/SYK/MAPK14/STAT3/IL1A/CD80/IL2/IFNG/IL4/CREB1/SHH/CCR1 21

BP GO:0048708 astrocyte differentiation 15/227 83/18866 5.94789E-14 1.52023E-12 5.74256E-13 EGFR/MAPK1/TNF/IL6/IL1B/TLR4/MAPK3/APP/CDK6/F2/STAT3/C5AR1/IFNG/PSEN1/SHH 15

BP GO:0045428 regulation of nitric oxide biosynthetic process 14/227 68/18866 6.18566E-14 1.57255E-12 5.94018E-13 PTGS2/AKT1/IL10/TNF/IL1B/TLR4/HSP90AA1/MMP8/EDN1/ICAM1/MTOR/IFNG/INSR/TRPV1 14

BP GO:0032102 negative regulation of response to external stimulus 29/227 433/18866 6.56137E-14 1.65919E-12 6.26747E-13 SLC6A3/PLAU/PPARG/DPP4/IL10/TNF/SOD1/PPARD/MMP12/CYP19A1/F2/EDN1/GJA1/CCL2/PLAT/PPARA/NR1H3/NFKB1/IL2/IL4/GSTP1/HMGCR/NR1H4/GPX1/TNFRSF1A/ALOX5/TNFRSF1B/PRKG1/DRD2 29

BP GO:0046209 nitric oxide metabolic process 15/227 84/18866 7.16453E-14 1.80194E-12 6.80669E-13 PTGS2/NOS2/AKT1/IL10/TNF/IL1B/TLR4/HSP90AA1/MMP8/EDN1/ICAM1/MTOR/IFNG/INSR/TRPV1 15

BP GO:0042594 response to starvation 21/227 206/18866 7.20168E-14 1.80194E-12 6.80669E-13 BCL2/JUN/PPARG/CDKN1A/MAPK1/TP53/GPT/NFE2L2/ATF3/DDIT3/MAPK3/SIRT1/MAPK8/FOXO1/PPARA/MTOR/EIF2S1/EIF2AK3/EIF2AK4/EIF2AK2/EIF2AK1 21

BP GO:0042116 macrophage activation 16/227 101/18866 7.34586E-14 1.82839E-12 6.90661E-13 JUN/IL10/TNF/IL6/TLR4/SNCA/APP/MMP8/SYK/C5AR1/NR1H3/IFNG/IL4/CSF2/TRPV1/PLA2G4A 16

BP GO:0030595 leukocyte chemotaxis 22/227 232/18866 7.99636E-14 1.97993E-12 7.47905E-13 PIK3CG/DPP4/VEGFA/MAPK1/IL10/IL6/IL1B/MAPK3/CYP19A1/SYK/MAPK14/EDN1/CCL2/CXCL8/C5AR1/CCR2/IL4/TRPV4/ALOX5/CCR1/CSF1R/CCR5 22

BP GO:0007249 I-kappaB kinase/NF-kappaB signaling 24/227 286/18866 8.47738E-14 2.08816E-12 7.88787E-13 CASP8/RELA/AKT1/TNF/STAT1/ESR1/IL1B/HMOX1/TLR4/FASLG/SIRT1/GJA1/PRKCB/TNFRSF10B/CFLAR/TNFSF10/BIRC3/GSTP1/LITAF/CASP1/NR1H4/TNFRSF1A/BIRC2/HTR2B 24

BP GO:0070838 divalent metal ion transport 30/227 471/18866 8.73409E-14 2.1403E-12 8.08483E-13 PTGS2/HTR2A/CHRNA7/PIK3CG/OPRM1/BCL2/BAX/EGF/DDIT3/SNCA/HTR2C/CDK5/F2/FASLG/BAK1/GJA1/ICAM1/CCL2/PRKCB/TRPV4/PSEN1/PSEN2/PTPN6/TRPV1/CCR1/VDR/BDKRB1/CCR5/DRD2/HTR2B 30

BP GO:0032355 response to estradiol 18/227 141/18866 9.3744E-14 2.28543E-12 8.63305E-13 PTGS2/SLC6A4/CASP9/CASP3/CASP8/TGFB1/EGFR/IL10/ESR2/ESR1/CAT/CYP19A1/STAT3/GJA1/CFLAR/GSTP1/PAM/GPX4 18

BP GO:0010876 lipid localization 29/227 440/18866 9.86759E-14 2.39339E-12 9.04088E-13 RXRA/PON1/PPARG/NOS2/AKT1/EGF/TNF/IL6/PPARD/IL1B/CYP19A1/SYK/ABCB1/SIRT1/EDN1/PPARA/CRP/AGTR1/NR1H3/NFKB1/GM2A/NPC1L1/NR1H4/SHH/PLA2G4A/CETP/ABCG1/REN/DRD2 29

BP GO:1904018 positive regulation of vasculature development 22/227 235/18866 1.04189E-13 2.51429E-12 9.49754E-13 PTGS2/CHRNA7/PRKCA/VEGFA/IL10/HIF1A/KDR/IL1B/HMOX1/NFE2L2/SIRT1/STAT3/CXCL8/BRCA1/SOD2/PRKCB/IL1A/JAK1/AGTR1/CFLAR/C5AR1/CCR3 22

BP GO:0002687 positive regulation of leukocyte migration 18/227 142/18866 1.06222E-13 2.55039E-12 9.63392E-13 VEGFA/MAPK1/TNF/IL6/MAPK3/APP/MAPK14/EDN1/ICAM1/SELE/CXCL8/C5AR1/CCR2/IL4/TRPV4/CCR1/CSF1R/BDKRB1 18

BP GO:0007259 receptor signaling pathway via JAK-STAT 19/227 164/18866 1.13589E-13 2.71357E-12 1.02503E-12 IL10/EGF/TNF/IL6/STAT1/AKR1B1/CDK5/F2/STAT3/CCL2/PECAM1/CCR2/IL2/IFNG/IL4/CSF2/TNFRSF1A/CSF1R/RET 19

BP GO:2001057 reactive nitrogen species metabolic process 15/227 87/18866 1.23345E-13 2.9319E-12 1.1075E-12 PTGS2/NOS2/AKT1/IL10/TNF/IL1B/TLR4/HSP90AA1/MMP8/EDN1/ICAM1/MTOR/IFNG/INSR/TRPV1 15

BP GO:0032872 regulation of stress-activated MAPK cascade 22/227 237/18866 1.24019E-13 2.93326E-12 1.10802E-12 EGFR/AKT1/VEGFA/MAPK1/TNF/XDH/MYC/IL1B/TLR4/MAPK3/APP/MMP8/SYK/IGF1R/EDN1/FOXO1/CDC42/GSTP1/CD40LG/TRPV4/HMGCR/EIF2AK2 22

BP GO:0072511 divalent inorganic cation transport 30/227 478/18866 1.28258E-13 3.01852E-12 1.14022E-12 PTGS2/HTR2A/CHRNA7/PIK3CG/OPRM1/BCL2/BAX/EGF/DDIT3/SNCA/HTR2C/CDK5/F2/FASLG/BAK1/GJA1/ICAM1/CCL2/PRKCB/TRPV4/PSEN1/PSEN2/PTPN6/TRPV1/CCR1/VDR/BDKRB1/CCR5/DRD2/HTR2B 30

BP GO:0006367 transcription initiation from RNA polymerase II promoter 20/227 189/18866 1.42602E-13 3.33956E-12 1.2615E-12 PGR/RXRA/BAX/AR/PPARG/CDKN1A/TP53/ESR2/PPARD/ESR1/CDK1/CDK7/PPARA/NR1H3/RXRB/NR1I2/CREB1/NR1H4/RXRG/VDR 20

BP GO:0001667 ameboidal-type cell migration 30/227 481/18866 1.50897E-13 3.5165E-12 1.32833E-12 PTGS2/JUN/PRKCA/TGFB1/PPARG/DPP4/AKT1/VEGFA/MMP9/TNF/HIF1A/KDR/PPARD/HMOX1/NFE2L2/GDNF/SIRT1/EDN1/GJA1/PECAM1/MTOR/IFNG/IL4/PRSS3/ENPP2/SHH/GPX1/TGFBR1/HTR2B/RET 30

BP GO:0033135 regulation of peptidyl-serine phosphorylation 18/227 145/18866 1.53604E-13 3.56212E-12 1.34557E-12 PTGS2/BCL2/BAX/TGFB1/EGFR/AKT1/VEGFA/TNF/IL6/SNCA/BDNF/NGF/NTRK2/HSP90AA1/APP/BAK1/IFNG/RET 18

BP GO:0042129 regulation of T cell proliferation 19/227 167/18866 1.58466E-13 3.65702E-12 1.38141E-12 CASP3/IL10/IL6/IL1B/SYK/VCAM1/IL1A/CD80/CCR2/CD28/ERBB2/IL2/IL4/CD40LG/GNRH1/PTPN6/SHH/TNFRSF1B/PDE5A 19

BP GO:0070302 regulation of stress-activated protein kinase signaling cascade 22/227 240/18866 1.60542E-13 3.67156E-12 1.38691E-12 EGFR/AKT1/VEGFA/MAPK1/TNF/XDH/MYC/IL1B/TLR4/MAPK3/APP/MMP8/SYK/IGF1R/EDN1/FOXO1/CDC42/GSTP1/CD40LG/TRPV4/HMGCR/EIF2AK2 22

BP GO:0019233 sensory perception of pain 16/227 106/18866 1.60641E-13 3.67156E-12 1.38691E-12 PTGS2/HTR2A/OPRM1/MAPK1/IL10/MAPK3/NTRK1/CDK5/EDN1/CCL2/MTOR/CCR2/GRM1/TRPV1/SCN9A/BDKRB1 16

BP GO:0009896 positive regulation of catabolic process 29/227 454/18866 2.18029E-13 4.95938E-12 1.87337E-12 HTR2A/BAX/AKT1/EGF/TNF/IL6/HIF1A/KDR/IL1B/HMOX1/NFE2L2/SNCA/MAPK3/HSP90AA1/APP/GCLC/SIRT1/FOXO1/GJA1/PPARA/MDM2/IFNG/IL4/INSR/VCP/PSEN1/TNFRSF1B/MAPK9/EIF2AK4 29

BP GO:0007584 response to nutrient 19/227 171/18866 2.44404E-13 5.52006E-12 2.08516E-12 PTGS2/SLC6A4/RELA/PPARG/EGFR/STAT1/PPARD/HMOX1/CAT/GCLC/TYR/MTOR/MDM2/GSTP1/ALDH3A1/BCHE/HMGCR/NR1H4/VDR 19

BP GO:0061900 glial cell activation 13/227 60/18866 2.46162E-13 5.52006E-12 2.08516E-12 JUN/EGFR/TNF/IL6/IL1B/SNCA/APP/MMP8/C5AR1/IFNG/IL4/PSEN1/TRPV1 13

BP GO:1903428 positive regulation of reactive oxygen species biosynthetic process 13/227 60/18866 2.46162E-13 5.52006E-12 2.08516E-12 PTGS2/AKT1/TNF/IL1B/TLR4/HSP90AA1/MMP8/EDN1/ICAM1/MTOR/IFNG/INSR/TRPV1 13

BP GO:0033138 positive regulation of peptidyl-serine phosphorylation 16/227 109/18866 2.51747E-13 5.6188E-12 2.12246E-12 PTGS2/BCL2/TGFB1/EGFR/AKT1/VEGFA/TNF/IL6/SNCA/BDNF/NGF/NTRK2/HSP90AA1/APP/IFNG/RET 16

BP GO:0070665 positive regulation of leukocyte proliferation 18/227 150/18866 2.78636E-13 6.18988E-12 2.33818E-12 BCL2/CDKN1A/MAPK1/IL6/IL1B/TLR4/MAPK3/SYK/VCAM1/IL1A/CD80/CCR2/CD28/IL2/IL4/CD40LG/SHH/CSF1R 18

BP GO:0060759 regulation of response to cytokine stimulus 20/227 196/18866 2.84866E-13 6.29885E-12 2.37935E-12 CASP8/PPARG/TNF/IL6/HIF1A/STAT1/TLR4/MMP12/SYK/EDN1/JAK1/BIRC3/NR1H3/IFNG/GSTP1/PTPN6/CASP1/NR1H4/TNFRSF1A/BIRC2 20

BP GO:1900407 regulation of cellular response to oxidative stress 15/227 92/18866 2.91151E-13 6.40802E-12 2.42058E-12 MMP3/AKT1/IL10/TNF/SOD1/HIF1A/NFE2L2/TLR4/MCL1/PARP1/SIRT1/SOD2/GPX1/ALOX5/DHFR 15

BP GO:0050863 regulation of T cell activation 25/227 332/18866 2.92535E-13 6.40881E-12 2.42088E-12 CASP3/DPP4/AKT1/IL10/IL6/SOD1/IL1B/SYK/CCL2/VCAM1/IL1A/CD80/CCR2/CDC42/CD28/ERBB2/IL2/IFNG/IL4/CD40LG/GNRH1/PTPN6/SHH/TNFRSF1B/PDE5A 25

BP GO:0097696 receptor signaling pathway via STAT 19/227 174/18866 3.35669E-13 7.31534E-12 2.76332E-12 IL10/EGF/TNF/IL6/STAT1/AKR1B1/CDK5/F2/STAT3/CCL2/PECAM1/CCR2/IL2/IFNG/IL4/CSF2/TNFRSF1A/CSF1R/RET 19

BP GO:0002526 acute inflammatory response 16/227 111/18866 3.36992E-13 7.31534E-12 2.76332E-12 PTGS2/PIK3CG/OPRM1/PPARG/TNF/IL6/IL1B/F2/STAT3/ICAM1/IL1A/CRP/IL4/GSTP1/TRPV1/EIF2AK1 16

BP GO:0006352 DNA-templated transcription, initiation 22/227 249/18866 3.40594E-13 7.35993E-12 2.78016E-12 PGR/RXRA/BAX/JUN/AR/PPARG/CDKN1A/TP53/ESR2/PPARD/ESR1/MAPK3/CDK1/CDK7/PPARA/NR1H3/RXRB/NR1I2/CREB1/NR1H4/RXRG/VDR 22

BP GO:0030098 lymphocyte differentiation 26/227 368/18866 4.17336E-13 8.94325E-12 3.37825E-12 BCL2/BAX/IL10/IL6/TP53/SOD1/IL1B/NTRK1/SYK/CDK6/STAT3/BAK1/VCAM1/IL1A/CD80/MTOR/CCR2/CD28/ERBB2/IL2/IFNG/IL4/CD40LG/PTPN6/SHH/EP300 26

BP GO:1901654 response to ketone 20/227 200/18866 4.17628E-13 8.94325E-12 3.37825E-12 CASP9/TGFB1/RELA/AR/PPARG/EGFR/AKT1/BCL2L1/FOS/CDKN1A/AKR1B1/PARP1/SIRT1/EDN1/FOXO1/ICAM1/CFLAR/NR1H3/GNRH1/GLB1 20

BP GO:0071276 cellular response to cadmium ion 11/227 38/18866 5.43029E-13 1.15765E-11 4.37294E-12 JUN/EGFR/AKT1/FOS/MMP9/MAPK1/SOD1/HMOX1/MAPK3/MAPK8/MAPK9 11

BP GO:0001890 placenta development 18/227 156/18866 5.52725E-13 1.17306E-11 4.43115E-12 RXRA/PTGS2/CASP8/PPARG/EGFR/AKT1/MAPK1/IL10/SOD1/HIF1A/PPARD/MAPK3/MAPK14/GJA1/CTSB/CSF2/VDR/BIRC2 18

BP GO:0032868 response to insulin 23/227 283/18866 5.6244E-13 1.18837E-11 4.48899E-12 RELA/PPARG/AKT1/IL10/STAT1/IL1B/GPT/CAT/GCLC/PARP1/SIRT1/IGF1R/FOXO1/ICAM1/PRKCB/PPARA/CFLAR/MTOR/TNFSF10/GSTP1/INSR/TRPV4/NR1H4 23

BP GO:0050729 positive regulation of inflammatory response 18/227 158/18866 6.89805E-13 1.45103E-11 5.48117E-12 PTGS2/PIK3CG/EGFR/TNF/IL6/IL1B/TLR4/SNCA/MMP8/STAT3/AGTR1/IL17B/CCR2/CD28/IL2/IFNG/TRPV4/TNFRSF1A 18

BP GO:1902893 regulation of pri-miRNA transcription by RNA polymerase II 12/227 51/18866 7.22364E-13 1.51283E-11 5.7146E-12 JUN/TGFB1/RELA/PPARG/FOS/IL10/TNF/TP53/HIF1A/PPARD/STAT3/PPARA 12

BP GO:0071383 cellular response to steroid hormone stimulus 20/227 206/18866 7.29216E-13 1.52048E-11 5.7435E-12 PGR/RXRA/CASP9/AR/EGFR/ESR2/PPARD/ESR1/PARP1/SIRT1/EDN1/FOXO1/ICAM1/BRCA1/PPARA/CFLAR/GSTP1/RXRB/RXRG/EP300 20

BP GO:0051341 regulation of oxidoreductase activity 16/227 117/18866 7.80387E-13 1.61303E-11 6.09309E-12 EGFR/AKT1/TNF/HIF1A/IL1B/GDNF/SNCA/HSP90AA1/EDN1/IL1A/AGTR1/NFKB1/IFNG/VDR/DHFR/HTR2B 16

BP GO:1904019 epithelial cell apoptotic process 16/227 117/18866 7.80387E-13 1.61303E-11 6.09309E-12 PIK3CG/BCL2L1/IL10/TNF/IL6/KDR/HMOX1/NFE2L2/FASLG/ICAM1/CCL2/CFLAR/MTOR/EIF2S1/IL4/CD40LG 16

BP GO:0001959 regulation of cytokine-mediated signaling pathway 19/227 183/18866 8.38054E-13 1.72472E-11 6.51501E-12 CASP8/PPARG/TNF/IL6/HIF1A/STAT1/MMP12/SYK/EDN1/JAK1/BIRC3/NR1H3/IFNG/GSTP1/PTPN6/CASP1/NR1H4/TNFRSF1A/BIRC2 19

BP GO:0045766 positive regulation of angiogenesis 20/227 208/18866 8.7441E-13 1.79179E-11 6.76835E-12 PTGS2/CHRNA7/PRKCA/VEGFA/IL10/HIF1A/KDR/IL1B/HMOX1/NFE2L2/SIRT1/STAT3/CXCL8/BRCA1/PRKCB/IL1A/JAK1/AGTR1/C5AR1/CCR3 20

BP GO:0045637 regulation of myeloid cell differentiation 22/227 263/18866 1.03176E-12 2.10515E-11 7.95204E-12 JUN/CASP8/PRKCA/TGFB1/FOS/TNF/HIF1A/STAT1/MYC/TLR4/CA2/CDK6/MAPK14/STAT3/PRKCB/MTOR/IFNG/IL4/CREB1/CSF2/CCR1/EP300 22

BP GO:0046777 protein autophosphorylation 21/227 237/18866 1.13693E-12 2.30981E-11 8.72513E-12 JUN/EGFR/AKT1/VEGFA/KDR/MAPK3/NTRK1/NTRK2/CDK5/SYK/IGF1R/MTOR/EIF2S1/ERBB2/INSR/PRKCG/CSF1R/EIF2AK3/EIF2AK4/EIF2AK2/EIF2AK1 21

BP GO:0006066 alcohol metabolic process 26/227 385/18866 1.17756E-12 2.38217E-11 8.99848E-12 RXRA/PON1/TNF/SOD1/PPARD/IL1B/SNCA/AKR1B1/APP/CAT/TTR/NFKB1/IFNG/IL4/CYP3A4/TPI1/HMGCR/NPC1L1/NR1H4/PLA2G4A/CETP/ABCG1/FDFT1/VDR/IDH1/DHFR 26

BP GO:0061614 pri-miRNA transcription by RNA polymerase II 12/227 53/18866 1.18852E-12 2.39416E-11 9.04376E-12 JUN/TGFB1/RELA/PPARG/FOS/IL10/TNF/TP53/HIF1A/PPARD/STAT3/PPARA 12

BP GO:0009914 hormone transport 24/227 323/18866 1.19784E-12 2.40064E-11 9.06824E-12 PRKCA/DPP4/NOS2/EGFR/TNF/IL6/HIF1A/PPARD/IL1B/HTR2C/CYP19A1/TTR/EDN1/GJA1/AGTR1/IFNG/TRPV4/HMGCR/CREB1/NR1H4/ALOX5/HTR1A/REN/DRD2 24

BP GO:1902882 regulation of response to oxidative stress 15/227 101/18866 1.20183E-12 2.40064E-11 9.06824E-12 MMP3/AKT1/IL10/TNF/SOD1/HIF1A/NFE2L2/TLR4/MCL1/PARP1/SIRT1/SOD2/GPX1/ALOX5/DHFR 15

BP GO:0045913 positive regulation of carbohydrate metabolic process 14/227 84/18866 1.3445E-12 2.67436E-11 1.01022E-11 HTR2A/AKT1/EGF/HIF1A/GPT/SNCA/APP/SIRT1/FOXO1/PPARA/NFKB1/IFNG/INSR/PSEN1 14

BP GO:0030879 mammary gland development 17/227 142/18866 1.35595E-12 2.68591E-11 1.01458E-11 PGR/SLC6A3/BAX/AR/AKT1/VEGFA/MAPK1/EGF/XDH/HIF1A/ESR1/CYP19A1/PAM/CREB1/GPX1/CSF1R/VDR 17

BP GO:0043123 positive regulation of I-kappaB kinase/NF-kappaB signaling 19/227 188/18866 1.36239E-12 2.68746E-11 1.01517E-11 CASP8/RELA/AKT1/TNF/IL1B/HMOX1/TLR4/FASLG/GJA1/PRKCB/TNFRSF10B/CFLAR/TNFSF10/BIRC3/LITAF/CASP1/TNFRSF1A/BIRC2/HTR2B 19

BP GO:1902042 negative regulation of extrinsic apoptotic signaling pathway via death domain receptors 11/227 41/18866 1.38137E-12 2.71365E-11 1.02506E-11 CASP8/BCL2L1/HMOX1/FASLG/ICAM1/BRCA1/TNFRSF10B/CFLAR/TNFSF10/TNFRSF10A/GPX1 11

BP GO:0046883 regulation of hormone secretion 22/227 267/18866 1.39813E-12 2.73526E-11 1.03323E-11 PRKCA/DPP4/NOS2/EGFR/TNF/IL6/HIF1A/PPARD/IL1B/HTR2C/CYP19A1/EDN1/GJA1/AGTR1/IFNG/HMGCR/CREB1/NR1H4/ALOX5/HTR1A/REN/DRD2 22

BP GO:0016049 cell growth 29/227 490/18866 1.46865E-12 2.86147E-11 1.0809E-11 BCL2/TGFB1/MAP2/PPARG/EGFR/AKT1/VEGFA/CDKN1A/TP53/ESR2/PPARD/BDNF/NGF/HSP90AA1/APP/CDK5/F2/SIRT1/EDN1/GJA1/PPARA/AGTR1/MTOR/CDC42/ERBB2/IL2/BDKRB1/TGFBR1/EIF2AK4 29

BP GO:0021782 glial cell development 16/227 122/18866 1.51383E-12 2.93744E-11 1.1096E-11 EGFR/AKT1/TNF/IL6/SOD1/IL1B/TLR4/NTRK2/APP/CDK5/CDK6/C5AR1/IFNG/GSTP1/PSEN1/SHH 16

BP GO:0045598 regulation of fat cell differentiation 17/227 143/18866 1.52267E-12 2.9426E-11 1.11154E-11 PTGS2/HTR2A/TGFB1/PPARG/AKT1/TNF/IL6/PPARD/DDIT3/HTR2C/MAPK14/SIRT1/FOXO1/MTOR/TRPV4/CREB1/ALOX5 17

BP GO:0043154 negative regulation of cysteine-type endopeptidase activity involved in apoptotic process 14/227 85/18866 1.59263E-12 3.06534E-11 1.15791E-11 PTGS2/AKT1/VEGFA/MMP9/TNF/SNCA/NGF/BIRC5/XIAP/CFLAR/BIRC3/MDM2/GPX1/BIRC2 14

BP GO:0008630 intrinsic apoptotic signaling pathway in response to DNA damage 15/227 103/18866 1.61462E-12 3.09511E-11 1.16916E-11 BCL2/BAX/CASP9/BCL2L1/CDKN1A/TNF/TP53/HMOX1/MCL1/SIRT1/BAK1/BRCA1/TNFRSF1A/TNFRSF1B/EP300 15

BP GO:2001242 regulation of intrinsic apoptotic signaling pathway 18/227 166/18866 1.62149E-12 3.0958E-11 1.16942E-11 PTGS2/BCL2/BAX/AKT1/BCL2L1/MMP9/TP53/SOD1/HIF1A/NFE2L2/DDIT3/MCL1/PARP1/SIRT1/SOD2/MDM2/GPX1/EIF2AK3 18

BP GO:0043279 response to alkaloid 15/227 104/18866 1.86684E-12 3.54998E-11 1.34098E-11 SLC6A3/HTR2A/OPRM1/CASP3/RELA/PPARG/BCL2L1/CDK5/ICAM1/MTOR/MDM2/BCHE/PRKCG/TRPV1/DRD2 15

BP GO:0097756 negative regulation of blood vessel diameter 14/227 86/18866 1.88225E-12 3.5509E-11 1.34133E-11 PTGS2/CHRM3/HTR2A/SLC6A4/EGFR/AKT1/EDN1/GJA1/ICAM1/CRP/AGTR1/HMGCR/HTR1A/HTR2B 14

BP GO:0006869 lipid transport 26/227 393/18866 1.88226E-12 3.5509E-11 1.34133E-11 RXRA/PON1/PPARG/NOS2/AKT1/EGF/PPARD/IL1B/CYP19A1/SYK/ABCB1/SIRT1/EDN1/PPARA/AGTR1/NR1H3/NFKB1/GM2A/NPC1L1/NR1H4/SHH/PLA2G4A/CETP/ABCG1/REN/DRD2 26

BP GO:0001655 urogenital system development 24/227 330/18866 1.89771E-12 3.55186E-11 1.34169E-11 RXRA/BCL2/BAX/CASP9/AR/VEGFA/MMP9/ODC1/STAT1/ESR1/MYC/GDNF/BDNF/CA2/AKR1B1/CAT/CYP19A1/PECAM1/AGTR1/CFLAR/SHH/REN/TGFBR1/RET 24

BP GO:0071375 cellular response to peptide hormone stimulus 24/227 330/18866 1.89771E-12 3.55186E-11 1.34169E-11 RELA/PPARG/AKT1/STAT1/IL1B/GPT/NFE2L2/CA2/GCLC/PARP1/SIRT1/STAT3/IGF1R/EDN1/FOXO1/PRKCB/AGTR1/CFLAR/NFKB1/MDM2/GSTP1/INSR/CREB1/NR1H4 24

BP GO:0051353 positive regulation of oxidoreductase activity 12/227 55/18866 1.91364E-12 3.56762E-11 1.34764E-11 AKT1/TNF/HIF1A/IL1B/GDNF/SNCA/EDN1/AGTR1/IFNG/VDR/DHFR/HTR2B 12

BP GO:0002688 regulation of leukocyte chemotaxis 16/227 124/18866 1.95607E-12 3.63249E-11 1.37215E-11 DPP4/VEGFA/MAPK1/IL6/MAPK3/CYP19A1/MAPK14/EDN1/CCL2/CXCL8/C5AR1/CCR2/IL4/TRPV4/CCR1/CSF1R 16

BP GO:0009306 protein secretion 28/227 462/18866 2.08486E-12 3.85658E-11 1.4568E-11 OPRM1/PRKCA/TGFB1/DPP4/NOS2/ACHE/EGFR/IL10/TNF/IL6/HIF1A/PPARD/IL1B/TLR4/SYK/GJA1/IL1A/NR1H3/IL2/IFNG/CD40LG/HMGCR/PAM/NR1H4/ALOX5/ABCG1/DRD2/HTR2B 28

BP GO:0042542 response to hydrogen peroxide 17/227 146/18866 2.14427E-12 3.9511E-11 1.4925E-11 BCL2/JUN/CASP3/RELA/IL10/IL6/SOD1/STAT1/HMOX1/NFE2L2/CAT/CDK1/SIRT1/BAK1/FOXO1/MDM2/GPX1 17

BP GO:0035592 establishment of protein localization to extracellular region 28/227 463/18866 2.19606E-12 4.03091E-11 1.52265E-11 OPRM1/PRKCA/TGFB1/DPP4/NOS2/ACHE/EGFR/IL10/TNF/IL6/HIF1A/PPARD/IL1B/TLR4/SYK/GJA1/IL1A/NR1H3/IL2/IFNG/CD40LG/HMGCR/PAM/NR1H4/ALOX5/ABCG1/DRD2/HTR2B 28

BP GO:1904705 regulation of vascular associated smooth muscle cell proliferation 14/227 87/18866 2.21958E-12 4.04286E-11 1.52716E-11 JUN/PPARG/CDKN1A/MMP2/MMP9/IL10/TNF/HMOX1/EDN1/GJA1/SOD2/MDM2/GSTP1/PRKG1 14

BP GO:1990874 vascular associated smooth muscle cell proliferation 14/227 87/18866 2.21958E-12 4.04286E-11 1.52716E-11 JUN/PPARG/CDKN1A/MMP2/MMP9/IL10/TNF/HMOX1/EDN1/GJA1/SOD2/MDM2/GSTP1/PRKG1 14

BP GO:0010631 epithelial cell migration 25/227 365/18866 2.41076E-12 4.37433E-11 1.65237E-11 PTGS2/JUN/PRKCA/TGFB1/PPARG/DPP4/AKT1/VEGFA/MMP9/TNF/HIF1A/KDR/PPARD/HMOX1/NFE2L2/SIRT1/EDN1/PECAM1/MTOR/IFNG/IL4/PRSS3/ENPP2/GPX1/TGFBR1 25

BP GO:0046889 positive regulation of lipid biosynthetic process 14/227 88/18866 2.61166E-12 4.72085E-11 1.78327E-11 PTGS2/HTR2A/AKT1/TNF/IL1B/HTR2C/MTOR/NR1H3/IFNG/CREB1/NR1H4/TNFRSF1A/ABCG1/HTR2B 14

BP GO:0010743 regulation of macrophage derived foam cell differentiation 10/227 32/18866 2.66916E-12 4.80651E-11 1.81563E-11 PPARG/PPARA/CRP/AGTR1/NR1H3/NFKB1/CSF2/CETP/ABCG1/MAPK9 10

BP GO:0014066 regulation of phosphatidylinositol 3-kinase signaling 16/227 127/18866 2.84782E-12 5.10888E-11 1.92984E-11 PIK3CG/EGFR/MAPK1/TNF/KDR/PPARD/MAPK3/NTRK1/NTRK2/CAT/F2/SIRT1/IGF1R/CD28/INSR/PTPN6 16

BP GO:0090132 epithelium migration 25/227 368/18866 2.88681E-12 5.15936E-11 1.94891E-11 PTGS2/JUN/PRKCA/TGFB1/PPARG/DPP4/AKT1/VEGFA/MMP9/TNF/HIF1A/KDR/PPARD/HMOX1/NFE2L2/SIRT1/EDN1/PECAM1/MTOR/IFNG/IL4/PRSS3/ENPP2/GPX1/TGFBR1 25

BP GO:0097529 myeloid leukocyte migration 20/227 222/18866 2.95061E-12 5.25364E-11 1.98452E-11 PIK3CG/DPP4/VEGFA/MAPK1/IL6/IL1B/MAPK3/CYP19A1/SYK/MAPK14/EDN1/CCL2/CXCL8/PECAM1/C5AR1/CCR2/IL4/TRPV4/CCR1/CSF1R 20

BP GO:0050714 positive regulation of protein secretion 18/227 172/18866 2.98465E-12 5.29442E-11 1.99993E-11 TGFB1/ACHE/EGFR/IL10/TNF/IL6/HIF1A/PPARD/TLR4/SYK/GJA1/IL1A/IL2/IFNG/NR1H4/ABCG1/DRD2/HTR2B 18

BP GO:0071692 protein localization to extracellular region 28/227 470/18866 3.14729E-12 5.56216E-11 2.10107E-11 OPRM1/PRKCA/TGFB1/DPP4/NOS2/ACHE/EGFR/IL10/TNF/IL6/HIF1A/PPARD/IL1B/TLR4/SYK/GJA1/IL1A/NR1H3/IL2/IFNG/CD40LG/HMGCR/PAM/NR1H4/ALOX5/ABCG1/DRD2/HTR2B 28

BP GO:1903829 positive regulation of cellular protein localization 24/227 338/18866 3.16546E-12 5.57356E-11 2.10537E-11 PTGS2/BCL2/CASP8/TGFB1/EGFR/AKT1/MAPK1/EGF/TNF/TP53/IL1B/CDK1/CDK5/PARP1/F2/MAPK14/MAPK8/CDC42/MDM2/ERBB2/IFNG/PSEN1/SHH/EIF2AK3 24

BP GO:0090130 tissue migration 25/227 374/18866 4.11756E-12 7.2232E-11 2.72851E-11 PTGS2/JUN/PRKCA/TGFB1/PPARG/DPP4/AKT1/VEGFA/MMP9/TNF/HIF1A/KDR/PPARD/HMOX1/NFE2L2/SIRT1/EDN1/PECAM1/MTOR/IFNG/IL4/PRSS3/ENPP2/GPX1/TGFBR1 25

BP GO:0006801 superoxide metabolic process 13/227 74/18866 4.29891E-12 7.51361E-11 2.83821E-11 TGFB1/NOS2/EGFR/TNF/SOD1/NFE2L2/SYK/MPO/EDN1/SOD2/CRP/GSTP1/DHFR 13

BP GO:0043525 positive regulation of neuron apoptotic process 12/227 59/18866 4.68071E-12 8.15095E-11 3.07896E-11 BAX/CASP9/JUN/CASP3/TNF/TP53/DDIT3/MCL1/CDK5/FASLG/CDC42/ATF2 12

BP GO:0046879 hormone secretion 23/227 314/18866 4.86582E-12 8.44238E-11 3.18905E-11 PRKCA/DPP4/NOS2/EGFR/TNF/IL6/HIF1A/PPARD/IL1B/HTR2C/CYP19A1/EDN1/GJA1/AGTR1/IFNG/TRPV4/HMGCR/CREB1/NR1H4/ALOX5/HTR1A/REN/DRD2 23

BP GO:0048771 tissue remodeling 18/227 178/18866 5.36174E-12 9.26898E-11 3.50129E-11 BAX/PRKCA/TGFB1/EGFR/MMP2/IL6/TP53/HIF1A/CA2/SYK/BAK1/GJA1/IL1A/CCR2/MDM2/IL2/CSF1R/VDR 18

BP GO:0031334 positive regulation of protein-containing complex assembly 21/227 257/18866 5.42845E-12 9.35032E-11 3.53202E-11 BAX/TGFB1/MMP3/VEGFA/TNF/TP53/MMP1/ESR1/TLR4/SNCA/HSP90AA1/SYK/PARP1/BAK1/ICAM1/MTOR/IFNG/VCP/CREB1/CSF2/MAPK9 21

BP GO:2000117 negative regulation of cysteine-type endopeptidase activity 14/227 93/18866 5.71156E-12 9.80244E-11 3.7028E-11 PTGS2/AKT1/VEGFA/MMP9/TNF/SNCA/NGF/BIRC5/XIAP/CFLAR/BIRC3/MDM2/GPX1/BIRC2 14

BP GO:0000187 activation of MAPK activity 17/227 156/18866 6.34399E-12 1.08487E-10 4.09801E-11 CHRNA7/MAPK1/EGF/TNF/SOD1/IL1B/TLR4/MAPK3/CDK1/SYK/MAPK14/C5AR1/INSR/CD40LG/GRM1/MAPK10/RET 17

BP GO:0001938 positive regulation of endothelial cell proliferation 15/227 113/18866 6.43759E-12 1.09693E-10 4.14357E-11 JUN/PRKCA/AKT1/VEGFA/IL10/HIF1A/KDR/HMOX1/SIRT1/STAT3/AGTR1/MTOR/CCR3/TGFBR1/HTR2B 15

BP GO:0033619 membrane protein proteolysis 12/227 61/18866 7.13134E-12 1.20504E-10 4.55194E-11 TGFB1/RELA/IL10/TNF/IL1B/NFKB1/IFNG/PSEN1/PSEN2/TNFRSF1B/RET/TMPRSS6 12

BP GO:1902041 regulation of extrinsic apoptotic signaling pathway via death domain receptors 12/227 61/18866 7.13134E-12 1.20504E-10 4.55194E-11 CASP8/BCL2L1/HMOX1/ATF3/FASLG/ICAM1/BRCA1/TNFRSF10B/CFLAR/TNFSF10/TNFRSF10A/GPX1 12

BP GO:0030574 collagen catabolic process 11/227 47/18866 7.14809E-12 1.20504E-10 4.55194E-11 MMP3/MMP2/MMP9/MMP1/MMP12/MMP7/MMP8/CTSB/MMP10/CTSD/TMPRSS6 11

BP GO:0032368 regulation of lipid transport 16/227 135/18866 7.38887E-12 1.24122E-10 4.68864E-11 PON1/PPARG/AKT1/EGF/IL1B/CYP19A1/SYK/SIRT1/EDN1/AGTR1/NR1H3/NFKB1/SHH/CETP/ABCG1/REN 16

BP GO:0031349 positive regulation of defense response 25/227 385/18866 7.75674E-12 1.29843E-10 4.90474E-11 PTGS2/PIK3CG/RELA/EGFR/TNF/IL6/IL1B/TLR4/SNCA/MAPK3/MMP12/MMP8/SYK/STAT3/GJA1/AGTR1/IL17B/CCR2/CD28/NFKB1/IL2/IFNG/TRPV4/TNFRSF1A/EP300 25

BP GO:0045444 fat cell differentiation 20/227 235/18866 8.44025E-12 1.40297E-10 5.29963E-11 PTGS2/HTR2A/TGFB1/PPARG/AKT1/TNF/IL6/PPARD/DDIT3/HTR2C/MAPK14/SIRT1/FOXO1/MTOR/TRPV4/CREB1/ATF2/GPX1/ALOX5/EP300 20

BP GO:0001558 regulation of cell growth 26/227 420/18866 8.44026E-12 1.40297E-10 5.29963E-11 BCL2/TGFB1/MAP2/PPARG/EGFR/AKT1/VEGFA/CDKN1A/TP53/ESR2/PPARD/BDNF/NGF/CDK5/F2/SIRT1/EDN1/GJA1/PPARA/AGTR1/MTOR/CDC42/ERBB2/IL2/BDKRB1/TGFBR1 26

BP GO:2001235 positive regulation of apoptotic signaling pathway 18/227 183/18866 8.58571E-12 1.42218E-10 5.37217E-11 BCL2/BAX/CASP8/BCL2L1/MMP9/TNF/TP53/SOD1/ATF3/DDIT3/MCL1/SIRT1/MAPK8/BAK1/TNFSF10/MAPK9/TGFBR1/RET 18

BP GO:1902107 positive regulation of leukocyte differentiation 17/227 159/18866 8.6471E-12 1.42737E-10 5.3918E-11 JUN/CASP8/PRKCA/TGFB1/FOS/TNF/IL1B/CA2/SYK/IL1A/CD80/IL2/IFNG/IL4/CREB1/SHH/CCR1 17

BP GO:0009895 negative regulation of catabolic process 23/227 323/18866 8.68036E-12 1.4279E-10 5.3938E-11 PIK3CG/BCL2/RELA/NOS2/EGFR/AKT1/IL10/TNF/TP53/IL1B/HMOX1/GDNF/SNCA/MCL1/MAPK14/STAT3/PPARA/MTOR/PRKCG/HMGCR/PSEN1/SHH/HTR2B 23

BP GO:0006109 regulation of carbohydrate metabolic process 19/227 210/18866 9.78429E-12 1.60395E-10 6.0588E-11 HTR2A/TGFB1/AKT1/EGF/TP53/HIF1A/GPT/SNCA/APP/SIRT1/STAT3/FOXO1/PPARA/MTOR/NFKB1/IFNG/INSR/PSEN1/EP300 19

BP GO:0043467 regulation of generation of precursor metabolites and energy 17/227 162/18866 1.17072E-11 1.91258E-10 7.22465E-11 HTR2A/NOS2/AKT1/TP53/HIF1A/SNCA/APP/CDK1/STAT3/PPARA/MTOR/IFNG/IL4/INSR/VCP/PSEN1/EP300 17

BP GO:0009267 cellular response to starvation 17/227 163/18866 1.29326E-11 2.10022E-10 7.93344E-11 BCL2/JUN/CDKN1A/MAPK1/TP53/NFE2L2/ATF3/MAPK3/SIRT1/MAPK8/FOXO1/PPARA/MTOR/EIF2S1/EIF2AK3/EIF2AK4/EIF2AK2 17

BP GO:0002683 negative regulation of immune system process 27/227 463/18866 1.29441E-11 2.10022E-10 7.93344E-11 CASP3/TGFB1/PPARG/DPP4/AKT1/IL10/TNF/MYC/HMOX1/NFE2L2/TLR4/MMP12/CYP19A1/CDK6/CCL2/CD80/CCR2/NR1H3/ERBB2/IL2/IL4/GNRH1/PTPN6/SHH/GPX1/DRD2/PDE5A 27

BP GO:0046660 female sex differentiation 15/227 119/18866 1.38146E-11 2.23383E-10 8.43812E-11 PGR/BCL2/BAX/CASP3/VEGFA/BCL2L1/SOD1/ESR1/CYP19A1/SIRT1/BAK1/ICAM1/INSR/GNRH1/IDH1 15

BP GO:0034644 cellular response to UV 13/227 81/18866 1.43176E-11 2.30732E-10 8.71573E-11 PTGS2/BAX/CASP9/CDKN1A/TP53/MYC/PARP1/SIRT1/BAK1/EIF2S1/MDM2/EP300/EIF2AK4 13

BP GO:0042176 regulation of protein catabolic process 25/227 397/18866 1.50986E-11 2.42495E-10 9.16009E-11 RELA/NOS2/EGFR/AKT1/IL10/EGF/TNF/ODC1/IL1B/NFE2L2/SNCA/HSP90AA1/GCLC/FOXO1/GJA1/MDM2/IFNG/VCP/PRKCG/HMGCR/PSEN1/SHH/GPX1/TNFRSF1B/MAPK9 25

BP GO:0008585 female gonad development 14/227 100/18866 1.57986E-11 2.52146E-10 9.52465E-11 PGR/BCL2/BAX/CASP3/VEGFA/BCL2L1/SOD1/ESR1/CYP19A1/SIRT1/ICAM1/INSR/GNRH1/IDH1 14

BP GO:1905953 negative regulation of lipid localization 12/227 65/18866 1.58056E-11 2.52146E-10 9.52465E-11 PPARG/AKT1/EGF/TNF/IL6/PPARD/PPARA/CRP/NR1H3/NFKB1/SHH/ABCG1 12

BP GO:0046425 regulation of receptor signaling pathway via JAK-STAT 16/227 142/18866 1.61508E-11 2.56793E-10 9.70017E-11 IL10/EGF/TNF/IL6/AKR1B1/CDK5/F2/STAT3/PECAM1/IL2/IFNG/IL4/CSF2/TNFRSF1A/CSF1R/RET 16

BP GO:0009408 response to heat 17/227 166/18866 1.73598E-11 2.75095E-10 1.03915E-10 PTGS2/AKT1/CDKN1A/MAPK1/SOD1/HMOX1/MAPK3/HSP90AA1/GCLC/SIRT1/IL1A/MTOR/EIF2S1/VCP/TRPV4/TRPV1/EP300 17

BP GO:1902175 regulation of oxidative stress-induced intrinsic apoptotic signaling pathway 9/227 27/18866 1.75126E-11 2.76594E-10 1.04481E-10 AKT1/SOD1/HIF1A/NFE2L2/MCL1/PARP1/SIRT1/SOD2/GPX1 9

BP GO:0045639 positive regulation of myeloid cell differentiation 14/227 101/18866 1.81476E-11 2.85674E-10 1.07911E-10 JUN/CASP8/PRKCA/TGFB1/FOS/TNF/HIF1A/STAT1/CA2/MAPK14/STAT3/IFNG/CREB1/CCR1 14

BP GO:0002697 regulation of immune effector process 27/227 470/18866 1.82536E-11 2.86395E-10 1.08184E-10 TGFB1/NOS2/IL10/TNF/IL6/STAT1/IL1B/HMOX1/TLR4/MMP12/SYK/F2/ICAM1/CD80/C5AR1/CCR2/BIRC3/CD28/IL2/IFNG/IL4/CD40LG/PTPN6/C1R/TNFRSF1B/BIRC2/EIF2AK4 27

BP GO:0045927 positive regulation of growth 21/227 274/18866 1.83707E-11 2.87284E-10 1.08519E-10 SLC6A3/BCL2/EGFR/AKT1/VEGFA/MAPK1/PPARD/BDNF/NGF/CDK1/F2/MAPK14/EDN1/MTOR/CDC42/ERBB2/IL2/INSR/CREB1/DRD2/TGFBR1 21

BP GO:0050435 amyloid-beta metabolic process 11/227 51/18866 1.87386E-11 2.92077E-10 1.1033E-10 CHRNA7/CASP3/RELA/TNF/NTRK2/APP/IFNG/PSEN1/PSEN2/ABCG1/REN 11

BP GO:0034250 positive regulation of cellular amide metabolic process 17/227 167/18866 1.91235E-11 2.97101E-10 1.12228E-10 RXRA/CHRNA7/CASP3/RELA/MAPK1/TNF/IL6/NFE2L2/MAPK3/APP/MTOR/CD28/ERBB2/IFNG/TNFRSF1A/ABCG1/EIF2AK4 17

BP GO:0045471 response to ethanol 15/227 122/18866 1.99053E-11 3.0824E-10 1.16435E-10 SLC6A3/OPRM1/CASP8/SOD1/CAT/CDK1/STAT3/BAK1/ICAM1/IL2/GSTP1/GNRH1/HMGCR/BIRC2/DRD2 15

BP GO:0002793 positive regulation of peptide secretion 18/227 193/18866 2.10743E-11 3.25283E-10 1.22873E-10 TGFB1/ACHE/EGFR/IL10/TNF/IL6/HIF1A/PPARD/TLR4/SYK/GJA1/IL1A/IL2/IFNG/NR1H4/ABCG1/DRD2/HTR2B 18

BP GO:0002761 regulation of myeloid leukocyte differentiation 15/227 123/18866 2.24307E-11 3.451E-10 1.30359E-10 JUN/CASP8/PRKCA/TGFB1/FOS/TNF/MYC/TLR4/CA2/CDK6/MTOR/IFNG/IL4/CREB1/CCR1 15

BP GO:0002696 positive regulation of leukocyte activation 25/227 406/18866 2.44849E-11 3.75487E-10 1.41838E-10 BCL2/TGFB1/DPP4/AKT1/CDKN1A/IL10/IL6/IL1B/TLR4/MMP8/SYK/CCL2/VCAM1/IL1A/CD80/CCR2/CDC42/CD28/IL2/IFNG/IL4/CD40LG/PTPN6/SHH/PLA2G4A 25

BP GO:0022612 gland morphogenesis 15/227 124/18866 2.52484E-11 3.85951E-10 1.4579E-10 PGR/RXRA/BCL2/BAX/TGFB1/AR/EGFR/TNF/IL6/ESR1/CFLAR/CDC42/SHH/CSF1R/VDR 15

BP GO:0010575 positive regulation of vascular endothelial growth factor production 9/227 28/18866 2.55398E-11 3.87911E-10 1.4653E-10 PTGS2/TGFB1/IL6/HIF1A/IL1B/BRCA1/IL1A/C5AR1/EIF2AK3 9

BP GO:0032800 receptor biosynthetic process 9/227 28/18866 2.55398E-11 3.87911E-10 1.4653E-10 PPARG/ACHE/IL10/TNF/HIF1A/EDN1/PPARA/NR1H3/IFNG 9

BP GO:0007548 sex differentiation 21/227 280/18866 2.76485E-11 4.17524E-10 1.57717E-10 PGR/BCL2/BAX/CASP3/AR/VEGFA/BCL2L1/SOD1/ESR1/NTRK1/CYP19A1/SIRT1/BAK1/ICAM1/TNFSF10/INSR/GNRH1/SHH/REN/IDH1/TGFBR1 21

BP GO:0042391 regulation of membrane potential 26/227 443/18866 2.76651E-11 4.17524E-10 1.57717E-10 CHRNA7/OPRM1/BCL2/BAX/JUN/AKT1/BCL2L1/SOD1/KDR/BDNF/NTRK2/APP/GCLC/CDK5/PARP1/BAK1/EDN1/GJA1/GABBR1/GRM1/VCP/KCNMA1/PSEN1/TRPV1/SCN9A/DRD2 26

BP GO:0008406 gonad development 19/227 223/18866 2.8072E-11 4.22324E-10 1.5953E-10 PGR/BCL2/BAX/CASP3/AR/VEGFA/BCL2L1/SOD1/ESR1/NTRK1/CYP19A1/SIRT1/ICAM1/TNFSF10/INSR/GNRH1/REN/IDH1/TGFBR1 19

BP GO:0008637 apoptotic mitochondrial changes 15/227 125/18866 2.83887E-11 4.24402E-10 1.60315E-10 BCL2/BAX/JUN/CASP8/AKT1/BCL2L1/MMP9/TP53/GCLC/MAPK8/BAK1/SOD2/TNFSF10/ATF2/GPX1 15

BP GO:1903578 regulation of ATP metabolic process 15/227 125/18866 2.83887E-11 4.24402E-10 1.60315E-10 HTR2A/TP53/HIF1A/SNCA/APP/CDK1/PARP1/STAT3/PPARA/IFNG/IL4/INSR/VCP/PSEN1/EP300 15

BP GO:0046545 development of primary female sexual characteristics 14/227 105/18866 3.11105E-11 4.63634E-10 1.75134E-10 PGR/BCL2/BAX/CASP3/VEGFA/BCL2L1/SOD1/ESR1/CYP19A1/SIRT1/ICAM1/INSR/GNRH1/IDH1 14

BP GO:1902895 positive regulation of pri-miRNA transcription by RNA polymerase II 10/227 40/18866 3.22443E-11 4.7903E-10 1.8095E-10 JUN/TGFB1/RELA/PPARG/FOS/IL10/TNF/TP53/HIF1A/STAT3 10

BP GO:0014074 response to purine-containing compound 16/227 149/18866 3.37984E-11 5.00554E-10 1.89081E-10 PTGS2/SLC6A3/PIK3CG/SLC6A4/RELA/PPARG/FOS/SOD1/STAT1/IL1B/APP/TYR/ALDH3A1/TRPV1/BIRC2/REN 16

BP GO:0051051 negative regulation of transport 27/227 483/18866 3.3987E-11 5.01783E-10 1.89545E-10 PTGS2/HTR2A/OPRM1/BCL2/AKT1/MMP9/EGF/TNF/IL1B/HMOX1/SNCA/GCLC/CDK5/EDN1/GJA1/ICAM1/PRKCB/MTOR/CCR2/NR1H3/NFKB1/HMGCR/SHH/TNFRSF1A/TNFRSF1B/HRH3/DRD2 27

BP GO:0042326 negative regulation of phosphorylation 27/227 484/18866 3.56205E-11 5.24272E-10 1.9804E-10 BAX/JUN/CASP3/TGFB1/AKT1/CDKN1A/XDH/MYC/IL1B/TLR4/ATF3/SNCA/SIRT1/STAT3/BAK1/IGF1R/FOXO1/PPARA/MTOR/IL2/IFNG/GSTP1/HMGCR/PSEN1/PTPN6/BDKRB1/DRD2 27

BP GO:0010506 regulation of autophagy 23/227 347/18866 3.70994E-11 5.44354E-10 2.05626E-10 BCL2/CASP3/AKT1/IL10/TP53/HIF1A/KDR/HMOX1/DDIT3/SNCA/MAPK3/MCL1/CDK5/SIRT1/STAT3/MAPK8/FOXO1/MTOR/IFNG/IL4/EP300/HTR2B/EIF2AK4 23

BP GO:0043254 regulation of protein-containing complex assembly 26/227 449/18866 3.72363E-11 5.44682E-10 2.0575E-10 BAX/TGFB1/MAP2/MMP3/VEGFA/TNF/TP53/MMP1/ESR1/TLR4/SNCA/HSP90AA1/SYK/PARP1/BAK1/ICAM1/MTOR/CDC42/IFNG/VCP/CREB1/CSF2/BIRC2/EP300/MAPK9/EIF2AK2 26

BP GO:0043312 neutrophil degranulation 27/227 487/18866 4.09773E-11 5.97565E-10 2.25726E-10 PLAU/MMP9/MAPK1/HSP90AA1/MMP8/CAT/MGAM/FUCA1/SYK/TTR/MPO/MAPK14/PECAM1/C5AR1/NFKB1/GSTP1/VCP/PRSS3/GM2A/PSEN1/CTSB/PTPN6/ALOX5/GLB1/TNFRSF1B/CTSD/IDH1 27

BP GO:1904892 regulation of receptor signaling pathway via STAT 16/227 151/18866 4.143E-11 6.02318E-10 2.27521E-10 IL10/EGF/TNF/IL6/AKR1B1/CDK5/F2/STAT3/PECAM1/IL2/IFNG/IL4/CSF2/TNFRSF1A/CSF1R/RET 16

BP GO:0014068 positive regulation of phosphatidylinositol 3-kinase signaling 13/227 88/18866 4.25219E-11 6.16308E-10 2.32806E-10 PIK3CG/TNF/KDR/PPARD/NTRK1/NTRK2/CAT/F2/SIRT1/IGF1R/CD28/INSR/PTPN6 13

BP GO:0045137 development of primary sexual characteristics 19/227 229/18866 4.45662E-11 6.42023E-10 2.4252E-10 PGR/BCL2/BAX/CASP3/AR/VEGFA/BCL2L1/SOD1/ESR1/NTRK1/CYP19A1/SIRT1/ICAM1/TNFSF10/INSR/GNRH1/REN/IDH1/TGFBR1 19

BP GO:0050920 regulation of chemotaxis 19/227 229/18866 4.45662E-11 6.42023E-10 2.4252E-10 TGFB1/DPP4/VEGFA/MAPK1/IL6/KDR/MAPK3/CYP19A1/MAPK14/EDN1/CCL2/CXCL8/C5AR1/CCR2/IL4/GSTP1/TRPV4/CCR1/CSF1R 19

BP GO:0002283 neutrophil activation involved in immune response 27/227 490/18866 4.70886E-11 6.76312E-10 2.55472E-10 PLAU/MMP9/MAPK1/HSP90AA1/MMP8/CAT/MGAM/FUCA1/SYK/TTR/MPO/MAPK14/PECAM1/C5AR1/NFKB1/GSTP1/VCP/PRSS3/GM2A/PSEN1/CTSB/PTPN6/ALOX5/GLB1/TNFRSF1B/CTSD/IDH1 27

BP GO:0060401 cytosolic calcium ion transport 17/227 178/18866 5.3118E-11 7.60226E-10 2.8717E-10 HTR2A/BCL2/BAX/DDIT3/SNCA/HTR2C/F2/FASLG/BAK1/TRPV4/PSEN2/PTPN6/TRPV1/BDKRB1/CCR5/DRD2/HTR2B 17

BP GO:0050867 positive regulation of cell activation 25/227 421/18866 5.3251E-11 7.60226E-10 2.8717E-10 BCL2/TGFB1/DPP4/AKT1/CDKN1A/IL10/IL6/IL1B/TLR4/MMP8/SYK/CCL2/VCAM1/IL1A/CD80/CCR2/CDC42/CD28/IL2/IFNG/IL4/CD40LG/PTPN6/SHH/PLA2G4A 25

BP GO:0010623 programmed cell death involved in cell development 7/227 13/18866 5.37193E-11 7.64615E-10 2.88828E-10 BCL2/BAX/IL1B/BDNF/NTRK1/FASLG/IL1A 7

BP GO:0046427 positive regulation of receptor signaling pathway via JAK-STAT 13/227 90/18866 5.69827E-11 8.08644E-10 3.05459E-10 IL10/TNF/IL6/AKR1B1/F2/STAT3/PECAM1/IL2/IFNG/IL4/CSF2/TNFRSF1A/CSF1R 13

BP GO:0034976 response to endoplasmic reticulum stress 21/227 294/18866 6.89799E-11 9.75983E-10 3.68671E-10 BCL2/BAX/JUN/BCL2L1/TP53/NFE2L2/ATF3/DDIT3/SIRT1/BAK1/CCL2/CXCL8/TNFRSF10B/EIF2S1/NR1H3/VCP/ALOX5/EIF2AK3/EP300/EIF2AK4/EIF2AK2 21

BP GO:0071453 cellular response to oxygen levels 19/227 235/18866 6.97563E-11 9.84039E-10 3.71714E-10 PTGS2/BCL2/PPARG/AKT1/VEGFA/TP53/HIF1A/PPARD/MYC/HMOX1/NFE2L2/SIRT1/EDN1/FOXO1/ICAM1/CFLAR/MTOR/MDM2/EP300 19

BP GO:0008631 intrinsic apoptotic signaling pathway in response to oxidative stress 10/227 43/18866 7.06715E-11 9.94001E-10 3.75477E-10 BCL2/AKT1/SOD1/HIF1A/NFE2L2/MCL1/PARP1/SIRT1/SOD2/GPX1 10

BP GO:0032770 positive regulation of monooxygenase activity 9/227 31/18866 7.22462E-11 1.01315E-09 3.82711E-10 AKT1/TNF/HIF1A/IL1B/GDNF/IFNG/VDR/DHFR/HTR2B 9

BP GO:0090316 positive regulation of intracellular protein transport 17/227 182/18866 7.56256E-11 1.05742E-09 3.99434E-10 PTGS2/BCL2/CASP8/TGFB1/MAPK1/TP53/IL1B/CDK1/CDK5/MAPK14/MAPK8/CDC42/MDM2/ERBB2/IFNG/PSEN1/SHH 17

BP GO:0046683 response to organophosphorus 15/227 134/18866 7.78246E-11 1.08498E-09 4.09844E-10 PTGS2/SLC6A3/PIK3CG/SLC6A4/RELA/FOS/SOD1/STAT1/IL1B/APP/TYR/ALDH3A1/TRPV1/BIRC2/REN 15

BP GO:0060402 calcium ion transport into cytosol 16/227 158/18866 8.24809E-11 1.14653E-09 4.33094E-10 HTR2A/BCL2/BAX/DDIT3/SNCA/HTR2C/F2/FASLG/BAK1/TRPV4/PTPN6/TRPV1/BDKRB1/CCR5/DRD2/HTR2B 16

BP GO:1904894 positive regulation of receptor signaling pathway via STAT 13/227 93/18866 8.7186E-11 1.2084E-09 4.56466E-10 IL10/TNF/IL6/AKR1B1/F2/STAT3/PECAM1/IL2/IFNG/IL4/CSF2/TNFRSF1A/CSF1R 13

BP GO:0003012 muscle system process 26/227 467/18866 8.81969E-11 1.21886E-09 4.60416E-10 PTGS2/CHRM3/HTR2A/PIK3CG/PRKCA/SOD1/IL1B/HMOX1/GDNF/PARP1/EDN1/FOXO1/GJA1/PPARA/CFLAR/MTOR/KCNMA1/GAMT/TRPV1/TNFRSF1A/TNFRSF1B/PRKG1/TACR1/DRD2/PDE5A/HTR2B 26

BP GO:0050671 positive regulation of lymphocyte proliferation 15/227 136/18866 9.63411E-11 1.32755E-09 5.01473E-10 BCL2/CDKN1A/IL6/IL1B/TLR4/SYK/VCAM1/IL1A/CD80/CCR2/CD28/IL2/IL4/CD40LG/SHH 15

BP GO:1904035 regulation of epithelial cell apoptotic process 13/227 94/18866 1.00113E-10 1.37554E-09 5.19601E-10 TNF/IL6/KDR/HMOX1/NFE2L2/FASLG/ICAM1/CCL2/CFLAR/MTOR/EIF2S1/IL4/CD40LG 13

BP GO:0032946 positive regulation of mononuclear cell proliferation 15/227 137/18866 1.07045E-10 1.46655E-09 5.53978E-10 BCL2/CDKN1A/IL6/IL1B/TLR4/SYK/VCAM1/IL1A/CD80/CCR2/CD28/IL2/IL4/CD40LG/SHH 15

BP GO:0010888 negative regulation of lipid storage 8/227 22/18866 1.07405E-10 1.46726E-09 5.54245E-10 PPARG/TNF/IL6/PPARD/PPARA/CRP/NR1H3/ABCG1 8

BP GO:0033555 multicellular organismal response to stress 12/227 76/18866 1.0837E-10 1.47619E-09 5.57621E-10 BCL2/DPP4/BDNF/NTRK1/HTR2C/GJA1/PRKCG/TRPV1/HTR1A/TACR1/SCN9A/RET 12

BP GO:0014002 astrocyte development 10/227 45/18866 1.15144E-10 1.55953E-09 5.891E-10 EGFR/TNF/IL6/IL1B/TLR4/APP/CDK6/C5AR1/IFNG/PSEN1 10

BP GO:0034198 cellular response to amino acid starvation 10/227 45/18866 1.15144E-10 1.55953E-09 5.891E-10 CDKN1A/MAPK1/ATF3/MAPK3/MAPK8/MTOR/EIF2S1/EIF2AK3/EIF2AK4/EIF2AK2 10

BP GO:0050870 positive regulation of T cell activation 18/227 214/18866 1.17598E-10 1.58824E-09 5.99947E-10 DPP4/AKT1/IL6/IL1B/SYK/CCL2/VCAM1/IL1A/CD80/CCR2/CDC42/CD28/IL2/IFNG/IL4/CD40LG/PTPN6/SHH 18

BP GO:0001836 release of cytochrome c from mitochondria 11/227 60/18866 1.2263E-10 1.65151E-09 6.23845E-10 BCL2/BAX/JUN/AKT1/BCL2L1/MMP9/TP53/BAK1/SOD2/TNFSF10/GPX1 11

BP GO:0045861 negative regulation of proteolysis 23/227 369/18866 1.26607E-10 1.70026E-09 6.4226E-10 PTGS2/AKT1/VEGFA/MMP9/IL10/TNF/TP53/SNCA/NGF/APP/CDK5/F2/BIRC5/PLAT/XIAP/CFLAR/BIRC3/MDM2/PRKCG/PSEN1/SHH/GPX1/BIRC2 23

BP GO:1903201 regulation of oxidative stress-induced cell death 12/227 77/18866 1.27027E-10 1.70109E-09 6.42574E-10 MMP3/AKT1/IL10/SOD1/HIF1A/NFE2L2/TLR4/MCL1/PARP1/SIRT1/SOD2/GPX1 12

BP GO:0002690 positive regulation of leukocyte chemotaxis 13/227 96/18866 1.3134E-10 1.7539E-09 6.62524E-10 VEGFA/MAPK1/IL6/MAPK3/MAPK14/EDN1/CXCL8/C5AR1/CCR2/IL4/TRPV4/CCR1/CSF1R 13

BP GO:0042310 vasoconstriction 12/227 78/18866 1.48544E-10 1.9781E-09 7.47211E-10 PTGS2/CHRM3/HTR2A/SLC6A4/EGFR/AKT1/EDN1/GJA1/ICAM1/AGTR1/HTR1A/HTR2B 12

BP GO:0036473 cell death in response to oxidative stress 13/227 97/18866 1.50069E-10 1.98726E-09 7.50674E-10 BCL2/MMP3/AKT1/IL10/SOD1/HIF1A/NFE2L2/TLR4/MCL1/PARP1/SIRT1/SOD2/GPX1 13

BP GO:0071674 mononuclear cell migration 13/227 97/18866 1.50069E-10 1.98726E-09 7.50674E-10 MAPK1/TNF/IL6/MAPK3/MAPK14/CCL2/PECAM1/C5AR1/CCR2/IL4/TRPV4/CCR1/CSF1R 13

BP GO:0035265 organ growth 17/227 191/18866 1.62121E-10 2.14089E-09 8.08707E-10 RXRA/SLC6A4/BCL2/AR/AKT1/MAPK1/SOD1/ESR1/CDK1/CYP19A1/MAPK14/EDN1/GJA1/PPARA/MTOR/SHH/TGFBR1 17

BP GO:0001933 negative regulation of protein phosphorylation 25/227 444/18866 1.64289E-10 2.16352E-09 8.17254E-10 BAX/JUN/CASP3/TGFB1/AKT1/CDKN1A/XDH/MYC/IL1B/TLR4/ATF3/SNCA/SIRT1/BAK1/IGF1R/FOXO1/MTOR/IL2/IFNG/GSTP1/HMGCR/PSEN1/PTPN6/BDKRB1/DRD2 25

BP GO:1901222 regulation of NIK/NF-kappaB signaling 14/227 119/18866 1.72853E-10 2.27001E-09 8.57479E-10 RELA/EGFR/TNF/IL1B/TLR4/APP/MMP8/EDN1/TNFRSF10B/TNFRSF10A/LITAF/BIRC2/EP300/EIF2AK2 14

BP GO:0050769 positive regulation of neurogenesis 26/227 485/18866 2.00519E-10 2.62608E-09 9.91982E-10 OPRM1/BCL2/TGFB1/RELA/PPARG/VEGFA/TNF/IL6/HIF1A/IL1B/NFE2L2/BDNF/NGF/NTRK1/NTRK2/CFLAR/MTOR/CCR2/IL2/IFNG/PSEN1/SHH/TNFRSF1B/EP300/DRD2/RET 26

BP GO:1900182 positive regulation of protein localization to nucleus 12/227 80/18866 2.01748E-10 2.6277E-09 9.92594E-10 PTGS2/TGFB1/AKT1/MAPK1/CDK1/PARP1/F2/MAPK14/IFNG/PSEN1/SHH/EIF2AK3 12

BP GO:1901224 positive regulation of NIK/NF-kappaB signaling 12/227 80/18866 2.01748E-10 2.6277E-09 9.92594E-10 RELA/EGFR/TNF/IL1B/TLR4/APP/MMP8/EDN1/TNFRSF10B/TNFRSF10A/EP300/EIF2AK2 12

BP GO:0032388 positive regulation of intracellular transport 18/227 222/18866 2.14938E-10 2.79185E-09 1.0546E-09 PTGS2/BCL2/CASP8/TGFB1/MAP2/MAPK1/TP53/IL1B/CDK1/CDK5/MAPK14/MAPK8/CDC42/MDM2/ERBB2/IFNG/PSEN1/SHH 18

BP GO:0010212 response to ionizing radiation 15/227 144/18866 2.18445E-10 2.82198E-09 1.06598E-09 BCL2/BAX/CASP3/BCL2L1/CDKN1A/TP53/MYC/PARP1/MAPK14/SIRT1/BAK1/ICAM1/BRCA1/MDM2/GPX1 15

BP GO:0050921 positive regulation of chemotaxis 15/227 144/18866 2.18445E-10 2.82198E-09 1.06598E-09 TGFB1/VEGFA/MAPK1/IL6/KDR/MAPK3/MAPK14/EDN1/CXCL8/C5AR1/CCR2/IL4/TRPV4/CCR1/CSF1R 15

BP GO:1990928 response to amino acid starvation 10/227 48/18866 2.28754E-10 2.93917E-09 1.11025E-09 CDKN1A/MAPK1/ATF3/MAPK3/MAPK8/MTOR/EIF2S1/EIF2AK3/EIF2AK4/EIF2AK2 10

BP GO:2001239 regulation of extrinsic apoptotic signaling pathway in absence of ligand 10/227 48/18866 2.28754E-10 2.93917E-09 1.11025E-09 BCL2/AKT1/BCL2L1/TNF/IL1B/GDNF/MCL1/IL1A/CSF2/RET 10

BP GO:0016485 protein processing 18/227 223/18866 2.31346E-10 2.96447E-09 1.11981E-09 PLAU/CASP3/CASP8/NGF/PARP1/BAK1/PLAT/XIAP/BIRC3/MDM2/PRSS3/PSEN1/PSEN2/CASP1/SHH/C1R/BIRC2/REN 18

BP GO:0019915 lipid storage 12/227 81/18866 2.34344E-10 2.99482E-09 1.13127E-09 PPARG/TNF/IL6/PPARD/IL1B/SIRT1/PPARA/CRP/NR1H3/NFKB1/GM2A/ABCG1 12

BP GO:0001822 kidney development 20/227 283/18866 2.41564E-10 3.07881E-09 1.163E-09 BCL2/BAX/CASP9/VEGFA/MMP9/ODC1/STAT1/MYC/GDNF/BDNF/CA2/AKR1B1/CAT/PECAM1/AGTR1/CFLAR/SHH/REN/TGFBR1/RET 20

BP GO:0050728 negative regulation of inflammatory response 17/227 196/18866 2.43202E-10 3.0914E-09 1.16775E-09 PPARG/IL10/SOD1/PPARD/CYP19A1/F2/PPARA/NR1H3/NFKB1/IL2/IL4/GSTP1/NR1H4/GPX1/TNFRSF1A/ALOX5/TNFRSF1B 17

BP GO:0022617 extracellular matrix disassembly 12/227 82/18866 2.71632E-10 3.44356E-09 1.30078E-09 TGFB1/DPP4/MMP3/MMP2/MMP9/IL6/MMP1/MMP12/MMP7/MMP8/MMP10/TMPRSS6 12

BP GO:0019932 second-messenger-mediated signaling 25/227 456/18866 2.87349E-10 3.63313E-09 1.37239E-09 CHRM3/OPRM1/PRKCA/NOS2/EGFR/VEGFA/TNF/KDR/HTR2C/SYK/EDN1/SELE/VCAM1/CXCL8/AGTR1/MTOR/CCR2/CCR1/CCR3/PRKG1/EIF2AK3/CCR5/DRD2/PDE5A/HTR2B 25

BP GO:0051101 regulation of DNA binding 14/227 124/18866 3.01565E-10 3.80275E-09 1.43646E-09 JUN/TGFB1/PPARG/MMP9/EGF/HMOX1/DDIT3/NGF/MMP8/PARP1/MAPK8/IFNG/PSEN1/EP300 14

BP GO:0010573 vascular endothelial growth factor production 11/227 65/18866 3.03842E-10 3.82134E-09 1.44348E-09 PTGS2/TGFB1/TNF/IL6/HIF1A/IL1B/BRCA1/IL1A/C5AR1/CCR2/EIF2AK3 11

BP GO:0001893 maternal placenta development 9/227 36/18866 3.20209E-10 3.99547E-09 1.50926E-09 RXRA/PTGS2/AKT1/MAPK1/PPARD/MAPK3/GJA1/CTSB/VDR 9

BP GO:1901099 negative regulation of signal transduction in absence of ligand 9/227 36/18866 3.20209E-10 3.99547E-09 1.50926E-09 BCL2/AKT1/BCL2L1/TNF/IL1B/GDNF/MCL1/IL1A/CSF2 9

BP GO:2001240 negative regulation of extrinsic apoptotic signaling pathway in absence of ligand 9/227 36/18866 3.20209E-10 3.99547E-09 1.50926E-09 BCL2/AKT1/BCL2L1/TNF/IL1B/GDNF/MCL1/IL1A/CSF2 9

BP GO:0034605 cellular response to heat 14/227 125/18866 3.36019E-10 4.17085E-09 1.57551E-09 PTGS2/CDKN1A/MAPK1/HMOX1/MAPK3/HSP90AA1/SIRT1/IL1A/MTOR/EIF2S1/VCP/TRPV4/TRPV1/EP300 14

BP GO:0035270 endocrine system development 14/227 125/18866 3.36019E-10 4.17085E-09 1.57551E-09 SLC6A3/AKT1/MAPK1/IL6/MAPK3/CDK6/BAK1/FOXO1/INSR/CREB1/SHH/EIF2AK3/DRD2/TGFBR1 14

BP GO:1901617 organic hydroxy compound biosynthetic process 19/227 258/18866 3.45117E-10 4.27262E-09 1.61395E-09 SLC6A3/TNF/SOD1/IL1B/SNCA/AKR1B1/TYR/CYP19A1/SIRT1/NFKB1/IFNG/CYP3A4/HMGCR/NPC1L1/NR1H4/ABCG1/FDFT1/VDR/DHFR 19

BP GO:0001774 microglial cell activation 10/227 50/18866 3.51795E-10 4.28829E-09 1.61987E-09 JUN/TNF/IL6/SNCA/APP/MMP8/C5AR1/IFNG/IL4/TRPV1 10

BP GO:0002269 leukocyte activation involved in inflammatory response 10/227 50/18866 3.51795E-10 4.28829E-09 1.61987E-09 JUN/TNF/IL6/SNCA/APP/MMP8/C5AR1/IFNG/IL4/TRPV1 10

BP GO:0042987 amyloid precursor protein catabolic process 10/227 50/18866 3.51795E-10 4.28829E-09 1.61987E-09 CHRNA7/CASP3/RELA/TNF/NTRK2/APP/IFNG/PSEN1/PSEN2/ABCG1 10

BP GO:0045981 positive regulation of nucleotide metabolic process 10/227 50/18866 3.51795E-10 4.28829E-09 1.61987E-09 HTR2A/NOS2/HIF1A/APP/STAT3/IFNG/IL4/INSR/VCP/PSEN1 10

BP GO:1900544 positive regulation of purine nucleotide metabolic process 10/227 50/18866 3.51795E-10 4.28829E-09 1.61987E-09 HTR2A/NOS2/HIF1A/APP/STAT3/IFNG/IL4/INSR/VCP/PSEN1 10

BP GO:1903580 positive regulation of ATP metabolic process 10/227 50/18866 3.51795E-10 4.28829E-09 1.61987E-09 HTR2A/HIF1A/APP/CDK1/STAT3/IFNG/IL4/INSR/VCP/PSEN1 10

BP GO:0046824 positive regulation of nucleocytoplasmic transport 11/227 66/18866 3.60788E-10 4.38666E-09 1.65703E-09 PTGS2/TGFB1/MAPK1/TP53/IL1B/CDK1/MAPK14/MDM2/IFNG/PSEN1/SHH 11

BP GO:0097530 granulocyte migration 15/227 150/18866 3.90128E-10 4.73129E-09 1.78721E-09 PIK3CG/DPP4/MAPK1/IL1B/MAPK3/SYK/MAPK14/EDN1/CCL2/CXCL8/PECAM1/C5AR1/IL4/TRPV4/CSF1R 15

BP GO:0051251 positive regulation of lymphocyte activation 22/227 357/18866 4.05716E-10 4.90782E-09 1.85389E-09 BCL2/TGFB1/DPP4/AKT1/CDKN1A/IL6/IL1B/TLR4/SYK/CCL2/VCAM1/IL1A/CD80/CCR2/CDC42/CD28/IL2/IFNG/IL4/CD40LG/PTPN6/SHH 22

BP GO:0071621 granulocyte chemotaxis 14/227 127/18866 4.15945E-10 5.007E-09 1.89136E-09 PIK3CG/DPP4/MAPK1/IL1B/MAPK3/SYK/MAPK14/EDN1/CCL2/CXCL8/C5AR1/IL4/TRPV4/CSF1R 14

BP GO:0010634 positive regulation of epithelial cell migration 16/227 176/18866 4.16021E-10 5.007E-09 1.89136E-09 PTGS2/JUN/PRKCA/TGFB1/AKT1/VEGFA/MMP9/HIF1A/KDR/HMOX1/NFE2L2/SIRT1/EDN1/MTOR/IFNG/ENPP2 16

BP GO:0006970 response to osmotic stress 12/227 85/18866 4.17901E-10 5.01413E-09 1.89405E-09 PTGS2/BAX/CASP3/EGFR/TNF/TP53/AKR1B1/ABCB1/KCNMA1/TRPV4/ATF2/MAPK10 12

BP GO:0090322 regulation of superoxide metabolic process 9/227 37/18866 4.18738E-10 5.01413E-09 1.89405E-09 TGFB1/EGFR/TNF/SOD1/NFE2L2/SYK/CRP/GSTP1/DHFR 9

BP GO:0072001 renal system development 20/227 292/18866 4.19777E-10 5.01413E-09 1.89405E-09 BCL2/BAX/CASP9/VEGFA/MMP9/ODC1/STAT1/MYC/GDNF/BDNF/CA2/AKR1B1/CAT/PECAM1/AGTR1/CFLAR/SHH/REN/TGFBR1/RET 20

BP GO:0061041 regulation of wound healing 15/227 151/18866 4.28587E-10 5.10652E-09 1.92895E-09 PLAU/PRKCA/TNF/NFE2L2/TLR4/SYK/F2/EDN1/GJA1/PLAT/MTOR/HMGCR/ALOX5/PLA2G4A/PRKG1 15

BP GO:0071675 regulation of mononuclear cell migration 10/227 51/18866 4.33028E-10 5.14653E-09 1.94407E-09 MAPK1/TNF/MAPK3/MAPK14/C5AR1/CCR2/IL4/TRPV4/CCR1/CSF1R 10

BP GO:0030198 extracellular matrix organization 23/227 395/18866 4.83028E-10 5.72647E-09 2.16313E-09 TGFB1/DPP4/MMP3/MMP2/MMP9/TNF/IL6/MMP1/KDR/MMP12/APP/MMP7/MMP8/TTR/ICAM1/VCAM1/PECAM1/CFLAR/MMP10/TNFRSF1A/TNFRSF1B/TGFBR1/TMPRSS6 23

BP GO:0051897 positive regulation of protein kinase B signaling 16/227 178/18866 4.91989E-10 5.81819E-09 2.19778E-09 PIK3CG/TGFB1/EGFR/EGF/TNF/ESR1/HSP90AA1/IGF1R/CD80/MTOR/CD28/ERBB2/INSR/GPX1/TGFBR1/RET 16

BP GO:0032642 regulation of chemokine production 11/227 68/18866 5.04217E-10 5.94801E-09 2.24682E-09 IL10/TNF/IL6/HIF1A/IL1B/TLR4/SYK/GSTP1/TRPV4/NR1H4/EIF2AK2 11

BP GO:0007050 cell cycle arrest 18/227 234/18866 5.06749E-10 5.95639E-09 2.24999E-09 BAX/TGFB1/CDKN1A/TP53/MYC/DDIT3/CDK1/CDK5/CDK6/CXCL8/BRCA1/CDK7/MTOR/MDM2/IFNG/EP300/TGFBR1/EIF2AK4 18

BP GO:0043062 extracellular structure organization 23/227 396/18866 5.07434E-10 5.95639E-09 2.24999E-09 TGFB1/DPP4/MMP3/MMP2/MMP9/TNF/IL6/MMP1/KDR/MMP12/APP/MMP7/MMP8/TTR/ICAM1/VCAM1/PECAM1/CFLAR/MMP10/TNFRSF1A/TNFRSF1B/TGFBR1/TMPRSS6 23

BP GO:0042113 B cell activation 21/227 328/18866 5.18071E-10 6.06628E-09 2.29149E-09 BCL2/BAX/CASP3/CASP8/TGFB1/CDKN1A/IL10/IL6/TP53/TLR4/NTRK1/SYK/BAK1/VCAM1/PRKCB/CD28/IL2/IL4/CD40LG/PTPN6/EP300 21

BP GO:0000186 activation of MAPKK activity 10/227 52/18866 5.3053E-10 6.1666E-09 2.32939E-09 EGFR/MAPK1/EGF/MAPK3/NGF/NTRK1/GRM1/PSEN1/TGFBR1/EIF2AK2 10

BP GO:0010883 regulation of lipid storage 10/227 52/18866 5.3053E-10 6.1666E-09 2.32939E-09 PPARG/TNF/IL6/PPARD/SIRT1/PPARA/CRP/NR1H3/NFKB1/ABCG1 10

BP GO:0071622 regulation of granulocyte chemotaxis 10/227 52/18866 5.3053E-10 6.1666E-09 2.32939E-09 DPP4/MAPK1/MAPK3/MAPK14/EDN1/CXCL8/C5AR1/IL4/TRPV4/CSF1R 10

BP GO:0071887 leukocyte apoptotic process 13/227 108/18866 5.90134E-10 6.84267E-09 2.58477E-09 BAX/CASP9/CASP3/AKT1/IL10/IL6/TP53/HIF1A/FASLG/SIRT1/BAK1/IL2/CCR5 13

BP GO:0071456 cellular response to hypoxia 17/227 208/18866 6.13508E-10 7.09639E-09 2.68061E-09 PTGS2/BCL2/AKT1/VEGFA/TP53/HIF1A/PPARD/MYC/HMOX1/NFE2L2/SIRT1/EDN1/ICAM1/CFLAR/MTOR/MDM2/EP300 17

BP GO:0034205 amyloid-beta formation 9/227 39/18866 6.98558E-10 8.04167E-09 3.03768E-09 CHRNA7/CASP3/RELA/TNF/NTRK2/APP/IFNG/PSEN1/ABCG1 9

BP GO:0031348 negative regulation of defense response 19/227 269/18866 6.98614E-10 8.04167E-09 3.03768E-09 PPARG/IL10/SOD1/PPARD/MMP12/CYP19A1/F2/PPARA/NR1H3/NFKB1/IL2/IL4/GSTP1/NR1H4/GPX1/TNFRSF1A/ALOX5/TNFRSF1B/DRD2 19

BP GO:0048143 astrocyte activation 8/227 27/18866 7.08035E-10 8.13043E-09 3.07121E-09 EGFR/TNF/IL6/IL1B/APP/C5AR1/IFNG/PSEN1 8

BP GO:1903034 regulation of response to wounding 16/227 183/18866 7.41155E-10 8.49024E-09 3.20713E-09 PLAU/PRKCA/IL10/TNF/NFE2L2/TLR4/SYK/F2/EDN1/GJA1/PLAT/MTOR/HMGCR/ALOX5/PLA2G4A/PRKG1 16

BP GO:0043393 regulation of protein binding 17/227 211/18866 7.6558E-10 8.74896E-09 3.30486E-09 BAX/MAP2/AKT1/CDKN1A/MMP9/IL10/BDNF/MAPK3/APP/CDK5/MAPK8/PPARA/EIF2S1/CDC42/PSEN1/EP300/TGFBR1 17

BP GO:0038061 NIK/NF-kappaB signaling 16/227 184/18866 8.03176E-10 9.1566E-09 3.45884E-09 RELA/EGFR/AKT1/TNF/IL1B/TLR4/APP/MMP8/EDN1/TNFRSF10B/TNFRSF10A/BIRC3/LITAF/BIRC2/EP300/EIF2AK2 16

BP GO:0042982 amyloid precursor protein metabolic process 11/227 71/18866 8.15945E-10 9.27991E-09 3.50542E-09 CHRNA7/CASP3/RELA/ACHE/TNF/NTRK2/APP/IFNG/PSEN1/PSEN2/ABCG1 11

BP GO:0007254 JNK cascade 17/227 213/18866 8.85531E-10 1.00351E-08 3.79067E-09 EGFR/AKT1/TNF/IL1B/TLR4/APP/MMP8/SYK/MAPK8/IGF1R/EDN1/CDC42/GSTP1/CD40LG/TRPV4/MAPK10/MAPK9 17

BP GO:0051092 positive regulation of NF-kappaB transcription factor activity 15/227 159/18866 8.86563E-10 1.00351E-08 3.79067E-09 RELA/AR/TNF/IL1B/TLR4/NTRK1/APP/CAT/STAT3/ICAM1/PRKCB/CFLAR/NFKB1/CD40LG/EIF2AK2 15

BP GO:0002285 lymphocyte activation involved in immune response 16/227 187/18866 1.01903E-09 1.1507E-08 4.34669E-09 TGFB1/IL10/IL6/TP53/TLR4/STAT3/ICAM1/CD80/MTOR/CD28/IL2/IFNG/IL4/CD40LG/PSEN1/EIF2AK4 16

BP GO:0051966 regulation of synaptic transmission, glutamatergic 11/227 73/18866 1.1103E-09 1.2508E-08 4.72481E-09 PTGS2/HTR2A/EGFR/TNF/NTRK1/CDK5/CCL2/CCR2/GRM1/PSEN1/DRD2 11

BP GO:0034504 protein localization to nucleus 19/227 277/18866 1.14184E-09 1.28329E-08 4.84754E-09 PTGS2/TGFB1/AKT1/CDKN1A/MAPK1/TP53/MMP12/CDK1/CDK5/SYK/PARP1/F2/MAPK14/STAT3/MDM2/IFNG/PSEN1/SHH/EIF2AK3 19

BP GO:0010332 response to gamma radiation 10/227 56/18866 1.14499E-09 1.2838E-08 4.84945E-09 BCL2/BAX/BCL2L1/CDKN1A/TP53/MYC/PARP1/BAK1/MDM2/GPX1 10

BP GO:1902652 secondary alcohol metabolic process 15/227 162/18866 1.15164E-09 1.28821E-08 4.86611E-09 RXRA/PON1/SOD1/PPARD/APP/CAT/IL4/CYP3A4/HMGCR/NPC1L1/NR1H4/CETP/ABCG1/FDFT1/IDH1 15

BP GO:0010721 negative regulation of cell development 21/227 343/18866 1.16418E-09 1.29752E-08 4.90128E-09 SLC6A4/BCL2/MAP2/VEGFA/TNF/IL6/TP53/IL1B/BDNF/APP/CDK5/F2/STAT3/PPARA/MDM2/GNRH1/TRPV4/PSEN1/SHH/TRPV1/EIF2AK4 21

BP GO:0043200 response to amino acid 13/227 114/18866 1.16542E-09 1.29752E-08 4.90128E-09 CASP3/RELA/EGFR/BCL2L1/MMP2/TNF/NTRK2/GCLC/EDN1/ICAM1/MTOR/GSTP1/CREB1 13

BP GO:0036294 cellular response to decreased oxygen levels 17/227 218/18866 1.26528E-09 1.4054E-08 5.30881E-09 PTGS2/BCL2/AKT1/VEGFA/TP53/HIF1A/PPARD/MYC/HMOX1/NFE2L2/SIRT1/EDN1/ICAM1/CFLAR/MTOR/MDM2/EP300 17

BP GO:0051881 regulation of mitochondrial membrane potential 11/227 74/18866 1.29049E-09 1.43007E-08 5.402E-09 BCL2/BAX/AKT1/BCL2L1/SOD1/KDR/GCLC/PARP1/BAK1/VCP/TRPV1 11

BP GO:0070588 calcium ion transmembrane transport 20/227 312/18866 1.33493E-09 1.47588E-08 5.57502E-09 HTR2A/CHRNA7/PIK3CG/OPRM1/BAX/DDIT3/SNCA/HTR2C/CDK5/F2/FASLG/TRPV4/PSEN1/PSEN2/PTPN6/TRPV1/BDKRB1/CCR5/DRD2/HTR2B 20

BP GO:0022408 negative regulation of cell-cell adhesion 16/227 191/18866 1.38996E-09 1.53315E-08 5.79137E-09 CASP3/TGFB1/AKT1/VEGFA/IL10/PPARA/CD80/ERBB2/IL2/IL4/GNRH1/TRPV4/PTPN6/SHH/PRKG1/PDE5A 16

BP GO:0032602 chemokine production 11/227 75/18866 1.49645E-09 1.64678E-08 6.22061E-09 IL10/TNF/IL6/HIF1A/IL1B/TLR4/SYK/GSTP1/TRPV4/NR1H4/EIF2AK2 11

BP GO:0097553 calcium ion transmembrane import into cytosol 14/227 140/18866 1.52317E-09 1.67232E-08 6.31705E-09 HTR2A/BAX/DDIT3/SNCA/HTR2C/F2/FASLG/TRPV4/PTPN6/TRPV1/BDKRB1/CCR5/DRD2/HTR2B 14

BP GO:0050817 coagulation 21/227 349/18866 1.58994E-09 1.7416E-08 6.57879E-09 PLAU/PIK3CG/PRKCA/MAPK1/IL6/NFE2L2/TLR4/MAPK3/SYK/F2/EDN1/PRKCB/PLAT/CDC42/CD40LG/PRKCG/PSEN1/PTPN6/SHH/PLA2G4A/PRKG1 21

BP GO:0018107 peptidyl-threonine phosphorylation 13/227 117/18866 1.61273E-09 1.76251E-08 6.65774E-09 BCL2/PRKCA/TGFB1/AKT1/MAPK1/EGF/APP/CDK1/CDK5/MAPK8/PRKCB/MTOR/TGFBR1 13

BP GO:0002763 positive regulation of myeloid leukocyte differentiation 10/227 58/18866 1.64305E-09 1.79152E-08 6.76736E-09 JUN/CASP8/PRKCA/TGFB1/FOS/TNF/CA2/IFNG/CREB1/CCR1 10

BP GO:0019722 calcium-mediated signaling 17/227 222/18866 1.67163E-09 1.81852E-08 6.86934E-09 CHRM3/EGFR/TNF/KDR/SYK/EDN1/SELE/VCAM1/CXCL8/AGTR1/MTOR/CCR2/CCR1/CCR3/EIF2AK3/CCR5/HTR2B 17

BP GO:0043270 positive regulation of ion transport 19/227 284/18866 1.73079E-09 1.87858E-08 7.09619E-09 SLC6A4/BAX/AKT1/IL1B/GDNF/SNCA/CDK5/ABCB1/F2/BAK1/EDN1/CCL2/CCR2/IFNG/PSEN1/CETP/CCR1/BDKRB1/DRD2 19

BP GO:0035249 synaptic transmission, glutamatergic 12/227 96/18866 1.76642E-09 1.91288E-08 7.22578E-09 PTGS2/HTR2A/EGFR/TNF/NTRK1/CDK5/CCL2/CCR2/GRM1/PSEN1/GRIA2/DRD2 12

BP GO:0030217 T cell differentiation 18/227 253/18866 1.77832E-09 1.92139E-08 7.25791E-09 BCL2/IL6/TP53/SOD1/IL1B/SYK/CDK6/STAT3/IL1A/CD80/MTOR/CCR2/CD28/ERBB2/IL2/IFNG/IL4/SHH 18

BP GO:0150077 regulation of neuroinflammatory response 9/227 43/18866 1.78289E-09 1.92197E-08 7.26009E-09 PTGS2/MMP3/MMP9/TNF/IL6/IL1B/MMP8/IL4/TNFRSF1B 9

BP GO:0051209 release of sequestered calcium ion into cytosol 13/227 118/18866 1.79331E-09 1.92882E-08 7.286E-09 HTR2A/BAX/DDIT3/SNCA/HTR2C/F2/FASLG/PTPN6/TRPV1/BDKRB1/CCR5/DRD2/HTR2B 13

BP GO:0002819 regulation of adaptive immune response 15/227 168/18866 1.91108E-09 2.05085E-08 7.74694E-09 TGFB1/IL10/TNF/IL6/IL1B/SIRT1/CD80/CCR2/CD28/IL2/IL4/PTPN6/PLA2G4A/TNFRSF1B/EIF2AK4 15

BP GO:0031663 lipopolysaccharide-mediated signaling pathway 10/227 59/18866 1.95771E-09 2.09616E-08 7.91809E-09 PRKCA/TGFB1/AKT1/MAPK1/TNF/IL1B/TLR4/MAPK3/MAPK14/CCL2 10

BP GO:0051283 negative regulation of sequestering of calcium ion 13/227 119/18866 1.99205E-09 2.12813E-08 8.03887E-09 HTR2A/BAX/DDIT3/SNCA/HTR2C/F2/FASLG/PTPN6/TRPV1/BDKRB1/CCR5/DRD2/HTR2B 13

BP GO:0014706 striated muscle tissue development 22/227 389/18866 2.02156E-09 2.15482E-08 8.13968E-09 RXRA/BCL2/TGFB1/VEGFA/FOS/MAPK1/ATF3/CDK1/CDK5/MAPK14/EDN1/GJA1/PPARA/CFLAR/MTOR/CDC42/HMGCR/CREB1/SHH/GPX1/EP300/TGFBR1 22

BP GO:0016125 sterol metabolic process 15/227 169/18866 2.0751E-09 2.20694E-08 8.33655E-09 RXRA/PON1/SOD1/PPARD/APP/CAT/CYP19A1/IL4/CYP3A4/HMGCR/NPC1L1/NR1H4/CETP/ABCG1/FDFT1 15

BP GO:0030316 osteoclast differentiation 12/227 98/18866 2.24904E-09 2.37045E-08 8.9542E-09 FOS/TNF/TLR4/CA2/GLO1/MAPK14/MTOR/IFNG/IL4/CREB1/CCR1/CSF1R 12

BP GO:2001243 negative regulation of intrinsic apoptotic signaling pathway 12/227 98/18866 2.24904E-09 2.37045E-08 8.9542E-09 PTGS2/BCL2/AKT1/BCL2L1/MMP9/HIF1A/NFE2L2/MCL1/SIRT1/SOD2/MDM2/GPX1 12

BP GO:0051767 nitric-oxide synthase biosynthetic process 7/227 20/18866 2.25876E-09 2.37045E-08 8.9542E-09 STAT1/KDR/TLR4/EDN1/CCL2/IFNG/GSTP1 7

BP GO:0051769 regulation of nitric-oxide synthase biosynthetic process 7/227 20/18866 2.25876E-09 2.37045E-08 8.9542E-09 STAT1/KDR/TLR4/EDN1/CCL2/IFNG/GSTP1 7

BP GO:0060965 negative regulation of gene silencing by miRNA 7/227 20/18866 2.25876E-09 2.37045E-08 8.9542E-09 TGFB1/PPARG/TNF/IL6/TP53/ESR1/STAT3 7

BP GO:1902004 positive regulation of amyloid-beta formation 7/227 20/18866 2.25876E-09 2.37045E-08 8.9542E-09 CHRNA7/CASP3/RELA/TNF/APP/IFNG/ABCG1 7

BP GO:0030072 peptide hormone secretion 18/227 257/18866 2.28298E-09 2.39059E-08 9.0303E-09 PRKCA/DPP4/NOS2/EGFR/TNF/IL6/HIF1A/PPARD/IL1B/HTR2C/EDN1/GJA1/IFNG/TRPV4/HMGCR/NR1H4/ALOX5/DRD2 18

BP GO:0007612 learning 14/227 145/18866 2.41818E-09 2.5266E-08 9.54406E-09 PTGS2/JUN/FOS/HIF1A/NTRK2/APP/CDK5/MTOR/INSR/BCHE/HMGCR/CREB1/DRD2/EIF2AK4 14

BP GO:0051282 regulation of sequestering of calcium ion 13/227 121/18866 2.45056E-09 2.54922E-08 9.6295E-09 HTR2A/BAX/DDIT3/SNCA/HTR2C/F2/FASLG/PTPN6/TRPV1/BDKRB1/CCR5/DRD2/HTR2B 13

BP GO:1900542 regulation of purine nucleotide metabolic process 13/227 121/18866 2.45056E-09 2.54922E-08 9.6295E-09 HTR2A/NOS2/HIF1A/APP/PARP1/STAT3/PPARA/IFNG/IL4/INSR/VCP/PSEN1/EP300 13

BP GO:0042368 vitamin D biosynthetic process 6/227 12/18866 2.47097E-09 2.56485E-08 9.68852E-09 TNF/IL1B/NFKB1/IFNG/CYP3A4/VDR 6

BP GO:0043255 regulation of carbohydrate biosynthetic process 12/227 99/18866 2.53236E-09 2.62284E-08 9.90758E-09 TGFB1/AKT1/EGF/GPT/SNCA/SIRT1/FOXO1/PPARA/MTOR/NFKB1/INSR/EP300 12

BP GO:0050806 positive regulation of synaptic transmission 15/227 172/18866 2.64752E-09 2.73615E-08 1.03356E-08 PTGS2/CHRNA7/EGFR/MAPK1/TNF/NTRK1/NTRK2/CA2/APP/CDK5/CCL2/CCR2/CREB1/DRD2/EIF2AK4 15

BP GO:0006690 icosanoid metabolic process 13/227 122/18866 2.71394E-09 2.79212E-08 1.05471E-08 PTGS2/PON1/IL1B/MAPK3/SYK/SIRT1/EDN1/GSTP1/GPX1/GPX4/TNFRSF1A/ALOX5/PLA2G4A 13

BP GO:0042752 regulation of circadian rhythm 13/227 122/18866 2.71394E-09 2.79212E-08 1.05471E-08 PPARG/TP53/CDK1/MAPK8/PPARA/MTOR/NR1H3/PRKCG/CREB1/CSF2/MAPK10/MAPK9/DRD2 13

BP GO:0051054 positive regulation of DNA metabolic process 16/227 200/18866 2.7193E-09 2.79212E-08 1.05471E-08 BAX/TGFB1/EGFR/AKT1/MAPK1/IL6/MYC/MAPK3/HSP90AA1/PARP1/SIRT1/BRCA1/CD28/IL2/IL4/PRKCG 16

BP GO:1904646 cellular response to amyloid-beta 9/227 45/18866 2.74381E-09 2.81123E-08 1.06192E-08 TLR4/APP/CDK5/PARP1/IGF1R/GJA1/ICAM1/VCAM1/PSEN1 9

BP GO:0010574 regulation of vascular endothelial growth factor production 10/227 61/18866 2.75152E-09 2.81306E-08 1.06261E-08 PTGS2/TGFB1/IL6/HIF1A/IL1B/BRCA1/IL1A/C5AR1/CCR2/EIF2AK3 10

BP GO:0006631 fatty acid metabolic process 22/227 396/18866 2.80997E-09 2.86665E-08 1.08286E-08 PTGS2/PON1/PPARG/AKT1/PPARD/IL1B/MAPK3/MAPK14/SIRT1/EDN1/BRCA1/PPARA/MTOR/NR1H3/GSTP1/CYP3A4/PAM/GPX1/GPX4/TNFRSF1A/ALOX5/PLA2G4A 22

BP GO:0051604 protein maturation 19/227 293/18866 2.90173E-09 2.95393E-08 1.11582E-08 PLAU/CASP3/CASP8/NGF/PARP1/BAK1/PLAT/XIAP/BIRC3/MDM2/PRSS3/PSEN1/PSEN2/CASP1/SHH/C1R/BIRC2/EP300/REN 19

BP GO:0001763 morphogenesis of a branching structure 16/227 201/18866 2.9233E-09 2.9632E-08 1.11933E-08 PGR/RXRA/BCL2/AR/VEGFA/IL10/EGF/TNF/KDR/ESR1/MYC/GDNF/EDN1/SHH/VDR/DRD2 16

BP GO:0031099 regeneration 16/227 201/18866 2.9233E-09 2.9632E-08 1.11933E-08 BCL2/JUN/PPARG/EGFR/CDKN1A/IL10/IL6/PPARD/HMOX1/CDK1/BAK1/CFLAR/C5AR1/GSTP1/GPX1/DHFR 16

BP GO:0006140 regulation of nucleotide metabolic process 13/227 123/18866 3.00273E-09 3.03723E-08 1.14729E-08 HTR2A/NOS2/HIF1A/APP/PARP1/STAT3/PPARA/IFNG/IL4/INSR/VCP/PSEN1/EP300 13

BP GO:0051099 positive regulation of binding 15/227 174/18866 3.10576E-09 3.13478E-08 1.18414E-08 TGFB1/PON1/PPARG/MMP9/EGF/BDNF/NGF/APP/MMP8/CDK5/PARP1/EIF2S1/IFNG/PSEN1/EP300 15

BP GO:0034341 response to interferon-gamma 16/227 202/18866 3.14127E-09 3.1639E-08 1.19514E-08 PPARG/NOS2/TP53/STAT1/TLR4/SNCA/FASLG/EDN1/ICAM1/CCL2/VCAM1/JAK1/CDC42/NR1H3/IFNG/CASP1 16

BP GO:0042102 positive regulation of T cell proliferation 12/227 101/18866 3.1974E-09 3.21362E-08 1.21392E-08 IL6/IL1B/SYK/VCAM1/IL1A/CD80/CCR2/CD28/IL2/IL4/CD40LG/SHH 12

BP GO:0051208 sequestering of calcium ion 13/227 124/18866 3.31908E-09 3.32889E-08 1.25746E-08 HTR2A/BAX/DDIT3/SNCA/HTR2C/F2/FASLG/PTPN6/TRPV1/BDKRB1/CCR5/DRD2/HTR2B 13

BP GO:0036499 PERK-mediated unfolded protein response 7/227 21/18866 3.35361E-09 3.34938E-08 1.2652E-08 NFE2L2/ATF3/DDIT3/CCL2/CXCL8/EIF2S1/EIF2AK3 7

BP GO:0140467 integrated stress response signaling 7/227 21/18866 3.35361E-09 3.34938E-08 1.2652E-08 NFE2L2/ATF3/DDIT3/CCL2/CXCL8/EIF2S1/EIF2AK3 7

BP GO:0051052 regulation of DNA metabolic process 21/227 365/18866 3.5368E-09 3.52493E-08 1.33152E-08 BAX/TGFB1/PPARG/EGFR/AKT1/CDKN1A/MAPK1/IL10/IL6/TP53/MYC/MAPK3/HSP90AA1/PARP1/SIRT1/GJA1/BRCA1/CD28/IL2/IL4/PRKCG 21

BP GO:0006606 protein import into nucleus 14/227 150/18866 3.76949E-09 3.74899E-08 1.41615E-08 PTGS2/TGFB1/AKT1/CDKN1A/MAPK1/TP53/MMP12/CDK1/SYK/MAPK14/STAT3/IFNG/PSEN1/SHH 14

BP GO:0018210 peptidyl-threonine modification 13/227 126/18866 4.04398E-09 4.01358E-08 1.5161E-08 BCL2/PRKCA/TGFB1/AKT1/MAPK1/EGF/APP/CDK1/CDK5/MAPK8/PRKCB/MTOR/TGFBR1 13

BP GO:0006953 acute-phase response 9/227 47/18866 4.13201E-09 4.0839E-08 1.54266E-08 PTGS2/TNF/IL6/IL1B/F2/STAT3/IL1A/CRP/TRPV1 9

BP GO:0046677 response to antibiotic 9/227 47/18866 4.13201E-09 4.0839E-08 1.54266E-08 CASP9/CASP3/CASP8/TP53/SOD1/HSP90AA1/JAK1/MDM2/PLA2G4A 9

BP GO:1902003 regulation of amyloid-beta formation 8/227 33/18866 4.16119E-09 4.10421E-08 1.55034E-08 CHRNA7/CASP3/RELA/TNF/NTRK2/APP/IFNG/ABCG1 8

BP GO:0050866 negative regulation of cell activation 16/227 207/18866 4.47239E-09 4.40202E-08 1.66283E-08 CASP3/IL10/HMOX1/GCLC/F2/CD80/CCR2/NR1H3/ERBB2/IL2/IL4/GNRH1/PTPN6/SHH/PRKG1/PDE5A 16

BP GO:0007200 phospholipase C-activating G protein-coupled receptor signaling pathway 12/227 104/18866 4.49192E-09 4.4121E-08 1.66664E-08 HTR2A/OPRM1/ESR1/HTR2C/F2/EDN1/AGTR1/C5AR1/GRM1/TACR1/DRD2/HTR2B 12

BP GO:0010632 regulation of epithelial cell migration 19/227 301/18866 4.52009E-09 4.43062E-08 1.67363E-08 PTGS2/JUN/PRKCA/TGFB1/PPARG/AKT1/VEGFA/MMP9/TNF/HIF1A/KDR/HMOX1/NFE2L2/SIRT1/EDN1/MTOR/IFNG/IL4/ENPP2 19

BP GO:0048145 regulation of fibroblast proliferation 11/227 83/18866 4.53284E-09 4.43398E-08 1.6749E-08 BAX/JUN/PPARG/EGFR/CDKN1A/TP53/ESR1/MYC/CDK6/GSTP1/CREB1 11

BP GO:0010745 negative regulation of macrophage derived foam cell differentiation 6/227 13/18866 4.5429E-09 4.43469E-08 1.67517E-08 PPARG/PPARA/CRP/NR1H3/CETP/ABCG1 6

BP GO:0008203 cholesterol metabolic process 14/227 153/18866 4.87947E-09 4.75349E-08 1.7956E-08 RXRA/PON1/SOD1/PPARD/APP/CAT/IL4/CYP3A4/HMGCR/NPC1L1/NR1H4/CETP/ABCG1/FDFT1 14

BP GO:0014013 regulation of gliogenesis 13/227 128/18866 4.90937E-09 4.77284E-08 1.8029E-08 TGFB1/RELA/PPARG/TNF/IL6/IL1B/CDK1/F2/MTOR/CCR2/CREB1/SHH/TNFRSF1B 13

BP GO:0060537 muscle tissue development 22/227 409/18866 5.08166E-09 4.93024E-08 1.86236E-08 RXRA/BCL2/TGFB1/VEGFA/FOS/MAPK1/ATF3/CDK1/CDK5/MAPK14/EDN1/GJA1/PPARA/CFLAR/MTOR/CDC42/HMGCR/CREB1/SHH/GPX1/EP300/TGFBR1 22

BP GO:0048144 fibroblast proliferation 11/227 84/18866 5.16122E-09 4.99724E-08 1.88767E-08 BAX/JUN/PPARG/EGFR/CDKN1A/TP53/ESR1/MYC/CDK6/GSTP1/CREB1 11

BP GO:0071482 cellular response to light stimulus 13/227 129/18866 5.40208E-09 5.21982E-08 1.97175E-08 PTGS2/BAX/CASP9/CDKN1A/TP53/MYC/PARP1/SIRT1/BAK1/EIF2S1/MDM2/EP300/EIF2AK4 13

BP GO:0071868 cellular response to monoamine stimulus 12/227 106/18866 5.59939E-09 5.38856E-08 2.03549E-08 CHRM3/HTR2A/OPRM1/MAPK1/SNCA/MAPK3/APP/HTR2C/HRH3/HTR1A/DRD2/HTR2B 12

BP GO:0071870 cellular response to catecholamine stimulus 12/227 106/18866 5.59939E-09 5.38856E-08 2.03549E-08 CHRM3/HTR2A/OPRM1/MAPK1/SNCA/MAPK3/APP/HTR2C/HRH3/HTR1A/DRD2/HTR2B 12

BP GO:0071346 cellular response to interferon-gamma 15/227 182/18866 5.75892E-09 5.53089E-08 2.08926E-08 PPARG/NOS2/TP53/STAT1/TLR4/FASLG/EDN1/ICAM1/CCL2/VCAM1/JAK1/CDC42/NR1H3/IFNG/CASP1 15

BP GO:0002703 regulation of leukocyte mediated immunity 16/227 211/18866 5.89017E-09 5.63418E-08 2.12827E-08 TGFB1/NOS2/IL10/TNF/IL6/IL1B/HMOX1/TLR4/SYK/ICAM1/CCR2/CD28/IL2/IL4/PTPN6/TNFRSF1B 16

BP GO:0070555 response to interleukin-1 16/227 211/18866 5.89017E-09 5.63418E-08 2.12827E-08 PRKCA/RELA/IL6/HIF1A/IL1B/SNCA/MAPK3/APP/GCLC/EDN1/ICAM1/CCL2/SELE/CXCL8/IL1A/NFKB1 16

BP GO:0070664 negative regulation of leukocyte proliferation 11/227 86/18866 6.65621E-09 6.35414E-08 2.40023E-08 CASP3/IL10/CRP/CD80/ERBB2/IL2/GSTP1/GNRH1/PTPN6/SHH/PDE5A 11

BP GO:0090276 regulation of peptide hormone secretion 16/227 213/18866 6.74384E-09 6.42489E-08 2.42696E-08 PRKCA/DPP4/NOS2/EGFR/TNF/IL6/HIF1A/PPARD/IL1B/HTR2C/GJA1/IFNG/HMGCR/NR1H4/ALOX5/DRD2 16

BP GO:0007596 blood coagulation 20/227 343/18866 6.77007E-09 6.43698E-08 2.43152E-08 PLAU/PIK3CG/PRKCA/MAPK1/IL6/NFE2L2/TLR4/MAPK3/SYK/F2/EDN1/PRKCB/PLAT/CDC42/CD40LG/PRKCG/PTPN6/SHH/PLA2G4A/PRKG1 20

BP GO:0060149 negative regulation of posttranscriptional gene silencing 7/227 23/18866 6.92716E-09 6.53407E-08 2.4682E-08 TGFB1/PPARG/TNF/IL6/TP53/ESR1/STAT3 7

BP GO:0060967 negative regulation of gene silencing by RNA 7/227 23/18866 6.92716E-09 6.53407E-08 2.4682E-08 TGFB1/PPARG/TNF/IL6/TP53/ESR1/STAT3 7

BP GO:1903798 regulation of production of miRNAs involved in gene silencing by miRNA 7/227 23/18866 6.92716E-09 6.53407E-08 2.4682E-08 TGFB1/EGFR/TNF/IL6/TP53/ESR1/STAT3 7

BP GO:1904996 positive regulation of leukocyte adhesion to vascular endothelial cell 7/227 23/18866 6.92716E-09 6.53407E-08 2.4682E-08 RELA/TNF/IL6/ICAM1/SELE/CCR2/ALOX5 7

BP GO:0097300 programmed necrotic cell death 9/227 50/18866 7.36318E-09 6.9316E-08 2.61836E-08 BAX/CASP8/TNF/TP53/TLR4/FASLG/CFLAR/BIRC3/BIRC2 9

BP GO:0030168 platelet activation 14/227 158/18866 7.40562E-09 6.95777E-08 2.62825E-08 PIK3CG/PRKCA/MAPK1/IL6/TLR4/MAPK3/SYK/F2/PRKCB/CD40LG/PRKCG/PTPN6/PLA2G4A/PRKG1 14

BP GO:0006939 smooth muscle contraction 12/227 109/18866 7.72407E-09 7.24265E-08 2.73586E-08 PTGS2/CHRM3/HTR2A/SOD1/GDNF/EDN1/KCNMA1/TRPV1/PRKG1/TACR1/DRD2/HTR2B 12

BP GO:1903799 negative regulation of production of miRNAs involved in gene silencing by miRNA 6/227 14/18866 7.87032E-09 7.36525E-08 2.78217E-08 TGFB1/TNF/IL6/TP53/ESR1/STAT3 6

BP GO:0051651 maintenance of location in cell 16/227 216/18866 8.23841E-09 7.69458E-08 2.90657E-08 HTR2A/BAX/AKT1/DDIT3/SNCA/HTR2C/CDK5/F2/FASLG/GJA1/PTPN6/TRPV1/BDKRB1/CCR5/DRD2/HTR2B 16

BP GO:0008217 regulation of blood pressure 15/227 187/18866 8.33564E-09 7.73977E-08 2.92364E-08 PTGS2/AR/PPARG/NOS2/SOD1/HMOX1/EDN1/GJA1/SOD2/PPARA/AGTR1/TRPV1/REN/BDKRB1/DRD2 15

BP GO:0048639 positive regulation of developmental growth 15/227 187/18866 8.33564E-09 7.73977E-08 2.92364E-08 SLC6A3/BCL2/AKT1/VEGFA/MAPK1/PPARD/BDNF/NGF/CDK1/MAPK14/EDN1/MTOR/INSR/CREB1/DRD2 15

BP GO:0061138 morphogenesis of a branching epithelium 15/227 187/18866 8.33564E-09 7.73977E-08 2.92364E-08 PGR/RXRA/BCL2/AR/VEGFA/IL10/EGF/TNF/KDR/ESR1/MYC/GDNF/EDN1/SHH/VDR 15

BP GO:0050818 regulation of coagulation 11/227 88/18866 8.52661E-09 7.88628E-08 2.97899E-08 PLAU/PRKCA/NFE2L2/TLR4/SYK/F2/EDN1/PLAT/PSEN1/PLA2G4A/PRKG1 11

BP GO:0090559 regulation of membrane permeability 11/227 88/18866 8.52661E-09 7.88628E-08 2.97899E-08 BCL2/BAX/CASP8/BCL2L1/TP53/GCLC/STAT3/MAPK8/BAK1/MTOR/ATF2 11

BP GO:0002821 positive regulation of adaptive immune response 12/227 110/18866 8.57878E-09 7.88849E-08 2.97982E-08 TGFB1/TNF/IL6/IL1B/SIRT1/CD80/CCR2/CD28/IL2/IL4/PLA2G4A/EIF2AK4 12

BP GO:0071867 response to monoamine 12/227 110/18866 8.57878E-09 7.88849E-08 2.97982E-08 CHRM3/HTR2A/OPRM1/MAPK1/SNCA/MAPK3/APP/HTR2C/HRH3/HTR1A/DRD2/HTR2B 12

BP GO:0071869 response to catecholamine 12/227 110/18866 8.57878E-09 7.88849E-08 2.97982E-08 CHRM3/HTR2A/OPRM1/MAPK1/SNCA/MAPK3/APP/HTR2C/HRH3/HTR1A/DRD2/HTR2B 12

BP GO:0007599 hemostasis 20/227 348/18866 8.64744E-09 7.93628E-08 2.99787E-08 PLAU/PIK3CG/PRKCA/MAPK1/IL6/NFE2L2/TLR4/MAPK3/SYK/F2/EDN1/PRKCB/PLAT/CDC42/CD40LG/PRKCG/PTPN6/SHH/PLA2G4A/PRKG1 20

BP GO:0071478 cellular response to radiation 15/227 188/18866 8.96283E-09 8.20988E-08 3.10122E-08 PTGS2/BAX/CASP9/BCL2L1/CDKN1A/TP53/MYC/PARP1/MAPK14/SIRT1/BAK1/EIF2S1/MDM2/EP300/EIF2AK4 15

BP GO:1902993 positive regulation of amyloid precursor protein catabolic process 7/227 24/18866 9.67989E-09 8.84965E-08 3.34289E-08 CHRNA7/CASP3/RELA/TNF/APP/IFNG/ABCG1 7

BP GO:0046822 regulation of nucleocytoplasmic transport 12/227 112/18866 1.05477E-08 9.62451E-08 3.63559E-08 PTGS2/TGFB1/MAPK1/TP53/IL1B/CDK1/CDK5/MAPK14/MDM2/IFNG/PSEN1/SHH 12

BP GO:0050999 regulation of nitric-oxide synthase activity 9/227 52/18866 1.05891E-08 9.64382E-08 3.64288E-08 EGFR/AKT1/TNF/HIF1A/IL1B/HSP90AA1/IL1A/DHFR/HTR2B 9

BP GO:0010821 regulation of mitochondrion organization 15/227 191/18866 1.11116E-08 1.01003E-07 3.81532E-08 BCL2/BAX/CASP8/AKT1/BCL2L1/MMP9/TP53/HIF1A/KDR/GCLC/MAPK8/BAK1/TNFSF10/GPX1/EP300 15

BP GO:0030183 B cell differentiation 13/227 137/18866 1.12684E-08 1.02233E-07 3.86176E-08 BCL2/BAX/IL10/IL6/TP53/NTRK1/SYK/BAK1/VCAM1/IL4/CD40LG/PTPN6/EP300 13

BP GO:0002064 epithelial cell development 16/227 221/18866 1.14171E-08 1.03384E-07 3.90527E-08 PGR/AR/VEGFA/CDKN1A/TNF/SOD1/HIF1A/ESR1/IL1B/NTRK1/AKR1B1/CDK6/ICAM1/PECAM1/GPX1/TNFRSF1A 16

BP GO:0051961 negative regulation of nervous system development 19/227 319/18866 1.16426E-08 1.05226E-07 3.97484E-08 SLC6A4/MAP2/TNF/IL6/TP53/IL1B/BDNF/APP/CDK5/F2/STAT3/MDM2/GNRH1/TRPV4/PSEN1/SHH/TRPV1/EIF2AK3/EIF2AK4 19

BP GO:0046165 alcohol biosynthetic process 14/227 164/18866 1.19746E-08 1.08021E-07 4.08043E-08 TNF/SOD1/IL1B/SNCA/AKR1B1/NFKB1/IFNG/CYP3A4/HMGCR/NPC1L1/ABCG1/FDFT1/VDR/DHFR 14

BP GO:0051924 regulation of calcium ion transport 17/227 253/18866 1.2011E-08 1.08144E-07 4.08507E-08 PTGS2/PIK3CG/BCL2/BAX/EGF/SNCA/CDK5/F2/BAK1/GJA1/ICAM1/CCL2/PSEN2/PTPN6/CCR1/BDKRB1/DRD2 17

BP GO:0033273 response to vitamin 11/227 91/18866 1.22157E-08 1.09572E-07 4.13901E-08 PTGS2/RELA/PPARG/EGFR/PPARD/CAT/TYR/MDM2/GSTP1/BCHE/VDR 11

BP GO:0034103 regulation of tissue remodeling 11/227 91/18866 1.22157E-08 1.09572E-07 4.13901E-08 BAX/PRKCA/TGFB1/EGFR/IL6/TP53/CA2/SYK/IL2/CSF1R/VDR 11

BP GO:0043542 endothelial cell migration 18/227 286/18866 1.22502E-08 1.09675E-07 4.14289E-08 PTGS2/PRKCA/TGFB1/PPARG/DPP4/AKT1/VEGFA/TNF/HIF1A/KDR/HMOX1/NFE2L2/SIRT1/EDN1/PECAM1/PRSS3/GPX1/TGFBR1 18

BP GO:0014823 response to activity 10/227 71/18866 1.26855E-08 1.1288E-07 4.26397E-08 IL10/IL6/HIF1A/PPARD/CAT/GCLC/CDK1/EDN1/MTOR/CREB1 10

BP GO:0042531 positive regulation of tyrosine phosphorylation of STAT protein 10/227 71/18866 1.26855E-08 1.1288E-07 4.26397E-08 TNF/IL6/STAT3/PECAM1/IL2/IFNG/IL4/CSF2/TNFRSF1A/CSF1R 10

BP GO:0061180 mammary gland epithelium development 10/227 71/18866 1.26855E-08 1.1288E-07 4.26397E-08 PGR/BAX/AR/AKT1/MAPK1/HIF1A/ESR1/GPX1/CSF1R/VDR 10

BP GO:0019318 hexose metabolic process 17/227 254/18866 1.27367E-08 1.1288E-07 4.26397E-08 AKT1/TNF/TP53/PPARD/GPT/ATF3/AKR1B1/FUCA1/MAPK14/SIRT1/FOXO1/PPARA/MTOR/INSR/TPI1/GLB1/EP300 17

BP GO:2000027 regulation of animal organ morphogenesis 17/227 254/18866 1.27367E-08 1.1288E-07 4.26397E-08 RXRA/BCL2/BAX/AR/VEGFA/TNF/STAT1/ESR1/MYC/GDNF/BDNF/EDN1/CFLAR/CDC42/SHH/VDR/TGFBR1 17

BP GO:0060249 anatomical structure homeostasis 23/227 469/18866 1.27507E-08 1.1288E-07 4.26397E-08 PTGS2/BCL2/BAX/PRKCA/EGFR/VEGFA/MAPK1/IL6/SOD1/HIF1A/MYC/TLR4/MAPK3/HSP90AA1/CA2/AKR1B1/CDK5/SYK/PARP1/GJA1/PECAM1/CCR2/CSF1R 23

BP GO:1990748 cellular detoxification 12/227 114/18866 1.29135E-08 1.1411E-07 4.31041E-08 PTGS2/TNF/SOD1/NFE2L2/CAT/ABCG2/MPO/SOD2/GSTP1/GPX1/GPX4/DHFR 12

BP GO:0034349 glial cell apoptotic process 6/227 15/18866 1.29856E-08 1.1411E-07 4.31043E-08 CASP9/CASP3/PRKCA/TP53/CDK5/CCL2 6

BP GO:0042362 fat-soluble vitamin biosynthetic process 6/227 15/18866 1.29856E-08 1.1411E-07 4.31043E-08 TNF/IL1B/NFKB1/IFNG/CYP3A4/VDR 6

BP GO:0090594 inflammatory response to wounding 6/227 15/18866 1.29856E-08 1.1411E-07 4.31043E-08 TGFB1/HIF1A/HMOX1/IL1A/CCR2/ALOX5 6

BP GO:0046697 decidualization 7/227 25/18866 1.33073E-08 1.16507E-07 4.40095E-08 PTGS2/MAPK1/PPARD/MAPK3/GJA1/CTSB/VDR 7

BP GO:0070920 regulation of production of small RNA involved in gene silencing by RNA 7/227 25/18866 1.33073E-08 1.16507E-07 4.40095E-08 TGFB1/EGFR/TNF/IL6/TP53/ESR1/STAT3 7

BP GO:0043401 steroid hormone mediated signaling pathway 13/227 139/18866 1.34364E-08 1.1742E-07 4.43547E-08 PGR/RXRA/AR/ESR2/PPARD/ESR1/PARP1/SIRT1/BRCA1/PPARA/RXRB/RXRG/EP300 13

BP GO:0045787 positive regulation of cell cycle 21/227 396/18866 1.4787E-08 1.28986E-07 4.87236E-08 SLC6A4/BAX/PRKCA/EGFR/AKT1/CDKN1A/IL10/EGF/TP53/IL1B/APP/CDK1/EDN1/BRCA1/IL1A/CDC42/CD28/MDM2/INSR/EP300/DRD2 21

BP GO:0032869 cellular response to insulin stimulus 16/227 226/18866 1.56837E-08 1.36558E-07 5.15837E-08 RELA/PPARG/AKT1/STAT1/IL1B/GPT/GCLC/PARP1/SIRT1/IGF1R/FOXO1/PRKCB/CFLAR/GSTP1/INSR/NR1H4 16

BP GO:0010517 regulation of phospholipase activity 10/227 73/18866 1.67156E-08 1.45276E-07 5.48769E-08 HTR2A/EGFR/ESR1/SNCA/BDNF/NTRK2/SELE/AGTR1/C5AR1/HTR2B 10

BP GO:0006694 steroid biosynthetic process 15/227 197/18866 1.68769E-08 1.4641E-07 5.53053E-08 TNF/SOD1/IL1B/AKR1B1/CYP19A1/SIRT1/NFKB1/IFNG/CYP3A4/HMGCR/NPC1L1/NR1H4/ABCG1/FDFT1/VDR 15

BP GO:0042180 cellular ketone metabolic process 17/227 260/18866 1.8005E-08 1.55912E-07 5.88947E-08 PTGS2/PPARG/AKT1/ODC1/PPARD/IL1B/AKR1B1/CYP19A1/GLO1/SIRT1/BRCA1/PPARA/MTOR/NR1H3/TPI1/HMGCR/NR1H4 17

BP GO:0032635 interleukin-6 production 14/227 170/18866 1.8972E-08 1.6369E-07 6.18327E-08 NOS2/IL10/TNF/IL6/IL1B/TLR4/APP/MMP8/SYK/STAT3/IL1A/IFNG/PTPN6/NR1H4 14

BP GO:0032874 positive regulation of stress-activated MAPK cascade 14/227 170/18866 1.8972E-08 1.6369E-07 6.18327E-08 VEGFA/TNF/XDH/IL1B/TLR4/APP/MMP8/SYK/EDN1/CDC42/CD40LG/TRPV4/HMGCR/EIF2AK2 14

BP GO:0051170 import into nucleus 14/227 171/18866 2.0446E-08 1.76088E-07 6.65159E-08 PTGS2/TGFB1/AKT1/CDKN1A/MAPK1/TP53/MMP12/CDK1/SYK/MAPK14/STAT3/IFNG/PSEN1/SHH 14

BP GO:0002700 regulation of production of molecular mediator of immune response 13/227 144/18866 2.05978E-08 1.77074E-07 6.68884E-08 TGFB1/IL10/TNF/IL6/IL1B/HMOX1/TLR4/CCR2/CD28/IL2/IL4/CD40LG/TNFRSF1B 13

BP GO:0009755 hormone-mediated signaling pathway 15/227 200/18866 2.06816E-08 1.77153E-07 6.69185E-08 PGR/RXRA/AR/PPARG/ESR2/PPARD/ESR1/PARP1/SIRT1/BRCA1/PPARA/RXRB/RXRG/EP300/REN 15

BP GO:0017038 protein import 15/227 200/18866 2.06816E-08 1.77153E-07 6.69185E-08 PTGS2/TGFB1/AKT1/CDKN1A/MAPK1/TP53/MMP12/HSP90AA1/CDK1/SYK/MAPK14/STAT3/IFNG/PSEN1/SHH 15

BP GO:0005996 monosaccharide metabolic process 18/227 296/18866 2.0841E-08 1.78198E-07 6.73132E-08 AKT1/TNF/TP53/PPARD/GPT/ATF3/AKR1B1/GCLC/FUCA1/MAPK14/SIRT1/FOXO1/PPARA/MTOR/INSR/TPI1/GLB1/EP300 18

BP GO:0046394 carboxylic acid biosynthetic process 20/227 367/18866 2.10838E-08 1.79766E-07 6.79055E-08 PTGS2/TGFB1/EGF/IL1B/GPT/FOLH1/SYK/SIRT1/EDN1/BRCA1/NR1H3/NFKB1/GSTP1/CYP3A4/GAMT/NR1H4/GPX4/ALOX5/PLA2G4A/DHFR 20

BP GO:0060562 epithelial tube morphogenesis 19/227 331/18866 2.11001E-08 1.79766E-07 6.79055E-08 PGR/BCL2/CASP3/TGFB1/AR/VEGFA/EGF/TNF/HIF1A/KDR/ESR1/MYC/GDNF/EDN1/PSEN1/SHH/CSF1R/VDR/RET 19

BP GO:0042307 positive regulation of protein import into nucleus 8/227 40/18866 2.14379E-08 1.81993E-07 6.87464E-08 PTGS2/TGFB1/MAPK1/CDK1/MAPK14/IFNG/PSEN1/SHH 8

BP GO:1902991 regulation of amyloid precursor protein catabolic process 8/227 40/18866 2.14379E-08 1.81993E-07 6.87464E-08 CHRNA7/CASP3/RELA/TNF/NTRK2/APP/IFNG/ABCG1 8

BP GO:0031100 animal organ regeneration 10/227 75/18866 2.18379E-08 1.85058E-07 6.99044E-08 PPARG/EGFR/CDKN1A/IL10/IL6/HMOX1/CDK1/BAK1/C5AR1/GSTP1 10

BP GO:0070304 positive regulation of stress-activated protein kinase signaling cascade 14/227 172/18866 2.20231E-08 1.8628E-07 7.03659E-08 VEGFA/TNF/XDH/IL1B/TLR4/APP/MMP8/SYK/EDN1/CDC42/CD40LG/TRPV4/HMGCR/EIF2AK2 14

BP GO:0016053 organic acid biosynthetic process 20/227 368/18866 2.20605E-08 1.8628E-07 7.03659E-08 PTGS2/TGFB1/EGF/IL1B/GPT/FOLH1/SYK/SIRT1/EDN1/BRCA1/NR1H3/NFKB1/GSTP1/CYP3A4/GAMT/NR1H4/GPX4/ALOX5/PLA2G4A/DHFR 20

BP GO:0038127 ERBB signaling pathway 13/227 145/18866 2.23882E-08 1.88712E-07 7.12846E-08 PRKCA/TGFB1/EGFR/AKT1/MMP9/MAPK1/EGF/HSP90AA1/APP/FASLG/CDC42/ERBB2/PSEN1 13

BP GO:0033157 regulation of intracellular protein transport 17/227 264/18866 2.25567E-08 1.89796E-07 7.16941E-08 PTGS2/BCL2/CASP8/TGFB1/MAPK1/TP53/IL1B/CDK1/CDK5/MAPK14/MAPK8/CDC42/MDM2/ERBB2/IFNG/PSEN1/SHH 17

BP GO:0033209 tumor necrosis factor-mediated signaling pathway 14/227 173/18866 2.37097E-08 1.99118E-07 7.52154E-08 CASP8/RELA/TNF/TP53/STAT1/SYK/BIRC3/GSTP1/CD40LG/CASP1/NR1H4/TNFRSF1A/TNFRSF1B/BIRC2 14

BP GO:0007517 muscle organ development 21/227 407/18866 2.37484E-08 1.99118E-07 7.52154E-08 RXRA/BCL2/TGFB1/FOS/MAPK1/ATF3/CDK1/CDK5/MAPK14/SIRT1/EDN1/GJA1/PPARA/CFLAR/MTOR/HMGCR/CREB1/SHH/GPX1/EP300/TGFBR1 21

BP GO:0001963 synaptic transmission, dopaminergic 7/227 27/18866 2.40853E-08 2.01588E-07 7.61483E-08 PTGS2/SLC6A3/SLC6A4/GDNF/SNCA/CDK5/DRD2 7

BP GO:0002460 adaptive immune response based on somatic recombination of immune receptors built from immunoglobulin superfamily domains 20/227 370/18866 2.41405E-08 2.01694E-07 7.61885E-08 TGFB1/IL10/TNF/IL6/IL1B/TLR4/STAT3/ICAM1/CRP/CD80/MTOR/CCR2/CD28/IL2/IL4/CD40LG/PTPN6/PLA2G4A/C1R/TNFRSF1B 20

BP GO:1905517 macrophage migration 9/227 57/18866 2.4578E-08 2.04989E-07 7.7433E-08 MAPK1/MAPK3/CYP19A1/MAPK14/CCL2/C5AR1/CCR2/TRPV4/CSF1R 9

BP GO:0060193 positive regulation of lipase activity 10/227 76/18866 2.48844E-08 2.07181E-07 7.82612E-08 HTR2A/EGFR/ESR1/BDNF/NTRK2/SELE/AGTR1/C5AR1/NR1H3/HTR2B 10

BP GO:0007613 memory 12/227 121/18866 2.54143E-08 2.10855E-07 7.96488E-08 PTGS2/HTR2A/CHRNA7/SLC6A4/BDNF/NGF/MTOR/INSR/PSEN1/CREB1/DRD2/EIF2AK4 12

BP GO:0032612 interleukin-1 production 12/227 121/18866 2.54143E-08 2.10855E-07 7.96488E-08 CASP8/IL10/TNF/IL6/IL1B/TLR4/APP/STAT3/IFNG/GSTP1/CASP1/NR1H4 12

BP GO:0002831 regulation of response to biotic stimulus 21/227 409/18866 2.58392E-08 2.14006E-07 8.08393E-08 PRKCA/RELA/PPARG/STAT1/IL1B/TLR4/MAPK3/MMP12/SYK/XIAP/JAK1/BIRC3/NR1H3/NFKB1/IFNG/IL4/PTPN6/BIRC2/EP300/DRD2/EIF2AK4 21

BP GO:0038083 peptidyl-tyrosine autophosphorylation 8/227 41/18866 2.63608E-08 2.17946E-07 8.23277E-08 EGFR/VEGFA/KDR/MAPK3/NTRK1/SYK/IGF1R/INSR 8

BP GO:0050810 regulation of steroid biosynthetic process 11/227 98/18866 2.6866E-08 2.21738E-07 8.37599E-08 TNF/SOD1/IL1B/SIRT1/NFKB1/IFNG/HMGCR/NR1H4/ABCG1/FDFT1/VDR 11

BP GO:0050680 negative regulation of epithelial cell proliferation 14/227 175/18866 2.74388E-08 2.26073E-07 8.53973E-08 TGFB1/AR/PPARG/TNF/XDH/STAT1/PPARD/CDK6/GJA1/CCL2/ATF2/ALOX5/VDR/TGFBR1 14

BP GO:0034765 regulation of ion transmembrane transport 23/227 489/18866 2.75921E-08 2.26943E-07 8.5726E-08 CHRM3/PIK3CG/OPRM1/BAX/AKT1/MMP9/SNCA/APP/CDK5/ABCB1/F2/CCL2/MTOR/CCR2/IFNG/KCNMA1/PSEN1/PSEN2/PTPN6/GRIA2/SCN9A/BDKRB1/DRD2 23

BP GO:0097237 cellular response to toxic substance 12/227 122/18866 2.78907E-08 2.29003E-07 8.65041E-08 PTGS2/TNF/SOD1/NFE2L2/CAT/ABCG2/MPO/SOD2/GSTP1/GPX1/GPX4/DHFR 12

BP GO:0007422 peripheral nervous system development 10/227 77/18866 2.83002E-08 2.31167E-07 8.73216E-08 RELA/AKT1/SOD1/GDNF/BDNF/NGF/NTRK2/CDK1/CDK5/ERBB2 10

BP GO:0014015 positive regulation of gliogenesis 10/227 77/18866 2.83002E-08 2.31167E-07 8.73216E-08 TGFB1/RELA/PPARG/TNF/IL6/IL1B/MTOR/CCR2/SHH/TNFRSF1B 10

BP GO:0046902 regulation of mitochondrial membrane permeability 10/227 77/18866 2.83002E-08 2.31167E-07 8.73216E-08 BCL2/BAX/CASP8/BCL2L1/TP53/GCLC/STAT3/MAPK8/BAK1/ATF2 10

BP GO:0090068 positive regulation of cell cycle process 18/227 302/18866 2.83658E-08 2.31305E-07 8.7374E-08 BAX/EGFR/AKT1/CDKN1A/EGF/TP53/IL1B/APP/CDK1/EDN1/BRCA1/IL1A/CDC42/CD28/MDM2/INSR/EP300/DRD2 18

BP GO:0007566 embryo implantation 9/227 58/18866 2.87905E-08 2.34366E-07 8.85302E-08 RXRA/PTGS2/VEGFA/MMP2/MMP9/SOD1/PPARD/IL1B/GJA1 9

BP GO:0048246 macrophage chemotaxis 8/227 42/18866 3.22282E-08 2.61455E-07 9.87628E-08 MAPK1/MAPK3/CYP19A1/MAPK14/CCL2/C5AR1/TRPV4/CSF1R 8

BP GO:1904591 positive regulation of protein import 8/227 42/18866 3.22282E-08 2.61455E-07 9.87628E-08 PTGS2/TGFB1/MAPK1/CDK1/MAPK14/IFNG/PSEN1/SHH 8

BP GO:0043536 positive regulation of blood vessel endothelial cell migration 10/227 79/18866 3.63953E-08 2.94759E-07 1.11343E-07 PTGS2/PRKCA/TGFB1/AKT1/VEGFA/HIF1A/KDR/HMOX1/NFE2L2/SIRT1 10

BP GO:0030968 endoplasmic reticulum unfolded protein response 12/227 125/18866 3.66708E-08 2.95981E-07 1.11805E-07 BAX/NFE2L2/ATF3/DDIT3/BAK1/CCL2/CXCL8/EIF2S1/VCP/EIF2AK3/EP300/EIF2AK2 12

BP GO:0043500 muscle adaptation 12/227 125/18866 3.66708E-08 2.95981E-07 1.11805E-07 PRKCA/IL1B/HMOX1/PARP1/EDN1/FOXO1/PPARA/CFLAR/MTOR/TNFRSF1A/TNFRSF1B/PDE5A 12

BP GO:0032637 interleukin-8 production 11/227 101/18866 3.69256E-08 2.9703E-07 1.12201E-07 RELA/NOS2/IL10/TNF/IL6/IL1B/TLR4/DDIT3/SYK/STAT3/CRP 11

BP GO:0060191 regulation of lipase activity 11/227 101/18866 3.69256E-08 2.9703E-07 1.12201E-07 HTR2A/EGFR/ESR1/SNCA/BDNF/NTRK2/SELE/AGTR1/C5AR1/NR1H3/HTR2B 11

BP GO:0019229 regulation of vasoconstriction 9/227 60/18866 3.91431E-08 3.13101E-07 1.18272E-07 PTGS2/CHRM3/HTR2A/EGFR/AKT1/EDN1/GJA1/ICAM1/AGTR1 9

BP GO:0043388 positive regulation of DNA binding 9/227 60/18866 3.91431E-08 3.13101E-07 1.18272E-07 TGFB1/PPARG/MMP9/EGF/NGF/MMP8/PARP1/IFNG/EP300 9

BP GO:0097755 positive regulation of blood vessel diameter 9/227 60/18866 3.91431E-08 3.13101E-07 1.18272E-07 EGFR/SOD1/PPARD/HMOX1/GJA1/SOD2/KCNMA1/GPX1/PRKG1 9

BP GO:0046688 response to copper ion 8/227 43/18866 3.91871E-08 3.13101E-07 1.18272E-07 SOD1/NFE2L2/SNCA/APP/CDK1/ICAM1/IL1A/PAM 8

BP GO:0034764 positive regulation of transmembrane transport 15/227 210/18866 3.96979E-08 3.16651E-07 1.19613E-07 BAX/AKT1/NFE2L2/SNCA/CA2/CDK5/ABCB1/F2/MAPK14/CCL2/CCR2/IFNG/INSR/PSEN1/BDKRB1 15

BP GO:0032945 negative regulation of mononuclear cell proliferation 10/227 80/18866 4.11608E-08 3.2777E-07 1.23813E-07 CASP3/IL10/CRP/CD80/ERBB2/IL2/GNRH1/PTPN6/SHH/PDE5A 10

BP GO:0050796 regulation of insulin secretion 14/227 181/18866 4.2035E-08 3.34172E-07 1.26231E-07 PRKCA/DPP4/NOS2/TNF/IL6/HIF1A/PPARD/IL1B/GJA1/IFNG/HMGCR/NR1H4/ALOX5/DRD2 14

BP GO:0002822 regulation of adaptive immune response based on somatic recombination of immune receptors built from immunoglobulin superfamily domains 13/227 153/18866 4.25999E-08 3.37533E-07 1.27501E-07 TGFB1/IL10/TNF/IL6/IL1B/CD80/CCR2/CD28/IL2/IL4/PTPN6/PLA2G4A/TNFRSF1B 13

BP GO:0016202 regulation of striated muscle tissue development 13/227 153/18866 4.25999E-08 3.37533E-07 1.27501E-07 BCL2/TGFB1/MAPK1/CDK1/MAPK14/EDN1/GJA1/PPARA/MTOR/HMGCR/CREB1/SHH/TGFBR1 13

BP GO:1900180 regulation of protein localization to nucleus 12/227 127/18866 4.38249E-08 3.46662E-07 1.30949E-07 PTGS2/TGFB1/AKT1/MAPK1/CDK1/PARP1/F2/MAPK14/IFNG/PSEN1/SHH/EIF2AK3 12

BP GO:0051701 interaction with host 15/227 212/18866 4.5025E-08 3.55563E-07 1.34311E-07 RXRA/HTR2A/CASP8/DPP4/EGFR/BCL2L1/CDK1/ICAM1/CXCL8/CD80/INSR/CTSB/CCR5/EIF2AK4/EIF2AK2 15

BP GO:0098869 cellular oxidant detoxification 11/227 103/18866 4.53725E-08 3.57713E-07 1.35123E-07 PTGS2/TNF/SOD1/NFE2L2/CAT/MPO/SOD2/GSTP1/GPX1/GPX4/DHFR 11

BP GO:0002262 myeloid cell homeostasis 13/227 154/18866 4.60379E-08 3.62358E-07 1.36878E-07 BAX/CASP3/VEGFA/IL6/SOD1/HIF1A/STAT1/HMOX1/CDK6/MAPK14/STAT3/BAK1/CCR2 13

BP GO:0035994 response to muscle stretch 6/227 18/18866 4.67305E-08 3.67201E-07 1.38708E-07 JUN/RELA/FOS/MAPK14/EDN1/NFKB1 6

BP GO:0016051 carbohydrate biosynthetic process 15/227 213/18866 4.7925E-08 3.75966E-07 1.42019E-07 TGFB1/AKT1/EGF/GPT/ATF3/SNCA/AKR1B1/SIRT1/FOXO1/PPARA/MTOR/NFKB1/INSR/TPI1/EP300 15

BP GO:0048754 branching morphogenesis of an epithelial tube 13/227 155/18866 4.97238E-08 3.88794E-07 1.46864E-07 PGR/BCL2/AR/VEGFA/EGF/TNF/KDR/ESR1/MYC/GDNF/EDN1/SHH/VDR 13

BP GO:1990845 adaptive thermogenesis 13/227 155/18866 4.97238E-08 3.88794E-07 1.46864E-07 ACHE/VEGFA/TLR4/DDIT3/SYK/IGF1R/GJA1/CCR2/NR1H3/IL4/TRPV4/TRPV1/UCP2 13

BP GO:0002367 cytokine production involved in immune response 11/227 104/18866 5.02078E-08 3.91934E-07 1.4805E-07 TGFB1/IL10/TNF/IL6/IL1B/HMOX1/TLR4/SIRT1/CCR2/IL4/TNFRSF1B 11

BP GO:0030336 negative regulation of cell migration 19/227 350/18866 5.12847E-08 3.9903E-07 1.50731E-07 BCL2/TGFB1/PPARG/DPP4/AKT1/TNF/PPARD/HMOX1/NFE2L2/CYP19A1/STAT3/GJA1/CCL2/IL4/GSTP1/GNRH1/SHH/PRKG1/DRD2 19

BP GO:0046942 carboxylic acid transport 19/227 350/18866 5.12847E-08 3.9903E-07 1.50731E-07 RXRA/PPARG/NOS2/AKT1/PPARD/IL1B/BDNF/NTRK2/SYK/ABCG2/EDN1/GJA1/PPARA/PSEN1/NR1H4/TRPV1/PLA2G4A/HRH3/DRD2 19

BP GO:0046328 regulation of JNK cascade 14/227 184/18866 5.17015E-08 4.01616E-07 1.51708E-07 EGFR/AKT1/TNF/IL1B/TLR4/APP/MMP8/SYK/IGF1R/EDN1/CDC42/GSTP1/CD40LG/TRPV4 14

BP GO:0030193 regulation of blood coagulation 10/227 82/18866 5.23704E-08 4.05487E-07 1.5317E-07 PLAU/PRKCA/NFE2L2/TLR4/SYK/F2/EDN1/PLAT/PLA2G4A/PRKG1 10

BP GO:1902930 regulation of alcohol biosynthetic process 10/227 82/18866 5.23704E-08 4.05487E-07 1.5317E-07 TNF/SOD1/IL1B/SNCA/NFKB1/IFNG/HMGCR/ABCG1/FDFT1/VDR 10

BP GO:0010518 positive regulation of phospholipase activity 9/227 62/18866 5.26069E-08 4.05996E-07 1.53362E-07 HTR2A/EGFR/ESR1/BDNF/NTRK2/SELE/AGTR1/C5AR1/HTR2B 9

BP GO:0010803 regulation of tumor necrosis factor-mediated signaling pathway 9/227 62/18866 5.26069E-08 4.05996E-07 1.53362E-07 CASP8/TNF/SYK/BIRC3/GSTP1/CASP1/NR1H4/TNFRSF1A/BIRC2 9

BP GO:0010758 regulation of macrophage chemotaxis 7/227 30/18866 5.3546E-08 4.11905E-07 1.55594E-07 MAPK1/MAPK3/CYP19A1/MAPK14/C5AR1/TRPV4/CSF1R 7

BP GO:0043457 regulation of cellular respiration 7/227 30/18866 5.3546E-08 4.11905E-07 1.55594E-07 NOS2/HIF1A/SNCA/CDK1/IFNG/IL4/VCP 7

BP GO:1901861 regulation of muscle tissue development 13/227 156/18866 5.36733E-08 4.12218E-07 1.55712E-07 BCL2/TGFB1/MAPK1/CDK1/MAPK14/EDN1/GJA1/PPARA/MTOR/HMGCR/CREB1/SHH/TGFBR1 13

BP GO:0007631 feeding behavior 11/227 105/18866 5.5496E-08 4.24843E-07 1.60482E-07 OPRM1/FOS/BDNF/NTRK2/APP/HTR2C/STAT3/MTOR/REN/DRD2/EIF2AK4 11

BP GO:0062014 negative regulation of small molecule metabolic process 11/227 105/18866 5.5496E-08 4.24843E-07 1.60482E-07 TGFB1/AKT1/TP53/SOD1/PARP1/SIRT1/STAT3/BRCA1/PPARA/NFKB1/EP300 11

BP GO:0032722 positive regulation of chemokine production 8/227 45/18866 5.70583E-08 4.35402E-07 1.6447E-07 TNF/IL6/HIF1A/IL1B/TLR4/SYK/TRPV4/EIF2AK2 8

BP GO:0070266 necroptotic process 8/227 45/18866 5.70583E-08 4.35402E-07 1.6447E-07 CASP8/TNF/TP53/TLR4/FASLG/CFLAR/BIRC3/BIRC2 8

BP GO:0042692 muscle cell differentiation 20/227 390/18866 5.75171E-08 4.37639E-07 1.65315E-07 RXRA/BCL2/CASP3/TGFB1/AKT1/VEGFA/BDNF/CDK1/MAPK14/SIRT1/EDN1/SOD2/PPARA/CFLAR/MTOR/CDC42/MDM2/IL4/SHH/GPX1 20

BP GO:0048634 regulation of muscle organ development 13/227 157/18866 5.7903E-08 4.37639E-07 1.65315E-07 BCL2/TGFB1/MAPK1/CDK1/MAPK14/EDN1/GJA1/PPARA/MTOR/HMGCR/CREB1/SHH/TGFBR1 13

BP GO:0001660 fever generation 5/227 10/18866 5.79038E-08 4.37639E-07 1.65315E-07 PTGS2/TNF/IL1B/IL1A/TRPV1 5

BP GO:0010887 negative regulation of cholesterol storage 5/227 10/18866 5.79038E-08 4.37639E-07 1.65315E-07 PPARG/PPARD/PPARA/NR1H3/ABCG1 5

BP GO:0033083 regulation of immature T cell proliferation 5/227 10/18866 5.79038E-08 4.37639E-07 1.65315E-07 IL1B/IL1A/ERBB2/GNRH1/SHH 5

BP GO:0045348 positive regulation of MHC class II biosynthetic process 5/227 10/18866 5.79038E-08 4.37639E-07 1.65315E-07 IL10/TLR4/SIRT1/IFNG/IL4 5

BP GO:0015849 organic acid transport 19/227 353/18866 5.86768E-08 4.42777E-07 1.67256E-07 RXRA/PPARG/NOS2/AKT1/PPARD/IL1B/BDNF/NTRK2/SYK/ABCG2/EDN1/GJA1/PPARA/PSEN1/NR1H4/TRPV1/PLA2G4A/HRH3/DRD2 19

BP GO:0014855 striated muscle cell proliferation 10/227 83/18866 5.89235E-08 4.43231E-07 1.67428E-07 RXRA/MAPK1/PPARD/CDK1/MAPK14/STAT3/GJA1/CFLAR/SHH/TGFBR1 10

BP GO:1900046 regulation of hemostasis 10/227 83/18866 5.89235E-08 4.43231E-07 1.67428E-07 PLAU/PRKCA/NFE2L2/TLR4/SYK/F2/EDN1/PLAT/PLA2G4A/PRKG1 10

BP GO:0051147 regulation of muscle cell differentiation 14/227 186/18866 5.92191E-08 4.44751E-07 1.68002E-07 BCL2/TGFB1/BDNF/MAPK14/SIRT1/EDN1/SOD2/PPARA/CFLAR/MTOR/CDC42/MDM2/IL4/SHH 14

BP GO:0070059 intrinsic apoptotic signaling pathway in response to endoplasmic reticulum stress 9/227 63/18866 6.07383E-08 4.55442E-07 1.7204E-07 BCL2/BAX/BCL2L1/TP53/DDIT3/SIRT1/BAK1/TNFRSF10B/EIF2AK3 9

BP GO:0019218 regulation of steroid metabolic process 12/227 131/18866 6.19902E-08 4.64097E-07 1.75309E-07 TNF/SOD1/IL1B/SIRT1/AGTR1/NFKB1/IFNG/HMGCR/NR1H4/ABCG1/FDFT1/VDR 12

BP GO:0032675 regulation of interleukin-6 production 13/227 159/18866 6.72738E-08 5.02072E-07 1.89654E-07 IL10/TNF/IL6/IL1B/TLR4/APP/MMP8/SYK/STAT3/IL1A/IFNG/PTPN6/NR1H4 13

BP GO:0050777 negative regulation of immune response 13/227 159/18866 6.72738E-08 5.02072E-07 1.89654E-07 TGFB1/PPARG/IL10/TNF/HMOX1/MMP12/CCR2/NR1H3/IL2/IL4/PTPN6/GPX1/DRD2 13

BP GO:0010759 positive regulation of macrophage chemotaxis 6/227 19/18866 6.76138E-08 5.02243E-07 1.89719E-07 MAPK1/MAPK3/MAPK14/C5AR1/TRPV4/CSF1R 6

BP GO:0032930 positive regulation of superoxide anion generation 6/227 19/18866 6.76138E-08 5.02243E-07 1.89719E-07 TGFB1/EGFR/SOD1/SYK/CRP/GSTP1 6

BP GO:1902176 negative regulation of oxidative stress-induced intrinsic apoptotic signaling pathway 6/227 19/18866 6.76138E-08 5.02243E-07 1.89719E-07 AKT1/HIF1A/NFE2L2/SIRT1/SOD2/GPX1 6

BP GO:0010863 positive regulation of phospholipase C activity 8/227 46/18866 6.83599E-08 5.06204E-07 1.91215E-07 HTR2A/EGFR/ESR1/BDNF/NTRK2/SELE/C5AR1/HTR2B 8

BP GO:0048538 thymus development 8/227 46/18866 6.83599E-08 5.06204E-07 1.91215E-07 BCL2/MAPK1/SOD1/MAPK3/TYR/PSEN1/SHH/TGFBR1 8

BP GO:0070265 necrotic cell death 9/227 64/18866 6.99444E-08 5.17132E-07 1.95343E-07 BAX/CASP8/TNF/TP53/TLR4/FASLG/CFLAR/BIRC3/BIRC2 9

BP GO:0032386 regulation of intracellular transport 19/227 358/18866 7.32047E-08 5.40396E-07 2.04131E-07 PTGS2/BCL2/CASP8/TGFB1/MAP2/MAPK1/TP53/IL1B/MAPK3/CDK1/CDK5/MAPK14/MAPK8/CDC42/MDM2/ERBB2/IFNG/PSEN1/SHH 19

BP GO:0010959 regulation of metal ion transport 20/227 396/18866 7.38081E-08 5.43755E-07 2.054E-07 PTGS2/HTR2A/PIK3CG/BCL2/BAX/AKT1/EGF/SNCA/CDK5/F2/BAK1/GJA1/ICAM1/CCL2/IFNG/PSEN2/PTPN6/CCR1/BDKRB1/DRD2 20

BP GO:0010507 negative regulation of autophagy 10/227 85/18866 7.42316E-08 5.43755E-07 2.054E-07 BCL2/AKT1/IL10/TP53/HMOX1/SNCA/MCL1/STAT3/MTOR/HTR2B 10

BP GO:0045844 positive regulation of striated muscle tissue development 10/227 85/18866 7.42316E-08 5.43755E-07 2.054E-07 BCL2/TGFB1/MAPK1/CDK1/MAPK14/EDN1/MTOR/HMGCR/CREB1/SHH 10

BP GO:0048636 positive regulation of muscle organ development 10/227 85/18866 7.42316E-08 5.43755E-07 2.054E-07 BCL2/TGFB1/MAPK1/CDK1/MAPK14/EDN1/MTOR/HMGCR/CREB1/SHH 10

BP GO:0110110 positive regulation of animal organ morphogenesis 10/227 85/18866 7.42316E-08 5.43755E-07 2.054E-07 BAX/AR/VEGFA/MYC/GDNF/EDN1/CFLAR/CDC42/VDR/TGFBR1 10

BP GO:0051048 negative regulation of secretion 14/227 190/18866 7.72906E-08 5.65292E-07 2.13535E-07 OPRM1/EGF/IL1B/HMOX1/SNCA/EDN1/GJA1/CCR2/NR1H3/HMGCR/TNFRSF1A/TNFRSF1B/HRH3/DRD2 14

BP GO:0002286 T cell activation involved in immune response 11/227 109/18866 8.19454E-08 5.98416E-07 2.26047E-07 IL6/TP53/STAT3/ICAM1/CD80/MTOR/IL2/IFNG/IL4/PSEN1/EIF2AK4 11

BP GO:0042509 regulation of tyrosine phosphorylation of STAT protein 10/227 86/18866 8.31231E-08 6.05157E-07 2.28594E-07 TNF/IL6/STAT3/PECAM1/IL2/IFNG/IL4/CSF2/TNFRSF1A/CSF1R 10

BP GO:1901863 positive regulation of muscle tissue development 10/227 86/18866 8.31231E-08 6.05157E-07 2.28594E-07 BCL2/TGFB1/MAPK1/CDK1/MAPK14/EDN1/MTOR/HMGCR/CREB1/SHH 10

BP GO:0060485 mesenchyme development 17/227 290/18866 8.86877E-08 6.44681E-07 2.43523E-07 BCL2/TGFB1/MAPK1/IL6/HIF1A/STAT1/MYC/IL1B/GDNF/MAPK3/EDN1/MTOR/MDM2/SHH/TGFBR1/HTR2B/RET 17

BP GO:0048872 homeostasis of number of cells 16/227 256/18866 8.94498E-08 6.48238E-07 2.44867E-07 BCL2/BAX/CASP3/AKT1/VEGFA/IL6/SOD1/HIF1A/STAT1/HMOX1/CDK6/MAPK14/STAT3/BAK1/CCR2/IL2 16

BP GO:0090257 regulation of muscle system process 16/227 256/18866 8.94498E-08 6.48238E-07 2.44867E-07 PTGS2/CHRM3/PIK3CG/PRKCA/SOD1/PARP1/EDN1/FOXO1/PPARA/MTOR/KCNMA1/TNFRSF1A/TNFRSF1B/PRKG1/TACR1/PDE5A 16

BP GO:0009409 response to cold 8/227 48/18866 9.68353E-08 6.99627E-07 2.64279E-07 CASP8/PPARG/FOS/HSP90AA1/FOXO1/UCP2/EIF2AK3/EIF2AK4 8

BP GO:1900274 regulation of phospholipase C activity 8/227 48/18866 9.68353E-08 6.99627E-07 2.64279E-07 HTR2A/EGFR/ESR1/BDNF/NTRK2/SELE/C5AR1/HTR2B 8

BP GO:0045732 positive regulation of protein catabolic process 15/227 225/18866 9.86948E-08 7.11741E-07 2.68855E-07 AKT1/EGF/TNF/IL1B/NFE2L2/HSP90AA1/GCLC/FOXO1/GJA1/MDM2/IFNG/VCP/PSEN1/TNFRSF1B/MAPK9 15

BP GO:0032652 regulation of interleukin-1 production 11/227 111/18866 9.8961E-08 7.11741E-07 2.68855E-07 CASP8/IL10/TNF/IL6/TLR4/APP/STAT3/IFNG/GSTP1/CASP1/NR1H4 11

BP GO:0051149 positive regulation of muscle cell differentiation 11/227 111/18866 9.8961E-08 7.11741E-07 2.68855E-07 BCL2/TGFB1/MAPK14/SIRT1/EDN1/SOD2/MTOR/CDC42/MDM2/IL4/SHH 11

BP GO:2000146 negative regulation of cell motility 19/227 365/18866 9.91292E-08 7.11873E-07 2.68905E-07 BCL2/TGFB1/PPARG/DPP4/AKT1/TNF/PPARD/HMOX1/NFE2L2/CYP19A1/STAT3/GJA1/CCL2/IL4/GSTP1/GNRH1/SHH/PRKG1/DRD2 19

BP GO:1903038 negative regulation of leukocyte cell-cell adhesion 12/227 137/18866 1.01948E-07 7.3101E-07 2.76134E-07 CASP3/AKT1/IL10/PPARA/CD80/ERBB2/IL2/IL4/GNRH1/PTPN6/SHH/PDE5A 12

BP GO:0032755 positive regulation of interleukin-6 production 10/227 88/18866 1.03761E-07 7.42893E-07 2.80623E-07 TNF/IL6/IL1B/TLR4/APP/MMP8/SYK/STAT3/IL1A/IFNG 10

BP GO:0033079 immature T cell proliferation 5/227 11/18866 1.05117E-07 7.50152E-07 2.83365E-07 IL1B/IL1A/ERBB2/GNRH1/SHH 5

BP GO:0045670 regulation of osteoclast differentiation 9/227 67/18866 1.05249E-07 7.50152E-07 2.83365E-07 FOS/TNF/TLR4/CA2/MTOR/IFNG/IL4/CREB1/CCR1 9

BP GO:1900015 regulation of cytokine production involved in inflammatory response 9/227 67/18866 1.05249E-07 7.50152E-07 2.83365E-07 NOS2/TNF/IL6/TLR4/F2/MAPK14/STAT3/IL17B/ALOX5 9

BP GO:0044262 cellular carbohydrate metabolic process 17/227 294/18866 1.08006E-07 7.68655E-07 2.90354E-07 AKT1/TP53/GPT/SNCA/AKR1B1/MGAM/SIRT1/STAT3/FOXO1/PPARA/MTOR/INSR/TPI1/PLA2G4A/GLB1/EP300/IDH1 17

BP GO:0010039 response to iron ion 7/227 33/18866 1.08968E-07 7.74341E-07 2.92502E-07 SLC6A3/BCL2/HIF1A/HMOX1/SNCA/MDM2/DRD2 7

BP GO:0098754 detoxification 12/227 138/18866 1.10485E-07 7.83947E-07 2.9613E-07 PTGS2/TNF/SOD1/NFE2L2/CAT/ABCG2/MPO/SOD2/GSTP1/GPX1/GPX4/DHFR 12

BP GO:1903531 negative regulation of secretion by cell 13/227 166/18866 1.1179E-07 7.92028E-07 2.99183E-07 OPRM1/IL1B/HMOX1/SNCA/EDN1/GJA1/CCR2/NR1H3/HMGCR/TNFRSF1A/TNFRSF1B/HRH3/DRD2 13

BP GO:0050768 negative regulation of neurogenesis 17/227 295/18866 1.13401E-07 8.02246E-07 3.03043E-07 SLC6A4/MAP2/TNF/IL6/TP53/IL1B/BDNF/APP/CDK5/F2/STAT3/MDM2/GNRH1/TRPV4/PSEN1/SHH/EIF2AK4 17

BP GO:0002673 regulation of acute inflammatory response 8/227 49/18866 1.14539E-07 8.06697E-07 3.04724E-07 PTGS2/PIK3CG/PPARG/TNF/IL6/IL1B/IL4/GSTP1 8

BP GO:0090199 regulation of release of cytochrome c from mitochondria 8/227 49/18866 1.14539E-07 8.06697E-07 3.04724E-07 BAX/AKT1/BCL2L1/MMP9/TP53/BAK1/TNFSF10/GPX1 8

BP GO:1990090 cellular response to nerve growth factor stimulus 8/227 49/18866 1.14539E-07 8.06697E-07 3.04724E-07 AKT1/BDNF/NGF/NTRK1/NTRK2/APP/CREB1/TRPV1 8

BP GO:0007260 tyrosine phosphorylation of STAT protein 10/227 89/18866 1.15677E-07 8.13504E-07 3.07295E-07 TNF/IL6/STAT3/PECAM1/IL2/IFNG/IL4/CSF2/TNFRSF1A/CSF1R 10

BP GO:0001894 tissue homeostasis 16/227 261/18866 1.16685E-07 8.19379E-07 3.09514E-07 PTGS2/BCL2/BAX/PRKCA/EGFR/VEGFA/IL6/SOD1/TLR4/CA2/AKR1B1/SYK/GJA1/PECAM1/CCR2/CSF1R 16

BP GO:0072577 endothelial cell apoptotic process 9/227 68/18866 1.20046E-07 8.41738E-07 3.17961E-07 IL10/TNF/KDR/NFE2L2/FASLG/ICAM1/CCL2/IL4/CD40LG 9

BP GO:0048762 mesenchymal cell differentiation 15/227 229/18866 1.2427E-07 8.70075E-07 3.28665E-07 BCL2/TGFB1/MAPK1/IL6/HIF1A/STAT1/IL1B/GDNF/MAPK3/EDN1/MTOR/SHH/TGFBR1/HTR2B/RET 15

BP GO:0043470 regulation of carbohydrate catabolic process 10/227 90/18866 1.2878E-07 8.97684E-07 3.39094E-07 HTR2A/TP53/HIF1A/APP/STAT3/PPARA/IFNG/INSR/PSEN1/EP300 10

BP GO:0070542 response to fatty acid 10/227 90/18866 1.2878E-07 8.97684E-07 3.39094E-07 PTGS2/PON1/PPARG/AKT1/CAT/EDN1/FOXO1/GNRH1/CREB1/NR1H4 10

BP GO:1903351 cellular response to dopamine 10/227 90/18866 1.2878E-07 8.97684E-07 3.39094E-07 CHRM3/HTR2A/OPRM1/MAPK1/MAPK3/HTR2C/HRH3/HTR1A/DRD2/HTR2B 10

BP GO:0009110 vitamin biosynthetic process 6/227 21/18866 1.32531E-07 9.22477E-07 3.48459E-07 TNF/IL1B/NFKB1/IFNG/CYP3A4/VDR 6

BP GO:1904707 positive regulation of vascular associated smooth muscle cell proliferation 8/227 50/18866 1.34954E-07 9.3797E-07 3.54311E-07 JUN/MMP2/MMP9/IL10/TNF/EDN1/GJA1/MDM2 8

BP GO:0007202 activation of phospholipase C activity 7/227 34/18866 1.35823E-07 9.39886E-07 3.55035E-07 HTR2A/EGFR/BDNF/NTRK2/SELE/C5AR1/HTR2B 7

BP GO:0045907 positive regulation of vasoconstriction 7/227 34/18866 1.35823E-07 9.39886E-07 3.55035E-07 PTGS2/CHRM3/HTR2A/EGFR/AKT1/GJA1/ICAM1 7

BP GO:0050715 positive regulation of cytokine secretion 7/227 34/18866 1.35823E-07 9.39886E-07 3.55035E-07 IL10/TNF/SYK/IL1A/IFNG/DRD2/HTR2B 7

BP GO:0042698 ovulation cycle 9/227 69/18866 1.36622E-07 9.42671E-07 3.56087E-07 PGR/OPRM1/CASP3/EGFR/ESR1/MMP7/SIRT1/GNRH1/PAM 9

BP GO:0048662 negative regulation of smooth muscle cell proliferation 9/227 69/18866 1.36622E-07 9.42671E-07 3.56087E-07 PPARG/CDKN1A/IL10/PPARD/HMOX1/SOD2/IFNG/GSTP1/PRKG1 9

BP GO:0051100 negative regulation of binding 13/227 169/18866 1.37898E-07 9.50099E-07 3.58893E-07 BAX/JUN/MAP2/AKT1/CDKN1A/IL10/HMOX1/DDIT3/MAPK3/MAPK8/PPARA/EIF2S1/PSEN1 13

BP GO:1903350 response to dopamine 10/227 91/18866 1.43171E-07 9.84998E-07 3.72076E-07 CHRM3/HTR2A/OPRM1/MAPK1/MAPK3/HTR2C/HRH3/HTR1A/DRD2/HTR2B 10

BP GO:0031330 negative regulation of cellular catabolic process 16/227 265/18866 1.43684E-07 9.87101E-07 3.7287E-07 PIK3CG/BCL2/AKT1/IL10/TP53/HMOX1/GDNF/SNCA/MCL1/MAPK14/STAT3/MTOR/PRKCG/PSEN1/SHH/HTR2B 16

BP GO:0016052 carbohydrate catabolic process 14/227 200/18866 1.46184E-07 1.00282E-06 3.78809E-07 HTR2A/TP53/HIF1A/APP/MGAM/STAT3/PPARA/IFNG/INSR/TPI1/GM2A/PSEN1/GLB1/EP300 14

BP GO:0007162 negative regulation of cell adhesion 17/227 301/18866 1.51275E-07 1.03626E-06 3.91439E-07 CASP3/TGFB1/AKT1/VEGFA/IL10/MMP12/PPARA/CD80/ERBB2/IL2/IL4/GNRH1/TRPV4/PTPN6/SHH/PRKG1/PDE5A 17

BP GO:0002534 cytokine production involved in inflammatory response 9/227 70/18866 1.55154E-07 1.0613E-06 4.00898E-07 NOS2/TNF/IL6/TLR4/F2/MAPK14/STAT3/IL17B/ALOX5 9

BP GO:0046620 regulation of organ growth 11/227 116/18866 1.55952E-07 1.06522E-06 4.02381E-07 SLC6A4/AKT1/MAPK1/SOD1/CDK1/MAPK14/EDN1/GJA1/PPARA/MTOR/TGFBR1 11

BP GO:0030307 positive regulation of cell growth 13/227 171/18866 1.5822E-07 1.07895E-06 4.07566E-07 BCL2/EGFR/AKT1/VEGFA/BDNF/NGF/F2/EDN1/MTOR/CDC42/ERBB2/IL2/TGFBR1 13

BP GO:0006984 ER-nucleus signaling pathway 8/227 51/18866 1.58416E-07 1.07895E-06 4.07566E-07 TP53/NFE2L2/ATF3/DDIT3/CCL2/CXCL8/EIF2S1/EIF2AK3 8

BP GO:0032371 regulation of sterol transport 9/227 71/18866 1.75835E-07 1.19529E-06 4.51512E-07 PON1/PPARG/EGF/SIRT1/NR1H3/NFKB1/SHH/CETP/ABCG1 9

BP GO:0002532 production of molecular mediator involved in inflammatory response 10/227 93/18866 1.76251E-07 1.19529E-06 4.51512E-07 NOS2/TNF/IL6/TLR4/SYK/F2/MAPK14/STAT3/IL17B/ALOX5 10

BP GO:0032677 regulation of interleukin-8 production 10/227 93/18866 1.76251E-07 1.19529E-06 4.51512E-07 RELA/IL10/TNF/IL6/IL1B/TLR4/DDIT3/SYK/STAT3/CRP 10

BP GO:0032928 regulation of superoxide anion generation 6/227 22/18866 1.80404E-07 1.21997E-06 4.60836E-07 TGFB1/EGFR/SOD1/SYK/CRP/GSTP1 6

BP GO:0042359 vitamin D metabolic process 6/227 22/18866 1.80404E-07 1.21997E-06 4.60836E-07 TNF/IL1B/NFKB1/IFNG/CYP3A4/VDR 6

BP GO:1990089 response to nerve growth factor 8/227 52/18866 1.85293E-07 1.25125E-06 4.72653E-07 AKT1/BDNF/NGF/NTRK1/NTRK2/APP/CREB1/TRPV1 8

BP GO:0033559 unsaturated fatty acid metabolic process 11/227 118/18866 1.85879E-07 1.25343E-06 4.73475E-07 PTGS2/IL1B/MAPK3/SIRT1/EDN1/GSTP1/GPX1/GPX4/TNFRSF1A/ALOX5/PLA2G4A 11

BP GO:0015718 monocarboxylic acid transport 13/227 174/18866 1.93759E-07 1.30472E-06 4.92848E-07 RXRA/PPARG/NOS2/AKT1/PPARD/IL1B/SYK/ABCG2/EDN1/PPARA/NR1H4/PLA2G4A/DRD2 13

BP GO:0010660 regulation of muscle cell apoptotic process 10/227 94/18866 1.95178E-07 1.31056E-06 4.95054E-07 PPARG/TP53/HMOX1/NFE2L2/SIRT1/EDN1/SOD2/CFLAR/IFNG/HMGCR 10

BP GO:0051591 response to cAMP 10/227 94/18866 1.95178E-07 1.31056E-06 4.95054E-07 SLC6A3/PIK3CG/RELA/FOS/STAT1/APP/TYR/ALDH3A1/BIRC2/REN 10

BP GO:0034620 cellular response to unfolded protein 12/227 146/18866 2.05285E-07 1.37454E-06 5.19223E-07 BAX/NFE2L2/ATF3/DDIT3/BAK1/CCL2/CXCL8/EIF2S1/VCP/EIF2AK3/EP300/EIF2AK2 12

BP GO:0046631 alpha-beta T cell activation 12/227 146/18866 2.05285E-07 1.37454E-06 5.19223E-07 BCL2/IL6/SYK/STAT3/CD80/MTOR/CCR2/CD28/IL2/IFNG/IL4/SHH 12

BP GO:0042554 superoxide anion generation 7/227 36/18866 2.06493E-07 1.38069E-06 5.21545E-07 TGFB1/EGFR/SOD1/SYK/EDN1/CRP/GSTP1 7

BP GO:1901568 fatty acid derivative metabolic process 13/227 175/18866 2.07106E-07 1.38284E-06 5.22358E-07 PTGS2/PON1/IL1B/MAPK3/SYK/SIRT1/EDN1/GSTP1/GPX1/GPX4/TNFRSF1A/ALOX5/PLA2G4A 13

BP GO:0007589 body fluid secretion 10/227 95/18866 2.15867E-07 1.4341E-06 5.41722E-07 SLC6A3/CHRM3/VEGFA/XDH/HIF1A/EDN1/NR1H3/PAM/CREB1/VDR 10

BP GO:0097194 execution phase of apoptosis 10/227 95/18866 2.15867E-07 1.4341E-06 5.41722E-07 BAX/CASP9/CASP3/CASP8/AKT1/BCL2L1/IL6/TP53/CFLAR/CASP1 10

BP GO:0032731 positive regulation of interleukin-1 beta production 8/227 53/18866 2.1599E-07 1.4341E-06 5.41722E-07 CASP8/TNF/IL6/TLR4/APP/STAT3/IFNG/CASP1 8

BP GO:0061756 leukocyte adhesion to vascular endothelial cell 8/227 53/18866 2.1599E-07 1.4341E-06 5.41722E-07 RELA/TNF/IL6/ICAM1/SELE/VCAM1/CCR2/ALOX5 8

BP GO:0001654 eye development 19/227 384/18866 2.17676E-07 1.44328E-06 5.45188E-07 RXRA/SLC6A3/BCL2/BAX/JUN/ACHE/EGFR/VEGFA/HIF1A/BDNF/NTRK2/FASLG/STAT3/BAK1/PSEN1/SHH/DRD2/TGFBR1/RET 19

BP GO:0010611 regulation of cardiac muscle hypertrophy 9/227 73/18866 2.24488E-07 1.48637E-06 5.61466E-07 PRKCA/PARP1/EDN1/FOXO1/PPARA/MTOR/TNFRSF1A/TNFRSF1B/PDE5A 9

BP GO:0046034 ATP metabolic process 17/227 311/18866 2.40727E-07 1.59168E-06 6.01245E-07 HTR2A/TGFB1/TP53/HIF1A/SNCA/APP/CDK1/PARP1/STAT3/PPARA/IFNG/IL4/INSR/VCP/TPI1/PSEN1/EP300 17

BP GO:1901522 positive regulation of transcription from RNA polymerase II promoter involved in cellular response to chemical stimulus 6/227 23/18866 2.41631E-07 1.59544E-06 6.02665E-07 RELA/VEGFA/TP53/HIF1A/NFE2L2/EP300 6

BP GO:0006749 glutathione metabolic process 8/227 54/18866 2.50947E-07 1.65235E-06 6.24165E-07 SOD1/NFE2L2/GCLC/GLO1/GSTP1/GPX1/GPX4/IDH1 8

BP GO:0032757 positive regulation of interleukin-8 production 8/227 54/18866 2.50947E-07 1.65235E-06 6.24165E-07 RELA/TNF/IL6/IL1B/TLR4/DDIT3/SYK/STAT3 8

BP GO:0060969 negative regulation of gene silencing 7/227 37/18866 2.52084E-07 1.65298E-06 6.24401E-07 TGFB1/PPARG/TNF/IL6/TP53/ESR1/STAT3 7

BP GO:0097242 amyloid-beta clearance 7/227 37/18866 2.52084E-07 1.65298E-06 6.24401E-07 TNF/IGF1R/C5AR1/IFNG/IL4/INSR/HMGCR 7

BP GO:1904994 regulation of leukocyte adhesion to vascular endothelial cell 7/227 37/18866 2.52084E-07 1.65298E-06 6.24401E-07 RELA/TNF/IL6/ICAM1/SELE/CCR2/ALOX5 7

BP GO:1903320 regulation of protein modification by small protein conjugation or removal 15/227 242/18866 2.5431E-07 1.66527E-06 6.29045E-07 RELA/AKT1/HSP90AA1/GCLC/CDK5/BRCA1/XIAP/MTOR/BIRC3/VCP/PRKCG/PSEN1/BIRC2/MAPK9/TGFBR1 15

BP GO:0150063 visual system development 19/227 388/18866 2.5524E-07 1.66907E-06 6.30479E-07 RXRA/SLC6A3/BCL2/BAX/JUN/ACHE/EGFR/VEGFA/HIF1A/BDNF/NTRK2/FASLG/STAT3/BAK1/PSEN1/SHH/DRD2/TGFBR1/RET 19

BP GO:1901655 cellular response to ketone 10/227 97/18866 2.63095E-07 1.71807E-06 6.48987E-07 CASP9/AR/PPARG/EGFR/AKT1/AKR1B1/SIRT1/FOXO1/ICAM1/CFLAR 10

BP GO:0035264 multicellular organism growth 12/227 150/18866 2.75684E-07 1.79534E-06 6.78178E-07 SLC6A3/BCL2/AR/TP53/SOD1/APP/STAT3/MTOR/GAMT/CREB1/EP300/DRD2 12

BP GO:0045580 regulation of T cell differentiation 12/227 150/18866 2.75684E-07 1.79534E-06 6.78178E-07 SOD1/IL1B/SYK/IL1A/CD80/CCR2/CD28/ERBB2/IL2/IFNG/IL4/SHH 12

BP GO:0043627 response to estrogen 9/227 75/18866 2.84434E-07 1.84475E-06 6.96839E-07 AR/PPARG/MAPK1/ESR1/HMOX1/CA2/BRCA1/MDM2/EP300 9

BP GO:0045685 regulation of glial cell differentiation 9/227 75/18866 2.84434E-07 1.84475E-06 6.96839E-07 TGFB1/RELA/PPARG/IL6/CDK1/F2/MTOR/SHH/TNFRSF1B 9

BP GO:0050805 negative regulation of synaptic transmission 9/227 75/18866 2.84434E-07 1.84475E-06 6.96839E-07 PTGS2/HTR2A/SLC6A4/ACHE/IL1B/SNCA/BDNF/BCHE/DRD2 9

BP GO:0030656 regulation of vitamin metabolic process 5/227 13/18866 2.87119E-07 1.85962E-06 7.02458E-07 TNF/IL1B/NFKB1/IFNG/VDR 5

BP GO:0072521 purine-containing compound metabolic process 21/227 472/18866 2.88232E-07 1.86429E-06 7.04222E-07 HTR2A/TGFB1/NOS2/XDH/HIF1A/APP/PARP1/TTR/ABCG2/STAT3/PPARA/IFNG/IL4/INSR/VCP/TPI1/HMGCR/PSEN1/GPX1/EP300/PDE5A 21

BP GO:0010657 muscle cell apoptotic process 10/227 98/18866 2.89939E-07 1.87278E-06 7.07431E-07 PPARG/TP53/HMOX1/NFE2L2/SIRT1/EDN1/SOD2/CFLAR/IFNG/HMGCR 10

BP GO:0042593 glucose homeostasis 15/227 245/18866 2.97998E-07 1.92223E-06 7.26109E-07 PPARG/AKT1/IL6/HIF1A/GCLC/SIRT1/STAT3/IGF1R/FOXO1/ICAM1/INSR/TRPV4/HMGCR/NR1H4/ALOX5 15

BP GO:0030224 monocyte differentiation 7/227 38/18866 3.05864E-07 1.96308E-06 7.41541E-07 JUN/PPARG/VEGFA/MYC/CDK6/CSF2/CSF1R 7

BP GO:0032733 positive regulation of interleukin-10 production 7/227 38/18866 3.05864E-07 1.96308E-06 7.41541E-07 IL6/TLR4/SYK/STAT3/CD28/IL4/CD40LG 7

BP GO:1903131 mononuclear cell differentiation 7/227 38/18866 3.05864E-07 1.96308E-06 7.41541E-07 JUN/PPARG/VEGFA/MYC/CDK6/CSF2/CSF1R 7

BP GO:0045619 regulation of lymphocyte differentiation 13/227 181/18866 3.05983E-07 1.96308E-06 7.41541E-07 SOD1/IL1B/SYK/IL1A/CD80/CCR2/CD28/ERBB2/IL2/IFNG/IL4/PTPN6/SHH 13

BP GO:0050808 synapse organization 20/227 433/18866 3.1043E-07 1.98893E-06 7.51304E-07 CHRNA7/ACHE/IL10/TNF/GDNF/SNCA/BDNF/NTRK1/NTRK2/APP/CDK5/MAPK14/IGF1R/C5AR1/CDC42/ERBB2/INSR/VCP/PSEN1/DRD2 20

BP GO:0033500 carbohydrate homeostasis 15/227 246/18866 3.14002E-07 2.00911E-06 7.58926E-07 PPARG/AKT1/IL6/HIF1A/GCLC/SIRT1/STAT3/IGF1R/FOXO1/ICAM1/INSR/TRPV4/HMGCR/NR1H4/ALOX5 15

BP GO:0030073 insulin secretion 14/227 213/18866 3.16671E-07 2.02346E-06 7.64348E-07 PRKCA/DPP4/NOS2/TNF/IL6/HIF1A/PPARD/IL1B/GJA1/IFNG/HMGCR/NR1H4/ALOX5/DRD2 14

BP GO:0010869 regulation of receptor biosynthetic process 6/227 24/18866 3.18949E-07 2.02393E-06 7.64524E-07 PPARG/HIF1A/EDN1/PPARA/NR1H3/IFNG 6

BP GO:0036003 positive regulation of transcription from RNA polymerase II promoter in response to stress 6/227 24/18866 3.18949E-07 2.02393E-06 7.64524E-07 VEGFA/TP53/HIF1A/NFE2L2/ATF3/DDIT3 6

BP GO:0045821 positive regulation of glycolytic process 6/227 24/18866 3.18949E-07 2.02393E-06 7.64524E-07 HTR2A/HIF1A/APP/IFNG/INSR/PSEN1 6

BP GO:0051817 modulation of process of other organism involved in symbiotic interaction 10/227 99/18866 3.19156E-07 2.02393E-06 7.64524E-07 RXRA/JUN/CASP8/BCL2L1/CRP/INSR/CSF1R/EP300/EIF2AK4/EIF2AK2 10

BP GO:0014743 regulation of muscle hypertrophy 9/227 76/18866 3.19299E-07 2.02393E-06 7.64524E-07 PRKCA/PARP1/EDN1/FOXO1/PPARA/MTOR/TNFRSF1A/TNFRSF1B/PDE5A 9

BP GO:0033077 T cell differentiation in thymus 9/227 76/18866 3.19299E-07 2.02393E-06 7.64524E-07 BCL2/TP53/SOD1/IL1B/CDK6/IL1A/CD28/ERBB2/SHH 9

BP GO:0034329 cell junction assembly 20/227 434/18866 3.21994E-07 2.03829E-06 7.69951E-07 BCL2/PRKCA/ACHE/VEGFA/TNF/KDR/IL1B/SNCA/BDNF/NTRK1/NTRK2/APP/CDK5/GJA1/PECAM1/CDC42/GJB1/TRPV4/NR1H4/DRD2 20

BP GO:0048880 sensory system development 19/227 394/18866 3.22804E-07 2.0407E-06 7.70861E-07 RXRA/SLC6A3/BCL2/BAX/JUN/ACHE/EGFR/VEGFA/HIF1A/BDNF/NTRK2/FASLG/STAT3/BAK1/PSEN1/SHH/DRD2/TGFBR1/RET 19

BP GO:0006006 glucose metabolic process 14/227 214/18866 3.35268E-07 2.11595E-06 7.99284E-07 AKT1/TNF/TP53/PPARD/GPT/ATF3/MAPK14/SIRT1/FOXO1/PPARA/MTOR/INSR/TPI1/EP300 14

BP GO:0090183 regulation of kidney development 8/227 56/18866 3.35596E-07 2.11595E-06 7.99284E-07 VEGFA/MMP9/STAT1/MYC/GDNF/CFLAR/SHH/RET 8

BP GO:0006986 response to unfolded protein 13/227 183/18866 3.47296E-07 2.18682E-06 8.26055E-07 BAX/NFE2L2/ATF3/DDIT3/HSP90AA1/BAK1/CCL2/CXCL8/EIF2S1/VCP/EIF2AK3/EP300/EIF2AK2 13

BP GO:0045807 positive regulation of endocytosis 10/227 100/18866 3.50923E-07 2.20673E-06 8.33576E-07 PPARG/VEGFA/EGF/SNCA/SYK/SELE/CDC42/IL4/INSR/DRD2 10

BP GO:0040013 negative regulation of locomotion 19/227 397/18866 3.6239E-07 2.27583E-06 8.59678E-07 BCL2/TGFB1/PPARG/DPP4/AKT1/TNF/PPARD/HMOX1/NFE2L2/CYP19A1/STAT3/GJA1/CCL2/IL4/GSTP1/GNRH1/SHH/PRKG1/DRD2 19

BP GO:1904706 negative regulation of vascular associated smooth muscle cell proliferation 7/227 39/18866 3.68981E-07 2.31416E-06 8.74159E-07 PPARG/CDKN1A/IL10/HMOX1/SOD2/GSTP1/PRKG1 7

BP GO:0002695 negative regulation of leukocyte activation 13/227 184/18866 3.69769E-07 2.31605E-06 8.7487E-07 CASP3/IL10/HMOX1/CD80/CCR2/NR1H3/ERBB2/IL2/IL4/GNRH1/PTPN6/SHH/PDE5A 13

BP GO:0015908 fatty acid transport 10/227 101/18866 3.85428E-07 2.40737E-06 9.09368E-07 PPARG/NOS2/AKT1/PPARD/IL1B/SYK/EDN1/PPARA/PLA2G4A/DRD2 10

BP GO:0019217 regulation of fatty acid metabolic process 10/227 101/18866 3.85428E-07 2.40737E-06 9.09368E-07 PTGS2/PPARG/AKT1/PPARD/IL1B/SIRT1/BRCA1/PPARA/MTOR/NR1H3 10

BP GO:0001541 ovarian follicle development 8/227 57/18866 3.86375E-07 2.40737E-06 9.09368E-07 BCL2/BAX/VEGFA/BCL2L1/SOD1/ESR1/ICAM1/GNRH1 8

BP GO:0042743 hydrogen peroxide metabolic process 8/227 57/18866 3.86375E-07 2.40737E-06 9.09368E-07 MMP3/EGFR/SOD1/SNCA/CAT/MPO/STAT3/GPX1 8

BP GO:0061045 negative regulation of wound healing 9/227 78/18866 4.00294E-07 2.49084E-06 9.40896E-07 PLAU/TNF/F2/EDN1/GJA1/PLAT/HMGCR/ALOX5/PRKG1 9

BP GO:0051271 negative regulation of cellular component movement 19/227 400/18866 4.06369E-07 2.52272E-06 9.52939E-07 BCL2/TGFB1/PPARG/DPP4/AKT1/TNF/PPARD/HMOX1/NFE2L2/CYP19A1/STAT3/GJA1/CCL2/IL4/GSTP1/GNRH1/SHH/PRKG1/DRD2 19

BP GO:0007409 axonogenesis 21/227 482/18866 4.06479E-07 2.52272E-06 9.52939E-07 BCL2/PRKCA/MAP2/VEGFA/MAPK1/GDNF/BDNF/MAPK3/NGF/NTRK1/NTRK2/HSP90AA1/APP/CDK5/ERBB2/PSEN1/CREB1/SHH/CSF1R/DRD2/RET 21

BP GO:0002092 positive regulation of receptor internalization 6/227 25/18866 4.15467E-07 2.57514E-06 9.72739E-07 VEGFA/EGF/SYK/SELE/INSR/DRD2 6

BP GO:0000082 G1/S transition of mitotic cell cycle 16/227 287/18866 4.22561E-07 2.61421E-06 9.87499E-07 BCL2/BAX/EGFR/AKT1/CDKN1A/TP53/MYC/CDK1/CDK5/CDK6/CCL2/CDK7/MDM2/PTPN6/EP300/DHFR 16

BP GO:0055024 regulation of cardiac muscle tissue development 10/227 102/18866 4.22871E-07 2.61421E-06 9.87499E-07 TGFB1/MAPK1/CDK1/MAPK14/EDN1/GJA1/PPARA/MTOR/CREB1/TGFBR1 10

BP GO:0043558 regulation of translational initiation in response to stress 5/227 14/18866 4.42256E-07 2.72971E-06 1.03113E-06 EIF2S1/EIF2AK3/EIF2AK4/EIF2AK2/EIF2AK1 5

BP GO:0002209 behavioral defense response 7/227 40/18866 4.42703E-07 2.72971E-06 1.03113E-06 BCL2/DPP4/BDNF/HTR2C/GJA1/HTR1A/EP300 7

BP GO:0031294 lymphocyte costimulation 8/227 58/18866 4.43588E-07 2.73163E-06 1.03185E-06 DPP4/AKT1/CD80/CDC42/CD28/IL4/CD40LG/PTPN6 8

BP GO:0050672 negative regulation of lymphocyte proliferation 9/227 79/18866 4.47082E-07 2.74958E-06 1.03863E-06 CASP3/IL10/CD80/ERBB2/IL2/GNRH1/PTPN6/SHH/PDE5A 9

BP GO:0045926 negative regulation of growth 15/227 254/18866 4.72773E-07 2.90383E-06 1.0969E-06 SLC6A4/BCL2/TGFB1/MAP2/PPARG/CDKN1A/TP53/HIF1A/ESR2/PPARD/CDK5/SIRT1/GJA1/PPARA/BDKRB1 15

BP GO:0006110 regulation of glycolytic process 9/227 80/18866 4.98536E-07 3.05812E-06 1.15518E-06 HTR2A/HIF1A/APP/STAT3/PPARA/IFNG/INSR/PSEN1/EP300 9

BP GO:0043534 blood vessel endothelial cell migration 13/227 189/18866 5.02855E-07 3.08063E-06 1.16369E-06 PTGS2/PRKCA/TGFB1/PPARG/AKT1/VEGFA/TNF/HIF1A/KDR/HMOX1/NFE2L2/SIRT1/GPX1 13

BP GO:0003300 cardiac muscle hypertrophy 10/227 104/18866 5.07425E-07 3.10276E-06 1.17205E-06 PRKCA/PARP1/EDN1/FOXO1/PPARA/MTOR/TNFRSF1A/TNFRSF1B/PDE5A/HTR2B 10

BP GO:0042306 regulation of protein import into nucleus 8/227 59/18866 5.07897E-07 3.10276E-06 1.17205E-06 PTGS2/TGFB1/MAPK1/CDK1/MAPK14/IFNG/PSEN1/SHH 8

BP GO:0032409 regulation of transporter activity 16/227 291/18866 5.08425E-07 3.10276E-06 1.17205E-06 CHRM3/OPRM1/BCL2/PON1/PPARG/MMP9/SNCA/APP/CDK5/ABCB1/GJA1/CCL2/CCR2/IFNG/GRIA2/DRD2 16

BP GO:0006575 cellular modified amino acid metabolic process 13/227 190/18866 5.34111E-07 3.24644E-06 1.22632E-06 SOD1/NFE2L2/GCLC/FOLH1/GLO1/GSTP1/GAMT/CTSB/GPX1/GPX4/PLA2G4A/IDH1/DHFR 13

BP GO:0030308 negative regulation of cell growth 13/227 190/18866 5.34111E-07 3.24644E-06 1.22632E-06 BCL2/TGFB1/MAP2/PPARG/CDKN1A/TP53/ESR2/PPARD/CDK5/SIRT1/GJA1/PPARA/BDKRB1 13

BP GO:0019430 removal of superoxide radicals 6/227 26/18866 5.347E-07 3.24644E-06 1.22632E-06 TNF/SOD1/NFE2L2/MPO/SOD2/DHFR 6

BP GO:1905523 positive regulation of macrophage migration 6/227 26/18866 5.347E-07 3.24644E-06 1.22632E-06 MAPK1/MAPK3/MAPK14/C5AR1/TRPV4/CSF1R 6

BP GO:0002699 positive regulation of immune effector process 14/227 223/18866 5.52397E-07 3.34961E-06 1.26529E-06 TGFB1/NOS2/TNF/IL6/IL1B/HMOX1/TLR4/SYK/CD80/CCR2/CD28/IL2/IFNG/IL4 14

BP GO:0002824 positive regulation of adaptive immune response based on somatic recombination of immune receptors built from immunoglobulin superfamily domains 10/227 105/18866 5.54997E-07 3.35681E-06 1.26801E-06 TGFB1/TNF/IL6/IL1B/CD80/CCR2/CD28/IL2/IL4/PLA2G4A 10

BP GO:0044070 regulation of anion transport 10/227 105/18866 5.54997E-07 3.35681E-06 1.26801E-06 AKT1/IL1B/CA2/SYK/ABCB1/EDN1/MTOR/PSEN1/CETP/HRH3 10

BP GO:0010951 negative regulation of endopeptidase activity 15/227 258/18866 5.7666E-07 3.48341E-06 1.31583E-06 PTGS2/AKT1/VEGFA/MMP9/TNF/SNCA/NGF/APP/BIRC5/XIAP/CFLAR/BIRC3/MDM2/GPX1/BIRC2 15

BP GO:0032653 regulation of interleukin-10 production 8/227 60/18866 5.80013E-07 3.49206E-06 1.3191E-06 IL6/TLR4/MMP8/SYK/STAT3/CD28/IL4/CD40LG 8

BP GO:0032732 positive regulation of interleukin-1 production 8/227 60/18866 5.80013E-07 3.49206E-06 1.3191E-06 CASP8/TNF/IL6/TLR4/APP/STAT3/IFNG/CASP1 8

BP GO:0010595 positive regulation of endothelial cell migration 11/227 132/18866 5.80297E-07 3.49206E-06 1.3191E-06 PTGS2/PRKCA/TGFB1/AKT1/VEGFA/HIF1A/KDR/HMOX1/NFE2L2/SIRT1/EDN1 11

BP GO:0048568 embryonic organ development 20/227 451/18866 5.89734E-07 3.54437E-06 1.33886E-06 CASP8/TGFB1/EGFR/AKT1/VEGFA/MAPK1/IL10/TNF/TP53/SOD1/HIF1A/KDR/GDNF/MAPK3/EDN1/CXCL8/PSEN1/CSF2/SHH/TGFBR1 20

BP GO:0032611 interleukin-1 beta production 10/227 106/18866 6.06426E-07 3.6355E-06 1.37328E-06 CASP8/TNF/IL6/IL1B/TLR4/APP/STAT3/IFNG/GSTP1/CASP1 10

BP GO:0046632 alpha-beta T cell differentiation 10/227 106/18866 6.06426E-07 3.6355E-06 1.37328E-06 BCL2/IL6/SYK/STAT3/CD80/MTOR/IL2/IFNG/IL4/SHH 10

BP GO:0051146 striated muscle cell differentiation 16/227 295/18866 6.0979E-07 3.65106E-06 1.37916E-06 RXRA/BCL2/CASP3/TGFB1/AKT1/VEGFA/BDNF/CDK1/MAPK14/EDN1/PPARA/CFLAR/MTOR/IL4/SHH/GPX1 16

BP GO:0006509 membrane protein ectodomain proteolysis 7/227 42/18866 6.27658E-07 3.75332E-06 1.41779E-06 IL10/TNF/IL1B/IFNG/PSEN1/PSEN2/TNFRSF1B 7

BP GO:0001503 ossification 19/227 412/18866 6.35483E-07 3.79533E-06 1.43366E-06 PTGS2/BCL2/TGFB1/ACHE/EGFR/AKT1/MMP2/MAPK1/TNF/IL6/HIF1A/MAPK3/CAT/CDK6/MAPK14/SHH/ALOX5/CCR1/EIF2AK3 19

BP GO:0045346 regulation of MHC class II biosynthetic process 5/227 15/18866 6.56892E-07 3.91827E-06 1.4801E-06 IL10/TLR4/SIRT1/IFNG/IL4 5

BP GO:1902110 positive regulation of mitochondrial membrane permeability involved in apoptotic process 8/227 61/18866 6.60703E-07 3.93607E-06 1.48682E-06 BCL2/BAX/CASP8/TP53/GCLC/MAPK8/BAK1/ATF2 8

BP GO:0014897 striated muscle hypertrophy 10/227 107/18866 6.61977E-07 3.93872E-06 1.48782E-06 PRKCA/PARP1/EDN1/FOXO1/PPARA/MTOR/TNFRSF1A/TNFRSF1B/PDE5A/HTR2B 10

BP GO:0045672 positive regulation of osteoclast differentiation 6/227 27/18866 6.80589E-07 4.0444E-06 1.52774E-06 FOS/TNF/CA2/IFNG/CREB1/CCR1 6

BP GO:0002718 regulation of cytokine production involved in immune response 9/227 83/18866 6.84895E-07 4.06491E-06 1.53549E-06 TGFB1/IL10/TNF/IL6/IL1B/HMOX1/TLR4/CCR2/TNFRSF1B 9

BP GO:1905475 regulation of protein localization to membrane 13/227 195/18866 7.17973E-07 4.25592E-06 1.60764E-06 BCL2/CASP8/TGFB1/AR/EGFR/AKT1/BCL2L1/TNF/TP53/CDK5/MAPK8/ERBB2/IFNG 13

BP GO:0043535 regulation of blood vessel endothelial cell migration 12/227 164/18866 7.21874E-07 4.2684E-06 1.61236E-06 PTGS2/PRKCA/TGFB1/PPARG/AKT1/VEGFA/TNF/HIF1A/KDR/HMOX1/NFE2L2/SIRT1 12

BP GO:0046661 male sex differentiation 12/227 164/18866 7.21874E-07 4.2684E-06 1.61236E-06 BCL2/BAX/AR/BCL2L1/ESR1/NTRK1/ICAM1/TNFSF10/INSR/SHH/REN/TGFBR1 12

BP GO:1904589 regulation of protein import 8/227 62/18866 7.50792E-07 4.42288E-06 1.67071E-06 PTGS2/TGFB1/MAPK1/CDK1/MAPK14/IFNG/PSEN1/SHH 8

BP GO:2000351 regulation of endothelial cell apoptotic process 8/227 62/18866 7.50792E-07 4.42288E-06 1.67071E-06 TNF/KDR/NFE2L2/FASLG/ICAM1/CCL2/IL4/CD40LG 8

BP GO:2001244 positive regulation of intrinsic apoptotic signaling pathway 8/227 62/18866 7.50792E-07 4.42288E-06 1.67071E-06 BCL2/BAX/BCL2L1/TP53/SOD1/DDIT3/MCL1/SIRT1 8

BP GO:0014031 mesenchymal cell development 9/227 84/18866 7.59139E-07 4.461E-06 1.68511E-06 BCL2/MAPK1/HIF1A/GDNF/MAPK3/EDN1/SHH/HTR2B/RET 9

BP GO:0015844 monoamine transport 9/227 84/18866 7.59139E-07 4.461E-06 1.68511E-06 SLC6A3/HTR2A/SLC6A4/GDNF/SNCA/SYK/HRH3/HTR1A/DRD2 9

BP GO:0048863 stem cell differentiation 15/227 264/18866 7.71316E-07 4.52696E-06 1.71003E-06 MAPK1/TP53/HIF1A/ESR1/NFE2L2/GDNF/MAPK3/CDK6/STAT3/EDN1/BCHE/SHH/HTR2B/EIF2AK2/RET 15

BP GO:0014896 muscle hypertrophy 10/227 109/18866 7.86573E-07 4.61081E-06 1.7417E-06 PRKCA/PARP1/EDN1/FOXO1/PPARA/MTOR/TNFRSF1A/TNFRSF1B/PDE5A/HTR2B 10

BP GO:0035967 cellular response to topologically incorrect protein 12/227 166/18866 8.21639E-07 4.81043E-06 1.81711E-06 BAX/NFE2L2/ATF3/DDIT3/BAK1/CCL2/CXCL8/EIF2S1/VCP/EIF2AK3/EP300/EIF2AK2 12

BP GO:0032613 interleukin-10 production 8/227 63/18866 8.51163E-07 4.97104E-06 1.87778E-06 IL6/TLR4/MMP8/SYK/STAT3/CD28/IL4/CD40LG 8

BP GO:1902686 mitochondrial outer membrane permeabilization involved in programmed cell death 8/227 63/18866 8.51163E-07 4.97104E-06 1.87778E-06 BCL2/BAX/CASP8/TP53/GCLC/MAPK8/BAK1/ATF2 8

BP GO:0002360 T cell lineage commitment 6/227 28/18866 8.57535E-07 4.97768E-06 1.88028E-06 BCL2/IL6/TP53/STAT3/MTOR/SHH 6

BP GO:0045932 negative regulation of muscle contraction 6/227 28/18866 8.57535E-07 4.97768E-06 1.88028E-06 PTGS2/PIK3CG/SOD1/KCNMA1/PRKG1/PDE5A 6

BP GO:0071450 cellular response to oxygen radical 6/227 28/18866 8.57535E-07 4.97768E-06 1.88028E-06 TNF/SOD1/NFE2L2/MPO/SOD2/DHFR 6

BP GO:0071451 cellular response to superoxide 6/227 28/18866 8.57535E-07 4.97768E-06 1.88028E-06 TNF/SOD1/NFE2L2/MPO/SOD2/DHFR 6

BP GO:1990776 response to angiotensin 6/227 28/18866 8.57535E-07 4.97768E-06 1.88028E-06 PTGS2/RELA/NFE2L2/CA2/AGTR1/NFKB1 6

BP GO:0019058 viral life cycle 17/227 341/18866 8.71877E-07 5.0519E-06 1.90832E-06 HTR2A/BCL2/DPP4/EGFR/TNF/CDK1/ICAM1/CCL2/CXCL8/CD80/CDC42/CD28/VCP/CTSB/CCR5/EIF2AK4/EIF2AK2 17

BP GO:0032620 interleukin-17 production 7/227 44/18866 8.7351E-07 5.0519E-06 1.90832E-06 TGFB1/IL6/TLR4/DDIT3/IL2/IFNG/NR1H4 7

BP GO:1905521 regulation of macrophage migration 7/227 44/18866 8.7351E-07 5.0519E-06 1.90832E-06 MAPK1/MAPK3/CYP19A1/MAPK14/C5AR1/TRPV4/CSF1R 7

BP GO:0001776 leukocyte homeostasis 9/227 86/18866 9.28742E-07 5.36481E-06 2.02652E-06 BCL2/BAX/CASP3/AKT1/IL6/HIF1A/BAK1/CCR2/IL2 9

BP GO:0006909 phagocytosis 18/227 382/18866 9.30852E-07 5.37048E-06 2.02866E-06 PPARG/MAPK1/TNF/SOD1/IL1B/TLR4/MAPK3/HSP90AA1/SYK/CCL2/PECAM1/CRP/CCR2/CDC42/NR1H3/IFNG/PRKCG/EIF2AK1 18

BP GO:0045931 positive regulation of mitotic cell cycle 12/227 168/18866 9.33449E-07 5.37893E-06 2.03185E-06 PRKCA/EGFR/AKT1/EGF/IL1B/APP/CDK1/EDN1/IL1A/CD28/MDM2/INSR 12

BP GO:0009743 response to carbohydrate 14/227 233/18866 9.35193E-07 5.38246E-06 2.03318E-06 PTGS2/CASP3/HIF1A/PPARD/IL1B/CAT/GCLC/IGF1R/GJA1/ICAM1/PRKCB/GSTP1/HMGCR/NR1H4 14

BP GO:0019372 lipoxygenase pathway 5/227 16/18866 9.4613E-07 5.41916E-06 2.04705E-06 PTGS2/PON1/GPX1/GPX4/ALOX5 5

BP GO:0032225 regulation of synaptic transmission, dopaminergic 5/227 16/18866 9.4613E-07 5.41916E-06 2.04705E-06 PTGS2/SLC6A4/GDNF/SNCA/DRD2 5

BP GO:0045342 MHC class II biosynthetic process 5/227 16/18866 9.4613E-07 5.41916E-06 2.04705E-06 IL10/TLR4/SIRT1/IFNG/IL4 5

BP GO:0051770 positive regulation of nitric-oxide synthase biosynthetic process 5/227 16/18866 9.4613E-07 5.41916E-06 2.04705E-06 STAT1/KDR/TLR4/CCL2/IFNG 5

BP GO:0042130 negative regulation of T cell proliferation 8/227 64/18866 9.62766E-07 5.50781E-06 2.08053E-06 CASP3/IL10/CD80/ERBB2/GNRH1/PTPN6/SHH/PDE5A 8

BP GO:0006775 fat-soluble vitamin metabolic process 7/227 45/18866 1.02391E-06 5.83652E-06 2.2047E-06 TNF/PPARD/IL1B/NFKB1/IFNG/CYP3A4/VDR 7

BP GO:0034105 positive regulation of tissue remodeling 7/227 45/18866 1.02391E-06 5.83652E-06 2.2047E-06 BAX/PRKCA/EGFR/CA2/SYK/IL2/VDR 7

BP GO:1901028 regulation of mitochondrial outer membrane permeabilization involved in apoptotic signaling pathway 7/227 45/18866 1.02391E-06 5.83652E-06 2.2047E-06 BCL2/BAX/CASP8/TP53/GCLC/MAPK8/BAK1 7

BP GO:0051781 positive regulation of cell division 9/227 87/18866 1.02518E-06 5.83678E-06 2.2048E-06 TGFB1/VEGFA/IL1B/CAT/IL1A/CDC42/SHH/DRD2/HTR2B 9

BP GO:0045088 regulation of innate immune response 16/227 307/18866 1.03302E-06 5.87438E-06 2.21901E-06 RELA/PPARG/STAT1/TLR4/MMP12/SYK/XIAP/JAK1/BIRC3/NR1H3/NFKB1/IFNG/PTPN6/BIRC2/EP300/DRD2 16

BP GO:0019048 modulation by virus of host process 6/227 29/18866 1.07042E-06 6.07253E-06 2.29386E-06 RXRA/CASP8/BCL2L1/INSR/EIF2AK4/EIF2AK2 6

BP GO:0036336 dendritic cell migration 6/227 29/18866 1.07042E-06 6.07253E-06 2.29386E-06 PIK3CG/CCR2/CDC42/ALOX5/CCR1/CCR5 6

BP GO:0035794 positive regulation of mitochondrial membrane permeability 8/227 65/18866 1.08661E-06 6.15705E-06 2.32578E-06 BCL2/BAX/CASP8/TP53/GCLC/MAPK8/BAK1/ATF2 8

BP GO:0035821 modulation of process of other organism 10/227 113/18866 1.09852E-06 6.21713E-06 2.34848E-06 RXRA/JUN/CASP8/BCL2L1/CRP/INSR/CSF1R/EP300/EIF2AK4/EIF2AK2 10

BP GO:0008584 male gonad development 11/227 141/18866 1.1216E-06 6.33719E-06 2.39383E-06 BCL2/BAX/AR/BCL2L1/ESR1/NTRK1/ICAM1/TNFSF10/INSR/REN/TGFBR1 11

BP GO:0010466 negative regulation of peptidase activity 15/227 272/18866 1.12241E-06 6.33719E-06 2.39383E-06 PTGS2/AKT1/VEGFA/MMP9/TNF/SNCA/NGF/APP/BIRC5/XIAP/CFLAR/BIRC3/MDM2/GPX1/BIRC2 15

BP GO:0044843 cell cycle G1/S phase transition 16/227 310/18866 1.17371E-06 6.61901E-06 2.50028E-06 BCL2/BAX/EGFR/AKT1/CDKN1A/TP53/MYC/CDK1/CDK5/CDK6/CCL2/CDK7/MDM2/PTPN6/EP300/DHFR 16

BP GO:0010874 regulation of cholesterol efflux 7/227 46/18866 1.19542E-06 6.73343E-06 2.5435E-06 PON1/EGF/SIRT1/NR1H3/SHH/CETP/ABCG1 7

BP GO:0009615 response to virus 17/227 349/18866 1.1992E-06 6.74677E-06 2.54854E-06 BCL2/RELA/BCL2L1/TNF/IL6/ODC1/STAT1/IL1B/MMP12/CDK6/MAPK14/BIRC3/IFNG/IL4/BIRC2/EIF2AK4/EIF2AK2 17

BP GO:0007006 mitochondrial membrane organization 11/227 142/18866 1.20307E-06 6.75251E-06 2.55071E-06 BCL2/BAX/CASP8/BCL2L1/TP53/HSP90AA1/GCLC/STAT3/MAPK8/BAK1/ATF2 11

BP GO:0046546 development of primary male sexual characteristics 11/227 142/18866 1.20307E-06 6.75251E-06 2.55071E-06 BCL2/BAX/AR/BCL2L1/ESR1/NTRK1/ICAM1/TNFSF10/INSR/REN/TGFBR1 11

BP GO:1902108 regulation of mitochondrial membrane permeability involved in apoptotic process 8/227 66/18866 1.22379E-06 6.85267E-06 2.58855E-06 BCL2/BAX/CASP8/TP53/GCLC/MAPK8/BAK1/ATF2 8

BP GO:2000378 negative regulation of reactive oxygen species metabolic process 8/227 66/18866 1.22379E-06 6.85267E-06 2.58855E-06 BCL2/MMP3/IL10/TP53/HIF1A/STAT3/BRCA1/CFLAR 8

BP GO:0035966 response to topologically incorrect protein 13/227 205/18866 1.26299E-06 7.06382E-06 2.66831E-06 BAX/NFE2L2/ATF3/DDIT3/HSP90AA1/BAK1/CCL2/CXCL8/EIF2S1/VCP/EIF2AK3/EP300/EIF2AK2 13

BP GO:0006096 glycolytic process 10/227 115/18866 1.29152E-06 7.19797E-06 2.71898E-06 HTR2A/HIF1A/APP/STAT3/PPARA/IFNG/INSR/TPI1/PSEN1/EP300 10

BP GO:0031623 receptor internalization 10/227 115/18866 1.29152E-06 7.19797E-06 2.71898E-06 ACHE/VEGFA/EGF/SNCA/SYK/SELE/CXCL8/INSR/DRD2/HTR2B 10

BP GO:0043620 regulation of DNA-templated transcription in response to stress 10/227 115/18866 1.29152E-06 7.19797E-06 2.71898E-06 JUN/RELA/VEGFA/TP53/HIF1A/HMOX1/NFE2L2/ATF3/DDIT3/EP300 10

BP GO:0000303 response to superoxide 6/227 30/18866 1.32464E-06 7.36531E-06 2.78219E-06 TNF/SOD1/NFE2L2/MPO/SOD2/DHFR 6

BP GO:0048265 response to pain 6/227 30/18866 1.32464E-06 7.36531E-06 2.78219E-06 NTRK1/PRKCG/TRPV1/TACR1/SCN9A/RET 6

BP GO:0031649 heat generation 5/227 17/18866 1.32724E-06 7.37113E-06 2.78439E-06 PTGS2/TNF/IL1B/IL1A/TRPV1 5

BP GO:0010469 regulation of signaling receptor activity 12/227 174/18866 1.35412E-06 7.51163E-06 2.83746E-06 PLAU/OPRM1/IL10/EGF/ESR2/APP/CDK5/CCL2/CCR2/IFNG/PSEN1/GRIA2 12

BP GO:0045123 cellular extravasation 8/227 67/18866 1.37546E-06 7.61227E-06 2.87548E-06 PIK3CG/TNF/ICAM1/CCL2/SELE/VCAM1/PECAM1/CCR2 8

BP GO:1905710 positive regulation of membrane permeability 8/227 67/18866 1.37546E-06 7.61227E-06 2.87548E-06 BCL2/BAX/CASP8/TP53/GCLC/MAPK8/BAK1/ATF2 8

BP GO:0106106 cold-induced thermogenesis 11/227 144/18866 1.38176E-06 7.62939E-06 2.88195E-06 ACHE/VEGFA/TLR4/DDIT3/SYK/IGF1R/GJA1/CCR2/NR1H3/IL4/UCP2 11

BP GO:0120161 regulation of cold-induced thermogenesis 11/227 144/18866 1.38176E-06 7.62939E-06 2.88195E-06 ACHE/VEGFA/TLR4/DDIT3/SYK/IGF1R/GJA1/CCR2/NR1H3/IL4/UCP2 11

BP GO:0008542 visual learning 7/227 47/18866 1.39035E-06 7.65012E-06 2.88978E-06 HIF1A/APP/CDK5/MTOR/HMGCR/CREB1/DRD2 7

BP GO:0035722 interleukin-12-mediated signaling pathway 7/227 47/18866 1.39035E-06 7.65012E-06 2.88978E-06 IL10/SOD1/SOD2/JAK1/CDC42/IFNG/TYK2 7

BP GO:0070849 response to epidermal growth factor 7/227 47/18866 1.39035E-06 7.65012E-06 2.88978E-06 EGFR/AKT1/MAPK1/MAPK3/CFLAR/ERBB2/GSTP1 7

BP GO:0006757 ATP generation from ADP 10/227 116/18866 1.39865E-06 7.68689E-06 2.90367E-06 HTR2A/HIF1A/APP/STAT3/PPARA/IFNG/INSR/TPI1/PSEN1/EP300 10

BP GO:0006913 nucleocytoplasmic transport 17/227 354/18866 1.4566E-06 7.99615E-06 3.02049E-06 PTGS2/TGFB1/AKT1/CDKN1A/MAPK1/TP53/IL1B/MMP12/CDK1/CDK5/SYK/MAPK14/STAT3/MDM2/IFNG/PSEN1/SHH 17

BP GO:2000177 regulation of neural precursor cell proliferation 9/227 91/18866 1.50268E-06 8.23962E-06 3.11246E-06 SLC6A4/VEGFA/EGF/TP53/HIF1A/BDNF/FOXO1/SHH/DRD2 9

BP GO:0016241 regulation of macroautophagy 12/227 176/18866 1.52763E-06 8.36675E-06 3.16048E-06 CASP3/AKT1/TP53/HIF1A/KDR/HMOX1/MAPK3/CDK5/SIRT1/MAPK8/MTOR/IL4 12

BP GO:0002637 regulation of immunoglobulin production 8/227 68/18866 1.54285E-06 8.43069E-06 3.18463E-06 TGFB1/IL10/TNF/IL6/CD28/IL2/IL4/CD40LG 8

BP GO:0040014 regulation of multicellular organism growth 8/227 68/18866 1.54285E-06 8.43069E-06 3.18463E-06 SLC6A3/BCL2/SOD1/APP/STAT3/GAMT/CREB1/DRD2 8

BP GO:0042220 response to cocaine 7/227 48/18866 1.61119E-06 8.78394E-06 3.31807E-06 SLC6A3/HTR2A/OPRM1/CDK5/MTOR/MDM2/DRD2 7

BP GO:1904036 negative regulation of epithelial cell apoptotic process 7/227 48/18866 1.61119E-06 8.78394E-06 3.31807E-06 KDR/HMOX1/NFE2L2/ICAM1/CFLAR/MTOR/IL4 7

BP GO:0000305 response to oxygen radical 6/227 31/18866 1.62612E-06 8.83496E-06 3.33734E-06 TNF/SOD1/NFE2L2/MPO/SOD2/DHFR 6

BP GO:0014072 response to isoquinoline alkaloid 6/227 31/18866 1.62612E-06 8.83496E-06 3.33734E-06 OPRM1/RELA/MTOR/MDM2/PRKCG/DRD2 6

BP GO:0043278 response to morphine 6/227 31/18866 1.62612E-06 8.83496E-06 3.33734E-06 OPRM1/RELA/MTOR/MDM2/PRKCG/DRD2 6

BP GO:0051169 nuclear transport 17/227 357/18866 1.63405E-06 8.85014E-06 3.34308E-06 PTGS2/TGFB1/AKT1/CDKN1A/MAPK1/TP53/IL1B/MMP12/CDK1/CDK5/SYK/MAPK14/STAT3/MDM2/IFNG/PSEN1/SHH 17

BP GO:0007569 cell aging 10/227 118/18866 1.63636E-06 8.85014E-06 3.34308E-06 BCL2/CDKN1A/TP53/SOD1/CDK1/CDK6/MAPK14/SIRT1/ICAM1/MTOR 10

BP GO:0015918 sterol transport 10/227 118/18866 1.63636E-06 8.85014E-06 3.34308E-06 PON1/PPARG/EGF/SIRT1/NR1H3/NFKB1/NPC1L1/SHH/CETP/ABCG1 10

BP GO:0050868 negative regulation of T cell activation 10/227 118/18866 1.63636E-06 8.85014E-06 3.34308E-06 CASP3/IL10/CD80/ERBB2/IL2/IL4/GNRH1/PTPN6/SHH/PDE5A 10

BP GO:1905897 regulation of response to endoplasmic reticulum stress 9/227 92/18866 1.64839E-06 8.90504E-06 3.36382E-06 BAX/BCL2L1/NFE2L2/DDIT3/SIRT1/BAK1/NR1H3/ALOX5/EIF2AK3 9

BP GO:0045216 cell-cell junction organization 13/227 210/18866 1.65425E-06 8.92657E-06 3.37195E-06 PRKCA/TGFB1/VEGFA/TNF/IL1B/GJA1/PECAM1/CDC42/GJB1/TRPV4/NR1H4/CSF1R/TGFBR1 13

BP GO:0031396 regulation of protein ubiquitination 13/227 211/18866 1.74433E-06 9.402E-06 3.55154E-06 AKT1/HSP90AA1/GCLC/CDK5/BRCA1/XIAP/MTOR/BIRC3/PRKCG/PSEN1/BIRC2/MAPK9/TGFBR1 13

BP GO:0010822 positive regulation of mitochondrion organization 10/227 119/18866 1.7679E-06 9.50744E-06 3.59137E-06 BCL2/BAX/CASP8/MMP9/TP53/HIF1A/KDR/MAPK8/BAK1/TNFSF10 10

BP GO:0051153 regulation of striated muscle cell differentiation 10/227 119/18866 1.7679E-06 9.50744E-06 3.59137E-06 BCL2/TGFB1/BDNF/MAPK14/EDN1/PPARA/CFLAR/MTOR/IL4/SHH 10

BP GO:1903035 negative regulation of response to wounding 9/227 93/18866 1.80612E-06 9.70201E-06 3.66487E-06 PLAU/TNF/F2/EDN1/GJA1/PLAT/HMGCR/ALOX5/PRKG1 9

BP GO:0006163 purine nucleotide metabolic process 19/227 442/18866 1.80885E-06 9.70322E-06 3.66532E-06 HTR2A/TGFB1/NOS2/XDH/HIF1A/APP/PARP1/STAT3/PPARA/IFNG/IL4/INSR/VCP/TPI1/HMGCR/PSEN1/GPX1/EP300/PDE5A 19

BP GO:0010675 regulation of cellular carbohydrate metabolic process 11/227 148/18866 1.81042E-06 9.70322E-06 3.66532E-06 AKT1/TP53/GPT/SNCA/SIRT1/STAT3/FOXO1/PPARA/MTOR/INSR/EP300 11

BP GO:0010885 regulation of cholesterol storage 5/227 18/18866 1.81974E-06 9.70937E-06 3.66765E-06 PPARG/PPARD/PPARA/NR1H3/ABCG1 5

BP GO:0031293 membrane protein intracellular domain proteolysis 5/227 18/18866 1.81974E-06 9.70937E-06 3.66765E-06 TGFB1/RELA/NFKB1/PSEN1/PSEN2 5

BP GO:0071605 monocyte chemotactic protein-1 production 5/227 18/18866 1.81974E-06 9.70937E-06 3.66765E-06 IL1B/SYK/GSTP1/TRPV4/NR1H4 5

BP GO:0071637 regulation of monocyte chemotactic protein-1 production 5/227 18/18866 1.81974E-06 9.70937E-06 3.66765E-06 IL1B/SYK/GSTP1/TRPV4/NR1H4 5

BP GO:0006692 prostanoid metabolic process 7/227 49/18866 1.86063E-06 9.86114E-06 3.72498E-06 PTGS2/IL1B/SIRT1/EDN1/GSTP1/TNFRSF1A/PLA2G4A 7

BP GO:0006693 prostaglandin metabolic process 7/227 49/18866 1.86063E-06 9.86114E-06 3.72498E-06 PTGS2/IL1B/SIRT1/EDN1/GSTP1/TNFRSF1A/PLA2G4A 7

BP GO:0007595 lactation 7/227 49/18866 1.86063E-06 9.86114E-06 3.72498E-06 SLC6A3/VEGFA/XDH/HIF1A/PAM/CREB1/VDR 7

BP GO:0035196 production of miRNAs involved in gene silencing by miRNA 7/227 49/18866 1.86063E-06 9.86114E-06 3.72498E-06 TGFB1/EGFR/TNF/IL6/TP53/ESR1/STAT3 7

BP GO:0045058 T cell selection 7/227 49/18866 1.86063E-06 9.86114E-06 3.72498E-06 BCL2/IL6/SYK/STAT3/MTOR/CD28/SHH 7

BP GO:0071349 cellular response to interleukin-12 7/227 49/18866 1.86063E-06 9.86114E-06 3.72498E-06 IL10/SOD1/SOD2/JAK1/CDC42/IFNG/TYK2 7

BP GO:0032370 positive regulation of lipid transport 8/227 70/18866 1.93013E-06 1.01954E-05 3.85122E-06 PON1/IL1B/CYP19A1/SIRT1/EDN1/NR1H3/CETP/ABCG1 8

BP GO:0032374 regulation of cholesterol transport 8/227 70/18866 1.93013E-06 1.01954E-05 3.85122E-06 PON1/EGF/SIRT1/NR1H3/NFKB1/SHH/CETP/ABCG1 8

BP GO:0050795 regulation of behavior 8/227 70/18866 1.93013E-06 1.01954E-05 3.85122E-06 STAT3/GJA1/MTOR/CSF2/HTR1A/DRD2/HTR2B/EIF2AK4 8

BP GO:0006936 muscle contraction 17/227 362/18866 1.97362E-06 1.04135E-05 3.93363E-06 PTGS2/CHRM3/HTR2A/PIK3CG/SOD1/GDNF/EDN1/GJA1/MTOR/KCNMA1/GAMT/TRPV1/PRKG1/TACR1/DRD2/PDE5A/HTR2B 17

BP GO:1905954 positive regulation of lipid localization 9/227 94/18866 1.97669E-06 1.04181E-05 3.93538E-06 PON1/IL1B/CYP19A1/SIRT1/EDN1/NR1H3/NFKB1/CETP/ABCG1 9

BP GO:0008361 regulation of cell size 12/227 181/18866 2.05031E-06 1.07942E-05 4.07743E-06 MAP2/AKT1/VEGFA/BDNF/NGF/CDK5/EDN1/MTOR/KCNMA1/TRPV4/CREB1/RET 12

BP GO:0070671 response to interleukin-12 7/227 50/18866 2.14156E-06 1.12621E-05 4.25419E-06 IL10/SOD1/SOD2/JAK1/CDC42/IFNG/TYK2 7

BP GO:0035690 cellular response to drug 8/227 71/18866 2.15289E-06 1.13092E-05 4.27198E-06 NOS2/EGFR/TP53/MYC/IL1B/NFE2L2/EDN1/REN 8

BP GO:0006090 pyruvate metabolic process 11/227 152/18866 2.35172E-06 1.234E-05 4.66136E-06 HTR2A/HIF1A/APP/GLO1/STAT3/PPARA/IFNG/INSR/TPI1/PSEN1/EP300 11

BP GO:0045600 positive regulation of fat cell differentiation 8/227 72/18866 2.39715E-06 1.25645E-05 4.74616E-06 PTGS2/HTR2A/PPARG/AKT1/PPARD/HTR2C/MAPK14/CREB1 8

BP GO:0060749 mammary gland alveolus development 5/227 19/18866 2.4455E-06 1.27898E-05 4.83125E-06 AR/VEGFA/EGF/HIF1A/ESR1 5

BP GO:0061377 mammary gland lobule development 5/227 19/18866 2.4455E-06 1.27898E-05 4.83125E-06 AR/VEGFA/EGF/HIF1A/ESR1 5

BP GO:0071354 cellular response to interleukin-6 7/227 51/18866 2.45706E-06 1.28361E-05 4.84874E-06 RELA/IL6/STAT1/STAT3/ICAM1/JAK1/NFKB1 7

BP GO:0046031 ADP metabolic process 10/227 124/18866 2.57323E-06 1.33857E-05 5.05634E-06 HTR2A/HIF1A/APP/STAT3/PPARA/IFNG/INSR/TPI1/PSEN1/EP300 10

BP GO:0032651 regulation of interleukin-1 beta production 9/227 97/18866 2.57428E-06 1.33857E-05 5.05634E-06 CASP8/TNF/IL6/TLR4/APP/STAT3/IFNG/GSTP1/CASP1 9

BP GO:0042100 B cell proliferation 9/227 97/18866 2.57428E-06 1.33857E-05 5.05634E-06 BCL2/BAX/CASP3/CDKN1A/IL10/TLR4/IL2/IL4/CD40LG 9

BP GO:1901184 regulation of ERBB signaling pathway 9/227 97/18866 2.57428E-06 1.33857E-05 5.05634E-06 EGFR/AKT1/MMP9/EGF/APP/FASLG/CDC42/ERBB2/PSEN1 9

BP GO:2000045 regulation of G1/S transition of mitotic cell cycle 12/227 185/18866 2.57633E-06 1.33857E-05 5.05634E-06 BCL2/BAX/EGFR/AKT1/CDKN1A/TP53/CDK1/CDK6/CCL2/MDM2/PTPN6/EP300 12

BP GO:0070227 lymphocyte apoptotic process 8/227 73/18866 2.66455E-06 1.38289E-05 5.22377E-06 BAX/AKT1/IL10/TP53/HIF1A/FASLG/BAK1/IL2 8

BP GO:0002706 regulation of lymphocyte mediated immunity 11/227 154/18866 2.67208E-06 1.38528E-05 5.23281E-06 TGFB1/IL10/TNF/IL6/IL1B/CCR2/CD28/IL2/IL4/PTPN6/TNFRSF1B 11

BP GO:0048260 positive regulation of receptor-mediated endocytosis 7/227 52/18866 2.81044E-06 1.45543E-05 5.49778E-06 VEGFA/EGF/SYK/SELE/IL4/INSR/DRD2 7

BP GO:0098664 G protein-coupled serotonin receptor signaling pathway 6/227 34/18866 2.88209E-06 1.48929E-05 5.62568E-06 CHRM3/HTR2A/HTR2C/HRH3/HTR1A/HTR2B 6

BP GO:1902692 regulation of neuroblast proliferation 6/227 34/18866 2.88209E-06 1.48929E-05 5.62568E-06 VEGFA/TP53/HIF1A/BDNF/SHH/DRD2 6

BP GO:0003007 heart morphogenesis 14/227 258/18866 3.10977E-06 1.6052E-05 6.06351E-06 RXRA/JUN/TGFB1/VEGFA/TP53/HIF1A/MTOR/MDM2/INSR/PSEN1/ATF2/SHH/TGFBR1/HTR2B 14

BP GO:1905477 positive regulation of protein localization to membrane 10/227 127/18866 3.19591E-06 1.64787E-05 6.22472E-06 BCL2/CASP8/EGFR/AKT1/TNF/TP53/CDK5/MAPK8/ERBB2/IFNG 10

BP GO:0007632 visual behavior 7/227 53/18866 3.20525E-06 1.64911E-05 6.22938E-06 HIF1A/APP/CDK5/MTOR/HMGCR/CREB1/DRD2 7

BP GO:0045599 negative regulation of fat cell differentiation 7/227 53/18866 3.20525E-06 1.64911E-05 6.22938E-06 TGFB1/TNF/IL6/DDIT3/SIRT1/FOXO1/TRPV4 7

BP GO:0010565 regulation of cellular ketone metabolic process 12/227 189/18866 3.21807E-06 1.6505E-05 6.23466E-06 PTGS2/PPARG/AKT1/ODC1/PPARD/IL1B/SIRT1/BRCA1/PPARA/MTOR/NR1H3/NR1H4 12

BP GO:0002544 chronic inflammatory response 5/227 20/18866 3.22879E-06 1.6505E-05 6.23466E-06 IL10/TNF/CYP19A1/GJA1/IL4 5

BP GO:0010878 cholesterol storage 5/227 20/18866 3.22879E-06 1.6505E-05 6.23466E-06 PPARG/PPARD/PPARA/NR1H3/ABCG1 5

BP GO:0043555 regulation of translation in response to stress 5/227 20/18866 3.22879E-06 1.6505E-05 6.23466E-06 EIF2S1/EIF2AK3/EIF2AK4/EIF2AK2/EIF2AK1 5

BP GO:0071071 regulation of phospholipid biosynthetic process 5/227 20/18866 3.22879E-06 1.6505E-05 6.23466E-06 HTR2A/HTR2C/NR1H4/IDH1/HTR2B 5

BP GO:1900409 positive regulation of cellular response to oxidative stress 5/227 20/18866 3.22879E-06 1.6505E-05 6.23466E-06 MMP3/TNF/SOD1/TLR4/MCL1 5

BP GO:0007519 skeletal muscle tissue development 11/227 158/18866 3.42937E-06 1.75115E-05 6.61486E-06 BCL2/TGFB1/FOS/ATF3/CDK5/MAPK14/CFLAR/HMGCR/SHH/GPX1/EP300 11

BP GO:0071312 cellular response to alkaloid 6/227 35/18866 3.44362E-06 1.75466E-05 6.62809E-06 OPRM1/CASP3/BCL2L1/ICAM1/MDM2/TRPV1 6

BP GO:1901030 positive regulation of mitochondrial outer membrane permeabilization involved in apoptotic signaling pathway 6/227 35/18866 3.44362E-06 1.75466E-05 6.62809E-06 BCL2/BAX/CASP8/TP53/MAPK8/BAK1 6

BP GO:0008306 associative learning 8/227 76/18866 3.62381E-06 1.83859E-05 6.94514E-06 FOS/HIF1A/APP/CDK5/MTOR/HMGCR/CREB1/DRD2 8

BP GO:0071229 cellular response to acid chemical 8/227 76/18866 3.62381E-06 1.83859E-05 6.94514E-06 EGFR/VEGFA/BCL2L1/MMP2/TNF/NTRK2/GCLC/MTOR 8

BP GO:0072091 regulation of stem cell proliferation 8/227 76/18866 3.62381E-06 1.83859E-05 6.94514E-06 VEGFA/TP53/HIF1A/BDNF/GJA1/SHH/DRD2/EIF2AK2 8

BP GO:1903524 positive regulation of blood circulation 8/227 76/18866 3.62381E-06 1.83859E-05 6.94514E-06 PTGS2/CHRM3/HTR2A/EGFR/AKT1/EDN1/GJA1/ICAM1 8

BP GO:0031050 dsRNA processing 7/227 54/18866 3.64525E-06 1.84553E-05 6.97136E-06 TGFB1/EGFR/TNF/IL6/TP53/ESR1/STAT3 7

BP GO:0070918 production of small RNA involved in gene silencing by RNA 7/227 54/18866 3.64525E-06 1.84553E-05 6.97136E-06 TGFB1/EGFR/TNF/IL6/TP53/ESR1/STAT3 7

BP GO:0061448 connective tissue development 14/227 262/18866 3.718E-06 1.88036E-05 7.10291E-06 TGFB1/RELA/HIF1A/PPARD/MAPK3/MAPK14/SIRT1/EDN1/CFLAR/TRPV4/ATF2/NR1H4/EIF2AK3/TGFBR1 14

BP GO:0007187 G protein-coupled receptor signaling pathway, coupled to cyclic nucleotide second messenger 14/227 263/18866 3.8857E-06 1.96308E-05 7.41541E-06 CHRM3/HTR2A/OPRM1/PRKCA/HTR2C/EDN1/CCL2/GABBR1/HRH3/CCR1/CCR3/HTR1A/DRD2/HTR2B 14

BP GO:0043502 regulation of muscle adaptation 9/227 102/18866 3.91669E-06 1.97664E-05 7.46662E-06 PRKCA/PARP1/EDN1/FOXO1/PPARA/MTOR/TNFRSF1A/TNFRSF1B/PDE5A 9

BP GO:0045930 negative regulation of mitotic cell cycle 16/227 341/18866 4.02558E-06 2.02944E-05 7.66605E-06 BCL2/BAX/TGFB1/EGFR/BCL2L1/CDKN1A/IL10/TNF/TP53/CDK1/CDK6/CCL2/BRCA1/MDM2/ATF2/EP300 16

BP GO:0002676 regulation of chronic inflammatory response 4/227 10/18866 4.05006E-06 2.03531E-05 7.68823E-06 IL10/TNF/CYP19A1/IL4 4

BP GO:0010998 regulation of translational initiation by eIF2 alpha phosphorylation 4/227 10/18866 4.05006E-06 2.03531E-05 7.68823E-06 EIF2AK3/EIF2AK4/EIF2AK2/EIF2AK1 4

BP GO:0070424 regulation of nucleotide-binding oligomerization domain containing signaling pathway 4/227 10/18866 4.05006E-06 2.03531E-05 7.68823E-06 TLR4/XIAP/BIRC3/BIRC2 4

BP GO:0010543 regulation of platelet activation 6/227 36/18866 4.09104E-06 2.0494E-05 7.74146E-06 PRKCA/TLR4/SYK/F2/PLA2G4A/PRKG1 6

BP GO:0034405 response to fluid shear stress 6/227 36/18866 4.09104E-06 2.0494E-05 7.74146E-06 PTGS2/AKT1/NFE2L2/CA2/GJA1/CSF2 6

BP GO:2000310 regulation of NMDA receptor activity 6/227 36/18866 4.09104E-06 2.0494E-05 7.74146E-06 OPRM1/APP/CCL2/CCR2/IFNG/GRIA2 6

BP GO:0070741 response to interleukin-6 7/227 55/18866 4.13446E-06 2.0668E-05 7.80718E-06 RELA/IL6/STAT1/STAT3/ICAM1/JAK1/NFKB1 7

BP GO:0097345 mitochondrial outer membrane permeabilization 7/227 55/18866 4.13446E-06 2.0668E-05 7.80718E-06 BCL2/BAX/CASP8/TP53/GCLC/MAPK8/BAK1 7

BP GO:0006837 serotonin transport 5/227 21/18866 4.19638E-06 2.09554E-05 7.91577E-06 SLC6A4/SNCA/SYK/HRH3/HTR1A 5

BP GO:0006766 vitamin metabolic process 10/227 131/18866 4.22717E-06 2.10871E-05 7.96549E-06 TNF/PPARD/IL1B/GCLC/NFKB1/IFNG/CYP3A4/PRSS3/VDR/DHFR 10

BP GO:0001823 mesonephros development 9/227 103/18866 4.24732E-06 2.11535E-05 7.99059E-06 BCL2/VEGFA/MYC/GDNF/BDNF/CAT/SHH/REN/RET 9

BP GO:1903522 regulation of blood circulation 15/227 303/18866 4.24939E-06 2.11535E-05 7.99059E-06 PTGS2/CHRM3/HTR2A/PIK3CG/EGFR/AKT1/EDN1/GJA1/ICAM1/AGTR1/MDM2/IL2/TRPV1/DRD2/PDE5A 15

BP GO:0007219 Notch signaling pathway 12/227 195/18866 4.44502E-06 2.20811E-05 8.34098E-06 EGFR/AKT1/EGF/STAT1/MYC/APP/CDK6/STAT3/PSEN1/PSEN2/NR1H4/EP300 12

BP GO:0050864 regulation of B cell activation 12/227 195/18866 4.44502E-06 2.20811E-05 8.34098E-06 BCL2/CASP3/TGFB1/CDKN1A/IL10/IL6/TLR4/SYK/CD28/IL2/IL4/PTPN6 12

BP GO:0002712 regulation of B cell mediated immunity 7/227 56/18866 4.67716E-06 2.31376E-05 8.74006E-06 TGFB1/IL10/TNF/CD28/IL2/IL4/PTPN6 7

BP GO:0002889 regulation of immunoglobulin mediated immune response 7/227 56/18866 4.67716E-06 2.31376E-05 8.74006E-06 TGFB1/IL10/TNF/CD28/IL2/IL4/PTPN6 7

BP GO:0031295 T cell costimulation 7/227 56/18866 4.67716E-06 2.31376E-05 8.74006E-06 DPP4/AKT1/CD80/CDC42/CD28/CD40LG/PTPN6 7

BP GO:0060688 regulation of morphogenesis of a branching structure 7/227 56/18866 4.67716E-06 2.31376E-05 8.74006E-06 RXRA/AR/VEGFA/TNF/ESR1/GDNF/SHH 7

BP GO:0048738 cardiac muscle tissue development 13/227 231/18866 4.73805E-06 2.34144E-05 8.84463E-06 RXRA/TGFB1/VEGFA/MAPK1/CDK1/MAPK14/EDN1/GJA1/PPARA/MTOR/CDC42/CREB1/TGFBR1 13

BP GO:0070423 nucleotide-binding oligomerization domain containing signaling pathway 6/227 37/18866 4.83406E-06 2.38349E-05 9.00345E-06 CASP8/RELA/TLR4/XIAP/BIRC3/BIRC2 6

BP GO:0098926 postsynaptic signal transduction 6/227 37/18866 4.83406E-06 2.38349E-05 9.00345E-06 CHRM3/CHRNA7/OPRM1/RELA/STAT3/HRH3 6

BP GO:0006165 nucleoside diphosphate phosphorylation 10/227 133/18866 4.84318E-06 2.38349E-05 9.00345E-06 HTR2A/HIF1A/APP/STAT3/PPARA/IFNG/INSR/TPI1/PSEN1/EP300 10

BP GO:0042770 signal transduction in response to DNA damage 10/227 133/18866 4.84318E-06 2.38349E-05 9.00345E-06 BAX/CASP9/CDKN1A/TP53/CDK1/MAPK14/SIRT1/BRCA1/MDM2/EP300 10

BP GO:0006446 regulation of translational initiation 8/227 79/18866 4.86115E-06 2.38492E-05 9.00887E-06 RXRA/TNF/MTOR/EIF2S1/EIF2AK3/EIF2AK4/EIF2AK2/EIF2AK1 8

BP GO:0010827 regulation of glucose transmembrane transport 8/227 79/18866 4.86115E-06 2.38492E-05 9.00887E-06 AKT1/TNF/IL1B/NFE2L2/MAPK14/EDN1/PRKCB/INSR 8

BP GO:0031016 pancreas development 8/227 79/18866 4.86115E-06 2.38492E-05 9.00887E-06 AKT1/IL6/CDK6/BAK1/FOXO1/INSR/SHH/EIF2AK3 8

BP GO:0030301 cholesterol transport 9/227 105/18866 4.981E-06 2.4412E-05 9.22147E-06 PON1/EGF/SIRT1/NR1H3/NFKB1/NPC1L1/SHH/CETP/ABCG1 9

BP GO:0002312 B cell activation involved in immune response 8/227 80/18866 5.34588E-06 2.60927E-05 9.85634E-06 TGFB1/IL10/IL6/TLR4/CD28/IL2/IL4/CD40LG 8

BP GO:0014032 neural crest cell development 8/227 80/18866 5.34588E-06 2.60927E-05 9.85634E-06 MAPK1/HIF1A/GDNF/MAPK3/EDN1/SHH/HTR2B/RET 8

BP GO:0048678 response to axon injury 8/227 80/18866 5.34588E-06 2.60927E-05 9.85634E-06 BCL2/BAX/JUN/SOD1/NTRK1/CDK1/DHFR/DRD2 8

BP GO:0055021 regulation of cardiac muscle tissue growth 8/227 80/18866 5.34588E-06 2.60927E-05 9.85634E-06 MAPK1/CDK1/MAPK14/EDN1/GJA1/PPARA/MTOR/TGFBR1 8

BP GO:0043369 CD4-positive or CD8-positive, alpha-beta T cell lineage commitment 5/227 22/18866 5.37754E-06 2.61667E-05 9.88428E-06 BCL2/IL6/STAT3/MTOR/SHH 5

BP GO:0051000 positive regulation of nitric-oxide synthase activity 5/227 22/18866 5.37754E-06 2.61667E-05 9.88428E-06 AKT1/TNF/HIF1A/DHFR/HTR2B 5

BP GO:1902884 positive regulation of response to oxidative stress 5/227 22/18866 5.37754E-06 2.61667E-05 9.88428E-06 MMP3/TNF/SOD1/TLR4/MCL1 5

BP GO:0032091 negative regulation of protein binding 9/227 106/18866 5.38689E-06 2.61853E-05 9.89133E-06 BAX/MAP2/AKT1/CDKN1A/IL10/MAPK3/MAPK8/PPARA/EIF2S1 9

BP GO:1904062 regulation of cation transmembrane transport 16/227 349/18866 5.40416E-06 2.62425E-05 9.9129E-06 PIK3CG/OPRM1/BAX/MMP9/SNCA/APP/CDK5/F2/CCL2/CCR2/IFNG/PSEN2/PTPN6/GRIA2/BDKRB1/DRD2 16

BP GO:0006839 mitochondrial transport 14/227 271/18866 5.48891E-06 2.66268E-05 1.00581E-05 BCL2/BAX/CASP8/BCL2L1/TP53/HSP90AA1/GCLC/STAT3/MAPK8/BAK1/PSEN1/PSEN2/ATF2/UCP2 14

BP GO:0003158 endothelium development 10/227 135/18866 5.53552E-06 2.67982E-05 1.01228E-05 VEGFA/TNF/XDH/KDR/IL1B/GJA1/ICAM1/PECAM1/GPX1/TNFRSF1A 10

BP GO:0046939 nucleotide phosphorylation 10/227 135/18866 5.53552E-06 2.67982E-05 1.01228E-05 HTR2A/HIF1A/APP/STAT3/PPARA/IFNG/INSR/TPI1/PSEN1/EP300 10

BP GO:0032094 response to food 6/227 38/18866 5.6831E-06 2.73733E-05 1.03401E-05 OPRM1/AKT1/MPO/PPARA/MTOR/PRKCG 6

BP GO:0032885 regulation of polysaccharide biosynthetic process 6/227 38/18866 5.6831E-06 2.73733E-05 1.03401E-05 TGFB1/AKT1/EGF/MTOR/NFKB1/INSR 6

BP GO:0035872 nucleotide-binding domain, leucine rich repeat containing receptor signaling pathway 6/227 38/18866 5.6831E-06 2.73733E-05 1.03401E-05 CASP8/RELA/TLR4/XIAP/BIRC3/BIRC2 6

BP GO:0044003 modulation by symbiont of host process 6/227 38/18866 5.6831E-06 2.73733E-05 1.03401E-05 RXRA/CASP8/BCL2L1/INSR/EIF2AK4/EIF2AK2 6

BP GO:0045923 positive regulation of fatty acid metabolic process 6/227 38/18866 5.6831E-06 2.73733E-05 1.03401E-05 PTGS2/PPARG/PPARD/IL1B/PPARA/NR1H3 6

BP GO:0071695 anatomical structure maturation 13/227 235/18866 5.71174E-06 2.74834E-05 1.03817E-05 PGR/BCL2/PPARG/VEGFA/CDKN1A/MMP2/HIF1A/DDIT3/AKR1B1/APP/MTOR/REN/RET 13

BP GO:0055017 cardiac muscle tissue growth 9/227 107/18866 5.82081E-06 2.79799E-05 1.05692E-05 RXRA/MAPK1/CDK1/MAPK14/EDN1/GJA1/PPARA/MTOR/TGFBR1 9

BP GO:0001937 negative regulation of endothelial cell proliferation 8/227 81/18866 5.8709E-06 2.81922E-05 1.06494E-05 PPARG/TNF/XDH/STAT1/GJA1/CCL2/ALOX5/TGFBR1 8

BP GO:0009135 purine nucleoside diphosphate metabolic process 10/227 136/18866 5.91272E-06 2.83358E-05 1.07036E-05 HTR2A/HIF1A/APP/STAT3/PPARA/IFNG/INSR/TPI1/PSEN1/EP300 10

BP GO:0009179 purine ribonucleoside diphosphate metabolic process 10/227 136/18866 5.91272E-06 2.83358E-05 1.07036E-05 HTR2A/HIF1A/APP/STAT3/PPARA/IFNG/INSR/TPI1/PSEN1/EP300 10

BP GO:1900408 negative regulation of cellular response to oxidative stress 7/227 58/18866 5.9414E-06 2.84159E-05 1.07339E-05 AKT1/IL10/HIF1A/NFE2L2/SIRT1/SOD2/GPX1 7

BP GO:1903202 negative regulation of oxidative stress-induced cell death 7/227 58/18866 5.9414E-06 2.84159E-05 1.07339E-05 AKT1/IL10/HIF1A/NFE2L2/SIRT1/SOD2/GPX1 7

BP GO:0060538 skeletal muscle organ development 11/227 168/18866 6.19715E-06 2.96093E-05 1.11847E-05 BCL2/TGFB1/FOS/ATF3/CDK5/MAPK14/CFLAR/HMGCR/SHH/GPX1/EP300 11

BP GO:0001820 serotonin secretion 4/227 11/18866 6.30432E-06 3.00308E-05 1.13439E-05 SLC6A4/SYK/HRH3/HTR1A 4

BP GO:0070106 interleukin-27-mediated signaling pathway 4/227 11/18866 6.30432E-06 3.00308E-05 1.13439E-05 STAT1/STAT3/JAK1/TYK2 4

BP GO:1903800 positive regulation of production of miRNAs involved in gene silencing by miRNA 4/227 11/18866 6.30432E-06 3.00308E-05 1.13439E-05 TGFB1/EGFR/IL6/TP53 4

BP GO:0010594 regulation of endothelial cell migration 13/227 238/18866 6.55407E-06 3.11892E-05 1.17815E-05 PTGS2/PRKCA/TGFB1/PPARG/AKT1/VEGFA/TNF/HIF1A/KDR/HMOX1/NFE2L2/SIRT1/EDN1 13

BP GO:0001662 behavioral fear response 6/227 39/18866 6.6493E-06 3.14709E-05 1.18879E-05 BCL2/DPP4/BDNF/HTR2C/GJA1/HTR1A 6

BP GO:0007210 serotonin receptor signaling pathway 6/227 39/18866 6.6493E-06 3.14709E-05 1.18879E-05 CHRM3/HTR2A/HTR2C/HRH3/HTR1A/HTR2B 6

BP GO:0014037 Schwann cell differentiation 6/227 39/18866 6.6493E-06 3.14709E-05 1.18879E-05 RELA/AKT1/SOD1/NTRK2/CDK1/CDK5 6

BP GO:0032660 regulation of interleukin-17 production 6/227 39/18866 6.6493E-06 3.14709E-05 1.18879E-05 TGFB1/IL6/TLR4/DDIT3/IL2/IFNG 6

BP GO:0006636 unsaturated fatty acid biosynthetic process 7/227 59/18866 6.67283E-06 3.14709E-05 1.18879E-05 PTGS2/IL1B/SIRT1/EDN1/GSTP1/ALOX5/PLA2G4A 7

BP GO:0043030 regulation of macrophage activation 7/227 59/18866 6.67283E-06 3.14709E-05 1.18879E-05 IL10/IL6/TLR4/MMP8/NR1H3/IL4/PLA2G4A 7

BP GO:0046456 icosanoid biosynthetic process 7/227 59/18866 6.67283E-06 3.14709E-05 1.18879E-05 PTGS2/IL1B/SYK/SIRT1/EDN1/ALOX5/PLA2G4A 7

BP GO:0050707 regulation of cytokine secretion 7/227 59/18866 6.67283E-06 3.14709E-05 1.18879E-05 IL10/TNF/SYK/IL1A/IFNG/DRD2/HTR2B 7

BP GO:0071385 cellular response to glucocorticoid stimulus 7/227 59/18866 6.67283E-06 3.14709E-05 1.18879E-05 CASP9/EGFR/EDN1/FOXO1/ICAM1/CFLAR/GSTP1 7

BP GO:0046330 positive regulation of JNK cascade 10/227 138/18866 6.73436E-06 3.16981E-05 1.19737E-05 TNF/IL1B/TLR4/APP/MMP8/SYK/EDN1/CDC42/CD40LG/TRPV4 10

BP GO:0055076 transition metal ion homeostasis 10/227 138/18866 6.73436E-06 3.16981E-05 1.19737E-05 SOD1/HIF1A/MYC/HMOX1/APP/ABCG2/XIAP/IFNG/EIF2AK1/TMPRSS6 10

BP GO:0022898 regulation of transmembrane transporter activity 14/227 276/18866 6.76706E-06 3.18206E-05 1.202E-05 CHRM3/OPRM1/BCL2/MMP9/SNCA/APP/CDK5/ABCB1/GJA1/CCL2/CCR2/IFNG/GRIA2/DRD2 14

BP GO:0043618 regulation of transcription from RNA polymerase II promoter in response to stress 9/227 109/18866 6.77914E-06 3.18459E-05 1.20296E-05 JUN/VEGFA/TP53/HIF1A/HMOX1/NFE2L2/ATF3/DDIT3/EP300 9

BP GO:0051043 regulation of membrane protein ectodomain proteolysis 5/227 23/18866 6.80419E-06 3.18691E-05 1.20383E-05 IL10/TNF/IL1B/IFNG/TNFRSF1B 5

BP GO:1900017 positive regulation of cytokine production involved in inflammatory response 5/227 23/18866 6.80419E-06 3.18691E-05 1.20383E-05 TNF/IL6/TLR4/STAT3/IL17B 5

BP GO:1900273 positive regulation of long-term synaptic potentiation 5/227 23/18866 6.80419E-06 3.18691E-05 1.20383E-05 CHRNA7/APP/CREB1/DRD2/EIF2AK4 5

BP GO:0009185 ribonucleoside diphosphate metabolic process 10/227 139/18866 7.18097E-06 3.36007E-05 1.26924E-05 HTR2A/HIF1A/APP/STAT3/PPARA/IFNG/INSR/TPI1/PSEN1/EP300 10

BP GO:1904659 glucose transmembrane transport 9/227 110/18866 7.30693E-06 3.41565E-05 1.29024E-05 AKT1/TNF/PPARD/IL1B/NFE2L2/MAPK14/EDN1/PRKCB/INSR 9

BP GO:0051302 regulation of cell division 11/227 171/18866 7.33961E-06 3.42755E-05 1.29473E-05 TGFB1/VEGFA/BCL2L1/MYC/IL1B/CAT/IL1A/CDC42/SHH/DRD2/HTR2B 11

BP GO:0015980 energy derivation by oxidation of organic compounds 14/227 278/18866 7.34811E-06 3.42816E-05 1.29496E-05 NOS2/AKT1/TP53/HIF1A/MYC/SNCA/CAT/CDK1/MTOR/IFNG/IL4/INSR/VCP/IDH1 14

BP GO:0050994 regulation of lipid catabolic process 7/227 60/18866 7.47754E-06 3.48171E-05 1.31519E-05 PIK3CG/AKT1/TNF/IL1B/PPARA/MTOR/IDH1 7

BP GO:1902883 negative regulation of response to oxidative stress 7/227 60/18866 7.47754E-06 3.48171E-05 1.31519E-05 AKT1/IL10/HIF1A/NFE2L2/SIRT1/SOD2/GPX1 7

BP GO:0048864 stem cell development 8/227 84/18866 7.71513E-06 3.58882E-05 1.35565E-05 MAPK1/HIF1A/GDNF/MAPK3/EDN1/SHH/HTR2B/RET 8

BP GO:0016572 histone phosphorylation 6/227 40/18866 7.74459E-06 3.59198E-05 1.35684E-05 PRKCA/IL1B/MAPK3/CDK1/CDK5/PRKCB 6

BP GO:0090184 positive regulation of kidney development 6/227 40/18866 7.74459E-06 3.59198E-05 1.35684E-05 VEGFA/MYC/GDNF/CFLAR/SHH/RET 6

BP GO:0140353 lipid export from cell 6/227 40/18866 7.74459E-06 3.59198E-05 1.35684E-05 NOS2/IL1B/CYP19A1/EDN1/AGTR1/REN 6

BP GO:0002244 hematopoietic progenitor cell differentiation 11/227 172/18866 7.7592E-06 3.59525E-05 1.35808E-05 BCL2/TGFB1/TP53/KDR/NFE2L2/CDK6/PSEN1/PTPN6/SHH/CSF1R/EIF2AK2 11

BP GO:1902806 regulation of cell cycle G1/S phase transition 12/227 206/18866 7.79302E-06 3.6074E-05 1.36267E-05 BCL2/BAX/EGFR/AKT1/CDKN1A/TP53/CDK1/CDK6/CCL2/MDM2/PTPN6/EP300 12

BP GO:0034284 response to monosaccharide 12/227 207/18866 8.18592E-06 3.78559E-05 1.42998E-05 PTGS2/CASP3/HIF1A/PPARD/CAT/GCLC/IGF1R/GJA1/ICAM1/GSTP1/HMGCR/NR1H4 12

BP GO:0006633 fatty acid biosynthetic process 11/227 173/18866 8.19955E-06 3.78821E-05 1.43097E-05 PTGS2/IL1B/SIRT1/EDN1/BRCA1/NR1H3/GSTP1/CYP3A4/GPX4/ALOX5/PLA2G4A 11

BP GO:0032469 endoplasmic reticulum calcium ion homeostasis 5/227 24/18866 8.51083E-06 3.92059E-05 1.48097E-05 BCL2/BAX/APP/BAK1/PSEN1 5

BP GO:0090335 regulation of brown fat cell differentiation 5/227 24/18866 8.51083E-06 3.92059E-05 1.48097E-05 PTGS2/MAPK14/SIRT1/MTOR/TRPV4 5

BP GO:2000637 positive regulation of gene silencing by miRNA 5/227 24/18866 8.51083E-06 3.92059E-05 1.48097E-05 TGFB1/EGFR/IL6/TP53/STAT3 5

BP GO:0072073 kidney epithelium development 10/227 142/18866 8.67794E-06 3.9937E-05 1.50859E-05 BCL2/VEGFA/STAT1/MYC/GDNF/BDNF/CAT/PECAM1/SHH/RET 10

BP GO:0042596 fear response 6/227 41/18866 8.98164E-06 4.12548E-05 1.55837E-05 BCL2/DPP4/BDNF/HTR2C/GJA1/HTR1A 6

BP GO:0071548 response to dexamethasone 6/227 41/18866 8.98164E-06 4.12548E-05 1.55837E-05 CASP9/EGFR/EDN1/FOXO1/ICAM1/CFLAR 6

BP GO:0001676 long-chain fatty acid metabolic process 9/227 113/18866 9.10651E-06 4.1788E-05 1.57851E-05 PTGS2/MAPK3/GSTP1/CYP3A4/PAM/GPX1/GPX4/ALOX5/PLA2G4A 9

BP GO:0009791 post-embryonic development 8/227 86/18866 9.1987E-06 4.21703E-05 1.59295E-05 BCL2/BAX/VEGFA/BAK1/MTOR/PSEN1/SCN9A/TGFBR1 8

BP GO:0002260 lymphocyte homeostasis 7/227 62/18866 9.32981E-06 4.24114E-05 1.60206E-05 BCL2/BAX/CASP3/AKT1/HIF1A/BAK1/IL2 7

BP GO:0002294 CD4-positive, alpha-beta T cell differentiation involved in immune response 7/227 62/18866 9.32981E-06 4.24114E-05 1.60206E-05 IL6/STAT3/CD80/MTOR/IL2/IFNG/IL4 7

BP GO:0010676 positive regulation of cellular carbohydrate metabolic process 7/227 62/18866 9.32981E-06 4.24114E-05 1.60206E-05 AKT1/GPT/SNCA/SIRT1/FOXO1/PPARA/INSR 7

BP GO:0030888 regulation of B cell proliferation 7/227 62/18866 9.32981E-06 4.24114E-05 1.60206E-05 BCL2/CASP3/CDKN1A/IL10/TLR4/IL2/IL4 7

BP GO:0055025 positive regulation of cardiac muscle tissue development 7/227 62/18866 9.32981E-06 4.24114E-05 1.60206E-05 TGFB1/MAPK1/CDK1/MAPK14/EDN1/MTOR/CREB1 7

BP GO:0071384 cellular response to corticosteroid stimulus 7/227 62/18866 9.32981E-06 4.24114E-05 1.60206E-05 CASP9/EGFR/EDN1/FOXO1/ICAM1/CFLAR/GSTP1 7

BP GO:0072330 monocarboxylic acid biosynthetic process 13/227 246/18866 9.36002E-06 4.24114E-05 1.60206E-05 PTGS2/IL1B/SIRT1/EDN1/BRCA1/NR1H3/GSTP1/CYP3A4/GAMT/NR1H4/GPX4/ALOX5/PLA2G4A 13

BP GO:0036462 TRAIL-activated apoptotic signaling pathway 4/227 12/18866 9.36726E-06 4.24114E-05 1.60206E-05 CASP8/ATF3/TNFRSF10B/TNFRSF10A 4

BP GO:0051583 dopamine uptake involved in synaptic transmission 4/227 12/18866 9.36726E-06 4.24114E-05 1.60206E-05 SLC6A3/GDNF/SNCA/DRD2 4

BP GO:0051934 catecholamine uptake involved in synaptic transmission 4/227 12/18866 9.36726E-06 4.24114E-05 1.60206E-05 SLC6A3/GDNF/SNCA/DRD2 4

BP GO:0060736 prostate gland growth 4/227 12/18866 9.36726E-06 4.24114E-05 1.60206E-05 AR/ESR1/CYP19A1/SHH 4

BP GO:0071073 positive regulation of phospholipid biosynthetic process 4/227 12/18866 9.36726E-06 4.24114E-05 1.60206E-05 HTR2A/HTR2C/NR1H4/HTR2B 4

BP GO:1902337 regulation of apoptotic process involved in morphogenesis 4/227 12/18866 9.36726E-06 4.24114E-05 1.60206E-05 BAX/TNFRSF1A/TNFRSF1B/VDR 4

BP GO:0008645 hexose transmembrane transport 9/227 114/18866 9.78493E-06 4.42603E-05 1.6719E-05 AKT1/TNF/PPARD/IL1B/NFE2L2/MAPK14/EDN1/PRKCB/INSR 9

BP GO:0060078 regulation of postsynaptic membrane potential 10/227 144/18866 9.81939E-06 4.43739E-05 1.67619E-05 CHRNA7/OPRM1/AKT1/BDNF/APP/CDK5/GABBR1/GRM1/TRPV1/DRD2 10

BP GO:0055072 iron ion homeostasis 8/227 87/18866 1.00264E-05 4.52234E-05 1.70828E-05 SOD1/HIF1A/MYC/HMOX1/ABCG2/IFNG/EIF2AK1/TMPRSS6 8

BP GO:0060420 regulation of heart growth 8/227 87/18866 1.00264E-05 4.52234E-05 1.70828E-05 MAPK1/CDK1/MAPK14/EDN1/GJA1/PPARA/MTOR/TGFBR1 8

BP GO:0010907 positive regulation of glucose metabolic process 6/227 42/18866 1.0374E-05 4.67288E-05 1.76515E-05 AKT1/GPT/SIRT1/FOXO1/PPARA/INSR 6

BP GO:0002287 alpha-beta T cell activation involved in immune response 7/227 63/18866 1.03896E-05 4.67288E-05 1.76515E-05 IL6/STAT3/CD80/MTOR/IL2/IFNG/IL4 7

BP GO:0002293 alpha-beta T cell differentiation involved in immune response 7/227 63/18866 1.03896E-05 4.67288E-05 1.76515E-05 IL6/STAT3/CD80/MTOR/IL2/IFNG/IL4 7

BP GO:0060419 heart growth 9/227 115/18866 1.05061E-05 4.7158E-05 1.78136E-05 RXRA/MAPK1/CDK1/MAPK14/EDN1/GJA1/PPARA/MTOR/TGFBR1 9

BP GO:0060148 positive regulation of posttranscriptional gene silencing 5/227 25/18866 1.05347E-05 4.7158E-05 1.78136E-05 TGFB1/EGFR/IL6/TP53/STAT3 5

BP GO:0060330 regulation of response to interferon-gamma 5/227 25/18866 1.05347E-05 4.7158E-05 1.78136E-05 PPARG/STAT1/JAK1/NR1H3/IFNG 5

BP GO:0060334 regulation of interferon-gamma-mediated signaling pathway 5/227 25/18866 1.05347E-05 4.7158E-05 1.78136E-05 PPARG/STAT1/JAK1/NR1H3/IFNG 5

BP GO:1904385 cellular response to angiotensin 5/227 25/18866 1.05347E-05 4.7158E-05 1.78136E-05 RELA/NFE2L2/CA2/AGTR1/NFKB1 5

BP GO:0006644 phospholipid metabolic process 18/227 455/18866 1.0669E-05 4.77142E-05 1.80237E-05 HTR2A/PIK3CG/TGFB1/PON1/ACHE/PPARD/HTR2C/CDC42/NR1H3/ENPP2/NR1H4/GPX4/PLA2G4A/CETP/FDFT1/CSF1R/IDH1/HTR2B 18

BP GO:0070098 chemokine-mediated signaling pathway 8/227 88/18866 1.0916E-05 4.87733E-05 1.84238E-05 HIF1A/EDN1/CCL2/CXCL8/CCR2/CCR1/CCR3/CCR5 8

BP GO:0015749 monosaccharide transmembrane transport 9/227 116/18866 1.12721E-05 5.0317E-05 1.90069E-05 AKT1/TNF/PPARD/IL1B/NFE2L2/MAPK14/EDN1/PRKCB/INSR 9

BP GO:0048469 cell maturation 11/227 179/18866 1.13277E-05 5.05178E-05 1.90827E-05 PGR/BCL2/PPARG/VEGFA/CDKN1A/HIF1A/AKR1B1/APP/MTOR/REN/RET 11

BP GO:0001885 endothelial cell development 7/227 64/18866 1.15473E-05 5.14007E-05 1.94163E-05 VEGFA/TNF/IL1B/ICAM1/PECAM1/GPX1/TNFRSF1A 7

BP GO:0033344 cholesterol efflux 7/227 64/18866 1.15473E-05 5.14007E-05 1.94163E-05 PON1/EGF/SIRT1/NR1H3/SHH/CETP/ABCG1 7

BP GO:0014033 neural crest cell differentiation 8/227 89/18866 1.18713E-05 5.27932E-05 1.99423E-05 MAPK1/HIF1A/GDNF/MAPK3/EDN1/SHH/HTR2B/RET 8

BP GO:0045124 regulation of bone resorption 6/227 43/18866 1.19359E-05 5.30311E-05 2.00321E-05 PRKCA/EGFR/IL6/CA2/SYK/CSF1R 6

BP GO:0045446 endothelial cell differentiation 9/227 117/18866 1.20853E-05 5.36449E-05 2.0264E-05 VEGFA/TNF/XDH/KDR/IL1B/ICAM1/PECAM1/GPX1/TNFRSF1A 9

BP GO:0031644 regulation of nervous system process 10/227 148/18866 1.24959E-05 5.54156E-05 2.09328E-05 CHRNA7/OPRM1/IL10/APP/HTR2C/EDN1/MTOR/GRM1/TNFRSF1B/EIF2AK3 10

BP GO:1905330 regulation of morphogenesis of an epithelium 11/227 181/18866 1.2579E-05 5.5732E-05 2.10524E-05 RXRA/AR/VEGFA/TNF/STAT1/ESR1/GDNF/GJA1/MTOR/CDC42/SHH 11

BP GO:0043010 camera-type eye development 15/227 332/18866 1.27025E-05 5.6227E-05 2.12393E-05 RXRA/SLC6A3/BAX/JUN/ACHE/EGFR/VEGFA/HIF1A/NTRK2/BAK1/PSEN1/SHH/DRD2/TGFBR1/RET 15

BP GO:0006940 regulation of smooth muscle contraction 7/227 65/18866 1.28098E-05 5.6544E-05 2.13591E-05 PTGS2/CHRM3/SOD1/EDN1/KCNMA1/PRKG1/TACR1 7

BP GO:0007588 excretion 7/227 65/18866 1.28098E-05 5.6544E-05 2.13591E-05 HMOX1/ABCG2/EDN1/AGTR1/KCNMA1/TRPV1/DRD2 7

BP GO:0051205 protein insertion into membrane 7/227 65/18866 1.28098E-05 5.6544E-05 2.13591E-05 BCL2/BAX/CASP8/EGFR/TP53/HSP90AA1/MAPK8 7

BP GO:0042058 regulation of epidermal growth factor receptor signaling pathway 8/227 90/18866 1.28959E-05 5.68526E-05 2.14757E-05 EGFR/AKT1/MMP9/EGF/APP/FASLG/CDC42/PSEN1 8

BP GO:0032743 positive regulation of interleukin-2 production 5/227 26/18866 1.29156E-05 5.68526E-05 2.14757E-05 IL1B/IL1A/CD80/CCR2/CD28 5

BP GO:0060561 apoptotic process involved in morphogenesis 5/227 26/18866 1.29156E-05 5.68526E-05 2.14757E-05 BAX/BAK1/TNFRSF1A/TNFRSF1B/VDR 5

BP GO:0034219 carbohydrate transmembrane transport 9/227 118/18866 1.29481E-05 5.69429E-05 2.15098E-05 AKT1/TNF/PPARD/IL1B/NFE2L2/MAPK14/EDN1/PRKCB/INSR 9

BP GO:0010870 positive regulation of receptor biosynthetic process 4/227 13/18866 1.34029E-05 5.88884E-05 2.22447E-05 HIF1A/EDN1/NR1H3/IFNG 4

BP GO:0032881 regulation of polysaccharide metabolic process 6/227 44/18866 1.36826E-05 5.9896E-05 2.26253E-05 TGFB1/AKT1/EGF/MTOR/NFKB1/INSR 6

BP GO:0042088 T-helper 1 type immune response 6/227 44/18866 1.36826E-05 5.9896E-05 2.26253E-05 IL1B/TLR4/CD80/MTOR/CCR2/PLA2G4A 6

BP GO:0045687 positive regulation of glial cell differentiation 6/227 44/18866 1.36826E-05 5.9896E-05 2.26253E-05 TGFB1/RELA/PPARG/MTOR/SHH/TNFRSF1B 6

BP GO:1903053 regulation of extracellular matrix organization 6/227 44/18866 1.36826E-05 5.9896E-05 2.26253E-05 TGFB1/DPP4/IL6/CFLAR/TNFRSF1A/TNFRSF1B 6

BP GO:0071347 cellular response to interleukin-1 11/227 183/18866 1.39486E-05 6.10043E-05 2.30439E-05 RELA/IL6/HIF1A/IL1B/MAPK3/EDN1/ICAM1/CCL2/CXCL8/IL1A/NFKB1 11

BP GO:0046849 bone remodeling 8/227 91/18866 1.39941E-05 6.10908E-05 2.30766E-05 PRKCA/TGFB1/EGFR/IL6/CA2/SYK/GJA1/CSF1R 8

BP GO:0060333 interferon-gamma-mediated signaling pathway 8/227 91/18866 1.39941E-05 6.10908E-05 2.30766E-05 PPARG/TP53/STAT1/ICAM1/VCAM1/JAK1/NR1H3/IFNG 8

BP GO:0006959 humoral immune response 16/227 377/18866 1.41725E-05 6.1813E-05 2.33494E-05 BCL2/TNF/IL6/IL1B/F2/CCL2/CXCL8/CRP/C5AR1/CCR2/CD28/IFNG/PRSS3/PTPN6/ALOX5/C1R 16

BP GO:0000075 cell cycle checkpoint 12/227 219/18866 1.44462E-05 6.29489E-05 2.37785E-05 BAX/TGFB1/BCL2L1/CDKN1A/TP53/CDK1/MAPK14/BRCA1/MDM2/ATF2/EP300/EIF2AK4 12

BP GO:0000077 DNA damage checkpoint 10/227 151/18866 1.48955E-05 6.48472E-05 2.44956E-05 BAX/CDKN1A/TP53/CDK1/MAPK14/BRCA1/MDM2/ATF2/EP300/EIF2AK4 10

BP GO:0051348 negative regulation of transferase activity 14/227 296/18866 1.49235E-05 6.49097E-05 2.45192E-05 CASP3/PPARG/AKT1/CDKN1A/TP53/IL1B/SNCA/SIRT1/IGF1R/IFNG/GSTP1/HMGCR/PSEN1/PTPN6 14

BP GO:0045582 positive regulation of T cell differentiation 8/227 92/18866 1.51699E-05 6.5921E-05 2.49012E-05 IL1B/SYK/IL1A/CD80/IL2/IFNG/IL4/SHH 8

BP GO:0051607 defense response to virus 13/227 258/18866 1.55521E-05 6.75203E-05 2.55053E-05 BCL2/RELA/BCL2L1/IL6/STAT1/IL1B/MMP12/BIRC3/IFNG/IL4/BIRC2/EIF2AK4/EIF2AK2 13

BP GO:0031018 endocrine pancreas development 6/227 45/18866 1.56301E-05 6.76734E-05 2.55632E-05 AKT1/IL6/CDK6/BAK1/FOXO1/EIF2AK3 6

BP GO:0051204 protein insertion into mitochondrial membrane 6/227 45/18866 1.56301E-05 6.76734E-05 2.55632E-05 BCL2/BAX/CASP8/TP53/HSP90AA1/MAPK8 6

BP GO:0060443 mammary gland morphogenesis 6/227 45/18866 1.56301E-05 6.76734E-05 2.55632E-05 PGR/BAX/AR/ESR1/CSF1R/VDR 6

BP GO:1903672 positive regulation of sprouting angiogenesis 7/227 67/18866 1.56788E-05 6.77135E-05 2.55783E-05 PTGS2/VEGFA/IL10/KDR/HMOX1/JAK1/AGTR1 7

BP GO:0009651 response to salt stress 5/227 27/18866 1.56963E-05 6.77135E-05 2.55783E-05 BAX/TNF/TP53/AKR1B1/TRPV4 5

BP GO:0033081 regulation of T cell differentiation in thymus 5/227 27/18866 1.56963E-05 6.77135E-05 2.55783E-05 SOD1/IL1B/IL1A/ERBB2/SHH 5

BP GO:1903203 regulation of oxidative stress-induced neuron death 5/227 27/18866 1.56963E-05 6.77135E-05 2.55783E-05 IL10/HIF1A/TLR4/MCL1/PARP1 5

BP GO:0010906 regulation of glucose metabolic process 9/227 121/18866 1.58585E-05 6.83512E-05 2.58192E-05 AKT1/TP53/GPT/SIRT1/FOXO1/PPARA/MTOR/INSR/EP300 9

BP GO:0014909 smooth muscle cell migration 8/227 93/18866 1.64276E-05 7.074E-05 2.67215E-05 PLAU/BCL2/PPARD/NFE2L2/PLAT/MDM2/GSTP1/PRKG1 8

BP GO:1990266 neutrophil migration 9/227 122/18866 1.69448E-05 7.29011E-05 2.75379E-05 PIK3CG/DPP4/IL1B/SYK/EDN1/CCL2/CXCL8/PECAM1/C5AR1 9

BP GO:0051250 negative regulation of lymphocyte activation 10/227 154/18866 1.76816E-05 7.59333E-05 2.86832E-05 CASP3/IL10/CD80/ERBB2/IL2/IL4/GNRH1/PTPN6/SHH/PDE5A 10

BP GO:0061351 neural precursor cell proliferation 10/227 154/18866 1.76816E-05 7.59333E-05 2.86832E-05 SLC6A4/VEGFA/EGF/TP53/HIF1A/BDNF/FOXO1/C5AR1/SHH/DRD2 10

BP GO:0019319 hexose biosynthetic process 8/227 94/18866 1.77719E-05 7.62151E-05 2.87897E-05 GPT/ATF3/AKR1B1/SIRT1/FOXO1/PPARA/TPI1/EP300 8

BP GO:0032309 icosanoid secretion 6/227 46/18866 1.77953E-05 7.62151E-05 2.87897E-05 NOS2/IL1B/SYK/EDN1/PLA2G4A/DRD2 6

BP GO:0061028 establishment of endothelial barrier 6/227 46/18866 1.77953E-05 7.62151E-05 2.87897E-05 VEGFA/TNF/IL1B/ICAM1/PECAM1/TNFRSF1A 6

BP GO:0051346 negative regulation of hydrolase activity 18/227 473/18866 1.79527E-05 7.68201E-05 2.90182E-05 PTGS2/MAP2/AKT1/VEGFA/MMP9/TNF/TP53/SNCA/NGF/APP/SIRT1/BIRC5/XIAP/CFLAR/BIRC3/MDM2/GPX1/BIRC2 18

BP GO:0007173 epidermal growth factor receptor signaling pathway 9/227 123/18866 1.80939E-05 7.73545E-05 2.92201E-05 TGFB1/EGFR/AKT1/MMP9/EGF/APP/FASLG/CDC42/PSEN1 9

BP GO:0014857 regulation of skeletal muscle cell proliferation 4/227 14/18866 1.85871E-05 7.91785E-05 2.99091E-05 PPARD/STAT3/CFLAR/SHH 4

BP GO:0030213 hyaluronan biosynthetic process 4/227 14/18866 1.85871E-05 7.91785E-05 2.99091E-05 TGFB1/EGF/IL1B/NFKB1 4

BP GO:1903054 negative regulation of extracellular matrix organization 4/227 14/18866 1.85871E-05 7.91785E-05 2.99091E-05 TGFB1/DPP4/TNFRSF1A/TNFRSF1B 4

BP GO:1904748 regulation of apoptotic process involved in development 4/227 14/18866 1.85871E-05 7.91785E-05 2.99091E-05 BAX/TNFRSF1A/TNFRSF1B/VDR 4

BP GO:0009132 nucleoside diphosphate metabolic process 10/227 155/18866 1.87047E-05 7.9608E-05 3.00713E-05 HTR2A/HIF1A/APP/STAT3/PPARA/IFNG/INSR/TPI1/PSEN1/EP300 10

BP GO:0090200 positive regulation of release of cytochrome c from mitochondria 5/227 28/18866 1.89221E-05 8.03176E-05 3.03394E-05 BAX/MMP9/TP53/BAK1/TNFSF10 5

BP GO:1902932 positive regulation of alcohol biosynthetic process 5/227 28/18866 1.89221E-05 8.03176E-05 3.03394E-05 TNF/IL1B/SNCA/IFNG/ABCG1 5

BP GO:1903579 negative regulation of ATP metabolic process 5/227 28/18866 1.89221E-05 8.03176E-05 3.03394E-05 TP53/SNCA/PARP1/STAT3/PPARA 5

BP GO:0071230 cellular response to amino acid stimulus 7/227 69/18866 1.90593E-05 8.08277E-05 3.05321E-05 EGFR/BCL2L1/MMP2/TNF/NTRK2/GCLC/MTOR 7

BP GO:0003073 regulation of systemic arterial blood pressure 8/227 95/18866 1.92074E-05 8.13832E-05 3.07419E-05 AR/EDN1/GJA1/SOD2/AGTR1/TRPV1/REN/DRD2 8

BP GO:2001020 regulation of response to DNA damage stimulus 12/227 226/18866 1.97664E-05 8.36772E-05 3.16085E-05 BCL2/CASP9/EGFR/BCL2L1/TP53/MYC/MCL1/PARP1/SIRT1/BRCA1/MDM2/PRKCG 12

BP GO:0045776 negative regulation of blood pressure 6/227 47/18866 2.0196E-05 8.53439E-05 3.2238E-05 NOS2/SOD2/PPARA/TRPV1/BDKRB1/DRD2 6

BP GO:0048806 genitalia development 6/227 47/18866 2.0196E-05 8.53439E-05 3.2238E-05 BAX/AR/ESR1/CYP19A1/BAK1/SHH 6

BP GO:0003014 renal system process 9/227 125/18866 2.05918E-05 8.69392E-05 3.28407E-05 BCL2/AKR1B1/EDN1/GJA1/AGTR1/KCNMA1/TRPV1/REN/DRD2 9

BP GO:0048167 regulation of synaptic plasticity 11/227 191/18866 2.08055E-05 8.77632E-05 3.31519E-05 PTGS2/CHRNA7/MAPK1/BDNF/NTRK2/APP/CDK5/PSEN1/CREB1/DRD2/EIF2AK4 11

BP GO:0043271 negative regulation of ion transport 10/227 157/18866 2.09044E-05 8.81025E-05 3.32801E-05 PTGS2/HTR2A/BCL2/AKT1/MMP9/SNCA/ICAM1/MTOR/HRH3/DRD2 10

BP GO:0006879 cellular iron ion homeostasis 7/227 70/18866 2.09625E-05 8.82692E-05 3.33431E-05 SOD1/HIF1A/MYC/HMOX1/ABCG2/IFNG/TMPRSS6 7

BP GO:0002440 production of molecular mediator of immune response 14/227 306/18866 2.16004E-05 9.08744E-05 3.43272E-05 TGFB1/IL10/TNF/IL6/IL1B/HMOX1/TLR4/SIRT1/CCR2/CD28/IL2/IL4/CD40LG/TNFRSF1B 14

BP GO:2000134 negative regulation of G1/S transition of mitotic cell cycle 9/227 126/18866 2.1947E-05 9.2251E-05 3.48471E-05 BCL2/BAX/CDKN1A/TP53/CDK1/CDK6/CCL2/MDM2/EP300 9

BP GO:0035051 cardiocyte differentiation 10/227 158/18866 2.20852E-05 9.27499E-05 3.50356E-05 RXRA/TGFB1/EGFR/VEGFA/MAPK1/MAPK3/CDK1/EDN1/PPARA/MTOR 10

BP GO:0046634 regulation of alpha-beta T cell activation 8/227 97/18866 2.23718E-05 9.35404E-05 3.53342E-05 SYK/CD80/CCR2/CD28/IL2/IFNG/IL4/SHH 8

BP GO:0050764 regulation of phagocytosis 8/227 97/18866 2.23718E-05 9.35404E-05 3.53342E-05 PPARG/TNF/SOD1/IL1B/SYK/CCL2/IFNG/PRKCG 8

BP GO:0120162 positive regulation of cold-induced thermogenesis 8/227 97/18866 2.23718E-05 9.35404E-05 3.53342E-05 ACHE/VEGFA/SYK/IGF1R/GJA1/CCR2/IL4/UCP2 8

BP GO:1990868 response to chemokine 8/227 97/18866 2.23718E-05 9.35404E-05 3.53342E-05 HIF1A/EDN1/CCL2/CXCL8/CCR2/CCR1/CCR3/CCR5 8

BP GO:1990869 cellular response to chemokine 8/227 97/18866 2.23718E-05 9.35404E-05 3.53342E-05 HIF1A/EDN1/CCL2/CXCL8/CCR2/CCR1/CCR3/CCR5 8

BP GO:0050807 regulation of synapse organization 12/227 229/18866 2.25248E-05 9.40976E-05 3.55447E-05 IL10/TNF/SNCA/BDNF/NTRK1/NTRK2/APP/CDK5/MAPK14/CDC42/VCP/DRD2 12

BP GO:0001782 B cell homeostasis 5/227 29/18866 2.26412E-05 9.42525E-05 3.56032E-05 BCL2/BAX/CASP3/HIF1A/BAK1 5

BP GO:0032373 positive regulation of sterol transport 5/227 29/18866 2.26412E-05 9.42525E-05 3.56032E-05 PON1/SIRT1/NR1H3/CETP/ABCG1 5

BP GO:0032376 positive regulation of cholesterol transport 5/227 29/18866 2.26412E-05 9.42525E-05 3.56032E-05 PON1/SIRT1/NR1H3/CETP/ABCG1 5

BP GO:0050820 positive regulation of coagulation 5/227 29/18866 2.26412E-05 9.42525E-05 3.56032E-05 NFE2L2/TLR4/F2/PSEN1/PLA2G4A 5

BP GO:0033628 regulation of cell adhesion mediated by integrin 6/227 48/18866 2.2851E-05 9.49594E-05 3.58702E-05 PLAU/PIK3CG/DPP4/SYK/PTPN6/RET 6

BP GO:0051972 regulation of telomerase activity 6/227 48/18866 2.2851E-05 9.49594E-05 3.58702E-05 PPARG/MAPK1/TP53/MYC/MAPK3/HSP90AA1 6

BP GO:0002292 T cell differentiation involved in immune response 7/227 71/18866 2.30198E-05 9.54942E-05 3.60722E-05 IL6/STAT3/CD80/MTOR/IL2/IFNG/IL4 7

BP GO:0033627 cell adhesion mediated by integrin 7/227 71/18866 2.30198E-05 9.54942E-05 3.60722E-05 PLAU/PIK3CG/DPP4/SYK/ICAM1/PTPN6/RET 7

BP GO:0019079 viral genome replication 9/227 127/18866 2.33771E-05 9.68918E-05 3.66002E-05 BCL2/TNF/CCL2/CXCL8/CDC42/CD28/VCP/EIF2AK4/EIF2AK2 9

BP GO:0001657 ureteric bud development 8/227 98/18866 2.4111E-05 9.96728E-05 3.76507E-05 BCL2/VEGFA/MYC/GDNF/BDNF/CAT/SHH/RET 8

BP GO:0045833 negative regulation of lipid metabolic process 8/227 98/18866 2.4111E-05 9.96728E-05 3.76507E-05 PIK3CG/AKT1/TNF/SOD1/IL1B/SIRT1/BRCA1/NFKB1 8

BP GO:0048010 vascular endothelial growth factor receptor signaling pathway 8/227 98/18866 2.4111E-05 9.96728E-05 3.76507E-05 VEGFA/HIF1A/KDR/IL1B/HSP90AA1/MAPK14/PRKCB/CDC42 8

BP GO:0090596 sensory organ morphogenesis 13/227 269/18866 2.4135E-05 9.96854E-05 3.76554E-05 BCL2/BAX/VEGFA/MAPK1/SOD1/HIF1A/BDNF/MAPK3/NTRK2/FASLG/STAT3/BAK1/EDN1 13

BP GO:0072089 stem cell proliferation 9/227 128/18866 2.48857E-05 0.000102697 3.87929E-05 VEGFA/TP53/HIF1A/BDNF/ABCB1/GJA1/SHH/DRD2/EIF2AK2 9

BP GO:0016236 macroautophagy 14/227 310/18866 2.4933E-05 0.000102803 3.8833E-05 CASP3/AKT1/TP53/HIF1A/KDR/HMOX1/MAPK3/CDK5/SIRT1/MAPK8/MTOR/IL4/VCP/PSEN1 14

BP GO:0009415 response to water 4/227 15/18866 2.51072E-05 0.000103074 3.89355E-05 NTRK1/AKR1B1/ATF2/MAPK10 4

BP GO:0014856 skeletal muscle cell proliferation 4/227 15/18866 2.51072E-05 0.000103074 3.89355E-05 PPARD/STAT3/CFLAR/SHH 4

BP GO:0051044 positive regulation of membrane protein ectodomain proteolysis 4/227 15/18866 2.51072E-05 0.000103074 3.89355E-05 TNF/IL1B/IFNG/TNFRSF1B 4

BP GO:1901550 regulation of endothelial cell development 4/227 15/18866 2.51072E-05 0.000103074 3.89355E-05 VEGFA/TNF/IL1B/TNFRSF1A 4

BP GO:1903140 regulation of establishment of endothelial barrier 4/227 15/18866 2.51072E-05 0.000103074 3.89355E-05 VEGFA/TNF/IL1B/TNFRSF1A 4

BP GO:0050663 cytokine secretion 7/227 72/18866 2.52408E-05 0.000103533 3.91088E-05 IL10/TNF/SYK/IL1A/IFNG/DRD2/HTR2B 7

BP GO:0015695 organic cation transport 6/227 49/18866 2.57799E-05 0.00010529 3.97725E-05 SLC6A4/SNCA/SYK/PSEN1/HRH3/HTR1A 6

BP GO:0031279 regulation of cyclase activity 6/227 49/18866 2.57799E-05 0.00010529 3.97725E-05 NOS2/MAPK3/MAPK14/MAPK8/CCR2/DRD2 6

BP GO:0032663 regulation of interleukin-2 production 6/227 49/18866 2.57799E-05 0.00010529 3.97725E-05 IL1B/IL1A/CD80/CCR2/CD28/NR1H4 6

BP GO:0070231 T cell apoptotic process 6/227 49/18866 2.57799E-05 0.00010529 3.97725E-05 BAX/AKT1/TP53/HIF1A/FASLG/BAK1 6

BP GO:0090151 establishment of protein localization to mitochondrial membrane 6/227 49/18866 2.57799E-05 0.00010529 3.97725E-05 BCL2/BAX/CASP8/TP53/HSP90AA1/MAPK8 6

BP GO:0072163 mesonephric epithelium development 8/227 99/18866 2.59622E-05 0.000105733 3.99397E-05 BCL2/VEGFA/MYC/GDNF/BDNF/CAT/SHH/RET 8

BP GO:0072164 mesonephric tubule development 8/227 99/18866 2.59622E-05 0.000105733 3.99397E-05 BCL2/VEGFA/MYC/GDNF/BDNF/CAT/SHH/RET 8

BP GO:0031570 DNA integrity checkpoint 10/227 161/18866 2.59772E-05 0.000105733 3.99397E-05 BAX/CDKN1A/TP53/CDK1/MAPK14/BRCA1/MDM2/ATF2/EP300/EIF2AK4 10

BP GO:0034767 positive regulation of ion transmembrane transport 10/227 161/18866 2.59772E-05 0.000105733 3.99397E-05 BAX/SNCA/CDK5/ABCB1/F2/CCL2/CCR2/IFNG/PSEN1/BDKRB1 10

BP GO:0001516 prostaglandin biosynthetic process 5/227 30/18866 2.69045E-05 0.000108947 4.11541E-05 PTGS2/IL1B/SIRT1/EDN1/PLA2G4A 5

BP GO:0001844 protein insertion into mitochondrial membrane involved in apoptotic signaling pathway 5/227 30/18866 2.69045E-05 0.000108947 4.11541E-05 BCL2/BAX/CASP8/TP53/MAPK8 5

BP GO:0036475 neuron death in response to oxidative stress 5/227 30/18866 2.69045E-05 0.000108947 4.11541E-05 IL10/HIF1A/TLR4/MCL1/PARP1 5

BP GO:0046457 prostanoid biosynthetic process 5/227 30/18866 2.69045E-05 0.000108947 4.11541E-05 PTGS2/IL1B/SIRT1/EDN1/PLA2G4A 5

BP GO:0048147 negative regulation of fibroblast proliferation 5/227 30/18866 2.69045E-05 0.000108947 4.11541E-05 BAX/PPARG/TP53/MYC/GSTP1 5

BP GO:0060055 angiogenesis involved in wound healing 5/227 30/18866 2.69045E-05 0.000108947 4.11541E-05 VEGFA/TNF/KDR/GPX1/ALOX5 5

BP GO:0051155 positive regulation of striated muscle cell differentiation 7/227 73/18866 2.76352E-05 0.000111621 4.2164E-05 BCL2/TGFB1/MAPK14/EDN1/MTOR/IL4/SHH 7

BP GO:0072401 signal transduction involved in DNA integrity checkpoint 7/227 73/18866 2.76352E-05 0.000111621 4.2164E-05 BAX/CDKN1A/TP53/CDK1/BRCA1/MDM2/EP300 7

BP GO:0072422 signal transduction involved in DNA damage checkpoint 7/227 73/18866 2.76352E-05 0.000111621 4.2164E-05 BAX/CDKN1A/TP53/CDK1/BRCA1/MDM2/EP300 7

BP GO:0046364 monosaccharide biosynthetic process 8/227 100/18866 2.7931E-05 0.000112624 4.2543E-05 GPT/ATF3/AKR1B1/SIRT1/FOXO1/PPARA/TPI1/EP300 8

BP GO:0070301 cellular response to hydrogen peroxide 8/227 100/18866 2.7931E-05 0.000112624 4.2543E-05 RELA/IL10/IL6/NFE2L2/CDK1/SIRT1/FOXO1/MDM2 8

BP GO:0014009 glial cell proliferation 6/227 50/18866 2.90033E-05 0.000116454 4.39897E-05 TNF/IL6/IL1B/MTOR/CREB1/CSF1R 6

BP GO:0046850 regulation of bone remodeling 6/227 50/18866 2.90033E-05 0.000116454 4.39897E-05 PRKCA/EGFR/IL6/CA2/SYK/CSF1R 6

BP GO:0071715 icosanoid transport 6/227 50/18866 2.90033E-05 0.000116454 4.39897E-05 NOS2/IL1B/SYK/EDN1/PLA2G4A/DRD2 6

BP GO:1901571 fatty acid derivative transport 6/227 50/18866 2.90033E-05 0.000116454 4.39897E-05 NOS2/IL1B/SYK/EDN1/PLA2G4A/DRD2 6

BP GO:1903727 positive regulation of phospholipid metabolic process 6/227 50/18866 2.90033E-05 0.000116454 4.39897E-05 HTR2A/TGFB1/HTR2C/CDC42/NR1H4/HTR2B 6

BP GO:0001505 regulation of neurotransmitter levels 12/227 235/18866 2.9063E-05 0.000116595 4.4043E-05 SLC6A3/HTR2A/SLC6A4/ACHE/GDNF/SNCA/CDK5/PRKCB/PRKCG/PSEN1/HRH3/DRD2 12

BP GO:0010508 positive regulation of autophagy 9/227 131/18866 2.99168E-05 0.000119819 4.52606E-05 HIF1A/KDR/HMOX1/MAPK3/SIRT1/FOXO1/IFNG/IL4/EIF2AK4 9

BP GO:0046887 positive regulation of hormone secretion 9/227 131/18866 2.99168E-05 0.000119819 4.52606E-05 EGFR/HIF1A/PPARD/CYP19A1/EDN1/GJA1/CREB1/NR1H4/DRD2 9

BP GO:0009150 purine ribonucleotide metabolic process 16/227 401/18866 3.00809E-05 0.000120374 4.54706E-05 HTR2A/TGFB1/HIF1A/APP/PARP1/STAT3/PPARA/IFNG/IL4/INSR/VCP/TPI1/HMGCR/PSEN1/EP300/PDE5A 16

BP GO:0072395 signal transduction involved in cell cycle checkpoint 7/227 74/18866 3.02135E-05 0.000120803 4.56326E-05 BAX/CDKN1A/TP53/CDK1/BRCA1/MDM2/EP300 7

BP GO:0002675 positive regulation of acute inflammatory response 5/227 31/18866 3.17658E-05 0.000126619 4.78296E-05 PTGS2/PIK3CG/TNF/IL6/IL1B 5

BP GO:0045940 positive regulation of steroid metabolic process 5/227 31/18866 3.17658E-05 0.000126619 4.78296E-05 TNF/IL1B/AGTR1/IFNG/ABCG1 5

BP GO:0071549 cellular response to dexamethasone stimulus 5/227 31/18866 3.17658E-05 0.000126619 4.78296E-05 CASP9/EGFR/FOXO1/ICAM1/CFLAR 5

BP GO:1902807 negative regulation of cell cycle G1/S phase transition 9/227 132/18866 3.17747E-05 0.000126619 4.78296E-05 BCL2/BAX/CDKN1A/TP53/CDK1/CDK6/CCL2/MDM2/EP300 9

BP GO:1903076 regulation of protein localization to plasma membrane 8/227 102/18866 3.22451E-05 0.000128279 4.84564E-05 TGFB1/AR/EGFR/AKT1/BCL2L1/TNF/CDK5/IFNG 8

BP GO:2000060 positive regulation of ubiquitin-dependent protein catabolic process 8/227 102/18866 3.22451E-05 0.000128279 4.84564E-05 AKT1/EGF/NFE2L2/GCLC/MDM2/VCP/PSEN1/MAPK9 8

BP GO:0002204 somatic recombination of immunoglobulin genes involved in immune response 6/227 51/18866 3.25428E-05 0.000128924 4.87E-05 TGFB1/IL10/CD28/IL2/IL4/CD40LG 6

BP GO:0002208 somatic diversification of immunoglobulins involved in immune response 6/227 51/18866 3.25428E-05 0.000128924 4.87E-05 TGFB1/IL10/CD28/IL2/IL4/CD40LG 6

BP GO:0035272 exocrine system development 6/227 51/18866 3.25428E-05 0.000128924 4.87E-05 TGFB1/EGFR/TNF/CDC42/INSR/SHH 6

BP GO:0045190 isotype switching 6/227 51/18866 3.25428E-05 0.000128924 4.87E-05 TGFB1/IL10/CD28/IL2/IL4/CD40LG 6

BP GO:0048146 positive regulation of fibroblast proliferation 6/227 51/18866 3.25428E-05 0.000128924 4.87E-05 JUN/EGFR/CDKN1A/ESR1/MYC/CDK6 6

BP GO:0002070 epithelial cell maturation 4/227 16/18866 3.31609E-05 0.000131045 4.95013E-05 PGR/CDKN1A/HIF1A/AKR1B1 4

BP GO:0021534 cell proliferation in hindbrain 4/227 16/18866 3.31609E-05 0.000131045 4.95013E-05 SLC6A4/EGF/C5AR1/SHH 4

BP GO:0034374 low-density lipoprotein particle remodeling 4/227 16/18866 3.31609E-05 0.000131045 4.95013E-05 MPO/AGTR1/CETP/ABCG1 4

BP GO:0042177 negative regulation of protein catabolic process 9/227 133/18866 3.37294E-05 0.000133181 5.03081E-05 RELA/NOS2/EGFR/IL10/SNCA/PRKCG/HMGCR/PSEN1/SHH 9

BP GO:0007093 mitotic cell cycle checkpoint 10/227 166/18866 3.37725E-05 0.00013324 5.03306E-05 BAX/TGFB1/BCL2L1/CDKN1A/TP53/CDK1/BRCA1/MDM2/ATF2/EP300 10

BP GO:0006417 regulation of translation 17/227 450/18866 3.38137E-05 0.000133292 5.03501E-05 RXRA/AKT1/MAPK1/TNF/IL6/MAPK3/APP/STAT3/MTOR/EIF2S1/CD28/ERBB2/EIF2AK3/DHFR/EIF2AK4/EIF2AK2/EIF2AK1 17

BP GO:0045165 cell fate commitment 13/227 278/18866 3.39957E-05 0.000133898 5.05792E-05 BCL2/CASP3/AR/PPARG/IL6/TP53/MCL1/STAT3/MTOR/CDC42/PSEN1/SHH/TGFBR1 13

BP GO:0030593 neutrophil chemotaxis 8/227 103/18866 3.46028E-05 0.000136064 5.13973E-05 PIK3CG/DPP4/IL1B/SYK/EDN1/CCL2/CXCL8/C5AR1 8

BP GO:0048709 oligodendrocyte differentiation 8/227 103/18866 3.46028E-05 0.000136064 5.13973E-05 PPARG/NTRK2/CDK5/MTOR/ERBB2/GSTP1/SHH/TNFRSF1B 8

BP GO:0050803 regulation of synapse structure or activity 12/227 240/18866 3.57146E-05 0.00014032 5.30049E-05 IL10/TNF/SNCA/BDNF/NTRK1/NTRK2/APP/CDK5/MAPK14/CDC42/VCP/DRD2 12

BP GO:2000677 regulation of transcription regulatory region DNA binding 6/227 52/18866 3.64209E-05 0.000142977 5.40085E-05 TGFB1/DDIT3/PARP1/IFNG/PSEN1/EP300 6

BP GO:0032649 regulation of interferon-gamma production 8/227 104/18866 3.71029E-05 0.000145534 5.49744E-05 IL10/TNF/IL1B/TLR4/DDIT3/CCR2/IL2/NR1H4 8

BP GO:0038093 Fc receptor signaling pathway 12/227 241/18866 3.71929E-05 0.000145633 5.5012E-05 JUN/RELA/FOS/MAPK1/MAPK3/HSP90AA1/SYK/MAPK8/CDC42/NFKB1/MAPK10/MAPK9 12

BP GO:0038128 ERBB2 signaling pathway 5/227 32/18866 3.72814E-05 0.000145633 5.5012E-05 PRKCA/EGFR/EGF/HSP90AA1/ERBB2 5

BP GO:0051968 positive regulation of synaptic transmission, glutamatergic 5/227 32/18866 3.72814E-05 0.000145633 5.5012E-05 PTGS2/EGFR/NTRK1/CCL2/CCR2 5

BP GO:0060603 mammary gland duct morphogenesis 5/227 32/18866 3.72814E-05 0.000145633 5.5012E-05 PGR/AR/ESR1/CSF1R/VDR 5

BP GO:0098810 neurotransmitter reuptake 5/227 32/18866 3.72814E-05 0.000145633 5.5012E-05 SLC6A3/SLC6A4/GDNF/SNCA/DRD2 5

BP GO:0038095 Fc-epsilon receptor signaling pathway 10/227 169/18866 3.93489E-05 0.000153584 5.80151E-05 JUN/RELA/FOS/MAPK1/MAPK3/SYK/MAPK8/NFKB1/MAPK10/MAPK9 10

BP GO:0002449 lymphocyte mediated immunity 15/227 366/18866 3.94273E-05 0.000153763 5.80829E-05 TGFB1/IL10/TNF/IL6/IL1B/ICAM1/CRP/CCR2/CD28/IL2/IL4/CD40LG/PTPN6/C1R/TNFRSF1B 15

BP GO:0045621 positive regulation of lymphocyte differentiation 8/227 105/18866 3.97521E-05 0.000154903 5.85134E-05 IL1B/SYK/IL1A/CD80/IL2/IFNG/IL4/SHH 8

BP GO:0045727 positive regulation of translation 9/227 136/18866 4.02157E-05 0.000156581 5.91474E-05 RXRA/MAPK1/TNF/IL6/MAPK3/MTOR/CD28/ERBB2/EIF2AK4 9

BP GO:0043124 negative regulation of I-kappaB kinase/NF-kappaB signaling 6/227 53/18866 4.06612E-05 0.000158186 5.97536E-05 CASP8/STAT1/ESR1/SIRT1/GSTP1/NR1H4 6

BP GO:0016579 protein deubiquitination 13/227 283/18866 4.08679E-05 0.00015886 6.00083E-05 AR/TP53/HIF1A/ESR1/MYC/CDK1/BRCA1/BIRC3/MDM2/VCP/BIRC2/EP300/TGFBR1 13

BP GO:2000241 regulation of reproductive process 10/227 170/18866 4.13745E-05 0.000160698 6.07025E-05 RXRA/AR/VEGFA/ESR1/GJA1/INSR/GNRH1/SHH/TACR1/PDE5A 10

BP GO:0007411 axon guidance 13/227 284/18866 4.2379E-05 0.000164465 6.21256E-05 PRKCA/VEGFA/MAPK1/GDNF/BDNF/MAPK3/NTRK1/APP/CDK5/ERBB2/SHH/CSF1R/RET 13

BP GO:0014812 muscle cell migration 8/227 106/18866 4.25574E-05 0.000164602 6.21773E-05 PLAU/BCL2/PPARD/NFE2L2/PLAT/MDM2/GSTP1/PRKG1 8

BP GO:0071156 regulation of cell cycle arrest 8/227 106/18866 4.25574E-05 0.000164602 6.21773E-05 BAX/CDKN1A/TP53/CDK1/CDK5/BRCA1/MDM2/EP300 8

BP GO:0043367 CD4-positive, alpha-beta T cell differentiation 7/227 78/18866 4.25874E-05 0.000164602 6.21773E-05 IL6/STAT3/CD80/MTOR/IL2/IFNG/IL4 7

BP GO:0050688 regulation of defense response to virus 7/227 78/18866 4.25874E-05 0.000164602 6.21773E-05 STAT1/IL1B/MMP12/BIRC3/IL4/BIRC2/EIF2AK4 7

BP GO:0072332 intrinsic apoptotic signaling pathway by p53 class mediator 7/227 78/18866 4.25874E-05 0.000164602 6.21773E-05 BCL2/BAX/CDKN1A/TP53/SIRT1/MDM2/EP300 7

BP GO:0009299 mRNA transcription 4/227 17/18866 4.29559E-05 0.00016482 6.22597E-05 TP53/PPARD/STAT3/C5AR1 4

BP GO:0030540 female genitalia development 4/227 17/18866 4.29559E-05 0.00016482 6.22597E-05 BAX/ESR1/CYP19A1/BAK1 4

BP GO:0030730 sequestering of triglyceride 4/227 17/18866 4.29559E-05 0.00016482 6.22597E-05 PPARG/TNF/IL1B/PPARA 4

BP GO:0035729 cellular response to hepatocyte growth factor stimulus 4/227 17/18866 4.29559E-05 0.00016482 6.22597E-05 RELA/IL10/GCLC/CREB1 4

BP GO:0045722 positive regulation of gluconeogenesis 4/227 17/18866 4.29559E-05 0.00016482 6.22597E-05 GPT/SIRT1/FOXO1/PPARA 4

BP GO:0045986 negative regulation of smooth muscle contraction 4/227 17/18866 4.29559E-05 0.00016482 6.22597E-05 PTGS2/SOD1/KCNMA1/PRKG1 4

BP GO:0070242 thymocyte apoptotic process 4/227 17/18866 4.29559E-05 0.00016482 6.22597E-05 BAX/TP53/HIF1A/BAK1 4

BP GO:0090494 dopamine uptake 4/227 17/18866 4.29559E-05 0.00016482 6.22597E-05 SLC6A3/GDNF/SNCA/DRD2 4

BP GO:1903209 positive regulation of oxidative stress-induced cell death 4/227 17/18866 4.29559E-05 0.00016482 6.22597E-05 MMP3/SOD1/TLR4/MCL1 4

BP GO:0030856 regulation of epithelial cell differentiation 10/227 171/18866 4.34885E-05 0.00016668 6.29622E-05 VEGFA/MMP9/TNF/XDH/STAT1/IL1B/GDNF/IFNG/TNFRSF1A/VDR 10

BP GO:0048011 neurotrophin TRK receptor signaling pathway 5/227 33/18866 4.35107E-05 0.00016668 6.29622E-05 CASP3/BDNF/NGF/NTRK1/NTRK2 5

BP GO:0097485 neuron projection guidance 13/227 285/18866 4.39387E-05 0.000168184 6.35303E-05 PRKCA/VEGFA/MAPK1/GDNF/BDNF/MAPK3/NTRK1/APP/CDK5/ERBB2/SHH/CSF1R/RET 13

BP GO:0002705 positive regulation of leukocyte mediated immunity 9/227 138/18866 4.51018E-05 0.000172497 6.51596E-05 TGFB1/NOS2/TNF/IL6/IL1B/SYK/CD28/IL2/IL4 9

BP GO:0032623 interleukin-2 production 6/227 54/18866 4.52882E-05 0.000173071 6.53763E-05 IL1B/IL1A/CD80/CCR2/CD28/NR1H4 6

BP GO:0006469 negative regulation of protein kinase activity 12/227 246/18866 4.54085E-05 0.000173391 6.54973E-05 CASP3/AKT1/CDKN1A/IL1B/SNCA/SIRT1/IGF1R/IFNG/GSTP1/HMGCR/PSEN1/PTPN6 12

BP GO:0030330 DNA damage response, signal transduction by p53 class mediator 8/227 107/18866 4.5526E-05 0.0001737 6.56141E-05 BAX/CDKN1A/TP53/CDK1/SIRT1/BRCA1/MDM2/EP300 8

BP GO:0099601 regulation of neurotransmitter receptor activity 7/227 79/18866 4.62553E-05 0.000176341 6.66116E-05 OPRM1/APP/CDK5/CCL2/CCR2/IFNG/GRIA2 7

BP GO:0009259 ribonucleotide metabolic process 16/227 416/18866 4.66909E-05 0.000177859 6.71851E-05 HTR2A/TGFB1/HIF1A/APP/PARP1/STAT3/PPARA/IFNG/IL4/INSR/VCP/TPI1/HMGCR/PSEN1/EP300/PDE5A 16

BP GO:0021700 developmental maturation 13/227 287/18866 4.72088E-05 0.000179688 6.7876E-05 PGR/BCL2/PPARG/VEGFA/CDKN1A/MMP2/HIF1A/DDIT3/AKR1B1/APP/MTOR/REN/RET 13

BP GO:0032526 response to retinoic acid 8/227 108/18866 4.86654E-05 0.000185084 6.99142E-05 RXRA/SLC6A4/PPARG/GJA1/RXRB/CREB1/RXRG/RET 8

BP GO:0030100 regulation of endocytosis 11/227 210/18866 4.97126E-05 0.000188916 7.13616E-05 PPARG/VEGFA/EGF/SNCA/SYK/SELE/CDC42/NR1H3/IL4/INSR/DRD2 11

BP GO:0032655 regulation of interleukin-12 production 6/227 55/18866 5.03274E-05 0.000190899 7.21108E-05 IL10/TLR4/SYK/MAPK14/IFNG/CD40LG 6

BP GO:0035633 maintenance of blood-brain barrier 5/227 34/18866 5.05156E-05 0.000190899 7.21108E-05 PTGS2/VEGFA/IL6/GJA1/PECAM1 5

BP GO:0042311 vasodilation 5/227 34/18866 5.05156E-05 0.000190899 7.21108E-05 SOD1/SOD2/KCNMA1/GPX1/PRKG1 5

BP GO:0042759 long-chain fatty acid biosynthetic process 5/227 34/18866 5.05156E-05 0.000190899 7.21108E-05 PTGS2/GSTP1/CYP3A4/GPX4/ALOX5 5

BP GO:0043276 anoikis 5/227 34/18866 5.05156E-05 0.000190899 7.21108E-05 BCL2/AKT1/NTRK2/MCL1/MTOR 5

BP GO:0046685 response to arsenic-containing substance 5/227 34/18866 5.05156E-05 0.000190899 7.21108E-05 CDKN1A/HMOX1/ATF3/GCLC/VCP 5

BP GO:0051482 positive regulation of cytosolic calcium ion concentration involved in phospholipase C-activating G protein-coupled signaling pathway 5/227 34/18866 5.05156E-05 0.000190899 7.21108E-05 HTR2C/EDN1/AGTR1/GRM1/DRD2 5

BP GO:0018958 phenol-containing compound metabolic process 8/227 109/18866 5.19831E-05 0.000196133 7.40878E-05 SLC6A3/BCL2/SNCA/AKR1B1/TYR/CTSB/HTR1A/DRD2 8

BP GO:2000278 regulation of DNA biosynthetic process 8/227 109/18866 5.19831E-05 0.000196133 7.40878E-05 PPARG/CDKN1A/MAPK1/TP53/MYC/MAPK3/HSP90AA1/GJA1 8

BP GO:0007586 digestion 9/227 141/18866 5.33664E-05 0.000201192 7.5999E-05 CHRM3/TLR4/SI/MGAM/NR1H3/PRSS3/NPC1L1/TRPV1/VDR 9

BP GO:0030278 regulation of ossification 11/227 212/18866 5.41702E-05 0.000204061 7.70826E-05 BCL2/TGFB1/MAPK1/TNF/IL6/HIF1A/MAPK3/CDK6/MAPK14/ALOX5/CCR1 11

BP GO:0071158 positive regulation of cell cycle arrest 7/227 81/18866 5.43693E-05 0.000204649 7.73047E-05 BAX/CDKN1A/TP53/CDK1/BRCA1/MDM2/EP300 7

BP GO:0051023 regulation of immunoglobulin secretion 4/227 18/18866 5.47091E-05 0.000205116 7.74811E-05 TNF/IL6/IL2/CD40LG 4

BP GO:0090493 catecholamine uptake 4/227 18/18866 5.47091E-05 0.000205116 7.74811E-05 SLC6A3/GDNF/SNCA/DRD2 4

BP GO:0150078 positive regulation of neuroinflammatory response 4/227 18/18866 5.47091E-05 0.000205116 7.74811E-05 TNF/IL6/IL1B/MMP8 4

BP GO:1900221 regulation of amyloid-beta clearance 4/227 18/18866 5.47091E-05 0.000205116 7.74811E-05 TNF/IFNG/IL4/HMGCR 4

BP GO:1990000 amyloid fibril formation 4/227 18/18866 5.47091E-05 0.000205116 7.74811E-05 CHRNA7/APP/MDM2/PSEN1 4

BP GO:0006977 DNA damage response, signal transduction by p53 class mediator resulting in cell cycle arrest 6/227 56/18866 5.58055E-05 0.000208897 7.89093E-05 BAX/CDKN1A/TP53/CDK1/MDM2/EP300 6

BP GO:0016447 somatic recombination of immunoglobulin gene segments 6/227 56/18866 5.58055E-05 0.000208897 7.89093E-05 TGFB1/IL10/CD28/IL2/IL4/CD40LG 6

BP GO:0030324 lung development 10/227 177/18866 5.82103E-05 0.000217436 8.21348E-05 PGR/EGFR/VEGFA/MAPK1/TNF/MAPK3/CDC42/CREB1/SHH/EP300 10

BP GO:0007435 salivary gland morphogenesis 5/227 35/18866 5.8361E-05 0.000217436 8.21348E-05 TGFB1/EGFR/TNF/CDC42/SHH 5

BP GO:0032735 positive regulation of interleukin-12 production 5/227 35/18866 5.8361E-05 0.000217436 8.21348E-05 TLR4/SYK/MAPK14/IFNG/CD40LG 5

BP GO:0034390 smooth muscle cell apoptotic process 5/227 35/18866 5.8361E-05 0.000217436 8.21348E-05 PPARG/SIRT1/EDN1/SOD2/IFNG 5

BP GO:0034391 regulation of smooth muscle cell apoptotic process 5/227 35/18866 5.8361E-05 0.000217436 8.21348E-05 PPARG/SIRT1/EDN1/SOD2/IFNG 5

BP GO:0060251 regulation of glial cell proliferation 5/227 35/18866 5.8361E-05 0.000217436 8.21348E-05 TNF/IL6/IL1B/MTOR/CREB1 5

BP GO:0008286 insulin receptor signaling pathway 9/227 143/18866 5.95577E-05 0.000221547 8.36878E-05 RELA/AKT1/IL1B/SIRT1/IGF1R/FOXO1/PRKCB/INSR/NR1H4 9

BP GO:0072006 nephron development 9/227 143/18866 5.95577E-05 0.000221547 8.36878E-05 BCL2/VEGFA/STAT1/MYC/GDNF/PECAM1/CFLAR/SHH/RET 9

BP GO:0002090 regulation of receptor internalization 6/227 57/18866 6.17499E-05 0.000228807 8.64301E-05 VEGFA/EGF/SYK/SELE/INSR/DRD2 6

BP GO:0032615 interleukin-12 production 6/227 57/18866 6.17499E-05 0.000228807 8.64301E-05 IL10/TLR4/SYK/MAPK14/IFNG/CD40LG 6

BP GO:0045840 positive regulation of mitotic nuclear division 6/227 57/18866 6.17499E-05 0.000228807 8.64301E-05 EGF/IL1B/EDN1/IL1A/CD28/INSR 6

BP GO:0072431 signal transduction involved in mitotic G1 DNA damage checkpoint 6/227 57/18866 6.17499E-05 0.000228807 8.64301E-05 BAX/CDKN1A/TP53/CDK1/MDM2/EP300 6

BP GO:1902400 intracellular signal transduction involved in G1 DNA damage checkpoint 6/227 57/18866 6.17499E-05 0.000228807 8.64301E-05 BAX/CDKN1A/TP53/CDK1/MDM2/EP300 6

BP GO:0099565 chemical synaptic transmission, postsynaptic 8/227 112/18866 6.30868E-05 0.000233579 8.82326E-05 CHRNA7/OPRM1/AKT1/BDNF/APP/CDK5/TRPV1/DRD2 8

BP GO:0019693 ribose phosphate metabolic process 16/227 427/18866 6.35718E-05 0.000235191 8.88417E-05 HTR2A/TGFB1/HIF1A/APP/PARP1/STAT3/PPARA/IFNG/IL4/INSR/VCP/TPI1/HMGCR/PSEN1/EP300/PDE5A 16

BP GO:0050792 regulation of viral process 11/227 217/18866 6.68444E-05 0.000247106 9.33425E-05 BCL2/JUN/TNF/STAT1/CXCL8/CRP/CD28/CSF1R/EP300/EIF2AK4/EIF2AK2 11

BP GO:0043368 positive T cell selection 5/227 36/18866 6.71142E-05 0.000247526 9.35011E-05 BCL2/IL6/STAT3/MTOR/SHH 5

BP GO:0071402 cellular response to lipoprotein particle stimulus 5/227 36/18866 6.71142E-05 0.000247526 9.35011E-05 PPARG/AKT1/TLR4/SYK/ABCG1 5

BP GO:1905332 positive regulation of morphogenesis of an epithelium 5/227 36/18866 6.71142E-05 0.000247526 9.35011E-05 AR/VEGFA/GDNF/GJA1/MTOR 5

BP GO:0019369 arachidonic acid metabolic process 6/227 58/18866 6.81893E-05 0.000251101 9.48518E-05 PTGS2/MAPK3/GPX1/GPX4/ALOX5/PLA2G4A 6

BP GO:0031638 zymogen activation 6/227 58/18866 6.81893E-05 0.000251101 9.48518E-05 PLAU/CASP8/BAK1/PLAT/PRSS3/C1R 6

BP GO:0010744 positive regulation of macrophage derived foam cell differentiation 4/227 19/18866 6.8646E-05 0.00025142 9.49721E-05 AGTR1/NFKB1/CSF2/MAPK9 4

BP GO:0032095 regulation of response to food 4/227 19/18866 6.8646E-05 0.00025142 9.49721E-05 OPRM1/PPARA/MTOR/PRKCG 4

BP GO:0035728 response to hepatocyte growth factor 4/227 19/18866 6.8646E-05 0.00025142 9.49721E-05 RELA/IL10/GCLC/CREB1 4

BP GO:0045780 positive regulation of bone resorption 4/227 19/18866 6.8646E-05 0.00025142 9.49721E-05 PRKCA/EGFR/CA2/SYK 4

BP GO:0045947 negative regulation of translational initiation 4/227 19/18866 6.8646E-05 0.00025142 9.49721E-05 EIF2S1/EIF2AK3/EIF2AK4/EIF2AK1 4

BP GO:0046852 positive regulation of bone remodeling 4/227 19/18866 6.8646E-05 0.00025142 9.49721E-05 PRKCA/EGFR/CA2/SYK 4

BP GO:0060252 positive regulation of glial cell proliferation 4/227 19/18866 6.8646E-05 0.00025142 9.49721E-05 TNF/IL6/IL1B/MTOR 4

BP GO:0030323 respiratory tube development 10/227 181/18866 7.02228E-05 0.000256997 9.70789E-05 PGR/EGFR/VEGFA/MAPK1/TNF/MAPK3/CDC42/CREB1/SHH/EP300 10

BP GO:0044409 entry into host 9/227 147/18866 7.37691E-05 0.000269561 0.000101825 HTR2A/DPP4/EGFR/CDK1/ICAM1/CXCL8/CD80/CTSB/CCR5 9

BP GO:0055123 digestive system development 9/227 147/18866 7.37691E-05 0.000269561 0.000101825 BCL2/EGFR/CDKN1A/TNF/HIF1A/CXCL8/INSR/SHH/RET 9

BP GO:0070646 protein modification by small protein removal 13/227 300/18866 7.41106E-05 0.0002706 0.000102217 AR/TP53/HIF1A/ESR1/MYC/CDK1/BRCA1/BIRC3/MDM2/VCP/BIRC2/EP300/TGFBR1 13

BP GO:0016042 lipid catabolic process 14/227 343/18866 7.46423E-05 0.000272333 0.000102872 PIK3CG/AKT1/TNF/PPARD/IL1B/FUCA1/CYP19A1/PPARA/MTOR/CYP3A4/GM2A/ENPP2/PLA2G4A/IDH1 14

BP GO:0045454 cell redox homeostasis 6/227 59/18866 7.51535E-05 0.000273358 0.000103259 NOS2/NFE2L2/DDIT3/GCLC/MPO/GPX1 6

BP GO:0072413 signal transduction involved in mitotic cell cycle checkpoint 6/227 59/18866 7.51535E-05 0.000273358 0.000103259 BAX/CDKN1A/TP53/CDK1/MDM2/EP300 6

BP GO:1902402 signal transduction involved in mitotic DNA damage checkpoint 6/227 59/18866 7.51535E-05 0.000273358 0.000103259 BAX/CDKN1A/TP53/CDK1/MDM2/EP300 6

BP GO:1902403 signal transduction involved in mitotic DNA integrity checkpoint 6/227 59/18866 7.51535E-05 0.000273358 0.000103259 BAX/CDKN1A/TP53/CDK1/MDM2/EP300 6

BP GO:0032609 interferon-gamma production 8/227 115/18866 7.60956E-05 0.000276574 0.000104474 IL10/TNF/IL1B/TLR4/DDIT3/CCR2/IL2/NR1H4 8

BP GO:0030212 hyaluronan metabolic process 5/227 37/18866 7.68454E-05 0.000278873 0.000105342 TGFB1/AKT1/EGF/IL1B/NFKB1 5

BP GO:0045191 regulation of isotype switching 5/227 37/18866 7.68454E-05 0.000278873 0.000105342 TGFB1/IL10/CD28/IL2/IL4 5

BP GO:1903362 regulation of cellular protein catabolic process 12/227 260/18866 7.72954E-05 0.000280292 0.000105878 AKT1/EGF/NFE2L2/HSP90AA1/GCLC/MDM2/VCP/PRKCG/PSEN1/SHH/GPX1/MAPK9 12

BP GO:0007193 adenylate cyclase-inhibiting G protein-coupled receptor signaling pathway 7/227 86/18866 7.9854E-05 0.000288689 0.00010905 CHRM3/OPRM1/EDN1/GABBR1/HRH3/HTR1A/DRD2 7

BP GO:0014910 regulation of smooth muscle cell migration 7/227 86/18866 7.9854E-05 0.000288689 0.00010905 PLAU/BCL2/PPARD/NFE2L2/MDM2/GSTP1/PRKG1 7

BP GO:0045445 myoblast differentiation 7/227 86/18866 7.9854E-05 0.000288689 0.00010905 TGFB1/TNF/PPARD/DDIT3/MAPK14/HMGCR/SHH 7

BP GO:2000106 regulation of leukocyte apoptotic process 7/227 86/18866 7.9854E-05 0.000288689 0.00010905 BAX/IL10/TP53/HIF1A/SIRT1/IL2/CCR5 7

BP GO:0030518 intracellular steroid hormone receptor signaling pathway 8/227 116/18866 8.08973E-05 0.000291795 0.000110223 PGR/AR/ESR2/ESR1/PARP1/SIRT1/BRCA1/EP300 8

BP GO:0046916 cellular transition metal ion homeostasis 8/227 116/18866 8.08973E-05 0.000291795 0.000110223 SOD1/HIF1A/MYC/HMOX1/APP/ABCG2/IFNG/TMPRSS6 8

BP GO:0060964 regulation of gene silencing by miRNA 8/227 116/18866 8.08973E-05 0.000291795 0.000110223 TGFB1/PPARG/EGFR/TNF/IL6/TP53/ESR1/STAT3 8

BP GO:1903364 positive regulation of cellular protein catabolic process 9/227 149/18866 8.18817E-05 0.000295122 0.00011148 AKT1/EGF/NFE2L2/HSP90AA1/GCLC/MDM2/VCP/PSEN1/MAPK9 9

BP GO:0017157 regulation of exocytosis 11/227 222/18866 8.19817E-05 0.000295258 0.000111532 HTR2A/HMOX1/SNCA/CDK5/SYK/PRKCB/CCR2/IFNG/IL4/PRKCG/DRD2 11

BP GO:0042093 T-helper cell differentiation 6/227 60/18866 8.2673E-05 0.000297523 0.000112387 IL6/STAT3/CD80/MTOR/IL2/IL4 6

BP GO:0030900 forebrain development 15/227 391/18866 8.30795E-05 0.000298759 0.000112854 SLC6A3/BAX/CASP3/EGFR/HIF1A/NTRK2/APP/CDK5/CDK6/PSEN1/CREB1/SHH/CSF1R/PRKG1/DRD2 15

BP GO:0002827 positive regulation of T-helper 1 type immune response 4/227 20/18866 8.50003E-05 0.000304286 0.000114942 IL1B/CD80/CCR2/PLA2G4A 4

BP GO:0010042 response to manganese ion 4/227 20/18866 8.50003E-05 0.000304286 0.000114942 PTGS2/APP/EIF2S1/EIF2AK3 4

BP GO:0010875 positive regulation of cholesterol efflux 4/227 20/18866 8.50003E-05 0.000304286 0.000114942 PON1/SIRT1/NR1H3/ABCG1 4

BP GO:0060546 negative regulation of necroptotic process 4/227 20/18866 8.50003E-05 0.000304286 0.000114942 CASP8/CFLAR/BIRC3/BIRC2 4

BP GO:0062099 negative regulation of programmed necrotic cell death 4/227 20/18866 8.50003E-05 0.000304286 0.000114942 CASP8/CFLAR/BIRC3/BIRC2 4

BP GO:2001169 regulation of ATP biosynthetic process 4/227 20/18866 8.50003E-05 0.000304286 0.000114942 PARP1/STAT3/IL4/VCP 4

BP GO:0014902 myotube differentiation 8/227 117/18866 8.59477E-05 0.000307042 0.000115983 BCL2/BDNF/MAPK14/CFLAR/MTOR/IL4/SHH/GPX1 8

BP GO:0032436 positive regulation of proteasomal ubiquitin-dependent protein catabolic process 7/227 87/18866 8.59639E-05 0.000307042 0.000115983 AKT1/NFE2L2/GCLC/MDM2/VCP/PSEN1/MAPK9 7

BP GO:0060291 long-term synaptic potentiation 7/227 87/18866 8.59639E-05 0.000307042 0.000115983 CHRNA7/MAPK1/NTRK2/APP/CREB1/DRD2/EIF2AK4 7

BP GO:0010661 positive regulation of muscle cell apoptotic process 5/227 38/18866 8.76276E-05 0.000311813 0.000117785 PPARG/TP53/SOD2/IFNG/HMGCR 5

BP GO:0043029 T cell homeostasis 5/227 38/18866 8.76276E-05 0.000311813 0.000117785 BCL2/BAX/CASP3/AKT1/IL2 5

BP GO:0051930 regulation of sensory perception of pain 5/227 38/18866 8.76276E-05 0.000311813 0.000117785 OPRM1/IL10/EDN1/MTOR/GRM1 5

BP GO:0071392 cellular response to estradiol stimulus 5/227 38/18866 8.76276E-05 0.000311813 0.000117785 EGFR/IL10/ESR2/ESR1/CFLAR 5

BP GO:1905898 positive regulation of response to endoplasmic reticulum stress 5/227 38/18866 8.76276E-05 0.000311813 0.000117785 BAX/NFE2L2/DDIT3/SIRT1/BAK1 5

BP GO:0031346 positive regulation of cell projection organization 15/227 394/18866 9.04504E-05 0.000321617 0.000121488 VEGFA/NFE2L2/BDNF/NGF/NTRK1/NTRK2/CFLAR/MTOR/CDC42/IL2/PSEN1/ENPP2/EP300/TGFBR1/RET 15

BP GO:0008643 carbohydrate transport 9/227 151/18866 9.0731E-05 0.000322065 0.000121658 AKT1/TNF/PPARD/IL1B/NFE2L2/MAPK14/EDN1/PRKCB/INSR 9

BP GO:0050871 positive regulation of B cell activation 9/227 151/18866 9.0731E-05 0.000322065 0.000121658 BCL2/TGFB1/CDKN1A/IL6/TLR4/SYK/CD28/IL2/IL4 9

BP GO:0002381 immunoglobulin production involved in immunoglobulin mediated immune response 6/227 61/18866 9.07797E-05 0.000322065 0.000121658 TGFB1/IL10/CD28/IL2/IL4/CD40LG 6

BP GO:0043409 negative regulation of MAPK cascade 10/227 187/18866 9.21615E-05 0.000326723 0.000123417 AKT1/MYC/IL1B/TLR4/ATF3/IGF1R/FOXO1/GSTP1/HMGCR/PTPN6 10

BP GO:1903050 regulation of proteolysis involved in cellular protein catabolic process 11/227 225/18866 9.24027E-05 0.000327334 0.000123648 AKT1/EGF/NFE2L2/GCLC/MDM2/VCP/PRKCG/PSEN1/SHH/GPX1/MAPK9 11

BP GO:1903169 regulation of calcium ion transmembrane transport 9/227 152/18866 9.54483E-05 0.000337871 0.000127628 PIK3CG/BAX/SNCA/CDK5/F2/PSEN2/PTPN6/BDKRB1/DRD2 9

BP GO:0001892 embryonic placenta development 7/227 89/18866 9.93282E-05 0.000350775 0.000132503 CASP8/EGFR/AKT1/MAPK1/IL10/HIF1A/CSF2 7

BP GO:0072331 signal transduction by p53 class mediator 12/227 267/18866 9.9459E-05 0.000350775 0.000132503 BCL2/BAX/AKT1/CDKN1A/TP53/CDK1/CDK5/MAPK14/SIRT1/BRCA1/MDM2/EP300 12

BP GO:0007405 neuroblast proliferation 6/227 62/18866 9.95065E-05 0.000350775 0.000132503 VEGFA/TP53/HIF1A/BDNF/SHH/DRD2 6

BP GO:0007431 salivary gland development 5/227 39/18866 9.95362E-05 0.000350775 0.000132503 TGFB1/EGFR/TNF/CDC42/SHH 5

BP GO:0038179 neurotrophin signaling pathway 5/227 39/18866 9.95362E-05 0.000350775 0.000132503 CASP3/BDNF/NGF/NTRK1/NTRK2 5

BP GO:0051931 regulation of sensory perception 5/227 39/18866 9.95362E-05 0.000350775 0.000132503 OPRM1/IL10/EDN1/MTOR/GRM1 5

BP GO:0060147 regulation of posttranscriptional gene silencing 8/227 120/18866 0.000102692 0.000361361 0.000136501 TGFB1/PPARG/EGFR/TNF/IL6/TP53/ESR1/STAT3 8

BP GO:0060966 regulation of gene silencing by RNA 8/227 120/18866 0.000102692 0.000361361 0.000136501 TGFB1/PPARG/EGFR/TNF/IL6/TP53/ESR1/STAT3 8

BP GO:0033673 negative regulation of kinase activity 12/227 268/18866 0.000103033 0.000362292 0.000136853 CASP3/AKT1/CDKN1A/IL1B/SNCA/SIRT1/IGF1R/IFNG/GSTP1/HMGCR/PSEN1/PTPN6 12

BP GO:0002363 alpha-beta T cell lineage commitment 4/227 21/18866 0.000104013 0.000363854 0.000137443 BCL2/IL6/STAT3/MTOR 4

BP GO:0010893 positive regulation of steroid biosynthetic process 4/227 21/18866 0.000104013 0.000363854 0.000137443 TNF/IL1B/IFNG/ABCG1 4

BP GO:0014821 phasic smooth muscle contraction 4/227 21/18866 0.000104013 0.000363854 0.000137443 GDNF/EDN1/DRD2/HTR2B 4

BP GO:0030220 platelet formation 4/227 21/18866 0.000104013 0.000363854 0.000137443 CASP9/CASP3/PTPN6/EP300 4

BP GO:0048305 immunoglobulin secretion 4/227 21/18866 0.000104013 0.000363854 0.000137443 TNF/IL6/IL2/CD40LG 4

BP GO:0061042 vascular wound healing 4/227 21/18866 0.000104013 0.000363854 0.000137443 VEGFA/TNF/KDR/ALOX5 4

BP GO:0072111 cell proliferation involved in kidney development 4/227 21/18866 0.000104013 0.000363854 0.000137443 STAT1/MYC/CFLAR/SHH 4

BP GO:0006836 neurotransmitter transport 11/227 229/18866 0.000108044 0.000377677 0.000142665 SLC6A3/HTR2A/SLC6A4/GDNF/SNCA/CDK5/PRKCB/PRKCG/PSEN1/HRH3/DRD2 11

BP GO:0120254 olefinic compound metabolic process 8/227 121/18866 0.00010884 0.000379231 0.000143252 PTGS2/MAPK3/CYP19A1/GSTP1/GPX1/GPX4/ALOX5/PLA2G4A 8

BP GO:0010830 regulation of myotube differentiation 6/227 63/18866 0.000108887 0.000379231 0.000143252 BCL2/BDNF/MAPK14/CFLAR/MTOR/IL4 6

BP GO:0031571 mitotic G1 DNA damage checkpoint 6/227 63/18866 0.000108887 0.000379231 0.000143252 BAX/CDKN1A/TP53/CDK1/MDM2/EP300 6

BP GO:0046622 positive regulation of organ growth 6/227 63/18866 0.000108887 0.000379231 0.000143252 AKT1/MAPK1/CDK1/MAPK14/EDN1/MTOR 6

BP GO:0046635 positive regulation of alpha-beta T cell activation 6/227 63/18866 0.000108887 0.000379231 0.000143252 SYK/CD80/CCR2/CD28/IFNG/SHH 6

BP GO:0002714 positive regulation of B cell mediated immunity 5/227 40/18866 0.000112649 0.000390616 0.000147552 TGFB1/TNF/CD28/IL2/IL4 5

BP GO:0002891 positive regulation of immunoglobulin mediated immune response 5/227 40/18866 0.000112649 0.000390616 0.000147552 TGFB1/TNF/CD28/IL2/IL4 5

BP GO:0010613 positive regulation of cardiac muscle hypertrophy 5/227 40/18866 0.000112649 0.000390616 0.000147552 PRKCA/PARP1/EDN1/MTOR/PDE5A 5

BP GO:0045740 positive regulation of DNA replication 5/227 40/18866 0.000112649 0.000390616 0.000147552 JUN/EGFR/EGF/CDK1/CDC42 5

BP GO:0050691 regulation of defense response to virus by host 5/227 40/18866 0.000112649 0.000390616 0.000147552 STAT1/IL1B/MMP12/IL4/EIF2AK4 5

BP GO:1902742 apoptotic process involved in development 5/227 40/18866 0.000112649 0.000390616 0.000147552 BAX/BAK1/TNFRSF1A/TNFRSF1B/VDR 5

BP GO:0001656 metanephros development 7/227 91/18866 0.000114336 0.0003956 0.000149435 BCL2/STAT1/MYC/GDNF/AKR1B1/SHH/RET 7

BP GO:0006094 gluconeogenesis 7/227 91/18866 0.000114336 0.0003956 0.000149435 GPT/ATF3/SIRT1/FOXO1/PPARA/TPI1/EP300 7

BP GO:0051899 membrane depolarization 7/227 91/18866 0.000114336 0.0003956 0.000149435 BCL2/JUN/KDR/GCLC/PARP1/EDN1/SCN9A 7

BP GO:0051928 positive regulation of calcium ion transport 8/227 122/18866 0.000115288 0.000398604 0.00015057 BAX/SNCA/CDK5/F2/BAK1/CCL2/CCR1/BDKRB1 8

BP GO:0048592 eye morphogenesis 9/227 156/18866 0.000116428 0.000402254 0.000151948 BCL2/BAX/VEGFA/HIF1A/BDNF/NTRK2/FASLG/STAT3/BAK1 9

BP GO:0043903 regulation of symbiotic process 11/227 231/18866 0.000116677 0.000402819 0.000152162 BCL2/JUN/TNF/STAT1/CXCL8/CRP/CD28/CSF1R/EP300/EIF2AK4/EIF2AK2 11

BP GO:0002437 inflammatory response to antigenic stimulus 6/227 64/18866 0.000118957 0.000408909 0.000154462 OPRM1/IL10/TNF/ICAM1/CD28/GPX1 6

BP GO:0044783 G1 DNA damage checkpoint 6/227 64/18866 0.000118957 0.000408909 0.000154462 BAX/CDKN1A/TP53/CDK1/MDM2/EP300 6

BP GO:0044819 mitotic G1/S transition checkpoint 6/227 64/18866 0.000118957 0.000408909 0.000154462 BAX/CDKN1A/TP53/CDK1/MDM2/EP300 6

BP GO:0045453 bone resorption 6/227 64/18866 0.000118957 0.000408909 0.000154462 PRKCA/EGFR/IL6/CA2/SYK/CSF1R 6

BP GO:0046637 regulation of alpha-beta T cell differentiation 6/227 64/18866 0.000118957 0.000408909 0.000154462 SYK/CD80/IL2/IFNG/IL4/SHH 6

BP GO:1900449 regulation of glutamate receptor signaling pathway 6/227 64/18866 0.000118957 0.000408909 0.000154462 OPRM1/APP/CCL2/CCR2/IFNG/GRIA2 6

BP GO:0061136 regulation of proteasomal protein catabolic process 10/227 193/18866 0.000119655 0.000411013 0.000155257 AKT1/NFE2L2/GCLC/MDM2/VCP/PRKCG/PSEN1/SHH/GPX1/MAPK9 10

BP GO:0007178 transmembrane receptor protein serine/threonine kinase signaling pathway 14/227 359/18866 0.000120802 0.000414651 0.000156631 JUN/TGFB1/FOS/TP53/MAPK3/PARP1/MAPK14/SIRT1/XIAP/CREB1/SHH/EP300/TGFBR1/TMPRSS6 14

BP GO:1904375 regulation of protein localization to cell periphery 8/227 123/18866 0.00012205 0.000418632 0.000158135 TGFB1/AR/EGFR/AKT1/BCL2L1/TNF/CDK5/IFNG 8

BP GO:0106027 neuron projection organization 7/227 92/18866 0.000122503 0.000419581 0.000158494 CHRNA7/APP/CDK5/IGF1R/CDC42/INSR/PSEN1 7

BP GO:1903321 negative regulation of protein modification by small protein conjugation or removal 7/227 92/18866 0.000122503 0.000419581 0.000158494 RELA/AKT1/GCLC/CDK5/MTOR/PRKCG/PSEN1 7

BP GO:0001818 negative regulation of cytokine production 14/227 360/18866 0.00012437 0.000425668 0.000160793 CHRNA7/TGFB1/IL10/TNF/HMOX1/TLR4/DDIT3/MMP8/F2/NFKB1/IFNG/GSTP1/PTPN6/NR1H4 14

BP GO:0006925 inflammatory cell apoptotic process 4/227 22/18866 0.000125932 0.000429778 0.000162346 IL6/FASLG/SIRT1/CCR5 4

BP GO:0036344 platelet morphogenesis 4/227 22/18866 0.000125932 0.000429778 0.000162346 CASP9/CASP3/PTPN6/EP300 4

BP GO:0051900 regulation of mitochondrial depolarization 4/227 22/18866 0.000125932 0.000429778 0.000162346 BCL2/KDR/GCLC/PARP1 4

BP GO:0055093 response to hyperoxia 4/227 22/18866 0.000125932 0.000429778 0.000162346 PPARG/CDKN1A/CAT/FOXO1 4

BP GO:0014742 positive regulation of muscle hypertrophy 5/227 41/18866 0.000127048 0.000432653 0.000163432 PRKCA/PARP1/EDN1/MTOR/PDE5A 5

BP GO:0030890 positive regulation of B cell proliferation 5/227 41/18866 0.000127048 0.000432653 0.000163432 BCL2/CDKN1A/TLR4/IL2/IL4 5

BP GO:0033574 response to testosterone 5/227 41/18866 0.000127048 0.000432653 0.000163432 AR/SIRT1/EDN1/CFLAR/GNRH1 5

BP GO:0002698 negative regulation of immune effector process 8/227 124/18866 0.000129135 0.000439448 0.000165998 TGFB1/IL10/TNF/HMOX1/CCR2/IL2/IL4/PTPN6 8

BP GO:0051702 interaction with symbiont 7/227 93/18866 0.000131137 0.000445941 0.000168451 JUN/F2/CRP/GPX1/CSF1R/EP300/EIF2AK4 7

BP GO:0055067 monovalent inorganic cation homeostasis 9/227 159/18866 0.000134585 0.000457011 0.000172633 BCL2/MAPK1/MAPK3/CA2/FASLG/EDN1/AGTR1/KCNMA1/DRD2 9

BP GO:2000058 regulation of ubiquitin-dependent protein catabolic process 9/227 159/18866 0.000134585 0.000457011 0.000172633 AKT1/EGF/NFE2L2/GCLC/MDM2/VCP/PSEN1/SHH/MAPK9 9

BP GO:0002040 sprouting angiogenesis 10/227 196/18866 0.000135818 0.000460542 0.000173967 PTGS2/AKT1/VEGFA/IL10/KDR/HMOX1/JAK1/AGTR1/CDC42/ALOX5 10

BP GO:0071897 DNA biosynthetic process 10/227 196/18866 0.000135818 0.000460542 0.000173967 PPARG/CDKN1A/MAPK1/TP53/MYC/MAPK3/HSP90AA1/SIRT1/GJA1/VCP 10

BP GO:0002562 somatic diversification of immune receptors via germline recombination within a single locus 6/227 66/18866 0.000141308 0.000478135 0.000180612 TGFB1/IL10/CD28/IL2/IL4/CD40LG 6

BP GO:0016444 somatic cell DNA recombination 6/227 66/18866 0.000141308 0.000478135 0.000180612 TGFB1/IL10/CD28/IL2/IL4/CD40LG 6

BP GO:0016445 somatic diversification of immunoglobulins 6/227 66/18866 0.000141308 0.000478135 0.000180612 TGFB1/IL10/CD28/IL2/IL4/CD40LG 6

BP GO:0001504 neurotransmitter uptake 5/227 42/18866 0.000142814 0.000481859 0.000182019 SLC6A3/SLC6A4/GDNF/SNCA/DRD2 5

BP GO:0071470 cellular response to osmotic stress 5/227 42/18866 0.000142814 0.000481859 0.000182019 PTGS2/CASP3/AKR1B1/TRPV4/MAPK10 5

BP GO:1900371 regulation of purine nucleotide biosynthetic process 5/227 42/18866 0.000142814 0.000481859 0.000182019 NOS2/PARP1/STAT3/IL4/VCP 5

BP GO:1904037 positive regulation of epithelial cell apoptotic process 5/227 42/18866 0.000142814 0.000481859 0.000182019 IL6/FASLG/CCL2/EIF2S1/CD40LG 5

BP GO:0048588 developmental cell growth 11/227 237/18866 0.000146192 0.000492906 0.000186192 MAP2/VEGFA/BDNF/NGF/HSP90AA1/APP/CDK5/EDN1/PPARA/MTOR/EIF2AK4 11

BP GO:0007626 locomotory behavior 10/227 198/18866 0.000147588 0.000497258 0.000187836 SLC6A3/OPRM1/DPP4/SOD1/GDNF/APP/HTR2C/MTOR/GRM1/DRD2 10

BP GO:1903725 regulation of phospholipid metabolic process 7/227 95/18866 0.000149887 0.000504649 0.000190628 HTR2A/TGFB1/HTR2C/CDC42/NR1H4/IDH1/HTR2B 7

BP GO:0002052 positive regulation of neuroblast proliferation 4/227 23/18866 0.000151012 0.00050664 0.00019138 VEGFA/HIF1A/SHH/DRD2 4

BP GO:0031281 positive regulation of cyclase activity 4/227 23/18866 0.000151012 0.00050664 0.00019138 NOS2/MAPK3/MAPK14/MAPK8 4

BP GO:0048714 positive regulation of oligodendrocyte differentiation 4/227 23/18866 0.000151012 0.00050664 0.00019138 PPARG/MTOR/SHH/TNFRSF1B 4

BP GO:0060547 negative regulation of necrotic cell death 4/227 23/18866 0.000151012 0.00050664 0.00019138 CASP8/CFLAR/BIRC3/BIRC2 4

BP GO:0072215 regulation of metanephros development 4/227 23/18866 0.000151012 0.00050664 0.00019138 STAT1/MYC/GDNF/RET 4

BP GO:1903052 positive regulation of proteolysis involved in cellular protein catabolic process 8/227 127/18866 0.000152458 0.000511133 0.000193077 AKT1/EGF/NFE2L2/GCLC/MDM2/VCP/PSEN1/MAPK9 8

BP GO:0032890 regulation of organic acid transport 6/227 67/18866 0.000153665 0.000514816 0.000194468 AKT1/IL1B/SYK/EDN1/PSEN1/HRH3 6

BP GO:1903305 regulation of regulated secretory pathway 9/227 162/18866 0.000155049 0.000519088 0.000196082 HTR2A/HMOX1/CDK5/SYK/PRKCB/CCR2/IL4/PRKCG/DRD2 9

BP GO:0002369 T cell cytokine production 5/227 43/18866 0.000160035 0.000534274 0.000201818 IL6/IL1B/CCR2/IL4/TNFRSF1B 5

BP GO:0030808 regulation of nucleotide biosynthetic process 5/227 43/18866 0.000160035 0.000534274 0.000201818 NOS2/PARP1/STAT3/IL4/VCP 5

BP GO:0071364 cellular response to epidermal growth factor stimulus 5/227 43/18866 0.000160035 0.000534274 0.000201818 EGFR/AKT1/CFLAR/ERBB2/GSTP1 5

BP GO:2000648 positive regulation of stem cell proliferation 5/227 43/18866 0.000160035 0.000534274 0.000201818 VEGFA/HIF1A/GJA1/SHH/DRD2 5

BP GO:1901888 regulation of cell junction assembly 10/227 200/18866 0.000160206 0.000534469 0.000201892 VEGFA/TNF/KDR/IL1B/SNCA/BDNF/NTRK1/NTRK2/APP/GJA1 10

BP GO:0002221 pattern recognition receptor signaling pathway 10/227 201/18866 0.000166848 0.000554728 0.000209544 CASP8/RELA/ESR1/TLR4/XIAP/BIRC3/NR1H3/CTSB/NR1H4/BIRC2 10

BP GO:0002753 cytoplasmic pattern recognition receptor signaling pathway 6/227 68/18866 0.000166862 0.000554728 0.000209544 CASP8/RELA/TLR4/XIAP/BIRC3/BIRC2 6

BP GO:0016239 positive regulation of macroautophagy 6/227 68/18866 0.000166862 0.000554728 0.000209544 HIF1A/KDR/HMOX1/MAPK3/SIRT1/IL4 6

BP GO:0050766 positive regulation of phagocytosis 6/227 68/18866 0.000166862 0.000554728 0.000209544 PPARG/TNF/SOD1/IL1B/CCL2/IFNG 6

BP GO:0071479 cellular response to ionizing radiation 6/227 68/18866 0.000166862 0.000554728 0.000209544 BCL2L1/CDKN1A/TP53/MAPK14/SIRT1/MDM2 6

BP GO:0034101 erythrocyte homeostasis 8/227 129/18866 0.000169855 0.000564284 0.000213154 CASP3/VEGFA/HIF1A/STAT1/HMOX1/CDK6/MAPK14/STAT3 8

BP GO:0001764 neuron migration 9/227 164/18866 0.000170086 0.000564657 0.000213295 BAX/VEGFA/NTRK2/CDK5/STAT3/GNRH1/PSEN1/PRKG1/DRD2 9

BP GO:0035710 CD4-positive, alpha-beta T cell activation 7/227 97/18866 0.000170753 0.000566476 0.000213982 IL6/STAT3/CD80/MTOR/IL2/IFNG/IL4 7

BP GO:0009746 response to hexose 10/227 202/18866 0.000173721 0.000575921 0.00021755 PTGS2/CASP3/HIF1A/PPARD/GCLC/IGF1R/GJA1/ICAM1/HMGCR/NR1H4 10

BP GO:0031670 cellular response to nutrient 5/227 44/18866 0.000178798 0.000591816 0.000223554 PPARG/HMOX1/MDM2/NR1H4/VDR 5

BP GO:0032965 regulation of collagen biosynthetic process 5/227 44/18866 0.000178798 0.000591816 0.000223554 TGFB1/PPARG/IL6/PPARD/F2 5

BP GO:0002407 dendritic cell chemotaxis 4/227 24/18866 0.000179512 0.000591816 0.000223554 PIK3CG/CCR2/CCR1/CCR5 4

BP GO:0002719 negative regulation of cytokine production involved in immune response 4/227 24/18866 0.000179512 0.000591816 0.000223554 TGFB1/IL10/TNF/HMOX1 4

BP GO:0044346 fibroblast apoptotic process 4/227 24/18866 0.000179512 0.000591816 0.000223554 PIK3CG/TP53/MYC/BAK1 4

BP GO:0050995 negative regulation of lipid catabolic process 4/227 24/18866 0.000179512 0.000591816 0.000223554 PIK3CG/AKT1/TNF/IL1B 4

BP GO:0051882 mitochondrial depolarization 4/227 24/18866 0.000179512 0.000591816 0.000223554 BCL2/KDR/GCLC/PARP1 4

BP GO:0060444 branching involved in mammary gland duct morphogenesis 4/227 24/18866 0.000179512 0.000591816 0.000223554 PGR/AR/ESR1/VDR 4

BP GO:0060038 cardiac muscle cell proliferation 6/227 69/18866 0.000180938 0.000596105 0.000225175 RXRA/MAPK1/CDK1/MAPK14/GJA1/TGFBR1 6

BP GO:0016570 histone modification 16/227 468/18866 0.000183089 0.000602773 0.000227693 PRKCA/VEGFA/TP53/IL1B/SNCA/MAPK3/CDK1/CDK5/SIRT1/MAPK8/BRCA1/PRKCB/ATF2/NR1H4/EP300/PADI4 16

BP GO:0060541 respiratory system development 10/227 204/18866 0.000188184 0.00061912 0.000233868 PGR/EGFR/VEGFA/MAPK1/TNF/MAPK3/CDC42/CREB1/SHH/EP300 10

BP GO:0032434 regulation of proteasomal ubiquitin-dependent protein catabolic process 8/227 131/18866 0.000188853 0.00062089 0.000234537 AKT1/NFE2L2/GCLC/MDM2/VCP/PSEN1/SHH/MAPK9 8

BP GO:0140014 mitotic nuclear division 12/227 286/18866 0.000189056 0.000621128 0.000234627 PRKCA/EGF/IL1B/CDK1/EDN1/PRKCB/BIRC5/IL1A/XIAP/CDC42/CD28/INSR 12

BP GO:0010749 regulation of nitric oxide mediated signal transduction 3/227 10/18866 0.00019381 0.000630214 0.000238059 EGFR/VEGFA/PDE5A 3

BP GO:0014041 regulation of neuron maturation 3/227 10/18866 0.00019381 0.000630214 0.000238059 BCL2/MTOR/RET 3

BP GO:0021936 regulation of cerebellar granule cell precursor proliferation 3/227 10/18866 0.00019381 0.000630214 0.000238059 SLC6A4/EGF/SHH 3

BP GO:0032025 response to cobalt ion 3/227 10/18866 0.00019381 0.000630214 0.000238059 CASP9/CASP3/CASP8 3

BP GO:0034350 regulation of glial cell apoptotic process 3/227 10/18866 0.00019381 0.000630214 0.000238059 PRKCA/CDK5/CCL2 3

BP GO:0044557 relaxation of smooth muscle 3/227 10/18866 0.00019381 0.000630214 0.000238059 SOD1/KCNMA1/PRKG1 3

BP GO:0048304 positive regulation of isotype switching to IgG isotypes 3/227 10/18866 0.00019381 0.000630214 0.000238059 CD28/IL2/IL4 3

BP GO:0060068 vagina development 3/227 10/18866 0.00019381 0.000630214 0.000238059 BAX/ESR1/BAK1 3

BP GO:0070391 response to lipoteichoic acid 3/227 10/18866 0.00019381 0.000630214 0.000238059 RELA/TLR4/MAPK14 3

BP GO:0071104 response to interleukin-9 3/227 10/18866 0.00019381 0.000630214 0.000238059 STAT1/STAT3/JAK1 3

BP GO:0071223 cellular response to lipoteichoic acid 3/227 10/18866 0.00019381 0.000630214 0.000238059 RELA/TLR4/MAPK14 3

BP GO:0072203 cell proliferation involved in metanephros development 3/227 10/18866 0.00019381 0.000630214 0.000238059 STAT1/MYC/SHH 3

BP GO:1900222 negative regulation of amyloid-beta clearance 3/227 10/18866 0.00019381 0.000630214 0.000238059 TNF/IFNG/HMGCR 3

BP GO:1903431 positive regulation of cell maturation 3/227 10/18866 0.00019381 0.000630214 0.000238059 BCL2/MTOR/RET 3

BP GO:1990535 neuron projection maintenance 3/227 10/18866 0.00019381 0.000630214 0.000238059 APP/INSR/PSEN1 3

BP GO:0060047 heart contraction 12/227 287/18866 0.000195246 0.000634449 0.000239658 PIK3CG/SOD1/EDN1/GJA1/MTOR/CDC42/MDM2/IL2/GPX1/TRPV1/DRD2/PDE5A 12

BP GO:0042476 odontogenesis 8/227 132/18866 0.000198987 0.000644197 0.000243341 BAX/CA2/EDN1/FOXO1/PPARA/PAM/ATF2/SHH 8

BP GO:0001974 blood vessel remodeling 5/227 45/18866 0.000199194 0.000644197 0.000243341 BAX/TGFB1/BAK1/CCR2/MDM2 5

BP GO:0002639 positive regulation of immunoglobulin production 5/227 45/18866 0.000199194 0.000644197 0.000243341 TGFB1/IL6/CD28/IL2/IL4 5

BP GO:0014047 glutamate secretion 5/227 45/18866 0.000199194 0.000644197 0.000243341 BDNF/NTRK2/GJA1/TRPV1/HRH3 5

BP GO:0042771 intrinsic apoptotic signaling pathway in response to DNA damage by p53 class mediator 5/227 45/18866 0.000199194 0.000644197 0.000243341 BCL2/CDKN1A/TP53/SIRT1/EP300 5

BP GO:0045911 positive regulation of DNA recombination 5/227 45/18866 0.000199194 0.000644197 0.000243341 TGFB1/PARP1/CD28/IL2/IL4 5

BP GO:0090311 regulation of protein deacetylation 5/227 45/18866 0.000199194 0.000644197 0.000243341 VEGFA/TP53/MAPK8/IFNG/EP300 5

BP GO:0002702 positive regulation of production of molecular mediator of immune response 7/227 100/18866 0.000206401 0.00066705 0.000251973 TGFB1/IL6/IL1B/TLR4/CD28/IL2/IL4 7

BP GO:0002053 positive regulation of mesenchymal cell proliferation 4/227 25/18866 0.000211698 0.000680649 0.00025711 VEGFA/STAT1/MYC/SHH 4

BP GO:0032104 regulation of response to extracellular stimulus 4/227 25/18866 0.000211698 0.000680649 0.00025711 OPRM1/PPARA/MTOR/PRKCG 4

BP GO:0032107 regulation of response to nutrient levels 4/227 25/18866 0.000211698 0.000680649 0.00025711 OPRM1/PPARA/MTOR/PRKCG 4

BP GO:0045662 negative regulation of myoblast differentiation 4/227 25/18866 0.000211698 0.000680649 0.00025711 TGFB1/TNF/PPARD/DDIT3 4

BP GO:0060571 morphogenesis of an epithelial fold 4/227 25/18866 0.000211698 0.000680649 0.00025711 AR/EGFR/HIF1A/SHH 4

BP GO:0071677 positive regulation of mononuclear cell migration 4/227 25/18866 0.000211698 0.000680649 0.00025711 TNF/CCR2/IL4/CCR1 4

BP GO:2000679 positive regulation of transcription regulatory region DNA binding 4/227 25/18866 0.000211698 0.000680649 0.00025711 TGFB1/PARP1/IFNG/EP300 4

BP GO:0015800 acidic amino acid transport 6/227 71/18866 0.000211897 0.000680649 0.00025711 BDNF/NTRK2/GJA1/PSEN1/TRPV1/HRH3 6

BP GO:0051785 positive regulation of nuclear division 6/227 71/18866 0.000211897 0.000680649 0.00025711 EGF/IL1B/EDN1/IL1A/CD28/INSR 6

BP GO:0052126 movement in host environment 9/227 169/18866 0.000213073 0.000683964 0.000258363 HTR2A/DPP4/EGFR/CDK1/ICAM1/CXCL8/CD80/CTSB/CCR5 9

BP GO:0010976 positive regulation of neuron projection development 12/227 290/18866 0.000214864 0.000689246 0.000260358 VEGFA/NFE2L2/BDNF/NGF/NTRK1/NTRK2/CFLAR/MTOR/IL2/PSEN1/EP300/RET 12

BP GO:0007215 glutamate receptor signaling pathway 7/227 101/18866 0.000219536 0.000702336 0.000265303 OPRM1/APP/CCL2/CCR2/IFNG/GRM1/GRIA2 7

BP GO:0010522 regulation of calcium ion transport into cytosol 7/227 101/18866 0.000219536 0.000702336 0.000265303 BCL2/BAX/SNCA/F2/BAK1/PTPN6/BDKRB1 7

BP GO:0042632 cholesterol homeostasis 7/227 101/18866 0.000219536 0.000702336 0.000265303 SIRT1/NR1H3/NR1I2/NR1H4/CETP/ABCG1/VDR 7

BP GO:0044773 mitotic DNA damage checkpoint 7/227 101/18866 0.000219536 0.000702336 0.000265303 BAX/CDKN1A/TP53/CDK1/MDM2/ATF2/EP300 7

BP GO:0045995 regulation of embryonic development 8/227 134/18866 0.000220597 0.000704784 0.000266227 AR/IL10/NFE2L2/GDNF/CDK1/GJA1/INSR/SHH 8

BP GO:0046718 viral entry into host cell 8/227 134/18866 0.000220597 0.000704784 0.000266227 HTR2A/DPP4/EGFR/CDK1/ICAM1/CD80/CTSB/CCR5 8

BP GO:0051937 catecholamine transport 6/227 72/18866 0.000228866 0.000730712 0.000276021 SLC6A3/HTR2A/GDNF/SNCA/HRH3/DRD2 6

BP GO:0048565 digestive tract development 8/227 135/18866 0.000232103 0.000740548 0.000279737 BCL2/EGFR/CDKN1A/TNF/HIF1A/CXCL8/SHH/RET 8

BP GO:0055092 sterol homeostasis 7/227 102/18866 0.000233336 0.000743983 0.000281034 SIRT1/NR1H3/NR1I2/NR1H4/CETP/ABCG1/VDR 7

BP GO:0001701 in utero embryonic development 14/227 383/18866 0.000235823 0.00075141 0.00028384 RXRA/CASP8/AR/EGFR/AKT1/VEGFA/BCL2L1/MAPK1/IL10/TP53/HIF1A/EDN1/CSF2/TGFBR1 14

BP GO:0022602 ovulation cycle process 5/227 47/18866 0.000245259 0.000780429 0.000294801 PGR/CASP3/ESR1/SIRT1/PAM 5

BP GO:0032369 negative regulation of lipid transport 5/227 47/18866 0.000245259 0.000780429 0.000294801 AKT1/EGF/NR1H3/NFKB1/SHH 5

BP GO:0060079 excitatory postsynaptic potential 7/227 103/18866 0.000247827 0.000783399 0.000295923 CHRNA7/OPRM1/AKT1/APP/CDK5/TRPV1/DRD2 7

BP GO:1901570 fatty acid derivative biosynthetic process 7/227 103/18866 0.000247827 0.000783399 0.000295923 PTGS2/IL1B/SYK/SIRT1/EDN1/ALOX5/PLA2G4A 7

BP GO:0001783 B cell apoptotic process 4/227 26/18866 0.00024784 0.000783399 0.000295923 BAX/IL10/BAK1/IL2 4

BP GO:0030810 positive regulation of nucleotide biosynthetic process 4/227 26/18866 0.00024784 0.000783399 0.000295923 NOS2/STAT3/IL4/VCP 4

BP GO:0035902 response to immobilization stress 4/227 26/18866 0.00024784 0.000783399 0.000295923 PPARG/FOS/MDM2/REN 4

BP GO:0060740 prostate gland epithelium morphogenesis 4/227 26/18866 0.00024784 0.000783399 0.000295923 RXRA/AR/ESR1/SHH 4

BP GO:0070633 transepithelial transport 4/227 26/18866 0.00024784 0.000783399 0.000295923 ABCG2/ABCB1/EDN1/CSF2 4

BP GO:1900373 positive regulation of purine nucleotide biosynthetic process 4/227 26/18866 0.00024784 0.000783399 0.000295923 NOS2/STAT3/IL4/VCP 4

BP GO:1900739 regulation of protein insertion into mitochondrial membrane involved in apoptotic signaling pathway 4/227 26/18866 0.00024784 0.000783399 0.000295923 BCL2/CASP8/TP53/MAPK8 4

BP GO:1900740 positive regulation of protein insertion into mitochondrial membrane involved in apoptotic signaling pathway 4/227 26/18866 0.00024784 0.000783399 0.000295923 BCL2/CASP8/TP53/MAPK8 4

BP GO:0071560 cellular response to transforming growth factor beta stimulus 11/227 252/18866 0.000248983 0.000786276 0.00029701 JUN/TGFB1/FOS/TP53/PARP1/SIRT1/EDN1/CFLAR/CREB1/EP300/TGFBR1 11

BP GO:0016569 covalent chromatin modification 16/227 481/18866 0.000249081 0.000786276 0.00029701 PRKCA/VEGFA/TP53/IL1B/SNCA/MAPK3/CDK1/CDK5/SIRT1/MAPK8/BRCA1/PRKCB/ATF2/NR1H4/EP300/PADI4 16

BP GO:0006937 regulation of muscle contraction 9/227 173/18866 0.000253646 0.000800155 0.000302253 PTGS2/CHRM3/PIK3CG/SOD1/EDN1/KCNMA1/PRKG1/TACR1/PDE5A 9

BP GO:0046486 glycerolipid metabolic process 15/227 434/18866 0.000259127 0.000816535 0.00030844 HTR2A/PIK3CG/PON1/ACHE/CAT/HTR2C/SIRT1/NR1H3/ENPP2/NR1H4/GPX1/PLA2G4A/CETP/CSF1R/HTR2B 15

BP GO:0031647 regulation of protein stability 12/227 296/18866 0.000259182 0.000816535 0.00030844 BCL2/CASP3/CDKN1A/MAPK1/TP53/SNCA/HSP90AA1/SIRT1/CDK7/MDM2/CREB1/EP300 12

BP GO:0031652 positive regulation of heat generation 3/227 11/18866 0.000264125 0.000826629 0.000312253 PTGS2/TNF/IL1B 3

BP GO:0033483 gas homeostasis 3/227 11/18866 0.000264125 0.000826629 0.000312253 HIF1A/SOD2/GSTP1 3

BP GO:0043619 regulation of transcription from RNA polymerase II promoter in response to oxidative stress 3/227 11/18866 0.000264125 0.000826629 0.000312253 HIF1A/HMOX1/NFE2L2 3

BP GO:0045899 positive regulation of RNA polymerase II transcription preinitiation complex assembly 3/227 11/18866 0.000264125 0.000826629 0.000312253 TP53/ESR1/CREB1 3

BP GO:0051712 positive regulation of killing of cells of other organism 3/227 11/18866 0.000264125 0.000826629 0.000312253 NOS2/SYK/IFNG 3

BP GO:0060439 trachea morphogenesis 3/227 11/18866 0.000264125 0.000826629 0.000312253 MAPK1/MAPK3/SHH 3

BP GO:0070417 cellular response to cold 3/227 11/18866 0.000264125 0.000826629 0.000312253 FOXO1/EIF2AK3/EIF2AK4 3

BP GO:0070757 interleukin-35-mediated signaling pathway 3/227 11/18866 0.000264125 0.000826629 0.000312253 STAT1/STAT3/JAK1 3

BP GO:0071803 positive regulation of podosome assembly 3/227 11/18866 0.000264125 0.000826629 0.000312253 TNF/CSF2/MAPK9 3

BP GO:1990440 positive regulation of transcription from RNA polymerase II promoter in response to endoplasmic reticulum stress 3/227 11/18866 0.000264125 0.000826629 0.000312253 TP53/ATF3/DDIT3 3

BP GO:0032481 positive regulation of type I interferon production 6/227 74/18866 0.000266005 0.000831966 0.000314269 RELA/STAT1/TLR4/SYK/NFKB1/EP300 6

BP GO:0003015 heart process 12/227 297/18866 0.000267276 0.000835392 0.000315563 PIK3CG/SOD1/EDN1/GJA1/MTOR/CDC42/MDM2/IL2/GPX1/TRPV1/DRD2/PDE5A 12

BP GO:0045601 regulation of endothelial cell differentiation 5/227 48/18866 0.00027112 0.000845739 0.000319472 VEGFA/TNF/XDH/IL1B/TNFRSF1A 5

BP GO:0055023 positive regulation of cardiac muscle tissue growth 5/227 48/18866 0.00027112 0.000845739 0.000319472 MAPK1/CDK1/MAPK14/EDN1/MTOR 5

BP GO:1900271 regulation of long-term synaptic potentiation 5/227 48/18866 0.00027112 0.000845739 0.000319472 CHRNA7/APP/CREB1/DRD2/EIF2AK4 5

BP GO:0048259 regulation of receptor-mediated endocytosis 7/227 105/18866 0.000278982 0.000869123 0.000328305 VEGFA/EGF/SYK/SELE/IL4/INSR/DRD2 7

BP GO:1902106 negative regulation of leukocyte differentiation 7/227 105/18866 0.000278982 0.000869123 0.000328305 MYC/TLR4/CDK6/ERBB2/IL2/IL4/SHH 7

BP GO:0007272 ensheathment of neurons 8/227 139/18866 0.000283156 0.000880971 0.000332781 AKT1/SOD1/PPARD/NTRK2/MTOR/ERBB2/TNFRSF1B/EIF2AK3 8

BP GO:0008366 axon ensheathment 8/227 139/18866 0.000283156 0.000880971 0.000332781 AKT1/SOD1/PPARD/NTRK2/MTOR/ERBB2/TNFRSF1B/EIF2AK3 8

BP GO:0033143 regulation of intracellular steroid hormone receptor signaling pathway 6/227 75/18866 0.000286268 0.000888906 0.000335778 AR/ESR1/PARP1/SIRT1/BRCA1/EP300 6

BP GO:0035924 cellular response to vascular endothelial growth factor stimulus 6/227 75/18866 0.000286268 0.000888906 0.000335778 RELA/AKT1/VEGFA/XDH/KDR/MAPK14 6

BP GO:0072088 nephron epithelium morphogenesis 6/227 75/18866 0.000286268 0.000888906 0.000335778 BCL2/VEGFA/STAT1/MYC/GDNF/SHH 6

BP GO:0002825 regulation of T-helper 1 type immune response 4/227 27/18866 0.000288213 0.000892615 0.000337179 IL1B/CD80/CCR2/PLA2G4A 4

BP GO:0060544 regulation of necroptotic process 4/227 27/18866 0.000288213 0.000892615 0.000337179 CASP8/CFLAR/BIRC3/BIRC2 4

BP GO:0062098 regulation of programmed necrotic cell death 4/227 27/18866 0.000288213 0.000892615 0.000337179 CASP8/CFLAR/BIRC3/BIRC2 4

BP GO:0072539 T-helper 17 cell differentiation 4/227 27/18866 0.000288213 0.000892615 0.000337179 IL6/STAT3/IL2/IL4 4

BP GO:0002708 positive regulation of lymphocyte mediated immunity 7/227 106/18866 0.000295699 0.000915205 0.000345712 TGFB1/TNF/IL6/IL1B/CD28/IL2/IL4 7

BP GO:0060968 regulation of gene silencing 8/227 140/18866 0.000297258 0.000918831 0.000347082 TGFB1/PPARG/EGFR/TNF/IL6/TP53/ESR1/STAT3 8

BP GO:0072655 establishment of protein localization to mitochondrion 8/227 140/18866 0.000297258 0.000918831 0.000347082 BCL2/BAX/CASP8/AKT1/TP53/DDIT3/HSP90AA1/MAPK8 8

BP GO:0002686 negative regulation of leukocyte migration 5/227 49/18866 0.000299 0.000921222 0.000347985 DPP4/AKT1/HMOX1/CYP19A1/CCL2 5

BP GO:0006111 regulation of gluconeogenesis 5/227 49/18866 0.000299 0.000921222 0.000347985 GPT/SIRT1/FOXO1/PPARA/EP300 5

BP GO:0010712 regulation of collagen metabolic process 5/227 49/18866 0.000299 0.000921222 0.000347985 TGFB1/PPARG/IL6/PPARD/F2 5

BP GO:0015872 dopamine transport 5/227 49/18866 0.000299 0.000921222 0.000347985 SLC6A3/HTR2A/GDNF/SNCA/DRD2 5

BP GO:0030850 prostate gland development 5/227 49/18866 0.000299 0.000921222 0.000347985 RXRA/AR/ESR1/CYP19A1/SHH 5

BP GO:0071559 response to transforming growth factor beta 11/227 258/18866 0.000304524 0.000937635 0.000354185 JUN/TGFB1/FOS/TP53/PARP1/SIRT1/EDN1/CFLAR/CREB1/EP300/TGFBR1 11

BP GO:0021537 telencephalon development 11/227 259/18866 0.000314727 0.000968421 0.000365814 BAX/CASP3/EGFR/HIF1A/NTRK2/CDK5/CDK6/PSEN1/SHH/CSF1R/DRD2 11

BP GO:0010718 positive regulation of epithelial to mesenchymal transition 5/227 50/18866 0.000328999 0.001009725 0.000381416 TGFB1/IL6/IL1B/MTOR/TGFBR1 5

BP GO:0030195 negative regulation of blood coagulation 5/227 50/18866 0.000328999 0.001009725 0.000381416 PLAU/F2/EDN1/PLAT/PRKG1 5

BP GO:0045646 regulation of erythrocyte differentiation 5/227 50/18866 0.000328999 0.001009725 0.000381416 HIF1A/STAT1/CDK6/MAPK14/STAT3 5

BP GO:0050873 brown fat cell differentiation 5/227 50/18866 0.000328999 0.001009725 0.000381416 PTGS2/MAPK14/SIRT1/MTOR/TRPV4 5

BP GO:0002200 somatic diversification of immune receptors 6/227 77/18866 0.000330417 0.001012768 0.000382566 TGFB1/IL10/CD28/IL2/IL4/CD40LG 6

BP GO:0072028 nephron morphogenesis 6/227 77/18866 0.000330417 0.001012768 0.000382566 BCL2/VEGFA/STAT1/MYC/GDNF/SHH 6

BP GO:0044774 mitotic DNA integrity checkpoint 7/227 108/18866 0.000331546 0.001015085 0.000383441 BAX/CDKN1A/TP53/CDK1/MDM2/ATF2/EP300 7

BP GO:0007205 protein kinase C-activating G protein-coupled receptor signaling pathway 4/227 28/18866 0.000333095 0.001015085 0.000383441 EDN1/IL2/BDKRB1/HTR2B 4

BP GO:0030194 positive regulation of blood coagulation 4/227 28/18866 0.000333095 0.001015085 0.000383441 NFE2L2/TLR4/F2/PLA2G4A 4

BP GO:0033598 mammary gland epithelial cell proliferation 4/227 28/18866 0.000333095 0.001015085 0.000383441 BAX/MAPK1/ESR1/GPX1 4

BP GO:0045830 positive regulation of isotype switching 4/227 28/18866 0.000333095 0.001015085 0.000383441 TGFB1/CD28/IL2/IL4 4

BP GO:0060512 prostate gland morphogenesis 4/227 28/18866 0.000333095 0.001015085 0.000383441 RXRA/AR/ESR1/SHH 4

BP GO:0095500 acetylcholine receptor signaling pathway 4/227 28/18866 0.000333095 0.001015085 0.000383441 CHRM3/CHRNA7/OPRM1/HRH3 4

BP GO:1900048 positive regulation of hemostasis 4/227 28/18866 0.000333095 0.001015085 0.000383441 NFE2L2/TLR4/F2/PLA2G4A 4

BP GO:2000191 regulation of fatty acid transport 4/227 28/18866 0.000333095 0.001015085 0.000383441 AKT1/IL1B/SYK/EDN1 4

BP GO:1901796 regulation of signal transduction by p53 class mediator 9/227 180/18866 0.00034002 0.001035527 0.000391163 BCL2/AKT1/TP53/CDK5/MAPK14/SIRT1/BRCA1/MDM2/EP300 9

BP GO:0000012 single strand break repair 3/227 12/18866 0.000349044 0.00105289 0.000397722 PARP1/SIRT1/TDP1 3

BP GO:0006983 ER overload response 3/227 12/18866 0.000349044 0.00105289 0.000397722 TP53/DDIT3/EIF2AK3 3

BP GO:0007077 mitotic nuclear envelope disassembly 3/227 12/18866 0.000349044 0.00105289 0.000397722 PRKCA/CDK1/PRKCB 3

BP GO:0010232 vascular transport 3/227 12/18866 0.000349044 0.00105289 0.000397722 ABCG2/ABCB1/GJA1 3

BP GO:0031392 regulation of prostaglandin biosynthetic process 3/227 12/18866 0.000349044 0.00105289 0.000397722 PTGS2/IL1B/SIRT1 3

BP GO:0032494 response to peptidoglycan 3/227 12/18866 0.000349044 0.00105289 0.000397722 RELA/IL6/C5AR1 3

BP GO:0038110 interleukin-2-mediated signaling pathway 3/227 12/18866 0.000349044 0.00105289 0.000397722 SYK/JAK1/IL2 3

BP GO:0038180 nerve growth factor signaling pathway 3/227 12/18866 0.000349044 0.00105289 0.000397722 BDNF/NGF/NTRK1 3

BP GO:0048148 behavioral response to cocaine 3/227 12/18866 0.000349044 0.00105289 0.000397722 HTR2A/CDK5/DRD2 3

BP GO:0060442 branching involved in prostate gland morphogenesis 3/227 12/18866 0.000349044 0.00105289 0.000397722 RXRA/ESR1/SHH 3

BP GO:0061517 macrophage proliferation 3/227 12/18866 0.000349044 0.00105289 0.000397722 MAPK1/MAPK3/CSF1R 3

BP GO:0071639 positive regulation of monocyte chemotactic protein-1 production 3/227 12/18866 0.000349044 0.00105289 0.000397722 IL1B/SYK/TRPV4 3

BP GO:0097284 hepatocyte apoptotic process 3/227 12/18866 0.000349044 0.00105289 0.000397722 PIK3CG/BCL2L1/CFLAR 3

BP GO:0097340 inhibition of cysteine-type endopeptidase activity 3/227 12/18866 0.000349044 0.00105289 0.000397722 XIAP/BIRC3/BIRC2 3

BP GO:0097341 zymogen inhibition 3/227 12/18866 0.000349044 0.00105289 0.000397722 XIAP/BIRC3/BIRC2 3

BP GO:0000271 polysaccharide biosynthetic process 6/227 78/18866 0.000354403 0.001068378 0.000403572 TGFB1/AKT1/EGF/MTOR/NFKB1/INSR 6

BP GO:0042445 hormone metabolic process 10/227 221/18866 0.00035731 0.001076459 0.000406625 HIF1A/ESR1/NGF/AKR1B1/CYP19A1/TTR/CYP3A4/CTSB/SHH/REN 10

BP GO:0046434 organophosphate catabolic process 8/227 144/18866 0.000359524 0.001081756 0.000408626 PON1/XDH/VCP/ENPP2/GPX1/PLA2G4A/IDH1/PDE5A 8

BP GO:0070585 protein localization to mitochondrion 8/227 144/18866 0.000359524 0.001081756 0.000408626 BCL2/BAX/CASP8/AKT1/TP53/DDIT3/HSP90AA1/MAPK8 8

BP GO:0045912 negative regulation of carbohydrate metabolic process 5/227 51/18866 0.000361223 0.001085496 0.000410038 TGFB1/TP53/STAT3/PPARA/EP300 5

BP GO:1900047 negative regulation of hemostasis 5/227 51/18866 0.000361223 0.001085496 0.000410038 PLAU/F2/EDN1/PLAT/PRKG1 5

BP GO:1901990 regulation of mitotic cell cycle phase transition 15/227 448/18866 0.000362603 0.001088955 0.000411345 BCL2/BAX/EGFR/AKT1/CDKN1A/TP53/HSP90AA1/APP/CDK1/CDK6/CCL2/BRCA1/MDM2/PTPN6/EP300 15

BP GO:0072009 nephron epithelium development 7/227 110/18866 0.000370797 0.001112158 0.00042011 BCL2/VEGFA/STAT1/MYC/GDNF/PECAM1/SHH 7

BP GO:1901800 positive regulation of proteasomal protein catabolic process 7/227 110/18866 0.000370797 0.001112158 0.00042011 AKT1/NFE2L2/GCLC/MDM2/VCP/PSEN1/MAPK9 7

BP GO:1900034 regulation of cellular response to heat 6/227 79/18866 0.00037973 0.001138233 0.000429959 MAPK1/MAPK3/HSP90AA1/SIRT1/MTOR/EP300 6

BP GO:0032412 regulation of ion transmembrane transporter activity 11/227 265/18866 0.000382185 0.001140148 0.000430683 CHRM3/OPRM1/MMP9/APP/CDK5/ABCB1/CCL2/CCR2/IFNG/GRIA2/DRD2 11

BP GO:0001773 myeloid dendritic cell activation 4/227 29/18866 0.000382767 0.001140148 0.000430683 IL10/IL4/PSEN1/CSF2 4

BP GO:0007263 nitric oxide mediated signal transduction 4/227 29/18866 0.000382767 0.001140148 0.000430683 NOS2/EGFR/VEGFA/PDE5A 4

BP GO:0008209 androgen metabolic process 4/227 29/18866 0.000382767 0.001140148 0.000430683 ESR1/CYP19A1/CYP3A4/SHH 4

BP GO:0036296 response to increased oxygen levels 4/227 29/18866 0.000382767 0.001140148 0.000430683 PPARG/CDKN1A/CAT/FOXO1 4

BP GO:0043032 positive regulation of macrophage activation 4/227 29/18866 0.000382767 0.001140148 0.000430683 IL10/TLR4/MMP8/PLA2G4A 4

BP GO:0045822 negative regulation of heart contraction 4/227 29/18866 0.000382767 0.001140148 0.000430683 PIK3CG/IL2/TRPV1/PDE5A 4

BP GO:0050901 leukocyte tethering or rolling 4/227 29/18866 0.000382767 0.001140148 0.000430683 TNF/SELE/VCAM1/CCR2 4

BP GO:0071624 positive regulation of granulocyte chemotaxis 4/227 29/18866 0.000382767 0.001140148 0.000430683 EDN1/CXCL8/C5AR1/IL4 4

BP GO:2000108 positive regulation of leukocyte apoptotic process 4/227 29/18866 0.000382767 0.001140148 0.000430683 BAX/IL10/TP53/SIRT1 4

BP GO:0034440 lipid oxidation 7/227 111/18866 0.000391771 0.001166236 0.000440537 PPARG/AKT1/PPARD/MAPK14/PPARA/MTOR/ALOX5 7

BP GO:0090287 regulation of cellular response to growth factor stimulus 12/227 310/18866 0.000393704 0.001171257 0.000442434 TGFB1/TP53/XDH/HIF1A/IL1B/SIRT1/PRKCB/XIAP/CFLAR/EP300/TGFBR1/TMPRSS6 12

BP GO:0060421 positive regulation of heart growth 5/227 52/18866 0.000395776 0.001175951 0.000444207 MAPK1/CDK1/MAPK14/EDN1/MTOR 5

BP GO:0060425 lung morphogenesis 5/227 52/18866 0.000395776 0.001175951 0.000444207 MAPK1/TNF/MAPK3/CDC42/SHH 5

BP GO:0031397 negative regulation of protein ubiquitination 6/227 80/18866 0.000406451 0.001206912 0.000455903 AKT1/GCLC/CDK5/MTOR/PRKCG/PSEN1 6

BP GO:0007043 cell-cell junction assembly 8/227 147/18866 0.000412876 0.001224463 0.000462532 PRKCA/TNF/IL1B/GJA1/PECAM1/GJB1/TRPV4/NR1H4 8

BP GO:1904064 positive regulation of cation transmembrane transport 8/227 147/18866 0.000412876 0.001224463 0.000462532 BAX/SNCA/CDK5/F2/CCL2/CCR2/IFNG/BDKRB1 8

BP GO:0005976 polysaccharide metabolic process 7/227 112/18866 0.000413683 0.001226091 0.000463147 TGFB1/AKT1/EGF/MGAM/MTOR/NFKB1/INSR 7

BP GO:2001257 regulation of cation channel activity 9/227 185/18866 0.000415538 0.001230821 0.000464934 OPRM1/MMP9/APP/CDK5/CCL2/CCR2/IFNG/GRIA2/DRD2 9

BP GO:0008277 regulation of G protein-coupled receptor signaling pathway 8/227 148/18866 0.000432027 0.001277081 0.000482408 OPRM1/PRKCA/SNCA/F2/EDN1/CXCL8/DRD2/HTR2B 8

BP GO:0071901 negative regulation of protein serine/threonine kinase activity 8/227 148/18866 0.000432027 0.001277081 0.000482408 CASP3/AKT1/CDKN1A/IL1B/SIRT1/GSTP1/HMGCR/PTPN6 8

BP GO:1903670 regulation of sprouting angiogenesis 8/227 148/18866 0.000432027 0.001277081 0.000482408 PTGS2/VEGFA/IL10/KDR/HMOX1/JAK1/AGTR1/ALOX5 8

BP GO:0032964 collagen biosynthetic process 5/227 53/18866 0.000432768 0.001277081 0.000482408 TGFB1/PPARG/IL6/PPARD/F2 5

BP GO:0045661 regulation of myoblast differentiation 5/227 53/18866 0.000432768 0.001277081 0.000482408 TGFB1/TNF/PPARD/DDIT3/MAPK14 5

BP GO:0045744 negative regulation of G protein-coupled receptor signaling pathway 5/227 53/18866 0.000432768 0.001277081 0.000482408 OPRM1/SNCA/CXCL8/DRD2/HTR2B 5

BP GO:0001960 negative regulation of cytokine-mediated signaling pathway 6/227 81/18866 0.000434617 0.001281743 0.000484169 PPARG/IL6/MMP12/NR1H3/GSTP1/NR1H4 6

BP GO:0010800 positive regulation of peptidyl-threonine phosphorylation 4/227 30/18866 0.000437515 0.001284712 0.000485291 TGFB1/MAPK1/EGF/APP 4

BP GO:0034110 regulation of homotypic cell-cell adhesion 4/227 30/18866 0.000437515 0.001284712 0.000485291 PRKCA/SYK/JAK1/PRKG1 4

BP GO:0035666 TRIF-dependent toll-like receptor signaling pathway 4/227 30/18866 0.000437515 0.001284712 0.000485291 CASP8/TLR4/BIRC3/BIRC2 4

BP GO:0061082 myeloid leukocyte cytokine production 4/227 30/18866 0.000437515 0.001284712 0.000485291 TGFB1/HMOX1/TLR4/SIRT1 4

BP GO:0071480 cellular response to gamma radiation 4/227 30/18866 0.000437515 0.001284712 0.000485291 BCL2L1/CDKN1A/TP53/MDM2 4

BP GO:1905144 response to acetylcholine 4/227 30/18866 0.000437515 0.001284712 0.000485291 CHRM3/CHRNA7/OPRM1/HRH3 4

BP GO:1905145 cellular response to acetylcholine 4/227 30/18866 0.000437515 0.001284712 0.000485291 CHRM3/CHRNA7/OPRM1/HRH3 4

BP GO:0016064 immunoglobulin mediated immune response 10/227 227/18866 0.000441287 0.001294986 0.000489172 TGFB1/IL10/TNF/CRP/CD28/IL2/IL4/CD40LG/PTPN6/C1R 10

BP GO:0045333 cellular respiration 9/227 187/18866 0.000449377 0.001308474 0.000494267 NOS2/HIF1A/SNCA/CAT/CDK1/IFNG/IL4/VCP/IDH1 9

BP GO:0002674 negative regulation of acute inflammatory response 3/227 13/18866 0.000449736 0.001308474 0.000494267 PPARG/IL4/GSTP1 3

BP GO:0014842 regulation of skeletal muscle satellite cell proliferation 3/227 13/18866 0.000449736 0.001308474 0.000494267 PPARD/STAT3/CFLAR 3

BP GO:0016264 gap junction assembly 3/227 13/18866 0.000449736 0.001308474 0.000494267 IL1B/GJA1/GJB1 3

BP GO:0031650 regulation of heat generation 3/227 13/18866 0.000449736 0.001308474 0.000494267 PTGS2/TNF/IL1B 3

BP GO:0032725 positive regulation of granulocyte macrophage colony-stimulating factor production 3/227 13/18866 0.000449736 0.001308474 0.000494267 IL1B/SYK/CD80 3

BP GO:0033089 positive regulation of T cell differentiation in thymus 3/227 13/18866 0.000449736 0.001308474 0.000494267 IL1B/IL1A/SHH 3

BP GO:0048266 behavioral response to pain 3/227 13/18866 0.000449736 0.001308474 0.000494267 NTRK1/TRPV1/SCN9A 3

BP GO:0048302 regulation of isotype switching to IgG isotypes 3/227 13/18866 0.000449736 0.001308474 0.000494267 CD28/IL2/IL4 3

BP GO:0071287 cellular response to manganese ion 3/227 13/18866 0.000449736 0.001308474 0.000494267 APP/EIF2S1/EIF2AK3 3

BP GO:0071352 cellular response to interleukin-2 3/227 13/18866 0.000449736 0.001308474 0.000494267 SYK/JAK1/IL2 3

BP GO:0072683 T cell extravasation 3/227 13/18866 0.000449736 0.001308474 0.000494267 ICAM1/CCL2/CCR2 3

BP GO:1902947 regulation of tau-protein kinase activity 3/227 13/18866 0.000449736 0.001308474 0.000494267 HSP90AA1/C5AR1/IFNG 3

BP GO:2000121 regulation of removal of superoxide radicals 3/227 13/18866 0.000449736 0.001308474 0.000494267 TNF/NFE2L2/DHFR 3

BP GO:0031056 regulation of histone modification 8/227 149/18866 0.000451894 0.001313946 0.000496334 VEGFA/TP53/IL1B/SNCA/MAPK3/SIRT1/MAPK8/BRCA1 8

BP GO:0030258 lipid modification 11/227 271/18866 0.000461401 0.00134077 0.000506466 PIK3CG/PPARG/AKT1/PPARD/MAPK14/PPARA/AGTR1/MTOR/CYP3A4/ALOX5/ABCG1 11

BP GO:0006584 catecholamine metabolic process 5/227 54/18866 0.000472306 0.001365781 0.000515914 SLC6A3/SNCA/AKR1B1/HTR1A/DRD2 5

BP GO:0006754 ATP biosynthetic process 5/227 54/18866 0.000472306 0.001365781 0.000515914 TGFB1/PARP1/STAT3/IL4/VCP 5

BP GO:0006968 cellular defense response 5/227 54/18866 0.000472306 0.001365781 0.000515914 RELA/C5AR1/CCR2/CCR3/CCR5 5

BP GO:0009712 catechol-containing compound metabolic process 5/227 54/18866 0.000472306 0.001365781 0.000515914 SLC6A3/SNCA/AKR1B1/HTR1A/DRD2 5

BP GO:0010524 positive regulation of calcium ion transport into cytosol 5/227 54/18866 0.000472306 0.001365781 0.000515914 BAX/SNCA/F2/BAK1/BDKRB1 5

BP GO:0030857 negative regulation of epithelial cell differentiation 5/227 54/18866 0.000472306 0.001365781 0.000515914 VEGFA/MMP9/XDH/STAT1/IFNG 5

BP GO:1901185 negative regulation of ERBB signaling pathway 5/227 54/18866 0.000472306 0.001365781 0.000515914 EGFR/EGF/CDC42/ERBB2/PSEN1 5

BP GO:2000179 positive regulation of neural precursor cell proliferation 5/227 54/18866 0.000472306 0.001365781 0.000515914 VEGFA/EGF/HIF1A/SHH/DRD2 5

BP GO:0019724 B cell mediated immunity 10/227 230/18866 0.000489066 0.001412528 0.000533573 TGFB1/IL10/TNF/CRP/CD28/IL2/IL4/CD40LG/PTPN6/C1R 10

BP GO:0098657 import into cell 10/227 230/18866 0.000489066 0.001412528 0.000533573 SLC6A3/SLC6A4/AKT1/GDNF/SNCA/IFNG/TRPV4/PSEN1/TRPV1/DRD2 10

BP GO:0001570 vasculogenesis 6/227 83/18866 0.000495505 0.001429387 0.000539941 TGFB1/VEGFA/XDH/KDR/NTRK2/SHH 6

BP GO:2000243 positive regulation of reproductive process 6/227 83/18866 0.000495505 0.001429387 0.000539941 AR/VEGFA/INSR/SHH/TACR1/PDE5A 6

BP GO:0014044 Schwann cell development 4/227 31/18866 0.000497626 0.001429436 0.000539959 AKT1/SOD1/NTRK2/CDK5 4

BP GO:0034368 protein-lipid complex remodeling 4/227 31/18866 0.000497626 0.001429436 0.000539959 MPO/AGTR1/CETP/ABCG1 4

BP GO:0034369 plasma lipoprotein particle remodeling 4/227 31/18866 0.000497626 0.001429436 0.000539959 MPO/AGTR1/CETP/ABCG1 4

BP GO:0045948 positive regulation of translational initiation 4/227 31/18866 0.000497626 0.001429436 0.000539959 RXRA/TNF/MTOR/EIF2AK4 4

BP GO:0072538 T-helper 17 type immune response 4/227 31/18866 0.000497626 0.001429436 0.000539959 IL6/STAT3/IL2/IL4 4

BP GO:0090075 relaxation of muscle 4/227 31/18866 0.000497626 0.001429436 0.000539959 SOD1/KCNMA1/PRKG1/PDE5A 4

BP GO:1902253 regulation of intrinsic apoptotic signaling pathway by p53 class mediator 4/227 31/18866 0.000497626 0.001429436 0.000539959 BCL2/TP53/SIRT1/MDM2 4

BP GO:0002931 response to ischemia 5/227 55/18866 0.000514504 0.001472579 0.000556256 BCL2/CASP9/TP53/GJA1/CSF1R 5

BP GO:0032720 negative regulation of tumor necrosis factor production 5/227 55/18866 0.000514504 0.001472579 0.000556256 CHRNA7/IL10/TLR4/GSTP1/PTPN6 5

BP GO:0035065 regulation of histone acetylation 5/227 55/18866 0.000514504 0.001472579 0.000556256 IL1B/SNCA/MAPK3/SIRT1/BRCA1 5

BP GO:0042304 regulation of fatty acid biosynthetic process 5/227 55/18866 0.000514504 0.001472579 0.000556256 PTGS2/IL1B/SIRT1/BRCA1/NR1H3 5

BP GO:0050433 regulation of catecholamine secretion 5/227 55/18866 0.000514504 0.001472579 0.000556256 HTR2A/GDNF/SNCA/HRH3/DRD2 5

BP GO:0050819 negative regulation of coagulation 5/227 55/18866 0.000514504 0.001472579 0.000556256 PLAU/F2/EDN1/PLAT/PRKG1 5

BP GO:0002224 toll-like receptor signaling pathway 8/227 152/18866 0.000515987 0.001475933 0.000557523 CASP8/ESR1/TLR4/BIRC3/NR1H3/CTSB/NR1H4/BIRC2 8

BP GO:0072676 lymphocyte migration 7/227 117/18866 0.000538401 0.00153912 0.000581392 PIK3CG/AKT1/APP/ICAM1/CCL2/CCR2/RET 7

BP GO:0001678 cellular glucose homeostasis 8/227 153/18866 0.000538917 0.001539671 0.0005816 HIF1A/GCLC/SIRT1/IGF1R/FOXO1/ICAM1/HMGCR/NR1H4 8

BP GO:0001755 neural crest cell migration 5/227 56/18866 0.000559475 0.001594944 0.000602479 HIF1A/GDNF/SHH/HTR2B/RET 5

BP GO:0070228 regulation of lymphocyte apoptotic process 5/227 56/18866 0.000559475 0.001594944 0.000602479 BAX/IL10/TP53/HIF1A/IL2 5

BP GO:0032092 positive regulation of protein binding 6/227 85/18866 0.000562835 0.001594944 0.000602479 MMP9/BDNF/APP/CDK5/PSEN1/EP300 6

BP GO:0070509 calcium ion import 6/227 85/18866 0.000562835 0.001594944 0.000602479 EGF/CDK5/CCL2/TRPV4/PSEN2/TRPV1 6

BP GO:0002724 regulation of T cell cytokine production 4/227 32/18866 0.00056339 0.001594944 0.000602479 IL6/IL1B/CCR2/TNFRSF1B 4

BP GO:0032673 regulation of interleukin-4 production 4/227 32/18866 0.00056339 0.001594944 0.000602479 DDIT3/SYK/CD28/CD40LG 4

BP GO:0034367 protein-containing complex remodeling 4/227 32/18866 0.00056339 0.001594944 0.000602479 MPO/AGTR1/CETP/ABCG1 4

BP GO:0034694 response to prostaglandin 4/227 32/18866 0.00056339 0.001594944 0.000602479 PPARG/AKT1/EDN1/GNRH1 4

BP GO:0042744 hydrogen peroxide catabolic process 4/227 32/18866 0.00056339 0.001594944 0.000602479 SNCA/CAT/MPO/GPX1 4

BP GO:0046320 regulation of fatty acid oxidation 4/227 32/18866 0.00056339 0.001594944 0.000602479 PPARG/AKT1/PPARA/MTOR 4

BP GO:0051385 response to mineralocorticoid 4/227 32/18866 0.00056339 0.001594944 0.000602479 FOS/CDKN1A/PARP1/EDN1 4

BP GO:0097421 liver regeneration 4/227 32/18866 0.00056339 0.001594944 0.000602479 EGFR/IL10/IL6/HMOX1 4

BP GO:1902235 regulation of endoplasmic reticulum stress-induced intrinsic apoptotic signaling pathway 4/227 32/18866 0.00056339 0.001594944 0.000602479 BCL2L1/DDIT3/SIRT1/EIF2AK3 4

BP GO:1903959 regulation of anion transmembrane transport 4/227 32/18866 0.00056339 0.001594944 0.000602479 AKT1/ABCB1/MTOR/PSEN1 4

BP GO:0014841 skeletal muscle satellite cell proliferation 3/227 14/18866 0.000567322 0.001594944 0.000602479 PPARD/STAT3/CFLAR 3

BP GO:0014854 response to inactivity 3/227 14/18866 0.000567322 0.001594944 0.000602479 IL10/CAT/DRD2 3

BP GO:0032310 prostaglandin secretion 3/227 14/18866 0.000567322 0.001594944 0.000602479 NOS2/IL1B/EDN1 3

BP GO:0045898 regulation of RNA polymerase II transcription preinitiation complex assembly 3/227 14/18866 0.000567322 0.001594944 0.000602479 TP53/ESR1/CREB1 3

BP GO:0047484 regulation of response to osmotic stress 3/227 14/18866 0.000567322 0.001594944 0.000602479 PTGS2/ABCB1/TRPV4 3

BP GO:0048291 isotype switching to IgG isotypes 3/227 14/18866 0.000567322 0.001594944 0.000602479 CD28/IL2/IL4 3

BP GO:0051709 regulation of killing of cells of other organism 3/227 14/18866 0.000567322 0.001594944 0.000602479 NOS2/SYK/IFNG 3

BP GO:0070669 response to interleukin-2 3/227 14/18866 0.000567322 0.001594944 0.000602479 SYK/JAK1/IL2 3

BP GO:0071236 cellular response to antibiotic 3/227 14/18866 0.000567322 0.001594944 0.000602479 TP53/MDM2/PLA2G4A 3

BP GO:0072216 positive regulation of metanephros development 3/227 14/18866 0.000567322 0.001594944 0.000602479 MYC/GDNF/RET 3

BP GO:0099550 trans-synaptic signaling, modulating synaptic transmission 3/227 14/18866 0.000567322 0.001594944 0.000602479 BDNF/NTRK2/PLAT 3

BP GO:1902894 negative regulation of pri-miRNA transcription by RNA polymerase II 3/227 14/18866 0.000567322 0.001594944 0.000602479 RELA/PPARD/PPARA 3

BP GO:2001279 regulation of unsaturated fatty acid biosynthetic process 3/227 14/18866 0.000567322 0.001594944 0.000602479 PTGS2/IL1B/SIRT1 3

BP GO:0071706 tumor necrosis factor superfamily cytokine production 7/227 119/18866 0.000595959 0.001674462 0.000632516 CHRNA7/IL10/TLR4/APP/IFNG/GSTP1/PTPN6 7

BP GO:0044242 cellular lipid catabolic process 10/227 236/18866 0.000597564 0.001676989 0.00063347 PIK3CG/AKT1/PPARD/FUCA1/PPARA/MTOR/GM2A/ENPP2/PLA2G4A/IDH1 10

BP GO:0060560 developmental growth involved in morphogenesis 10/227 236/18866 0.000597564 0.001676989 0.00063347 MAP2/VEGFA/ESR1/BDNF/NGF/HSP90AA1/APP/CDK5/SHH/EIF2AK4 10

BP GO:0034109 homotypic cell-cell adhesion 6/227 86/18866 0.000599059 0.001680191 0.00063468 PIK3CG/PRKCA/SYK/JAK1/PTPN6/PRKG1 6

BP GO:0019748 secondary metabolic process 5/227 57/18866 0.000607333 0.00169739 0.000641177 BCL2/NFE2L2/AKR1B1/TYR/PAM 5

BP GO:0030520 intracellular estrogen receptor signaling pathway 5/227 57/18866 0.000607333 0.00169739 0.000641177 AR/ESR2/ESR1/PARP1/BRCA1 5

BP GO:0050432 catecholamine secretion 5/227 57/18866 0.000607333 0.00169739 0.000641177 HTR2A/GDNF/SNCA/HRH3/DRD2 5

BP GO:0060760 positive regulation of response to cytokine stimulus 5/227 57/18866 0.000607333 0.00169739 0.000641177 HIF1A/TLR4/MMP12/EDN1/CASP1 5

BP GO:0071398 cellular response to fatty acid 5/227 57/18866 0.000607333 0.00169739 0.000641177 PPARG/AKT1/EDN1/CREB1/NR1H4 5

BP GO:1903556 negative regulation of tumor necrosis factor superfamily cytokine production 5/227 57/18866 0.000607333 0.00169739 0.000641177 CHRNA7/IL10/TLR4/GSTP1/PTPN6 5

BP GO:0030902 hindbrain development 8/227 156/18866 0.000612704 0.001711396 0.000646468 SLC6A4/BCL2/EGF/TP53/CDK5/C5AR1/PSEN1/SHH 8

BP GO:0051588 regulation of neurotransmitter transport 7/227 120/18866 0.000626516 0.001748946 0.000660652 HTR2A/GDNF/SNCA/CDK5/PRKCB/PRKCG/DRD2 7

BP GO:0002861 regulation of inflammatory response to antigenic stimulus 4/227 33/18866 0.000635097 0.001764613 0.00066657 IL10/TNF/CD28/GPX1 4

BP GO:0010464 regulation of mesenchymal cell proliferation 4/227 33/18866 0.000635097 0.001764613 0.00066657 VEGFA/STAT1/MYC/SHH 4

BP GO:0010955 negative regulation of protein processing 4/227 33/18866 0.000635097 0.001764613 0.00066657 XIAP/BIRC3/MDM2/BIRC2 4

BP GO:0033028 myeloid cell apoptotic process 4/227 33/18866 0.000635097 0.001764613 0.00066657 BCL2/IL6/SIRT1/CCR5 4

BP GO:0040018 positive regulation of multicellular organism growth 4/227 33/18866 0.000635097 0.001764613 0.00066657 SLC6A3/BCL2/CREB1/DRD2 4

BP GO:0048384 retinoic acid receptor signaling pathway 4/227 33/18866 0.000635097 0.001764613 0.00066657 RXRA/PPARG/RXRB/RXRG 4

BP GO:0090022 regulation of neutrophil chemotaxis 4/227 33/18866 0.000635097 0.001764613 0.00066657 DPP4/EDN1/CXCL8/C5AR1 4

BP GO:1903318 negative regulation of protein maturation 4/227 33/18866 0.000635097 0.001764613 0.00066657 XIAP/BIRC3/MDM2/BIRC2 4

BP GO:0048013 ephrin receptor signaling pathway 6/227 87/18866 0.000637066 0.001768015 0.000667855 MMP2/MMP9/NTRK1/CDC42/PSEN1/PSEN2 6

BP GO:0060761 negative regulation of response to cytokine stimulus 6/227 87/18866 0.000637066 0.001768015 0.000667855 PPARG/IL6/MMP12/NR1H3/GSTP1/NR1H4 6

BP GO:0030111 regulation of Wnt signaling pathway 13/227 375/18866 0.000645749 0.001791067 0.000676563 TGFB1/EGFR/EGF/ESR1/DDIT3/APP/MAPK14/FOXO1/XIAP/NFKB1/VCP/PSEN1/SHH 13

BP GO:0006898 receptor-mediated endocytosis 12/227 328/18866 0.000649539 0.001800529 0.000680137 ACHE/VEGFA/EGF/SNCA/HSP90AA1/SYK/SELE/CXCL8/IL4/INSR/DRD2/HTR2B 12

BP GO:0009749 response to glucose 9/227 197/18866 0.000654378 0.001811831 0.000684406 CASP3/HIF1A/PPARD/GCLC/IGF1R/GJA1/ICAM1/HMGCR/NR1H4 9

BP GO:0051216 cartilage development 9/227 197/18866 0.000654378 0.001811831 0.000684406 TGFB1/RELA/HIF1A/MAPK3/MAPK14/EDN1/TRPV4/EIF2AK3/TGFBR1 9

BP GO:0010656 negative regulation of muscle cell apoptotic process 5/227 58/18866 0.000658195 0.001818461 0.000686911 HMOX1/NFE2L2/EDN1/CFLAR/HMGCR 5

BP GO:1903793 positive regulation of anion transport 5/227 58/18866 0.000658195 0.001818461 0.000686911 IL1B/ABCB1/EDN1/PSEN1/CETP 5

BP GO:0016079 synaptic vesicle exocytosis 7/227 121/18866 0.000658303 0.001818461 0.000686911 HTR2A/SNCA/CDK5/PRKCB/PRKCG/PSEN1/DRD2 7

BP GO:0030218 erythrocyte differentiation 7/227 121/18866 0.000658303 0.001818461 0.000686911 CASP3/VEGFA/HIF1A/STAT1/CDK6/MAPK14/STAT3 7

BP GO:1903707 negative regulation of hemopoiesis 8/227 158/18866 0.000666278 0.001839423 0.000694829 MYC/NFE2L2/TLR4/CDK6/ERBB2/IL2/IL4/SHH 8

BP GO:0046470 phosphatidylcholine metabolic process 6/227 88/18866 0.000676916 0.001867705 0.000705512 PON1/ACHE/NR1H3/ENPP2/PLA2G4A/CETP 6

BP GO:0020027 hemoglobin metabolic process 3/227 15/18866 0.000702876 0.001921491 0.00072583 HIF1A/CAT/EIF2AK1 3

BP GO:0021924 cell proliferation in external granule layer 3/227 15/18866 0.000702876 0.001921491 0.00072583 SLC6A4/EGF/SHH 3

BP GO:0021930 cerebellar granule cell precursor proliferation 3/227 15/18866 0.000702876 0.001921491 0.00072583 SLC6A4/EGF/SHH 3

BP GO:0030238 male sex determination 3/227 15/18866 0.000702876 0.001921491 0.00072583 AR/INSR/GNRH1 3

BP GO:0032645 regulation of granulocyte macrophage colony-stimulating factor production 3/227 15/18866 0.000702876 0.001921491 0.00072583 IL1B/SYK/CD80 3

BP GO:0044849 estrous cycle 3/227 15/18866 0.000702876 0.001921491 0.00072583 OPRM1/MMP7/GNRH1 3

BP GO:0045651 positive regulation of macrophage differentiation 3/227 15/18866 0.000702876 0.001921491 0.00072583 CASP8/PRKCA/TGFB1 3

BP GO:0048308 organelle inheritance 3/227 15/18866 0.000702876 0.001921491 0.00072583 MAPK1/MAPK3/CDK1 3

BP GO:0048313 Golgi inheritance 3/227 15/18866 0.000702876 0.001921491 0.00072583 MAPK1/MAPK3/CDK1 3

BP GO:0048569 post-embryonic animal organ development 3/227 15/18866 0.000702876 0.001921491 0.00072583 BAX/VEGFA/BAK1 3

BP GO:0060391 positive regulation of SMAD protein signal transduction 3/227 15/18866 0.000702876 0.001921491 0.00072583 TGFB1/PARP1/TGFBR1 3

BP GO:0071801 regulation of podosome assembly 3/227 15/18866 0.000702876 0.001921491 0.00072583 TNF/CSF2/MAPK9 3

BP GO:0072075 metanephric mesenchyme development 3/227 15/18866 0.000702876 0.001921491 0.00072583 STAT1/MYC/SHH 3

BP GO:0099171 presynaptic modulation of chemical synaptic transmission 3/227 15/18866 0.000702876 0.001921491 0.00072583 CDK5/PRKCB/PRKCG 3

BP GO:1900119 positive regulation of execution phase of apoptosis 3/227 15/18866 0.000702876 0.001921491 0.00072583 BAX/IL6/TP53 3

BP GO:2001171 positive regulation of ATP biosynthetic process 3/227 15/18866 0.000702876 0.001921491 0.00072583 STAT3/IL4/VCP 3

BP GO:0001658 branching involved in ureteric bud morphogenesis 5/227 59/18866 0.000712181 0.001938136 0.000732117 BCL2/VEGFA/MYC/GDNF/SHH 5

BP GO:0060043 regulation of cardiac muscle cell proliferation 5/227 59/18866 0.000712181 0.001938136 0.000732117 MAPK1/CDK1/MAPK14/GJA1/TGFBR1 5

BP GO:0002756 MyD88-independent toll-like receptor signaling pathway 4/227 34/18866 0.000713042 0.001938136 0.000732117 CASP8/TLR4/BIRC3/BIRC2 4

BP GO:0016242 negative regulation of macroautophagy 4/227 34/18866 0.000713042 0.001938136 0.000732117 AKT1/TP53/HMOX1/MTOR 4

BP GO:0030431 sleep 4/227 34/18866 0.000713042 0.001938136 0.000732117 HTR2A/FOS/CSF2/DRD2 4

BP GO:0033198 response to ATP 4/227 34/18866 0.000713042 0.001938136 0.000732117 PTGS2/SOD1/IL1B/TRPV1 4

BP GO:0045648 positive regulation of erythrocyte differentiation 4/227 34/18866 0.000713042 0.001938136 0.000732117 HIF1A/STAT1/MAPK14/STAT3 4

BP GO:0055094 response to lipoprotein particle 4/227 34/18866 0.000713042 0.001938136 0.000732117 PPARG/AKT1/TLR4/SYK 4

BP GO:0070102 interleukin-6-mediated signaling pathway 4/227 34/18866 0.000713042 0.001938136 0.000732117 IL6/STAT1/STAT3/JAK1 4

BP GO:2000352 negative regulation of endothelial cell apoptotic process 4/227 34/18866 0.000713042 0.001938136 0.000732117 KDR/NFE2L2/ICAM1/IL4 4

BP GO:0045666 positive regulation of neuron differentiation 13/227 380/18866 0.000729867 0.001982736 0.000748964 BCL2/VEGFA/NFE2L2/BDNF/NGF/NTRK1/NTRK2/CFLAR/MTOR/IL2/PSEN1/EP300/RET 13

BP GO:0002429 immune response-activating cell surface receptor signaling pathway 15/227 481/18866 0.000755427 0.00204983 0.000774308 BCL2/BAX/RELA/MAPK1/MAPK3/HSP90AA1/SYK/PRKCB/C5AR1/CDC42/CD28/NFKB1/PSEN1/PTPN6/EP300 15

BP GO:0002757 immune response-activating signal transduction 15/227 481/18866 0.000755427 0.00204983 0.000774308 BCL2/BAX/RELA/MAPK1/MAPK3/HSP90AA1/SYK/PRKCB/C5AR1/CDC42/CD28/NFKB1/PSEN1/PTPN6/EP300 15

BP GO:0010823 negative regulation of mitochondrion organization 5/227 60/18866 0.000769409 0.002086577 0.00078819 AKT1/BCL2L1/TP53/GCLC/GPX1 5

BP GO:0007179 transforming growth factor beta receptor signaling pathway 9/227 202/18866 0.00078243 0.002120679 0.000801071 JUN/TGFB1/FOS/TP53/PARP1/SIRT1/CREB1/EP300/TGFBR1 9

BP GO:0032535 regulation of cellular component size 13/227 383/18866 0.000784636 0.002125446 0.000802872 MAP2/AKT1/VEGFA/BDNF/NGF/CDK5/EDN1/ICAM1/MTOR/KCNMA1/TRPV4/CREB1/RET 13

BP GO:0009112 nucleobase metabolic process 4/227 35/18866 0.000797517 0.002154201 0.000813734 MAPK1/XDH/TTR/MTOR 4

BP GO:0014046 dopamine secretion 4/227 35/18866 0.000797517 0.002154201 0.000813734 HTR2A/GDNF/SNCA/DRD2 4

BP GO:0014059 regulation of dopamine secretion 4/227 35/18866 0.000797517 0.002154201 0.000813734 HTR2A/GDNF/SNCA/DRD2 4

BP GO:0032633 interleukin-4 production 4/227 35/18866 0.000797517 0.002154201 0.000813734 DDIT3/SYK/CD28/CD40LG 4

BP GO:0098801 regulation of renal system process 4/227 35/18866 0.000797517 0.002154201 0.000813734 EDN1/GJA1/AGTR1/DRD2 4

BP GO:0032479 regulation of type I interferon production 7/227 125/18866 0.00079847 0.002155552 0.000814244 RELA/IL10/STAT1/TLR4/SYK/NFKB1/EP300 7

BP GO:0042475 odontogenesis of dentin-containing tooth 6/227 91/18866 0.000808132 0.002179159 0.000823162 BAX/CA2/FOXO1/PPARA/ATF2/SHH 6

BP GO:0051952 regulation of amine transport 6/227 91/18866 0.000808132 0.002179159 0.000823162 HTR2A/GDNF/SNCA/PSEN1/HRH3/DRD2 6

BP GO:0031214 biomineral tissue development 8/227 163/18866 0.000816884 0.002199019 0.000830664 PTGS2/TGFB1/HIF1A/FOXO1/PPARA/ALOX5/CCR1/EIF2AK3 8

BP GO:0055088 lipid homeostasis 8/227 163/18866 0.000816884 0.002199019 0.000830664 PPARG/SIRT1/NR1H3/NR1I2/NR1H4/CETP/ABCG1/VDR 8

BP GO:0110148 biomineralization 8/227 163/18866 0.000816884 0.002199019 0.000830664 PTGS2/TGFB1/HIF1A/FOXO1/PPARA/ALOX5/CCR1/EIF2AK3 8

BP GO:0003170 heart valve development 5/227 61/18866 0.000830001 0.00222802 0.000841619 TGFB1/MTOR/MDM2/TNFRSF1A/TNFRSF1B 5

BP GO:0046324 regulation of glucose import 5/227 61/18866 0.000830001 0.00222802 0.000841619 AKT1/TNF/NFE2L2/MAPK14/INSR 5

BP GO:0046888 negative regulation of hormone secretion 5/227 61/18866 0.000830001 0.00222802 0.000841619 IL1B/EDN1/GJA1/HMGCR/DRD2 5

BP GO:0090303 positive regulation of wound healing 5/227 61/18866 0.000830001 0.00222802 0.000841619 NFE2L2/TLR4/F2/MTOR/PLA2G4A 5

BP GO:2000756 regulation of peptidyl-lysine acetylation 5/227 61/18866 0.000830001 0.00222802 0.000841619 IL1B/SNCA/MAPK3/SIRT1/BRCA1 5

BP GO:0055007 cardiac muscle cell differentiation 7/227 126/18866 0.000836949 0.002245404 0.000848185 RXRA/TGFB1/VEGFA/CDK1/EDN1/PPARA/MTOR 7

BP GO:1901987 regulation of cell cycle phase transition 15/227 486/18866 0.000838751 0.002248968 0.000849531 BCL2/BAX/EGFR/AKT1/CDKN1A/TP53/HSP90AA1/APP/CDK1/CDK6/CCL2/BRCA1/MDM2/PTPN6/EP300 15

BP GO:0016358 dendrite development 10/227 247/18866 0.000848149 0.002272886 0.000858567 CHRNA7/MAP2/BDNF/APP/CDK5/MTOR/CDC42/IL2/PSEN1/PRKG1 10

BP GO:0031058 positive regulation of histone modification 6/227 92/18866 0.000855966 0.002279755 0.000861161 VEGFA/TP53/IL1B/MAPK3/SIRT1/BRCA1 6

BP GO:0002295 T-helper cell lineage commitment 3/227 16/18866 0.000857426 0.002279755 0.000861161 IL6/STAT3/MTOR 3

BP GO:0010225 response to UV-C 3/227 16/18866 0.000857426 0.002279755 0.000861161 TP53/BAK1/MDM2 3

BP GO:0010934 macrophage cytokine production 3/227 16/18866 0.000857426 0.002279755 0.000861161 TGFB1/TLR4/SIRT1 3

BP GO:0030397 membrane disassembly 3/227 16/18866 0.000857426 0.002279755 0.000861161 PRKCA/CDK1/PRKCB 3

BP GO:0032604 granulocyte macrophage colony-stimulating factor production 3/227 16/18866 0.000857426 0.002279755 0.000861161 IL1B/SYK/CD80 3

BP GO:0034116 positive regulation of heterotypic cell-cell adhesion 3/227 16/18866 0.000857426 0.002279755 0.000861161 IL10/TNF/IL1B 3

BP GO:0035067 negative regulation of histone acetylation 3/227 16/18866 0.000857426 0.002279755 0.000861161 SNCA/SIRT1/BRCA1 3

BP GO:0050965 detection of temperature stimulus involved in sensory perception of pain 3/227 16/18866 0.000857426 0.002279755 0.000861161 HTR2A/NTRK1/TRPV1 3

BP GO:0051081 nuclear envelope disassembly 3/227 16/18866 0.000857426 0.002279755 0.000861161 PRKCA/CDK1/PRKCB 3

BP GO:0071391 cellular response to estrogen stimulus 3/227 16/18866 0.000857426 0.002279755 0.000861161 AR/ESR1/MDM2 3

BP GO:0071732 cellular response to nitric oxide 3/227 16/18866 0.000857426 0.002279755 0.000861161 MMP3/FOXO1/CFLAR 3

BP GO:1900451 positive regulation of glutamate receptor signaling pathway 3/227 16/18866 0.000857426 0.002279755 0.000861161 CCL2/CCR2/IFNG 3

BP GO:1905050 positive regulation of metallopeptidase activity 3/227 16/18866 0.000857426 0.002279755 0.000861161 MAPK3/MAPK14/STAT3 3

BP GO:0032606 type I interferon production 7/227 127/18866 0.000876878 0.002330171 0.000880206 RELA/IL10/STAT1/TLR4/SYK/NFKB1/EP300 7

BP GO:0001569 branching involved in blood vessel morphogenesis 4/227 36/18866 0.000888817 0.002355315 0.000889704 VEGFA/KDR/EDN1/SHH 4

BP GO:0002701 negative regulation of production of molecular mediator of immune response 4/227 36/18866 0.000888817 0.002355315 0.000889704 TGFB1/IL10/TNF/HMOX1 4

BP GO:0045742 positive regulation of epidermal growth factor receptor signaling pathway 4/227 36/18866 0.000888817 0.002355315 0.000889704 AKT1/MMP9/EGF/FASLG 4

BP GO:0051973 positive regulation of telomerase activity 4/227 36/18866 0.000888817 0.002355315 0.000889704 MAPK1/MYC/MAPK3/HSP90AA1 4

BP GO:0071634 regulation of transforming growth factor beta production 4/227 36/18866 0.000888817 0.002355315 0.000889704 PTGS2/HIF1A/CREB1/ATF2 4

BP GO:0051851 modulation by host of symbiont process 5/227 62/18866 0.000894079 0.002365305 0.000893477 JUN/CRP/CSF1R/EP300/EIF2AK4 5

BP GO:0055081 anion homeostasis 5/227 62/18866 0.000894079 0.002365305 0.000893477 FASLG/SIRT1/NR1H4/CETP/ABCG1 5

BP GO:0070527 platelet aggregation 5/227 62/18866 0.000894079 0.002365305 0.000893477 PIK3CG/PRKCA/SYK/PTPN6/PRKG1 5

BP GO:0061097 regulation of protein tyrosine kinase activity 6/227 93/18866 0.000905954 0.002394056 0.000904338 EGF/BDNF/NTRK2/APP/PSEN1/CSF1R 6

BP GO:0072080 nephron tubule development 6/227 93/18866 0.000905954 0.002394056 0.000904338 BCL2/VEGFA/STAT1/MYC/GDNF/SHH 6

BP GO:0034763 negative regulation of transmembrane transport 7/227 128/18866 0.000918294 0.002425317 0.000916146 AKT1/MMP9/TNF/IL1B/PRKCB/MTOR/DRD2 7

BP GO:0060828 regulation of canonical Wnt signaling pathway 11/227 295/18866 0.000929208 0.002452779 0.00092652 TGFB1/EGFR/EGF/DDIT3/MAPK14/FOXO1/XIAP/NFKB1/VCP/PSEN1/SHH 11

BP GO:0006650 glycerophospholipid metabolic process 12/227 343/18866 0.000957997 0.002525515 0.000953995 HTR2A/PIK3CG/PON1/ACHE/HTR2C/NR1H3/ENPP2/NR1H4/PLA2G4A/CETP/CSF1R/HTR2B 12

BP GO:0060993 kidney morphogenesis 6/227 94/18866 0.000958161 0.002525515 0.000953995 BCL2/VEGFA/STAT1/MYC/GDNF/SHH 6

BP GO:1901991 negative regulation of mitotic cell cycle phase transition 10/227 251/18866 0.000958357 0.002525515 0.000953995 BCL2/BAX/CDKN1A/TP53/CDK1/CDK6/CCL2/BRCA1/MDM2/EP300 10

BP GO:0002576 platelet degranulation 7/227 129/18866 0.000961238 0.002531698 0.000956331 TGFB1/VEGFA/EGF/SOD1/APP/SYK/PECAM1 7

BP GO:0006081 cellular aldehyde metabolic process 5/227 63/18866 0.000961768 0.002531698 0.000956331 RELA/GLO1/ALDH3A1/TPI1/IDH1 5

BP GO:1902905 positive regulation of supramolecular fiber organization 9/227 208/18866 0.000962319 0.002531745 0.000956349 APP/EDN1/ICAM1/MTOR/CDC42/TRPV4/PSEN1/GPX1/TGFBR1 9

BP GO:0010614 negative regulation of cardiac muscle hypertrophy 4/227 37/18866 0.000987238 0.002591567 0.000978946 FOXO1/PPARA/TNFRSF1A/TNFRSF1B 4

BP GO:0043243 positive regulation of protein-containing complex disassembly 4/227 37/18866 0.000987238 0.002591567 0.000978946 TNF/IGF1R/INSR/TRPV4 4

BP GO:0090050 positive regulation of cell migration involved in sprouting angiogenesis 4/227 37/18866 0.000987238 0.002591567 0.000978946 PTGS2/VEGFA/KDR/HMOX1 4

BP GO:2000273 positive regulation of signaling receptor activity 4/227 37/18866 0.000987238 0.002591567 0.000978946 EGF/CCL2/CCR2/IFNG 4

BP GO:0061326 renal tubule development 6/227 95/18866 0.001012651 0.002656812 0.001003592 BCL2/VEGFA/STAT1/MYC/GDNF/SHH 6

BP GO:0006978 DNA damage response, signal transduction by p53 class mediator resulting in transcription of p21 class mediator 3/227 17/18866 0.001031955 0.002688129 0.001015422 CDKN1A/TP53/BRCA1 3

BP GO:0007635 chemosensory behavior 3/227 17/18866 0.001031955 0.002688129 0.001015422 NTRK1/PRKCG/TRPV1 3

BP GO:0010224 response to UV-B 3/227 17/18866 0.001031955 0.002688129 0.001015422 BCL2/RELA/CDKN1A 3

BP GO:0010715 regulation of extracellular matrix disassembly 3/227 17/18866 0.001031955 0.002688129 0.001015422 TGFB1/DPP4/IL6 3

BP GO:0015732 prostaglandin transport 3/227 17/18866 0.001031955 0.002688129 0.001015422 NOS2/IL1B/EDN1 3

BP GO:0030949 positive regulation of vascular endothelial growth factor receptor signaling pathway 3/227 17/18866 0.001031955 0.002688129 0.001015422 HIF1A/IL1B/PRKCB 3

BP GO:0032682 negative regulation of chemokine production 3/227 17/18866 0.001031955 0.002688129 0.001015422 IL10/GSTP1/NR1H4 3

BP GO:0032966 negative regulation of collagen biosynthetic process 3/227 17/18866 0.001031955 0.002688129 0.001015422 PPARG/IL6/PPARD 3

BP GO:0043117 positive regulation of vascular permeability 3/227 17/18866 0.001031955 0.002688129 0.001015422 TGFB1/VEGFA/TRPV4 3

BP GO:0051580 regulation of neurotransmitter uptake 3/227 17/18866 0.001031955 0.002688129 0.001015422 GDNF/SNCA/DRD2 3

BP GO:0090185 negative regulation of kidney development 3/227 17/18866 0.001031955 0.002688129 0.001015422 MMP9/STAT1/SHH 3

BP GO:2000811 negative regulation of anoikis 3/227 17/18866 0.001031955 0.002688129 0.001015422 BCL2/NTRK2/MCL1 3

BP GO:2001267 regulation of cysteine-type endopeptidase activity involved in apoptotic signaling pathway 3/227 17/18866 0.001031955 0.002688129 0.001015422 BAX/CASP8/MMP9 3

BP GO:0055002 striated muscle cell development 8/227 169/18866 0.001032504 0.002688129 0.001015422 BCL2/VEGFA/CDK1/EDN1/PPARA/CFLAR/MTOR/GPX1 8

BP GO:0051898 negative regulation of protein kinase B signaling 5/227 64/18866 0.001033193 0.00268845 0.001015543 AKT1/XDH/DDIT3/SIRT1/DRD2 5

BP GO:0042742 defense response to bacterium 12/227 348/18866 0.001084663 0.002820836 0.001065551 NOS2/IL10/TNF/IL6/TLR4/SYK/F2/MPO/CRP/C5AR1/NR1H4/TNFRSF1A 12

BP GO:0022604 regulation of cell morphogenesis 15/227 499/18866 0.001092716 0.002830324 0.001069135 MAP2/VEGFA/KDR/BDNF/NGF/NTRK2/CDK5/F2/ICAM1/CCL2/CDC42/PSEN1/ENPP2/CSF1R/RET 15

BP GO:0014912 negative regulation of smooth muscle cell migration 4/227 38/18866 0.001093074 0.002830324 0.001069135 PPARD/NFE2L2/GSTP1/PRKG1 4

BP GO:0032689 negative regulation of interferon-gamma production 4/227 38/18866 0.001093074 0.002830324 0.001069135 IL10/TLR4/DDIT3/NR1H4 4

BP GO:0048009 insulin-like growth factor receptor signaling pathway 4/227 38/18866 0.001093074 0.002830324 0.001069135 AR/AKT1/IGF1R/EIF2AK3 4

BP GO:0071604 transforming growth factor beta production 4/227 38/18866 0.001093074 0.002830324 0.001069135 PTGS2/HIF1A/CREB1/ATF2 4

BP GO:1901186 positive regulation of ERBB signaling pathway 4/227 38/18866 0.001093074 0.002830324 0.001069135 AKT1/MMP9/EGF/FASLG 4

BP GO:1903523 negative regulation of blood circulation 4/227 38/18866 0.001093074 0.002830324 0.001069135 PIK3CG/IL2/TRPV1/PDE5A 4

BP GO:2000144 positive regulation of DNA-templated transcription, initiation 4/227 38/18866 0.001093074 0.002830324 0.001069135 JUN/TP53/ESR1/CREB1 4

BP GO:0009206 purine ribonucleoside triphosphate biosynthetic process 5/227 65/18866 0.00110848 0.002860864 0.001080671 TGFB1/PARP1/STAT3/IL4/VCP 5

BP GO:0032729 positive regulation of interferon-gamma production 5/227 65/18866 0.00110848 0.002860864 0.001080671 TNF/IL1B/TLR4/CCR2/IL2 5

BP GO:0048645 animal organ formation 5/227 65/18866 0.00110848 0.002860864 0.001080671 AR/MAPK1/GDNF/MAPK3/SHH 5

BP GO:0060675 ureteric bud morphogenesis 5/227 65/18866 0.00110848 0.002860864 0.001080671 BCL2/VEGFA/MYC/GDNF/SHH 5

BP GO:0090181 regulation of cholesterol metabolic process 5/227 65/18866 0.00110848 0.002860864 0.001080671 SOD1/HMGCR/NR1H4/ABCG1/FDFT1 5

BP GO:0098586 cellular response to virus 5/227 65/18866 0.00110848 0.002860864 0.001080671 IL6/MMP12/MAPK14/BIRC3/BIRC2 5

BP GO:0006476 protein deacetylation 6/227 97/18866 0.001128749 0.002911597 0.001099835 VEGFA/TP53/SIRT1/MAPK8/IFNG/EP300 6

BP GO:0006360 transcription by RNA polymerase I 5/227 66/18866 0.001187756 0.003053863 0.001153575 MAPK3/CDK7/MTOR/ERBB2/EIF2AK3 5

BP GO:0009145 purine nucleoside triphosphate biosynthetic process 5/227 66/18866 0.001187756 0.003053863 0.001153575 TGFB1/PARP1/STAT3/IL4/VCP 5

BP GO:0032715 negative regulation of interleukin-6 production 5/227 66/18866 0.001187756 0.003053863 0.001153575 IL10/TNF/TLR4/PTPN6/NR1H4 5

BP GO:0046626 regulation of insulin receptor signaling pathway 5/227 66/18866 0.001187756 0.003053863 0.001153575 RELA/IL1B/SIRT1/PRKCB/NR1H4 5

BP GO:0072171 mesonephric tubule morphogenesis 5/227 66/18866 0.001187756 0.003053863 0.001153575 BCL2/VEGFA/MYC/GDNF/SHH 5

BP GO:1905207 regulation of cardiocyte differentiation 5/227 66/18866 0.001187756 0.003053863 0.001153575 TGFB1/EGFR/EDN1/PPARA/MTOR 5

BP GO:0006835 dicarboxylic acid transport 6/227 98/18866 0.001190491 0.003059241 0.001155607 BDNF/NTRK2/GJA1/PSEN1/TRPV1/HRH3 6

BP GO:0021543 pallium development 8/227 173/18866 0.001199953 0.003080224 0.001163533 BAX/CASP3/EGFR/HIF1A/NTRK2/CDK5/CDK6/PSEN1 8

BP GO:0050954 sensory perception of mechanical stimulus 8/227 173/18866 0.001199953 0.003080224 0.001163533 HTR2A/CASP3/SOD1/NTRK1/ICAM1/BIRC5/GPX1/TRPV1 8

BP GO:0010831 positive regulation of myotube differentiation 4/227 39/18866 0.001206621 0.003084021 0.001164967 BCL2/MAPK14/MTOR/IL4 4

BP GO:0014741 negative regulation of muscle hypertrophy 4/227 39/18866 0.001206621 0.003084021 0.001164967 FOXO1/PPARA/TNFRSF1A/TNFRSF1B 4

BP GO:0033146 regulation of intracellular estrogen receptor signaling pathway 4/227 39/18866 0.001206621 0.003084021 0.001164967 AR/ESR1/PARP1/BRCA1 4

BP GO:0042092 type 2 immune response 4/227 39/18866 0.001206621 0.003084021 0.001164967 IL10/IL6/CCR2/IL4 4

BP GO:0046326 positive regulation of glucose import 4/227 39/18866 0.001206621 0.003084021 0.001164967 AKT1/NFE2L2/MAPK14/INSR 4

BP GO:0055090 acylglycerol homeostasis 4/227 39/18866 0.001206621 0.003084021 0.001164967 SIRT1/NR1H3/NR1H4/CETP 4

BP GO:0070328 triglyceride homeostasis 4/227 39/18866 0.001206621 0.003084021 0.001164967 SIRT1/NR1H3/NR1H4/CETP 4

BP GO:2000279 negative regulation of DNA biosynthetic process 4/227 39/18866 0.001206621 0.003084021 0.001164967 PPARG/CDKN1A/TP53/GJA1 4

BP GO:0010713 negative regulation of collagen metabolic process 3/227 18/18866 0.001227404 0.003123704 0.001179957 PPARG/IL6/PPARD 3

BP GO:0032740 positive regulation of interleukin-17 production 3/227 18/18866 0.001227404 0.003123704 0.001179957 TGFB1/IL6/IL2 3

BP GO:0042136 neurotransmitter biosynthetic process 3/227 18/18866 0.001227404 0.003123704 0.001179957 SLC6A3/SLC6A4/ACHE 3

BP GO:0042772 DNA damage response, signal transduction resulting in transcription 3/227 18/18866 0.001227404 0.003123704 0.001179957 CDKN1A/TP53/BRCA1 3

BP GO:0043011 myeloid dendritic cell differentiation 3/227 18/18866 0.001227404 0.003123704 0.001179957 IL4/PSEN1/CSF2 3

BP GO:0050961 detection of temperature stimulus involved in sensory perception 3/227 18/18866 0.001227404 0.003123704 0.001179957 HTR2A/NTRK1/TRPV1 3

BP GO:0070230 positive regulation of lymphocyte apoptotic process 3/227 18/18866 0.001227404 0.003123704 0.001179957 BAX/IL10/TP53 3

BP GO:0071318 cellular response to ATP 3/227 18/18866 0.001227404 0.003123704 0.001179957 PTGS2/SOD1/TRPV1 3

BP GO:0015837 amine transport 6/227 99/18866 0.001254787 0.003188273 0.001204347 HTR2A/GDNF/SNCA/PSEN1/HRH3/DRD2 6

BP GO:0062207 regulation of pattern recognition receptor signaling pathway 6/227 99/18866 0.001254787 0.003188273 0.001204347 ESR1/TLR4/XIAP/BIRC3/NR1H3/BIRC2 6

BP GO:0090277 positive regulation of peptide hormone secretion 6/227 99/18866 0.001254787 0.003188273 0.001204347 EGFR/HIF1A/PPARD/GJA1/NR1H4/DRD2 6

BP GO:0072678 T cell migration 5/227 67/18866 0.00127115 0.003228124 0.001219401 PIK3CG/APP/ICAM1/CCL2/CCR2 5

BP GO:0007269 neurotransmitter secretion 8/227 175/18866 0.001291433 0.003276134 0.001237536 HTR2A/SNCA/CDK5/PRKCB/PRKCG/PSEN1/HRH3/DRD2 8

BP GO:0099643 signal release from synapse 8/227 175/18866 0.001291433 0.003276134 0.001237536 HTR2A/SNCA/CDK5/PRKCB/PRKCG/PSEN1/HRH3/DRD2 8

BP GO:0045069 regulation of viral genome replication 6/227 100/18866 0.001321705 0.00335114 0.001265869 BCL2/TNF/CXCL8/CD28/EIF2AK4/EIF2AK2 6

BP GO:0010939 regulation of necrotic cell death 4/227 40/18866 0.001328174 0.003360372 0.001269356 CASP8/CFLAR/BIRC3/BIRC2 4

BP GO:0042417 dopamine metabolic process 4/227 40/18866 0.001328174 0.003360372 0.001269356 SLC6A3/SNCA/HTR1A/DRD2 4

BP GO:0048713 regulation of oligodendrocyte differentiation 4/227 40/18866 0.001328174 0.003360372 0.001269356 PPARG/MTOR/SHH/TNFRSF1B 4

BP GO:0051281 positive regulation of release of sequestered calcium ion into cytosol 4/227 40/18866 0.001328174 0.003360372 0.001269356 BAX/SNCA/F2/BDKRB1 4

BP GO:0030534 adult behavior 7/227 137/18866 0.00136456 0.003448761 0.001302745 HTR2A/OPRM1/GDNF/APP/CDK5/PPARA/DRD2 7

BP GO:0042552 myelination 7/227 137/18866 0.00136456 0.003448761 0.001302745 AKT1/SOD1/NTRK2/MTOR/ERBB2/TNFRSF1B/EIF2AK3 7

BP GO:0006638 neutral lipid metabolic process 7/227 138/18866 0.001423069 0.003592815 0.00135716 PIK3CG/CAT/SIRT1/NR1H3/GPX1/PLA2G4A/CETP 7

BP GO:0006639 acylglycerol metabolic process 7/227 138/18866 0.001423069 0.003592815 0.00135716 PIK3CG/CAT/SIRT1/NR1H3/GPX1/PLA2G4A/CETP 7

BP GO:0007213 G protein-coupled acetylcholine receptor signaling pathway 3/227 19/18866 0.001444671 0.003624257 0.001369037 CHRM3/OPRM1/HRH3 3

BP GO:0019054 modulation by virus of host cellular process 3/227 19/18866 0.001444671 0.003624257 0.001369037 CASP8/BCL2L1/EIF2AK4 3

BP GO:0033189 response to vitamin A 3/227 19/18866 0.001444671 0.003624257 0.001369037 PPARG/PPARD/CAT 3

BP GO:0034393 positive regulation of smooth muscle cell apoptotic process 3/227 19/18866 0.001444671 0.003624257 0.001369037 PPARG/SOD2/IFNG 3

BP GO:0043373 CD4-positive, alpha-beta T cell lineage commitment 3/227 19/18866 0.001444671 0.003624257 0.001369037 IL6/STAT3/MTOR 3

BP GO:0051546 keratinocyte migration 3/227 19/18866 0.001444671 0.003624257 0.001369037 MMP9/PPARD/MTOR 3

BP GO:0060438 trachea development 3/227 19/18866 0.001444671 0.003624257 0.001369037 MAPK1/MAPK3/SHH 3

BP GO:0071800 podosome assembly 3/227 19/18866 0.001444671 0.003624257 0.001369037 TNF/CSF2/MAPK9 3

BP GO:0072074 kidney mesenchyme development 3/227 19/18866 0.001444671 0.003624257 0.001369037 STAT1/MYC/SHH 3

BP GO:1902170 cellular response to reactive nitrogen species 3/227 19/18866 0.001444671 0.003624257 0.001369037 MMP3/FOXO1/CFLAR 3

BP GO:2000696 regulation of epithelial cell differentiation involved in kidney development 3/227 19/18866 0.001444671 0.003624257 0.001369037 MMP9/STAT1/GDNF 3

BP GO:2000757 negative regulation of peptidyl-lysine acetylation 3/227 19/18866 0.001444671 0.003624257 0.001369037 SNCA/SIRT1/BRCA1 3

BP GO:0046825 regulation of protein export from nucleus 4/227 41/18866 0.001458025 0.003651977 0.001379508 TP53/IL1B/CDK5/MDM2 4

BP GO:1900117 regulation of execution phase of apoptosis 4/227 41/18866 0.001458025 0.003651977 0.001379508 BAX/BCL2L1/IL6/TP53 4

BP GO:2000008 regulation of protein localization to cell surface 4/227 41/18866 0.001458025 0.003651977 0.001379508 AKT1/EGF/TNF/BDNF 4

BP GO:0022600 digestive system process 6/227 102/18866 0.00146369 0.003664235 0.001384139 CHRM3/TLR4/NR1H3/NPC1L1/TRPV1/VDR 6

BP GO:0006885 regulation of pH 6/227 103/18866 0.001538898 0.003848459 0.001453728 BCL2/MAPK1/MAPK3/CA2/FASLG/EDN1 6

BP GO:0070498 interleukin-1-mediated signaling pathway 6/227 103/18866 0.001538898 0.003848459 0.001453728 RELA/IL6/IL1B/MAPK3/IL1A/NFKB1 6

BP GO:0009268 response to pH 4/227 42/18866 0.001596469 0.003988236 0.001506527 CA2/GJA1/PAM/TRPV1 4

BP GO:1902622 regulation of neutrophil migration 4/227 42/18866 0.001596469 0.003988236 0.001506527 DPP4/EDN1/CXCL8/C5AR1 4

BP GO:0001889 liver development 7/227 141/18866 0.001610418 0.00402097 0.001518893 JUN/RELA/EGFR/IL10/IL6/HMOX1/CFLAR 7

BP GO:0002456 T cell mediated immunity 6/227 104/18866 0.001617012 0.004033198 0.001523512 IL6/IL1B/ICAM1/CCR2/IL4/TNFRSF1B 6

BP GO:0048525 negative regulation of viral process 6/227 104/18866 0.001617012 0.004033198 0.001523512 JUN/TNF/STAT1/CRP/EIF2AK4/EIF2AK2 6

BP GO:0009201 ribonucleoside triphosphate biosynthetic process 5/227 71/18866 0.001648511 0.004107454 0.001551561 TGFB1/PARP1/STAT3/IL4/VCP 5

BP GO:0045739 positive regulation of DNA repair 5/227 71/18866 0.001648511 0.004107454 0.001551561 EGFR/PARP1/SIRT1/BRCA1/PRKCG 5

BP GO:1901988 negative regulation of cell cycle phase transition 10/227 270/18866 0.001654245 0.004119582 0.001556143 BCL2/BAX/CDKN1A/TP53/CDK1/CDK6/CCL2/BRCA1/MDM2/EP300 10

BP GO:0045665 negative regulation of neuron differentiation 9/227 225/18866 0.001660726 0.004133555 0.001561421 SLC6A4/MAP2/APP/CDK5/MDM2/TRPV4/PSEN1/SHH/EIF2AK4 9

BP GO:0002689 negative regulation of leukocyte chemotaxis 3/227 20/18866 0.001684613 0.004171173 0.001575631 DPP4/CYP19A1/CCL2 3

BP GO:0002902 regulation of B cell apoptotic process 3/227 20/18866 0.001684613 0.004171173 0.001575631 BAX/IL10/IL2 3

BP GO:0007252 I-kappaB phosphorylation 3/227 20/18866 0.001684613 0.004171173 0.001575631 AKT1/TNF/TLR4 3

BP GO:0031998 regulation of fatty acid beta-oxidation 3/227 20/18866 0.001684613 0.004171173 0.001575631 AKT1/PPARA/MTOR 3

BP GO:0032303 regulation of icosanoid secretion 3/227 20/18866 0.001684613 0.004171173 0.001575631 IL1B/SYK/EDN1 3

BP GO:0042089 cytokine biosynthetic process 3/227 20/18866 0.001684613 0.004171173 0.001575631 SYK/CD80/CD28 3

BP GO:0071243 cellular response to arsenic-containing substance 3/227 20/18866 0.001684613 0.004171173 0.001575631 HMOX1/ATF3/VCP 3

BP GO:0071636 positive regulation of transforming growth factor beta production 3/227 20/18866 0.001684613 0.004171173 0.001575631 PTGS2/CREB1/ATF2 3

BP GO:0071731 response to nitric oxide 3/227 20/18866 0.001684613 0.004171173 0.001575631 MMP3/FOXO1/CFLAR 3

BP GO:0097709 connective tissue replacement 3/227 20/18866 0.001684613 0.004171173 0.001575631 TGFB1/HIF1A/IL1A 3

BP GO:0097006 regulation of plasma lipoprotein particle levels 6/227 105/18866 0.001698105 0.004200203 0.001586597 HMOX1/MPO/AGTR1/NR1H4/CETP/ABCG1 6

BP GO:2001022 positive regulation of response to DNA damage stimulus 6/227 105/18866 0.001698105 0.004200203 0.001586597 EGFR/MYC/PARP1/SIRT1/BRCA1/PRKCG 6

BP GO:0043433 negative regulation of DNA-binding transcription factor activity 8/227 183/18866 0.001714376 0.004236042 0.001600134 IL10/ESR1/HMOX1/DDIT3/CAT/SIRT1/NR1H4/EIF2AK4 8

BP GO:0055001 muscle cell development 8/227 183/18866 0.001714376 0.004236042 0.001600134 BCL2/VEGFA/CDK1/EDN1/PPARA/CFLAR/MTOR/GPX1 8

BP GO:0040019 positive regulation of embryonic development 4/227 43/18866 0.001743795 0.004304259 0.001625903 AR/GDNF/GJA1/SHH 4

BP GO:1901031 regulation of response to reactive oxygen species 4/227 43/18866 0.001743795 0.004304259 0.001625903 IL10/TNF/NFE2L2/DHFR 4

BP GO:1904427 positive regulation of calcium ion transmembrane transport 5/227 72/18866 0.001754455 0.004328323 0.001634993 BAX/SNCA/CDK5/F2/BDKRB1 5

BP GO:0007416 synapse assembly 8/227 184/18866 0.001774147 0.004374634 0.001652487 ACHE/SNCA/BDNF/NTRK1/NTRK2/APP/CDK5/DRD2 8

BP GO:0019395 fatty acid oxidation 6/227 106/18866 0.001782249 0.004390058 0.001658313 PPARG/AKT1/PPARD/MAPK14/PPARA/MTOR 6

BP GO:1905269 positive regulation of chromatin organization 6/227 106/18866 0.001782249 0.004390058 0.001658313 VEGFA/TP53/IL1B/MAPK3/SIRT1/BRCA1 6

BP GO:0006720 isoprenoid metabolic process 7/227 144/18866 0.001816435 0.004469635 0.001688373 EGFR/PPARD/AKR1B1/TTR/CYP3A4/HMGCR/FDFT1 7

BP GO:0061008 hepaticobiliary system development 7/227 144/18866 0.001816435 0.004469635 0.001688373 JUN/RELA/EGFR/IL10/IL6/HMOX1/CFLAR 7

BP GO:0072078 nephron tubule morphogenesis 5/227 73/18866 0.001865305 0.004587512 0.0017329 BCL2/VEGFA/MYC/GDNF/SHH 5

BP GO:0000018 regulation of DNA recombination 6/227 107/18866 0.001869519 0.004593123 0.001735019 TGFB1/IL10/PARP1/CD28/IL2/IL4 6

BP GO:0035601 protein deacylation 6/227 107/18866 0.001869519 0.004593123 0.001735019 VEGFA/TP53/SIRT1/MAPK8/IFNG/EP300 6

BP GO:0071322 cellular response to carbohydrate stimulus 7/227 145/18866 0.001889468 0.004639738 0.001752628 HIF1A/GCLC/IGF1R/ICAM1/PRKCB/HMGCR/NR1H4 7

BP GO:0050770 regulation of axonogenesis 8/227 186/18866 0.001898625 0.004656702 0.001759036 MAP2/VEGFA/BDNF/NGF/NTRK2/CDK5/PSEN1/RET 8

BP GO:0010463 mesenchymal cell proliferation 4/227 44/18866 0.001900295 0.004656702 0.001759036 VEGFA/STAT1/MYC/SHH 4

BP GO:0044060 regulation of endocrine process 4/227 44/18866 0.001900295 0.004656702 0.001759036 IL1B/GJA1/AGTR1/REN 4

BP GO:0060612 adipose tissue development 4/227 44/18866 0.001900295 0.004656702 0.001759036 PPARD/SIRT1/ATF2/NR1H4 4

BP GO:0007160 cell-matrix adhesion 9/227 230/18866 0.001929527 0.004720191 0.001783018 PLAU/BCL2/VEGFA/KDR/MMP12/CDK5/CDK6/VCAM1/PECAM1 9

BP GO:1902903 regulation of supramolecular fiber organization 12/227 373/18866 0.001945364 0.004720191 0.001783018 CHRNA7/MAP2/SNCA/APP/EDN1/ICAM1/MTOR/CDC42/TRPV4/PSEN1/GPX1/TGFBR1 12

BP GO:0002693 positive regulation of cellular extravasation 3/227 21/18866 0.001948047 0.004720191 0.001783018 ICAM1/SELE/CCR2 3

BP GO:0010310 regulation of hydrogen peroxide metabolic process 3/227 21/18866 0.001948047 0.004720191 0.001783018 MMP3/SNCA/STAT3 3

BP GO:0010829 negative regulation of glucose transmembrane transport 3/227 21/18866 0.001948047 0.004720191 0.001783018 TNF/IL1B/PRKCB 3

BP GO:0016540 protein autoprocessing 3/227 21/18866 0.001948047 0.004720191 0.001783018 PARP1/CASP1/SHH 3

BP GO:0019370 leukotriene biosynthetic process 3/227 21/18866 0.001948047 0.004720191 0.001783018 SYK/ALOX5/PLA2G4A 3

BP GO:0030728 ovulation 3/227 21/18866 0.001948047 0.004720191 0.001783018 PGR/PTGS2/SIRT1 3

BP GO:0030878 thyroid gland development 3/227 21/18866 0.001948047 0.004720191 0.001783018 MAPK1/MAPK3/SHH 3

BP GO:0033630 positive regulation of cell adhesion mediated by integrin 3/227 21/18866 0.001948047 0.004720191 0.001783018 SYK/PTPN6/RET 3

BP GO:0035357 peroxisome proliferator activated receptor signaling pathway 3/227 21/18866 0.001948047 0.004720191 0.001783018 RXRA/PPARG/SIRT1 3

BP GO:0035813 regulation of renal sodium excretion 3/227 21/18866 0.001948047 0.004720191 0.001783018 EDN1/AGTR1/DRD2 3

BP GO:0042107 cytokine metabolic process 3/227 21/18866 0.001948047 0.004720191 0.001783018 SYK/CD80/CD28 3

BP GO:0045943 positive regulation of transcription by RNA polymerase I 3/227 21/18866 0.001948047 0.004720191 0.001783018 MTOR/ERBB2/EIF2AK3 3

BP GO:0046641 positive regulation of alpha-beta T cell proliferation 3/227 21/18866 0.001948047 0.004720191 0.001783018 SYK/CCR2/CD28 3

BP GO:0046827 positive regulation of protein export from nucleus 3/227 21/18866 0.001948047 0.004720191 0.001783018 TP53/IL1B/MDM2 3

BP GO:0071498 cellular response to fluid shear stress 3/227 21/18866 0.001948047 0.004720191 0.001783018 PTGS2/NFE2L2/CA2 3

BP GO:0090201 negative regulation of release of cytochrome c from mitochondria 3/227 21/18866 0.001948047 0.004720191 0.001783018 AKT1/BCL2L1/GPX1 3

BP GO:0090312 positive regulation of protein deacetylation 3/227 21/18866 0.001948047 0.004720191 0.001783018 VEGFA/TP53/IFNG 3

BP GO:0090330 regulation of platelet aggregation 3/227 21/18866 0.001948047 0.004720191 0.001783018 PRKCA/SYK/PRKG1 3

BP GO:1900543 negative regulation of purine nucleotide metabolic process 3/227 21/18866 0.001948047 0.004720191 0.001783018 PARP1/STAT3/PPARA 3

BP GO:1903055 positive regulation of extracellular matrix organization 3/227 21/18866 0.001948047 0.004720191 0.001783018 TGFB1/IL6/CFLAR 3

BP GO:0021536 diencephalon development 5/227 74/18866 0.001981197 0.004793185 0.001810591 SLC6A3/BAX/CREB1/SHH/DRD2 5

BP GO:0034121 regulation of toll-like receptor signaling pathway 5/227 74/18866 0.001981197 0.004793185 0.001810591 ESR1/TLR4/BIRC3/NR1H3/BIRC2 5

BP GO:1903036 positive regulation of response to wounding 5/227 74/18866 0.001981197 0.004793185 0.001810591 NFE2L2/TLR4/F2/MTOR/PLA2G4A 5

BP GO:0010828 positive regulation of glucose transmembrane transport 4/227 45/18866 0.002066256 0.00499643 0.001887366 AKT1/NFE2L2/MAPK14/INSR 4

BP GO:0006695 cholesterol biosynthetic process 5/227 75/18866 0.002102263 0.005068032 0.001914413 SOD1/HMGCR/NPC1L1/ABCG1/FDFT1 5

BP GO:0015909 long-chain fatty acid transport 5/227 75/18866 0.002102263 0.005068032 0.001914413 PPARG/AKT1/SYK/PLA2G4A/DRD2 5

BP GO:0046323 glucose import 5/227 75/18866 0.002102263 0.005068032 0.001914413 AKT1/TNF/NFE2L2/MAPK14/INSR 5

BP GO:1900076 regulation of cellular response to insulin stimulus 5/227 75/18866 0.002102263 0.005068032 0.001914413 RELA/IL1B/SIRT1/PRKCB/NR1H4 5

BP GO:1901983 regulation of protein acetylation 5/227 75/18866 0.002102263 0.005068032 0.001914413 IL1B/SNCA/MAPK3/SIRT1/BRCA1 5

BP GO:1902653 secondary alcohol biosynthetic process 5/227 75/18866 0.002102263 0.005068032 0.001914413 SOD1/HMGCR/NPC1L1/ABCG1/FDFT1 5

BP GO:0007188 adenylate cyclase-modulating G protein-coupled receptor signaling pathway 9/227 233/18866 0.002106805 0.005076408 0.001917577 CHRM3/OPRM1/PRKCA/EDN1/GABBR1/HRH3/CCR3/HTR1A/DRD2 9

BP GO:0006641 triglyceride metabolic process 6/227 110/18866 0.002150823 0.005179844 0.001956649 PIK3CG/CAT/SIRT1/NR1H3/GPX1/CETP 6

BP GO:0010498 proteasomal protein catabolic process 14/227 483/18866 0.002225748 0.005357574 0.002023785 AKT1/NFE2L2/DDIT3/GCLC/CDK1/SIRT1/MDM2/VCP/PRKCG/PSEN1/SHH/GPX1/BIRC2/MAPK9 14

BP GO:0032700 negative regulation of interleukin-17 production 3/227 22/18866 0.002235749 0.005365346 0.002026721 TLR4/DDIT3/IFNG 3

BP GO:0035809 regulation of urine volume 3/227 22/18866 0.002235749 0.005365346 0.002026721 AKR1B1/EDN1/DRD2 3

BP GO:0045655 regulation of monocyte differentiation 3/227 22/18866 0.002235749 0.005365346 0.002026721 JUN/MYC/CDK6 3

BP GO:0045980 negative regulation of nucleotide metabolic process 3/227 22/18866 0.002235749 0.005365346 0.002026721 PARP1/STAT3/PPARA 3

BP GO:0051152 positive regulation of smooth muscle cell differentiation 3/227 22/18866 0.002235749 0.005365346 0.002026721 SIRT1/SOD2/SHH 3

BP GO:1901739 regulation of myoblast fusion 3/227 22/18866 0.002235749 0.005365346 0.002026721 MAPK14/CFLAR/IL4 3

BP GO:0031641 regulation of myelination 4/227 46/18866 0.002241965 0.005369421 0.00202826 AKT1/MTOR/TNFRSF1B/EIF2AK3 4

BP GO:0032570 response to progesterone 4/227 46/18866 0.002241965 0.005369421 0.00202826 TGFB1/RELA/FOS/NR1H3 4

BP GO:0045581 negative regulation of T cell differentiation 4/227 46/18866 0.002241965 0.005369421 0.00202826 ERBB2/IL2/IL4/SHH 4

BP GO:2000142 regulation of DNA-templated transcription, initiation 4/227 46/18866 0.002241965 0.005369421 0.00202826 JUN/TP53/ESR1/CREB1 4

BP GO:0042303 molting cycle 6/227 111/18866 0.00225134 0.005383738 0.002033669 PTGS2/BCL2/RELA/EGFR/TNF/SHH 6

BP GO:0042633 hair cycle 6/227 111/18866 0.00225134 0.005383738 0.002033669 PTGS2/BCL2/RELA/EGFR/TNF/SHH 6

BP GO:0098732 macromolecule deacylation 6/227 111/18866 0.00225134 0.005383738 0.002033669 VEGFA/TP53/SIRT1/MAPK8/IFNG/EP300 6

BP GO:0035148 tube formation 7/227 150/18866 0.002289432 0.005472077 0.002067038 CASP3/TGFB1/VEGFA/HIF1A/GDNF/SHH/RET 7

BP GO:0006413 translational initiation 8/227 192/18866 0.002313844 0.00552487 0.00208698 RXRA/TNF/MTOR/EIF2S1/EIF2AK3/EIF2AK4/EIF2AK2/EIF2AK1 8

BP GO:0021953 central nervous system neuron differentiation 8/227 192/18866 0.002313844 0.00552487 0.00208698 MAP2/NTRK2/HSP90AA1/CDK5/PSEN1/SHH/CSF1R/DRD2 8

BP GO:0006275 regulation of DNA replication 6/227 112/18866 0.002355359 0.005621172 0.002123358 JUN/EGFR/EGF/TP53/CDK1/CDC42 6

BP GO:0061333 renal tubule morphogenesis 5/227 77/18866 0.00236046 0.005627697 0.002125822 BCL2/VEGFA/MYC/GDNF/SHH 5

BP GO:0061418 regulation of transcription from RNA polymerase II promoter in response to hypoxia 5/227 77/18866 0.00236046 0.005627697 0.002125822 VEGFA/TP53/HIF1A/NFE2L2/EP300 5

BP GO:0016331 morphogenesis of embryonic epithelium 7/227 151/18866 0.002376717 0.005663616 0.00213939 CASP3/TGFB1/AR/HIF1A/GDNF/SHH/RET 7

BP GO:0014075 response to amine 4/227 47/18866 0.002427705 0.00576777 0.002178734 SOD1/CDK1/ICAM1/DRD2 4

BP GO:0042551 neuron maturation 4/227 47/18866 0.002427705 0.00576777 0.002178734 BCL2/APP/MTOR/RET 4

BP GO:0043300 regulation of leukocyte degranulation 4/227 47/18866 0.002427705 0.00576777 0.002178734 HMOX1/SYK/CCR2/IL4 4

BP GO:0043303 mast cell degranulation 4/227 47/18866 0.002427705 0.00576777 0.002178734 PIK3CG/HMOX1/SYK/IL4 4

BP GO:0046638 positive regulation of alpha-beta T cell differentiation 4/227 47/18866 0.002427705 0.00576777 0.002178734 SYK/CD80/IFNG/SHH 4

BP GO:0060986 endocrine hormone secretion 4/227 47/18866 0.002427705 0.00576777 0.002178734 IL1B/GJA1/AGTR1/REN 4

BP GO:0034249 negative regulation of cellular amide metabolic process 9/227 238/18866 0.00243094 0.005772572 0.002180548 CHRNA7/NTRK2/STAT3/EIF2S1/EIF2AK3/DHFR/EIF2AK4/EIF2AK2/EIF2AK1 9

BP GO:1903510 mucopolysaccharide metabolic process 6/227 113/18866 0.002462955 0.005842758 0.00220706 TGFB1/AKT1/EGF/IL1B/NFKB1/GLB1 6

BP GO:1903555 regulation of tumor necrosis factor superfamily cytokine production 6/227 113/18866 0.002462955 0.005842758 0.00220706 CHRNA7/IL10/TLR4/IFNG/GSTP1/PTPN6 6

BP GO:2000181 negative regulation of blood vessel morphogenesis 8/227 194/18866 0.002467043 0.005849538 0.002209621 PPARG/TNF/XDH/STAT1/FASLG/CCR2/ATF2/ALOX5 8

BP GO:0060395 SMAD protein signal transduction 5/227 78/18866 0.002497861 0.005919657 0.002236108 JUN/TGFB1/FOS/PARP1/TGFBR1 5

BP GO:1902275 regulation of chromatin organization 8/227 195/18866 0.002546565 0.005991779 0.002263352 VEGFA/TP53/IL1B/SNCA/MAPK3/SIRT1/MAPK8/BRCA1 8

BP GO:0001919 regulation of receptor recycling 3/227 23/18866 0.00254846 0.005991779 0.002263352 ACHE/SNCA/PSEN1 3

BP GO:0006359 regulation of transcription by RNA polymerase III 3/227 23/18866 0.00254846 0.005991779 0.002263352 AR/BRCA1/MTOR 3

BP GO:0010288 response to lead ion 3/227 23/18866 0.00254846 0.005991779 0.002263352 PTGS2/APP/CAT 3

BP GO:0016048 detection of temperature stimulus 3/227 23/18866 0.00254846 0.005991779 0.002263352 HTR2A/NTRK1/TRPV1 3

BP GO:0032098 regulation of appetite 3/227 23/18866 0.00254846 0.005991779 0.002263352 OPRM1/HTR2C/PPARA 3

BP GO:0035162 embryonic hemopoiesis 3/227 23/18866 0.00254846 0.005991779 0.002263352 VEGFA/HIF1A/KDR 3

BP GO:0035743 CD4-positive, alpha-beta T cell cytokine production 3/227 23/18866 0.00254846 0.005991779 0.002263352 IL6/IL1B/IL4 3

BP GO:0035812 renal sodium excretion 3/227 23/18866 0.00254846 0.005991779 0.002263352 EDN1/AGTR1/DRD2 3

BP GO:0045649 regulation of macrophage differentiation 3/227 23/18866 0.00254846 0.005991779 0.002263352 CASP8/PRKCA/TGFB1 3

BP GO:0045723 positive regulation of fatty acid biosynthetic process 3/227 23/18866 0.00254846 0.005991779 0.002263352 PTGS2/IL1B/NR1H3 3

BP GO:0050951 sensory perception of temperature stimulus 3/227 23/18866 0.00254846 0.005991779 0.002263352 HTR2A/NTRK1/TRPV1 3

BP GO:1901984 negative regulation of protein acetylation 3/227 23/18866 0.00254846 0.005991779 0.002263352 SNCA/SIRT1/BRCA1 3

BP GO:1902254 negative regulation of intrinsic apoptotic signaling pathway by p53 class mediator 3/227 23/18866 0.00254846 0.005991779 0.002263352 BCL2/SIRT1/MDM2 3

BP GO:1903589 positive regulation of blood vessel endothelial cell proliferation involved in sprouting angiogenesis 3/227 23/18866 0.00254846 0.005991779 0.002263352 VEGFA/HMOX1/AGTR1 3

BP GO:2000178 negative regulation of neural precursor cell proliferation 3/227 23/18866 0.00254846 0.005991779 0.002263352 SLC6A4/TP53/BDNF 3

BP GO:0030004 cellular monovalent inorganic cation homeostasis 6/227 114/18866 0.002574205 0.006043344 0.00228283 BCL2/MAPK1/MAPK3/CA2/FASLG/KCNMA1 6

BP GO:0032411 positive regulation of transporter activity 6/227 114/18866 0.002574205 0.006043344 0.00228283 PON1/CDK5/ABCB1/CCL2/CCR2/IFNG 6

BP GO:0098693 regulation of synaptic vesicle cycle 6/227 114/18866 0.002574205 0.006043344 0.00228283 HTR2A/SNCA/CDK5/PRKCB/PRKCG/DRD2 6

BP GO:0002279 mast cell activation involved in immune response 4/227 48/18866 0.002623758 0.006147533 0.002322187 PIK3CG/HMOX1/SYK/IL4 4

BP GO:0010799 regulation of peptidyl-threonine phosphorylation 4/227 48/18866 0.002623758 0.006147533 0.002322187 TGFB1/MAPK1/EGF/APP 4

BP GO:0043370 regulation of CD4-positive, alpha-beta T cell differentiation 4/227 48/18866 0.002623758 0.006147533 0.002322187 CD80/IL2/IFNG/IL4 4

BP GO:0060324 face development 4/227 48/18866 0.002623758 0.006147533 0.002322187 MMP2/MAPK1/MAPK3/EP300 4

BP GO:0009205 purine ribonucleoside triphosphate metabolic process 5/227 79/18866 0.002640978 0.006178745 0.002333977 TGFB1/PARP1/STAT3/IL4/VCP 5

BP GO:0021675 nerve development 5/227 79/18866 0.002640978 0.006178745 0.002333977 BDNF/NGF/NTRK1/PRKCG/RET 5

BP GO:0090398 cellular senescence 5/227 79/18866 0.002640978 0.006178745 0.002333977 CDKN1A/TP53/CDK6/MAPK14/SIRT1 5

BP GO:0048640 negative regulation of developmental growth 6/227 115/18866 0.002689188 0.00628844 0.002375413 SLC6A4/MAP2/CDKN1A/CDK5/GJA1/PPARA 6

BP GO:0060070 canonical Wnt signaling pathway 11/227 339/18866 0.002781151 0.006500291 0.002455438 TGFB1/EGFR/EGF/DDIT3/MAPK14/FOXO1/XIAP/NFKB1/VCP/PSEN1/SHH 11

BP GO:2000300 regulation of synaptic vesicle exocytosis 5/227 80/18866 0.002789947 0.006517645 0.002461994 HTR2A/CDK5/PRKCB/PRKCG/DRD2 5

BP GO:0032640 tumor necrosis factor production 6/227 116/18866 0.00280798 0.00655655 0.00247669 CHRNA7/IL10/TLR4/APP/GSTP1/PTPN6 6

BP GO:0002448 mast cell mediated immunity 4/227 49/18866 0.002830403 0.006583041 0.002486697 PIK3CG/HMOX1/SYK/IL4 4

BP GO:0003254 regulation of membrane depolarization 4/227 49/18866 0.002830403 0.006583041 0.002486697 BCL2/KDR/GCLC/PARP1 4

BP GO:0042059 negative regulation of epidermal growth factor receptor signaling pathway 4/227 49/18866 0.002830403 0.006583041 0.002486697 EGFR/EGF/CDC42/PSEN1 4

BP GO:0042149 cellular response to glucose starvation 4/227 49/18866 0.002830403 0.006583041 0.002486697 BCL2/TP53/NFE2L2/EIF2AK3 4

BP GO:0042398 cellular modified amino acid biosynthetic process 4/227 49/18866 0.002830403 0.006583041 0.002486697 NFE2L2/GCLC/GAMT/DHFR 4

BP GO:0048512 circadian behavior 4/227 49/18866 0.002830403 0.006583041 0.002486697 TP53/MTOR/CSF2/DRD2 4

BP GO:0048641 regulation of skeletal muscle tissue development 4/227 49/18866 0.002830403 0.006583041 0.002486697 BCL2/TGFB1/HMGCR/SHH 4

BP GO:0060711 labyrinthine layer development 4/227 49/18866 0.002830403 0.006583041 0.002486697 CASP8/AKT1/MAPK1/IL10 4

BP GO:0032753 positive regulation of interleukin-4 production 3/227 24/18866 0.002886881 0.006691483 0.00252766 SYK/CD28/CD40LG 3

BP GO:0034695 response to prostaglandin E 3/227 24/18866 0.002886881 0.006691483 0.00252766 PPARG/AKT1/GNRH1 3

BP GO:0044062 regulation of excretion 3/227 24/18866 0.002886881 0.006691483 0.00252766 EDN1/AGTR1/DRD2 3

BP GO:0044321 response to leptin 3/227 24/18866 0.002886881 0.006691483 0.00252766 SIRT1/STAT3/EDN1 3

BP GO:0097186 amelogenesis 3/227 24/18866 0.002886881 0.006691483 0.00252766 FOXO1/PPARA/ATF2 3

BP GO:1903429 regulation of cell maturation 3/227 24/18866 0.002886881 0.006691483 0.00252766 BCL2/MTOR/RET 3

BP GO:2000209 regulation of anoikis 3/227 24/18866 0.002886881 0.006691483 0.00252766 BCL2/NTRK2/MCL1 3

BP GO:0030282 bone mineralization 6/227 117/18866 0.00293066 0.006789647 0.002564741 PTGS2/TGFB1/HIF1A/ALOX5/CCR1/EIF2AK3 6

BP GO:0016126 sterol biosynthetic process 5/227 81/18866 0.002944903 0.006816002 0.002574696 SOD1/HMGCR/NPC1L1/ABCG1/FDFT1 5

BP GO:0051279 regulation of release of sequestered calcium ion into cytosol 5/227 81/18866 0.002944903 0.006816002 0.002574696 BAX/SNCA/F2/PTPN6/BDKRB1 5

BP GO:0001961 positive regulation of cytokine-mediated signaling pathway 4/227 50/18866 0.003047916 0.007027058 0.002654421 HIF1A/MMP12/EDN1/CASP1 4

BP GO:0003044 regulation of systemic arterial blood pressure mediated by a chemical signal 4/227 50/18866 0.003047916 0.007027058 0.002654421 EDN1/SOD2/AGTR1/REN 4

BP GO:0007622 rhythmic behavior 4/227 50/18866 0.003047916 0.007027058 0.002654421 TP53/MTOR/CSF2/DRD2 4

BP GO:0031648 protein destabilization 4/227 50/18866 0.003047916 0.007027058 0.002654421 SNCA/SIRT1/MDM2/EP300 4

BP GO:0045540 regulation of cholesterol biosynthetic process 4/227 50/18866 0.003047916 0.007027058 0.002654421 SOD1/HMGCR/ABCG1/FDFT1 4

BP GO:0048546 digestive tract morphogenesis 4/227 50/18866 0.003047916 0.007027058 0.002654421 BCL2/EGFR/HIF1A/SHH 4

BP GO:0106118 regulation of sterol biosynthetic process 4/227 50/18866 0.003047916 0.007027058 0.002654421 SOD1/HMGCR/ABCG1/FDFT1 4

BP GO:2000725 regulation of cardiac muscle cell differentiation 4/227 50/18866 0.003047916 0.007027058 0.002654421 TGFB1/EDN1/PPARA/MTOR 4

BP GO:0021987 cerebral cortex development 6/227 118/18866 0.003057306 0.00704529 0.002661308 BAX/EGFR/HIF1A/NTRK2/CDK5/PSEN1 6

BP GO:0030203 glycosaminoglycan metabolic process 7/227 158/18866 0.003061684 0.007051961 0.002663828 TGFB1/AKT1/EGF/IL1B/FUCA1/NFKB1/GLB1 7

BP GO:0009142 nucleoside triphosphate biosynthetic process 5/227 82/18866 0.003105982 0.007147066 0.002699753 TGFB1/PARP1/STAT3/IL4/VCP 5

BP GO:0021954 central nervous system neuron development 5/227 82/18866 0.003105982 0.007147066 0.002699753 MAP2/NTRK2/HSP90AA1/CDK5/DRD2 5

BP GO:0016573 histone acetylation 7/227 159/18866 0.003170702 0.007292461 0.002754675 IL1B/SNCA/MAPK3/SIRT1/BRCA1/ATF2/EP300 7

BP GO:0003071 renal system process involved in regulation of systemic arterial blood pressure 3/227 25/18866 0.003251678 0.007439114 0.002810072 GJA1/AGTR1/REN 3

BP GO:0007530 sex determination 3/227 25/18866 0.003251678 0.007439114 0.002810072 AR/INSR/GNRH1 3

BP GO:0014829 vascular associated smooth muscle contraction 3/227 25/18866 0.003251678 0.007439114 0.002810072 CHRM3/HTR2A/EDN1 3

BP GO:0015874 norepinephrine transport 3/227 25/18866 0.003251678 0.007439114 0.002810072 SLC6A3/SNCA/HRH3 3

BP GO:0019400 alditol metabolic process 3/227 25/18866 0.003251678 0.007439114 0.002810072 AKR1B1/TPI1/PLA2G4A 3

BP GO:0032647 regulation of interferon-alpha production 3/227 25/18866 0.003251678 0.007439114 0.002810072 IL10/STAT1/TLR4 3

BP GO:0046639 negative regulation of alpha-beta T cell differentiation 3/227 25/18866 0.003251678 0.007439114 0.002810072 IL2/IL4/SHH 3

BP GO:0050927 positive regulation of positive chemotaxis 3/227 25/18866 0.003251678 0.007439114 0.002810072 VEGFA/KDR/CXCL8 3

BP GO:0060259 regulation of feeding behavior 3/227 25/18866 0.003251678 0.007439114 0.002810072 STAT3/MTOR/EIF2AK4 3

BP GO:0061050 regulation of cell growth involved in cardiac muscle cell development 3/227 25/18866 0.003251678 0.007439114 0.002810072 EDN1/PPARA/MTOR 3

BP GO:0062149 detection of stimulus involved in sensory perception of pain 3/227 25/18866 0.003251678 0.007439114 0.002810072 HTR2A/NTRK1/TRPV1 3

BP GO:0006473 protein acetylation 8/227 203/18866 0.003257394 0.007448606 0.002813658 IL1B/SNCA/MAPK3/SIRT1/FOXO1/BRCA1/ATF2/EP300 8

BP GO:0055013 cardiac muscle cell development 5/227 83/18866 0.00327332 0.007474481 0.002823432 VEGFA/CDK1/EDN1/PPARA/MTOR 5

BP GO:0002762 negative regulation of myeloid leukocyte differentiation 4/227 51/18866 0.003276571 0.007474481 0.002823432 MYC/TLR4/CDK6/IL4 4

BP GO:0032873 negative regulation of stress-activated MAPK cascade 4/227 51/18866 0.003276571 0.007474481 0.002823432 AKT1/MYC/FOXO1/GSTP1 4

BP GO:0070303 negative regulation of stress-activated protein kinase signaling cascade 4/227 51/18866 0.003276571 0.007474481 0.002823432 AKT1/MYC/FOXO1/GSTP1 4

BP GO:0071827 plasma lipoprotein particle organization 4/227 51/18866 0.003276571 0.007474481 0.002823432 MPO/AGTR1/CETP/ABCG1 4

BP GO:0030279 negative regulation of ossification 5/227 84/18866 0.003447053 0.00785208 0.002966067 BCL2/TNF/HIF1A/CDK6/CCR1 5

BP GO:0050886 endocrine process 5/227 84/18866 0.003447053 0.00785208 0.002966067 IL1B/EDN1/GJA1/AGTR1/REN 5

BP GO:0097061 dendritic spine organization 5/227 84/18866 0.003447053 0.00785208 0.002966067 CHRNA7/CDK5/IGF1R/CDC42/INSR 5

BP GO:0038066 p38MAPK cascade 4/227 52/18866 0.003516637 0.008002917 0.003023045 VEGFA/XDH/IL1B/MAPK14 4

BP GO:2001238 positive regulation of extrinsic apoptotic signaling pathway 4/227 52/18866 0.003516637 0.008002917 0.003023045 TNF/ATF3/TNFSF10/RET 4

BP GO:0009199 ribonucleoside triphosphate metabolic process 5/227 85/18866 0.003627317 0.008236386 0.003111236 TGFB1/PARP1/STAT3/IL4/VCP 5

BP GO:0007088 regulation of mitotic nuclear division 7/227 163/18866 0.003636713 0.008236386 0.003111236 EGF/IL1B/EDN1/IL1A/CDC42/CD28/INSR 7

BP GO:0006309 apoptotic DNA fragmentation 3/227 26/18866 0.003643483 0.008236386 0.003111236 BAX/CASP3/IL6 3

BP GO:0034114 regulation of heterotypic cell-cell adhesion 3/227 26/18866 0.003643483 0.008236386 0.003111236 IL10/TNF/IL1B 3

BP GO:0042730 fibrinolysis 3/227 26/18866 0.003643483 0.008236386 0.003111236 PLAU/F2/PLAT 3

BP GO:0044068 modulation by symbiont of host cellular process 3/227 26/18866 0.003643483 0.008236386 0.003111236 CASP8/BCL2L1/EIF2AK4 3

BP GO:0048643 positive regulation of skeletal muscle tissue development 3/227 26/18866 0.003643483 0.008236386 0.003111236 BCL2/HMGCR/SHH 3

BP GO:0050926 regulation of positive chemotaxis 3/227 26/18866 0.003643483 0.008236386 0.003111236 VEGFA/KDR/CXCL8 3

BP GO:0051123 RNA polymerase II preinitiation complex assembly 3/227 26/18866 0.003643483 0.008236386 0.003111236 TP53/ESR1/CREB1 3

BP GO:0060384 innervation 3/227 26/18866 0.003643483 0.008236386 0.003111236 NTRK1/PRKCG/RET 3

BP GO:0060390 regulation of SMAD protein signal transduction 3/227 26/18866 0.003643483 0.008236386 0.003111236 TGFB1/PARP1/TGFBR1 3

BP GO:0090023 positive regulation of neutrophil chemotaxis 3/227 26/18866 0.003643483 0.008236386 0.003111236 EDN1/CXCL8/C5AR1 3

BP GO:0097066 response to thyroid hormone 3/227 26/18866 0.003643483 0.008236386 0.003111236 AKR1B1/GCLC/CTSB 3

BP GO:1903649 regulation of cytoplasmic transport 3/227 26/18866 0.003643483 0.008236386 0.003111236 MAP2/MAPK1/MAPK3 3

BP GO:1901343 negative regulation of vasculature development 8/227 207/18866 0.003666217 0.008283838 0.00312916 PPARG/TNF/XDH/STAT1/FASLG/CCR2/ATF2/ALOX5 8

BP GO:0090092 regulation of transmembrane receptor protein serine/threonine kinase signaling pathway 9/227 254/18866 0.003741962 0.008450969 0.003192293 TGFB1/TP53/PARP1/SIRT1/XIAP/SHH/EP300/TGFBR1/TMPRSS6 9

BP GO:0018393 internal peptidyl-lysine acetylation 7/227 164/18866 0.003760959 0.008489839 0.003206976 IL1B/SNCA/MAPK3/SIRT1/BRCA1/ATF2/EP300 7

BP GO:0043392 negative regulation of DNA binding 4/227 53/18866 0.003768382 0.008502557 0.00321178 JUN/HMOX1/DDIT3/PSEN1 4

BP GO:0006721 terpenoid metabolic process 6/227 124/18866 0.003904897 0.008806395 0.003326553 EGFR/PPARD/AKR1B1/TTR/CYP3A4/FDFT1 6

BP GO:0002377 immunoglobulin production 8/227 210/18866 0.00399808 0.009012268 0.00340432 TGFB1/IL10/TNF/IL6/CD28/IL2/IL4/CD40LG 8

BP GO:0001942 hair follicle development 5/227 87/18866 0.004007977 0.009026017 0.003409513 BCL2/RELA/EGFR/TNF/SHH 5

BP GO:0009144 purine nucleoside triphosphate metabolic process 5/227 87/18866 0.004007977 0.009026017 0.003409513 TGFB1/PARP1/STAT3/IL4/VCP 5

BP GO:0006475 internal protein amino acid acetylation 7/227 166/18866 0.004019107 0.009046797 0.003417363 IL1B/SNCA/MAPK3/SIRT1/BRCA1/ATF2/EP300 7

BP GO:0033044 regulation of chromosome organization 11/227 356/18866 0.004025225 0.00905628 0.003420945 VEGFA/MAPK1/TP53/MYC/IL1B/SNCA/MAPK3/PARP1/SIRT1/MAPK8/BRCA1 11

BP GO:0009620 response to fungus 4/227 54/18866 0.004032068 0.009063097 0.00342352 TLR4/SYK/MPO/BAK1 4

BP GO:0010043 response to zinc ion 4/227 54/18866 0.004032068 0.009063097 0.00342352 CA2/PARP1/PAM/CREB1 4

BP GO:0001952 regulation of cell-matrix adhesion 6/227 125/18866 0.004061524 0.009102257 0.003438313 PLAU/BCL2/VEGFA/KDR/MMP12/CDK6 6

BP GO:0032607 interferon-alpha production 3/227 27/18866 0.004062892 0.009102257 0.003438313 IL10/STAT1/TLR4 3

BP GO:0042501 serine phosphorylation of STAT protein 3/227 27/18866 0.004062892 0.009102257 0.003438313 CDK5/IFNG/RET 3

BP GO:0048668 collateral sprouting 3/227 27/18866 0.004062892 0.009102257 0.003438313 BDNF/NGF/APP 3

BP GO:0051953 negative regulation of amine transport 3/227 27/18866 0.004062892 0.009102257 0.003438313 SNCA/HRH3/DRD2 3

BP GO:1900120 regulation of receptor binding 3/227 27/18866 0.004062892 0.009102257 0.003438313 MMP9/IL10/BDNF 3

BP GO:2000353 positive regulation of endothelial cell apoptotic process 3/227 27/18866 0.004062892 0.009102257 0.003438313 FASLG/CCL2/CD40LG 3

BP GO:0031589 cell-substrate adhesion 11/227 359/18866 0.004284901 0.009595111 0.003624485 PLAU/BCL2/VEGFA/KDR/PPARD/MMP12/CDK5/CDK6/VCAM1/PECAM1/CDC42 11

BP GO:0022029 telencephalon cell migration 4/227 55/18866 0.004307955 0.009633123 0.003638843 EGFR/CDK5/PSEN1/DRD2 4

BP GO:0045620 negative regulation of lymphocyte differentiation 4/227 55/18866 0.004307955 0.009633123 0.003638843 ERBB2/IL2/IL4/SHH 4

BP GO:0071825 protein-lipid complex subunit organization 4/227 55/18866 0.004307955 0.009633123 0.003638843 MPO/AGTR1/CETP/ABCG1 4

BP GO:0022404 molting cycle process 5/227 89/18866 0.00441638 0.009852403 0.003721675 BCL2/RELA/EGFR/TNF/SHH 5

BP GO:0022405 hair cycle process 5/227 89/18866 0.00441638 0.009852403 0.003721675 BCL2/RELA/EGFR/TNF/SHH 5

BP GO:0043506 regulation of JUN kinase activity 5/227 89/18866 0.00441638 0.009852403 0.003721675 TNF/SYK/EDN1/GSTP1/CD40LG 5

BP GO:0055006 cardiac cell development 5/227 89/18866 0.00441638 0.009852403 0.003721675 VEGFA/CDK1/EDN1/PPARA/MTOR 5

BP GO:1903845 negative regulation of cellular response to transforming growth factor beta stimulus 5/227 89/18866 0.00441638 0.009852403 0.003721675 TGFB1/TP53/SIRT1/CFLAR/TGFBR1 5

CC GO:0045121 membrane raft 36/227 329/19559 1.07011E-24 2.0401E-22 1.35861E-22 PTGS2/SLC6A3/HTR2A/CHRNA7/SLC6A4/OPRM1/CASP3/CASP8/DPP4/EGFR/MAPK1/TNF/KDR/HMOX1/MAPK3/APP/ABCG2/FASLG/GJA1/ICAM1/SELE/PECAM1/CFLAR/TNFRSF10A/BIRC3/INSR/KCNMA1/PSEN1/PSEN2/SHH/TNFRSF1A/TNFRSF1B/BIRC2/CTSD/TGFBR1/RET 36

CC GO:0098857 membrane microdomain 36/227 330/19559 1.18957E-24 2.0401E-22 1.35861E-22 PTGS2/SLC6A3/HTR2A/CHRNA7/SLC6A4/OPRM1/CASP3/CASP8/DPP4/EGFR/MAPK1/TNF/KDR/HMOX1/MAPK3/APP/ABCG2/FASLG/GJA1/ICAM1/SELE/PECAM1/CFLAR/TNFRSF10A/BIRC3/INSR/KCNMA1/PSEN1/PSEN2/SHH/TNFRSF1A/TNFRSF1B/BIRC2/CTSD/TGFBR1/RET 36

CC GO:0098589 membrane region 36/227 343/19559 4.55623E-24 5.20929E-22 3.46913E-22 PTGS2/SLC6A3/HTR2A/CHRNA7/SLC6A4/OPRM1/CASP3/CASP8/DPP4/EGFR/MAPK1/TNF/KDR/HMOX1/MAPK3/APP/ABCG2/FASLG/GJA1/ICAM1/SELE/PECAM1/CFLAR/TNFRSF10A/BIRC3/INSR/KCNMA1/PSEN1/PSEN2/SHH/TNFRSF1A/TNFRSF1B/BIRC2/CTSD/TGFBR1/RET 36

CC GO:0031983 vesicle lumen 23/227 328/19559 5.80383E-12 4.97679E-10 3.31429E-10 TGFB1/EGFR/VEGFA/MAPK1/EGF/HSP90AA1/APP/MMP8/CAT/FUCA1/TTR/MPO/MAPK14/FASLG/NFKB1/GSTP1/VCP/GM2A/PTPN6/ALOX5/GLB1/CTSD/IDH1 23

CC GO:0060205 cytoplasmic vesicle lumen 22/227 326/19559 3.62472E-11 2.48656E-09 1.65592E-09 TGFB1/VEGFA/MAPK1/EGF/HSP90AA1/APP/MMP8/CAT/FUCA1/TTR/MPO/MAPK14/FASLG/NFKB1/GSTP1/VCP/GM2A/PTPN6/ALOX5/GLB1/CTSD/IDH1 22

CC GO:0034774 secretory granule lumen 21/227 322/19559 1.94187E-10 1.1101E-08 7.39275E-09 TGFB1/VEGFA/MAPK1/EGF/HSP90AA1/APP/MMP8/CAT/FUCA1/TTR/MPO/MAPK14/NFKB1/GSTP1/VCP/GM2A/PTPN6/ALOX5/GLB1/CTSD/IDH1 21

CC GO:0090575 RNA polymerase II transcription regulator complex 14/227 161/19559 6.02226E-09 2.95091E-07 1.96516E-07 RXRA/JUN/PPARG/FOS/TP53/HIF1A/DDIT3/STAT3/CDK7/NR1H3/RXRB/CREB1/RXRG/VDR 14

CC GO:0045177 apical part of cell 22/227 433/19559 7.53577E-09 3.23096E-07 2.15166E-07 DPP4/EGFR/CA2/APP/SI/MGAM/ABCG2/ABCB1/GJA1/VCAM1/PLAT/C5AR1/CDC42/ERBB2/KCNMA1/GM2A/TRPV4/PSEN1/PSEN2/CTSB/NPC1L1/REN 22

CC GO:0009897 external side of plasma membrane 21/227 417/19559 1.96029E-08 7.06678E-07 4.70613E-07 TNF/TLR4/ABCG2/ABCB1/F2/FASLG/ICAM1/VCAM1/PECAM1/CD80/CCR2/CD28/INSR/CD40LG/CTSB/GRIA2/TRPV1/ABCG1/CCR1/CCR3/CCR5 21

CC GO:0101002 ficolin-1-rich granule 12/227 124/19559 2.26632E-08 7.06678E-07 4.70613E-07 MMP9/MAPK1/HSP90AA1/CAT/MAPK14/GSTP1/VCP/CTSB/ALOX5/GLB1/CTSD/IDH1 12

CC GO:1904813 ficolin-1-rich granule lumen 12/227 124/19559 2.26632E-08 7.06678E-07 4.70613E-07 MMP9/MAPK1/HSP90AA1/CAT/MAPK14/GSTP1/VCP/CTSB/ALOX5/GLB1/CTSD/IDH1 12

CC GO:0005901 caveola 10/227 82/19559 3.75026E-08 1.07195E-06 7.13865E-07 PTGS2/SLC6A3/HTR2A/MAPK1/HMOX1/MAPK3/FASLG/SELE/INSR/KCNMA1 10

CC GO:0044853 plasma membrane raft 11/227 113/19559 8.31628E-08 2.19422E-06 1.46124E-06 PTGS2/SLC6A3/HTR2A/CHRNA7/MAPK1/HMOX1/MAPK3/FASLG/SELE/INSR/KCNMA1 11

CC GO:0099056 integral component of presynaptic membrane 8/227 74/19559 2.26991E-06 5.56127E-05 3.70353E-05 SLC6A3/CHRM3/HTR2A/SLC6A4/OPRM1/PSEN1/PSEN2/DRD2 8

CC GO:0098889 intrinsic component of presynaptic membrane 8/227 83/19559 5.43192E-06 0.00012421 8.27177E-05 SLC6A3/CHRM3/HTR2A/SLC6A4/OPRM1/PSEN1/PSEN2/DRD2 8

CC GO:0005775 vacuolar lumen 11/227 173/19559 5.85765E-06 0.000125573 8.36257E-05 MAPK1/HSP90AA1/FUCA1/TTR/MPO/FASLG/VCP/GM2A/CTSB/GLB1/CTSD 11

CC GO:0005667 transcription regulator complex 17/227 413/19559 7.12293E-06 0.000143716 9.57075E-05 RXRA/JUN/RELA/PPARG/FOS/TP53/HIF1A/DDIT3/PARP1/STAT3/CDK7/NR1H3/RXRB/CREB1/RXRG/VDR/EP300 17

CC GO:0031968 organelle outer membrane 12/227 218/19559 9.66849E-06 0.000184238 0.000122694 PGR/PTGS2/BCL2/BAX/CASP8/BCL2L1/MCL1/BAK1/GJA1/MTOR/PSEN1/ATF2 12

CC GO:0019867 outer membrane 12/227 220/19559 1.06016E-05 0.000191001 0.000127197 PGR/PTGS2/BCL2/BAX/CASP8/BCL2L1/MCL1/BAK1/GJA1/MTOR/PSEN1/ATF2 12

CC GO:0005635 nuclear envelope 18/227 473/19559 1.11371E-05 0.000191001 0.000127197 PTGS2/BCL2/BAX/EGFR/BCL2L1/TP53/MAPK3/APP/PARP1/SIRT1/MTOR/INSR/BCHE/PSEN1/PSEN2/GPX4/ALOX5/PLA2G4A 18

CC GO:0043204 perikaryon 10/227 156/19559 1.45569E-05 0.000237763 0.000158338 OPRM1/MAPK1/TOP1/APP/CDK5/CCR2/GNRH1/PAM/MAPK10/DRD2 10

CC GO:0150034 distal axon 14/227 309/19559 1.62241E-05 0.000252949 0.000168452 CHRM3/MAP2/SNCA/NTRK2/HSP90AA1/APP/CDK5/PRKCB/GNRH1/PRKCG/TRPV4/PSEN1/PSEN2/DRD2 14

CC GO:0016324 apical plasma membrane 15/227 361/19559 2.23367E-05 0.000333108 0.000221833 DPP4/EGFR/SI/MGAM/ABCG2/ABCB1/GJA1/ERBB2/KCNMA1/GM2A/TRPV4/PSEN1/PSEN2/CTSB/NPC1L1 15

CC GO:1902911 protein kinase complex 8/227 104/19559 2.87954E-05 0.000411535 0.000274062 CDKN1A/CDK1/CDK5/CDK6/IGF1R/CDK7/INSR/TGFBR1 8

CC GO:0005769 early endosome 15/227 377/19559 3.682E-05 0.00050517 0.000336419 EGFR/MAPK1/KDR/TLR4/MAPK3/NTRK1/NTRK2/APP/GJA1/VCAM1/ERBB2/PSEN1/PSEN2/LITAF/RET 15

CC GO:1904724 tertiary granule lumen 6/227 55/19559 4.12457E-05 0.000544126 0.000362361 MMP9/MMP8/PRSS3/PTPN6/CTSD/IDH1 6

CC GO:0097060 synaptic membrane 15/227 387/19559 4.9602E-05 0.000630129 0.000419635 SLC6A3/CHRM3/HTR2A/CHRNA7/SLC6A4/OPRM1/GABBR1/GRM1/KCNMA1/PRKCG/PSEN1/PSEN2/GRIA2/TRPV1/DRD2 15

CC GO:0043198 dendritic shaft 5/227 38/19559 7.40099E-05 0.000906621 0.000603765 HTR2A/MAP2/APP/PSEN1/PSEN2 5

CC GO:0005741 mitochondrial outer membrane 10/227 192/19559 8.5501E-05 0.001011271 0.000673457 PGR/BCL2/BAX/CASP8/BCL2L1/MCL1/BAK1/GJA1/MTOR/ATF2 10

CC GO:0042734 presynaptic membrane 9/227 156/19559 8.88312E-05 0.001013025 0.000674624 SLC6A3/CHRM3/HTR2A/SLC6A4/OPRM1/GABBR1/PSEN1/PSEN2/DRD2 9

CC GO:0035578 azurophil granule lumen 7/227 91/19559 9.15562E-05 0.001013025 0.000674624 MAPK1/FUCA1/TTR/MPO/VCP/GM2A/GLB1 7

CC GO:0045211 postsynaptic membrane 12/227 280/19559 0.000111362 0.001193658 0.000794918 SLC6A3/CHRM3/HTR2A/CHRNA7/SLC6A4/OPRM1/GABBR1/GRM1/KCNMA1/GRIA2/TRPV1/DRD2 12

CC GO:0043202 lysosomal lumen 7/227 96/19559 0.000128387 0.001334445 0.000888675 HSP90AA1/FUCA1/FASLG/GM2A/CTSB/GLB1/CTSD 7

CC GO:0005641 nuclear envelope lumen 3/227 10/19559 0.000174316 0.001758545 0.001171104 APP/BCHE/ALOX5 3

CC GO:0043209 myelin sheath 5/227 47/19559 0.000207788 0.002036319 0.001356088 BCL2/HSP90AA1/CA2/AKR1B1/ERBB2 5

CC GO:0035253 ciliary rootlet 3/227 11/19559 0.000237634 0.002264122 0.001507793 APP/PSEN1/PSEN2 3

CC GO:0098802 plasma membrane signaling receptor complex 12/227 307/19559 0.000261242 0.002421781 0.001612787 CHRNA7/IL6/SYK/IGF1R/ERBB2/INSR/GABBR1/GRM1/PTPN6/GRIA2/BIRC2/TGFBR1 12

CC GO:0098685 Schaffer collateral - CA1 synapse 6/227 84/19559 0.000437428 0.003883544 0.002586248 CDK5/STAT3/PLAT/CDC42/GABBR1/GRM1 6

CC GO:0099699 integral component of synaptic membrane 8/227 154/19559 0.000444618 0.003883544 0.002586248 SLC6A3/CHRM3/HTR2A/SLC6A4/OPRM1/PSEN1/PSEN2/DRD2 8

CC GO:0005766 primary lysosome 8/227 155/19559 0.000464214 0.003883544 0.002586248 MAPK1/FUCA1/TTR/MPO/VCP/GM2A/PSEN1/GLB1 8

CC GO:0042582 azurophil granule 8/227 155/19559 0.000464214 0.003883544 0.002586248 MAPK1/FUCA1/TTR/MPO/VCP/GM2A/PSEN1/GLB1 8

CC GO:1902554 serine/threonine protein kinase complex 6/227 89/19559 0.000596048 0.004860107 0.003236591 CDKN1A/CDK1/CDK5/CDK6/CDK7/TGFBR1 6

CC GO:0005719 nuclear euchromatin 4/227 34/19559 0.000623454 0.004860107 0.003236591 JUN/SIRT1/CREB1/NR1H4 4

CC GO:0032839 dendrite cytoplasm 4/227 34/19559 0.000623454 0.004860107 0.003236591 OPRM1/MAP2/MAPK1/SOD1 4

CC GO:0031091 platelet alpha granule 6/227 91/19559 0.000670713 0.005026545 0.003347431 TGFB1/VEGFA/EGF/SNCA/APP/PECAM1 6

CC GO:0070820 tertiary granule 8/227 164/19559 0.000674114 0.005026545 0.003347431 PLAU/MMP9/MMP8/MGAM/PRSS3/PTPN6/CTSD/IDH1 8

CC GO:0099240 intrinsic component of synaptic membrane 8/227 166/19559 0.000729815 0.005326096 0.003546917 SLC6A3/CHRM3/HTR2A/SLC6A4/OPRM1/PSEN1/PSEN2/DRD2 8

CC GO:0061695 transferase complex, transferring phosphorus-containing groups 10/227 253/19559 0.000776057 0.005545573 0.003693078 PIK3CG/CDKN1A/TP53/CDK1/CDK5/CDK6/IGF1R/CDK7/INSR/TGFBR1 10

CC GO:0043679 axon terminus 7/227 130/19559 0.000816582 0.005716073 0.003806622 CHRM3/SNCA/NTRK2/PRKCB/GNRH1/PRKCG/DRD2 7

CC GO:0098978 glutamatergic synapse 12/227 361/19559 0.001093552 0.007471725 0.0049758 CHRM3/HTR2A/RELA/CDK5/MAPK14/STAT3/PLAT/MTOR/GRM1/VCP/MAPK10/DRD2 12

CC GO:0098858 actin-based cell projection 9/227 220/19559 0.001110956 0.007471725 0.0049758 TGFB1/MAP2/CA2/AKR1B1/APP/CDK5/VCAM1/CDC42/TRPV4 9

CC GO:0005925 focal adhesion 13/227 415/19559 0.001175818 0.007755879 0.005165032 PLAU/SLC6A4/OPRM1/DPP4/EGFR/MAPK1/MAPK3/CAT/GJA1/ICAM1/JAK1/CDC42/TRPV4 13

CC GO:0030055 cell-substrate junction 13/227 423/19559 0.001394942 0.008836797 0.00588487 PLAU/SLC6A4/OPRM1/DPP4/EGFR/MAPK1/MAPK3/CAT/GJA1/ICAM1/JAK1/CDC42/TRPV4 13

CC GO:0000791 euchromatin 4/227 42/19559 0.001399574 0.008836797 0.00588487 JUN/SIRT1/CREB1/NR1H4 4

CC GO:0030426 growth cone 8/227 184/19559 0.001416979 0.008836797 0.00588487 MAP2/SNCA/HSP90AA1/APP/CDK5/TRPV4/PSEN1/PSEN2 8

CC GO:0030175 filopodium 6/227 106/19559 0.001486897 0.009107244 0.006064975 MAP2/APP/CDK5/VCAM1/CDC42/TRPV4 6

CC GO:0000307 cyclin-dependent protein kinase holoenzyme complex 4/227 43/19559 0.001529233 0.009202229 0.00612823 CDKN1A/CDK1/CDK6/CDK7 4

MF GO:0005126 cytokine receptor binding 29/227 271/18352 5.03342E-19 3.12072E-16 2.02396E-16 CASP3/CASP8/TGFB1/VEGFA/IL10/TNF/IL6/STAT1/IL1B/BDNF/NGF/NTRK1/SYK/FASLG/STAT3/CCL2/CXCL8/IL1A/JAK1/CFLAR/CCR2/TNFSF10/IL2/IFNG/IL4/CD40LG/CSF2/TYK2/TGFBR1 29

MF GO:0004879 nuclear receptor activity 15/227 52/18352 4.55605E-17 9.41583E-15 6.1067E-15 PGR/RXRA/AR/PPARG/ESR2/PPARD/ESR1/STAT3/PPARA/NR1H3/RXRB/NR1I2/NR1H4/RXRG/VDR 15

MF GO:0098531 ligand-activated transcription factor activity 15/227 52/18352 4.55605E-17 9.41583E-15 6.1067E-15 PGR/RXRA/AR/PPARG/ESR2/PPARD/ESR1/STAT3/PPARA/NR1H3/RXRB/NR1I2/NR1H4/RXRG/VDR 15

MF GO:0019902 phosphatase binding 21/227 194/18352 3.67546E-14 5.69696E-12 3.6948E-12 SLC6A3/BCL2/PPARG/EGFR/AKT1/MAPK1/TP53/SOD1/STAT1/MAPK3/SYK/MAPK14/STAT3/FOXO1/PPARA/JAK1/ERBB2/VCP/HMGCR/CSF1R/EIF2AK3 21

MF GO:0140297 DNA-binding transcription factor binding 25/227 347/18352 1.4268E-12 1.76923E-10 1.14745E-10 RXRA/BCL2/JUN/RELA/PPARG/FOS/HIF1A/STAT1/PPARD/ESR1/MYC/NFE2L2/DDIT3/PARP1/MAPK14/SIRT1/STAT3/PRKCB/PPARA/NR1I2/CREB1/ATF2/NR1H4/VDR/EP300 25

MF GO:0061629 RNA polymerase II-specific DNA-binding transcription factor binding 22/227 267/18352 2.3879E-12 2.4675E-10 1.60031E-10 RXRA/JUN/RELA/PPARG/FOS/HIF1A/STAT1/PPARD/ESR1/NFE2L2/PARP1/MAPK14/SIRT1/STAT3/PRKCB/PPARA/NR1I2/CREB1/ATF2/NR1H4/VDR/EP300 22

MF GO:0030546 signaling receptor activator activity 29/227 492/18352 3.1617E-12 2.80037E-10 1.8162E-10 TGFB1/DPP4/VEGFA/IL10/EGF/TNF/IL6/IL1B/GDNF/BDNF/NGF/APP/CDK5/TTR/F2/FASLG/EDN1/CCL2/CXCL8/IL1A/IL17B/TNFSF10/IL2/IFNG/IL4/CD40LG/GNRH1/CSF2/SHH 29

MF GO:0019903 protein phosphatase binding 17/227 149/18352 4.60626E-12 3.56985E-10 2.31525E-10 SLC6A3/BCL2/PPARG/EGFR/AKT1/TP53/SOD1/STAT1/MAPK14/STAT3/FOXO1/JAK1/ERBB2/VCP/HMGCR/CSF1R/EIF2AK3 17

MF GO:0008144 drug binding 14/227 104/18352 3.90566E-11 2.69057E-09 1.74499E-09 SLC6A3/CHRM3/HTR2A/SLC6A4/PPARG/PPARD/HTR2C/PPARA/GSTP1/NR1I2/NPC1L1/DHFR/DRD2/HTR2B 14

MF GO:0048018 receptor ligand activity 27/227 487/18352 7.54644E-11 4.67879E-09 3.03446E-09 TGFB1/DPP4/VEGFA/IL10/EGF/TNF/IL6/IL1B/GDNF/BDNF/NGF/TTR/F2/FASLG/EDN1/CCL2/CXCL8/IL1A/IL17B/TNFSF10/IL2/IFNG/IL4/CD40LG/GNRH1/CSF2/SHH 27

MF GO:0004674 protein serine/threonine kinase activity 25/227 435/18352 1.88532E-10 1.06263E-08 6.89179E-09 PIK3CG/PRKCA/EGFR/AKT1/MAPK1/TOP1/MAPK3/CDK1/CDK5/SYK/CDK6/MAPK14/MAPK8/PRKCB/CDK7/MTOR/PRKCG/PRKG1/EIF2AK3/MAPK10/MAPK9/TGFBR1/EIF2AK4/EIF2AK2/EIF2AK1 25

MF GO:0004175 endopeptidase activity 25/227 440/18352 2.39633E-10 1.2381E-08 8.02981E-09 PLAU/CASP9/CASP3/CASP8/DPP4/MMP3/MMP2/MMP9/MMP1/MMP12/MMP7/MMP8/F2/PLAT/CFLAR/PRSS3/PSEN1/PSEN2/CTSB/MMP10/CASP1/C1R/CTSD/REN/TMPRSS6 25

MF GO:0032813 tumor necrosis factor receptor superfamily binding 10/227 48/18352 2.9818E-10 1.42209E-08 9.22306E-09 CASP3/CASP8/TNF/STAT1/BDNF/NGF/FASLG/CFLAR/TNFSF10/CD40LG 10

MF GO:0003707 steroid hormone receptor activity 8/227 26/18352 6.24671E-10 2.7664E-08 1.79417E-08 PGR/RXRA/ESR2/PPARD/ESR1/PPARA/RXRB/RXRG 8

MF GO:0051427 hormone receptor binding 16/227 177/18352 6.7108E-10 2.7738E-08 1.79896E-08 RXRA/PPARG/HIF1A/STAT1/ESR1/PARP1/SIRT1/STAT3/PRKCB/JAK1/GNRH1/NR1I2/NR1H4/VDR/EP300/TYK2 16

MF GO:0005125 cytokine activity 18/227 235/18352 8.369E-10 3.24299E-08 2.10326E-08 TGFB1/VEGFA/IL10/TNF/IL6/IL1B/FASLG/EDN1/CCL2/CXCL8/IL1A/IL17B/TNFSF10/IL2/IFNG/IL4/CD40LG/CSF2 18

MF GO:0001223 transcription coactivator binding 8/227 29/18352 1.66223E-09 6.06226E-08 3.93172E-08 PGR/RELA/AR/PPARD/ESR1/FOXO1/PPARA/CREB1 8

MF GO:0001221 transcription cofactor binding 9/227 51/18352 1.12069E-08 3.86016E-07 2.50353E-07 PGR/RELA/AR/PPARD/ESR1/NFE2L2/FOXO1/PPARA/CREB1 9

MF GO:0004713 protein tyrosine kinase activity 13/227 135/18352 1.30114E-08 4.24582E-07 2.75366E-07 EGFR/KDR/NTRK1/NTRK2/SYK/IGF1R/JAK1/ERBB2/INSR/CSF1R/TYK2/EIF2AK2/RET 13

MF GO:0035257 nuclear hormone receptor binding 13/227 144/18352 2.83701E-08 8.79473E-07 5.70388E-07 RXRA/PPARG/HIF1A/STAT1/ESR1/PARP1/SIRT1/STAT3/PRKCB/NR1I2/NR1H4/VDR/EP300 13

MF GO:0019199 transmembrane receptor protein kinase activity 10/227 80/18352 5.31493E-08 1.56917E-06 1.0177E-06 EGFR/KDR/NTRK1/NTRK2/IGF1R/ERBB2/INSR/CSF1R/TGFBR1/RET 10

MF GO:0004714 transmembrane receptor protein tyrosine kinase activity 9/227 61/18352 5.7387E-08 1.61727E-06 1.04889E-06 EGFR/KDR/NTRK1/NTRK2/IGF1R/ERBB2/INSR/CSF1R/RET 9

MF GO:0097199 cysteine-type endopeptidase activity involved in apoptotic signaling pathway 5/227 10/18352 6.63886E-08 1.78961E-06 1.16066E-06 CASP9/CASP3/CASP8/CFLAR/CASP1 5

MF GO:0044389 ubiquitin-like protein ligase binding 18/227 316/18352 8.51144E-08 2.19879E-06 1.42604E-06 BCL2/JUN/CASP8/RELA/EGFR/CDKN1A/TP53/HIF1A/STAT1/HSP90AA1/ABCB1/FOXO1/BRCA1/JAK1/MDM2/VCP/TPI1/TNFRSF1B 18

MF GO:0051721 protein phosphatase 2A binding 7/227 32/18352 1.04487E-07 2.552E-06 1.65512E-06 SLC6A3/BCL2/AKT1/TP53/STAT1/FOXO1/HMGCR 7

MF GO:0004707 MAP kinase activity 6/227 20/18352 1.12415E-07 2.552E-06 1.65512E-06 MAPK1/MAPK3/MAPK14/MAPK8/MAPK10/MAPK9 6

MF GO:0005123 death receptor binding 6/227 20/18352 1.12415E-07 2.552E-06 1.65512E-06 CASP3/CASP8/BDNF/NGF/FASLG/CFLAR 6

MF GO:0017171 serine hydrolase activity 14/227 191/18352 1.15252E-07 2.552E-06 1.65512E-06 PLAU/DPP4/MMP3/ACHE/MMP2/MMP9/MMP1/MMP7/MMP8/F2/PLAT/PRSS3/C1R/TMPRSS6 14

MF GO:0051400 BH domain binding 5/227 11/18352 1.20487E-07 2.57592E-06 1.67063E-06 BCL2/BAX/BCL2L1/MCL1/BAK1 5

MF GO:0030594 neurotransmitter receptor activity 11/227 111/18352 1.30192E-07 2.69063E-06 1.74503E-06 CHRM3/HTR2A/CHRNA7/HTR2C/GABBR1/GRM1/GRIA2/HRH3/HTR1A/DRD2/HTR2B 11

MF GO:0031625 ubiquitin protein ligase binding 17/227 297/18352 1.838E-07 3.542E-06 2.29719E-06 BCL2/JUN/CASP8/RELA/EGFR/CDKN1A/TP53/HIF1A/HSP90AA1/ABCB1/FOXO1/BRCA1/JAK1/MDM2/VCP/TPI1/TNFRSF1B 17

MF GO:0070851 growth factor receptor binding 12/227 141/18352 1.8778E-07 3.542E-06 2.29719E-06 VEGFA/IL10/EGF/IL6/IL1B/GDNF/APP/IL1A/IL2/IL4/PSEN1/CSF2 12

MF GO:0004252 serine-type endopeptidase activity 13/227 169/18352 1.88526E-07 3.542E-06 2.29719E-06 PLAU/DPP4/MMP3/MMP2/MMP9/MMP1/MMP7/MMP8/F2/PLAT/PRSS3/C1R/TMPRSS6 13

MF GO:0043176 amine binding 5/227 12/18352 2.04469E-07 3.62202E-06 2.34909E-06 HTR2A/SLC6A4/HTR2C/HTR1A/HTR2B 5

MF GO:0051378 serotonin binding 5/227 12/18352 2.04469E-07 3.62202E-06 2.34909E-06 HTR2A/SLC6A4/HTR2C/HTR1A/HTR2B 5

MF GO:0016922 nuclear receptor binding 10/227 101/18352 4.94625E-07 8.51855E-06 5.52476E-06 RXRA/PPARG/ESR1/PARP1/STAT3/PRKCB/NR1I2/NR1H4/VDR/EP300 10

MF GO:0008236 serine-type peptidase activity 13/227 187/18352 6.05407E-07 1.01447E-05 6.57939E-06 PLAU/DPP4/MMP3/MMP2/MMP9/MMP1/MMP7/MMP8/F2/PLAT/PRSS3/C1R/TMPRSS6 13

MF GO:0033613 activating transcription factor binding 9/227 80/18352 6.26101E-07 1.02153E-05 6.62523E-06 JUN/RELA/PPARG/FOS/MYC/NFE2L2/CREB1/ATF2/EP300 9

MF GO:0097153 cysteine-type endopeptidase activity involved in apoptotic process 5/227 15/18352 7.52107E-07 1.19566E-05 7.75452E-06 CASP9/CASP3/CASP8/CFLAR/CASP1 5

MF GO:0005496 steroid binding 10/227 106/18352 7.77094E-07 1.2045E-05 7.81184E-06 PGR/AR/ESR2/ESR1/NR1H3/CYP3A4/NR1H4/CETP/ABCG1/VDR 10

MF GO:0008083 growth factor activity 12/227 162/18352 8.43435E-07 1.27544E-05 8.27195E-06 TGFB1/VEGFA/IL10/EGF/IL6/GDNF/BDNF/NGF/F2/IL2/IL4/CSF2 12

MF GO:0002020 protease binding 11/227 137/18352 1.09949E-06 1.62306E-05 1.05265E-05 SLC6A3/BCL2/CASP3/DPP4/TNF/TP53/NTRK2/CFLAR/TNFRSF10A/CD28/VCP 11

MF GO:0004712 protein serine/threonine/tyrosine kinase activity 7/227 45/18352 1.22886E-06 1.77185E-05 1.14914E-05 AKT1/MAPK1/MAPK3/MAPK14/PRKCG/MAPK10/MAPK9 7

MF GO:0005164 tumor necrosis factor receptor binding 6/227 31/18352 1.90576E-06 2.68539E-05 1.74163E-05 CASP8/TNF/STAT1/FASLG/TNFSF10/CD40LG 6

MF GO:0004993 G protein-coupled serotonin receptor activity 6/227 34/18352 3.37485E-06 4.54872E-05 2.9501E-05 CHRM3/HTR2A/HTR2C/HRH3/HTR1A/HTR2B 6

MF GO:0099589 serotonin receptor activity 6/227 34/18352 3.37485E-06 4.54872E-05 2.9501E-05 CHRM3/HTR2A/HTR2C/HRH3/HTR1A/HTR2B 6

MF GO:0070491 repressing transcription factor binding 8/227 74/18352 3.61821E-06 4.77296E-05 3.09554E-05 BCL2/RELA/PPARG/STAT1/PPARD/MYC/STAT3/PPARA 8

MF GO:0099528 G protein-coupled neurotransmitter receptor activity 4/227 10/18352 4.51602E-06 5.83319E-05 3.78316E-05 CHRM3/GABBR1/GRM1/HRH3 4

MF GO:0033218 amide binding 17/227 381/18352 5.62993E-06 7.12359E-05 4.62005E-05 RXRA/CHRNA7/OPRM1/RELA/PPARG/ACHE/TLR4/CAT/FOLH1/IGF1R/GSTP1/INSR/BCHE/GRIA2/PLA2G4A/BDKRB1/DHFR 17

MF GO:0070513 death domain binding 4/227 11/18352 7.02775E-06 8.71441E-05 5.65179E-05 BCL2/BAX/BCL2L1/MCL1 4

MF GO:0042277 peptide binding 15/227 308/18352 7.18592E-06 8.73582E-05 5.66568E-05 RXRA/CHRNA7/OPRM1/RELA/PPARG/ACHE/TLR4/CAT/FOLH1/IGF1R/GSTP1/INSR/BCHE/GRIA2/BDKRB1 15

MF GO:0051219 phosphoprotein binding 8/227 85/18352 1.02821E-05 0.000122121 7.92025E-05 MAPK1/SNCA/MAPK3/SYK/PLAT/MTOR/PTPN6/TRPV1 8

MF GO:0008353 RNA polymerase II CTD heptapeptide repeat kinase activity 4/227 12/18352 1.04394E-05 0.000122121 7.92025E-05 MAPK1/CDK1/CDK6/CDK7 4

MF GO:0016209 antioxidant activity 8/227 86/18352 1.12173E-05 0.000128791 8.35281E-05 PTGS2/SOD1/CAT/MPO/SOD2/GSTP1/GPX1/GPX4 8

MF GO:0004190 aspartic-type endopeptidase activity 5/227 25/18352 1.20285E-05 0.000133172 8.63698E-05 CASP3/PSEN1/PSEN2/CTSD/REN 5

MF GO:0043027 cysteine-type endopeptidase inhibitor activity involved in apoptotic process 5/227 25/18352 1.20285E-05 0.000133172 8.63698E-05 SNCA/BIRC5/XIAP/BIRC3/BIRC2 5

MF GO:0031406 carboxylic acid binding 12/227 212/18352 1.36839E-05 0.000148843 9.65329E-05 RXRA/PPARG/NOS2/PPARD/GCLC/SELE/GSTP1/PAM/NR1H4/VDR/DHFR/PADI4 12

MF GO:0043028 cysteine-type endopeptidase regulator activity involved in apoptotic process 6/227 43/18352 1.39413E-05 0.000149028 9.66532E-05 SNCA/BIRC5/XIAP/BIRC3/CASP1/BIRC2 6

MF GO:0070001 aspartic-type peptidase activity 5/227 26/18352 1.4743E-05 0.000154926 0.000100478 CASP3/PSEN1/PSEN2/CTSD/REN 5

MF GO:0001046 core promoter sequence-specific DNA binding 6/227 46/18352 2.07677E-05 0.0002146 0.00013918 RELA/FOS/TP53/STAT1/MYC/MMP12 6

MF GO:0001102 RNA polymerase II activating transcription factor binding 6/227 47/18352 2.35628E-05 0.00023674 0.000153539 JUN/FOS/NFE2L2/CREB1/ATF2/EP300 6

MF GO:0043177 organic acid binding 12/227 224/18352 2.3674E-05 0.00023674 0.000153539 RXRA/PPARG/NOS2/PPARD/GCLC/SELE/GSTP1/PAM/NR1H4/VDR/DHFR/PADI4 12

MF GO:0031072 heat shock protein binding 9/227 127/18352 2.89607E-05 0.00028501 0.000184845 BAX/HIF1A/KDR/SNCA/CDK1/CDK5/BAK1/CREB1/EIF2AK3 9

MF GO:0001618 virus receptor activity 7/227 74/18352 3.59628E-05 0.00034303 0.000222475 HTR2A/DPP4/EGFR/CDK1/ICAM1/CD80/CCR5 7

MF GO:0140272 exogenous protein binding 7/227 74/18352 3.59628E-05 0.00034303 0.000222475 HTR2A/DPP4/EGFR/CDK1/ICAM1/CD80/CCR5 7

MF GO:0008227 G protein-coupled amine receptor activity 6/227 51/18352 3.79254E-05 0.000356269 0.000231061 CHRM3/HTR2A/HTR2C/HRH3/HTR1A/HTR2B 6

MF GO:0004601 peroxidase activity 6/227 52/18352 4.24332E-05 0.000392665 0.000254666 PTGS2/CAT/MPO/GSTP1/GPX1/GPX4 6

MF GO:0004222 metalloendopeptidase activity 8/227 108/18352 5.8971E-05 0.000537677 0.000348714 MMP3/MMP2/MMP9/MMP1/MMP12/MMP7/MMP8/MMP10 8

MF GO:0004708 MAP kinase kinase activity 4/227 18/18352 6.08738E-05 0.000546982 0.000354749 MAPK1/MAPK3/MAPK14/MAPK10 4

MF GO:0016684 oxidoreductase activity, acting on peroxide as acceptor 6/227 56/18352 6.49451E-05 0.000575228 0.000373068 PTGS2/CAT/MPO/GSTP1/GPX1/GPX4 6

MF GO:0005507 copper ion binding 6/227 60/18352 9.61056E-05 0.000839232 0.00054429 TP53/SOD1/SNCA/TYR/IL1A/PAM 6

MF GO:0008081 phosphoric diester hydrolase activity 7/227 89/18352 0.000117728 0.001013773 0.000657489 CHRM3/HMOX1/ENPP2/TDP1/CCR1/CCR5/PDE5A 7

MF GO:0098960 postsynaptic neurotransmitter receptor activity 6/227 65/18352 0.000150624 0.00127927 0.000829679 CHRM3/CHRNA7/GABBR1/GRM1/HRH3/DRD2 6

MF GO:0002039 p53 binding 6/227 66/18352 0.000163994 0.001374003 0.000891119 TP53/HIF1A/CDK5/SIRT1/MDM2/EP300 6

MF GO:0016493 C-C chemokine receptor activity 4/227 23/18352 0.000167806 0.001387195 0.000899675 CCR2/CCR1/CCR3/CCR5 4

MF GO:0046982 protein heterodimerization activity 13/227 321/18352 0.000190409 0.001553334 0.001007426 BCL2/BAX/BCL2L1/TP53/HIF1A/TLR4/ATF3/DDIT3/MCL1/BAK1/AGTR1/ERBB2/ABCG1 13

MF GO:0019957 C-C chemokine binding 4/227 24/18352 0.000199423 0.001605743 0.001041416 CCR2/CCR1/CCR3/CCR5 4

MF GO:0048156 tau protein binding 5/227 45/18352 0.0002262 0.001797997 0.001166103 MAP2/SNCA/HSP90AA1/CDK5/EP300 5

MF GO:0033293 monocarboxylic acid binding 6/227 72/18352 0.000265167 0.002081054 0.001349682 RXRA/PPARG/PPARD/GSTP1/NR1H4/VDR 6

MF GO:0001637 G protein-coupled chemoattractant receptor activity 4/227 26/18352 0.000275185 0.002106356 0.001366091 CCR2/CCR1/CCR3/CCR5 4

MF GO:0004950 chemokine receptor activity 4/227 26/18352 0.000275185 0.002106356 0.001366091 CCR2/CCR1/CCR3/CCR5 4

MF GO:0019955 cytokine binding 8/227 135/18352 0.000279097 0.002110247 0.001368615 CCR2/TNFRSF1A/TNFRSF1B/CCR1/CCR3/CSF1R/CCR5/TGFBR1 8

MF GO:0019838 growth factor binding 8/227 136/18352 0.000293435 0.002191925 0.001421588 EGFR/KDR/NTRK1/NTRK2/IGF1R/ERBB2/INSR/TGFBR1 8

MF GO:0020037 heme binding 8/227 138/18352 0.000323918 0.002390825 0.001550586 PTGS2/NOS2/HMOX1/CAT/CYP19A1/MPO/CYP3A4/EIF2AK1 8

MF GO:0035035 histone acetyltransferase binding 4/227 28/18352 0.000369652 0.002693444 0.001746851 TP53/HIF1A/STAT1/CREB1 4

MF GO:0005035 death receptor activity 3/227 12/18352 0.000378348 0.002693444 0.001746851 TNFRSF10A/TNFRSF1A/TNFRSF1B 3

MF GO:0005165 neurotrophin receptor binding 3/227 12/18352 0.000378348 0.002693444 0.001746851 BDNF/NGF/NTRK1 3

MF GO:0001540 amyloid-beta binding 6/227 77/18352 0.000382295 0.002693444 0.001746851 CHRNA7/ACHE/TLR4/INSR/BCHE/GRIA2 6

MF GO:0004693 cyclin-dependent protein serine/threonine kinase activity 4/227 29/18352 0.000424664 0.002925465 0.001897331 CDK1/CDK5/CDK6/CDK7 4

MF GO:0097472 cyclin-dependent protein kinase activity 4/227 29/18352 0.000424664 0.002925465 0.001897331 CDK1/CDK5/CDK6/CDK7 4

MF GO:0005178 integrin binding 8/227 144/18352 0.000431215 0.002937951 0.001905428 PRKCA/EGFR/KDR/IL1B/SYK/ICAM1/VCAM1/CD40LG 8

MF GO:0042166 acetylcholine binding 3/227 13/18352 0.000487373 0.003284469 0.002130165 CHRM3/CHRNA7/ACHE 3

MF GO:0035258 steroid hormone receptor binding 6/227 81/18352 0.0005023 0.003348667 0.002171801 PPARG/ESR1/PARP1/STAT3/PRKCB/EP300 6

MF GO:0008528 G protein-coupled peptide receptor activity 8/227 148/18352 0.00051759 0.003377958 0.002190798 OPRM1/AGTR1/CCR2/CCR1/CCR3/TACR1/BDKRB1/CCR5 8

MF GO:0046906 tetrapyrrole binding 8/227 148/18352 0.00051759 0.003377958 0.002190798 PTGS2/NOS2/HMOX1/CAT/CYP19A1/MPO/CYP3A4/EIF2AK1 8

MF GO:0050661 NADP binding 5/227 54/18352 0.000535032 0.003455413 0.002241032 NOS2/CAT/HMGCR/IDH1/DHFR 5

MF GO:0061134 peptidase regulator activity 10/227 229/18352 0.000584456 0.003735695 0.002422811 SNCA/NGF/APP/BIRC5/XIAP/CFLAR/BIRC3/VCP/CASP1/BIRC2 10

MF GO:0008237 metallopeptidase activity 9/227 189/18352 0.000590787 0.00373763 0.002424065 MMP3/MMP2/MMP9/MMP1/MMP12/MMP7/MMP8/FOLH1/MMP10 9

MF GO:0015562 efflux transmembrane transporter activity 3/227 14/18352 0.000614646 0.003790466 0.002458333 ABCG2/ABCB1/GJA1 3

MF GO:0035497 cAMP response element binding 3/227 14/18352 0.000614646 0.003790466 0.002458333 JUN/CREB1/ATF2 3

MF GO:0001653 peptide receptor activity 8/227 152/18352 0.000617479 0.003790466 0.002458333 OPRM1/AGTR1/CCR2/CCR1/CCR3/TACR1/BDKRB1/CCR5 8

MF GO:0008013 beta-catenin binding 6/227 85/18352 0.000649768 0.003949572 0.002561522 AR/ESR1/FOXO1/GJA1/PSEN1/EP300 6

MF GO:0019956 chemokine binding 4/227 33/18352 0.000703876 0.004236922 0.002747885 CCR2/CCR1/CCR3/CCR5 4

MF GO:0004869 cysteine-type endopeptidase inhibitor activity 5/227 59/18352 0.000805675 0.004803062 0.003115059 SNCA/BIRC5/XIAP/BIRC3/BIRC2 5

MF GO:0001103 RNA polymerase II repressing transcription factor binding 4/227 35/18352 0.000883422 0.005216397 0.00338313 PPARG/PPARD/STAT3/PPARA 4

MF GO:0004697 protein kinase C activity 3/227 16/18352 0.000928488 0.005380023 0.003489251 PRKCA/PRKCB/PRKCG 3

MF GO:0004698 calcium-dependent protein kinase C activity 3/227 16/18352 0.000928488 0.005380023 0.003489251 PRKCA/PRKCB/PRKCG 3

MF GO:0097718 disordered domain specific binding 4/227 36/18352 0.0009843 0.00565061 0.003664742 TP53/HSP90AA1/GJA1/MDM2 4

MF GO:0035173 histone kinase activity 3/227 17/18352 0.001117205 0.006354743 0.004121412 PRKCA/CDK1/PRKCB 3

MF GO:0046965 retinoid X receptor binding 3/227 18/18352 0.001328471 0.007487745 0.004856229 PPARG/NR1H4/VDR 3

MF GO:0016504 peptidase activator activity 4/227 40/18352 0.001469323 0.00820703 0.005322726 APP/CFLAR/VCP/CASP1 4

MF GO:0001091 RNA polymerase II general transcription initiation factor binding 3/227 19/18352 0.001563242 0.008653659 0.00561239 AR/TP53/ESR1 3

MF GO:0045182 translation regulator activity 7/227 139/18352 0.001733913 0.009354117 0.006066677 MTOR/EIF2S1/EIF2AK3/DHFR/EIF2AK4/EIF2AK2/EIF2AK1 7

MF GO:0042379 chemokine receptor binding 5/227 70/18352 0.001745309 0.009354117 0.006066677 STAT1/STAT3/CCL2/CXCL8/CCR2 5

MF GO:0001784 phosphotyrosine residue binding 4/227 42/18352 0.001765212 0.009354117 0.006066677 MAPK1/MAPK3/SYK/PTPN6 4

MF GO:0030544 Hsp70 protein binding 4/227 42/18352 0.001765212 0.009354117 0.006066677 BAX/SNCA/CDK1/CREB1 4

MF GO:0051879 Hsp90 protein binding 4/227 42/18352 0.001765212 0.009354117 0.006066677 HIF1A/KDR/CDK5/EIF2AK3 4

MF GO:0051087 chaperone binding 6/227 104/18352 0.00185707 0.009757484 0.006328283 BAX/TP53/SOD1/BAK1/BIRC5/BIRC2 6
