# Supplementary material for: Study on the Anti-demyelination Mechanism of Bu-Shen-Yi-Sui Capsule in the Central Nervous System Based on Network Pharmacology and Experimental Verification
Source: Mediators Inflamm. 2022 Jul 12;2022:9241261. doi: 10.1155/2022/9241261 (PMC9296285; doi:10.1155/2022/9241261)
Supplement: Supplementary Materials — Table S1: all the potential targets of BSYS Capsule. Table S2: known CNSD-related targets. Table S3: BSYS Capsule shared 227 intersection targets with known CNSD-related targets. Table S4: PPI information of 227 intersection targets in Metascape. Table S5: the degree values of all nodes in the PPI network. Table S6: results for GO pathway enrichment analysis. Table S7: results for KEGG pathway enrichment analysis. Table S8: information of gene-pathway network. Table S9: information of the “active ingredients-intersection targets” network. [file 9241261.f1.zip › Table S7.docx]

ID Description GeneRatio BgRatio pvalue p.adjust qvalue geneID Count

hsa05200 Pathways in cancer 66/208 393/6879 3.42E-32 7.98E-30 4.21E-30 RET/CDKN1A/CXCL8/FASLG/PIK3CG/IGF1R/CASP9/SHH/CASP8/MYC/CASP3/AKT1/BDKRB1/EP300/JAK1/PRKCG/HSP90AA1/PRKCB/MMP1/MMP2/PRKCA/FOS/MMP9/TGFBR1/AR/AGTR1/BIRC5/PPARG/TP53/BIRC2/BIRC3/PPARD/CSF1R/GSTP1/XIAP/PTGS2/HIF1A/FOXO1/EGFR/RELA/CDC42/RXRB/MAPK9/MAPK8/RXRA/ERBB2/MAPK1/RXRG/MAPK3/NTRK1/JUN/TGFB1/NOS2/EGF/STAT1/STAT3/MTOR/NFKB1/VEGFA/MAPK10/IL6/CDK6/BCL2/MDM2/BAX/BCL2L1 66

hsa04151 PI3K-Akt signaling pathway 37/208 345/6879 2.9E-11 6.75E-09 1.42E-10 CSF1R/ATF2/CDKN1A/FASLG/BRCA1/RELA/EGFR/PIK3CG/IGF1R/CASP9/RXRA/MYC/KDR/AKT1/MAPK1/JAK1/MCL1/MAPK3/HSP90AA1/SYK/EGF/INSR/PRKCA/NGF/IL2/MTOR/NFKB1/VEGFA/IL4/IL6/CREB1/CDK6/BCL2/MDM2/TP53/TLR4/BCL2L1 37

hsa04010 MAPK signaling pathway 34/208 253/6879 4.46E-13 1.04E-10 2.74E-12 ATF2/FASLG/TNF/RELA/EGFR/CDC42/MAPK9/MAPK8/MYC/CASP3/AKT1/MAPK1/MAPK3/PRKCG/NTRK1/NTRK2/JUN/TGFB1/PRKCB/EGF/BDNF/PLA2G4A/PRKCA/FOS/NGF/MAPK14/TGFBR1/NFKB1/TNFRSF1A/MAPK10/IL1A/IL1B/DDIT3/TP53 34

hsa04668 TNF signaling pathway 33/208 107/6879 4.67E-24 1.09E-21 1.44E-22 ATF2/CSF2/PTGS2/TNF/RELA/PIK3CG/ICAM1/MAPK9/MAPK8/CASP8/CASP3/CCL2/AKT1/MAPK1/MAPK3/JUN/EDN1/VCAM1/MMP3/CFLAR/FOS/MAPK14/TNFRSF1B/SELE/MMP9/NFKB1/TNFRSF1A/MAPK10/IL6/CREB1/IL1B/BIRC2/BIRC3 33

hsa04066 HIF-1 signaling pathway 27/208 96/6879 1.75E-18 4.08E-16 2.69E-17 CDKN1A/HIF1A/RELA/EGFR/PIK3CG/IGF1R/ERBB2/AKT1/HMOX1/MAPK1/EP300/MAPK3/PRKCG/EDN1/NOS2/PRKCB/EGF/INSR/STAT3/PRKCA/MTOR/NFKB1/VEGFA/IL6/IFNG/BCL2/TLR4 27

hsa04068 FoxO signaling pathway 27/208 134/6879 1.28E-14 2.97E-12 1.21E-13 CDKN1A/FASLG/FOXO1/GRM1/EGFR/PIK3CG/IGF1R/MAPK9/MAPK8/TNFSF10/AKT1/MAPK1/EP300/MAPK3/IL10/TGFB1/EGF/INSR/STAT3/MAPK14/SOD2/SIRT1/TGFBR1/MAPK10/IL6/CAT/MDM2 27

hsa04060 Cytokine-cytokine receptor interaction 26/208 243/6879 5.54E-08 0.0000129 0.000000131 CSF1R/CSF2/CXCL8/FASLG/TNF/TNFSF10/CCL2/CCR5/CCR3/CCR2/IL10/CCR1/TGFB1/TNFRSF10B/TNFRSF10A/TNFRSF1B/TGFBR1/IL2/TNFRSF1A/IL4/IL1A/IL6/CD40LG/IFNG/IL1B/IL17B 26

hsa04014 Ras signaling pathway 24/208 226/6879 0.00000024 0.000056 0.000000509 PRKCG/CSF1R/PRKCB/EGF/INSR/PLA2G4A/FASLG/PRKCA/NGF/EGFR/PIK3CG/RELA/NFKB1/IGF1R/VEGFA/MAPK10/CDC42/MAPK9/MAPK8/KDR/AKT1/MAPK1/BCL2L1/MAPK3 24

hsa04210 Apoptosis 23/208 62/6879 1.25E-18 2.91E-16 2.2E-17 NTRK1/XIAP/TNFRSF10B/FASLG/TNFRSF10A/CFLAR/NGF/TNF/PIK3CG/RELA/NFKB1/TNFRSF1A/CASP9/CASP8/CASP3/TNFSF10/BCL2/BAX/AKT1/TP53/BIRC2/BIRC3/BCL2L1 23

hsa04722 Neurotrophin signaling pathway 22/208 120/6879 4.16E-11 9.7E-09 1.97E-10 NTRK1/NTRK2/JUN/BDNF/PSEN2/FASLG/PSEN1/NGF/MAPK14/PIK3CG/RELA/NFKB1/MAPK10/CDC42/MAPK9/MAPK8/BCL2/BAX/AKT1/MAPK1/TP53/MAPK3 22

hsa04620 Toll-like receptor signaling pathway 20/208 106/6879 2.41E-10 5.62E-08 9.88E-10 JUN/CXCL8/STAT1/CD80/FOS/MAPK14/TNF/PIK3CG/RELA/NFKB1/MAPK10/MAPK9/IL6/MAPK8/CASP8/IL1B/AKT1/MAPK1/TLR4/MAPK3 20

hsa04919 Thyroid hormone signaling pathway 20/208 115/6879 1.03E-09 0.00000024 3.62E-09 PRKCG/PRKCB/STAT1/PRKCA/HIF1A/ESR1/FOXO1/MTOR/PIK3CG/CASP9/RXRB/RXRA/MYC/MDM2/AKT1/EP300/MAPK1/RXRG/TP53/MAPK3 20

hsa05169 Epstein-Barr virus infection 20/208 122/6879 2.89E-09 0.000000673 9.12E-09 CDKN1A/JUN/SYK/STAT3/EIF2AK2/TYK2/MAPK14/PIK3CG/RELA/NFKB1/ICAM1/MAPK10/MAPK9/MAPK8/MYC/MDM2/BCL2/AKT1/TP53/JAK1 20

hsa04064 NF-kappa B signaling pathway 19/208 87/6879 6.04E-11 1.41E-08 2.66E-10 VCAM1/CXCL8/SYK/XIAP/CFLAR/PTGS2/TNF/RELA/NFKB1/ICAM1/TNFRSF1A/CD40LG/PLAU/IL1B/BCL2/TLR4/BIRC2/BIRC3/BCL2L1 19

hsa04660 T cell receptor signaling pathway 19/208 100/6879 6.82E-10 0.000000159 2.54E-09 IL10/JUN/CSF2/FOS/MAPK14/TNF/IL2/PIK3CG/RELA/NFKB1/CDC42/IL4/CD40LG/IFNG/CD28/AKT1/MAPK1/PTPN6/MAPK3 19

hsa04071 Sphingolipid signaling pathway 19/208 120/6879 1.42E-08 0.00000331 3.72E-08 PRKCG/PRKCB/PRKCA/MAPK14/TNF/PIK3CG/RELA/NFKB1/TNFRSF1A/MAPK10/MAPK9/MAPK8/BCL2/BAX/AKT1/MAPK1/CTSD/TP53/MAPK3 19

hsa04024 cAMP signaling pathway 19/208 198/6879 0.0000252 0.005850942 0.0000436 GRIA2/JUN/GABBR1/BDNF/HTR1A/FOS/PIK3CG/RELA/NFKB1/MAPK10/MAPK9/MAPK8/CREB1/AKT1/EP300/MAPK1/DRD2/PPARA/MAPK3 19

hsa04621 NOD-like receptor signaling pathway 18/208 56/6879 2.46E-13 5.73E-11 1.61E-12 HSP90AA1/CXCL8/MAPK14/TNF/RELA/NFKB1/MAPK10/MAPK9/IL6/MAPK8/CASP8/IL1B/CASP1/CCL2/MAPK1/BIRC2/BIRC3/MAPK3 18

hsa05210 Colorectal cancer 18/208 62/6879 1.58E-12 3.69E-10 8.85E-12 JUN/TGFB1/FOS/TGFBR1/PIK3CG/MAPK10/CASP9/MAPK9/MAPK8/MYC/CASP3/BCL2/BAX/BIRC5/AKT1/MAPK1/TP53/MAPK3 18

hsa05222 Small cell lung cancer 18/208 85/6879 3.71E-10 8.64E-08 1.47E-09 NOS2/XIAP/PTGS2/PIK3CG/RELA/NFKB1/CASP9/RXRB/RXRA/CDK6/MYC/BCL2/AKT1/RXRG/TP53/BIRC2/BIRC3/BCL2L1 18

hsa05323 Rheumatoid arthritis 18/208 88/6879 6.58E-10 0.000000153 2.53E-09 JUN/TGFB1/CXCL8/CSF2/MMP1/CD80/MMP3/FOS/TNF/ICAM1/VEGFA/IL1A/IL6/IFNG/IL1B/CD28/CCL2/TLR4 18

hsa04750 Inflammatory mediator regulation of TRP channels 18/208 98/6879 3.76E-09 0.000000876 1.16E-08 PRKCG/NTRK1/PRKCB/HTR2B/PLA2G4A/HTR2C/PRKCA/TRPV1/HTR2A/NGF/MAPK14/PIK3CG/MAPK10/MAPK9/MAPK8/IL1B/TRPV4/BDKRB1 18

hsa04012 ErbB signaling pathway 17/208 87/6879 4.55E-09 0.00000106 1.33E-08 PRKCG/CDKN1A/JUN/PRKCB/EGF/PRKCA/EGFR/MTOR/PIK3CG/MAPK10/MAPK9/MAPK8/MYC/ERBB2/AKT1/MAPK1/MAPK3 17

hsa04915 Estrogen signaling pathway 17/208 99/6879 3.16E-08 0.00000736 7.77E-08 ATF2/JUN/GABBR1/HSP90AA1/MMP2/FOS/OPRM1/ESR1/MMP9/EGFR/PIK3CG/ESR2/GRM1/CREB1/AKT1/MAPK1/MAPK3 17

hsa05231 Choline metabolism in cancer 17/208 101/6879 4.24E-08 0.00000987 0.000000102 PRKCG/JUN/PRKCB/EGF/PLA2G4A/PRKCA/FOS/HIF1A/EGFR/MTOR/PIK3CG/MAPK10/MAPK9/MAPK8/AKT1/MAPK1/MAPK3 17

hsa05146 Amoebiasis 17/208 106/6879 8.55E-08 0.0000199 0.000000198 IL10/PRKCG/TGFB1/CXCL8/CSF2/NOS2/PRKCB/PRKCA/TNF/PIK3CG/RELA/NFKB1/IL6/IFNG/IL1B/CASP3/TLR4 17

hsa04931 Insulin resistance 17/208 108/6879 0.000000112 0.0000261 0.00000025 INSR/STAT3/NR1H3/TNF/FOXO1/MTOR/PIK3CG/RELA/NFKB1/TNFRSF1A/MAPK10/MAPK9/IL6/MAPK8/CREB1/AKT1/PPARA 17

hsa04650 Natural killer cell mediated cytotoxicity 17/208 122/6879 0.000000624 0.000145 0.00000126 PRKCG/CSF2/SYK/PRKCB/TNFRSF10B/FASLG/PRKCA/TNFRSF10A/TNF/PIK3CG/ICAM1/IFNG/CASP3/TNFSF10/MAPK1/PTPN6/MAPK3 17

hsa05223 Non-small cell lung cancer 16/208 56/6879 4.96E-11 1.15E-08 2.26E-10 PRKCG/PRKCB/EGF/PRKCA/EGFR/PIK3CG/CASP9/RXRB/RXRA/CDK6/ERBB2/AKT1/MAPK1/RXRG/TP53/MAPK3 16

hsa04630 Jak-STAT signaling pathway 16/208 145/6879 0.0000272 0.006316014 0.0000458 IL10/CSF2/STAT1/STAT3/TYK2/IL2/PIK3CG/IL4/IL6/IFNG/MYC/AKT1/EP300/PTPN6/JAK1/BCL2L1 16

hsa05321 Inflammatory bowel disease (IBD) 15/208 64/6879 4.08E-09 0.000000952 1.23E-08 IL10/JUN/TGFB1/STAT1/STAT3/TNF/IL2/RELA/NFKB1/IL4/IL1A/IL6/IFNG/IL1B/TLR4 15

hsa05214 Glioma 15/208 65/6879 5.07E-09 0.00000118 1.45E-08 PRKCG/CDKN1A/PRKCB/EGF/PRKCA/EGFR/MTOR/PIK3CG/IGF1R/CDK6/MDM2/AKT1/MAPK1/TP53/MAPK3 15

hsa04664 Fc epsilon RI signaling pathway 15/208 68/6879 9.46E-09 0.0000022 2.59E-08 CSF2/SYK/PRKCB/PLA2G4A/PRKCA/MAPK14/TNF/PIK3CG/MAPK10/IL4/MAPK9/MAPK8/AKT1/MAPK1/MAPK3 15

hsa04920 Adipocytokine signaling pathway 15/208 70/6879 1.41E-08 0.00000328 3.72E-08 STAT3/TNFRSF1B/TNF/MTOR/RELA/NFKB1/TNFRSF1A/MAPK10/RXRB/MAPK9/MAPK8/RXRA/AKT1/PPARA/RXRG 15

hsa04917 Prolactin signaling pathway 15/208 71/6879 1.71E-08 0.00000397 4.37E-08 STAT1/STAT3/FOS/MAPK14/ESR1/PIK3CG/RELA/ESR2/NFKB1/MAPK10/MAPK9/MAPK8/AKT1/MAPK1/MAPK3 15

hsa04540 Gap junction 15/208 88/6879 0.00000029 0.0000676 0.000000605 PRKCG/PRKCB/EGF/HTR2B/HTR2C/PRKCA/HTR2A/EGFR/GRM1/GJA1/CDK1/MAPK1/DRD2/PRKG1/MAPK3 15

hsa04726 Serotonergic synapse 15/208 111/6879 0.00000514 0.001197672 0.00000944 PRKCG/APP/PRKCB/HTR1A/HTR2B/PLA2G4A/HTR2C/PRKCA/HTR2A/PTGS2/SLC6A4/CASP3/ALOX5/MAPK1/MAPK3 15

hsa05219 Bladder cancer 14/208 41/6879 9.56E-11 2.23E-08 4.05E-10 CDKN1A/CXCL8/MMP1/EGF/MMP2/MMP9/EGFR/VEGFA/MYC/ERBB2/MDM2/MAPK1/TP53/MAPK3 14

hsa05144 Malaria 14/208 49/6879 1.15E-09 0.000000268 3.93E-09 IL10/TGFB1/VCAM1/CXCL8/SELE/TNF/ICAM1/IL6/CD40LG/IFNG/IL1B/PECAM1/CCL2/TLR4 14

hsa05014 Amyotrophic lateral sclerosis (ALS) 14/208 50/6879 1.52E-09 0.000000353 4.93E-09 GRIA2/MAPK14/TNFRSF1B/TNF/SOD1/TNFRSF1A/CASP9/CASP3/CAT/CASP1/BCL2/BAX/TP53/BCL2L1 14

hsa04370 VEGF signaling pathway 14/208 61/6879 2.09E-08 0.00000488 5.26E-08 PRKCG/PRKCB/PLA2G4A/PRKCA/PTGS2/MAPK14/PIK3CG/VEGFA/CDC42/CASP9/KDR/AKT1/MAPK1/MAPK3 14

hsa05220 Chronic myeloid leukemia 14/208 72/6879 0.000000169 0.0000393 0.000000364 CDKN1A/TGFB1/TGFBR1/PIK3CG/RELA/NFKB1/CDK6/MYC/MDM2/AKT1/MAPK1/TP53/BCL2L1/MAPK3 14

hsa04912 GnRH signaling pathway 14/208 91/6879 0.00000276 0.000642 0.00000522 JUN/PRKCB/MMP2/PLA2G4A/PRKCA/MAPK14/EGFR/MAPK10/CDC42/MAPK9/MAPK8/GNRH1/MAPK1/MAPK3 14

hsa05143 African trypanosomiasis 12/208 33/6879 1.52E-09 0.000000355 4.93E-09 IL10/PRKCG/IL6/VCAM1/IFNG/PRKCB/IL1B/FASLG/PRKCA/SELE/TNF/ICAM1 12

hsa05134 Legionellosis 12/208 54/6879 0.000000432 0.000101 0.000000886 CASP9/IL6/VCP/CXCL8/CASP8/IL1B/CASP3/CASP1/TNF/TLR4/RELA/NFKB1 12

hsa05230 Central carbon metabolism in cancer 12/208 64/6879 0.00000258 0.0006 0.00000495 NTRK1/RET/MYC/ERBB2/MAPK1/AKT1/HIF1A/TP53/EGFR/MTOR/PIK3CG/MAPK3 12

hsa05120 Epithelial cell signaling in Helicobacter pylori infection 11/208 67/6879 0.0000272 0.006307739 0.0000458 MAPK10/CDC42/MAPK9/JUN/MAPK8/CXCL8/CASP3/MAPK14/RELA/EGFR/NFKB1 11

hsa05216 Thyroid cancer 10/208 29/6879 0.000000101 0.0000235 0.00000023 RXRB/NTRK1/RET/RXRA/MYC/MAPK1/PPARG/RXRG/TP53/MAPK3 10

hsa05030 Cocaine addiction 10/208 49/6879 0.0000123 0.002857324 0.0000219 ATF2/GRIA2/JUN/CREB1/CDK5/BDNF/DRD2/RELA/SLC6A3/NFKB1 10

hsa05213 Endometrial cancer 10/208 52/6879 0.0000203 0.004727011 0.0000357 CASP9/EGF/MYC/ERBB2/MAPK1/AKT1/TP53/EGFR/PIK3CG/MAPK3 10

hsa05221 Acute myeloid leukemia 10/208 56/6879 0.0000377 0.00875179 0.0000627 MYC/STAT3/MAPK1/AKT1/RELA/NFKB1/MTOR/PIK3CG/PPARD/MAPK3 10

hsa05332 Graft-versus-host disease 9/208 33/6879 0.00000427 0.000993 0.00000795 IL1A/IL6/IFNG/IL1B/CD80/CD28/FASLG/TNF/IL2 9

hsa05330 Allograft rejection 9/208 37/6879 0.0000107 0.002488121 0.0000193 IL10/IL4/CD40LG/IFNG/CD80/CD28/FASLG/TNF/IL2 9
