# Supplementary material for: Study on the Anti-demyelination Mechanism of Bu-Shen-Yi-Sui Capsule in the Central Nervous System Based on Network Pharmacology and Experimental Verification
Source: Mediators Inflamm. 2022 Jul 12;2022:9241261. doi: 10.1155/2022/9241261 (PMC9296285; doi:10.1155/2022/9241261)
Supplement: Supplementary Materials — Table S1: all the potential targets of BSYS Capsule. Table S2: known CNSD-related targets. Table S3: BSYS Capsule shared 227 intersection targets with known CNSD-related targets. Table S4: PPI information of 227 intersection targets in Metascape. Table S5: the degree values of all nodes in the PPI network. Table S6: results for GO pathway enrichment analysis. Table S7: results for KEGG pathway enrichment analysis. Table S8: information of gene-pathway network. Table S9: information of the “active ingredients-intersection targets” network. [file 9241261.f1.zip › Table S8.docx]

SUID Type Gene Symbol Degree

217 gene AKT1 15

180 gene MAPK3 15

182 gene MAPK1 15

224 gene PIK3CG 15

171 gene NFKB1 14

189 gene RELA 14

169 gene MAPK10 11

185 gene MAPK8 11

186 gene MAPK9 11

145 gene MAPK14 10

149 gene TNF 9

208 gene PRKCA 9

168 gene IL6 8

211 gene PRKCB 8

213 gene PRKCG 8

225 gene FASLG 8

142 gene IL1B 7

155 gene NGF 7

166 gene BCL2 7

175 gene EGF 7

178 gene JUN 7

190 gene EGFR 7

200 gene TP53 7

144 gene TNFRSF1A 6

188 gene CDC42 6

207 gene FOS 6

223 gene IGF1R 6

156 gene INSR 5

163 gene BCL2L1 5

170 gene VEGFA 5

179 gene NTRK1 5

196 gene CSF1R 5

198 gene BIRC3 5

199 gene BIRC2 5

215 gene EP300 5

220 gene CASP8 5

226 gene CXCL8 5

151 gene TLR4 4

164 gene BAX 4

165 gene MDM2 4

172 gene MTOR 4

177 gene TGFB1 4

205 gene TGFBR1 4

218 gene CASP3 4

219 gene MYC 4

222 gene CASP9 4

227 gene CDKN1A 4

91 gene DRD2 3

113 gene CD40LG 3

124 gene IL10 3

125 gene TNFSF10 3

128 gene IFNG 3

133 gene CFLAR 3

137 gene CCL2 3

139 gene CSF2 3

146 gene PLA2G4A 3

147 gene BDNF 3

152 gene CREB1 3

153 gene IL4 3

154 gene IL2 3

159 gene KDR 3

161 gene ATF2 3

173 gene STAT3 3

174 gene STAT1 3

184 gene RXRA 3

191 gene FOXO1 3

192 gene HIF1A 3

193 gene PTGS2 3

194 gene XIAP 3

212 gene HSP90AA1 3

216 gene BDKRB1 3

81 gene HTR2A 2

83 gene TRPV1 2

84 gene HTR2C 2

85 gene HTR2B 2

92 gene HTR1A 2

93 gene GABBR1 2

94 gene GRIA2 2

114 gene TNFRSF10A 2

115 gene TNFRSF10B 2

126 gene GRM1 2

132 gene TNFRSF1B 2

135 gene VCAM1 2

136 gene EDN1 2

138 gene ICAM1 2

143 gene IL1A 2

148 gene NTRK2 2

157 gene SYK 2

167 gene CDK6 2

176 gene NOS2 2

181 gene RXRG 2

183 gene ERBB2 2

187 gene RXRB 2

203 gene AGTR1 2

206 gene MMP9 2

214 gene JAK1 2

73 gene TRPV4 1

75 gene CASP1 1

77 gene PRSS3 1

78 gene HRH3 1

79 gene F2 1

80 gene OPRM1 1

82 gene TACR1 1

86 gene C5AR1 1

87 gene CHRNA7 1

88 gene CHRM3 1

90 gene PPARA 1

96 gene CTSD 1

98 gene PTPN6 1

99 gene CD28 1

101 gene PLAU 1

103 gene ESR1 1

105 gene CD80 1

107 gene PSEN1 1

108 gene PSEN2 1

112 gene IL17B 1

116 gene CCR1 1

117 gene CCR2 1

118 gene CCR3 1

119 gene CCR5 1

121 gene CAT 1

122 gene SIRT1 1

123 gene SOD2 1

129 gene HMOX1 1

131 gene SELE 1

134 gene MMP3 1

141 gene DDIT3 1

158 gene MCL1 1

160 gene BRCA1 1

195 gene GSTP1 1

197 gene PPARD 1

201 gene PPARG 1

202 gene BIRC5 1

204 gene AR 1

209 gene MMP2 1

210 gene MMP1 1

221 gene SHH 1

228 gene RET 1
