# Supplementary material for: Study on the Anti-demyelination Mechanism of Bu-Shen-Yi-Sui Capsule in the Central Nervous System Based on Network Pharmacology and Experimental Verification
Source: Mediators Inflamm. 2022 Jul 12;2022:9241261. doi: 10.1155/2022/9241261 (PMC9296285; doi:10.1155/2022/9241261)
Supplement: Supplementary Materials — Table S1: all the potential targets of BSYS Capsule. Table S2: known CNSD-related targets. Table S3: BSYS Capsule shared 227 intersection targets with known CNSD-related targets. Table S4: PPI information of 227 intersection targets in Metascape. Table S5: the degree values of all nodes in the PPI network. Table S6: results for GO pathway enrichment analysis. Table S7: results for KEGG pathway enrichment analysis. Table S8: information of gene-pathway network. Table S9: information of the “active ingredients-intersection targets” network. [file 9241261.f1.zip › Table S9.docx]

name Type Degree AverageShortestPathLength BetweennessCentrality ClosenessCentrality Eccentricity NeighborhoodConnectivity NumberOfDirectedEdges Radiality Stress TopologicalCoefficient

MOL012744 Heshouwu 83 2.30526316 0.36837489 0.43378995 5 3.98795181 83 0.85497076 622928 0.05858729

MOL000511 Shuizhi 39 2.86666667 0.09548862 0.34883721 7 5.43589744 39 0.79259259 276084 0.10316041

MOL000098 multiDrug 39 2.67017544 0.07981105 0.37450723 5 7.15384615 39 0.81442495 266052 0.1309329

MOL000006 Lianqiao 37 2.65614035 0.10506673 0.37648613 5 6.21621622 37 0.81598441 221806 0.10432432

MOL004444 Zhebeimu 31 2.87368421 0.13133971 0.34798535 5 3.64516129 31 0.79181287 251128 0.07779886

MOL004450 Zhebeimu 26 3.1754386 0.15445087 0.31491713 6 2.26923077 26 0.7582846 388850 0.05769231

Citreorosein Heshouwu 24 3.14035088 0.0627313 0.31843575 7 4.25 24 0.76218324 143306 0.11607143

MOL003333 multiDrug 23 2.91578947 0.071908 0.34296029 7 6.30434783 23 0.7871345 153522 0.10608696

MOL001439 Yimucao 21 3.02105263 0.08456042 0.33101045 5 5 21 0.7754386 165524 0.1

MOL003734 Dihuang 18 3.02105263 0.05510289 0.33101045 5 4.5 18 0.7754386 94690 0.13461538

MOL000519 multiDrug 18 3.02105263 0.02798612 0.33101045 7 8.16666667 18 0.7754386 103608 0.15925926

MOL000358 multiDrug 18 3.00701754 0.0280521 0.33255543 7 8.22222222 18 0.77699805 101260 0.15700483

crocetinate Shuizhi 17 3.26666667 0.02058701 0.30612245 7 6.17647059 17 0.74814815 58152 0.23529412

MOL000422 Yimucao 15 3.07017544 0.00968918 0.32571429 7 10.66666667 15 0.76998051 41206 0.2248062

MOL000354 Yimucao 13 3.00701754 0.01943048 0.33255543 5 9.07692308 13 0.77699805 54144 0.20192308

MOL001004 Zhebeimu 13 2.95087719 0.01462401 0.33888228 5 8.46153846 13 0.78323587 40904 0.17765568

MOL002819 Dihuang 12 2.97894737 0.01772944 0.33568905 5 8.91666667 12 0.78011696 39710 0.18849206

MOL000791 Lianqiao 12 3.12631579 0.04188187 0.31986532 5 5.66666667 12 0.76374269 60474 0.12962963

MOL004440 Zhebeimu 11 3.49122807 0.02675564 0.28643216 6 2.72727273 11 0.72319688 54158 0.15702479

MOL000471 Dahuang 10 3.14736842 0.00534591 0.31772575 7 10.6 10 0.76140351 15192 0.24

MOL004082 Quanxie 9 3.60350877 0.03845474 0.2775073 7 2.11111111 9 0.71072125 38738 0.11111111

MOL002083 Heshouwu 8 3.07719298 0.00659838 0.32497149 5 10.25 8 0.76920078 19782 0.24342105

MOL003730 Dihuang 7 3.54035088 0.01232156 0.28245788 5 3.71428571 7 0.71773879 28368 0.27142857

MOL003727 Dihuang 7 3.54035088 0.01232156 0.28245788 5 3.71428571 7 0.71773879 28368 0.27142857

MOL000513 Heshouwu 7 3.18947368 0.00682635 0.31353135 7 12.14285714 7 0.75672515 30064 0.25913621

MOL000295 Tianma 7 3.49122807 0.01225992 0.28643216 7 3.42857143 7 0.72319688 19320 0.18681319

MOL003370 Lianqiao 7 3.2245614 0.00949441 0.3101197 7 10.71428571 7 0.75282651 14808 0.2556391

MOL002235 Dahuang 6 3.1122807 0.00999586 0.32130778 5 10.5 6 0.76530214 14842 0.25675676

MOL002281 Dahuang 5 3.38596491 0.00150945 0.29533679 7 13.2 5 0.73489279 4736 0.33888889

MOL000003 Shuizhi 5 3.79298246 0.01003266 0.26364477 7 3 5 0.68966862 14978 0.22222222

MOL002156 Quanxie 5 5.10526316 0.02792192 0.19587629 8 1.2 5 0.54385965 70428 0.2

MOL001418 Yimucao 5 3.1754386 0.00448738 0.31491713 5 11.2 5 0.7582846 11052 0.28333333

MOL004443 Zhebeimu 5 3.66666667 0.00952833 0.27272727 5 4.4 5 0.7037037 21786 0.2125

MOL000635 Tianma 4 3.16140351 0.00143414 0.31631521 5 14.75 4 0.75984405 7768 0.36184211

MOL003290 Lianqiao 4 3.40701754 0.00166782 0.29351184 7 12.75 4 0.73255361 4350 0.33571429

MOL003347 Lianqiao 4 3.99649123 0.01405331 0.25021949 7 2.25 4 0.66705653 20968 0.3125

MOL002268 multiDrug 3 3.44912281 3.33E-04 0.28992879 7 18.33333333 3 0.72787524 1198 0.4952381

MOL000096 Dahuang 3 3.41403509 6.79E-04 0.29290853 7 16.66666667 3 0.73177388 2176 0.44761905

MOL00051 Heshouwu 3 3.6245614 4.39E-04 0.27589545 7 6 3 0.70838207 866 0.41666667

MOL007986 Tianma 3 4.35438596 0.00722696 0.22965351 7 2.33333333 3 0.62729045 8834 0.33333333

MOL001406 Shuizhi 3 3.47017544 7.37E-04 0.28816987 7 15 3 0.72553606 2444 0.41176471

MOL001421 Yimucao 3 3.47017544 7.10E-04 0.28816987 7 15.33333333 3 0.72553606 2478 0.42156863

MOL001422 Yimucao 3 3.47017544 0.00158003 0.28816987 7 14.66666667 3 0.72553606 2418 0.40196078

MOL003295 Lianqiao 3 3.47017544 0.00714592 0.28816987 7 14.33333333 3 0.72553606 8158 0.39215686

MOL003726 Dihuang 2 3.65263158 2.55E-04 0.27377522 5 6.5 2 0.70526316 992 0.6875

rehmannioside聽D Dihuang 2 3.61052632 7.86E-04 0.27696793 7 8.5 2 0.70994152 1720 0.5

MOL004557 Shuizhi 2 3.58947368 1.26E-04 0.27859238 7 9 2 0.7122807 350 0.57142857

MOL000953 quanxie 2 4.03157895 3.37E-04 0.24804178 7 5.5 2 0.66315789 1500 0.5

MOL001420 Yimucao 2 3.44912281 0.00138866 0.28992879 7 22 2 0.72787524 6326 0.55263158

MOL003330 Lianqiao 2 3.47719298 2.66E-04 0.28758829 7 21 2 0.72475634 1518 0.58823529

MOL000522 Lianqiao 2 3.19649123 0.00122077 0.31284303 5 20 2 0.75594542 5102 0.52777778

MOL002297 Dahuang 1 4.73333333 0 0.21126761 9 9 1 0.58518519 0 0

MOL000702 Heshouwu 1 3.48421053 0 0.28700906 7 35 1 0.72397661 0 0

MOL008647 Heshouwu 1 4.57894737 0 0.2183908 7 3 1 0.60233918 0 0

MOL002320 Heshouwu 1 4.73333333 0 0.21126761 9 9 1 0.58518519 0 0

MOL003306 Lianqiao 1 3.48421053 0 0.28700906 7 35 1 0.72397661 0 0

MOL003308 Lianqiao 1 3.48421053 0 0.28700906 7 35 1 0.72397661 0 0

MOL000211 Lianqiao 1 4.73333333 0 0.21126761 9 9 1 0.58518519 0 0

MOL003322 Lianqiao 1 3.48421053 0 0.28700906 7 35 1 0.72397661 0 0
